# Supplementary material for: Histone methyltransferases MLL2 and SETD1A/B play distinct roles in H3K4me3 deposition during the transition from totipotency to pluripotency
Source: EMBO J. 2024 Dec 5;44(2):437–56. doi: 10.1038/s44318-024-00329-5 (PMC11730331; doi:10.1038/s44318-024-00329-5)
Supplement: Supplementary file 2 — Appendix [file 44318_2024_329_MOESM2_ESM.pdf]

Appendix File

Table of Contents

Appendix Figure S1. Evaluation of knockdown efficiency for Mll2, Setd1a and Setd1b in early embryos. 2

Appendix Figure S2. Analysis of H3K4me3 dynamics in early embryos with *Mll2* and *Setd1a/b* knockdown. 4

Appendix Figure S3. Comparative analysis of H3K4me3, H3K27ac, H3K9ac, and H2A.Z in early embryos. 6

Appendix Figure S4. Immunostaining assesses changes in transcription and H3K4me3 levels following Triptolide treatment. 7

Appendix Figure S5. Impact of Pol II and H3K4me3 enrichment levels following Trp treatment. 9

Appendix Figure S6. Knocking down of *Mll2* does not obstruct ZGA and early embryonic development. 11

Appendix Figure S7. Efficient knockdown of *Mll2* in mouse early embryos utilizing three additional siRNAs. 13

Appendix Figure S8. Overexpression of *Kdm5b* does not inhibit ZGA. 15

Appendix Figure S9. KDM5B inhibition from zygote to Late2C does not block ZGA. 17

Appendix Figure S10. Effect of *Setd1a/b* KD on cell fate commitment. 20

Appendix Figure S11. Immunostaining of SETD1A and SETD1B proteins at the blastocyst stage. 22

Appendix Figure S12. Effect of *Kdm5b* KD on cell fate commitment. 23

Appendix Figure S13. The catalytic role of KDM5B in the first lineage segregation. 25

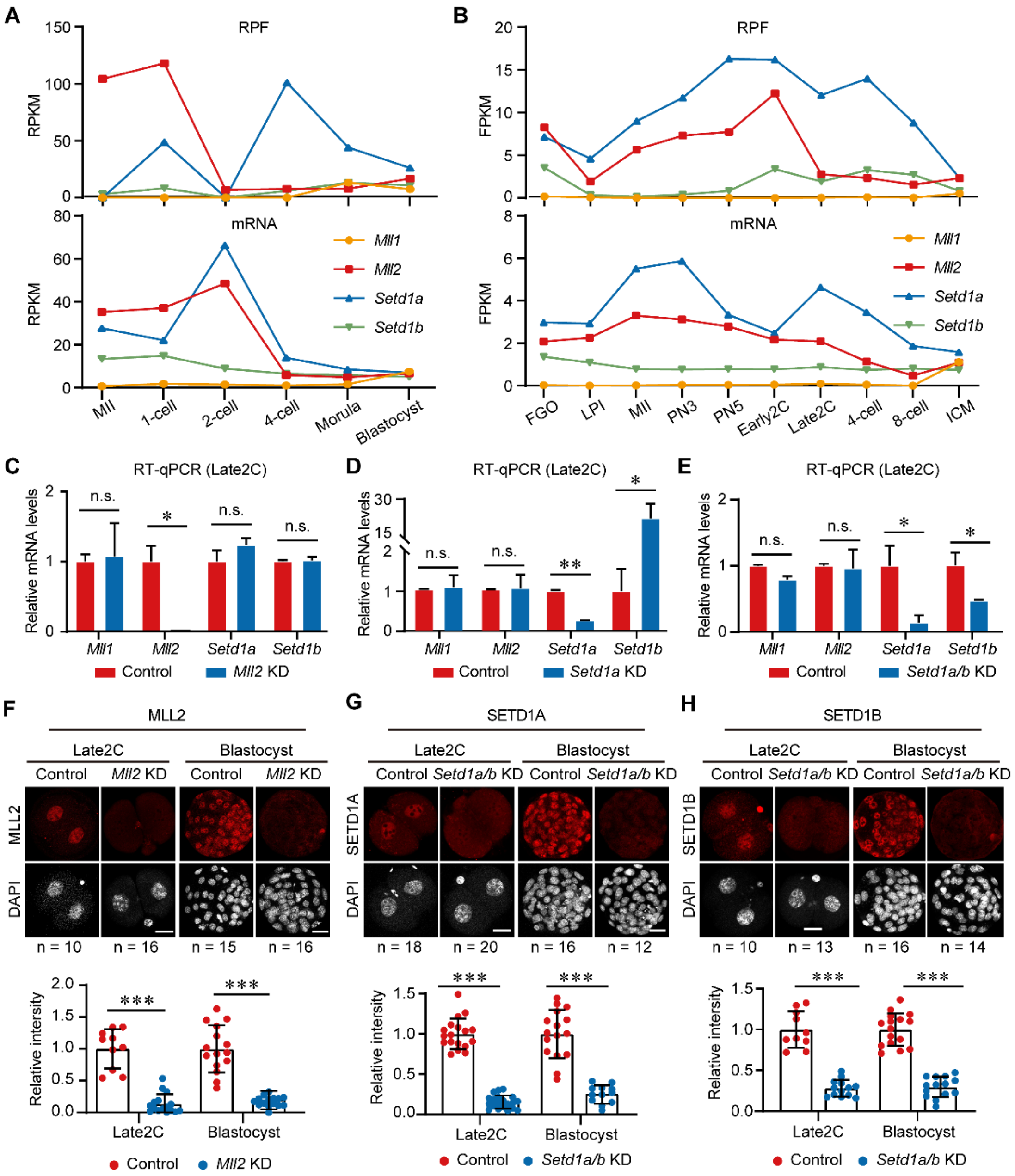

Appendix Figure S1. Evaluation of knockdown efficiency for Mll2, Setd1a and Setd1b in early embryos.

(A-B) Line graphs illustrate Ribo-seq (RPF) and mRNA-seq (mRNA) levels for *Mll1*, *Mll2, Setd1a* and *Setd1b* in mouse oocytes and early embryos, based on Ribo-lite and mRNA-seq data from GSE169632(Zhang *et al*, 2022) **(A)** and GSE165782(Xiong *et al*, 2022) **(B)**. **(C-D)** Bar charts showing the relative expression levels of *Mll1*, *Mll2*, *Setd1a* and *Setd1b* at the late 2-cell (Late2C) stage after knocking down of *Mll2* **(C)**, *Setd1a* **(D)**, or *Setd1a/b* **(E)** in MII oocytes, as detected by RT-qPCR. Error bars represent mean ± standard deviation (SD) from two biological replicates. n.s.,

no significance; *$P < 0.05$; **$P < 0.01$; two-sided unpaired Student's *t*-test (**C:** *Mll1* $P = 0.88$, *Mll2* $P = 0.021$, *Setd1a* $P = 0.21$, *Setd1b* $P = 0.59$; **D:** *Mll1* $P = 0.85$, *Mll2* $P = 0.73$, *Setd1a* $P = 0.0020$, *Setd1b* $P = 0.037$; **E:** *Mll1* $P = 0.21$, *Mll2* $P = 0.74$, *Setd1a* $P = 0.041$, *Setd1b* $P = 0.020$). **(F)** Immunostaining for MLL2 (red) and DNA (gray) in Control and *Mll2* KD embryos at the Late2C and blastocyst stage is shown (Up). The down panel quantifies the relative intensities of MLL2. Error bars represent mean ± SD. ***$P < 0.001$; two-sided unpaired Student's *t*-test (Late2C P = 1.2E-09; Blastocyst P = 1.1E-08). **(G)** Immunostaining for SETD1A (red) and DNA (gray) in Control and *Setd1a/b* KD embryos at the Late2C and blastocyst stage is shown (Up). The down panel quantifies the relative intensities of SETD1A. Error bars represent mean ± SD. ***$P < 0.001$; two-sided unpaired Student's *t*-test (Late2C P = 9.4E-20; Blastocyst P = 2.5E-07). **(H)** Immunostaining for SETD1B (red) and DNA (gray) in Control and *Setd1a/b* KD embryos at the Late2C and blastocyst stage is shown (Up). The down panel quantifies the relative intensities of SETD1B. Error bars represent mean ± SD. ***$P < 0.001$; two-sided unpaired Student's *t*-test (Late2C P = 1.2E-09; Blastocyst P = 3.4E-12).

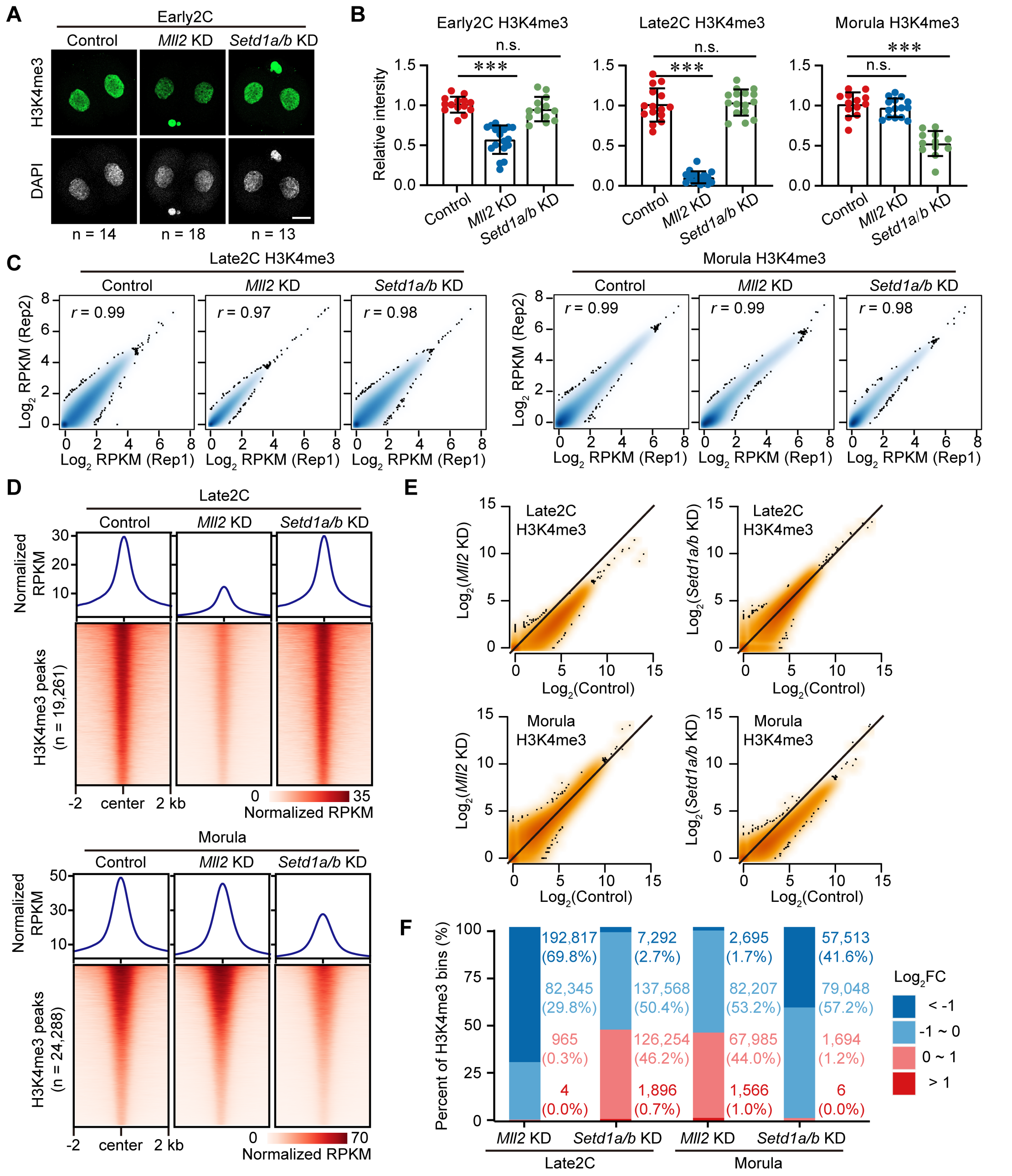

Appendix Figure S2. Analysis of H3K4me3 dynamics in early embryos with *Mll2* and *Setd1a/b* knockdown.

(A) Immunostaining showcases H3K4me3 (green) and DNA (gray) in the Control, *Mll2* knockdown (KD) and *Setd1a/b* KD embryos at the early 2-cell (Early2C) stage. Scale bar, 20 μm. Detailed quantifications in Figure S2B. **(B)** Immunostaining quantification of H3K4me3 relative intensities was conducted in Control, *Mll2* KD, and *Setd1a/b* KD embryos at the early 2-cell (Early2C), Late2C, and morula stages. The number of embryos examined (n) is specified in Figure 1B and Appendix Figure S2A. Error bars represent mean ± SD. n.s., no significance; ***$P < 0.001$; two-sided unpaired

Student's *t*-test (Early2C: Control vs *Mll2* KD *P* = 5.4E-09, Control vs *Setd1a/b* KD *P* = 0.29; Late2C: Control vs *Mll2* KD *P* = 9.4E-17, Control vs *Setd1a/b* KD *P* = 0.56; Morula: Control vs *Mll2* KD *P* = 0.28, Control vs *Setd1a/b* KD *P* = 3.1E-08). **(C)** Scatter plots illustrate correlations between biological replicates of H3K4me3 CUT&Tag data in Control, *Mll2* KD, and *Setd1a/b* KD embryos at the Late2C and morula stages. H3K4me3 enrichment was calculated as reads per kilobase of bin per million mapped reads (RPKM) using 5-kb bins (n = 546,206). Pearson correlation coefficients are shown in the top-left panel. **(D)** H3K4me3 CUT&Tag signals within a ± 2 kb region around the peak centers were analyzed for Late2C embryos (n = 19,261) and morulae (n = 24,288) in Control, *Mll2* KD, and *Setd1a/b* KD groups. Data rows are ranked by descending H3K4me3 enrichment (normalized RPKM value), with two replicates per sample. **(E)** Scatter plots depict alterations in genome-wide H3K4me3 enrichment levels within 5-kb bins (n = 546,206) following *Mll2* KD or *Setd1a/b* KD during the Late2C and morula stages. **(F)** Bar charts display the distribution of 5-kb H3K4me3 bins across different categories of fold change following *Mll2* or *Setd1a/b* KD during the Late2C and morula stages.

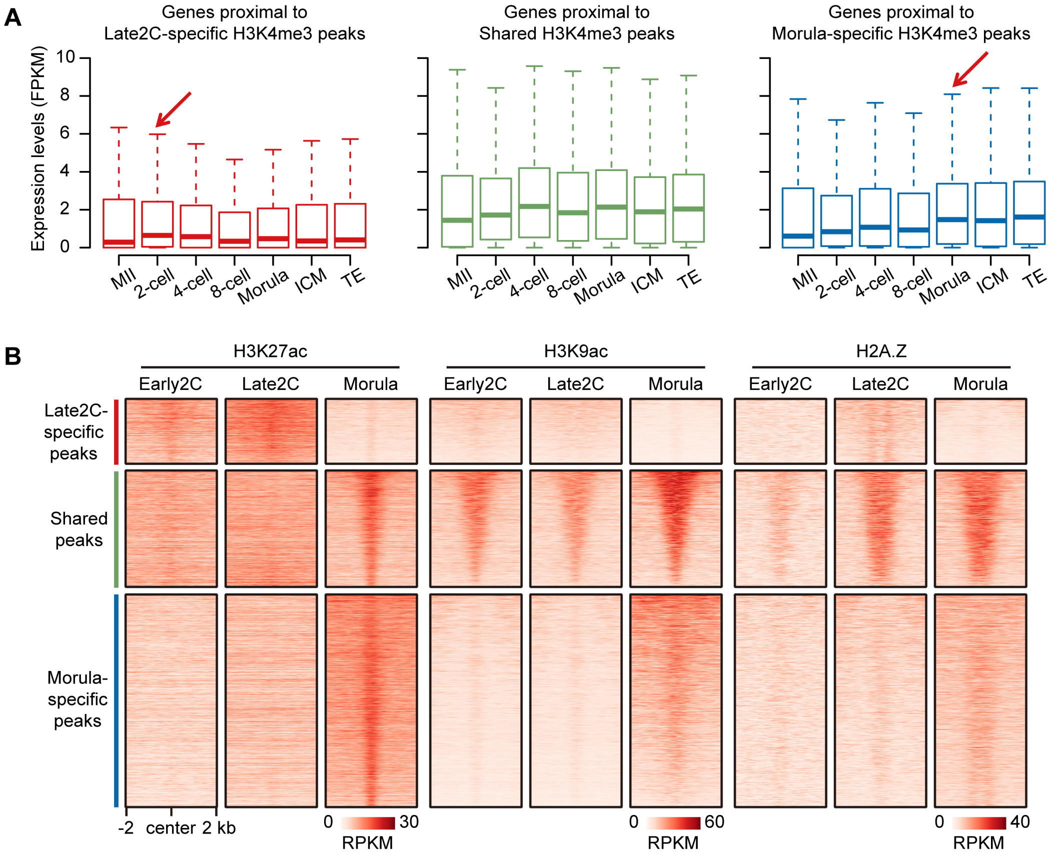

Appendix Figure S3. Comparative analysis of H3K4me3, H3K27ac, H3K9ac, and H2A.Z in early embryos.

(A) Box plots showing the RNA expression levels across different early developmental stages of Late2C-specific (n = 1,586), shared (n = 2,856) and morula-specific (n = 5,175) H3K4me3 peaks. RNA-seq data is from GSE98150(Wang *et al*, 2018). Boxplot was used to display data distribution, with the median as the central line, the box showing the IQR from the 25th to 75th percentile, and whiskers extending to data points within 1.5 times the IQR. **(B)** Heatmaps showing the H3K27ac, H3K9ac and H2A.Z enrichment at the Early2C, Late2C and morula stages with Late2C-specific (n = 1,586), shared (n = 2,856) and morula-specific (n = 5,175) H3K4me3 peaks.

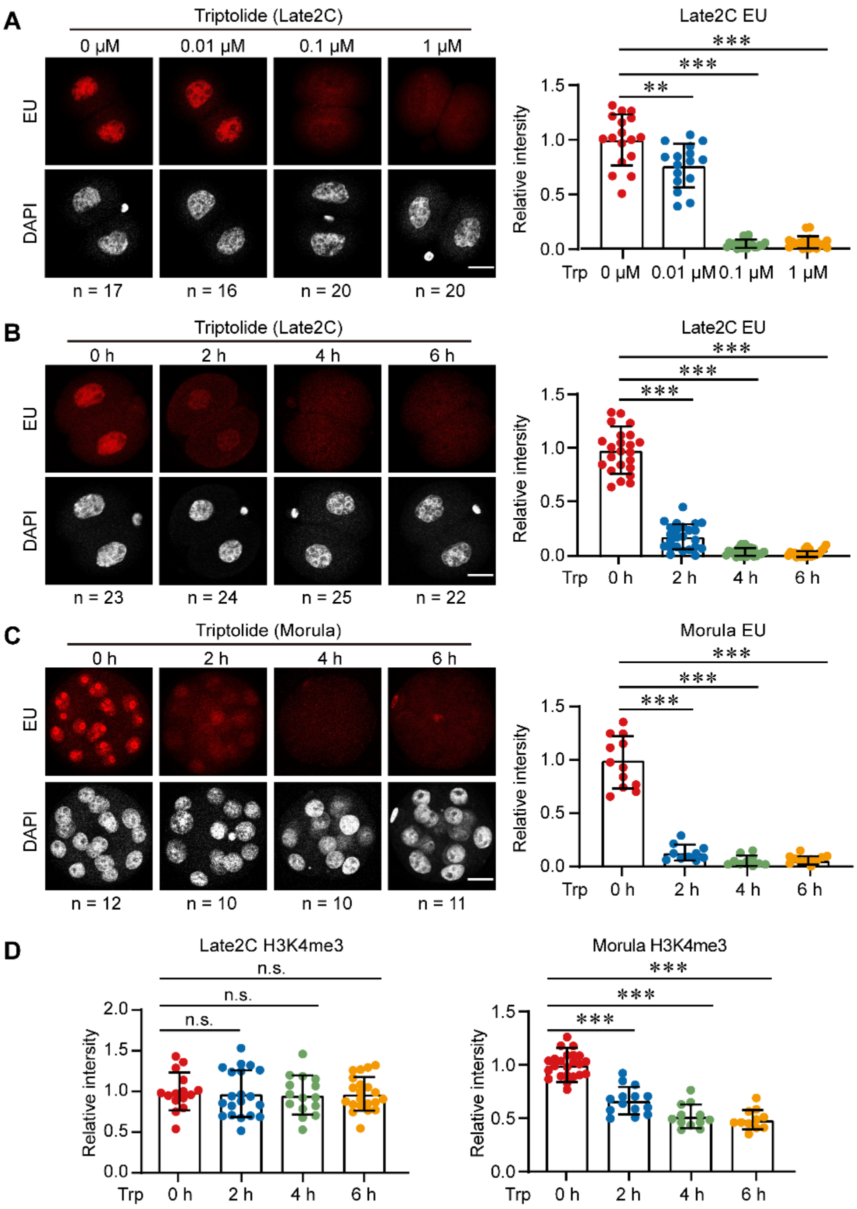

Appendix Figure S4. Immunostaining assesses changes in transcription and H3K4me3 levels following Triptolide treatment.

(A) Representative images of the 5-ethynyl uridine (EU) staining assay for Late2C embryos treated with different Triptolide (Trp) concentrations (0 μM, 0.01 μM, 0.1 μM, and 1 μM) for 2 h on the left, alongside a quantitative analysis of EU signal intensities on the right panel. Scale bar, 20 μm. Error bars represent mean ± SD. $**P < 0.01$; $***P < 0.001$; two-sided unpaired Student's *t*-test (0 μM vs 0.01 μM P = 0.0041, 0 μM vs 0.1 μM P = 3.7E-19, 0 μM vs 1 μM P = 9.4E-19). **(B)** Representative images of the EU-staining for Late2C embryos treated with Trp at different time points (0, 2, 4, and 6 h) on the left, alongside a quantitative analysis of EU signal intensities on the right panel. Scale bar, 20 μm. Error bars represent mean ± SD. $***P < 0.001$; two-sided unpaired

Student's *t*-test (0 h vs 2 h *P* = 6.0E-20, 0 h vs 4 h *P* = 2.1E-25, 0 h vs 6 h *P* = 9.2E-24). **(C)** Representative images of the EU-staining for morulae treated with Trp at different time points (0, 2, 4, and 6 h) on the left, alongside a quantitative analysis of EU signal intensities on the right panel. Scale bar, 20 μm. Error bars represent mean ± SD. ***$P < 0.001$; two-sided unpaired Student's *t*-test (0 h vs 2 h *P* = 3.4E-09, 0 h vs 4 h *P* = 5.2E-10, 0 h vs 6 h *P* = 1.4E-10). **(D)** Immunostaining quantification of H3K4me3 relative intensities was conducted in Late2C embryos and morulae following Trp treatment for 0, 2, 4, and 6 h. The number of embryos examined (n) is specified in Figure 2C. Error bars represent mean ± SD. n.s., no significance; ***$P < 0.001$; two-sided unpaired Student's *t*-test (Late2C: 0 h vs 2 h *P* = 0.77, 0 h vs 4 h *P* = 0.61, 0 h vs 6 h *P* = 0.69; Morula: 0 h vs 2 h *P* = 8.4E-08, 0 h vs 4 h *P* = 5.4E-11, 0 h vs 6 h *P* = 8.4E-10).

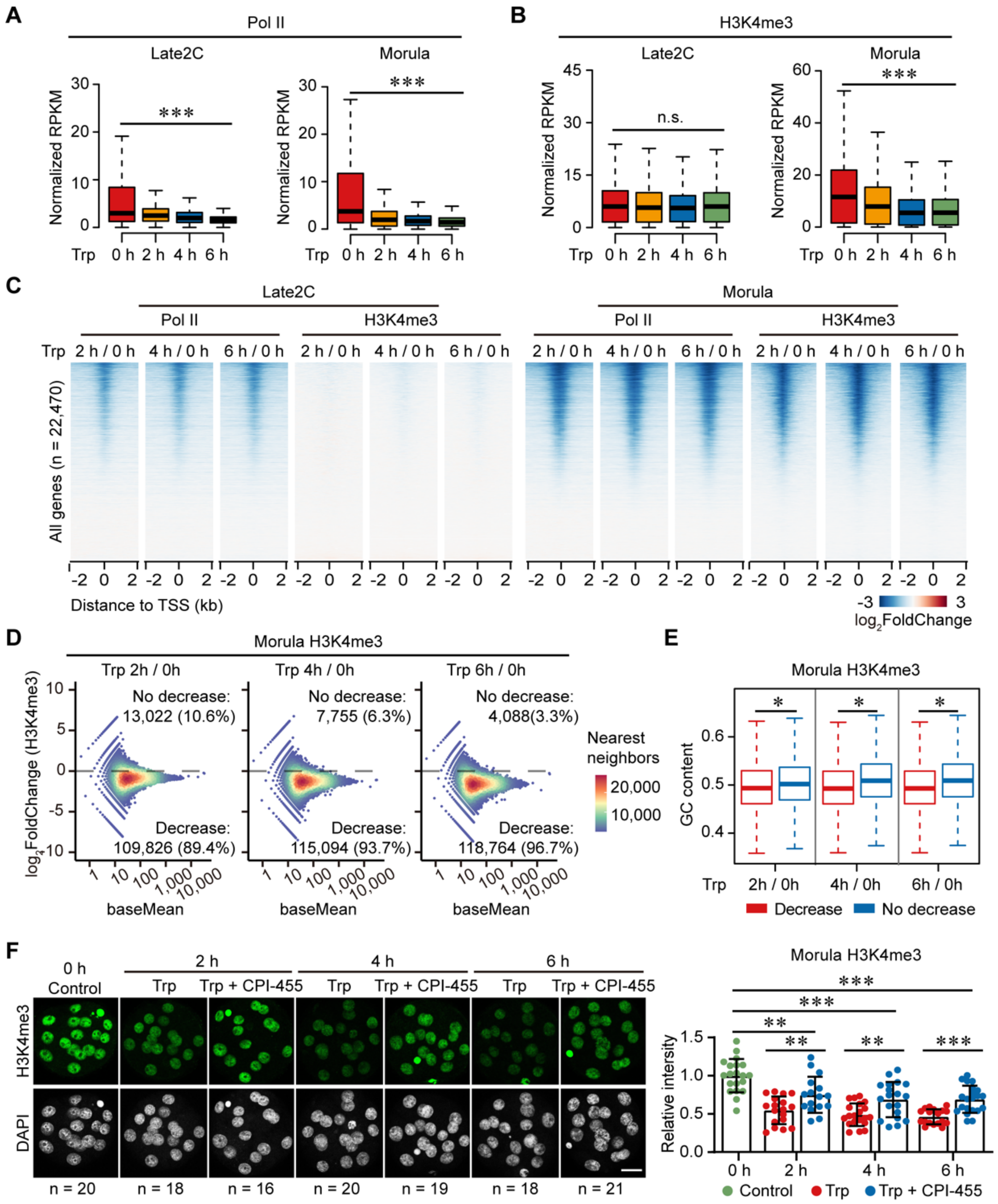

Appendix Figure S5. Impact of Pol II and H3K4me3 enrichment levels following Trp treatment.

(A-B) Box plots showing the enrichment levels of Pol II **(A)** and H3K4me3 **(B)** at the promoter regions (n = 22,470; TSS ± 2 kb) in the Late2C and morula stages after treatment with Trp for 0, 2, 4 and 6 h. Boxplot was used to display data distribution, with the median as the central line, the box showing the IQR from the 25th to 75th percentile, and whiskers extending to data points within 1.5 times the IQR. n.s., no significance; ****P* < 0.001; two-sided Wilcoxon-Mann-Whitney test (**A:** Late2C 0 h vs 2 h *P* < 2.2E-16, Late2C 0 h vs 4 h *P* < 2.2E-16, Late2C 0 h vs 6 h *P* < 2.2E-16,

Morula 0 h vs 2 h *P* < 2.2E-16, Morula 0 h vs 4 h *P* < 2.2E-16, Morula 0 h vs 6 h *P* < 2.2E-16; **B:** Late2C 0 h vs 2 h *P* = 1, Late2C 0 h vs 4 h *P* = 1, Late2C 0 h vs 6 h *P* = 1, Morula 0 h vs 2 h *P* < 2.2E-16, Morula 0 h vs 4 h *P* < 2.2E-16, Morula 0 h vs 6 h *P* < 2.2E-16). **(C)** Heatmaps showing the changes in Pol II and H3K4me3 enrichment at the promoter regions of all genes (n = 22,470), comparing the effect of Trp treatment at 2, 4, and 6 h against the baseline (0 h) for the Late2C (left) and morula (right) stages. Each row represents a promoter region (TSS ± 2 kb), organized by descending Pol II enrichment change. **(D)** MA plots display the decrease in H3K4me3 levels from 0 h to subsequent time points (2, 4, and 6 h) of Trp treatment at the morula stage, with a gray bar indicating a log2 fold-change of 0. The color gradient within these plots shifts from blue to red, reflecting observation density; blue denotes fewer and red more data point concentrations. **(E)** Box plots compare the GC content of H3K4me3 peaks showing a decrease versus no decrease in enrichment following Trp treatment at the morula stage. Boxplot was used to display data distribution, with the median as the central line, the box showing the IQR from the 25th to 75th percentile, and whiskers extending to data points within 1.5 times the IQR. **P* < 0.05; two-sided Wilcoxon-Mann-Whitney test (0 h vs 2 h *P* = 0.041, 0 h vs 4 h *P* = 0.013, 0 h vs 6 h *P* = 0.016). **(F)** Immunostaining shows H3K4me3 (green) and DNA (gray) in morulae treated with Triptolide alone or in combination with CPI-455 for 0, 2, 4, and 6 h on the left, alongside a quantitative analysis of H3K4me3 relative intensities on the right panel. Scale bar, 20 µm. Error bars represent mean ± SD. **P* < 0.01; ***P* < 0.001; two-sided unpaired Student's *t*-test (2 h Trp vs Trp + CPI-455 *P* = 0.008, 4 h Trp vs Trp + CPI-455 *P* = 0.003, 6 h Trp vs Trp + CPI-455 *P* = 6.6E-06, 2 h Trp + CPI-455 vs 0 h *P* = 0.002, 4 h Trp + CPI-455 vs 0 h *P* = 0.0001, 6 h Trp + CPI-455 vs 0 h *P* = 1.4E-05).

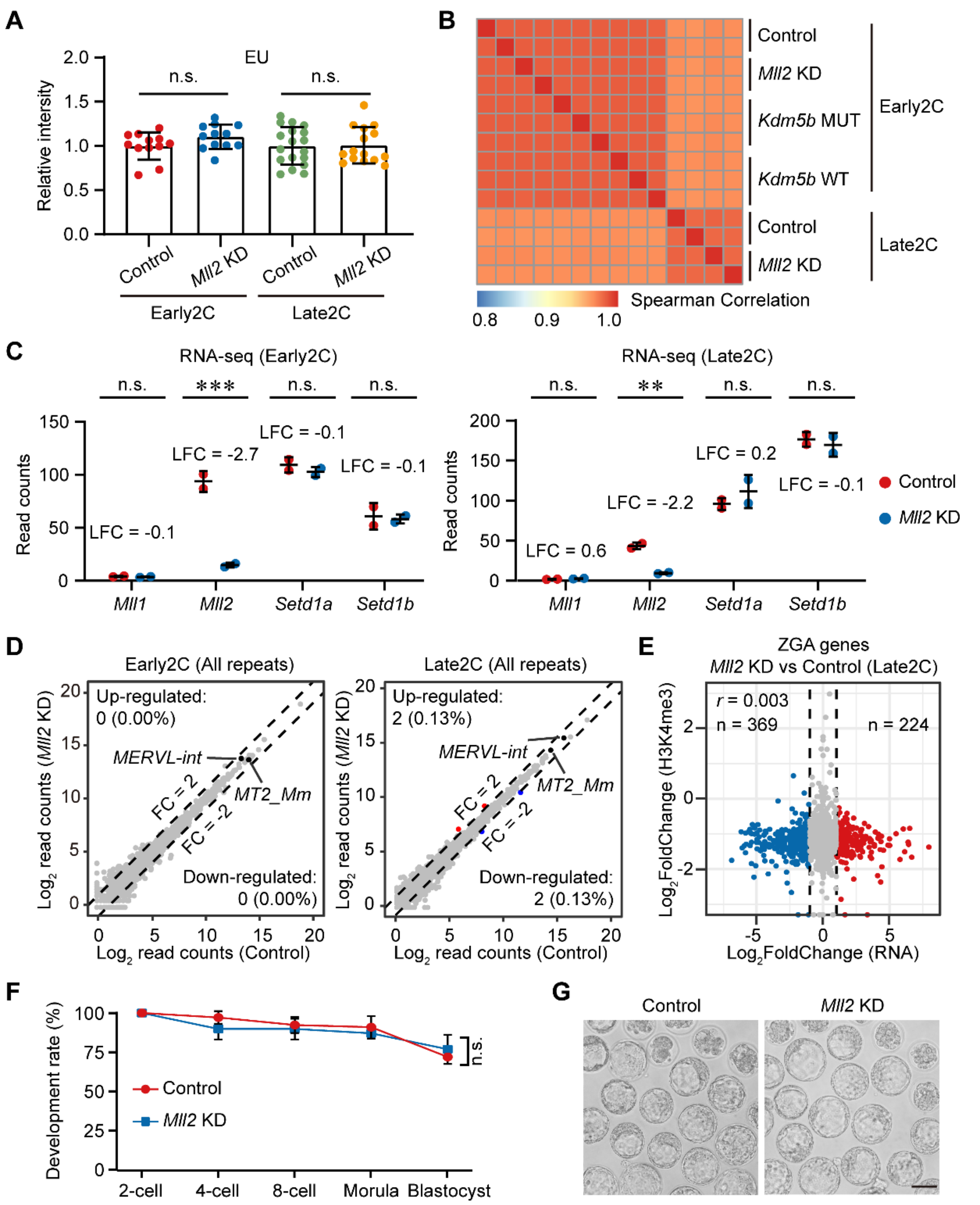

Appendix Figure S6. Knocking down of *Mll2* does not obstruct ZGA and early embryonic development.

(A) Quantitative immunostaining of EU-staining intensities was performed for Control and *Mll2* KD embryos at the Early2C and Late2C stages, with the number of embryos analyzed (n) detailed in Figure 3A. Error bars represent mean ± SD. n.s., no significance; two-sided unpaired Student's *t*-test. (Early2C: Control vs *Mll2* KD $P = 0.10$; Late2C: Control vs *Mll2* KD $P = 0.93$). **(B)** Spearman correlation analysis of RNA levels among Early2C embryos (Control, *Mll2* KD, *Kdm5b* MUT, and

Kdm5b WT), and Late2C embryos (Control and *Mll2* KD). **(C)** Expression levels of *Mll1*, *Mll2*, *Setd1a*, and *Setd1b* at the Early2C and Late2C stages following *Mll2* KD are assessed via RNA-seq. Adjusted *P*-values are computed using DESeq2. Error bars denote mean ± SD from two biological replicates. n.s., no significance; ***P* < 0.01; ****P* < 0.001 (Early2C: *Mll1 P* = 1, *Mll2 P* = 1.29E-05, *Setd1a P* = 1, *Setd1b P* = 1; Late2C: *Mll1 P* =1, *Mll2 P* =0.008, *Setd1a P* = 1, *Setd1b P* = 1). LFC, Log_2 (fold change). **(D)** Scatter plots comparing the repeats expression levels between Control and *Mll2* KD embryos at the Early2C and Late2C stages, highlighting repeats up-regulated in *Mll2* KD embryos (red) and down-regulated in Control embryos (blue). **(E)** Dot plot showcases the correlation between transcriptional changes in ZGA genes associated with H3K4me3 at promoters (n = 2,773) and changes in H3K4me3 ChIP-seq signal in Control embryos versus *Mll2* KD embryos at the Late2C stage. **(F)** Line plots showing the development rate of Control and *Mll2* KD embryos at specified time points. Error bars represent mean ± SD from three biological replicates. n.s., no significance; two-sided unpaired Student's *t*-test (*P* = 0.28). **(G)** Representative images of Control and *Mll2* KD groups at the blastocyst stage. One representative image from three independent experiments is shown. Scale bar, 50 µm.

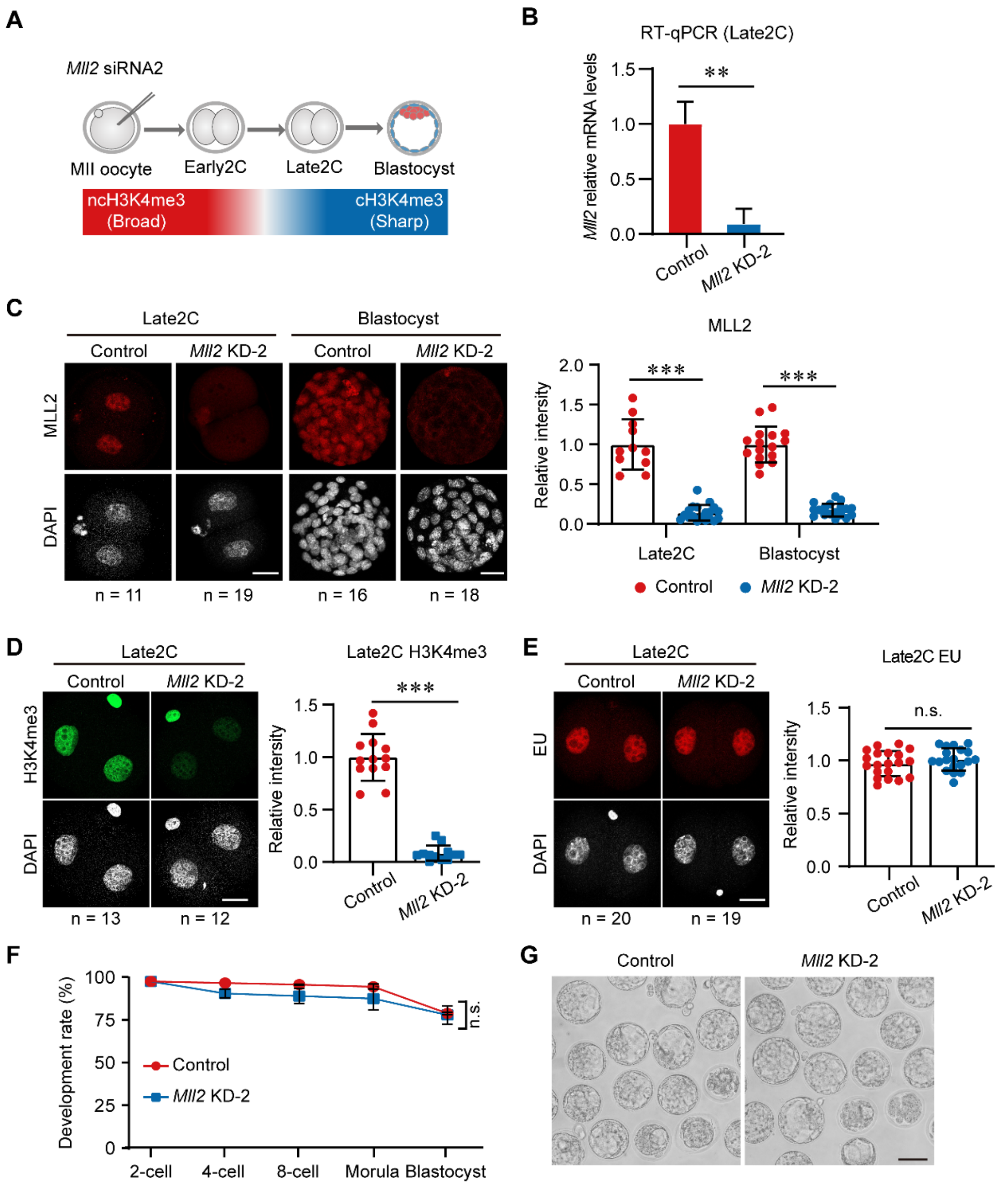

Appendix Figure S7. Efficient knockdown of *Mll2* in mouse early embryos utilizing three additional siRNAs.

(A) Diagram detailing the KD strategy for *Mll2*. This is shown through the schematic representation of microinjecting *Mll2* siRNA2 into MII oocytes to effectuate gene knockdown, followed by the initiating *in vitro* fertilization to generate early embryos. **(B)** Bar chart showing the relative expression levels of *Mll2* following *Mll2* KD at the Late2C stage, as detected by RT-qPCR. Error bars represent mean ± SD from three biological replicates. $^{**}P < 0.01$; two-sided Student's *t*-test ($P = 0.006$). **(C)** Immunostaining for MLL2 (red) and DNA (gray) in Control and *Mll2* KD-2 embryos

at the Late2C and blastocyst stage is shown on the left. The right panel quantifies the relative intensities of MLL2. Scale bar, 20 μm. Error bars represent mean ± SD. ***$P < 0.001$; two-sided Student's *t*-test (Late2C: Control vs *Mll2* KD-2 P = 1.1E-11; Blastocyst: Control vs *Mll2* KD-2 P = 1.1E-15). **(D)** Immunostaining for H3K4me3 (green) and DNA (gray) in Control and *Mll2* KD-2 embryos at the Late2C stage is shown on the left. The right panel quantifies the relative intensities of H3K4me3. Scale bar, 20 μm. Error bars represent mean ± SD. ***$P < 0.001$; two-sided Student's *t*-test (P = 1.8E-12). **(E)** Representative images of the EU-staining for Control and *Mll2* KD-2 embryos at the Late2C stages on the left, alongside a quantitative analysis of EU signal intensities on the right panel. Scale bar, 20 μm. Error bars represent mean ± SD. n.s., no significance; two-sided Student's *t*-test ($P = 0.27$). **(F)** Line plots showing the development rate of Control and *Mll2* KD-2 embryos at specified time points. Error bars represent mean ± SD from three biological replicates. n.s., no significance; two-sided Student's *t*-test ($P = 0.60$). **(G)** Representative images of Control and *Mll2* KD-2 groups at the blastocyst stage. One representative image from three independent experiments is shown. Scale bar, 50 μm.

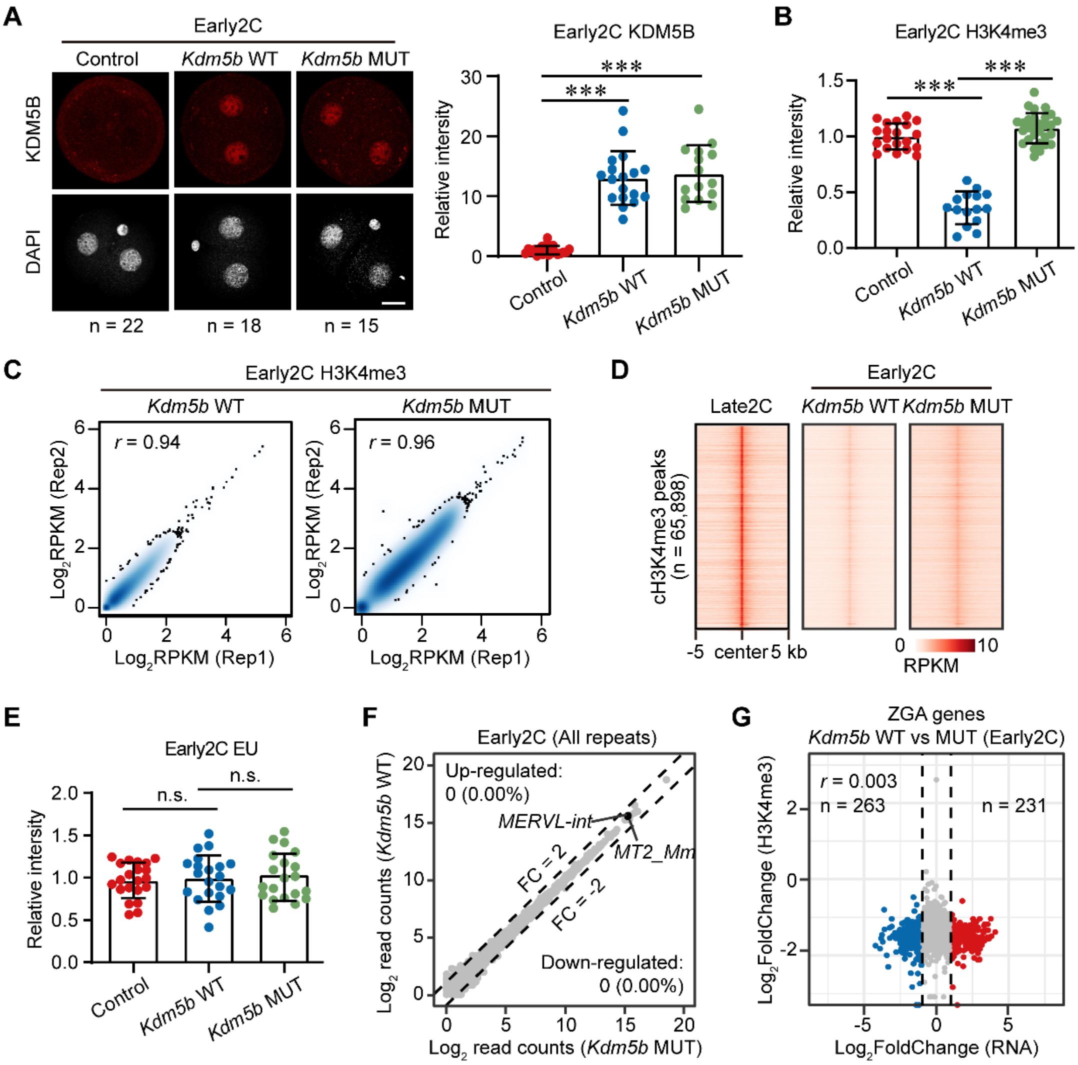

Appendix Figure S8. Overexpression of *Kdm5b* does not inhibit ZGA.

(A) Immunostaining for KDM5B (red) and DNA (gray) in Control, *Kdm5b* WT, and *Kdm5b* MUT embryos at the Early2C stage is shown on the left. The right panel quantifies the relative intensities of KDM5B. Scale bar, 20 µm. Error bars represent mean ± SD. ***$P < 0.001$; two-sided unpaired Student's *t*-test (Control vs *Kdm5b* WT P = 6.1E-15, Control vs *Kdm5b* MUT P = 1.6E-14). **(B)** Immunostaining quantification of H3K4me3 relative intensities was conducted in Control, *Kdm5b* WT, and *Kdm5b* MUT embryos at the Early2C stage. The number of embryos examined (n) is specified in Figure 3E. Error bars represent mean ± SD. ***$P < 0.001$; two-sided unpaired Student's *t*-test (Control vs *Kdm5b* WT P = 8.0E-16, *Kdm5b* WT vs *Kdm5b* MUT P = 1.1E-19). **(C)** Scatter plots illustrate correlations between two biological replicates of H3K4me3 CUT&Tag data in *Kdm5b* WT and *Kdm5b* MUT embryos at the Early2C stage. H3K4me3 enrichment was calculated

as RPKM using 5-kb bins (n = 546,206). Pearson correlation coefficients are shown on the top-left panel. **(D)** Heatmap showing H3K4me3 enrichment levels within cH3K4me3 peaks (n = 65,898) in Early2C embryos (both *Kdm5b* WT and *Kdm5b* MUT) and Late2C embryos. The characterization of these cH3K4me3 peaks were based on the ChIP-seq dataset GSE73952(Liu *et al*, 2016) derived from Late2C embryos. **(E)** Immunostaining quantification of EU-staining relative intensities was conducted in Control, *Kdm5b* WT, and *Kdm5b* MUT embryos at the Early2C stage. The number of embryos examined (n) is specified in Figure 3G. Error bars represent mean ± SD. n.s., no significance; two-sided unpaired Student's *t*-test (Control vs *Kdm5b* WT $P = 0.60$, *Kdm5b* WT vs *Kdm5b* MUT $P = 0.81$). **(F)** Scatter plots comparing the repeats expression levels between *Kdm5b* WT and *Kdm5b* MUT embryos at the Early2C stage, highlighting repeats up-regulated in *Kdm5b* WT embryos (red) and down-regulated in *Kdm5b* MUT embryos (blue). **(G)** Dot plot showcases the correlation between transcriptional changes in ZGA genes associated with H3K4me3 at promoters (n = 2,773) and changes in H3K4me3 ChIP-seq signal in *Kdm5b* WT versus *Kdm5b* MUT embryos at the Early2C stage.

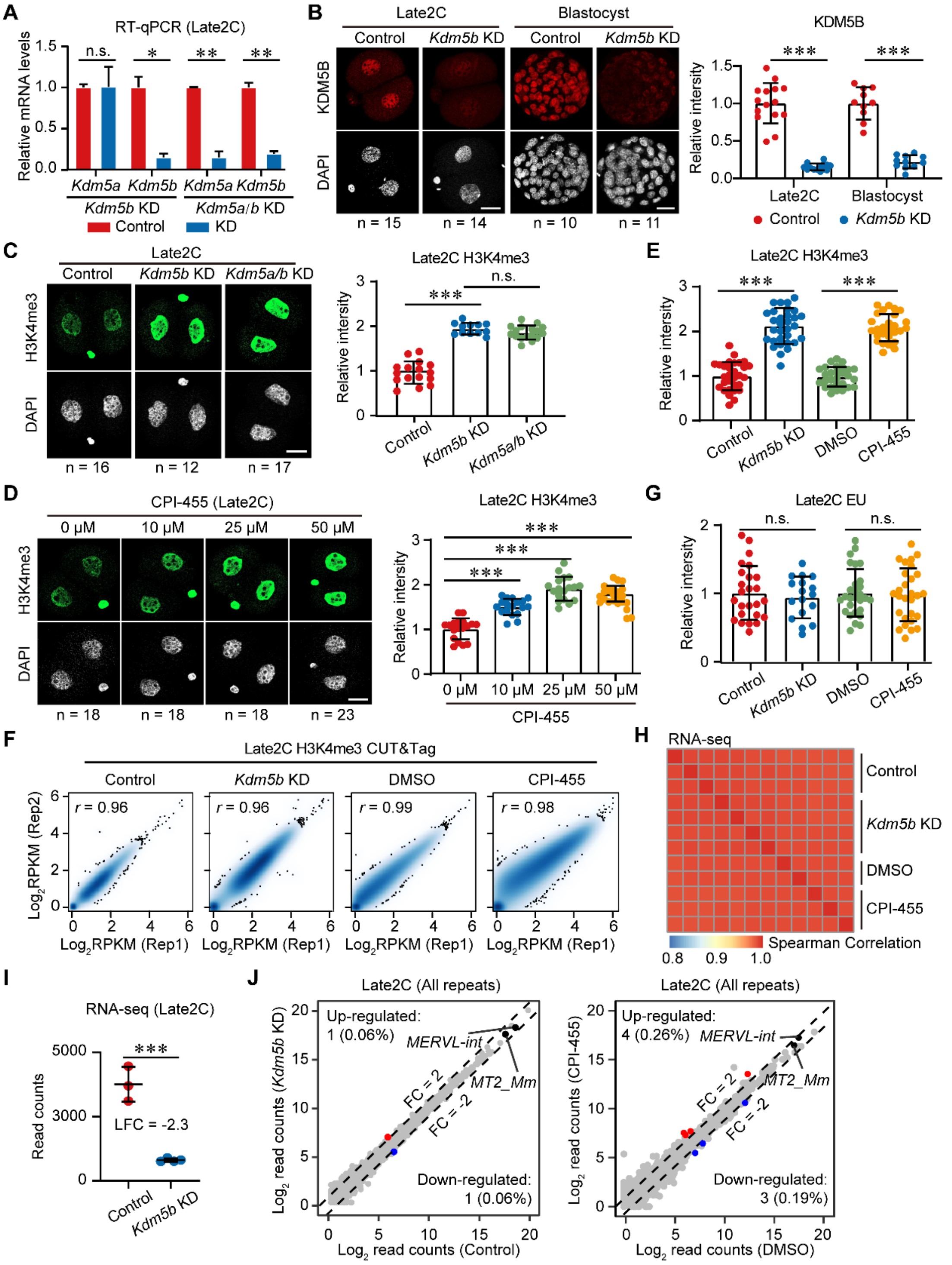

Appendix Figure S9. KDM5B inhibition from zygote to Late2C does not block ZGA.

(A) Bar chart showing the relative expression levels of *Kdm5a* and *Kdm5b* after knocking down of *Kdm5b* or *Kdm5a/b* at the Late2C stage, as detected by RT-qPCR. Error bars represent mean ± SD from two biological replicates. n.s., no significance; *$P < 0.05$; **$P < 0.01$; two-sided unpaired Student's t-test (*Kdm5b* KD: *Kdm5a* $P = 1$, *Kdm5b* $P = 0.02$; *Kdm5a/b* KD: *Kdm5a* $P = 0.002$,

Kdm5b $P = 0.004$). **(B)** Immunostaining for KDM5B (red) and DNA (gray) in Control and *Kdm5b* KD embryos at the Late2C and blastocyst stage is shown on the left. The right panel quantifies the relative intensities of KDM5B. Scale bar, 20 μm. Error bars represent mean ± SD. ***$P < 0.001$; two-sided unpaired Student's *t*-test (Late2C: $P = 5.4E\text{-}12$; Blastocyst: $P = 1.0E\text{-}09$). **(C)** Immunostaining for H3K4me3 (green) and DNA (gray) in Control, *Kdm5b* KD, and *Kdm5a*/*b* KD embryos at the Late2C stage is shown on the left. The right panel quantifies the relative intensities of H3K4me3. Scale bar, 20 μm. Error bars represent mean ± SD. n.s., no significance; ***$P < 0.001$; two-sided unpaired Student's *t*-test (Control vs *Kdm5b* KD $P = 9.6E\text{-}13$, *Kdm5b* KD vs *Kdm5a/b* KD $P = 0.13$). **(D)** The left segment presents immunostaining for H3K4me3 (green) and DNA (gray) in Late2C stage embryos treated with various CPI-455 concentrations (0, 10, 25, and 50 μM). The right bar plot visualizes the statistical data. Scale bar, 20 μm. Error bars represent mean ± SD. ***$P < 0.001$; two-sided unpaired Student's *t*-test (0 μM vs 10 μM $P = 3.5E\text{-}08$, 0 μM vs 25 μM $P = 1.6E\text{-}12$, 0 μM vs 50 μM $P = 4.3E\text{-}15$). **(E)** Immunostaining quantification of H3K4me3 relative intensities in Control, *Kdm5b* KD, DMSO-treated and CPI-455-treated embryos at the Late2C stage. The number of embryos examined (n) is specified in Figure 4B. Error bars represent mean ± SD. ***$P < 0.001$; two-sided unpaired Student's *t*-test (Control vs *Kdm5b* KD $P = 8.7E\text{-}18$, DMSO vs CPI-455 $P = 3.6E\text{-}21$). **(F)** Scatter plots display the correlation between two biological replicates of H3K4me3 CUT&Tag data for Control, *Kdm5b* KD, DMSO-treated and CPI-455-treated embryos at the Late2C stage. H3K4me3 enrichment was calculated as RPKM using 5-kb bins (n = 546,206). Pearson correlation coefficients are shown on the top-left panel. **(G)** Quantification of EU-staining intensities in Control, *Kdm5b* KD, DMSO-treated, and CPI-455-treated embryos at the Late2C stages, with the count of analyzed embryos (n) indicated in Figure 4E. Error bars represent mean ± SD. n.s., no significance; two-sided unpaired Student's *t*-test (Control vs *Kdm5b* KD $P = 0.56$, DMSO vs CPI-455 $P = 0.63$). **(H)** The heatmap visualizes the spearman correlation coefficients for RNA expression profiles across Control, *Kdm5b* KD, DMSO-treated, and CPI-455-treated embryos at the Late2C stage. **(I)** Expression levels of *Kdm5b* at the Late2C stage following *Kdm5b* KD are assessed via RNA-seq. Adjusted *P*-values are computed using DESeq2. Error bars denote mean ± SD from two biological replicates. ***$P < 0.001$ ($P = 0.0003$). LFC, Log_2 (fold change). **(J)** Scatter plots comparing the repeats expression levels between Control and *Kdm5b* KD embryos at the

Late2C stage (left), highlighting repeats up-regulated in *Kdm5b* KD embryos (red) and down-regulated in Control embryos (blue). Similarly, comparison plots for DMSO-treated versus CPI-455-treated embryos at the Late2C stage (right) identify repeats up-regulated in CPI-455-treated (red) and down-regulated in DMSO-treated embryos (blue).

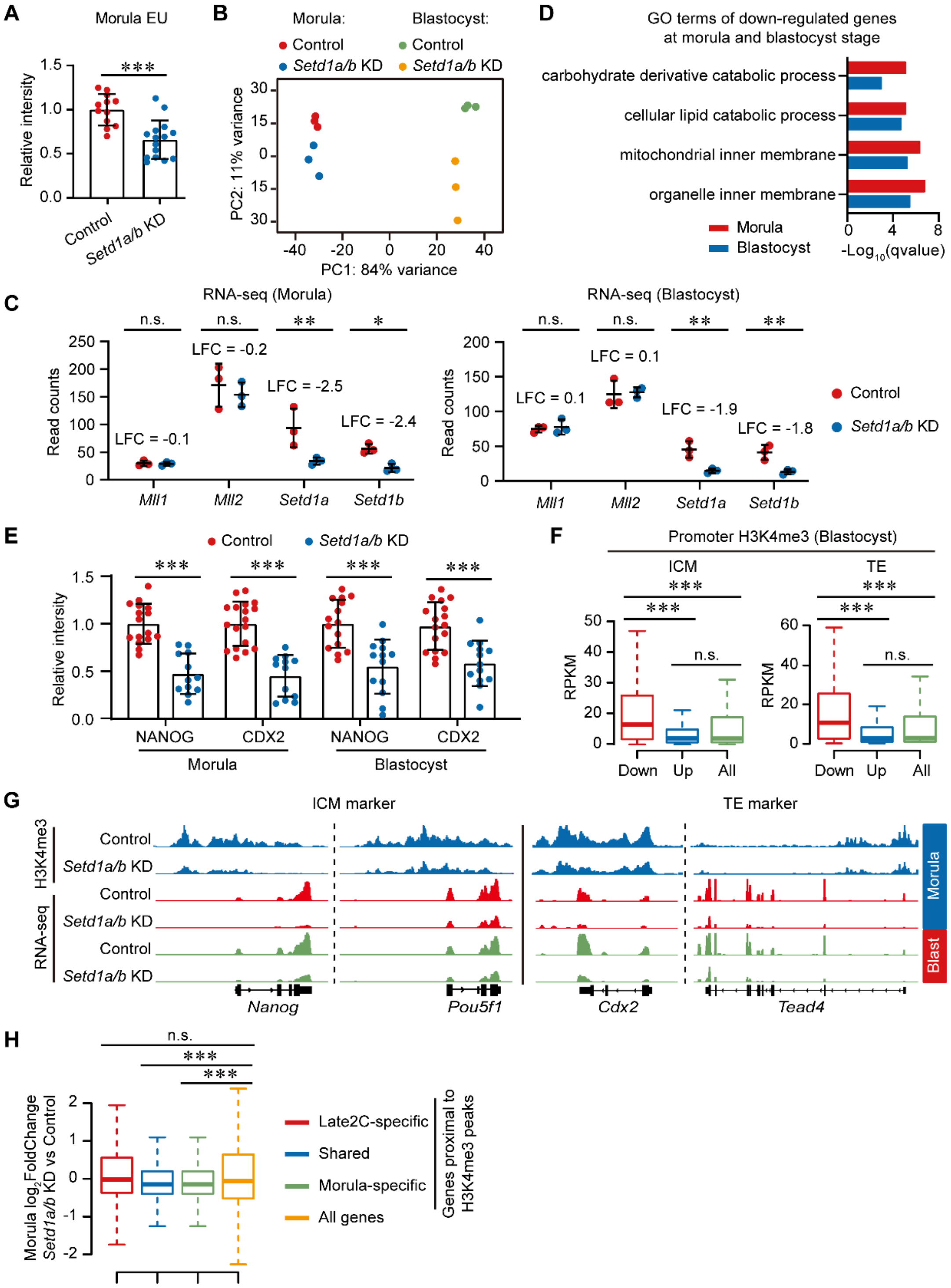

Appendix Figure S10. Effect of *Setd1a/b* KD on cell fate commitment.

(A) Quantification of EU-staining intensities in Control, *Setd1a/b* KD embryos at the morula stage, referencing the number of embryos examined (n) from Figure 5C. Error bars represent mean ± SD. ***$P < 0.001$; two-sided unpaired Student's t-test ($P = 0.0002$). **(B)** Principal component analysis of gene expression in Control and *Setd1a/b* KD embryos at the morula and blastocyst stages. **(C)**

Expression levels of *Mll1*, *Mll2*, *Setd1a*, and *Setd1b* at the morula and blastocyst stages following *Setd1a/b* KD are assessed via RNA-seq. Adjusted *P*-values are computed using DESeq2. Error bars denote mean ± SD from two biological replicates. n.s., no significance; $*P < 0.05$; $**P < 0.01$ (Morula: *Mll1* $P = 0.25$, *Mll2* $P = 0.062$, *Setd1a* $P = 0.0029$, *Setd1b* $P = 0.014$; Blastocyst: *Mll1* $P = 0.35$, *Mll2* $P = 0.076$, *Setd1a* $P = 0.0023$, *Setd1b* $P = 0.0035$). LFC, Log_2 (fold change). **(D)** Bar charts showing the enriched Gene Ontology (GO) terms for genes down-regulated in *Setd1a/b* KD embryos at the morula and blastocyst stages. **(E)** Quantification of relative intensities for NANOG and CDX2 in Control and *Setd1a/b* KD embryos at morula and blastocyst stage, with embryo counts as noted in Figure 5F. Error bars represent mean ± SD. $***P < 0.001$; two-sided unpaired Student's *t*-test (Morula: NANOG $P = 7.0E\text{-}07$, CDX2 $P = 4.8E\text{-}07$; Blastocyst: NANOG $P = 0.0002$, CDX2 $P = 0.0002$). **(F)** Box plot showing H3K4me3 enrichment levels at the promoter regions (TSS ± 2 kb) for down-regulated (n = 4,654), up-regulated (n = 250), and all genes (n = 22,470) at the blastocyst stage. Boxplot was used to display data distribution, with the median as the central line, the box showing the IQR from the 25th to 75th percentile, and whiskers extending to data points within 1.5 times the IQR. n.s., not significant; $***P < 0.001$; two-sided Wilcoxon-Mann-Whitney test (ICM: down-regulated genes vs all genes $P < 2.2E\text{-}16$, up-regulated genes vs all genes $P = 0.064$, down-regulated genes vs up-regulated genes $P < 2.2E\text{-}16$; TE: down-regulated genes vs all genes $P < 2.2E\text{-}16$, up-regulated genes vs all genes $P = 0.47$, down-regulated genes vs up-regulated genes $P < 2.2E\text{-}16$). The H3K4me3 ChIP-seq data for blastocysts (ICM and TE) is sourced from GSE73952(Liu *et al.*, 2016). **(G)** Genome browser views showing the H3K4me3 enrichments and RNA expression for representative ICM and TE marker genes in Control and *Setd1a/b* KD embryos. **(H)** Box plots showing expression changes of genes near to Late2C-specific (n = 1,586), shared (n = 2,856) and morula-specific (n = 5,175) H3K4me3 peaks in Control and *Setd1a/b* KD embryos at the morula stage. Boxplot was used to display data distribution, with the median as the central line, the box showing the IQR from the 25th to 75th percentile, and whiskers extending to data points within 1.5 times the IQR. n.s., not significant; $***P < 0.001$; two-sided Wilcoxon-Mann-Whitney test (Late2C-specific genes vs all genes $P = 0.09$, Shared genes vs all genes $P = 6.47E\text{-}15$, Morula-specific genes vs all genes $P < 2.2E\text{-}16$).

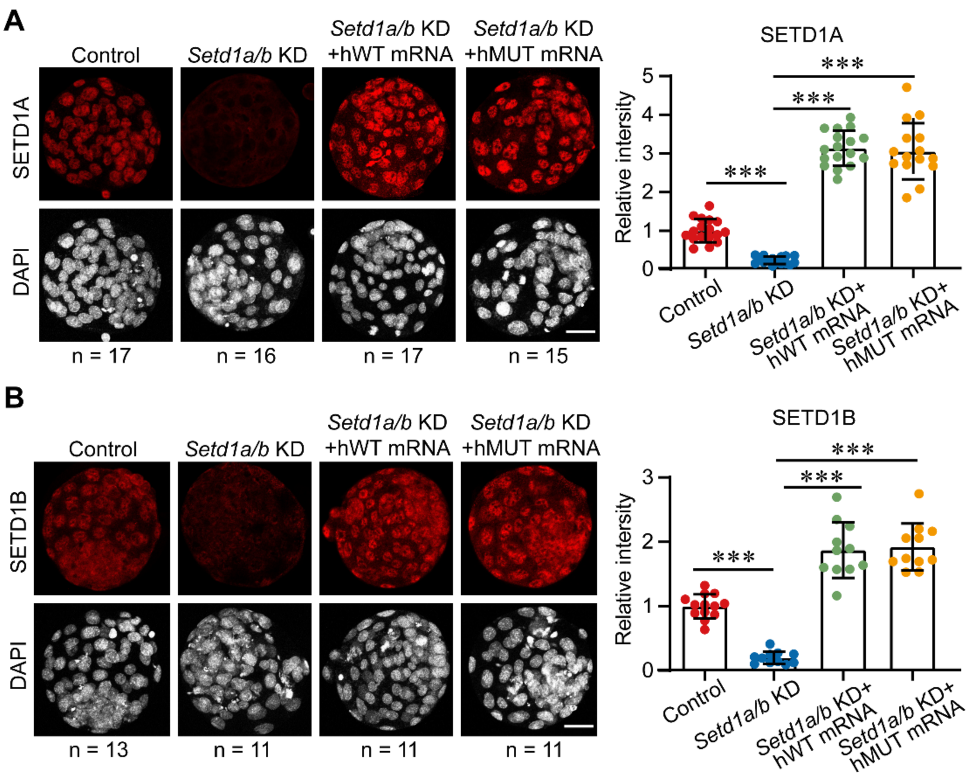

Appendix Figure S11. Immunostaining of SETD1A and SETD1B proteins at the blastocyst stage.

(A) Immunostaining for SETD1A (red) and DNA (gray) in Control, *Setd1a/b* KD, *Setd1a/b* KD + hWT, and *Setd1a/b* KD + hMUT mRNA embryos at the blastocyst stage is shown on the left. The right panel quantifies the relative intensities of blastocyst. Error bars represent mean ± SD. ***$P < 0.001$ two-sided Student's *t*-test (Control vs *Setd1a/b* KD P = 5.4E-11, *Setd1a/b* KD vs *Setd1a/b* KD + hWT mRNA P = 3.6E-22, *Setd1a/b* KD vs *Setd1a/b* KD + hMUT mRNA P = 1.8E-15). **(B)** Immunostaining for SETD1B (red) and DNA (gray) in Control, *Setd1a/b* KD, *Setd1a/b* KD + hWT, and *Setd1a/b* KD + hMUT mRNA embryos at the blastocyst stage is shown on the left. The right panel quantifies the relative intensities of blastocyst. Error bars represent mean ± SD. ***$P < 0.001$ two-sided Student's *t*-test (Control vs *Setd1a/b* KD P = 9.8E-12, *Setd1a/b* KD vs *Setd1a/b* KD + hWT mRNA P = 6.0E-11, *Setd1a/b* KD vs *Setd1a/b* KD + hMUT mRNA P = 2.0E-12).

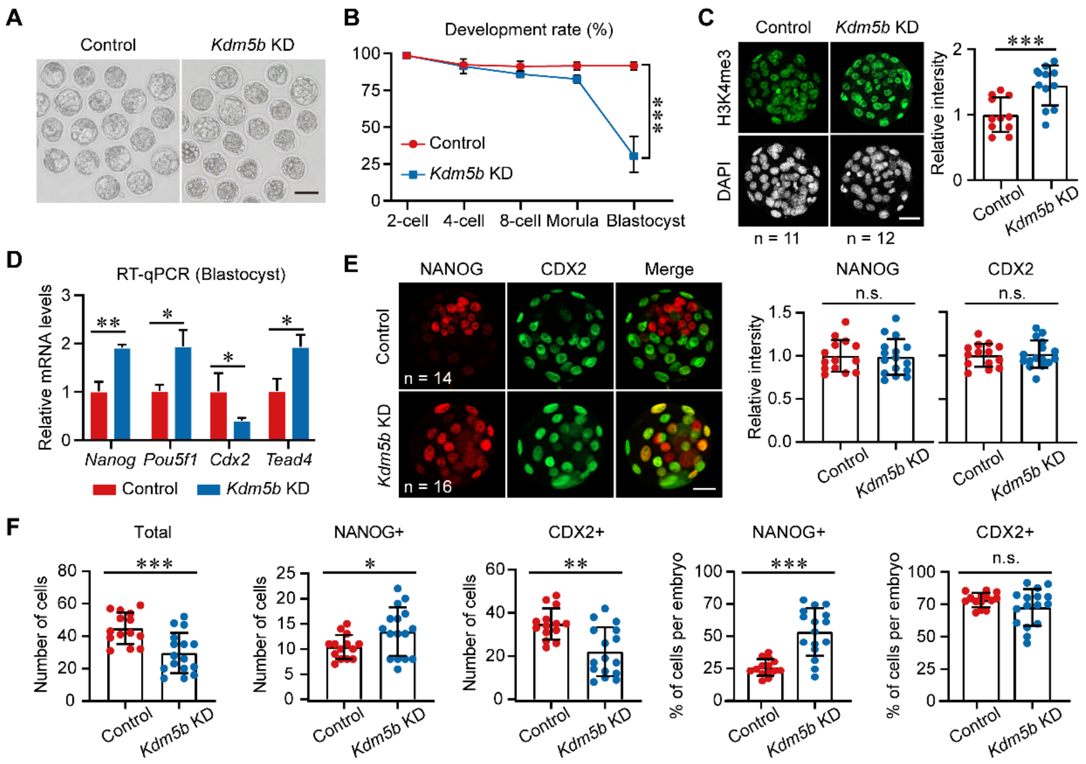

Appendix Figure S12. Effect of *Kdm5b* KD on cell fate commitment.

(A) Representative blastocyst images from Control and *Kdm5b* KD group, with a typical example from three independent experiments shown. Scale bar, 50 µm. **(B)** Line plot showing the development rate of embryos from Control and *Kdm5b* KD embryos at specified time points. Error bars represent mean ± SD from three biological replicates. ****P* < 0.001; two-sided unpaired Student's *t*-test (*P* = 0.0009). **(C)** Left: Immunofluorescence images of H3K4me3 (green) and DNA (gray) in Control and *Kdm5b* KD blastocysts. Right: Quantification of relative H3K4me3 intensities. Scale bar, 20 µm. Error bars represent mean ± SD. ****P* < 0.001; two-sided Student's *t*-test (*P* = 0.0003). **(D)** Bar charts detail relative expression of ICM markers (*Nanog*, *Pou5f1*) and TE markers (*Cdx2*, *Tead4*) in Control and *Kdm5b* KD blastocysts. Error bars represent mean ± SD from three biological replicates. **P* < 0.05; ***P* < 0.01; two-sided unpaired Student's *t*-test (*Nanog P* = 0.002, *Pou5f1 P* = 0.01, *Cdx2 P* = 0.05, *Tead4 P* = 0.01). **(E)** Immunostaining of NANOG (red), CDX2 (green), and DNA (gray) in Control and *Kdm5b* KD embryos at the blastocyst stage (left), with quantification of NANOG and CDX2 intensities on the right. Scale bar, 20 µm. Error bars represent mean ± SD. n.s., no significance; two-sided unpaired Student's *t*-test (NANOG *P* = 0.87, CDX2 *P* = 0.75). **(F)** Bar plots illustrate the variance in total cell counts, NANOG+ (positive), and CDX2+

(positive) cell numbers between Control and *Kdm5b* KD blastocysts, alongside ratios of NANOG+ to CDX2+ cells. The embryo count (n) referred from Appendix Figure S12E. Error bars represent mean ± SD. n.s., no significance; **P* < 0.05; ***P* < 0.01; ****P* < 0.001; two-sided unpaired Student's *t*-test (Number of cells: Total *P* = 0.0009, NANOG *P* = 0.04, CDX2 *P* = 0.001; % of cells per embryo: NANOG *P* = 1.3E-05, CDX2 *P* = 0.39).

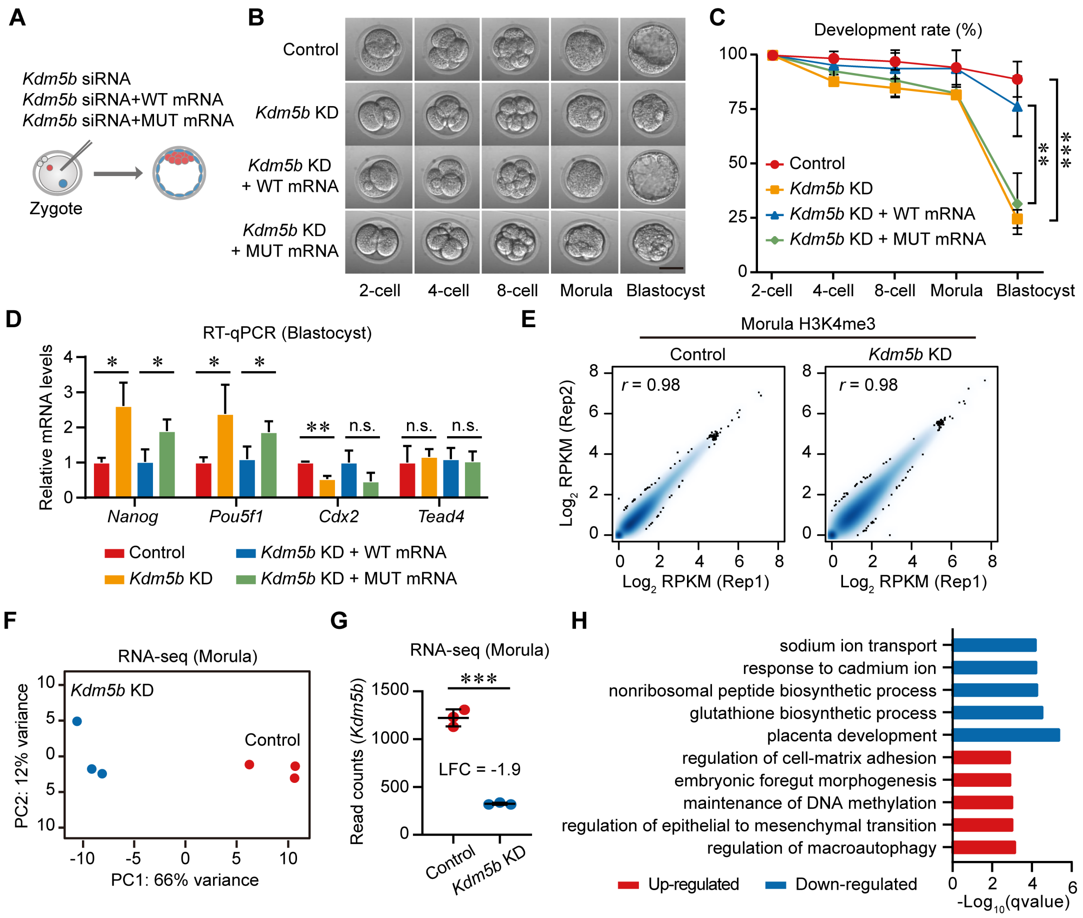

Appendix Figure S13. The catalytic role of KDM5B in the first lineage segregation.

(A) A schematic outline the methodology for microinjection into zygotes, detailing the introduction of *Kdm5b* siRNA to achieve knockdown (*Kdm5b* KD), the co-injection of *Kdm5b* siRNA with WT *Kdm5b* mRNA (*Kdm5b* KD + WT mRNA), and the combination of *Kdm5b* siRNA with MUT *Kdm5b* mRNA (*Kdm5b* KD + MUT mRNA). **(B)** Images of early embryos from Control, *Kdm5b* KD, *Kdm5b* KD + *Kdm5b* WT mRNA, and *Kdm5b* KD + *Kdm5b* MUT mRNA treatment, displaying a typical example from three independent experiments. Scale bar, 50 µm. **(C)** Line plot showing the development rate of embryos from Control, *Kdm5b* KD, *Kdm5b* KD + *Kdm5b* WT mRNA, and *Kdm5b* KD + *Kdm5b* MUT mRNA groups. Error bars represent mean ± SD from three biological replicates. $**P < 0.01$; $***P < 0.001$; two-sided unpaired Student's *t*-test (Control vs *Kdm5b* KD $P = 7.0E\text{-}05$, *Kdm5b* KD + WT mRNA vs *Kdm5b* KD + MUT mRNA $P = 0.005$). **(D)** Bar chart showing the relative expression levels of *Nanog*, *Pou5f1*, *Cdx2* and *Tead4* in blastocysts from Control, *Kdm5b* KD, *Kdm5b* KD + *Kdm5b* WT mRNA, and *Kdm5b* KD + *Kdm5b* MUT mRNA groups. Error bars represent mean ± SD from three biological replicates. n.s., no significance; $*P <$

0.05; **P < 0.01; two-sided unpaired Student's t-test (*Nanog*: Control vs *Kdm5b* KD P = 0.014, *Kdm5b* KD + WT mRNA vs *Kdm5b* KD + MUT mRNA P = 0.035; *Pou5f1*: Control vs *Kdm5b* KD P = 0.046, *Kdm5b* KD + WT mRNA vs *Kdm5b* KD + MUT mRNA P = 0.046; *Cdx2*: Control vs *Kdm5b* KD P = 0.0012, *Kdm5b* KD + WT mRNA vs *Kdm5b* KD + MUT mRNA P = 0.089; *Tead4*: Control vs *Kdm5b* KD P = 0.61, *Kdm5b* KD + WT mRNA vs *Kdm5b* KD + MUT mRNA P = 0.82). **(E)** Scatter plots illustrate correlations between biological replicates of H3K4me3 CUT&Tag data in Control and *Kdm5b* KD morulae. H3K4me3 enrichment was calculated as RPKM using 5-kb bins (n = 546,206). Pearson correlation coefficients are shown on the top-left panel. **(F)** Principal component analysis of gene expression in Control and *Kdm5b* KD morulae. **(G)** Expression levels of *Kdm5b* at the morula stage following *Kdm5b* KD are assessed via RNA-seq. Adjusted P-values are computed using DESeq2. Error bars denote mean ± SD from three biological replicates. ***P < 0.001 (P = 6.6E-05). LFC, Log_2 (fold change). **(H)** Bar charts showing the enriched GO terms for genes up-regulated and down-regulated in *Kdm5b* KD morulae.

References

Liu X, Wang C, Liu W, Li J, Li C, Kou X, Chen J, Zhao Y, Gao H, Wang H *et al* (2016) Distinct features of H3K4me3 and H3K27me3 chromatin domains in pre-implantation embryos. *Nature* 537: 558-562

Wang C, Liu X, Gao Y, Yang L, Li C, Liu W, Chen C, Kou X, Zhao Y, Chen J *et al* (2018) Reprogramming of H3K9me3-dependent heterochromatin during mammalian embryo development. *Nat Cell Biol* 20: 620-631

Xiong Z, Xu K, Lin Z, Kong F, Wang Q, Quan Y, Sha QQ, Li F, Zou Z, Liu L *et al* (2022) Ultrasensitive Ribo-seq reveals translational landscapes during mammalian oocyte-to-embryo transition and pre-implantation development. *Nat Cell Biol* 24: 968-980

Zhang C, Wang M, Li Y, Zhang Y (2022) Profiling and functional characterization of maternal mRNA translation during mouse maternal-to-zygotic transition. *Sci Adv* 8: eabj3967
