# Supplementary figures and images for: Histone methyltransferases MLL2 and SETD1A/B play distinct roles in H3K4me3 deposition during the transition from totipotency to pluripotency (part 1 of 3)
Source: EMBO J. 2024 Dec 5;44(2):437–56. doi: 10.1038/s44318-024-00329-5 (PMC11730331; doi:10.1038/s44318-024-00329-5)

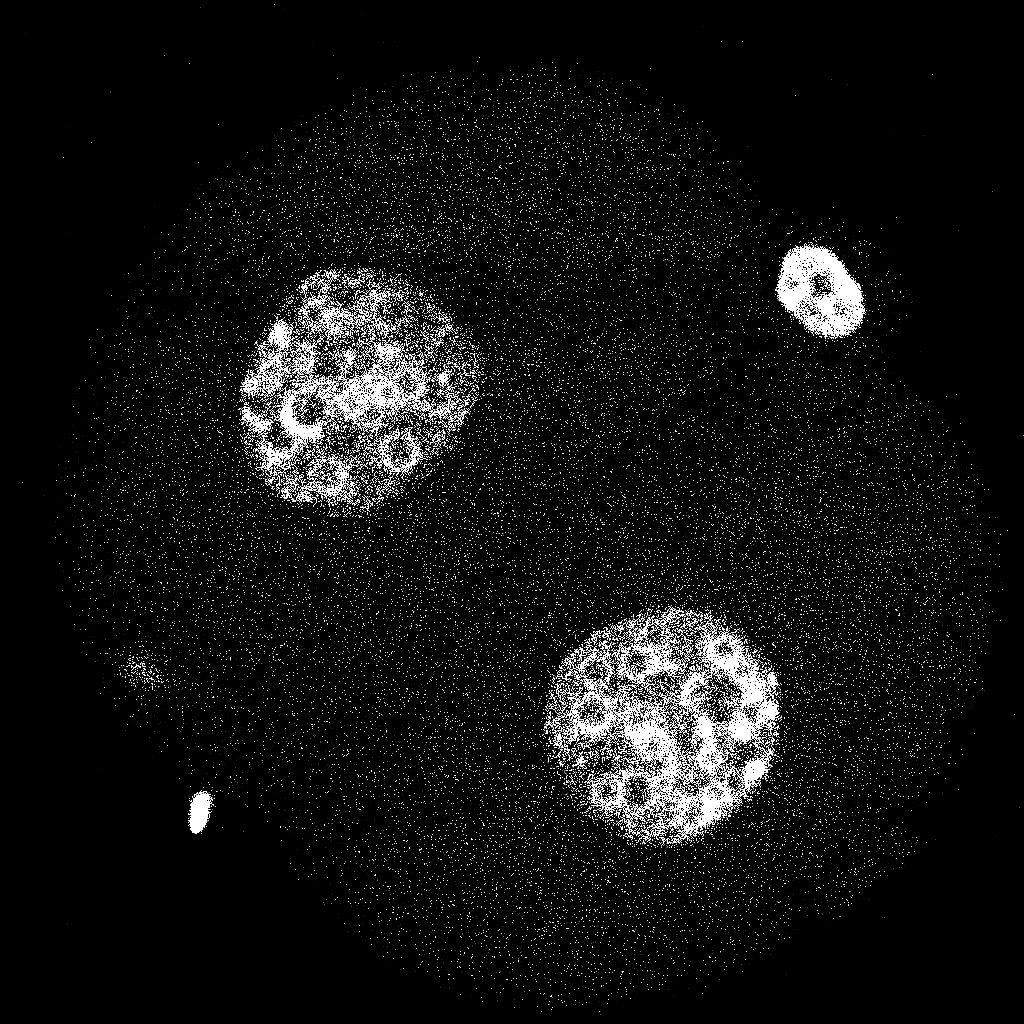

Supplement: Supplementary file 11 — Source data Fig. 1 [file 44318_2024_329_MOESM11_ESM.zip › SD figure 1/1B/Late2C_Control_DAPI.jpg]

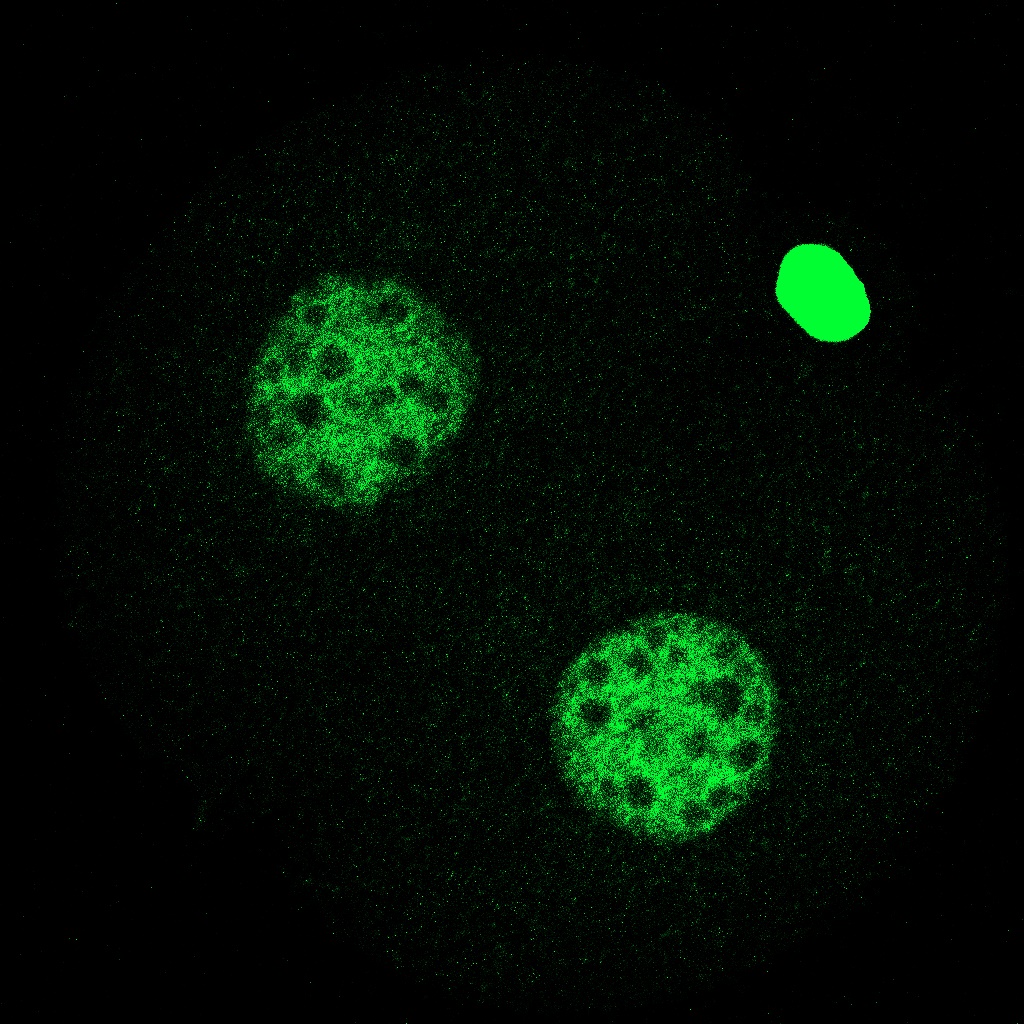

Supplement: Supplementary file 11 — Source data Fig. 1 [file 44318_2024_329_MOESM11_ESM.zip › SD figure 1/1B/Late2C_Control_H3K4me3.jpg]

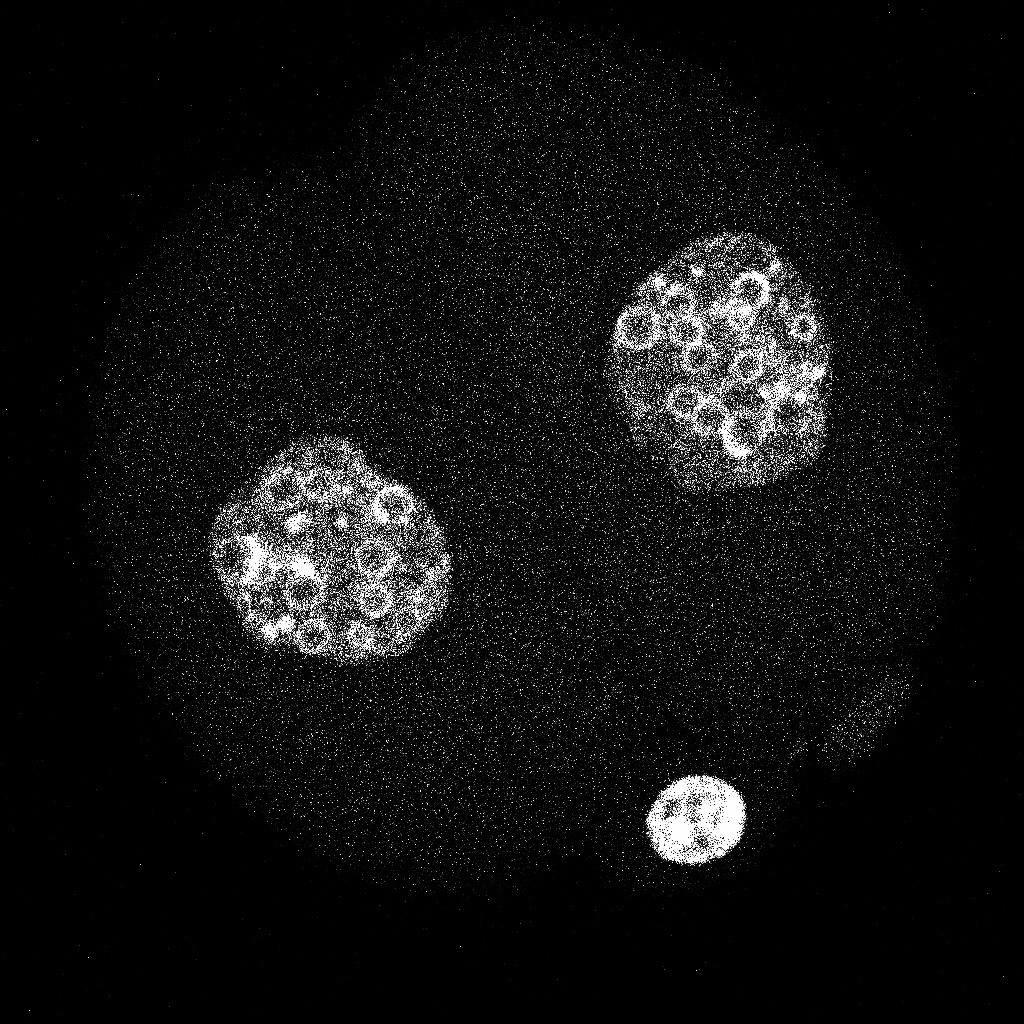

Supplement: Supplementary file 11 — Source data Fig. 1 [file 44318_2024_329_MOESM11_ESM.zip › SD figure 1/1B/Late2C_Mll2 KD_DAPI.jpg]

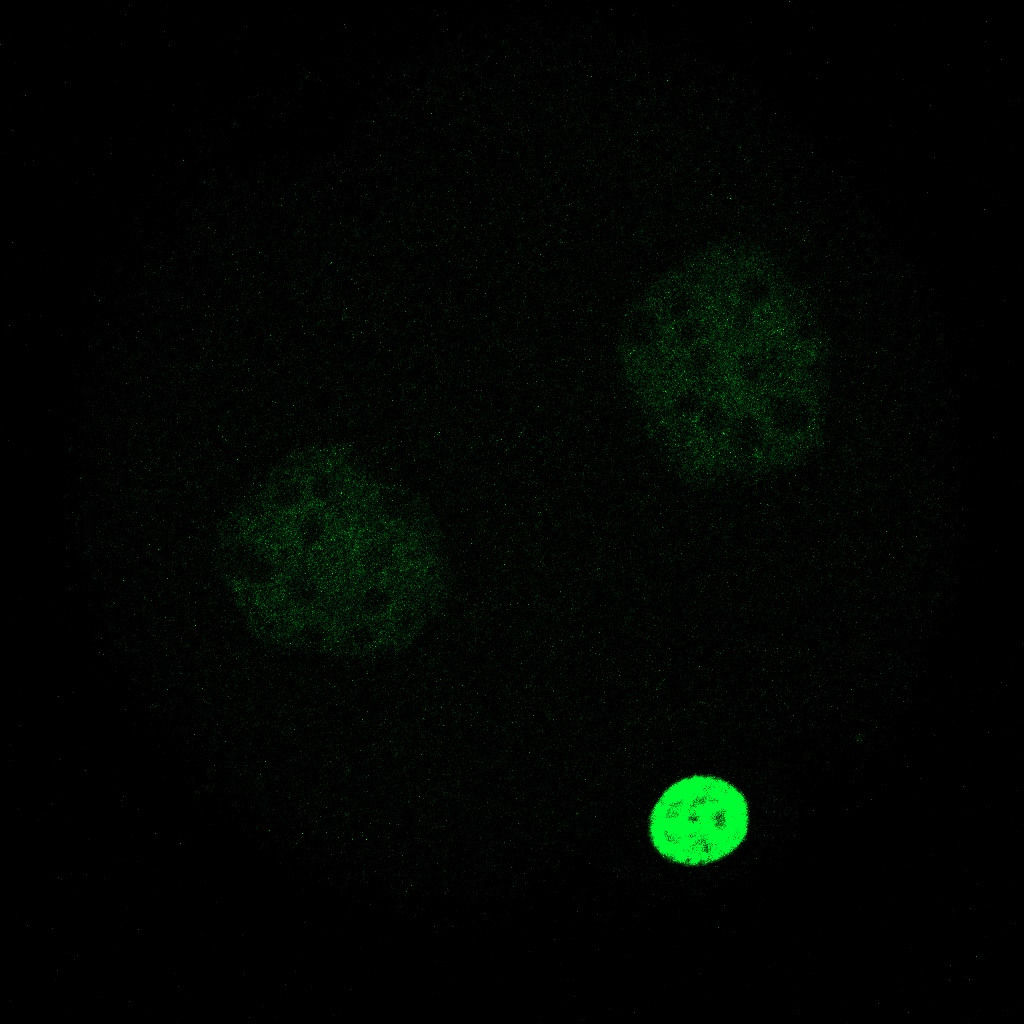

Supplement: Supplementary file 11 — Source data Fig. 1 [file 44318_2024_329_MOESM11_ESM.zip › SD figure 1/1B/Late2C_Mll2 KD_H3K4me3.jpg]

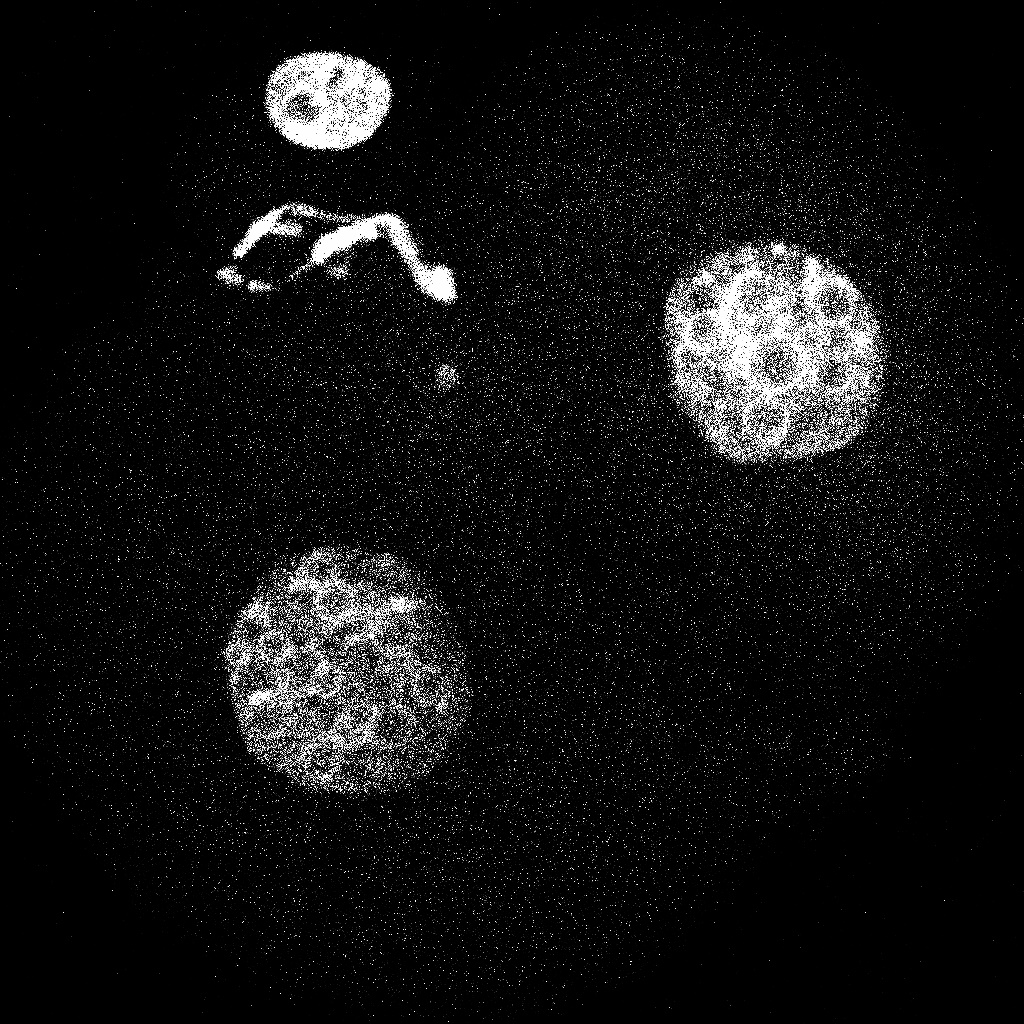

Supplement: Supplementary file 11 — Source data Fig. 1 [file 44318_2024_329_MOESM11_ESM.zip › SD figure 1/1B/Late2C_Setd1ab KD_DAPI.jpg]

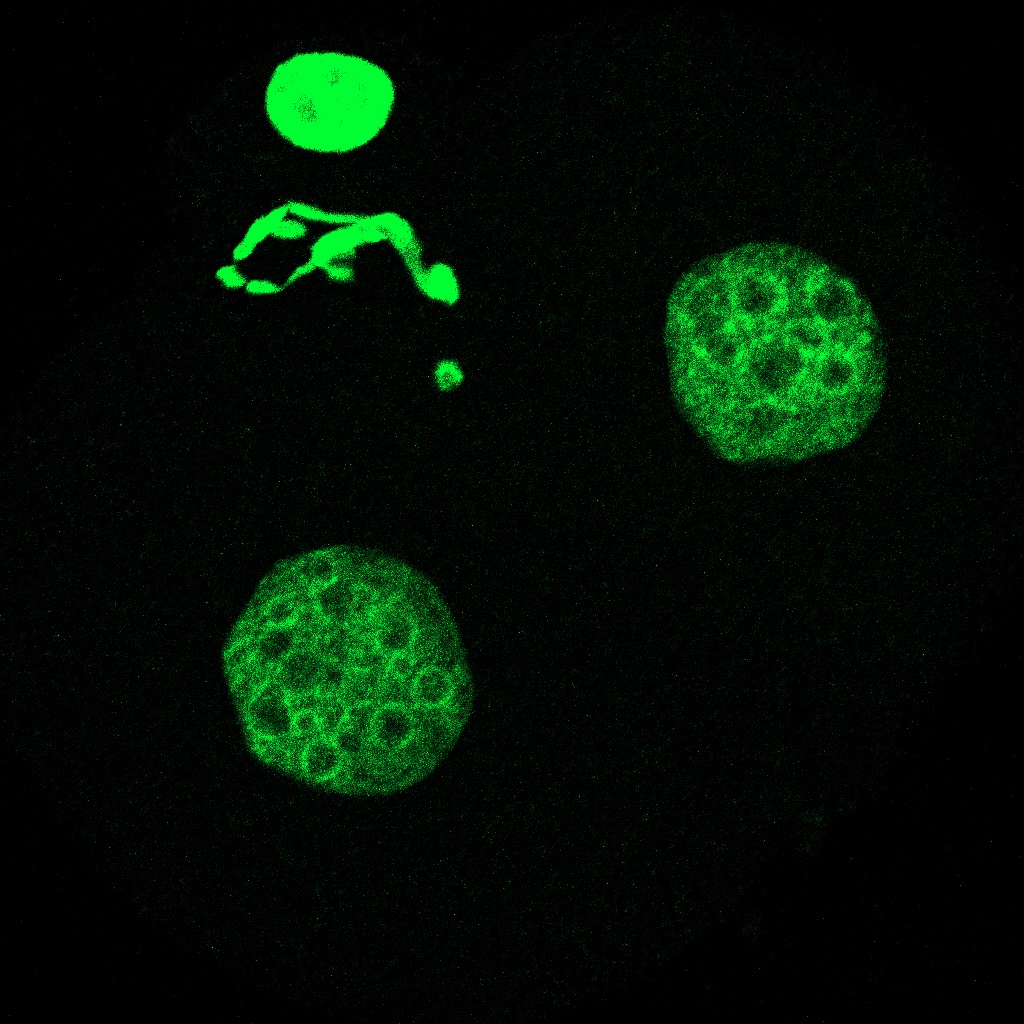

Supplement: Supplementary file 11 — Source data Fig. 1 [file 44318_2024_329_MOESM11_ESM.zip › SD figure 1/1B/Late2C_Setd1ab KD_H3K4me3.jpg]

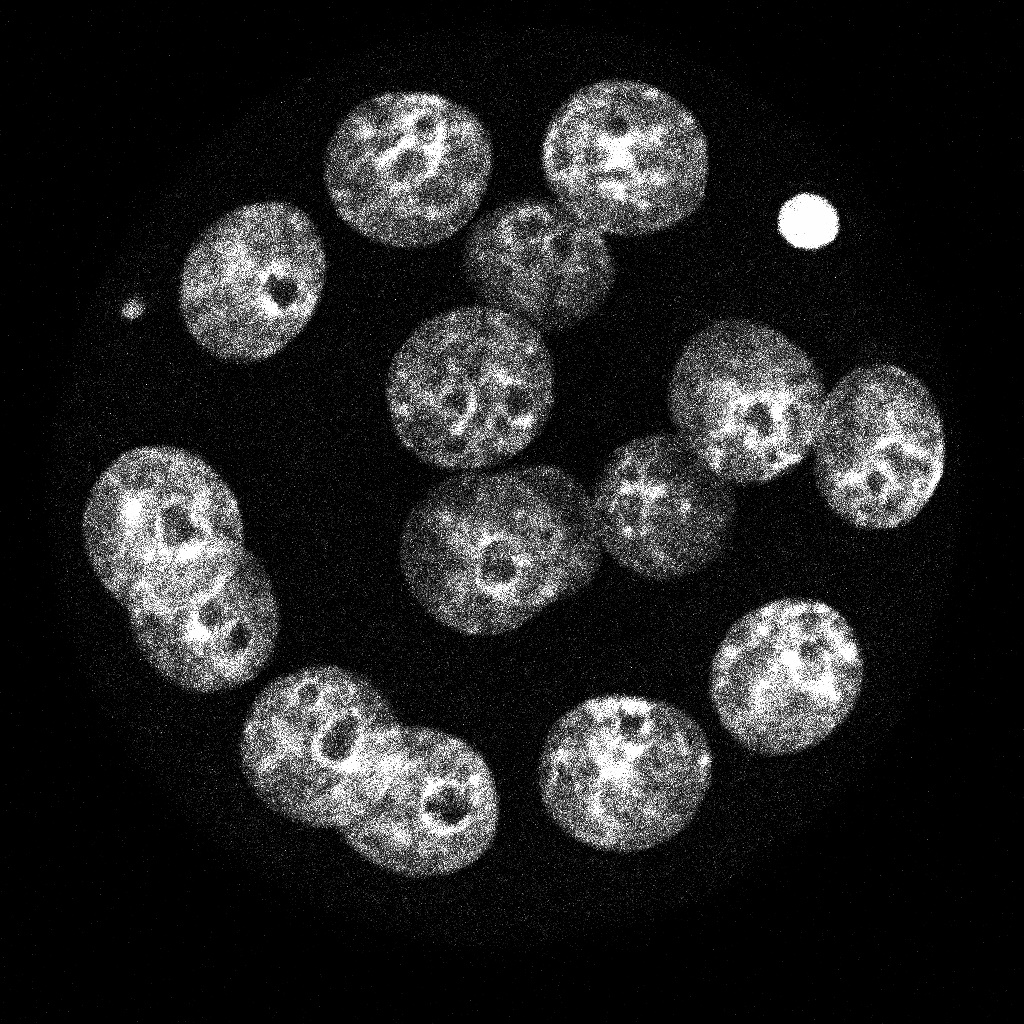

Supplement: Supplementary file 11 — Source data Fig. 1 [file 44318_2024_329_MOESM11_ESM.zip › SD figure 1/1B/Morula_Control_DAPI.jpg]

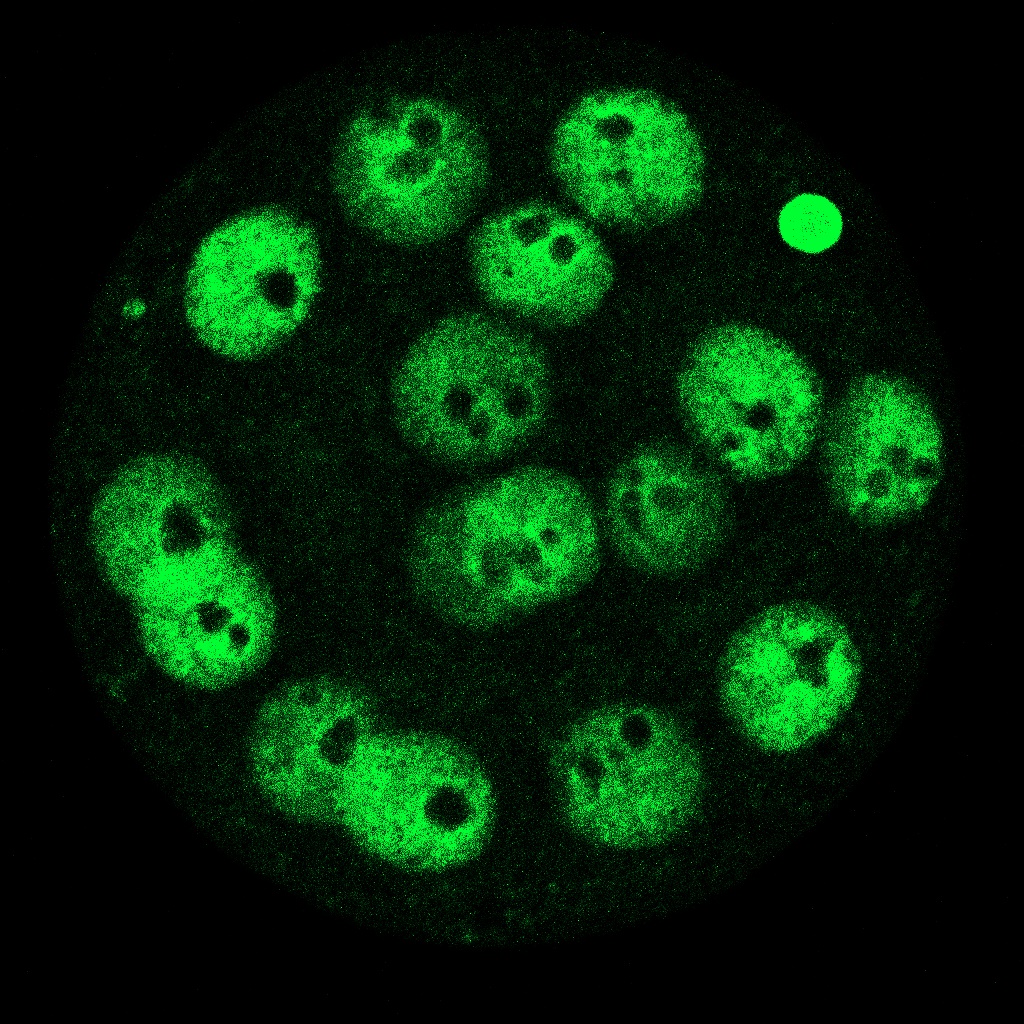

Supplement: Supplementary file 11 — Source data Fig. 1 [file 44318_2024_329_MOESM11_ESM.zip › SD figure 1/1B/Morula_Control_H3K4me3.jpg]

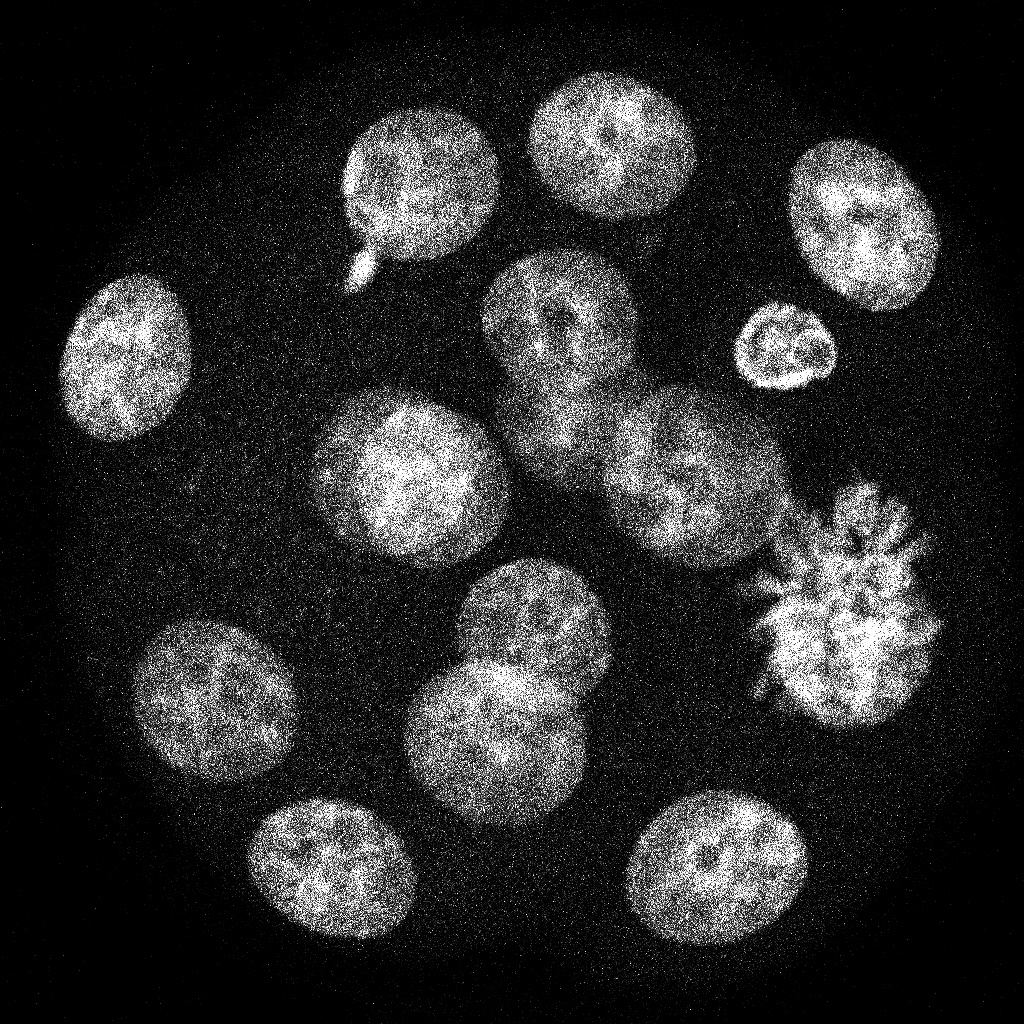

Supplement: Supplementary file 11 — Source data Fig. 1 [file 44318_2024_329_MOESM11_ESM.zip › SD figure 1/1B/Morula_Mll2 KD_DAPI.jpg]

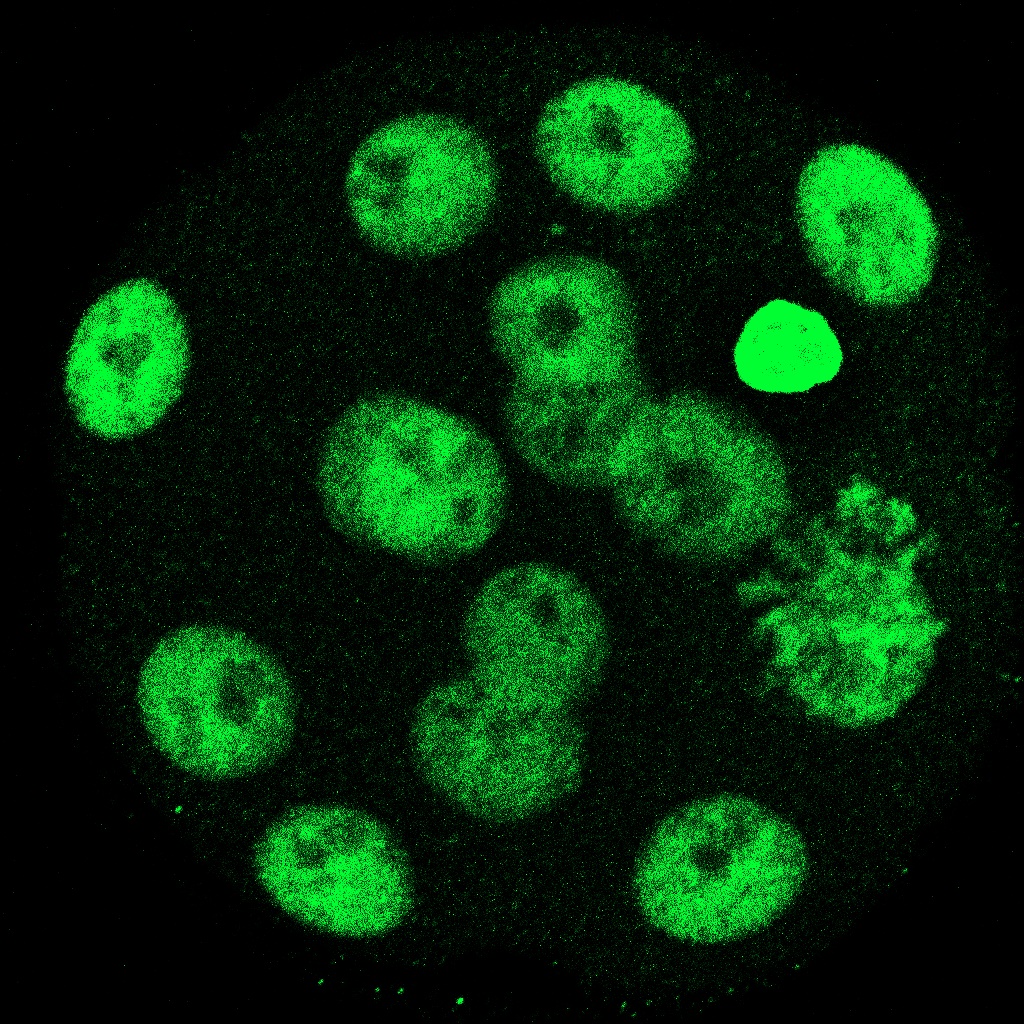

Supplement: Supplementary file 11 — Source data Fig. 1 [file 44318_2024_329_MOESM11_ESM.zip › SD figure 1/1B/Morula_Mll2 KD_H3K4me3.jpg]

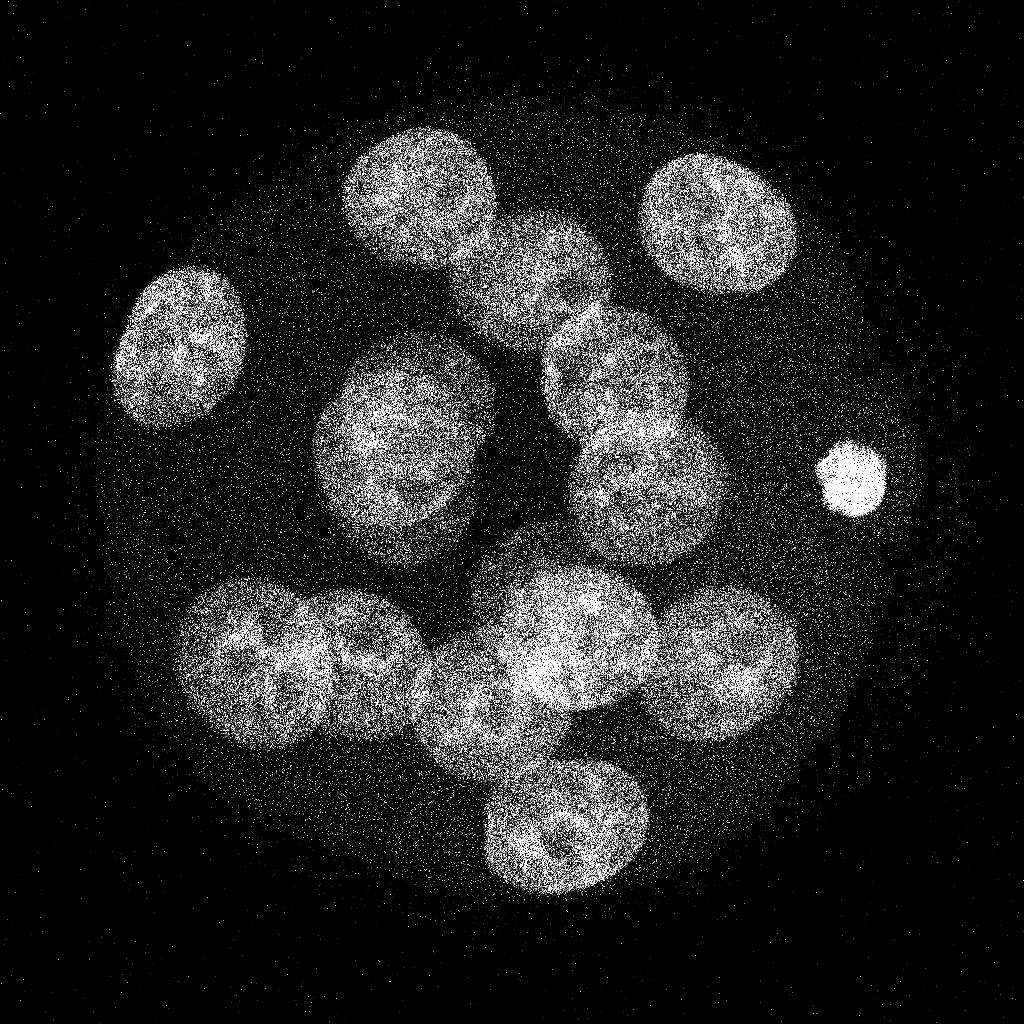

Supplement: Supplementary file 11 — Source data Fig. 1 [file 44318_2024_329_MOESM11_ESM.zip › SD figure 1/1B/Morula_Setd1ab KD_DAPI.jpg]

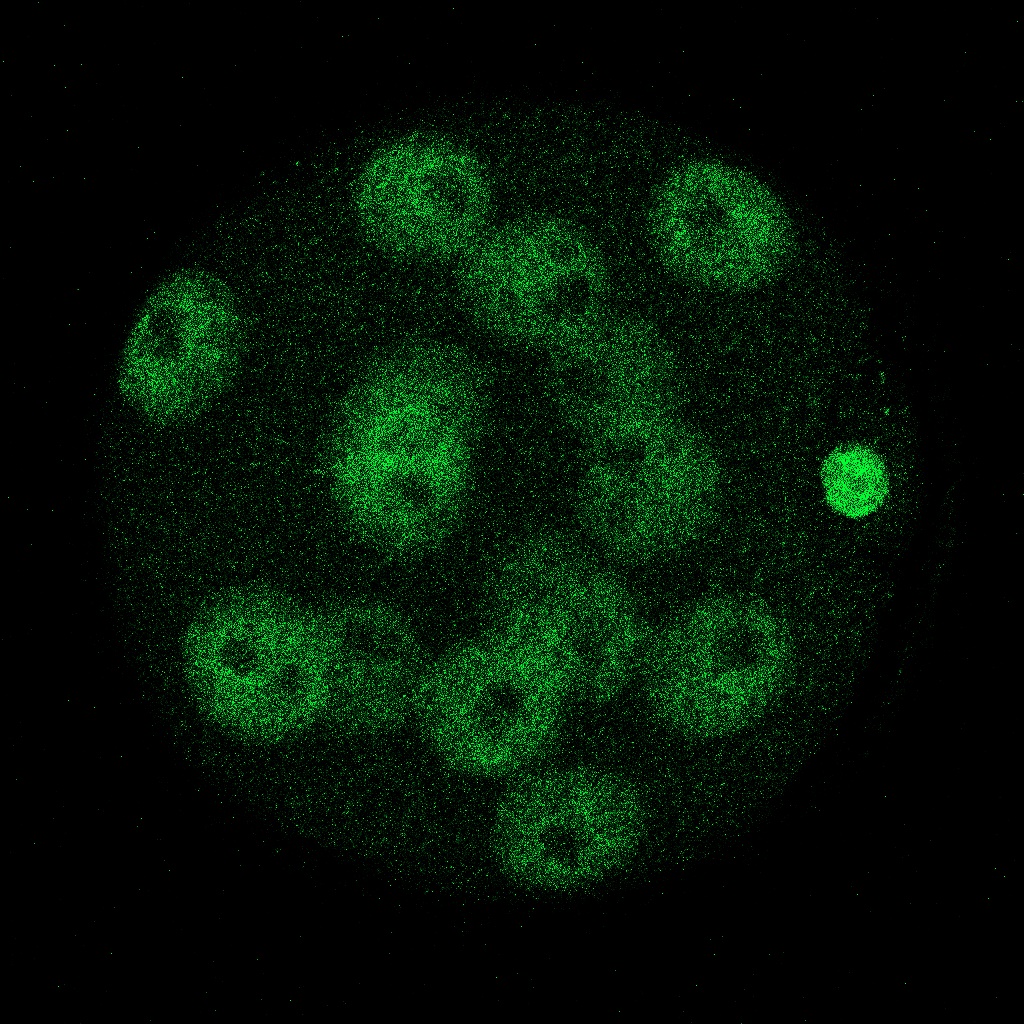

Supplement: Supplementary file 11 — Source data Fig. 1 [file 44318_2024_329_MOESM11_ESM.zip › SD figure 1/1B/Morula_Setd1ab KD_H3K4me3.jpg]

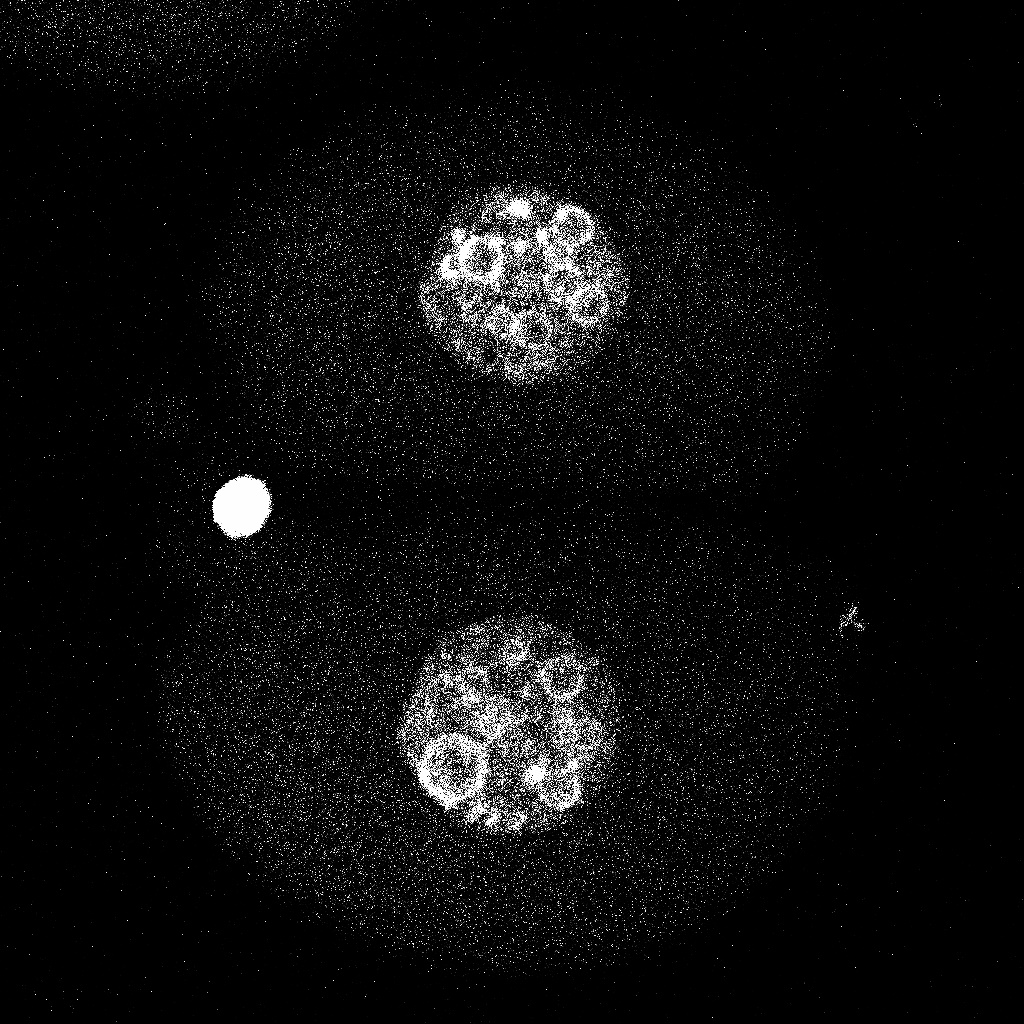

Supplement: Supplementary file 12 — Source data Fig. 2 [file 44318_2024_329_MOESM12_ESM.zip › SD figure 2/2C/Late2C_Trp_0h_DAPI.jpg]

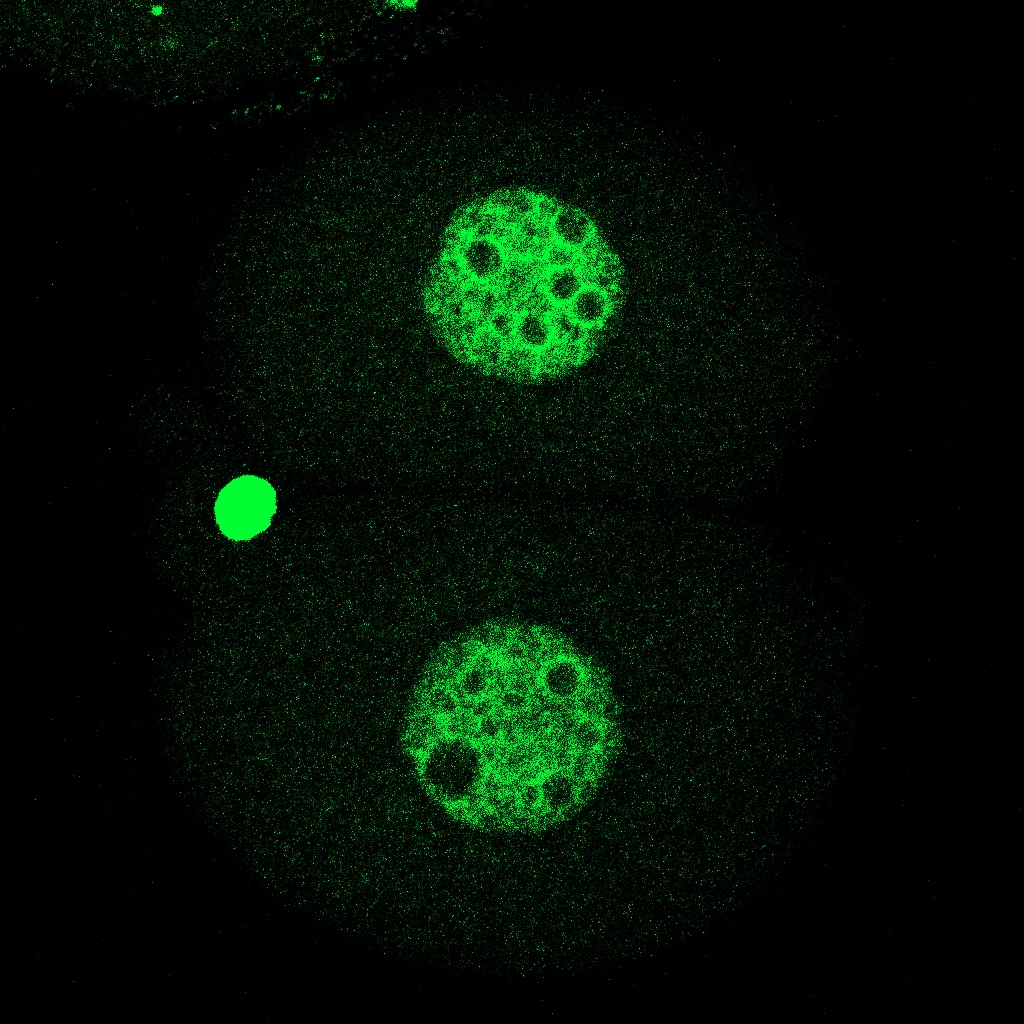

Supplement: Supplementary file 12 — Source data Fig. 2 [file 44318_2024_329_MOESM12_ESM.zip › SD figure 2/2C/Late2C_Trp_0h_H3K4me3.jpg]

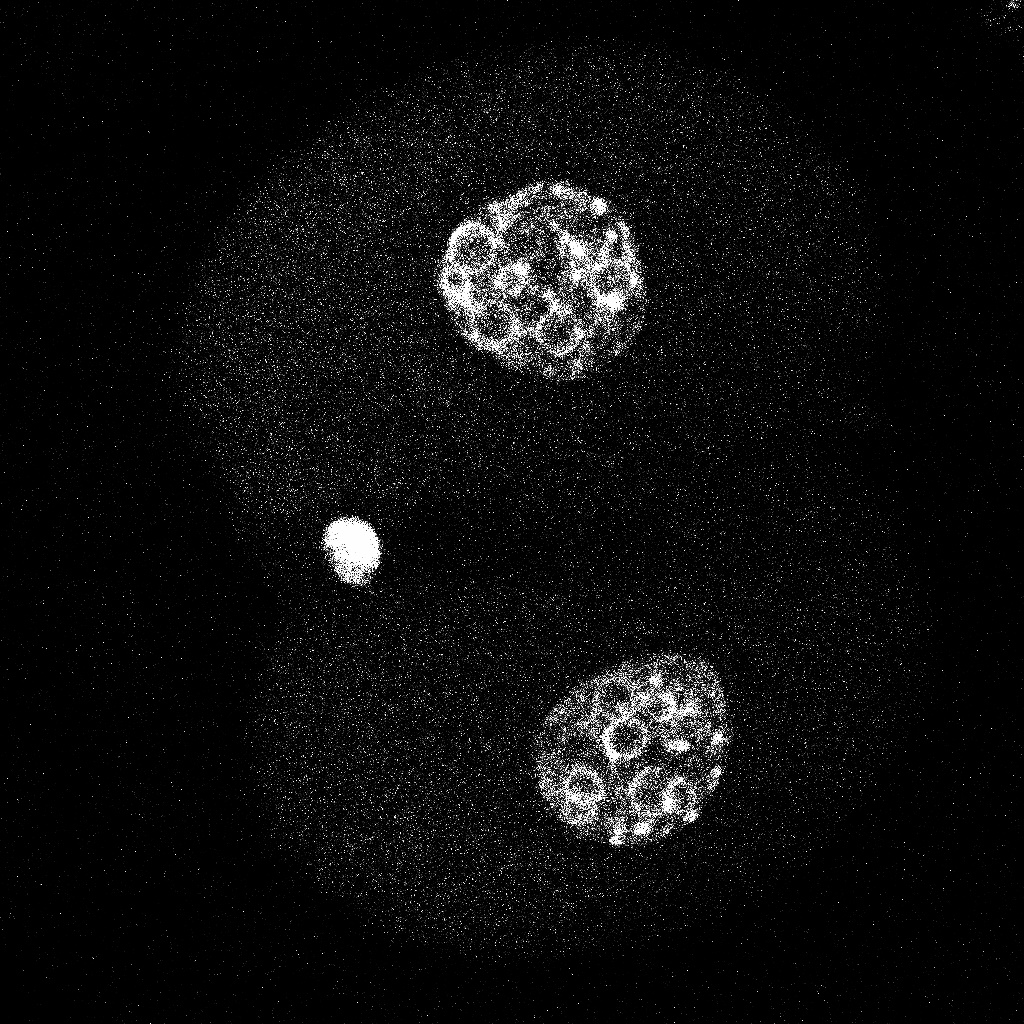

Supplement: Supplementary file 12 — Source data Fig. 2 [file 44318_2024_329_MOESM12_ESM.zip › SD figure 2/2C/Late2C_Trp_2h_DAPI.jpg]

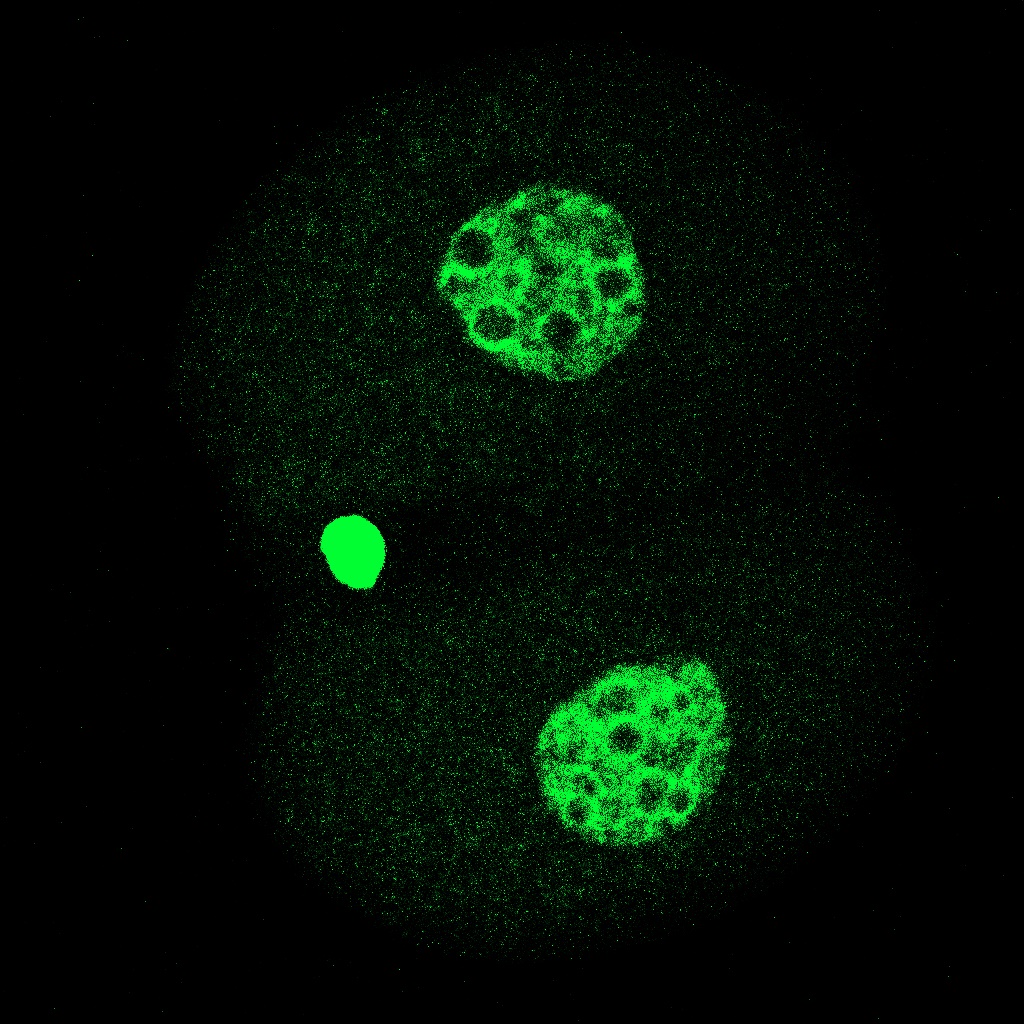

Supplement: Supplementary file 12 — Source data Fig. 2 [file 44318_2024_329_MOESM12_ESM.zip › SD figure 2/2C/Late2C_Trp_2h_H3K4me3.jpg]

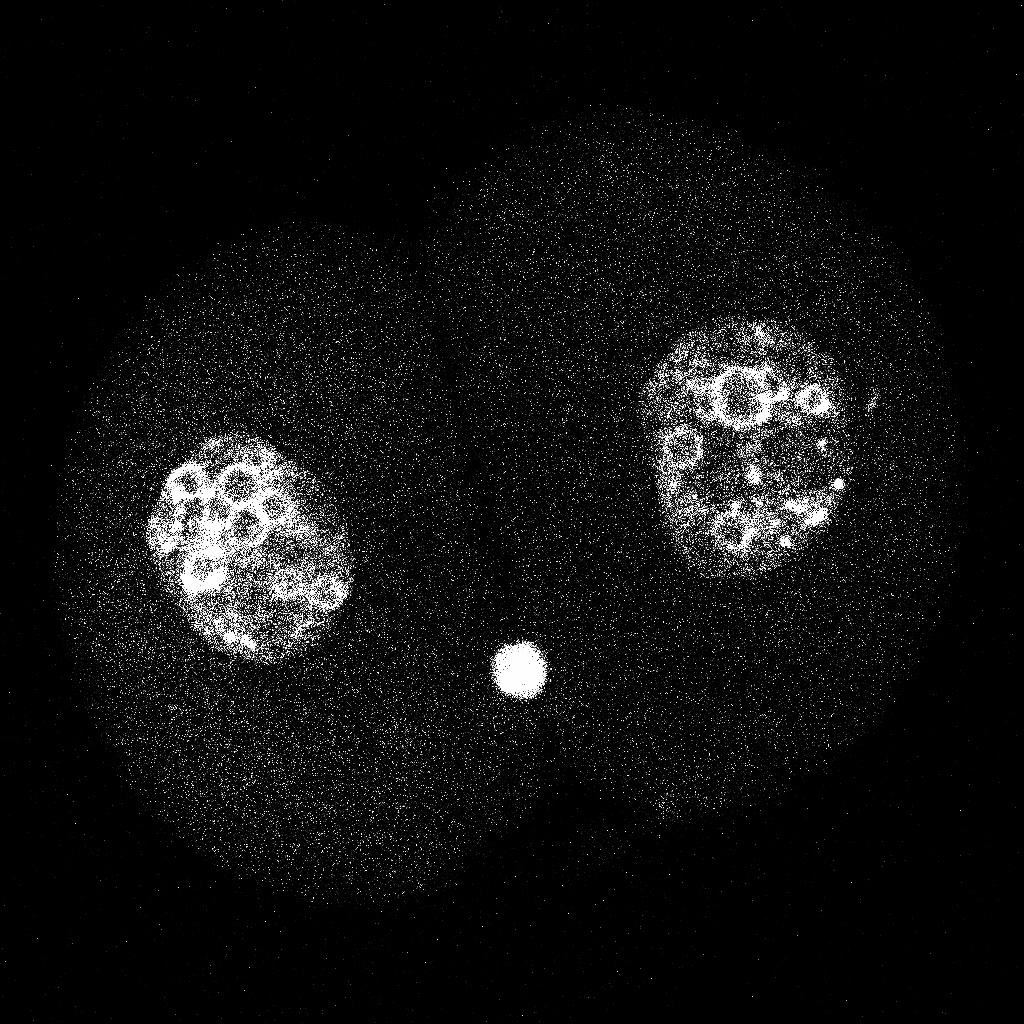

Supplement: Supplementary file 12 — Source data Fig. 2 [file 44318_2024_329_MOESM12_ESM.zip › SD figure 2/2C/Late2C_Trp_4h_DAPI.jpg]

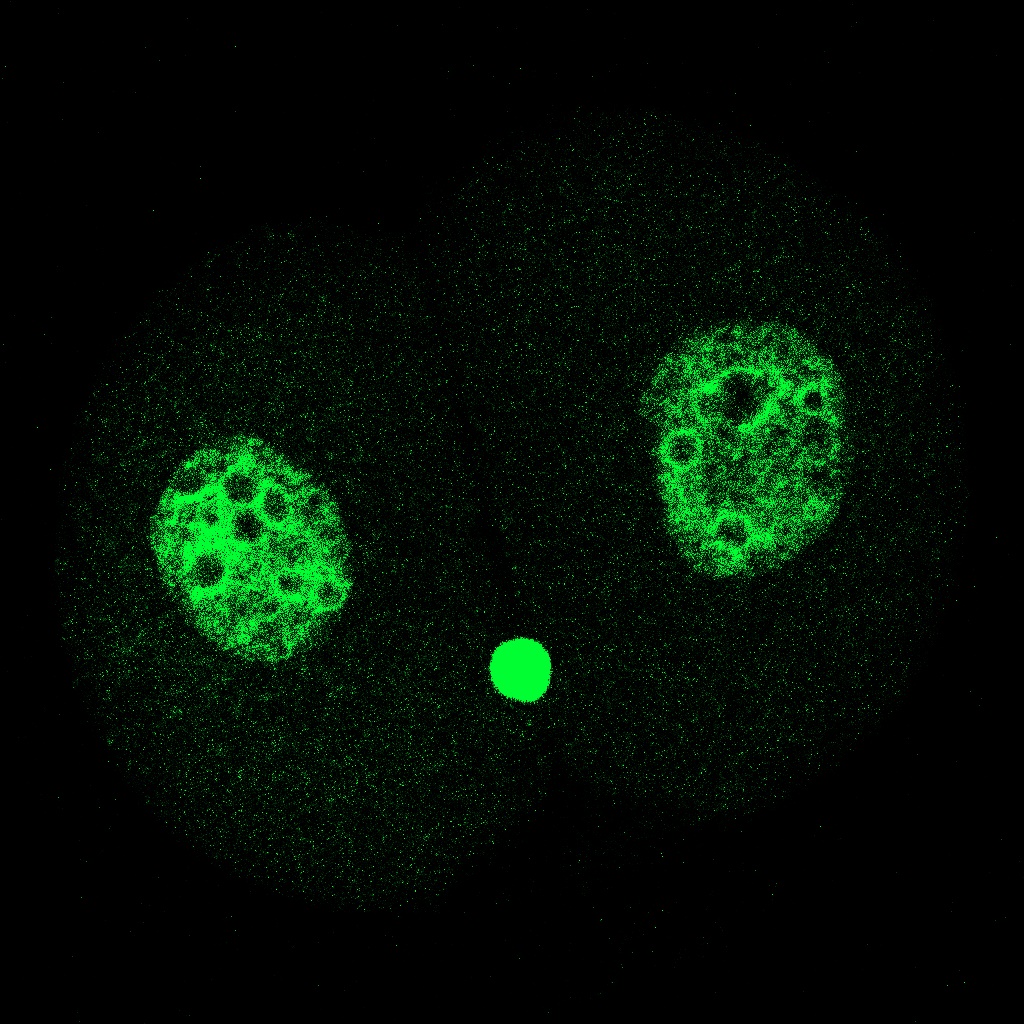

Supplement: Supplementary file 12 — Source data Fig. 2 [file 44318_2024_329_MOESM12_ESM.zip › SD figure 2/2C/Late2C_Trp_4h_H3K4me3.jpg]

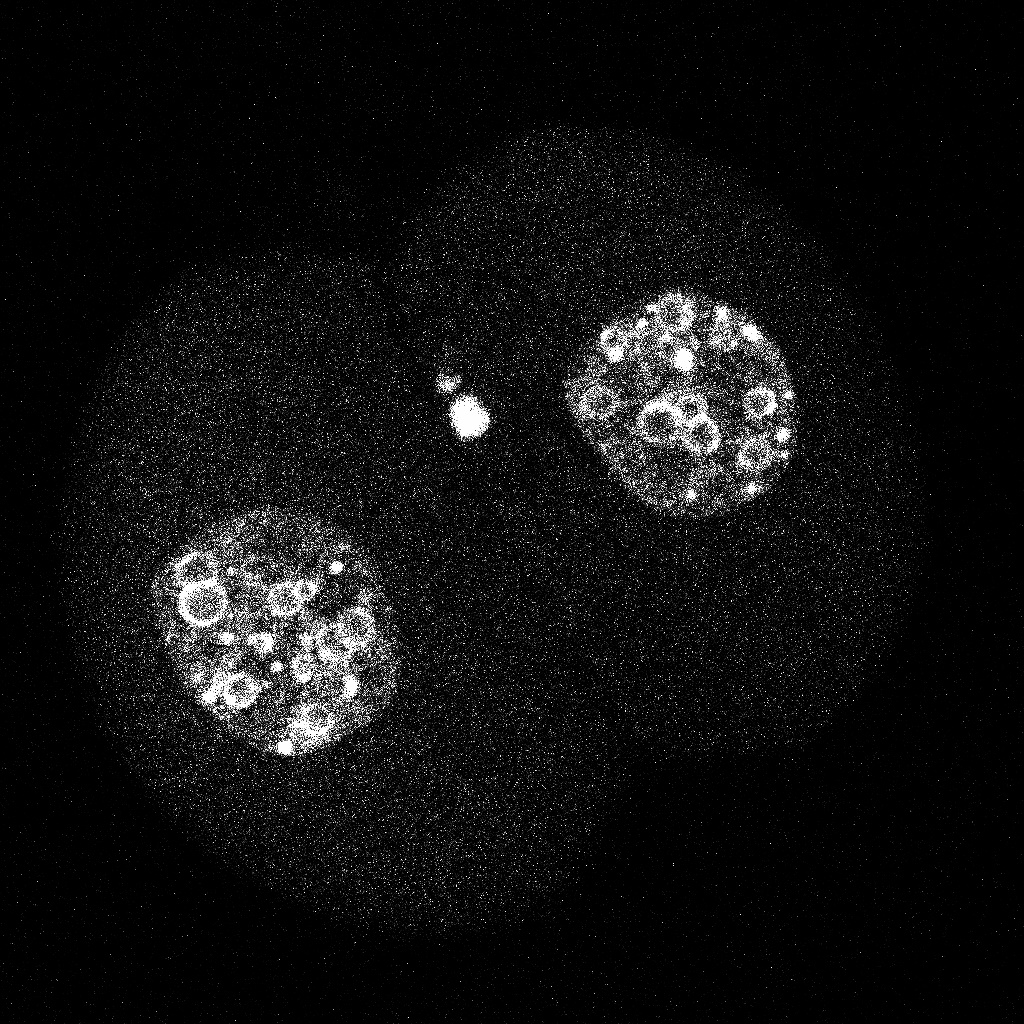

Supplement: Supplementary file 12 — Source data Fig. 2 [file 44318_2024_329_MOESM12_ESM.zip › SD figure 2/2C/Late2C_Trp_6h_DAPI.jpg]

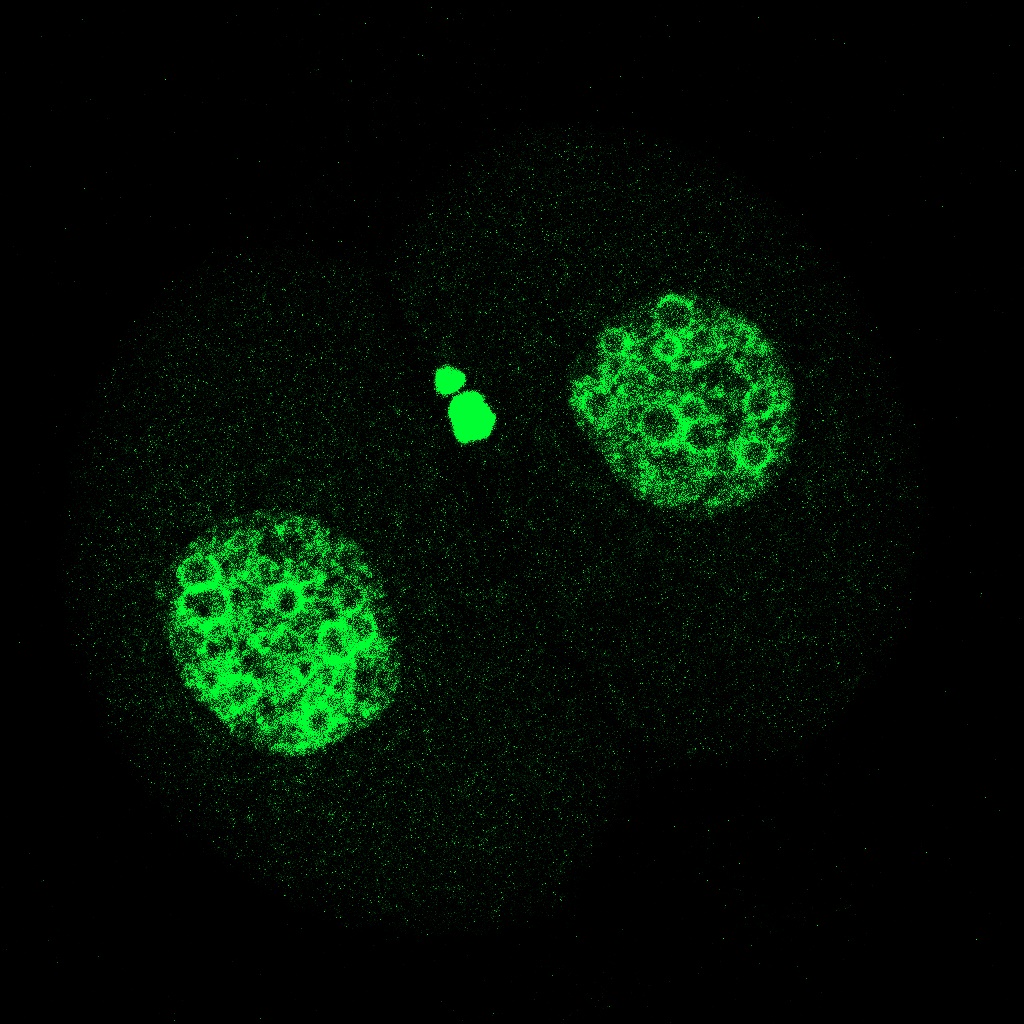

Supplement: Supplementary file 12 — Source data Fig. 2 [file 44318_2024_329_MOESM12_ESM.zip › SD figure 2/2C/Late2C_Trp_6h_H3K4me3.jpg]

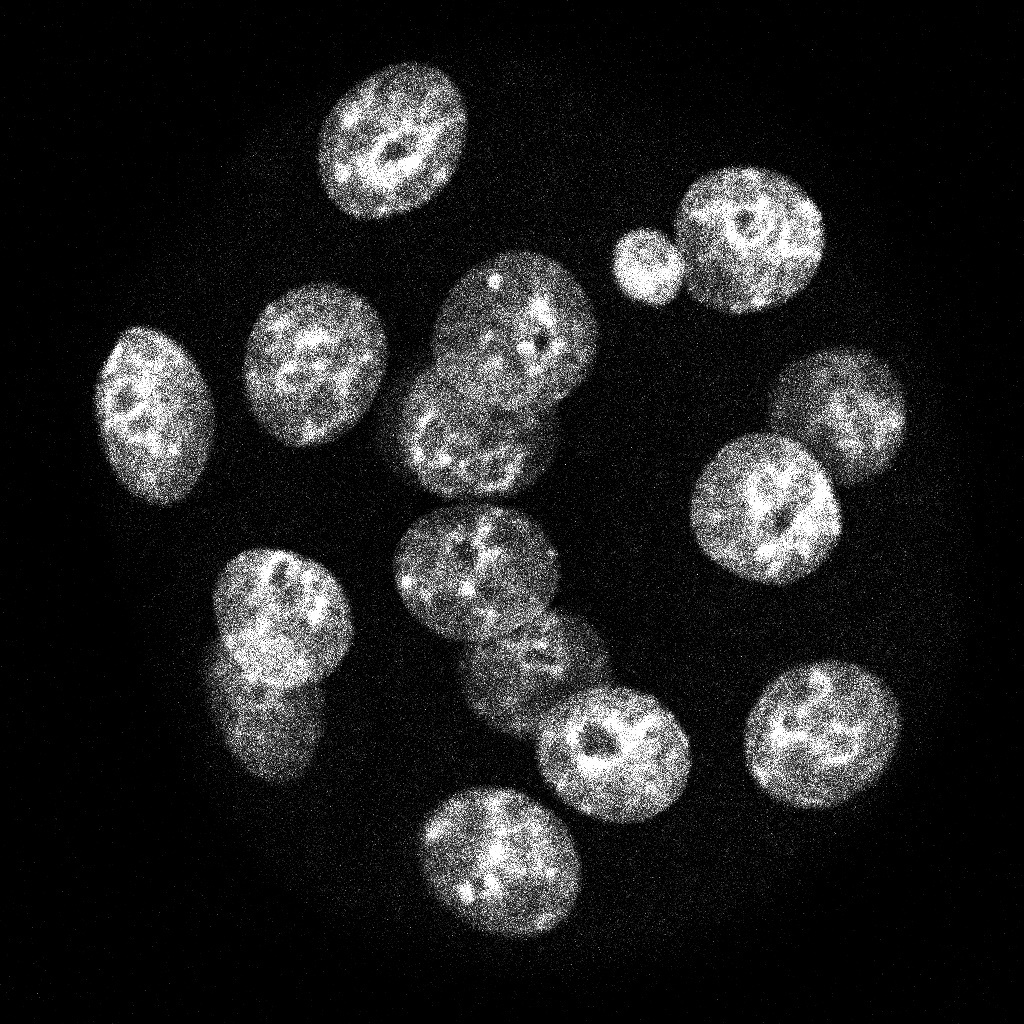

Supplement: Supplementary file 12 — Source data Fig. 2 [file 44318_2024_329_MOESM12_ESM.zip › SD figure 2/2C/Morula_Trp_0h_DAPI.jpg]

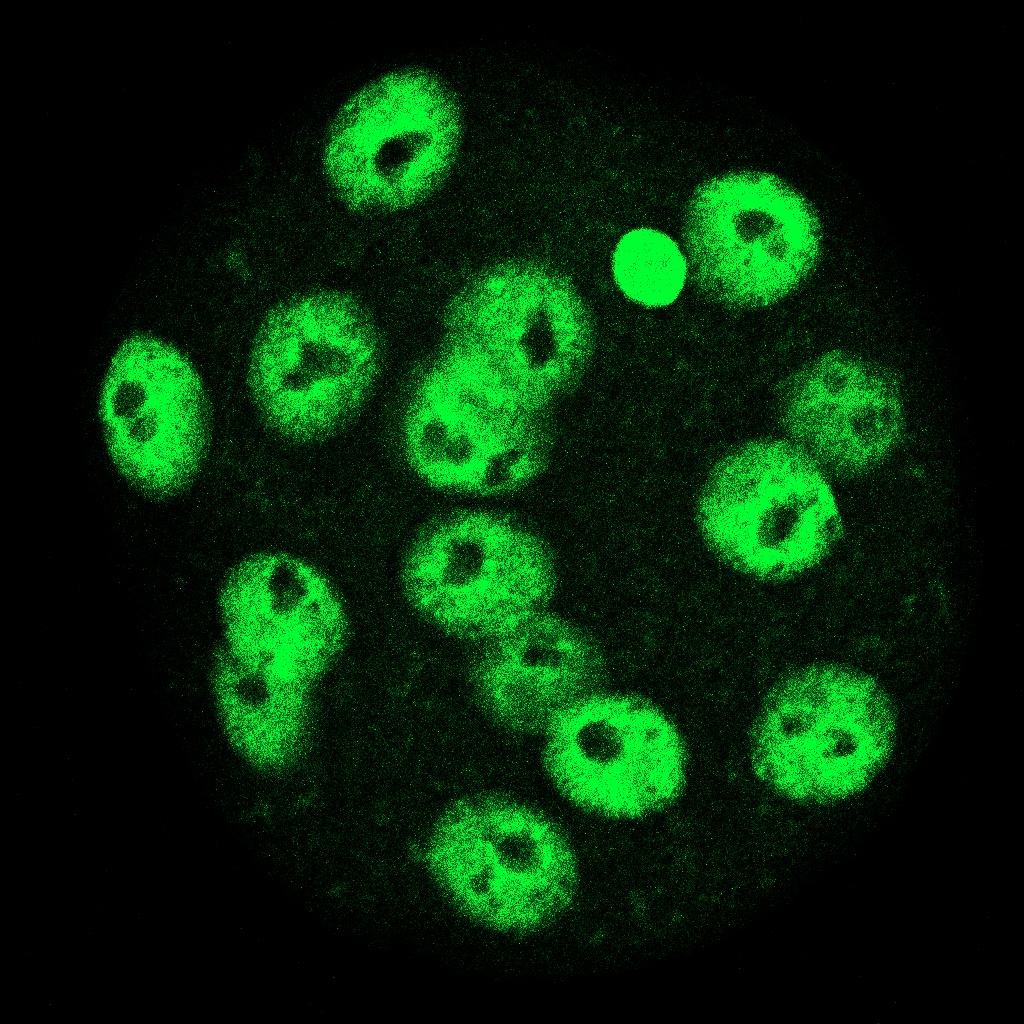

Supplement: Supplementary file 12 — Source data Fig. 2 [file 44318_2024_329_MOESM12_ESM.zip › SD figure 2/2C/Morula_Trp_0h_H3K4me3.jpg]

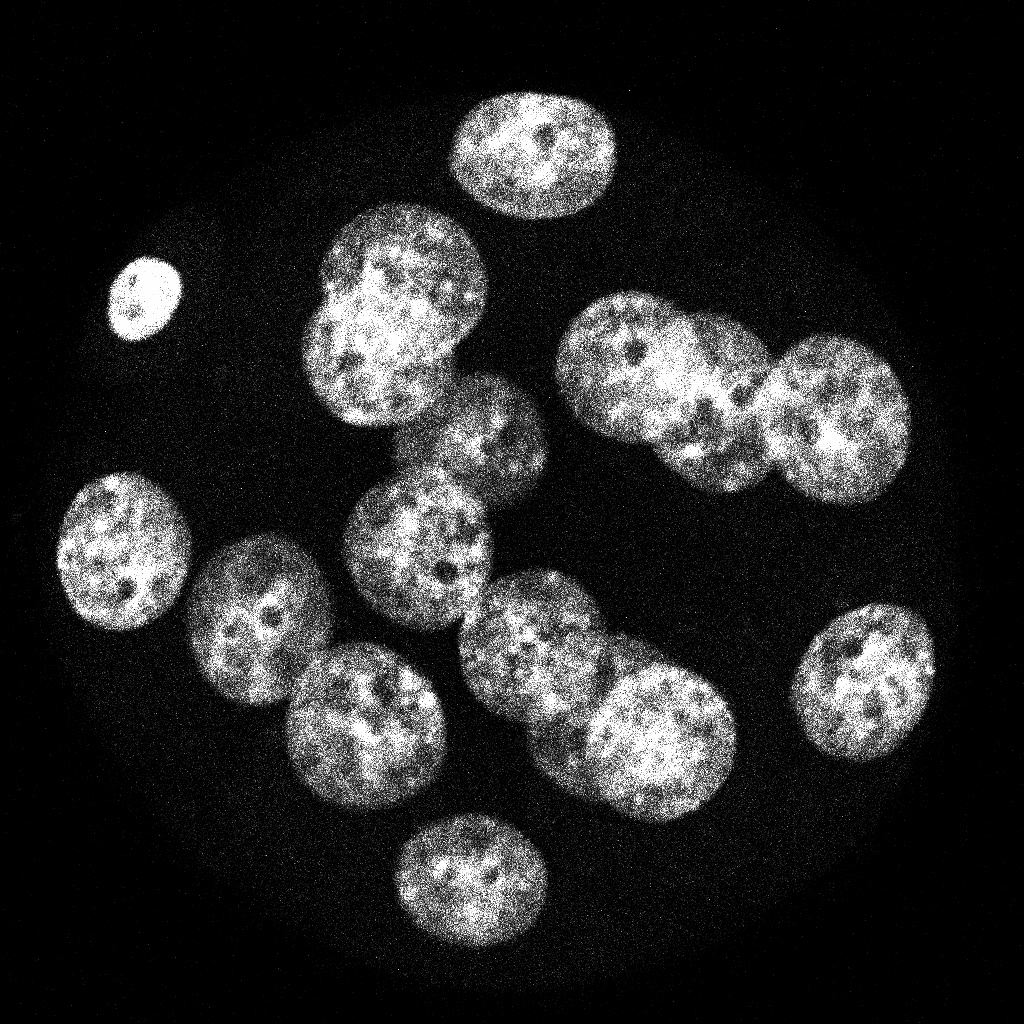

Supplement: Supplementary file 12 — Source data Fig. 2 [file 44318_2024_329_MOESM12_ESM.zip › SD figure 2/2C/Morula_Trp_2h_DAPI.jpg]

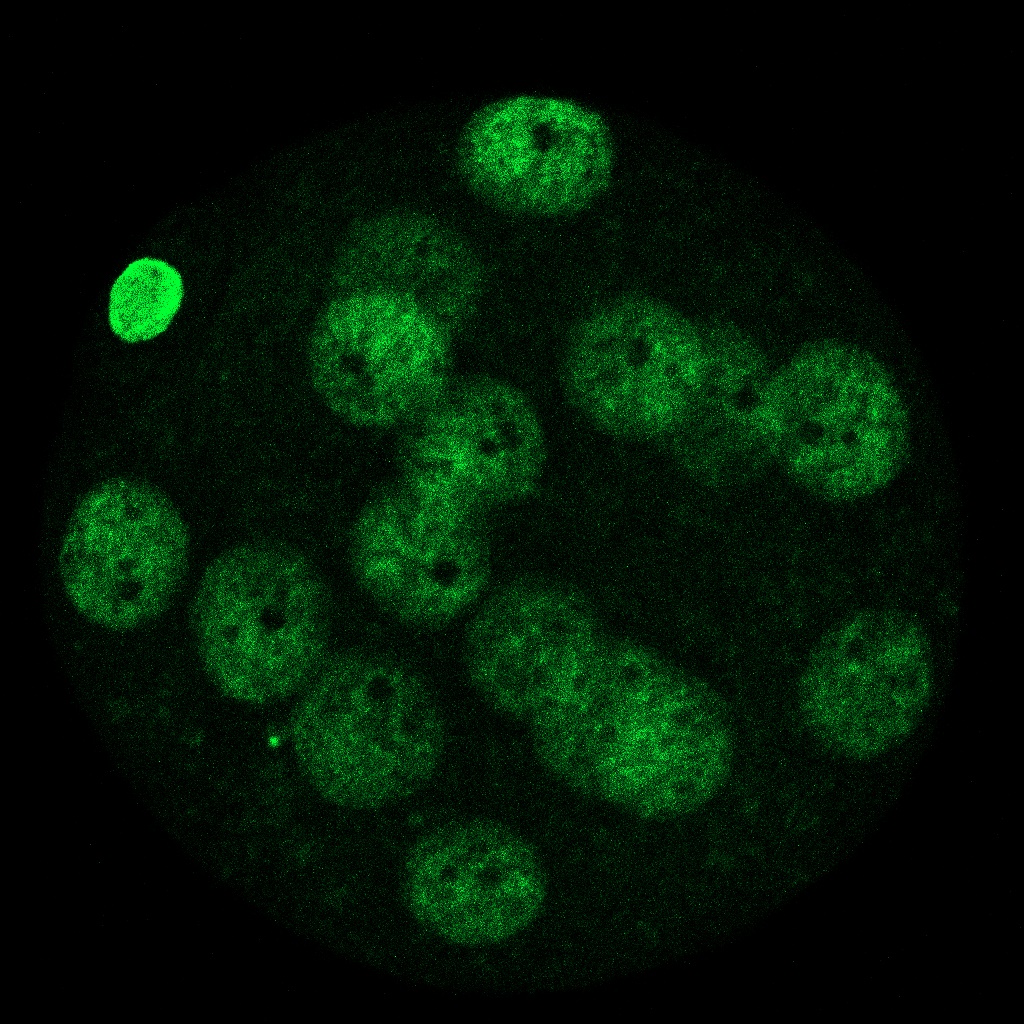

Supplement: Supplementary file 12 — Source data Fig. 2 [file 44318_2024_329_MOESM12_ESM.zip › SD figure 2/2C/Morula_Trp_2h_H3K4me3.jpg]

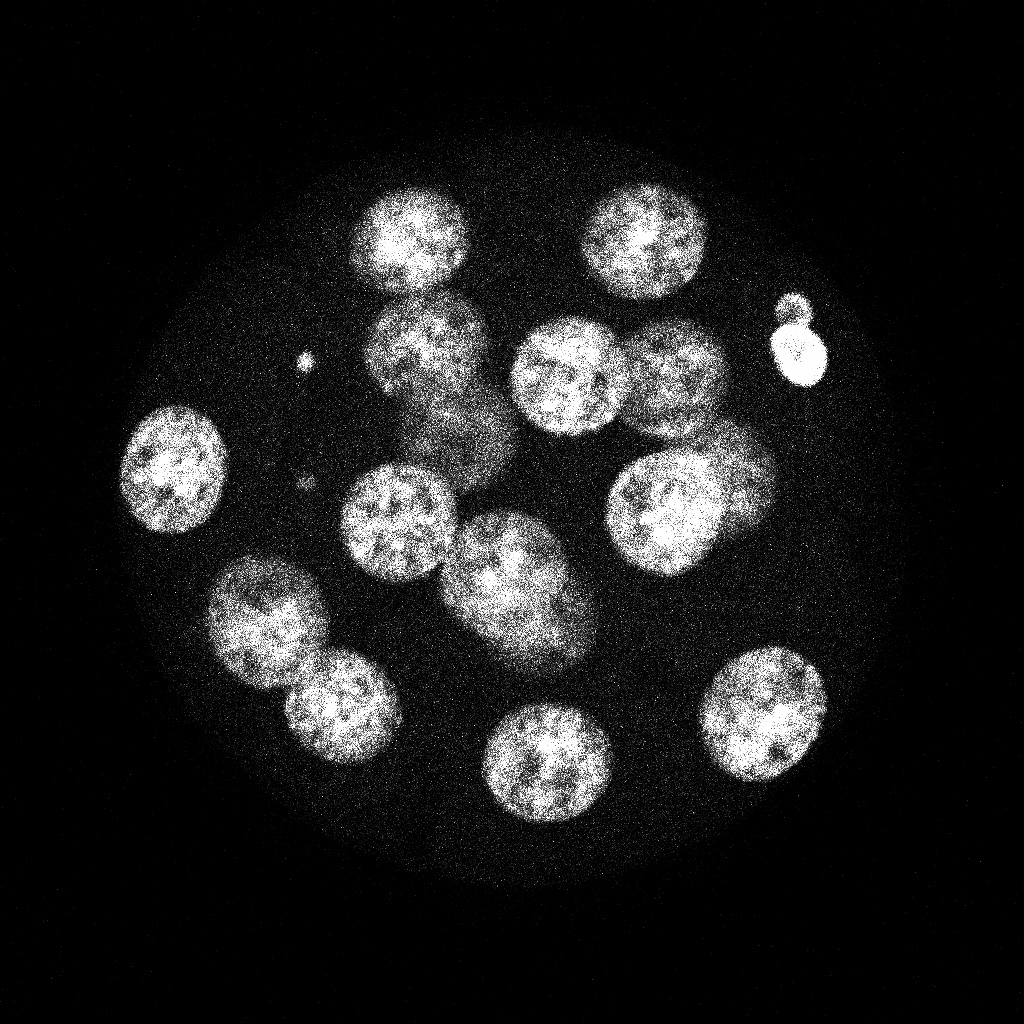

Supplement: Supplementary file 12 — Source data Fig. 2 [file 44318_2024_329_MOESM12_ESM.zip › SD figure 2/2C/Morula_Trp_4h_DAPI.jpg]

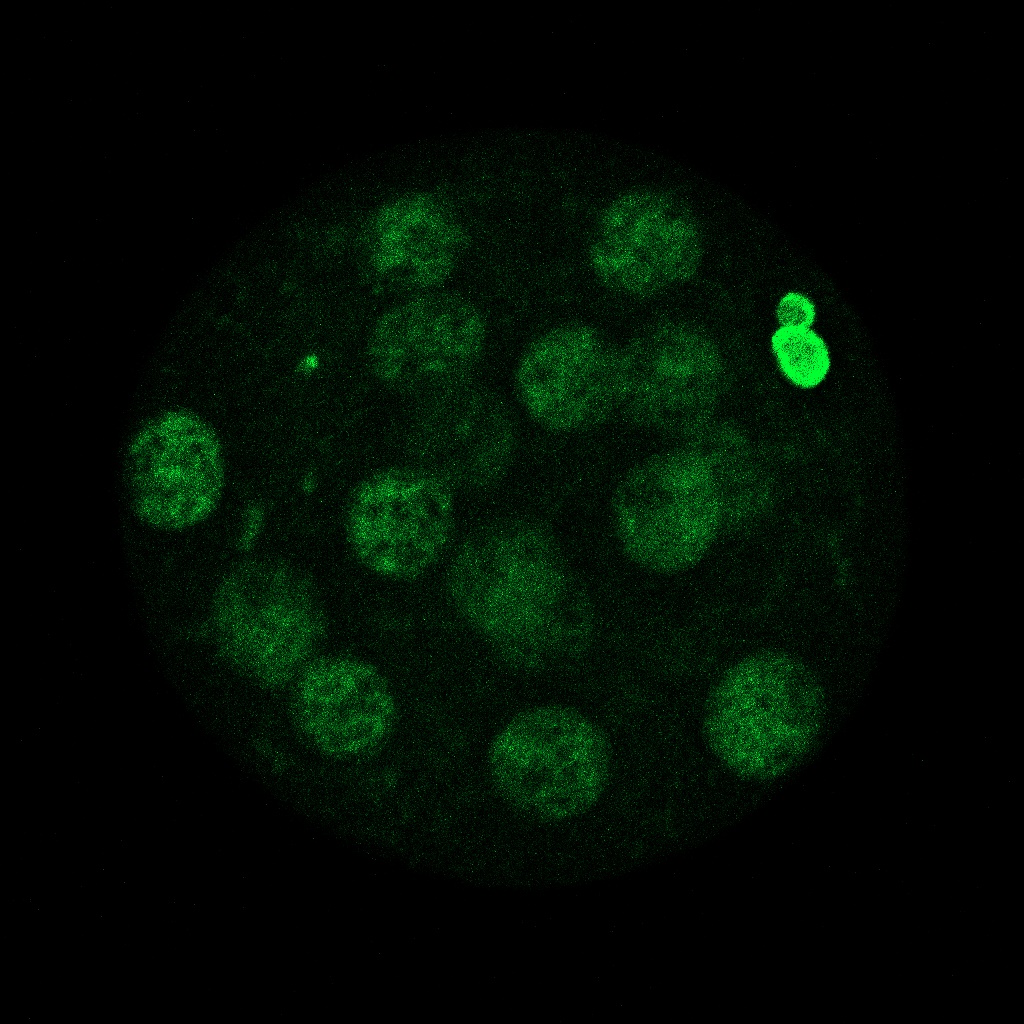

Supplement: Supplementary file 12 — Source data Fig. 2 [file 44318_2024_329_MOESM12_ESM.zip › SD figure 2/2C/Morula_Trp_4h_H3K4me3.jpg]

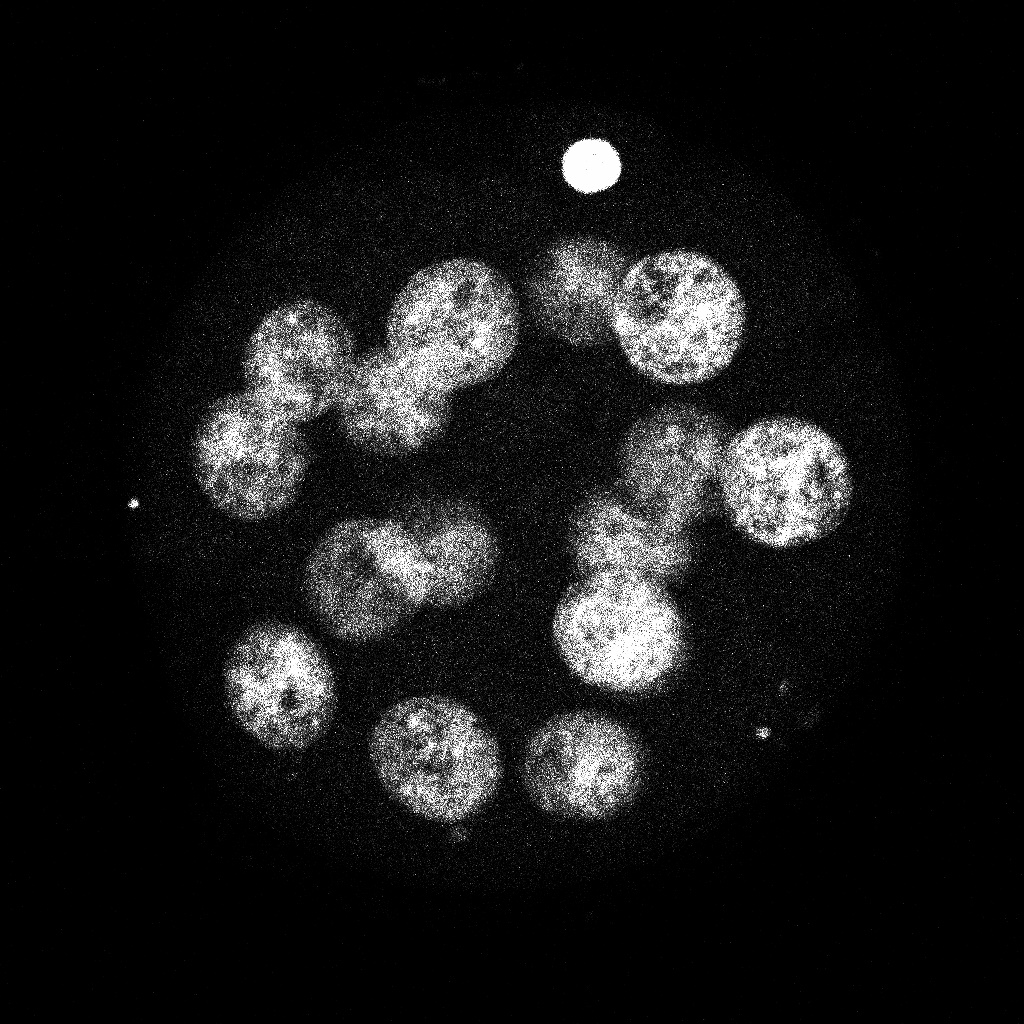

Supplement: Supplementary file 12 — Source data Fig. 2 [file 44318_2024_329_MOESM12_ESM.zip › SD figure 2/2C/Morula_Trp_6h_DAPI.jpg]

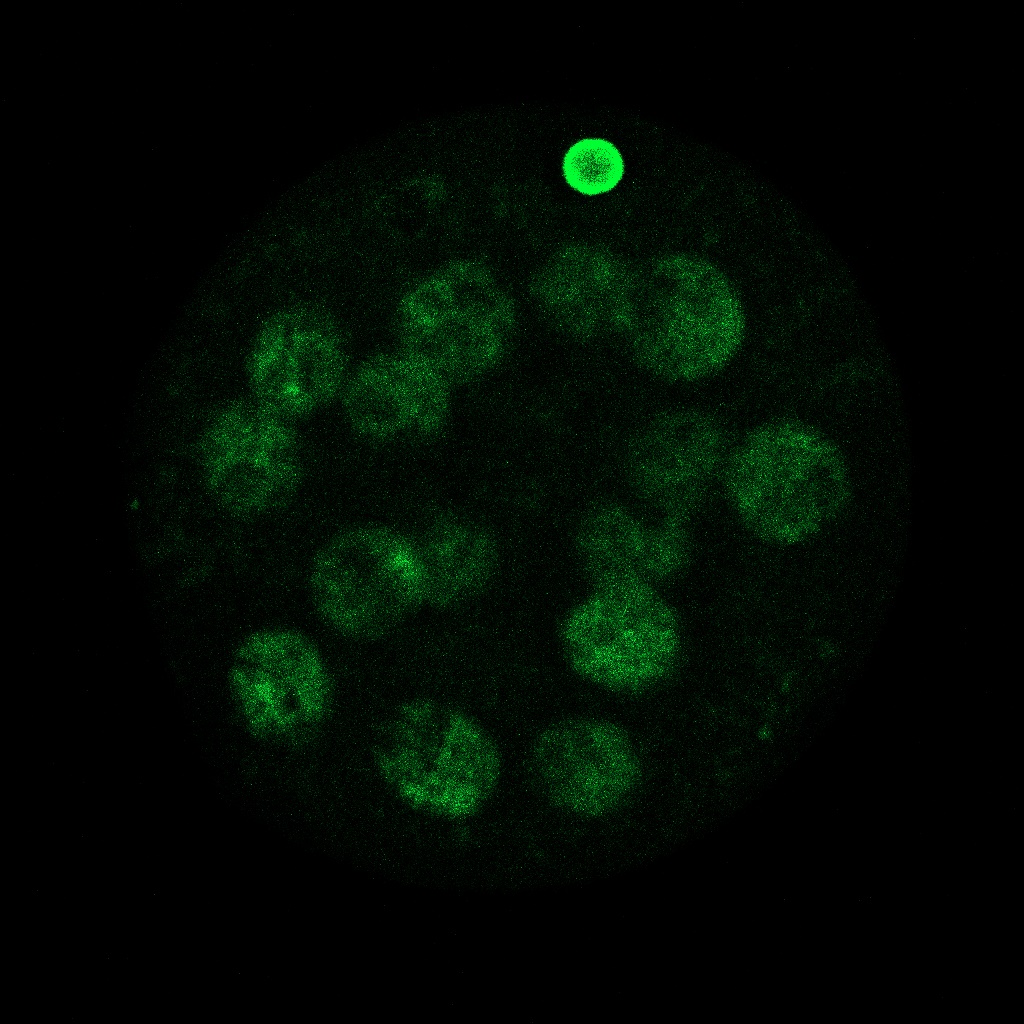

Supplement: Supplementary file 12 — Source data Fig. 2 [file 44318_2024_329_MOESM12_ESM.zip › SD figure 2/2C/Morula_Trp_6h_H3K4me3.jpg]

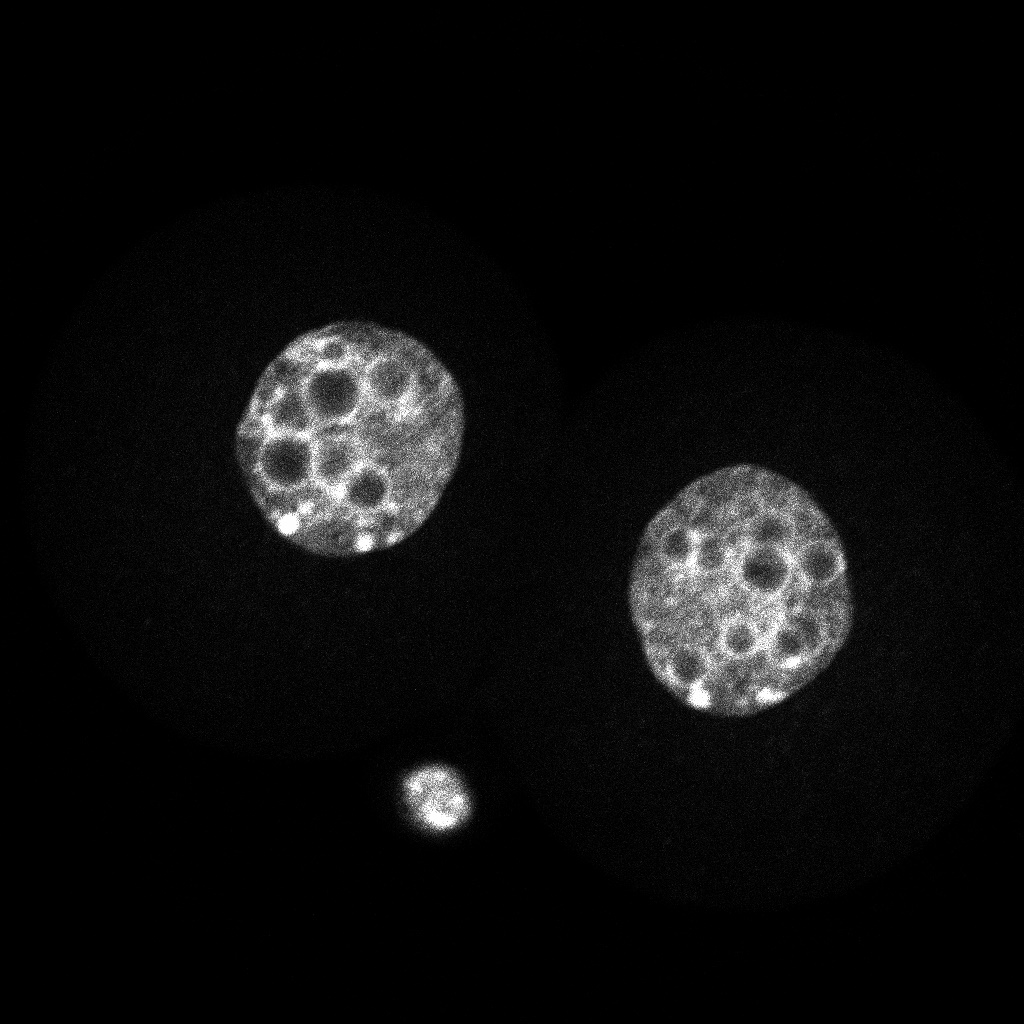

Supplement: Supplementary file 13 — Source data Fig. 3 [file 44318_2024_329_MOESM13_ESM.zip › SD figure 3/3A/Early2C_Control_DAPI.jpg]

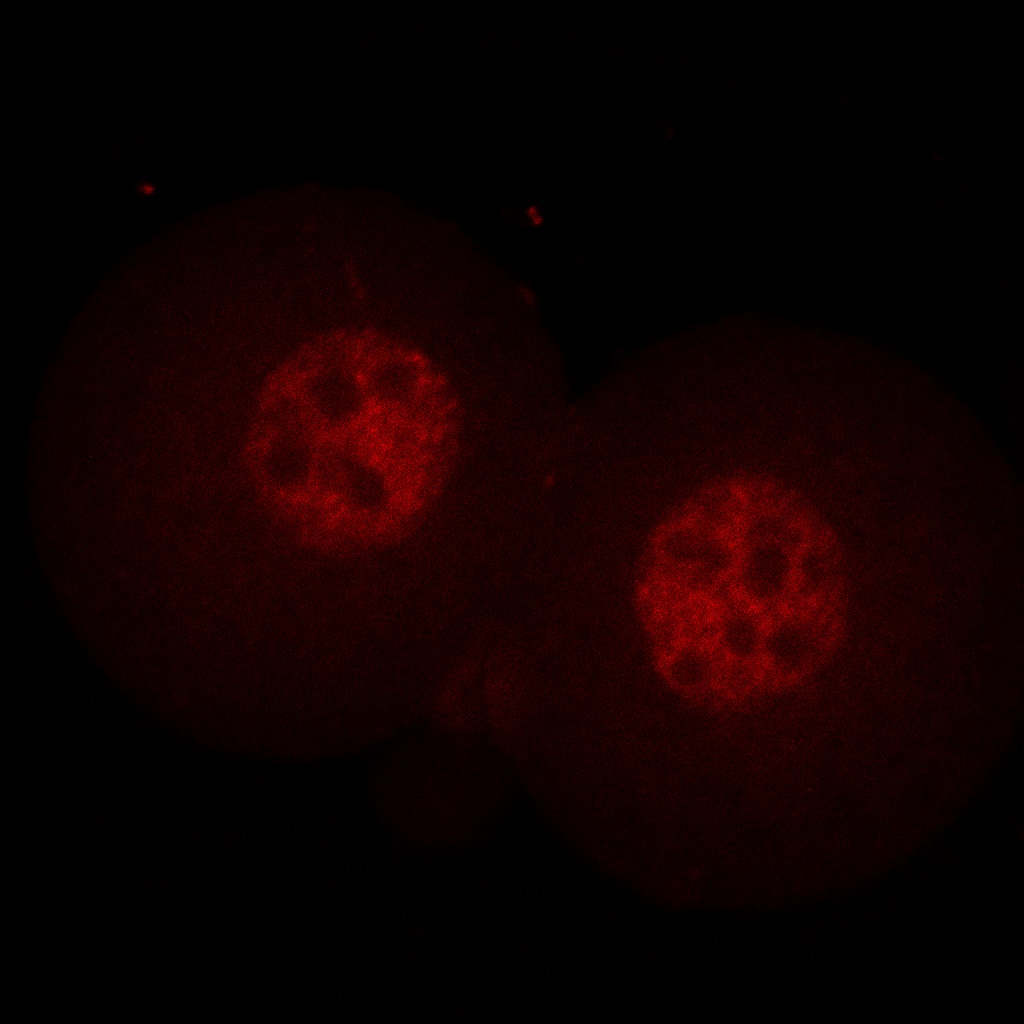

Supplement: Supplementary file 13 — Source data Fig. 3 [file 44318_2024_329_MOESM13_ESM.zip › SD figure 3/3A/Early2C_Control_EU.jpg]

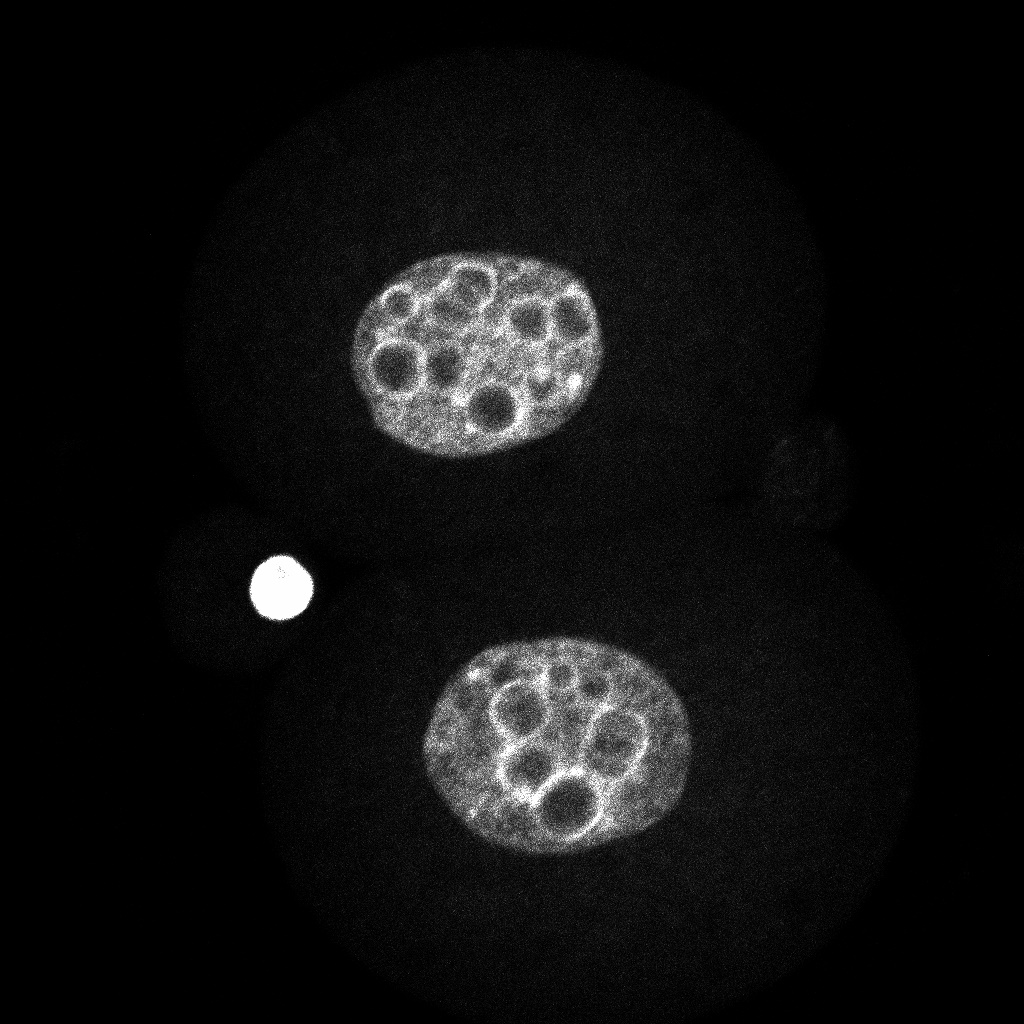

Supplement: Supplementary file 13 — Source data Fig. 3 [file 44318_2024_329_MOESM13_ESM.zip › SD figure 3/3A/Early2C_Mll2 KD_DAPI.jpg]

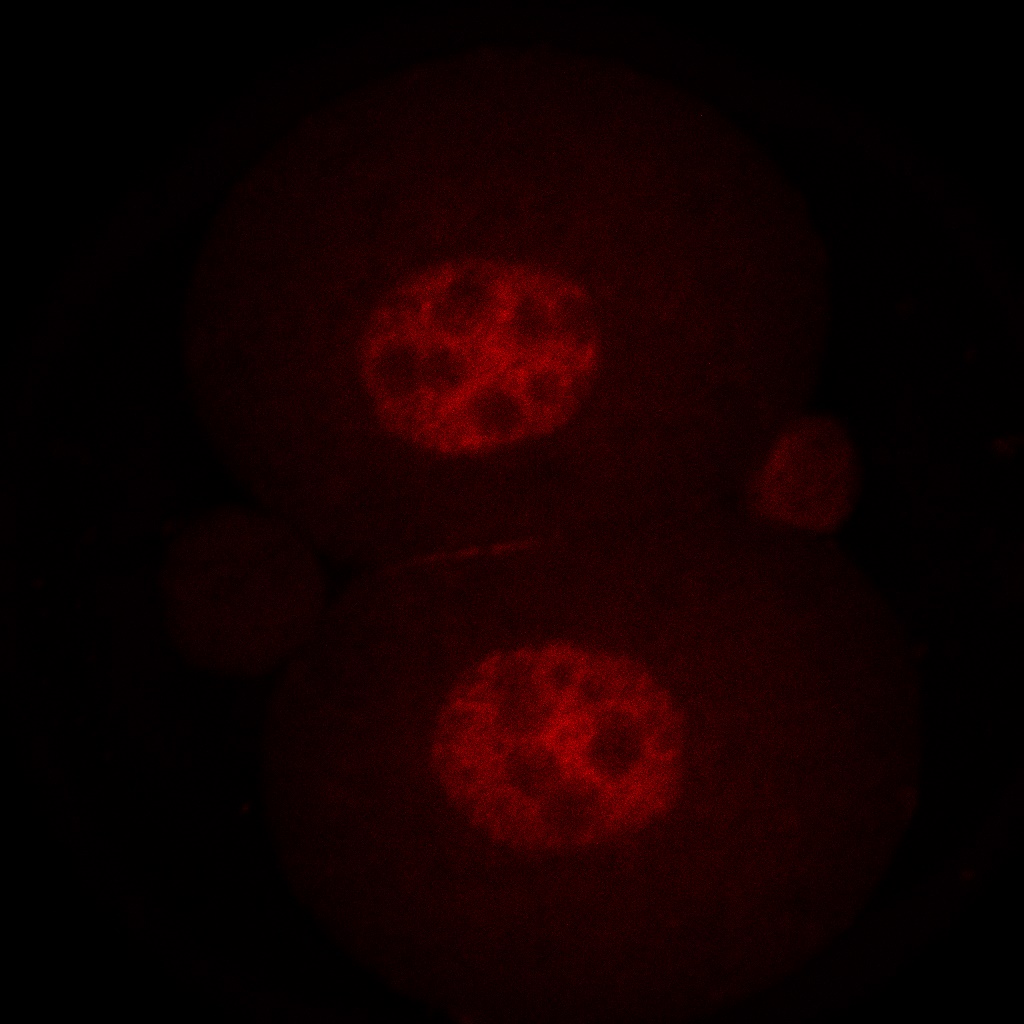

Supplement: Supplementary file 13 — Source data Fig. 3 [file 44318_2024_329_MOESM13_ESM.zip › SD figure 3/3A/Early2C_Mll2 KD_EU.jpg]

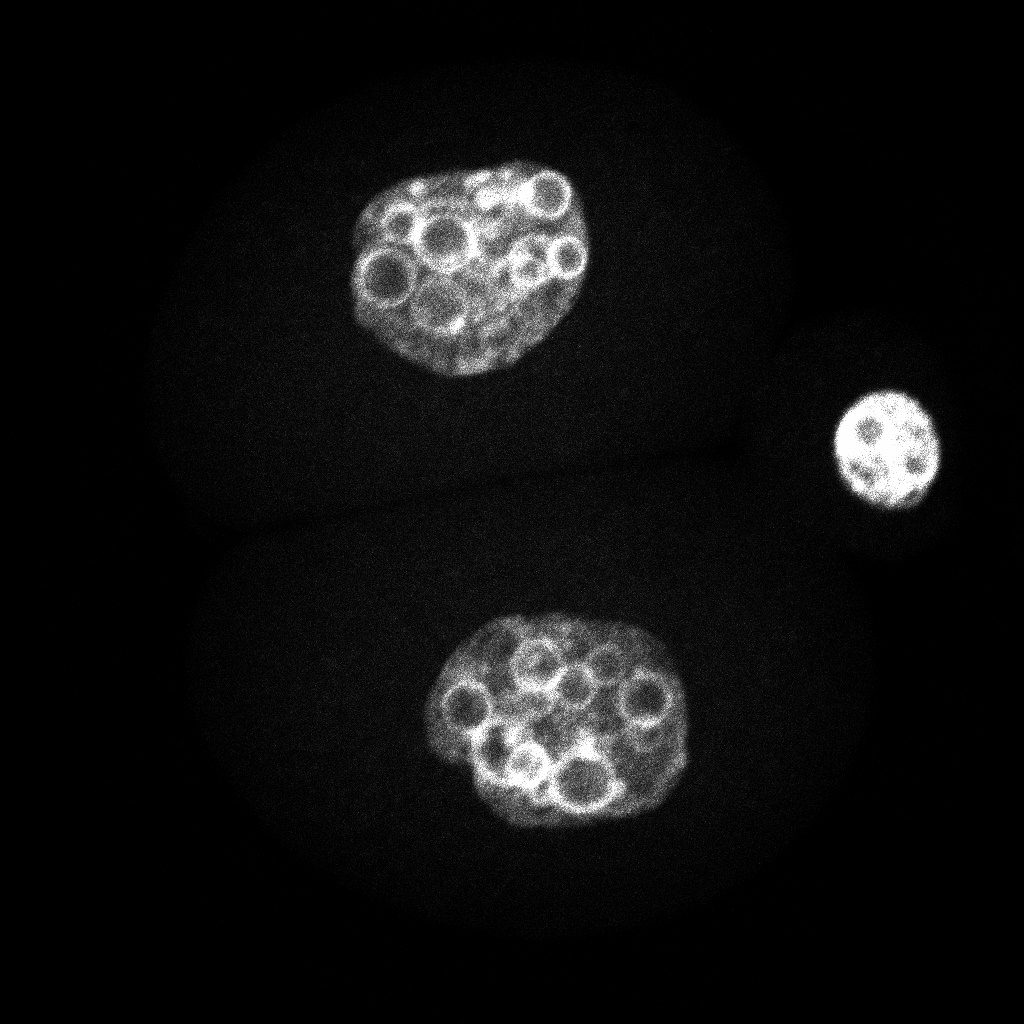

Supplement: Supplementary file 13 — Source data Fig. 3 [file 44318_2024_329_MOESM13_ESM.zip › SD figure 3/3A/Late2C_Control_DAPI.jpg]

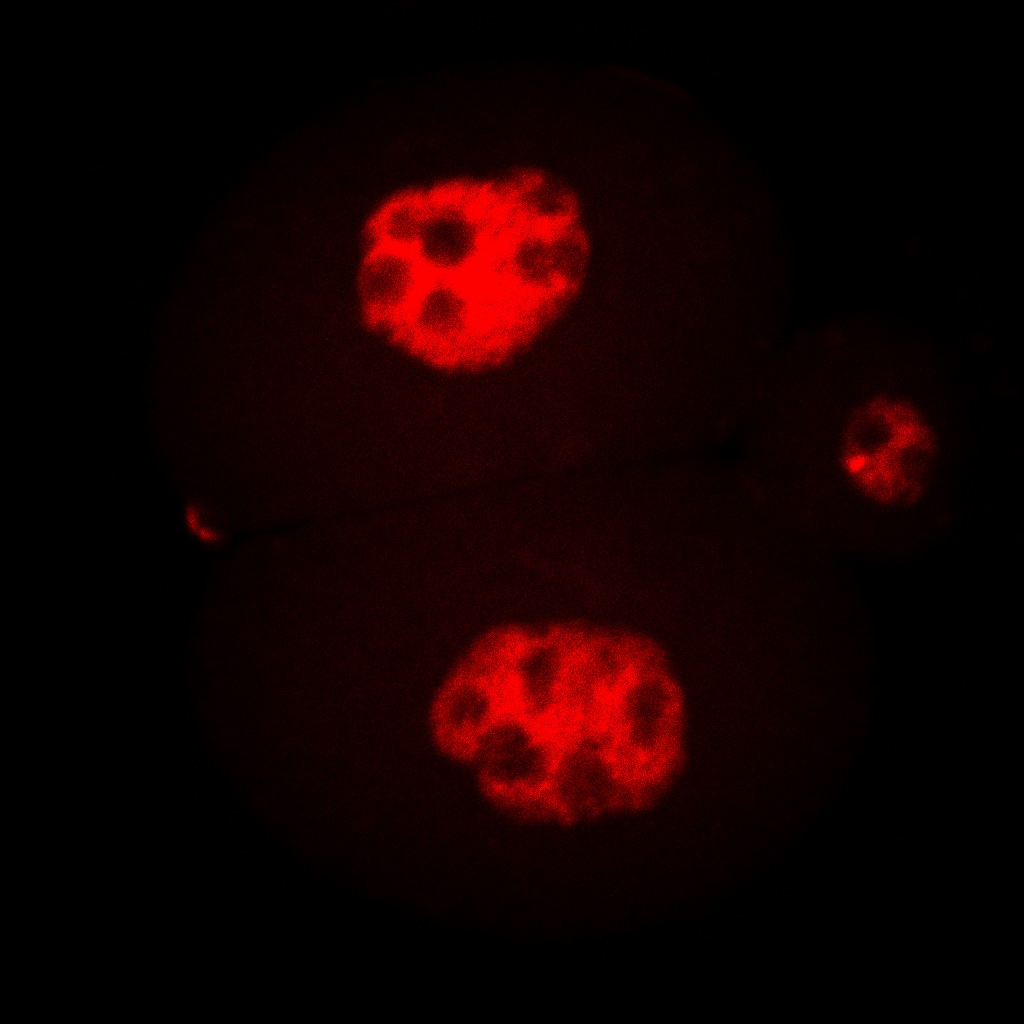

Supplement: Supplementary file 13 — Source data Fig. 3 [file 44318_2024_329_MOESM13_ESM.zip › SD figure 3/3A/Late2C_Control_EU.jpg]

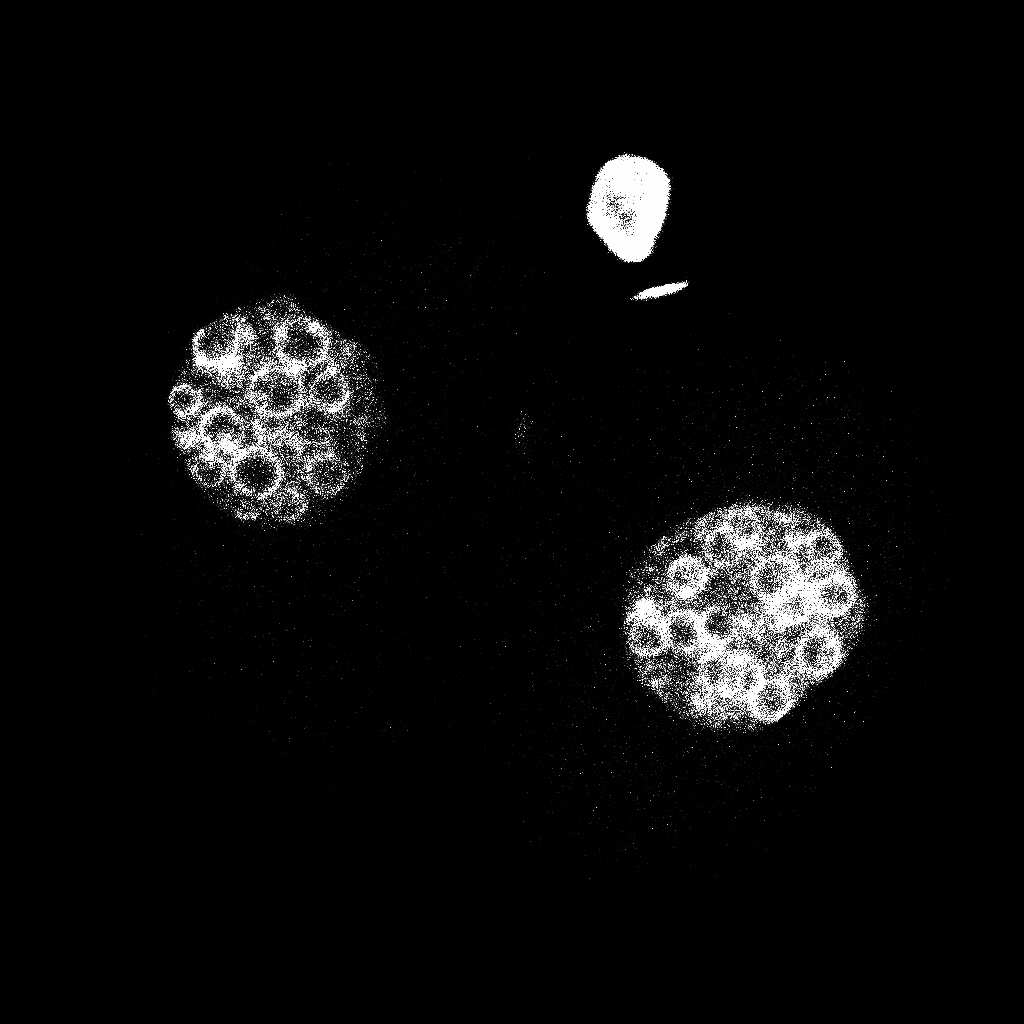

Supplement: Supplementary file 13 — Source data Fig. 3 [file 44318_2024_329_MOESM13_ESM.zip › SD figure 3/3A/Late2C_Mll2 KD_DAPI.jpg]

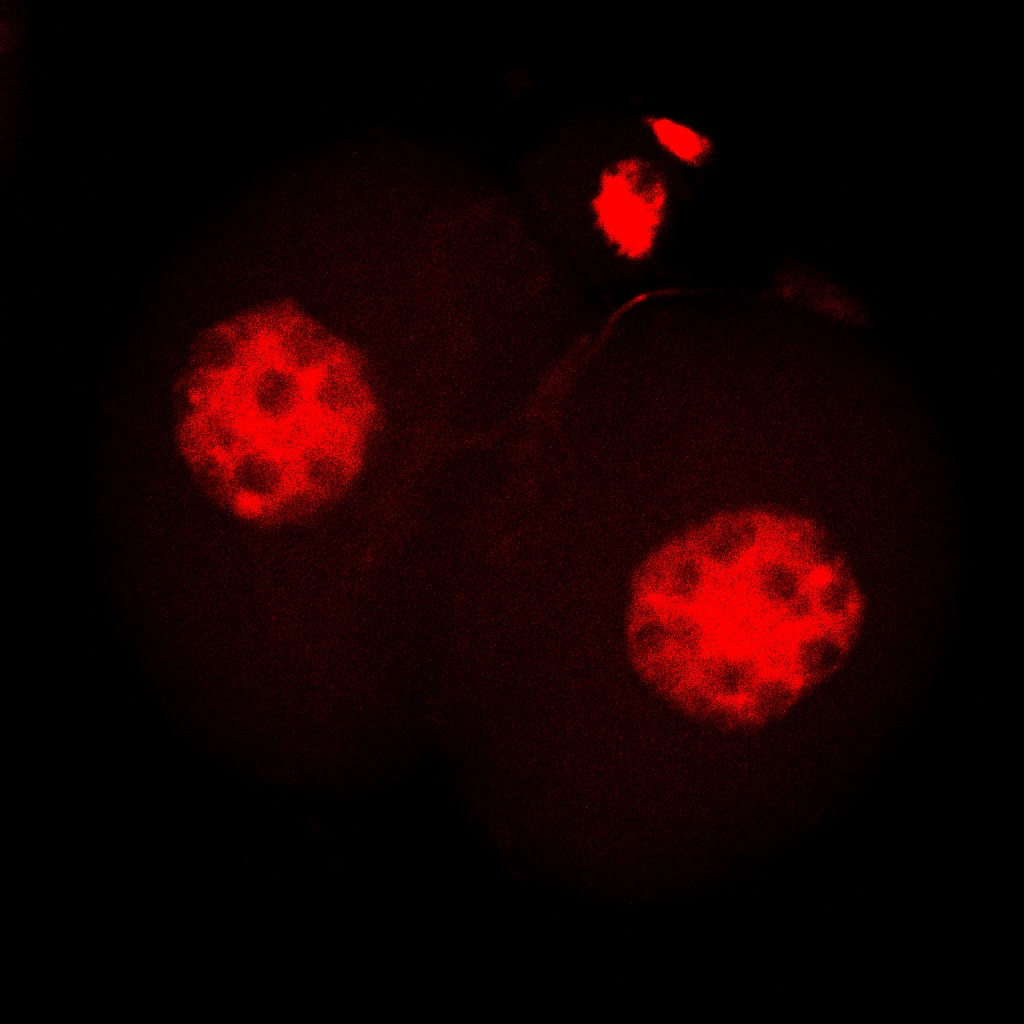

Supplement: Supplementary file 13 — Source data Fig. 3 [file 44318_2024_329_MOESM13_ESM.zip › SD figure 3/3A/Late2C_Mll2 KD_EU.jpg]

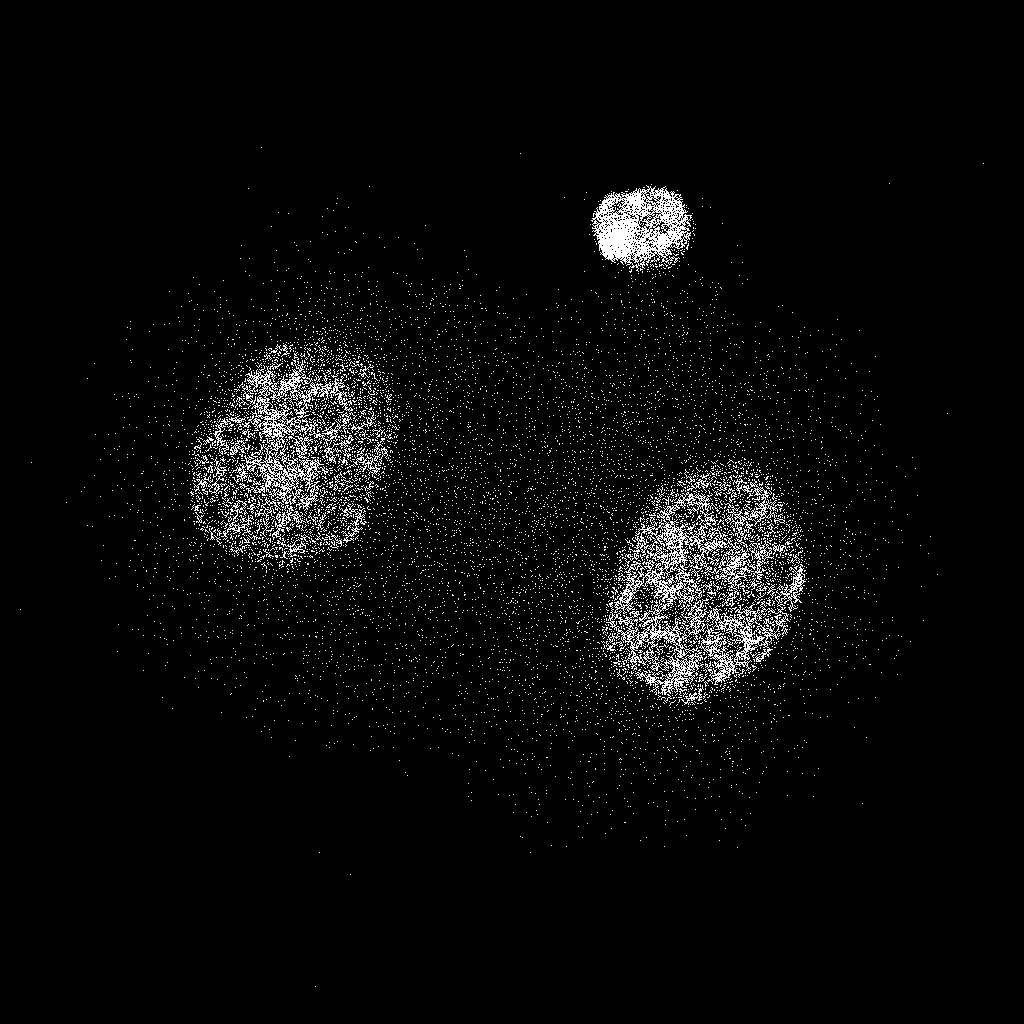

Supplement: Supplementary file 13 — Source data Fig. 3 [file 44318_2024_329_MOESM13_ESM.zip › SD figure 3/3E/Early2C_Control_DAPI.jpg]

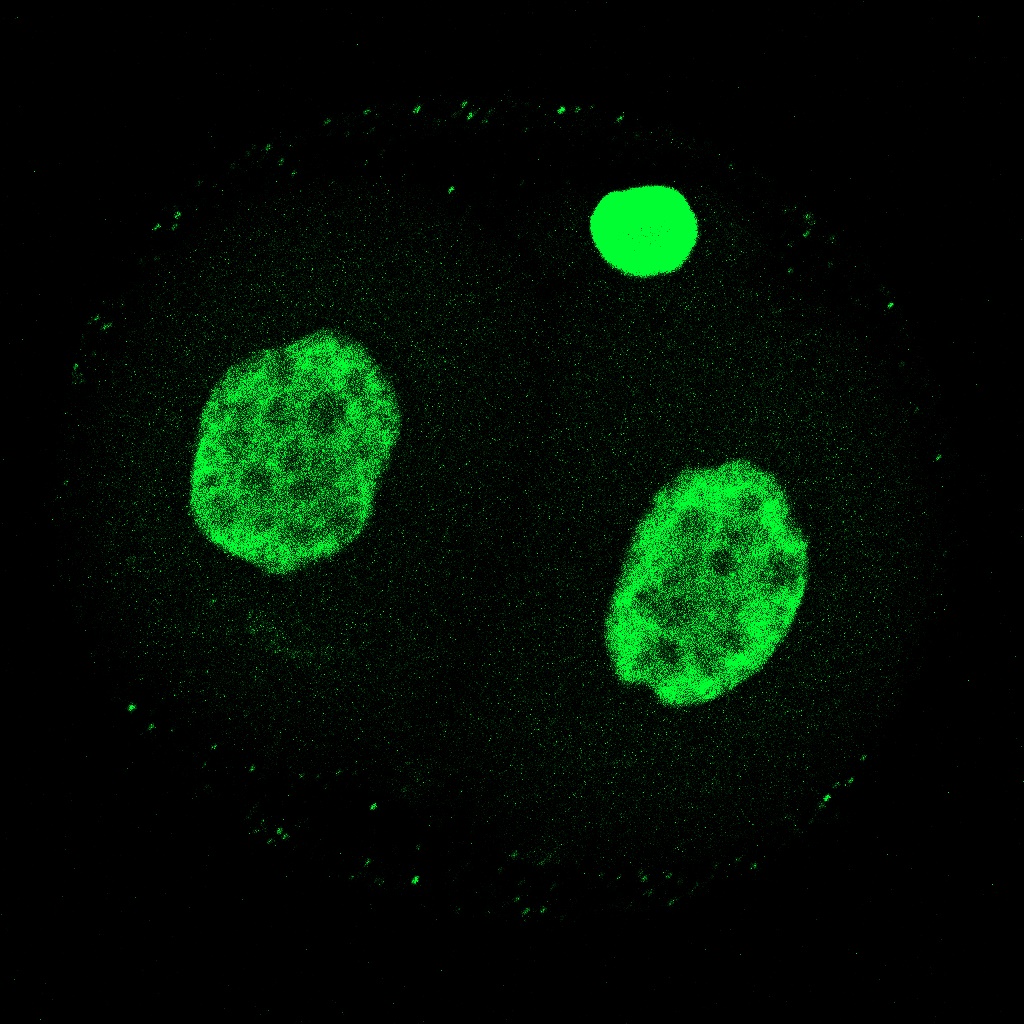

Supplement: Supplementary file 13 — Source data Fig. 3 [file 44318_2024_329_MOESM13_ESM.zip › SD figure 3/3E/Early2C_Control_H3K4me3.jpg]

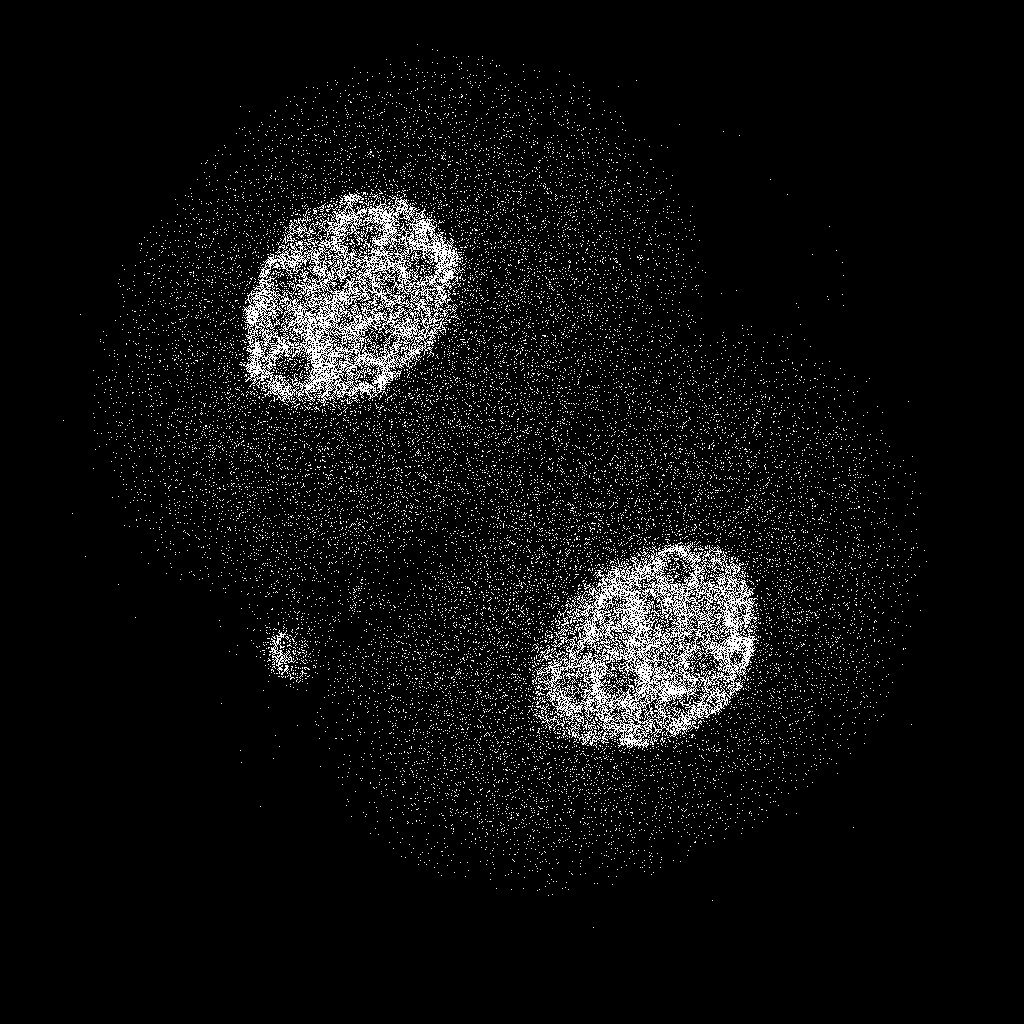

Supplement: Supplementary file 13 — Source data Fig. 3 [file 44318_2024_329_MOESM13_ESM.zip › SD figure 3/3E/Early2C_Kdm5b MUT_DAPI.jpg]

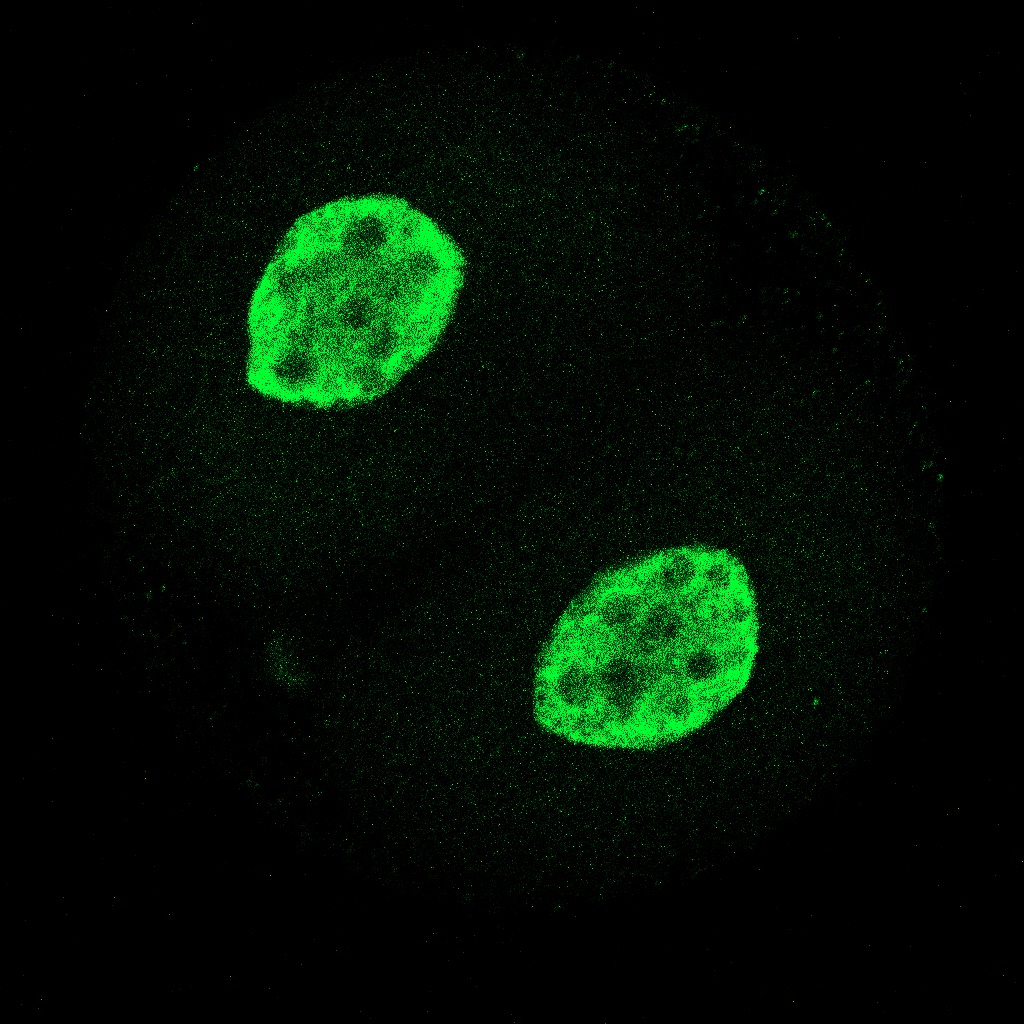

Supplement: Supplementary file 13 — Source data Fig. 3 [file 44318_2024_329_MOESM13_ESM.zip › SD figure 3/3E/Early2C_Kdm5b MUT_H3K4me3.jpg]

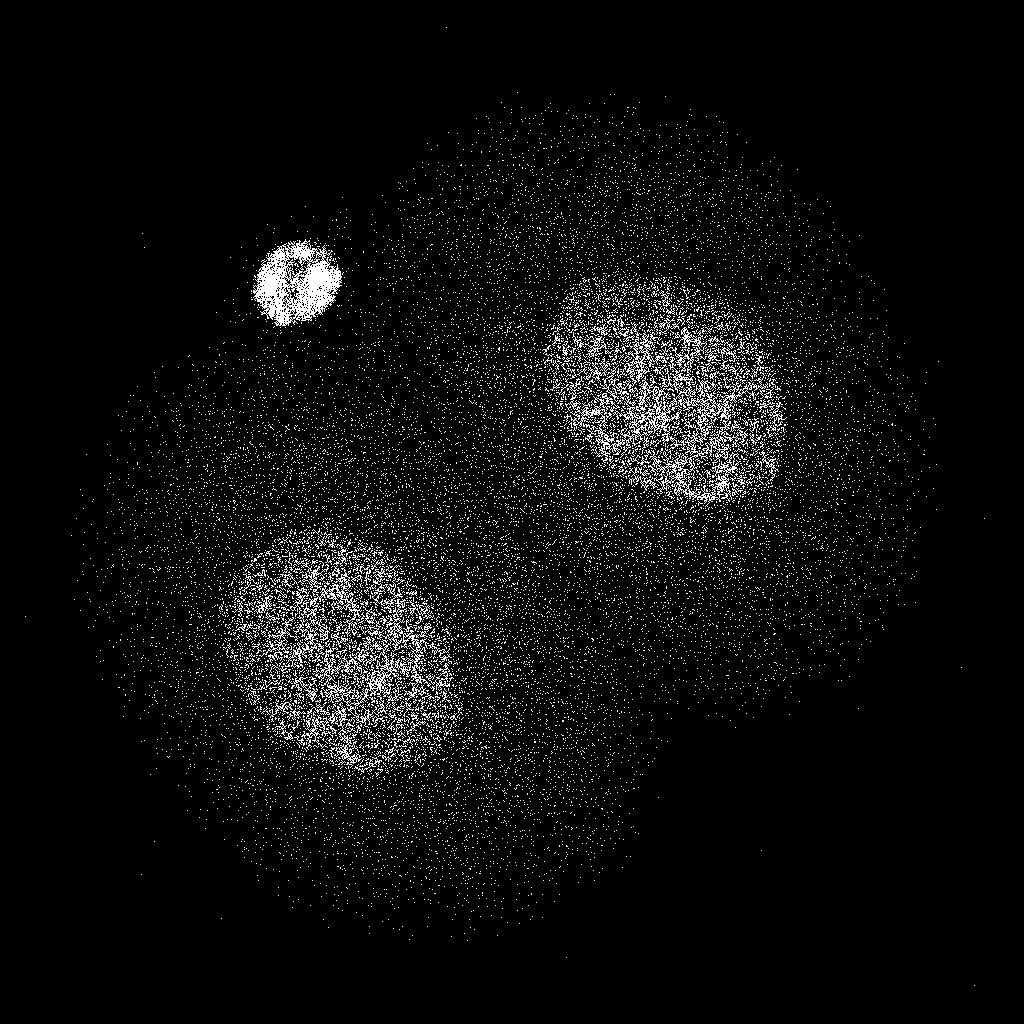

Supplement: Supplementary file 13 — Source data Fig. 3 [file 44318_2024_329_MOESM13_ESM.zip › SD figure 3/3E/Early2C_Kdm5b WT_DAPI.jpg]

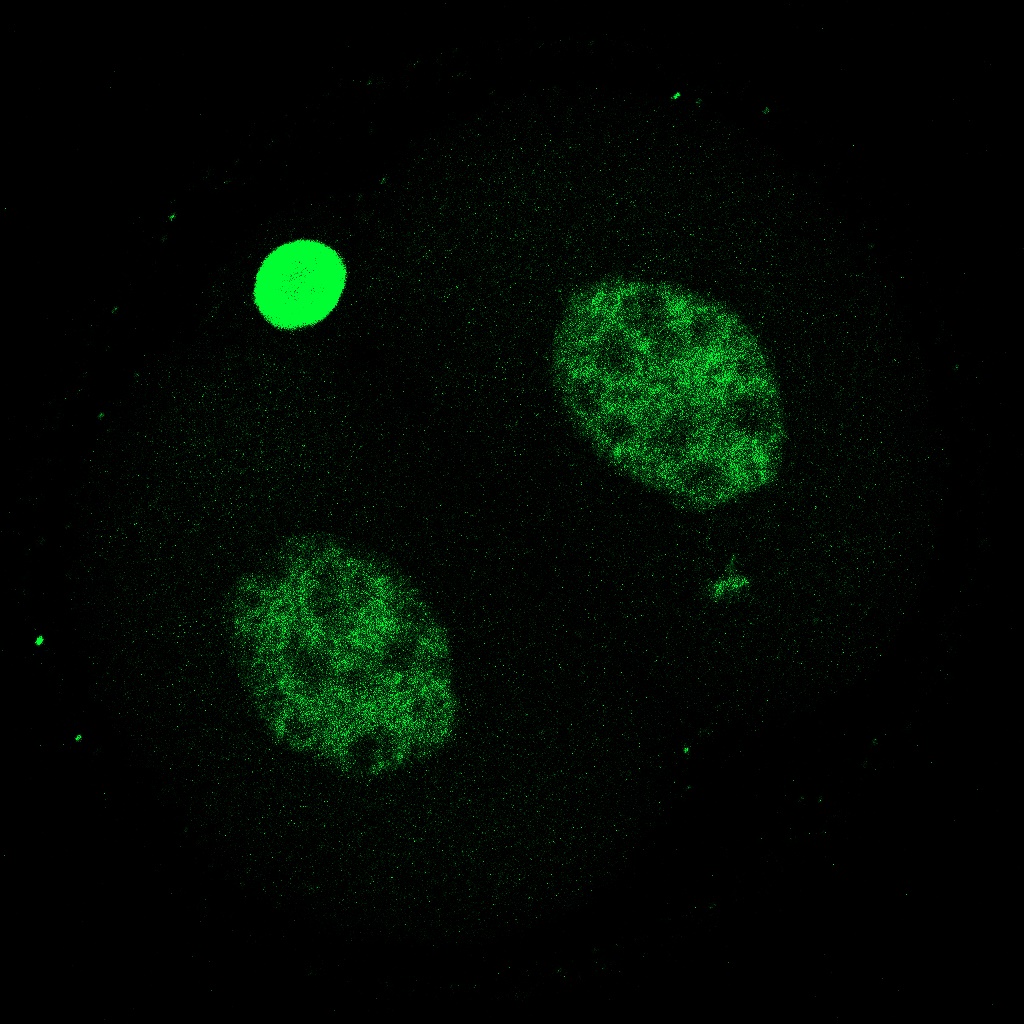

Supplement: Supplementary file 13 — Source data Fig. 3 [file 44318_2024_329_MOESM13_ESM.zip › SD figure 3/3E/Early2C_Kdm5b WT_H3K4me3.jpg]

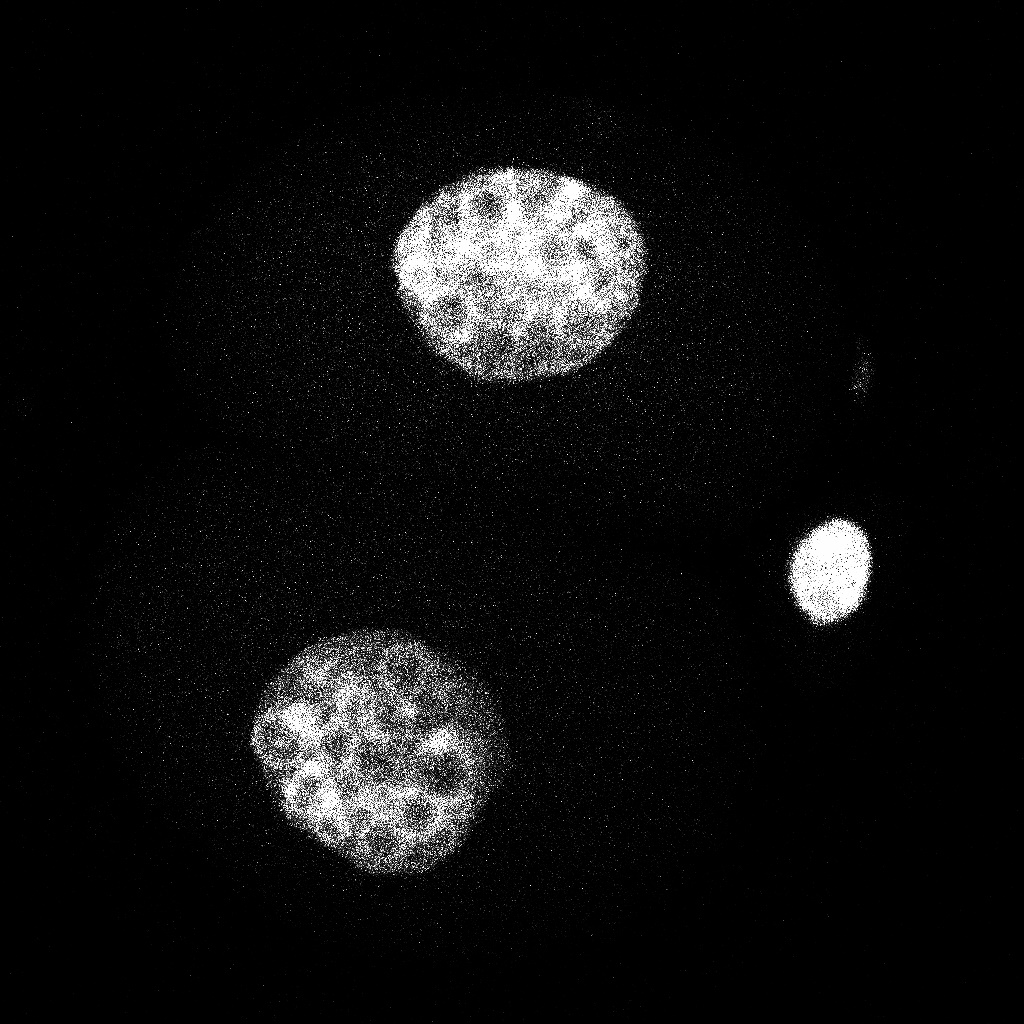

Supplement: Supplementary file 13 — Source data Fig. 3 [file 44318_2024_329_MOESM13_ESM.zip › SD figure 3/3G/Early2C_Control_DAPI.jpg]

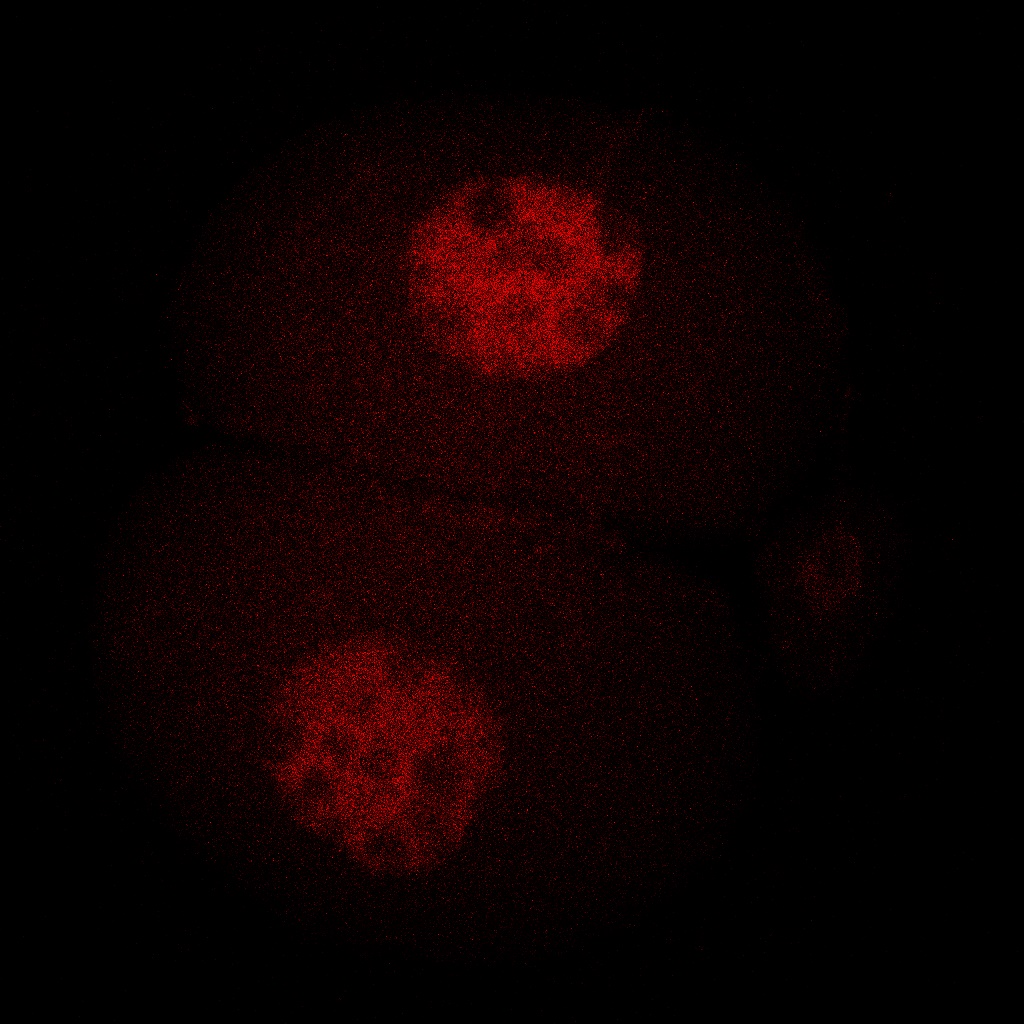

Supplement: Supplementary file 13 — Source data Fig. 3 [file 44318_2024_329_MOESM13_ESM.zip › SD figure 3/3G/Early2C_Control_EU.jpg]

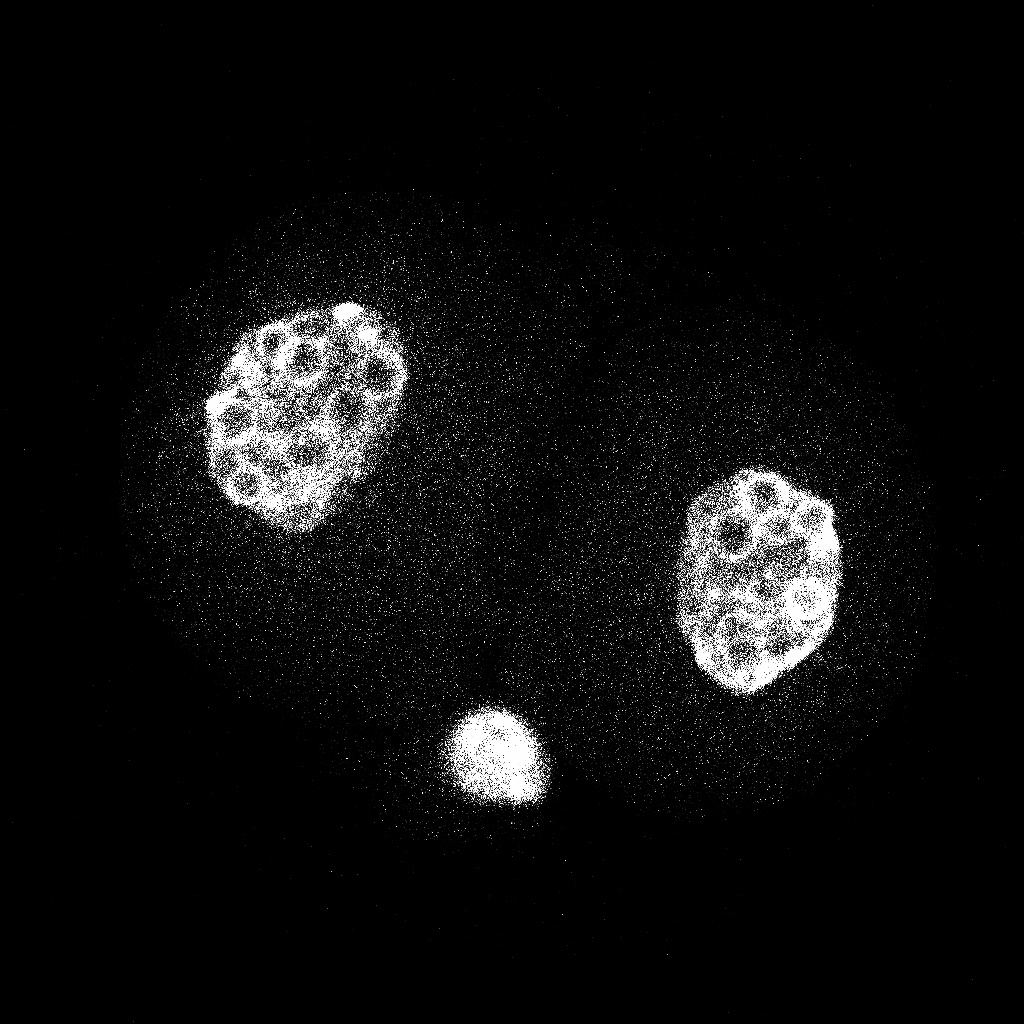

Supplement: Supplementary file 13 — Source data Fig. 3 [file 44318_2024_329_MOESM13_ESM.zip › SD figure 3/3G/Early2C_Kdm5b MUT_DAPI.jpg]

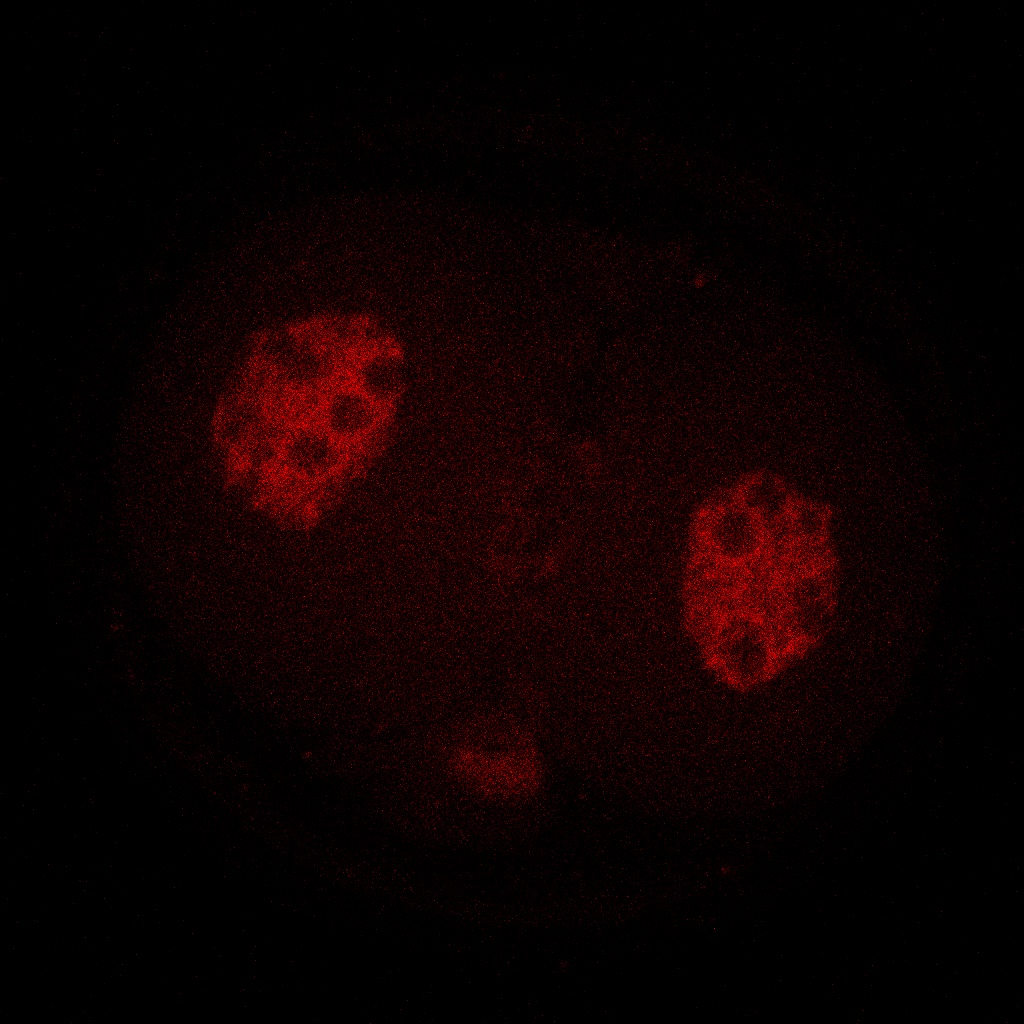

Supplement: Supplementary file 13 — Source data Fig. 3 [file 44318_2024_329_MOESM13_ESM.zip › SD figure 3/3G/Early2C_Kdm5b MUT_EU.jpg]

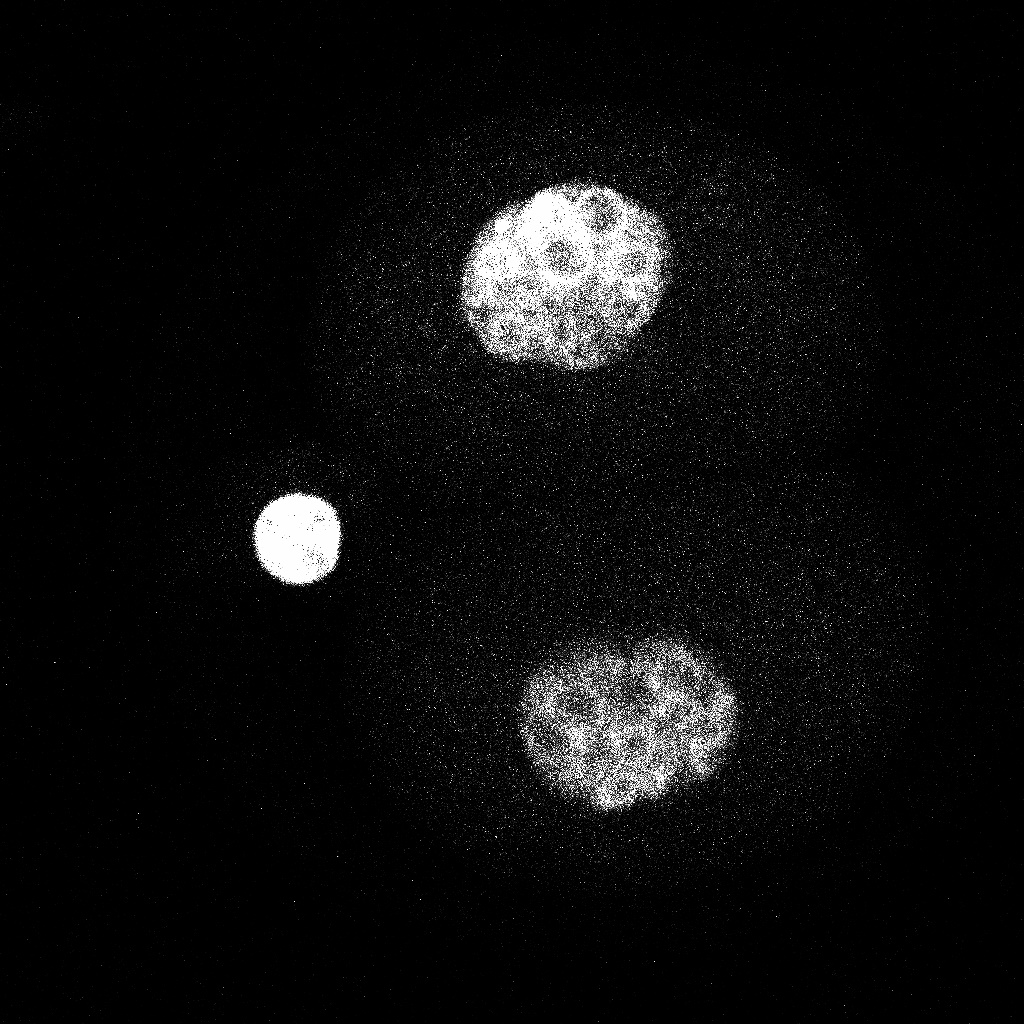

Supplement: Supplementary file 13 — Source data Fig. 3 [file 44318_2024_329_MOESM13_ESM.zip › SD figure 3/3G/Early2C_Kdm5b WT_DAPI.jpg]

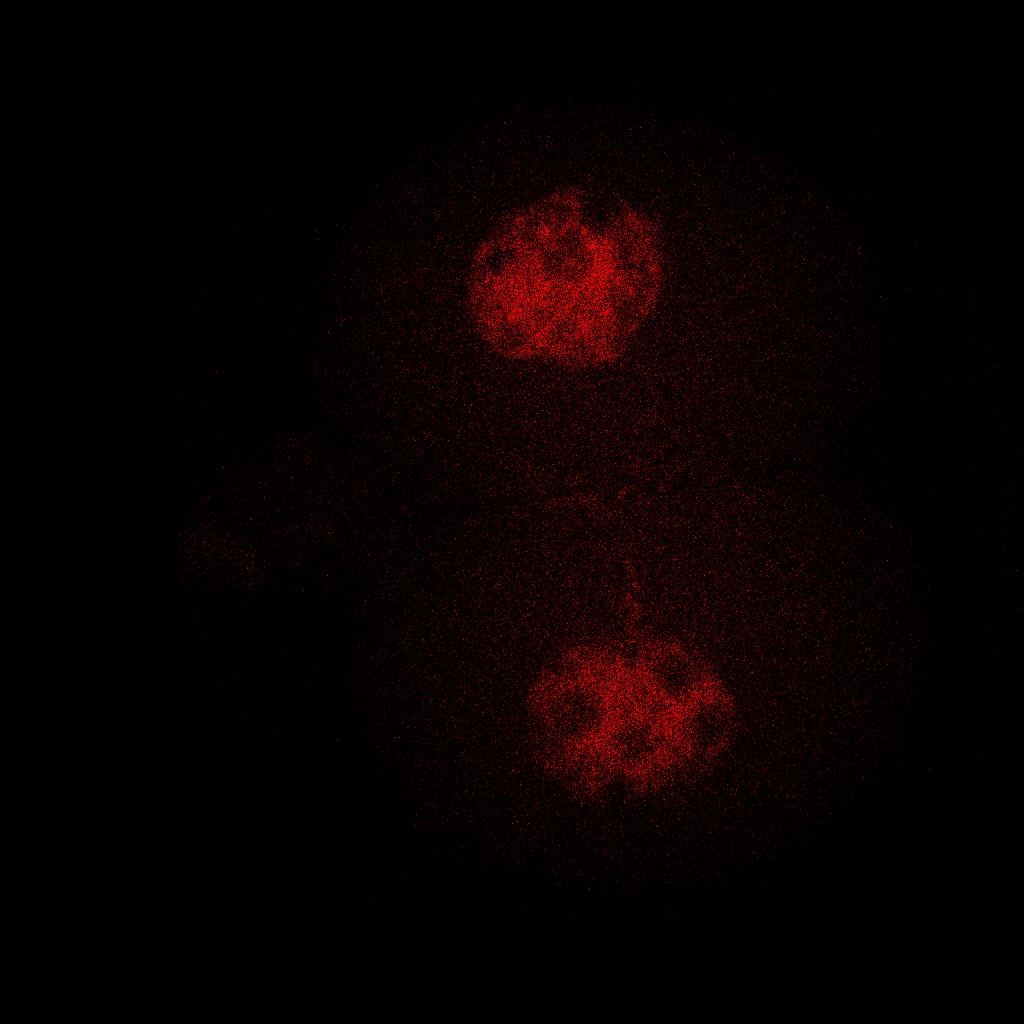

Supplement: Supplementary file 13 — Source data Fig. 3 [file 44318_2024_329_MOESM13_ESM.zip › SD figure 3/3G/Early2C_Kdm5b WT_EU.jpg]

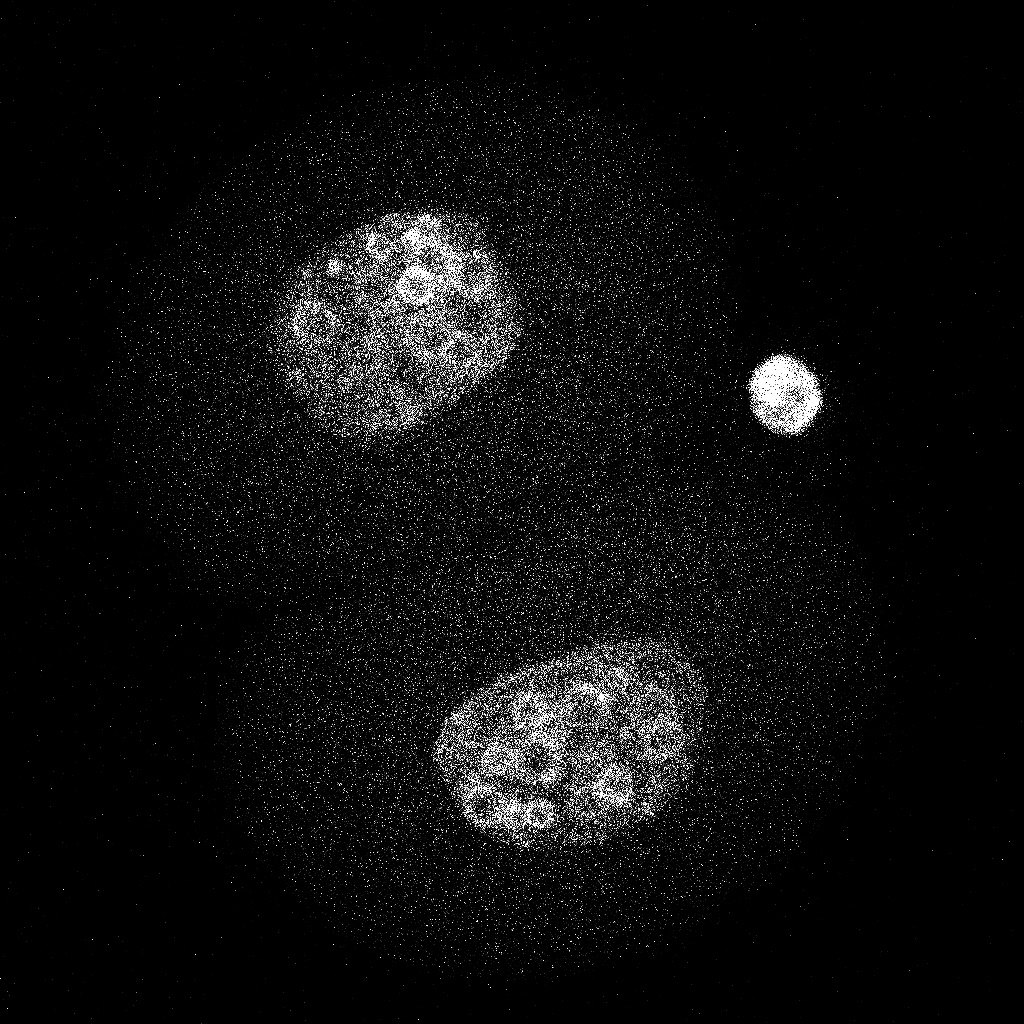

Supplement: Supplementary file 14 — Source data Fig. 4 [file 44318_2024_329_MOESM14_ESM.zip › SD figure 4/4B/Late2C_Control_DAPI.jpg]

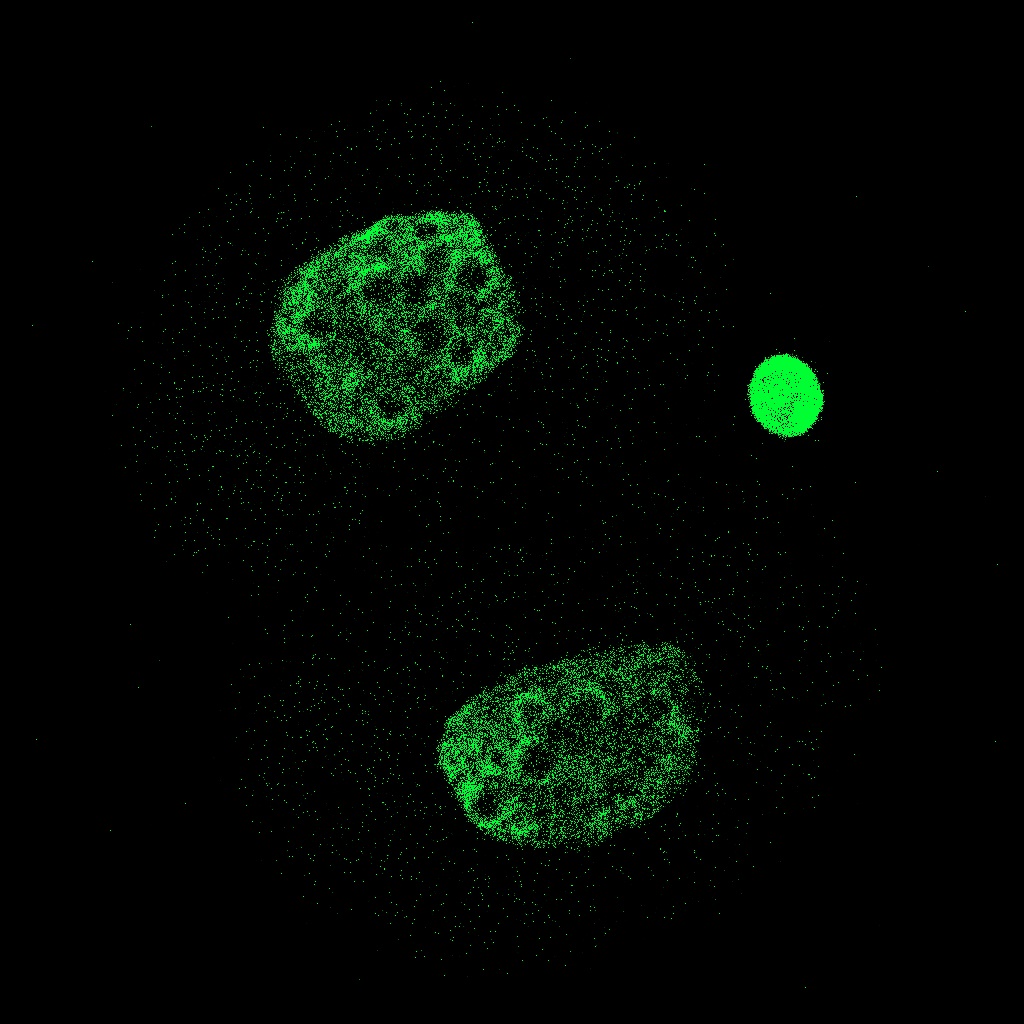

Supplement: Supplementary file 14 — Source data Fig. 4 [file 44318_2024_329_MOESM14_ESM.zip › SD figure 4/4B/Late2C_Control_H3K4me3.jpg]

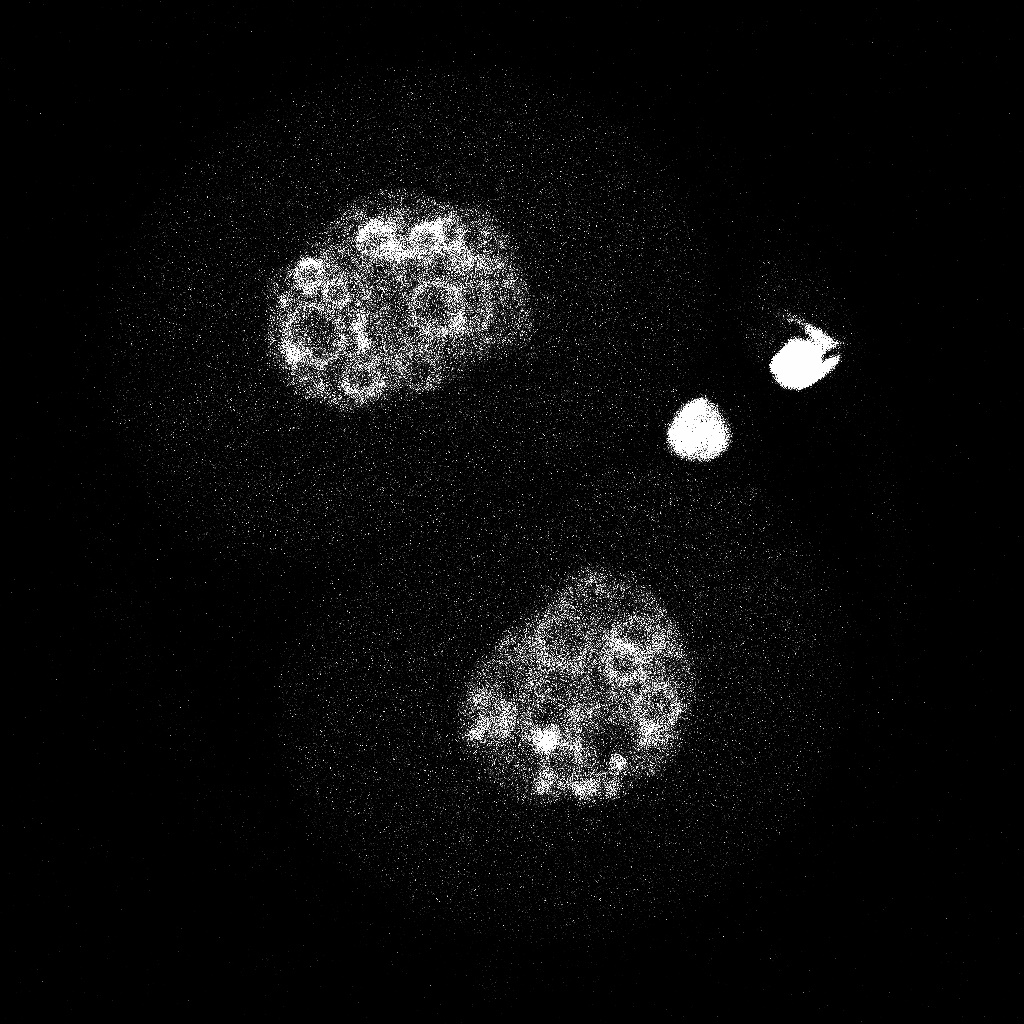

Supplement: Supplementary file 14 — Source data Fig. 4 [file 44318_2024_329_MOESM14_ESM.zip › SD figure 4/4B/Late2C_CPI-455_DAPI.jpg]

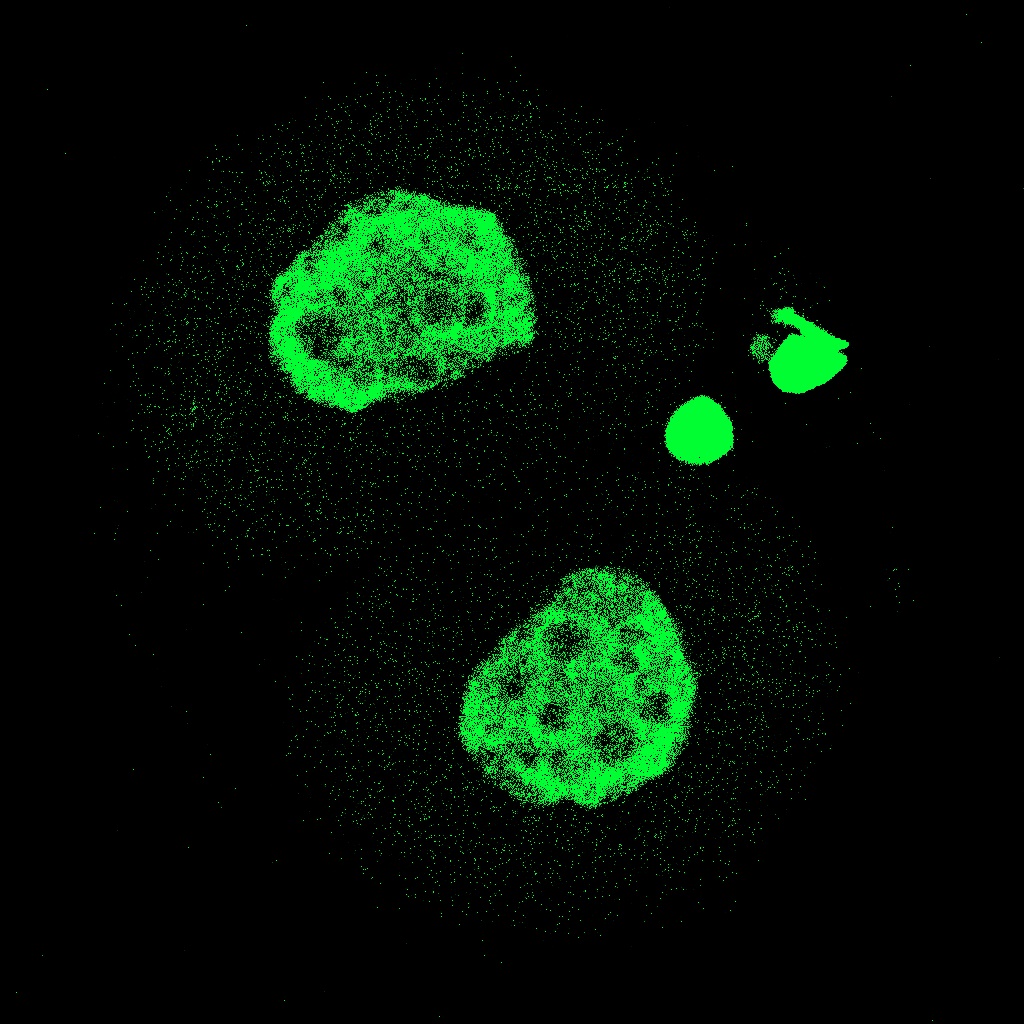

Supplement: Supplementary file 14 — Source data Fig. 4 [file 44318_2024_329_MOESM14_ESM.zip › SD figure 4/4B/Late2C_CPI-455_H3K4me3.jpg]

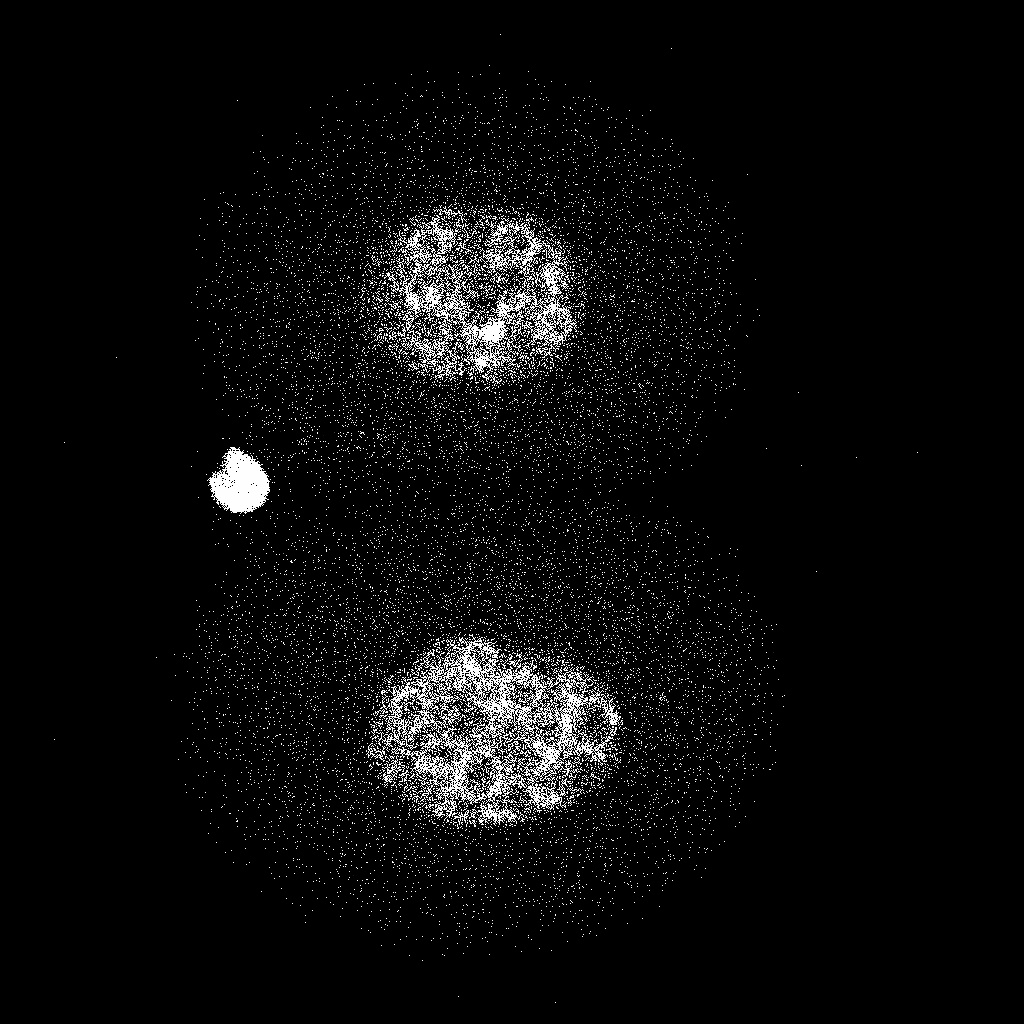

Supplement: Supplementary file 14 — Source data Fig. 4 [file 44318_2024_329_MOESM14_ESM.zip › SD figure 4/4B/Late2C_DMSO_DAPI.jpg]

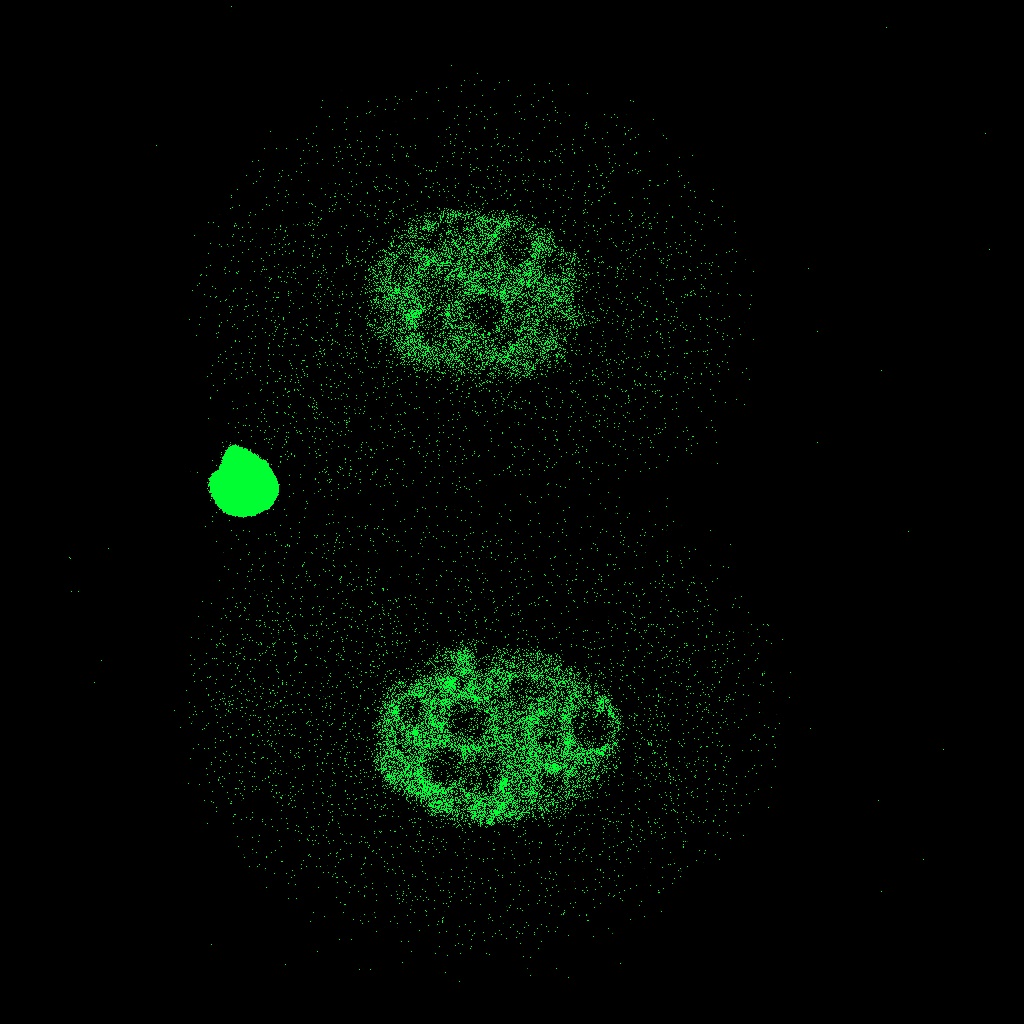

Supplement: Supplementary file 14 — Source data Fig. 4 [file 44318_2024_329_MOESM14_ESM.zip › SD figure 4/4B/Late2C_DMSO_H3K4me3.jpg]

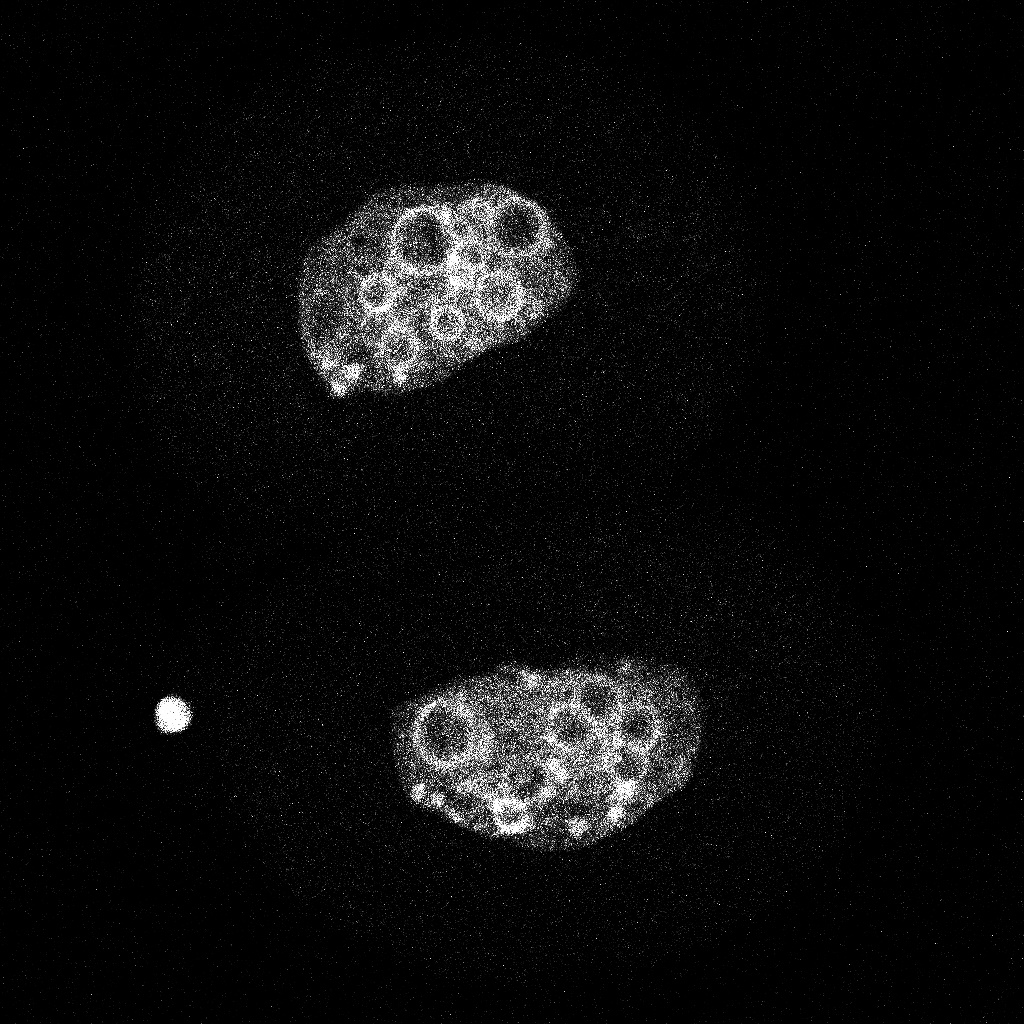

Supplement: Supplementary file 14 — Source data Fig. 4 [file 44318_2024_329_MOESM14_ESM.zip › SD figure 4/4B/Late2C_Kdm5b KD_DAPI.jpg]

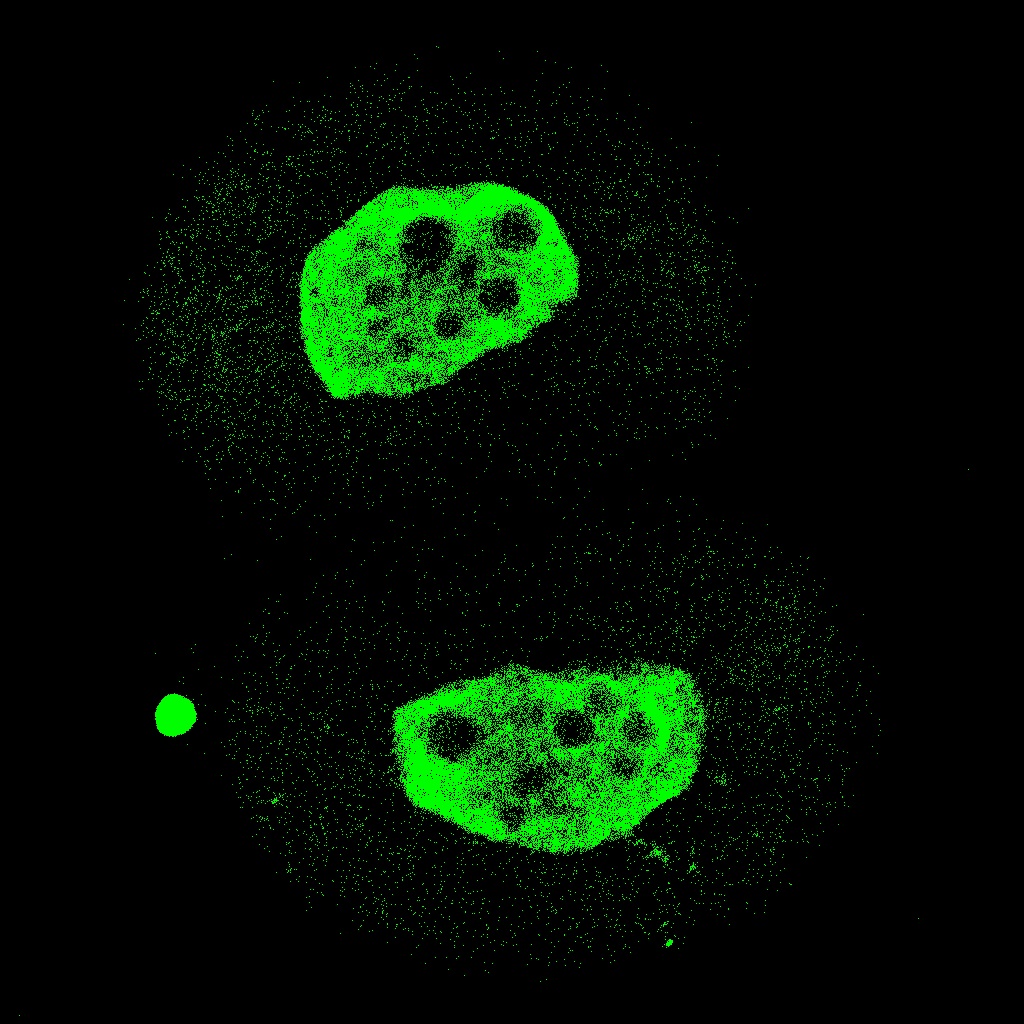

Supplement: Supplementary file 14 — Source data Fig. 4 [file 44318_2024_329_MOESM14_ESM.zip › SD figure 4/4B/Late2C_Kdm5b KD_H3K4me3.jpg]

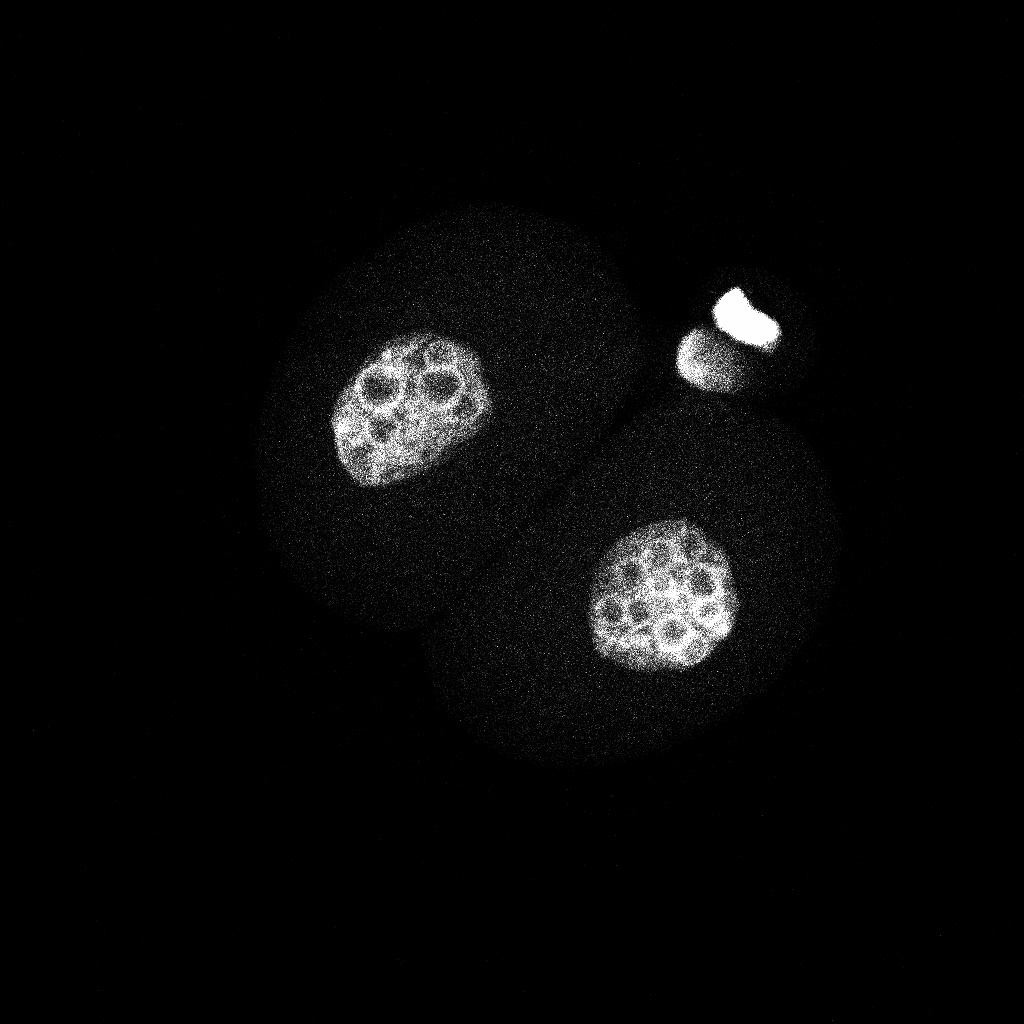

Supplement: Supplementary file 14 — Source data Fig. 4 [file 44318_2024_329_MOESM14_ESM.zip › SD figure 4/4E/Late2C_Control_DAPI.jpg]

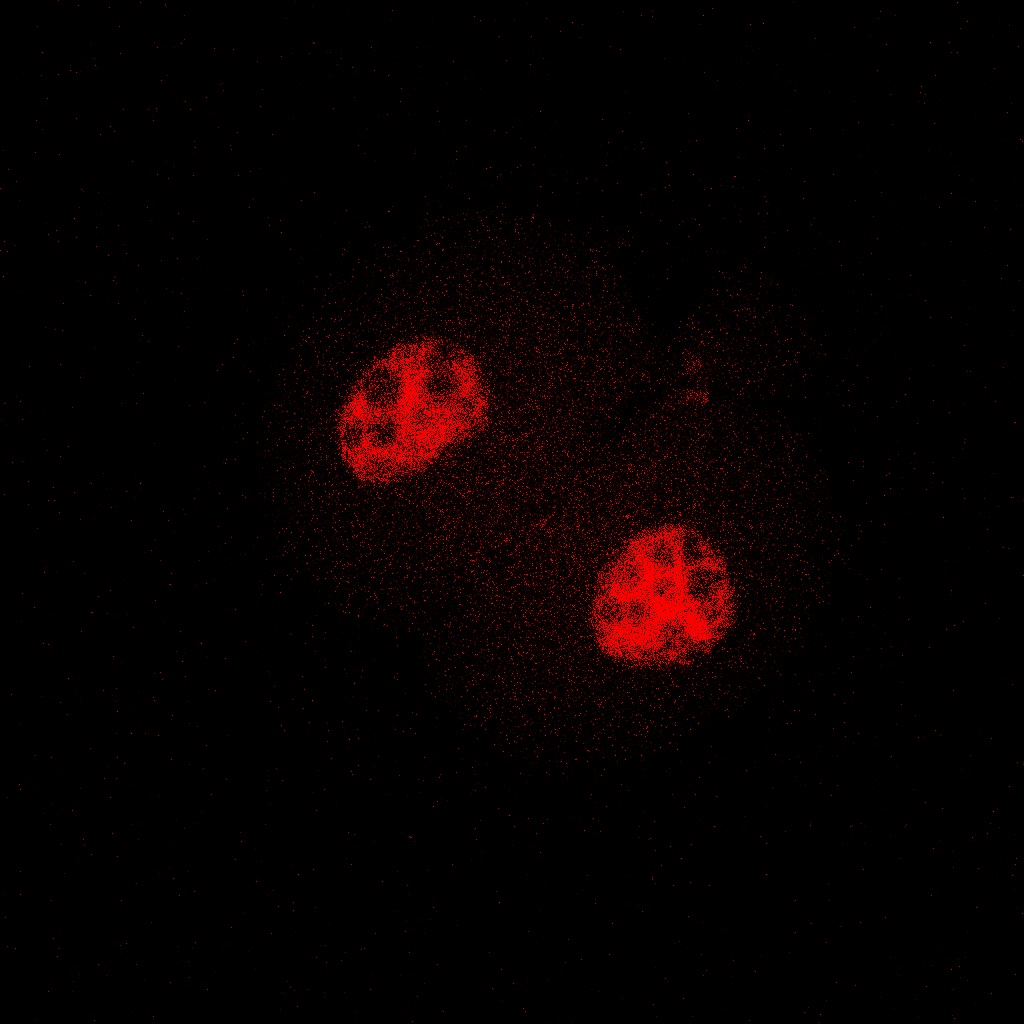

Supplement: Supplementary file 14 — Source data Fig. 4 [file 44318_2024_329_MOESM14_ESM.zip › SD figure 4/4E/Late2C_Control_EU.jpg]

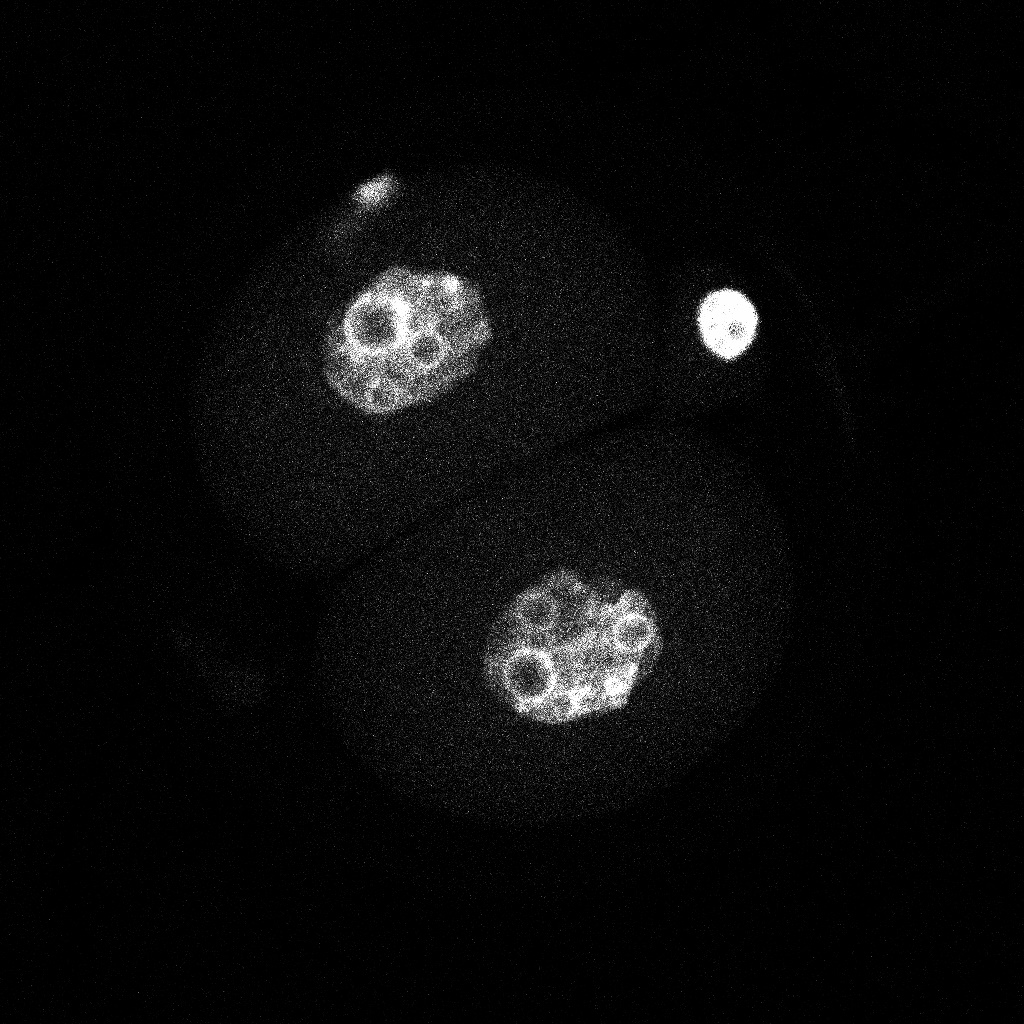

Supplement: Supplementary file 14 — Source data Fig. 4 [file 44318_2024_329_MOESM14_ESM.zip › SD figure 4/4E/Late2C_CPI-455_DAPI.jpg]

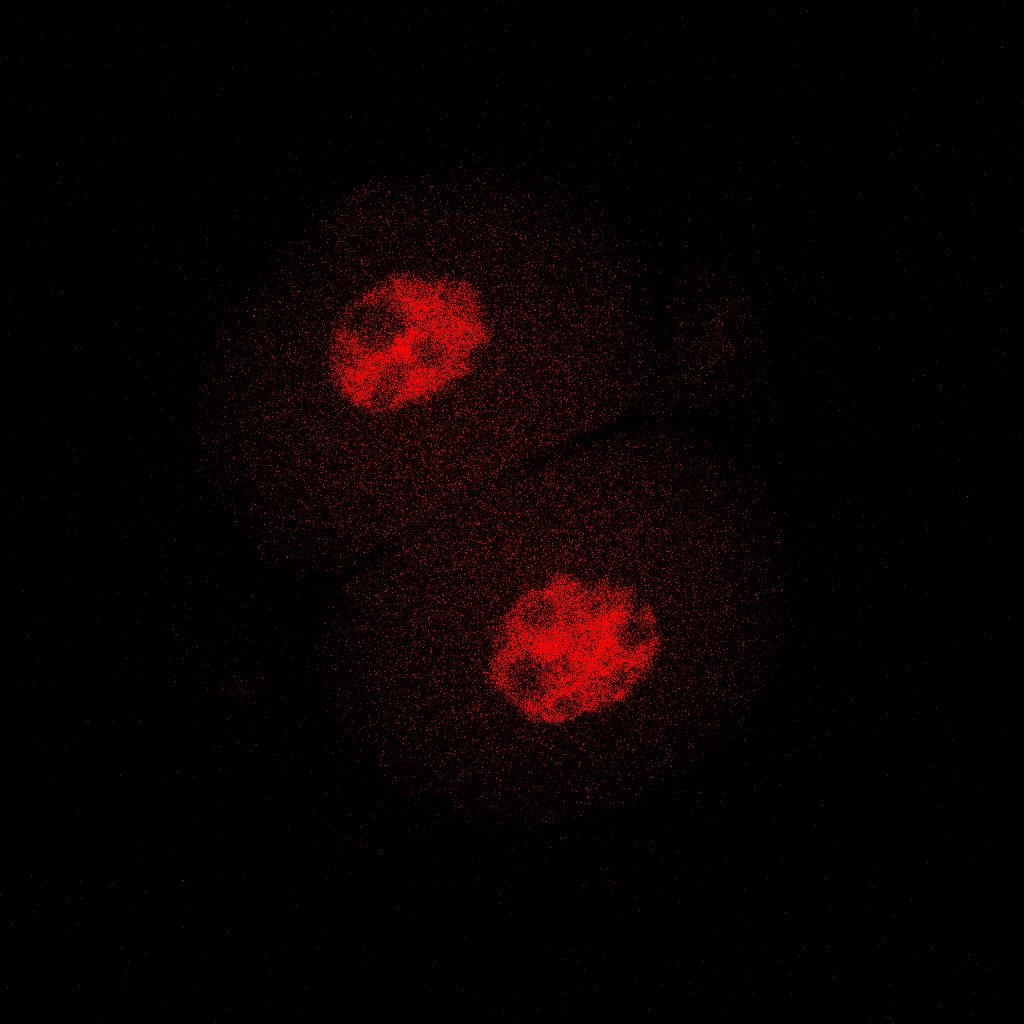

Supplement: Supplementary file 14 — Source data Fig. 4 [file 44318_2024_329_MOESM14_ESM.zip › SD figure 4/4E/Late2C_CPI-455_EU.jpg]

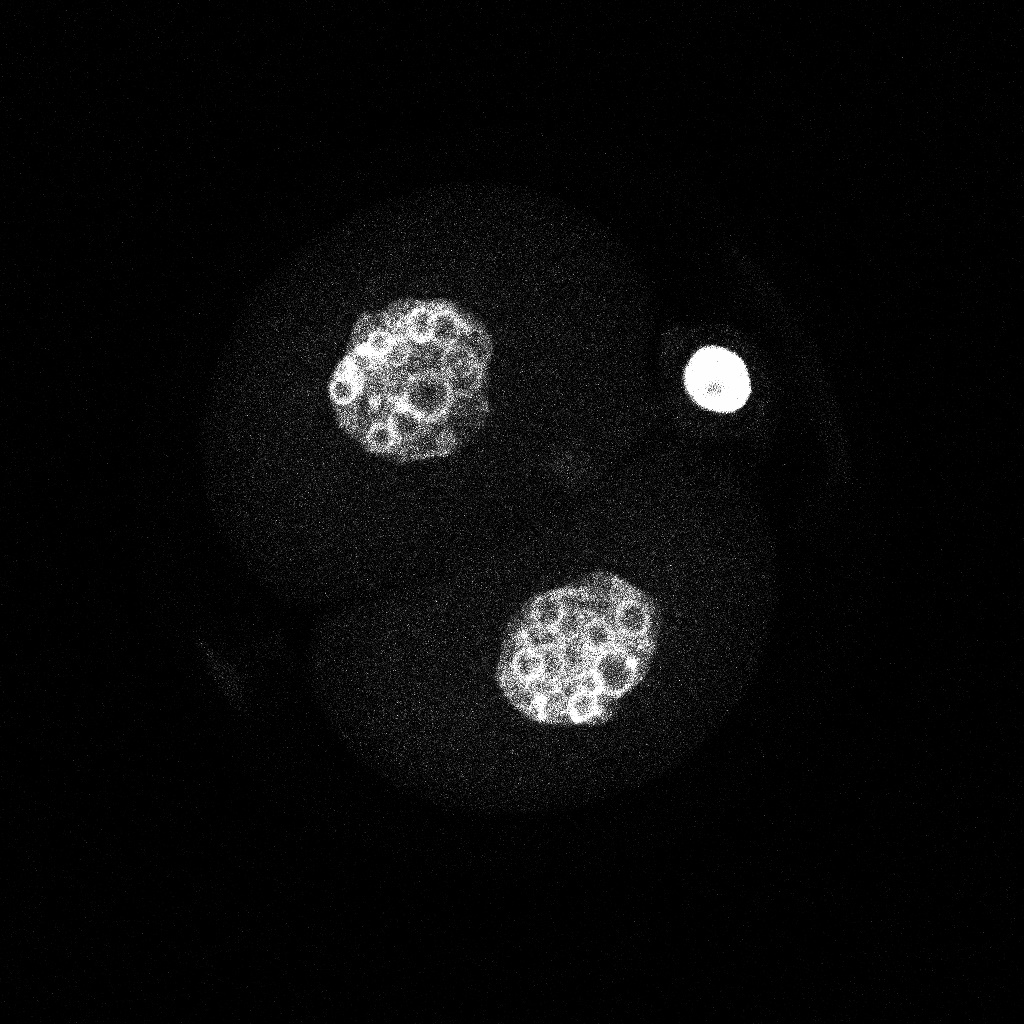

Supplement: Supplementary file 14 — Source data Fig. 4 [file 44318_2024_329_MOESM14_ESM.zip › SD figure 4/4E/Late2C_DMSO_DAPI.jpg]

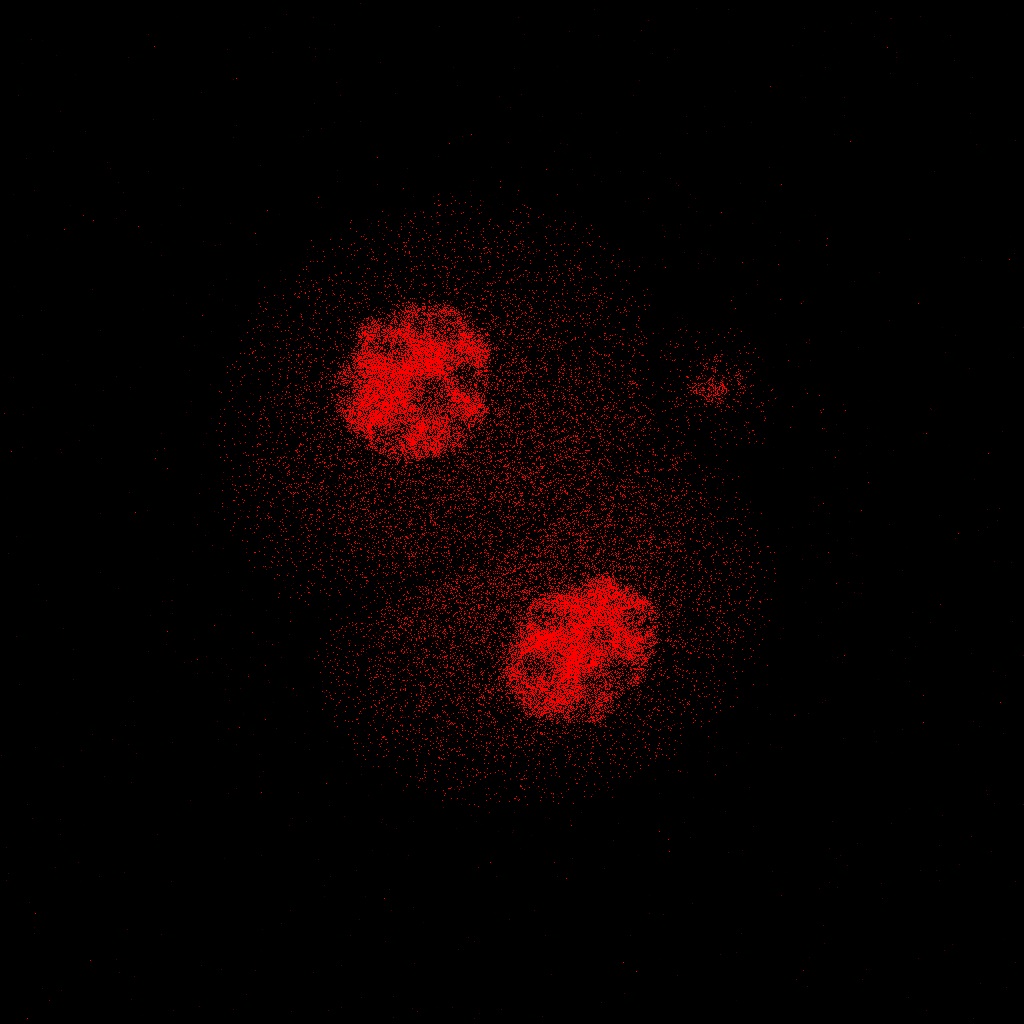

Supplement: Supplementary file 14 — Source data Fig. 4 [file 44318_2024_329_MOESM14_ESM.zip › SD figure 4/4E/Late2C_DMSO_EU.jpg]

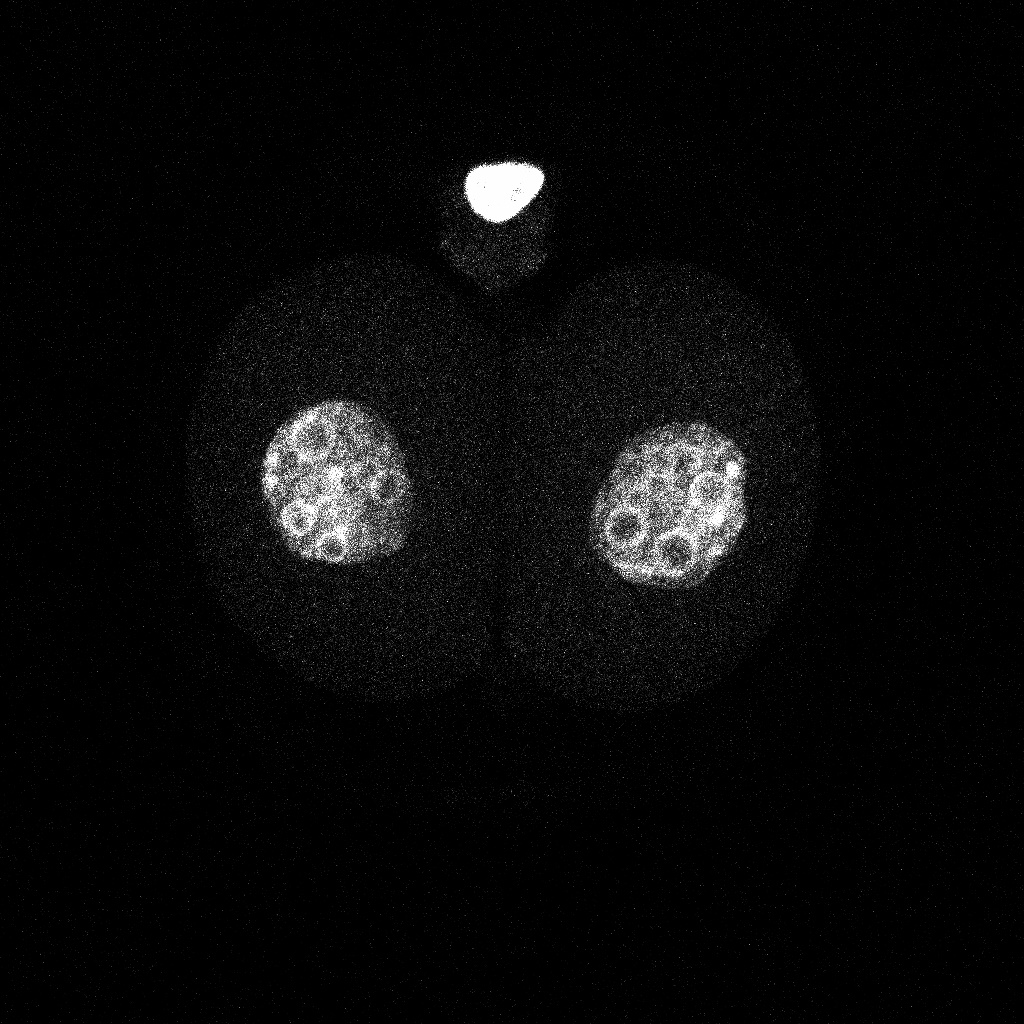

Supplement: Supplementary file 14 — Source data Fig. 4 [file 44318_2024_329_MOESM14_ESM.zip › SD figure 4/4E/Late2C_Kdm5b KD_DAPI.jpg]

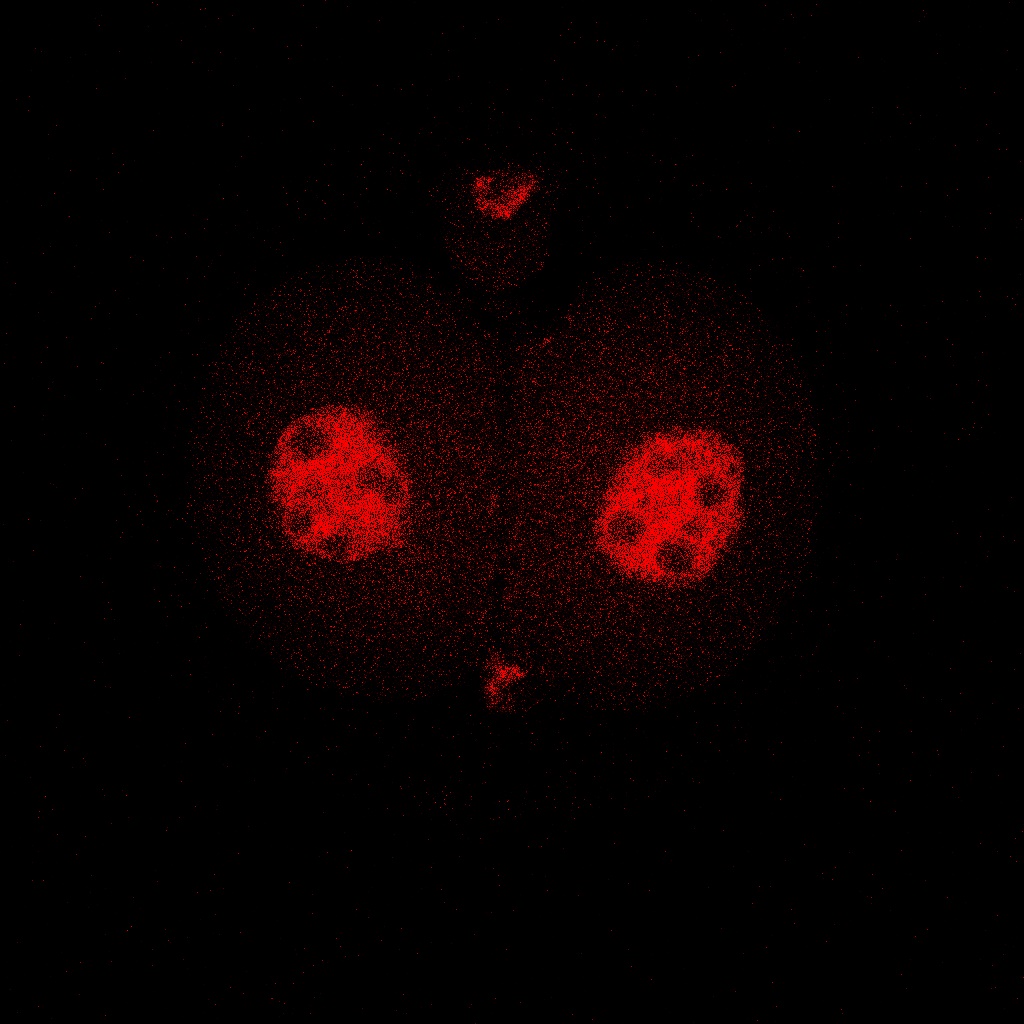

Supplement: Supplementary file 14 — Source data Fig. 4 [file 44318_2024_329_MOESM14_ESM.zip › SD figure 4/4E/Late2C_Kdm5b KD_EU.jpg]

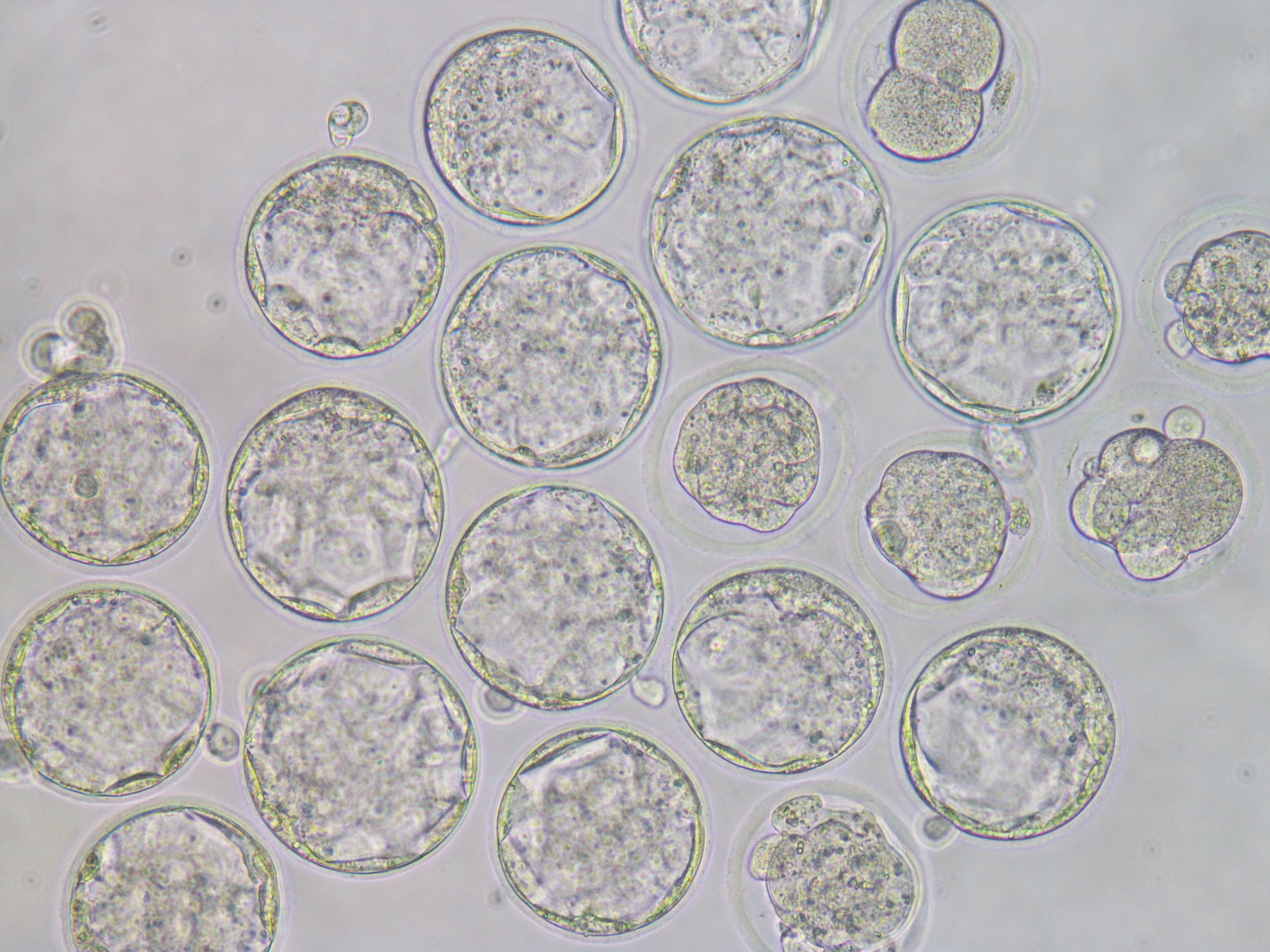

Supplement: Supplementary file 15 — Source data Fig. 5 [file 44318_2024_329_MOESM15_ESM.zip › SD figure 5/5B/Control.tif]

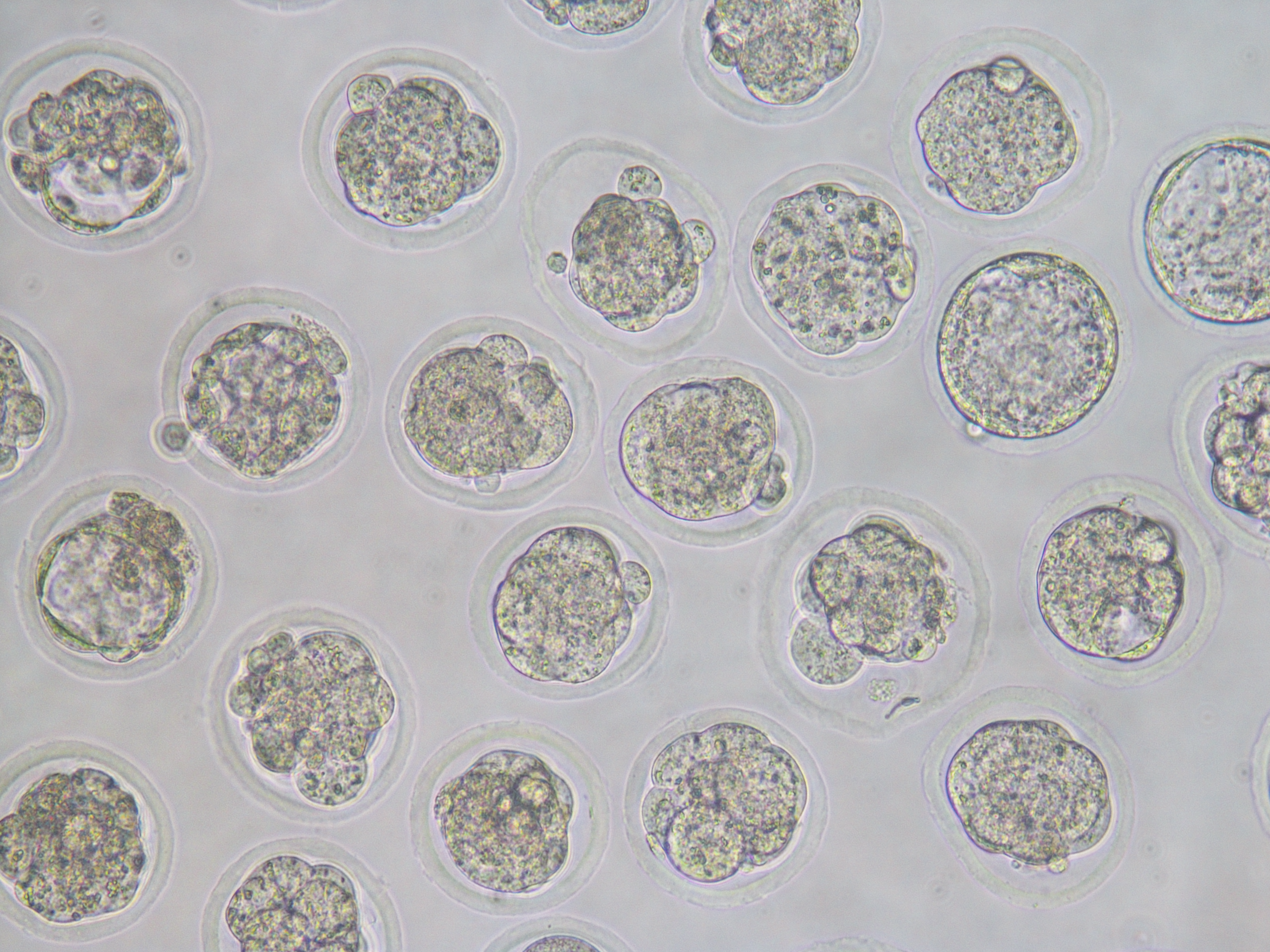

Supplement: Supplementary file 15 — Source data Fig. 5 [file 44318_2024_329_MOESM15_ESM.zip › SD figure 5/5B/Setd1ab KD.tif]

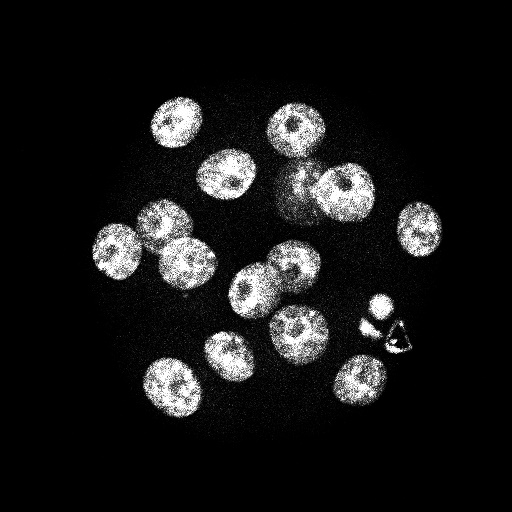

Supplement: Supplementary file 15 — Source data Fig. 5 [file 44318_2024_329_MOESM15_ESM.zip › SD figure 5/5C/Control_DAPI.jpg]

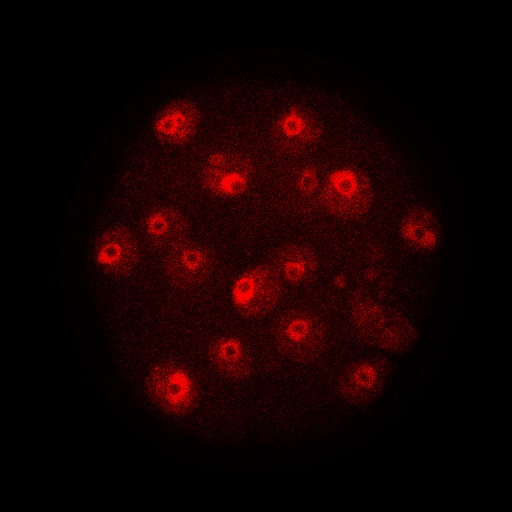

Supplement: Supplementary file 15 — Source data Fig. 5 [file 44318_2024_329_MOESM15_ESM.zip › SD figure 5/5C/Control_EU.jpg]

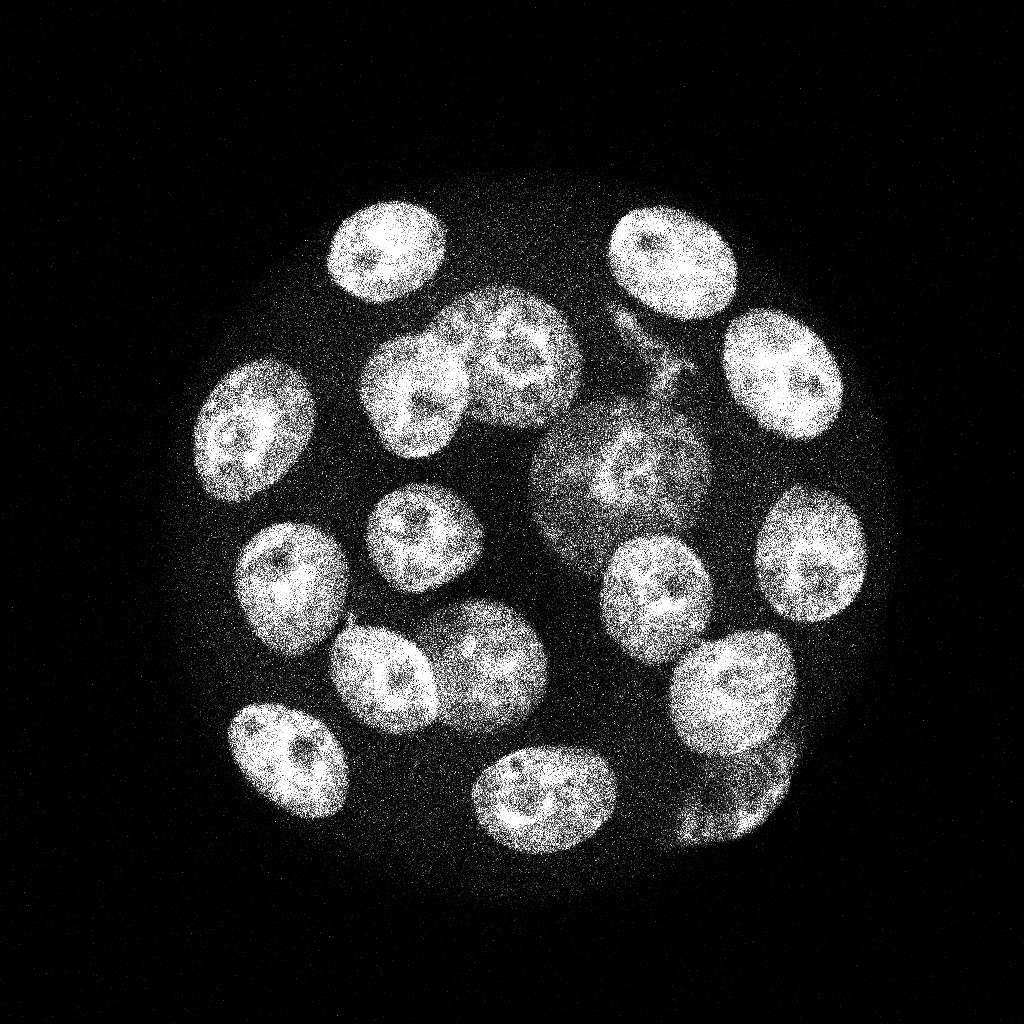

Supplement: Supplementary file 15 — Source data Fig. 5 [file 44318_2024_329_MOESM15_ESM.zip › SD figure 5/5C/Setd1ab KD_DAPI.jpg]

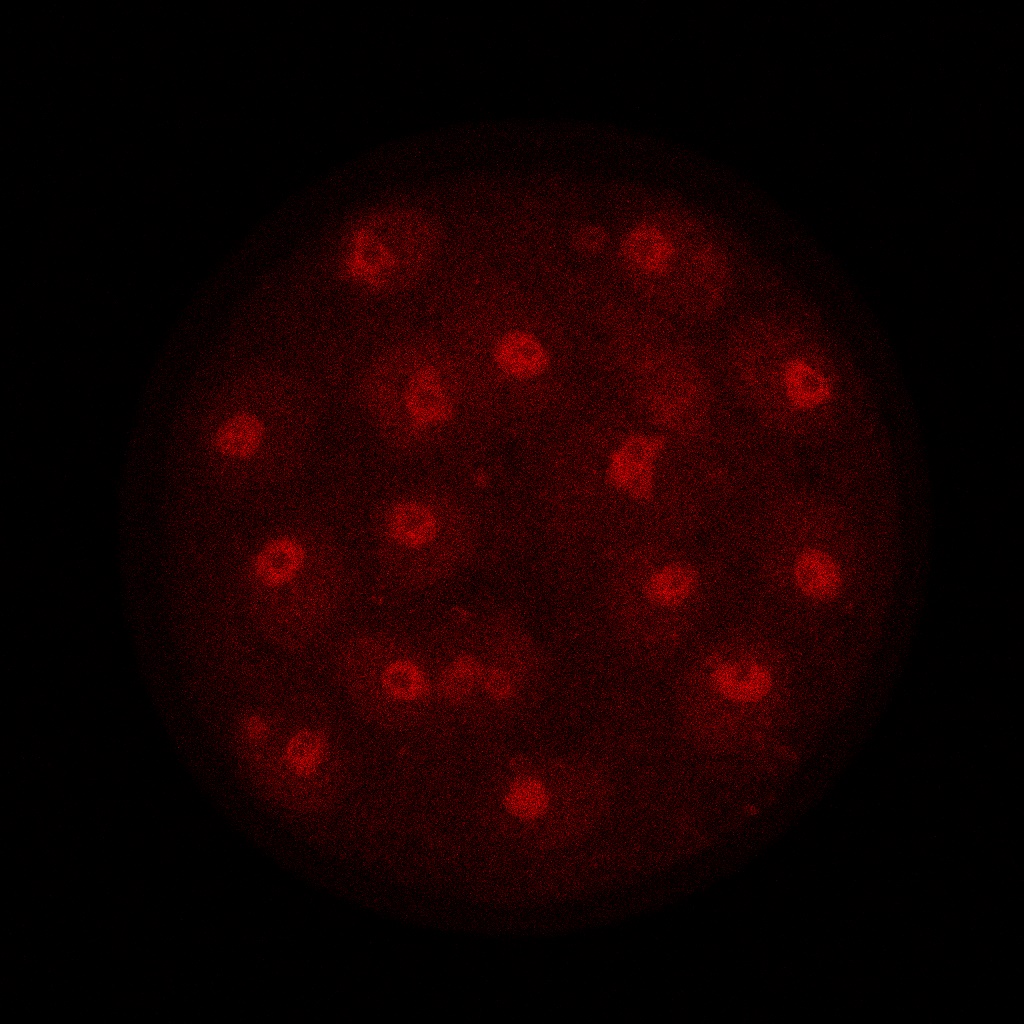

Supplement: Supplementary file 15 — Source data Fig. 5 [file 44318_2024_329_MOESM15_ESM.zip › SD figure 5/5C/Setd1ab KD_EU.jpg]

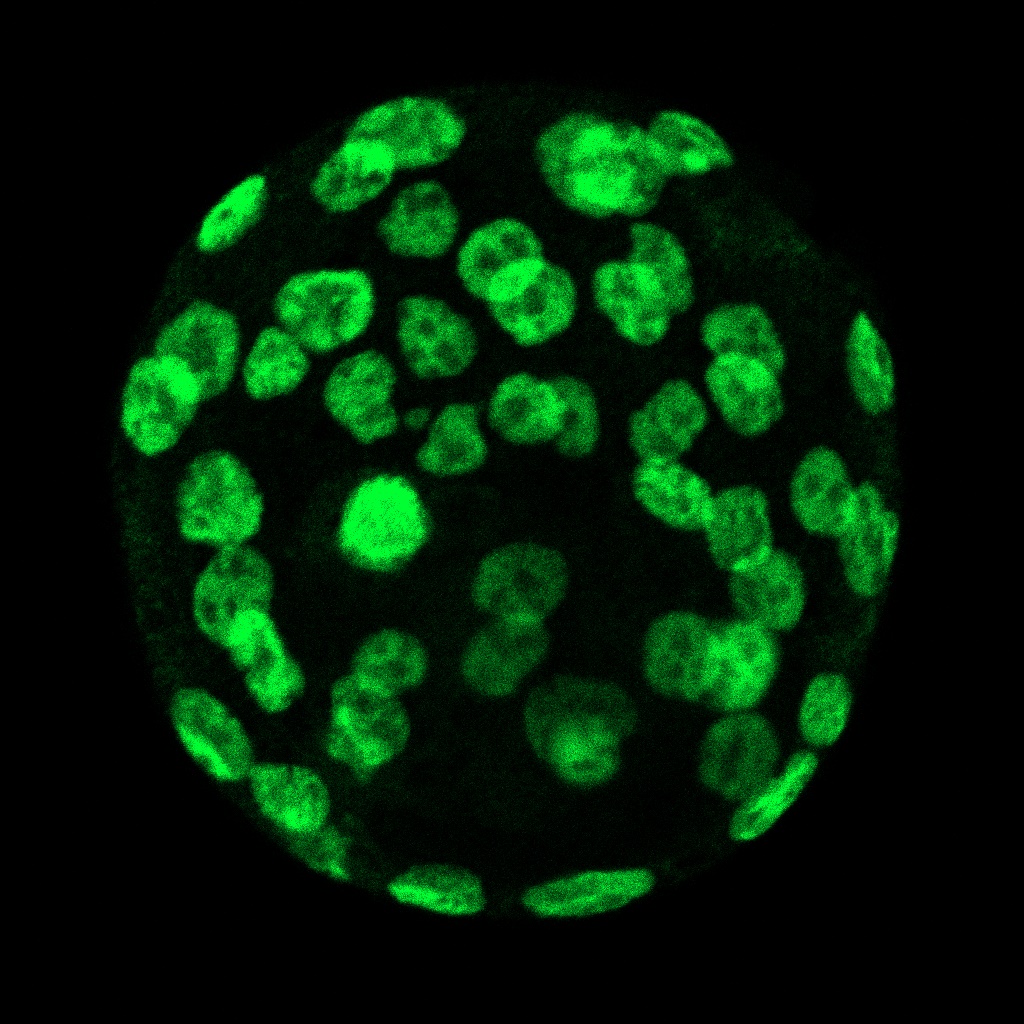

Supplement: Supplementary file 15 — Source data Fig. 5 [file 44318_2024_329_MOESM15_ESM.zip › SD figure 5/5F/Blastocyst_Control_CDX2.jpg]

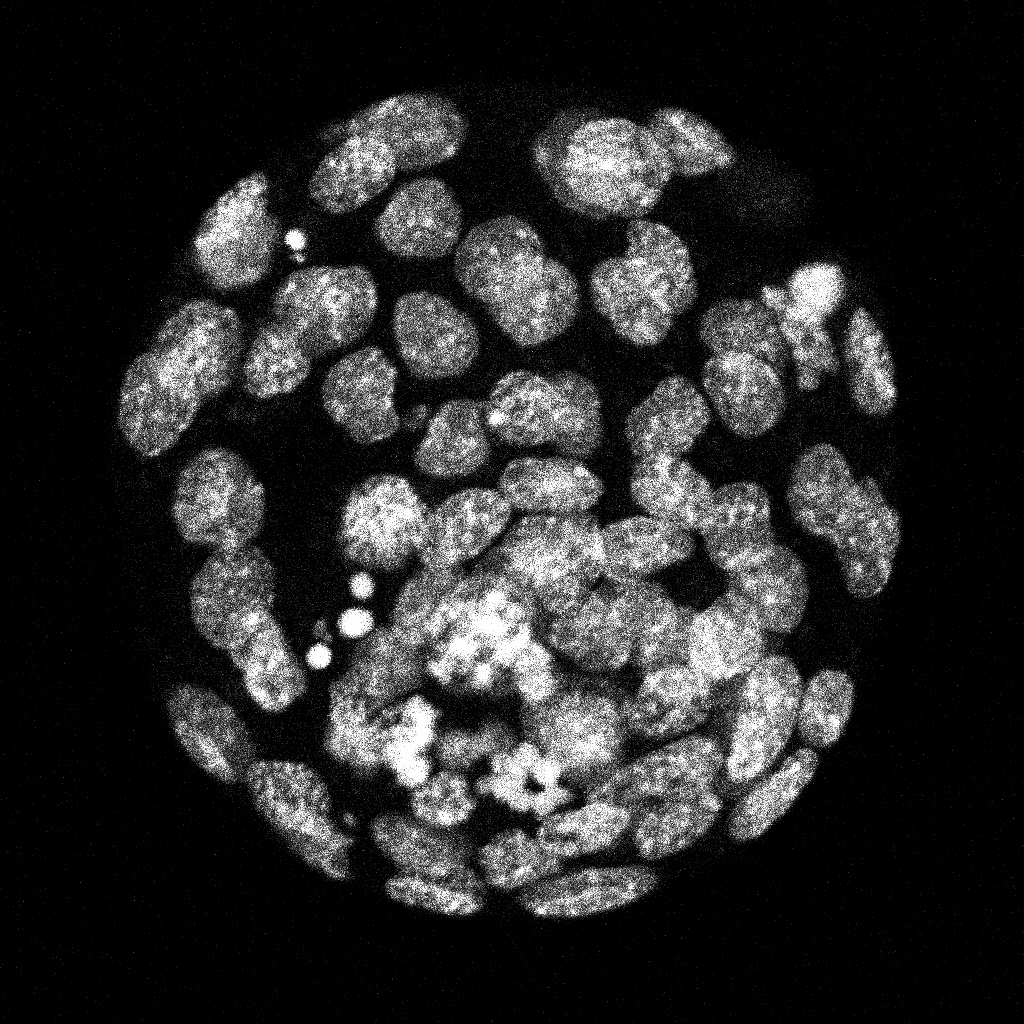

Supplement: Supplementary file 15 — Source data Fig. 5 [file 44318_2024_329_MOESM15_ESM.zip › SD figure 5/5F/Blastocyst_Control_DAPI_CDX2.jpg]

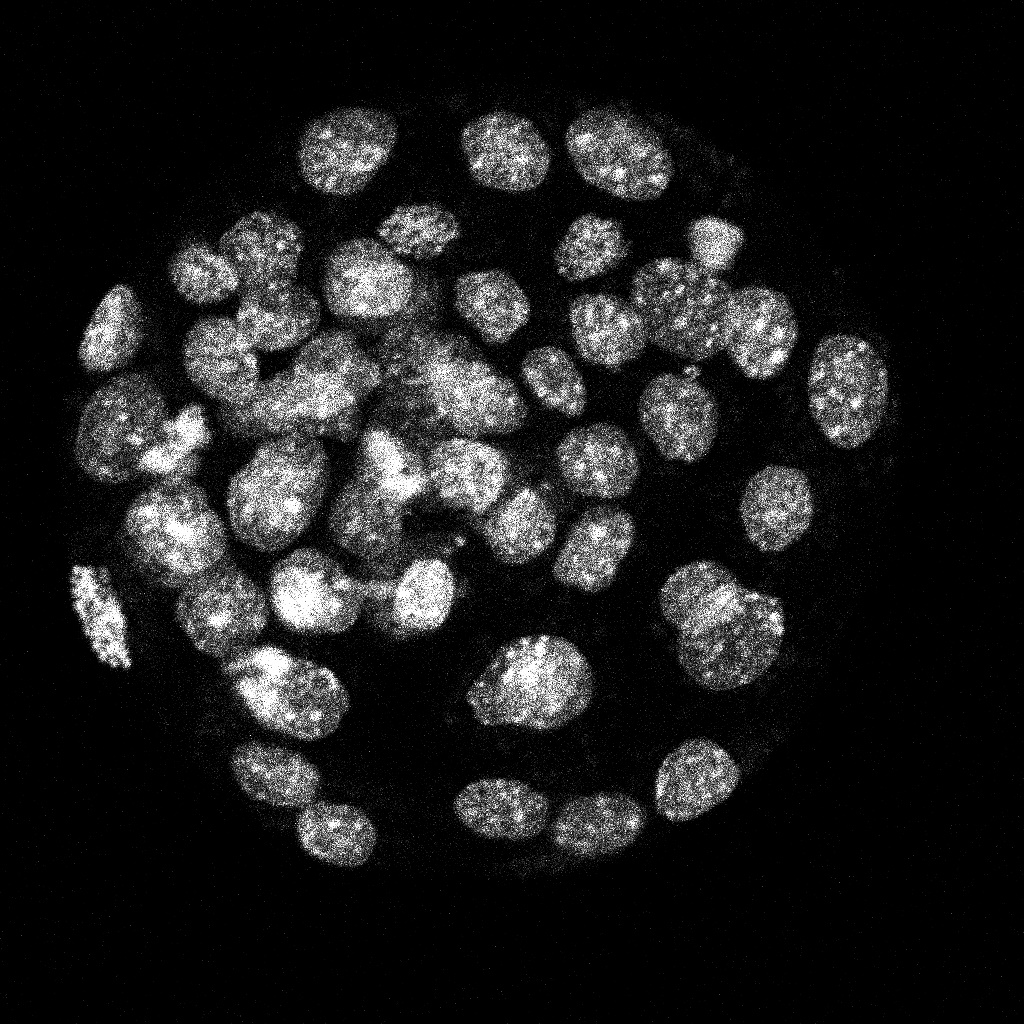

Supplement: Supplementary file 15 — Source data Fig. 5 [file 44318_2024_329_MOESM15_ESM.zip › SD figure 5/5F/Blastocyst_Control_DAPI_NANOG.jpg]

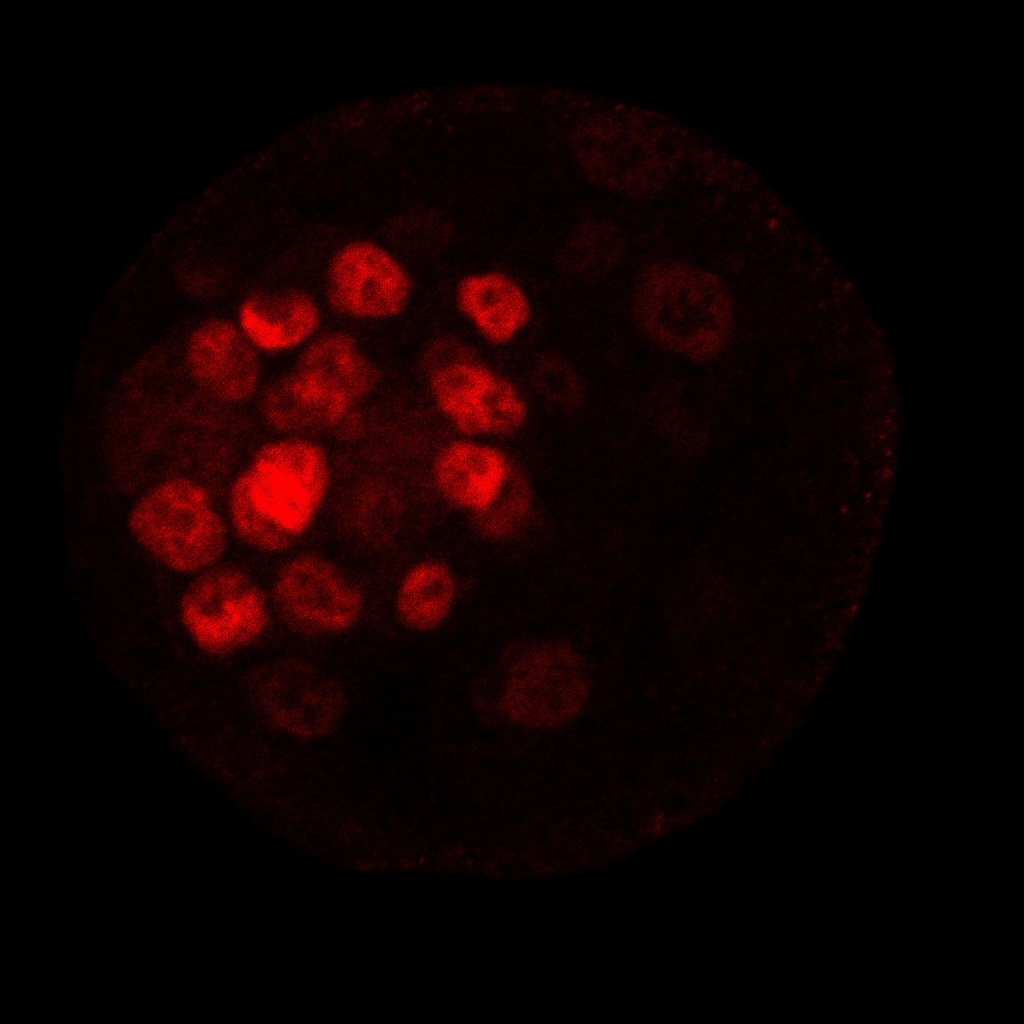

Supplement: Supplementary file 15 — Source data Fig. 5 [file 44318_2024_329_MOESM15_ESM.zip › SD figure 5/5F/Blastocyst_Control_NANOG.jpg]

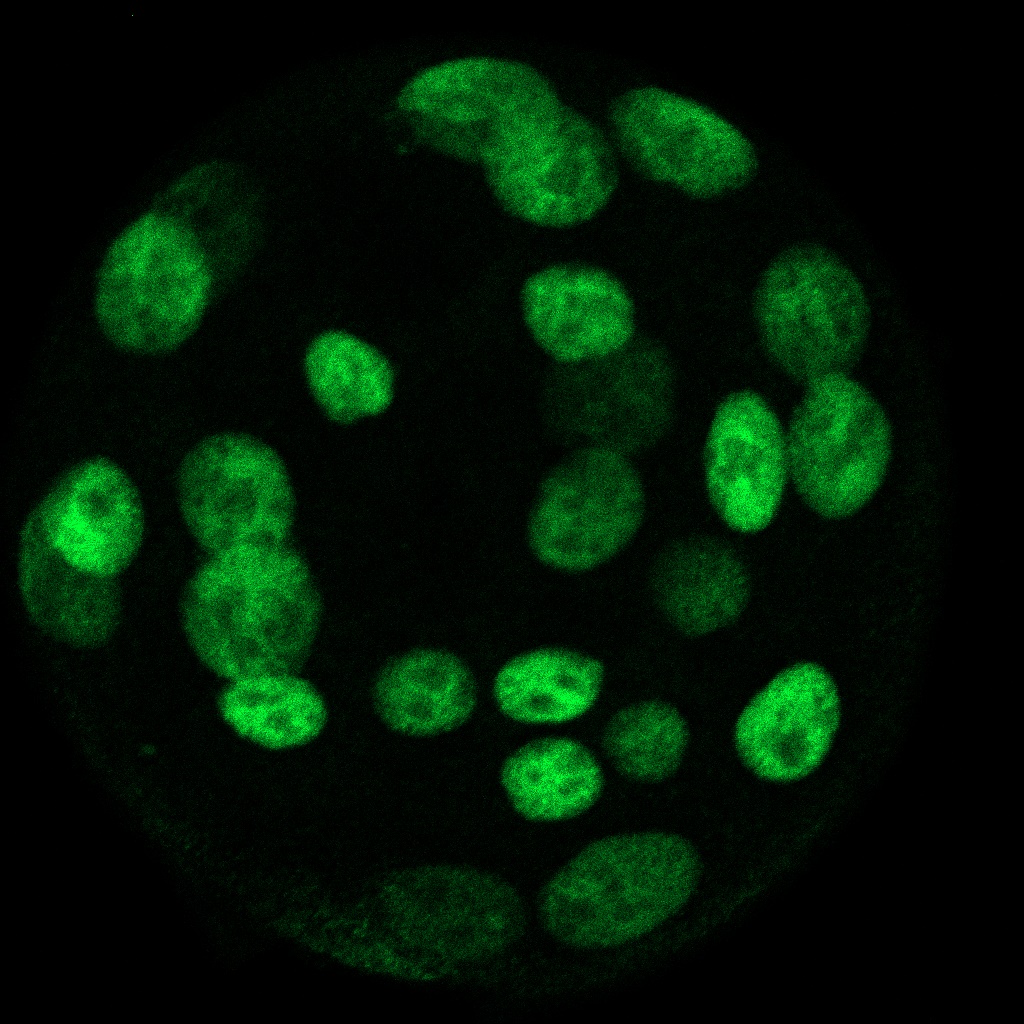

Supplement: Supplementary file 15 — Source data Fig. 5 [file 44318_2024_329_MOESM15_ESM.zip › SD figure 5/5F/Blastocyst_Setd1ab CDX2.jpg]

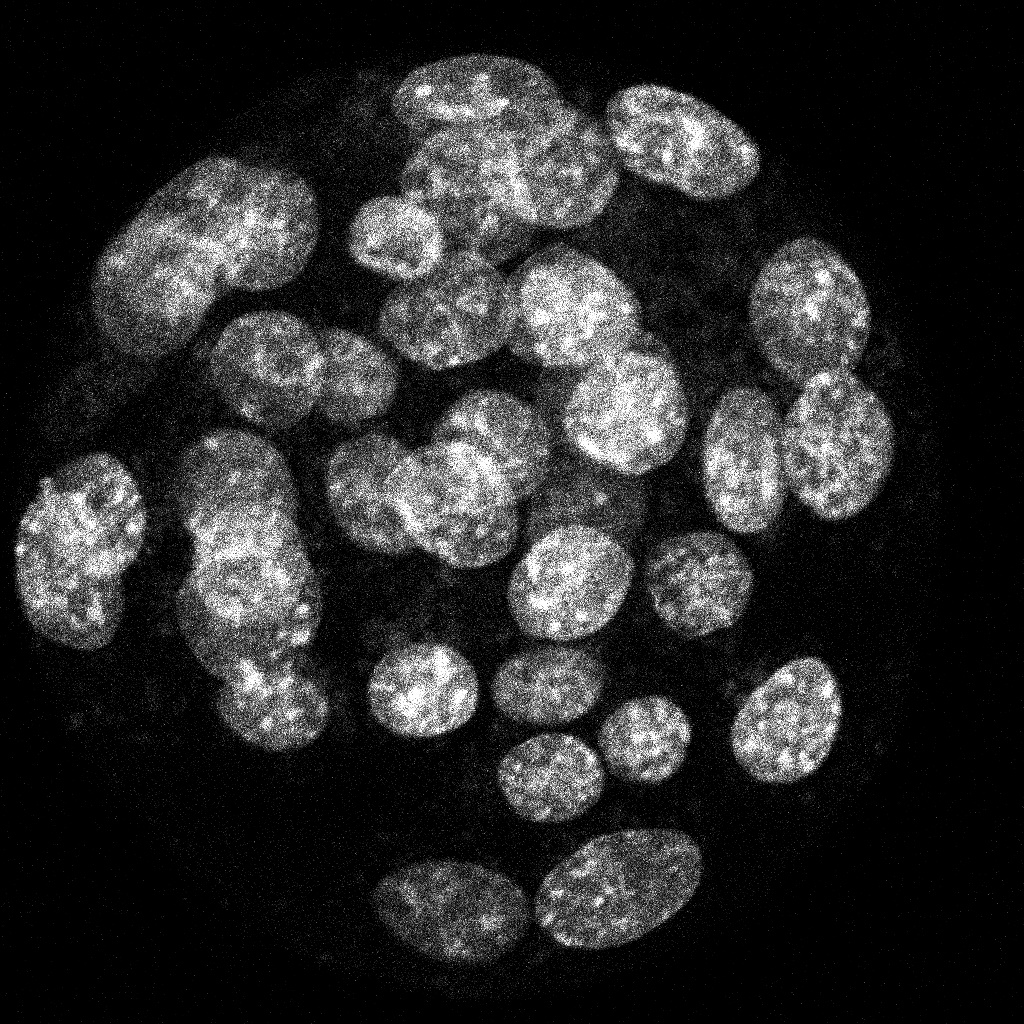

Supplement: Supplementary file 15 — Source data Fig. 5 [file 44318_2024_329_MOESM15_ESM.zip › SD figure 5/5F/Blastocyst_Setd1ab KD_DAPI_CDX2.jpg]

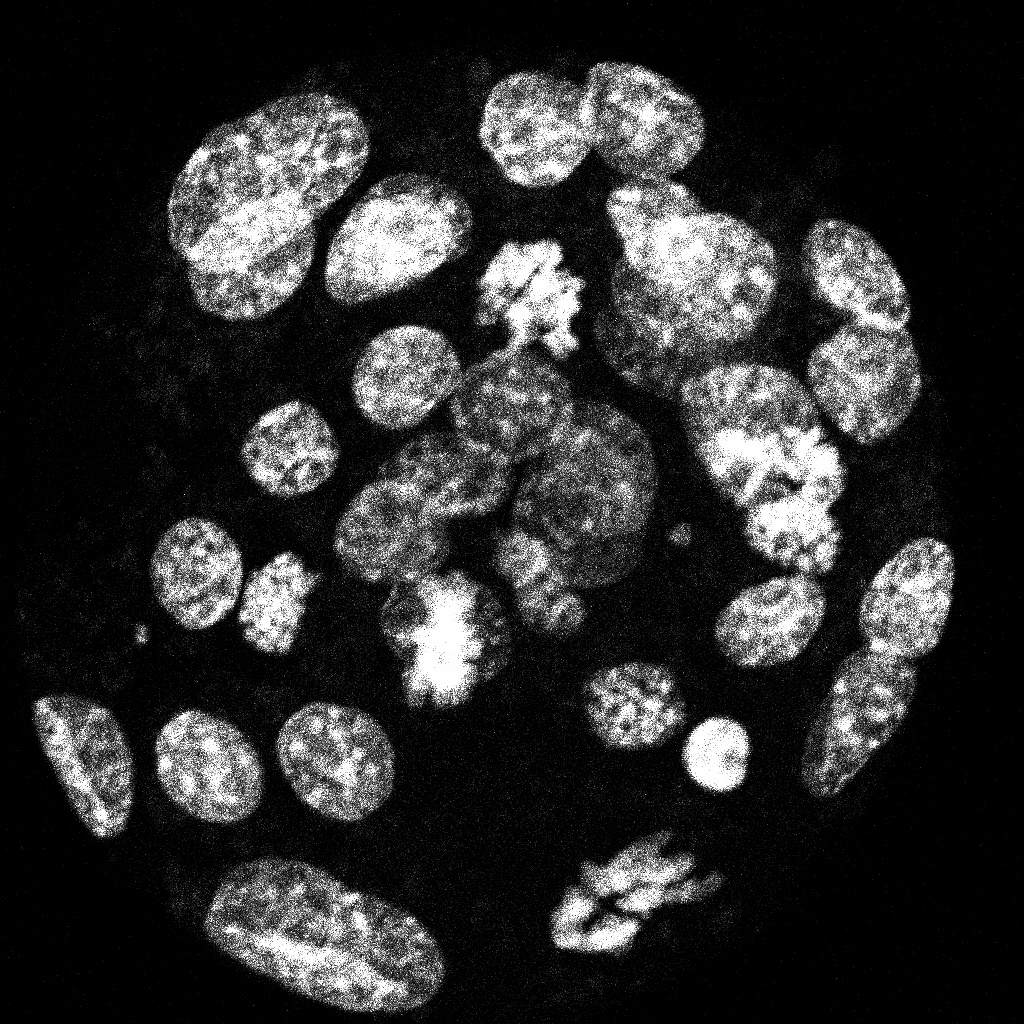

Supplement: Supplementary file 15 — Source data Fig. 5 [file 44318_2024_329_MOESM15_ESM.zip › SD figure 5/5F/Blastocyst_Setd1ab KD_DAPI_NANOG.jpg]

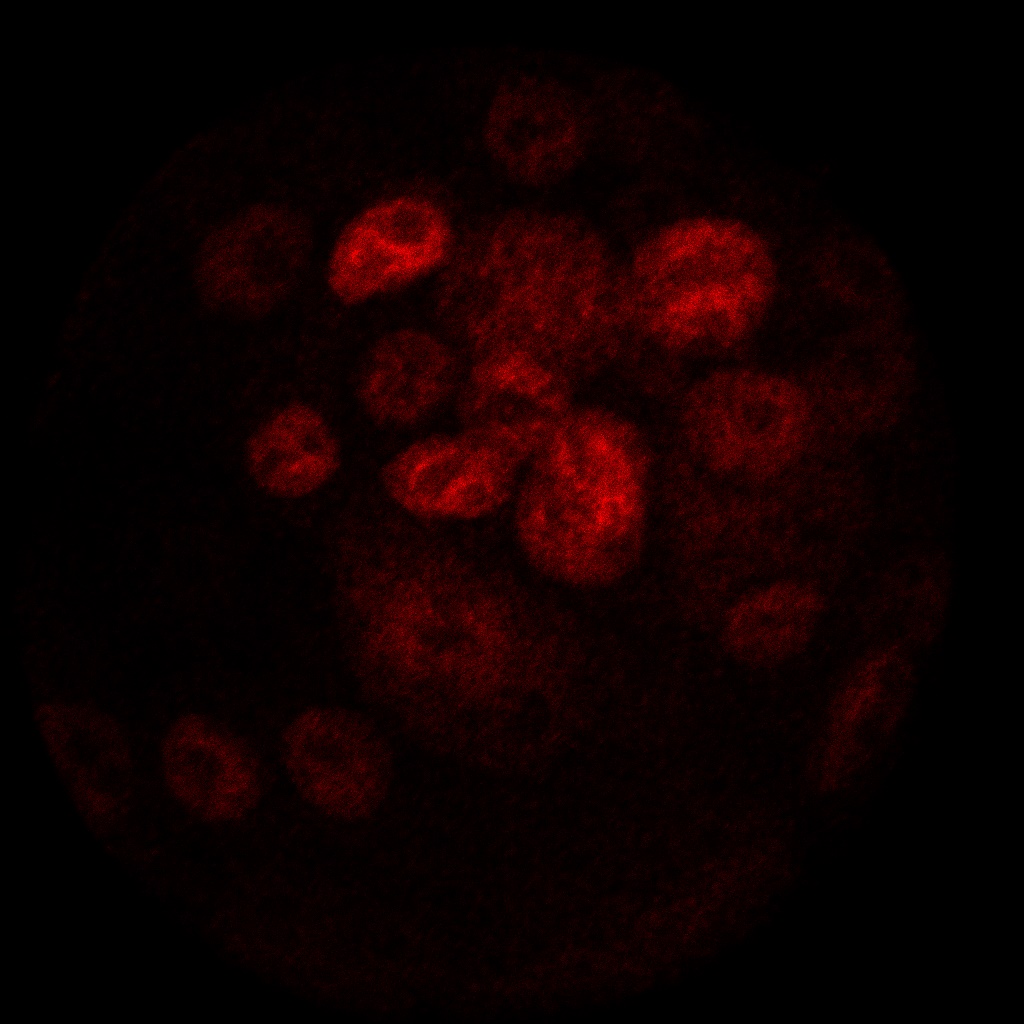

Supplement: Supplementary file 15 — Source data Fig. 5 [file 44318_2024_329_MOESM15_ESM.zip › SD figure 5/5F/Blastocyst_Setd1ab NANOG.jpg]

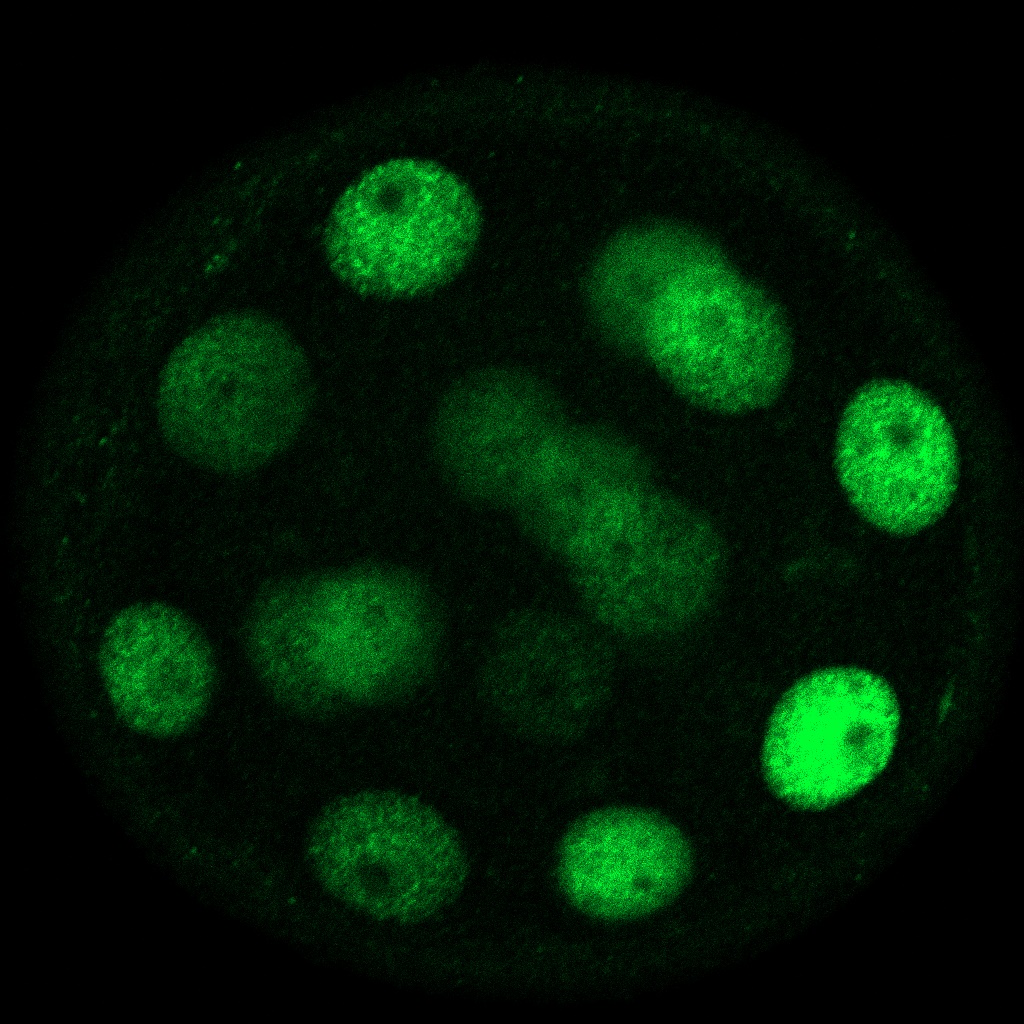

Supplement: Supplementary file 15 — Source data Fig. 5 [file 44318_2024_329_MOESM15_ESM.zip › SD figure 5/5F/Morula_Control_CDX2.jpg]

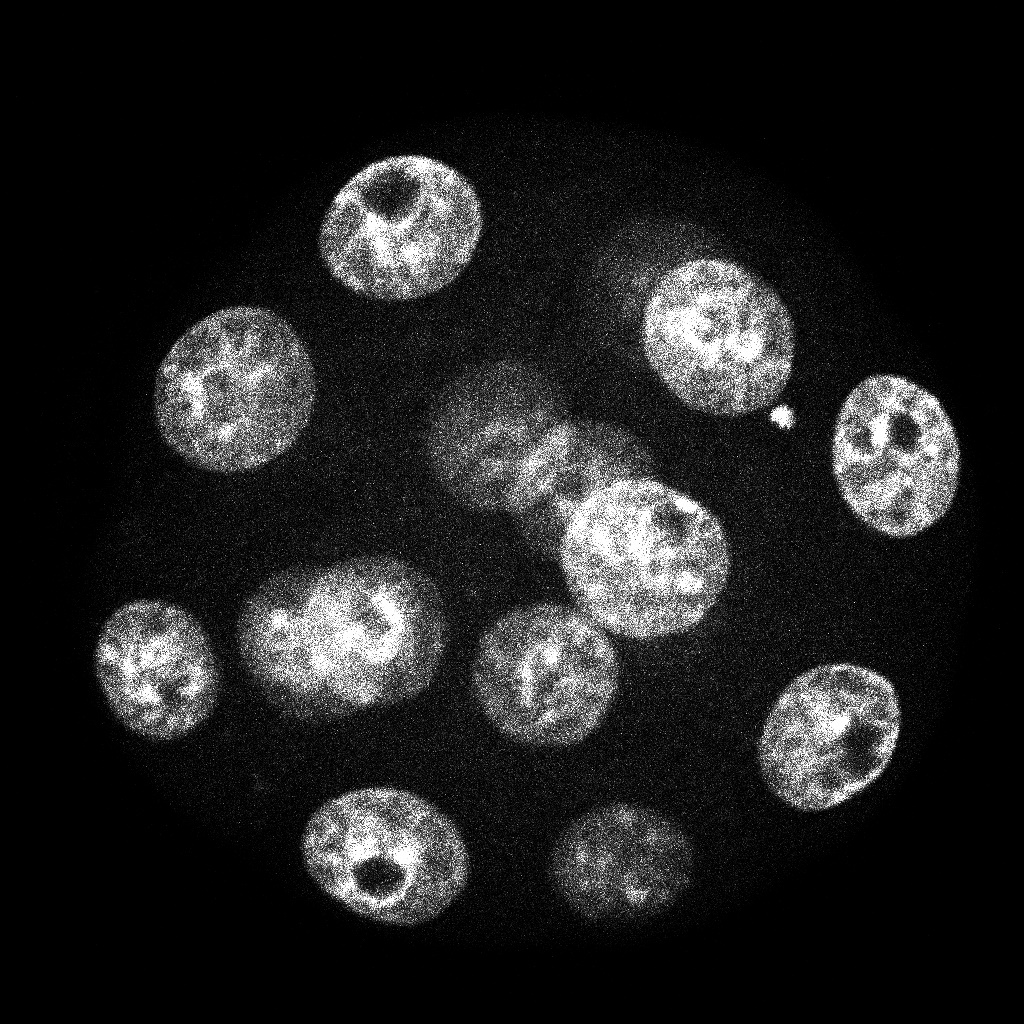

Supplement: Supplementary file 15 — Source data Fig. 5 [file 44318_2024_329_MOESM15_ESM.zip › SD figure 5/5F/Morula_Control_DAPI_CDX2.jpg]

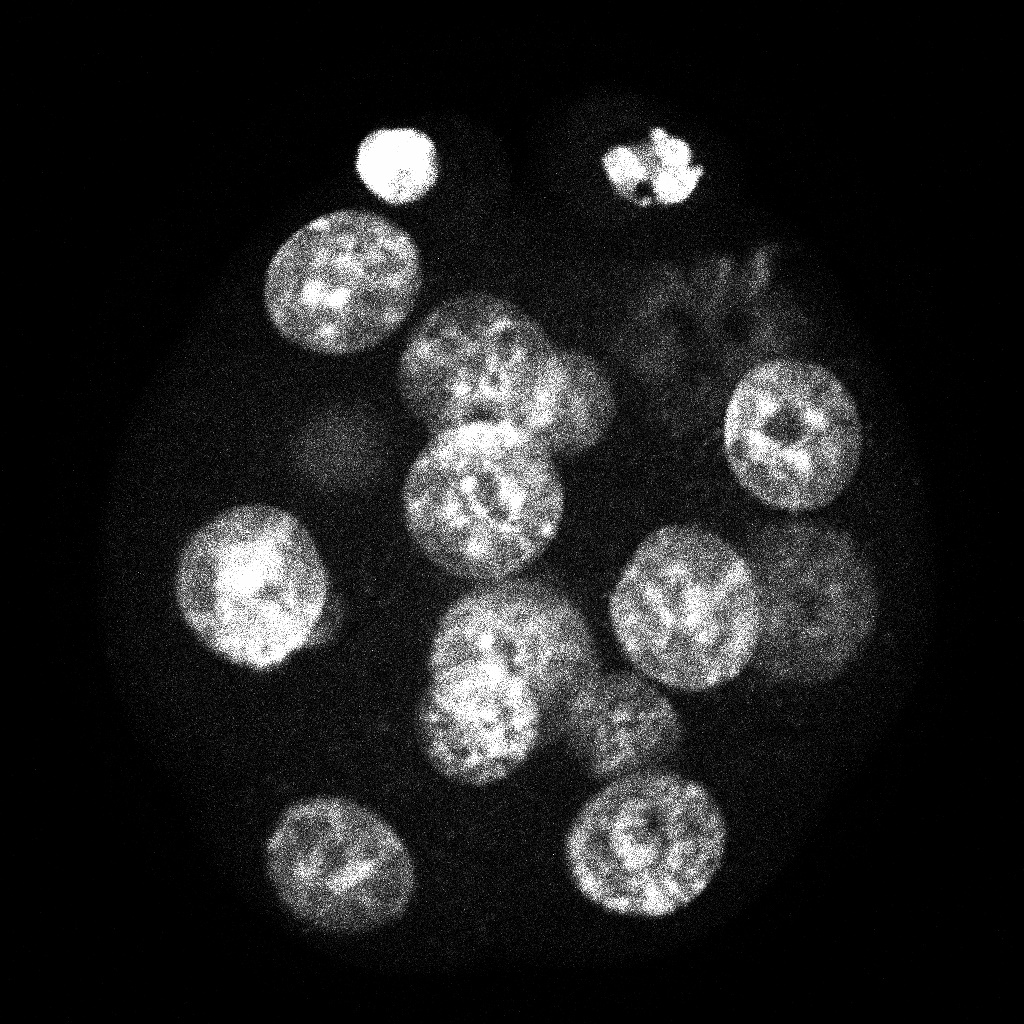

Supplement: Supplementary file 15 — Source data Fig. 5 [file 44318_2024_329_MOESM15_ESM.zip › SD figure 5/5F/Morula_Control_DAPI_NANOG.jpg]

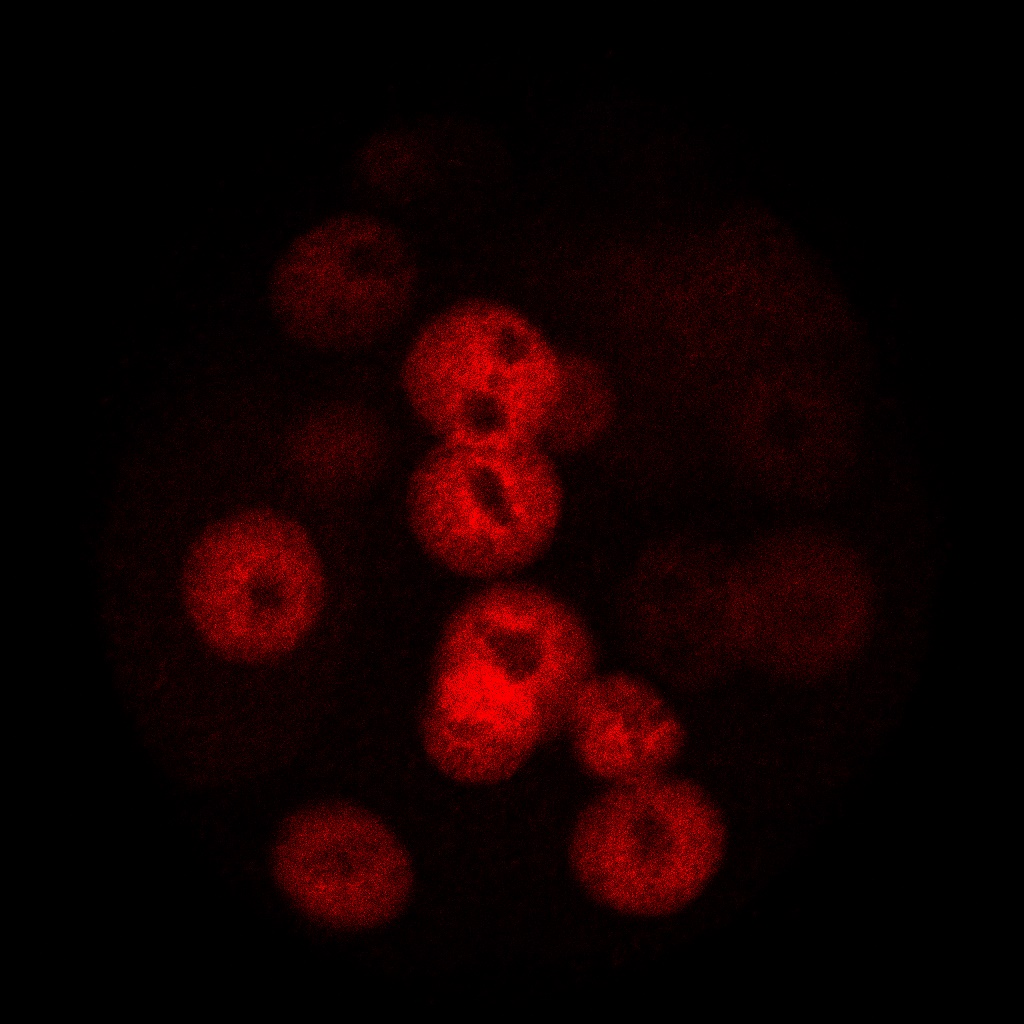

Supplement: Supplementary file 15 — Source data Fig. 5 [file 44318_2024_329_MOESM15_ESM.zip › SD figure 5/5F/Morula_Control_NANOG.jpg]

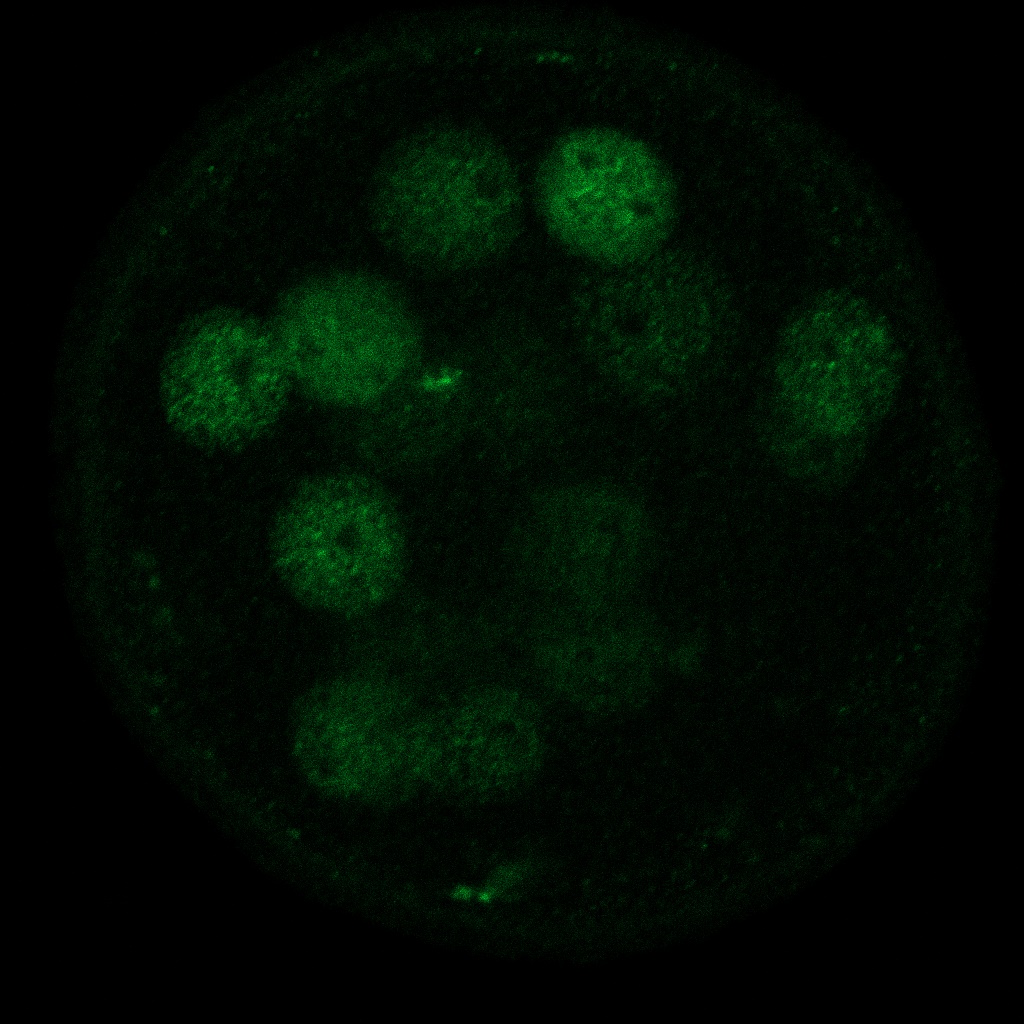

Supplement: Supplementary file 15 — Source data Fig. 5 [file 44318_2024_329_MOESM15_ESM.zip › SD figure 5/5F/Morula_Setd1ab CDX2.jpg]

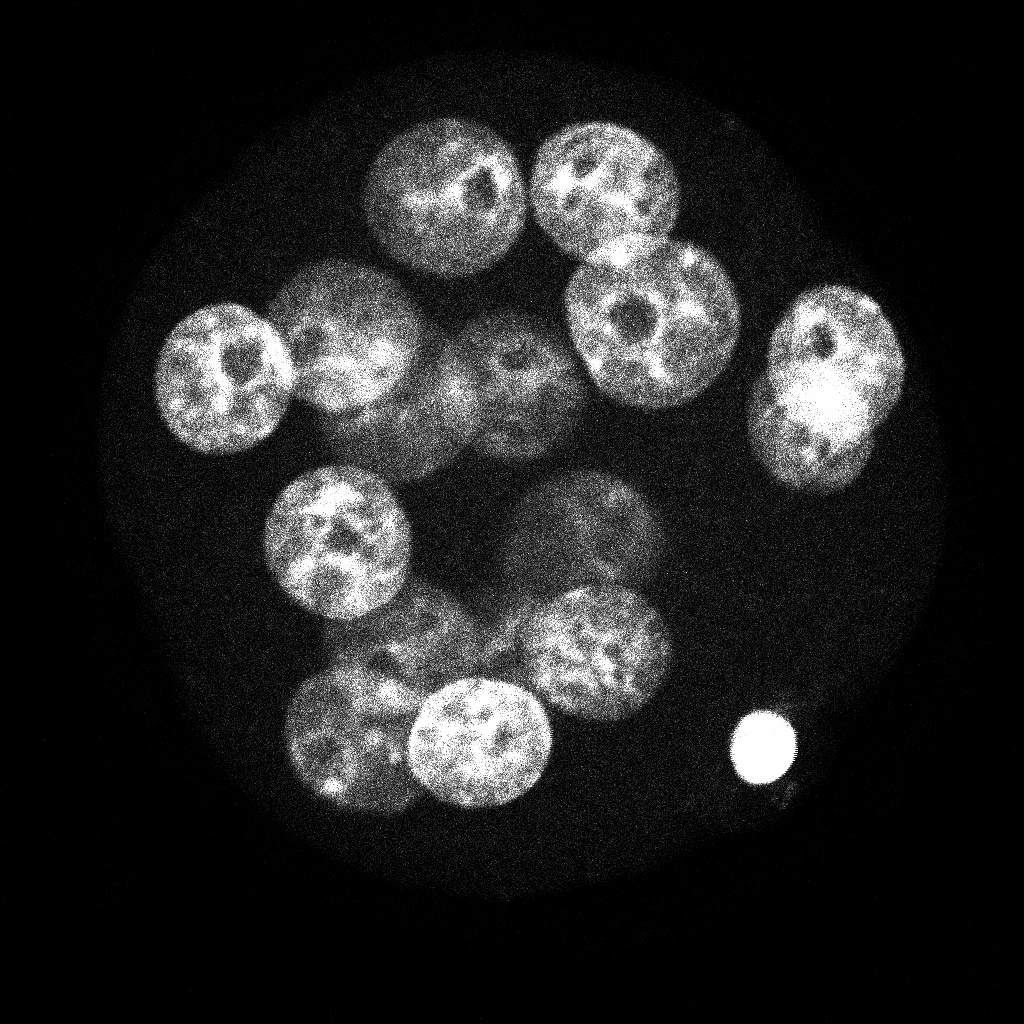

Supplement: Supplementary file 15 — Source data Fig. 5 [file 44318_2024_329_MOESM15_ESM.zip › SD figure 5/5F/Morula_Setd1ab KD_DAPI_CDX2.jpg]

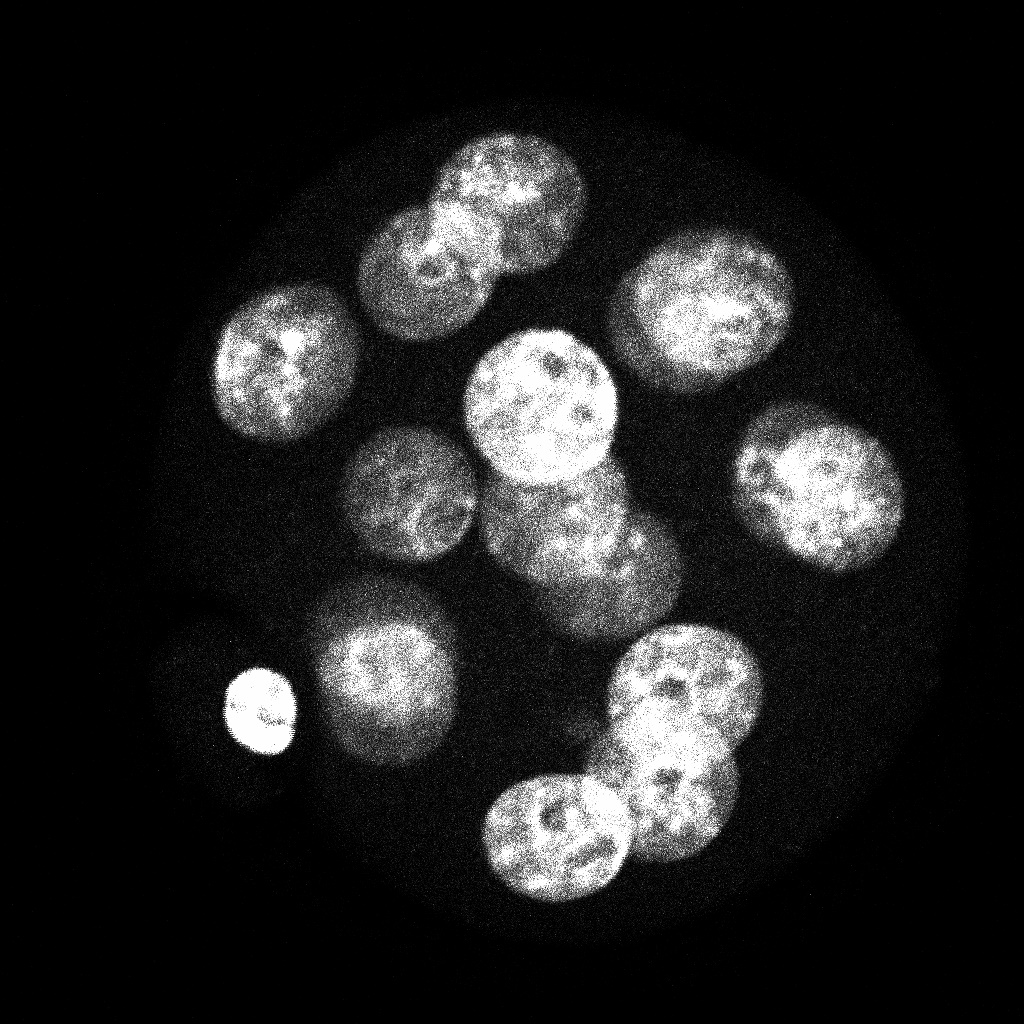

Supplement: Supplementary file 15 — Source data Fig. 5 [file 44318_2024_329_MOESM15_ESM.zip › SD figure 5/5F/Morula_Setd1ab KD_DAPI_NANOG.jpg]

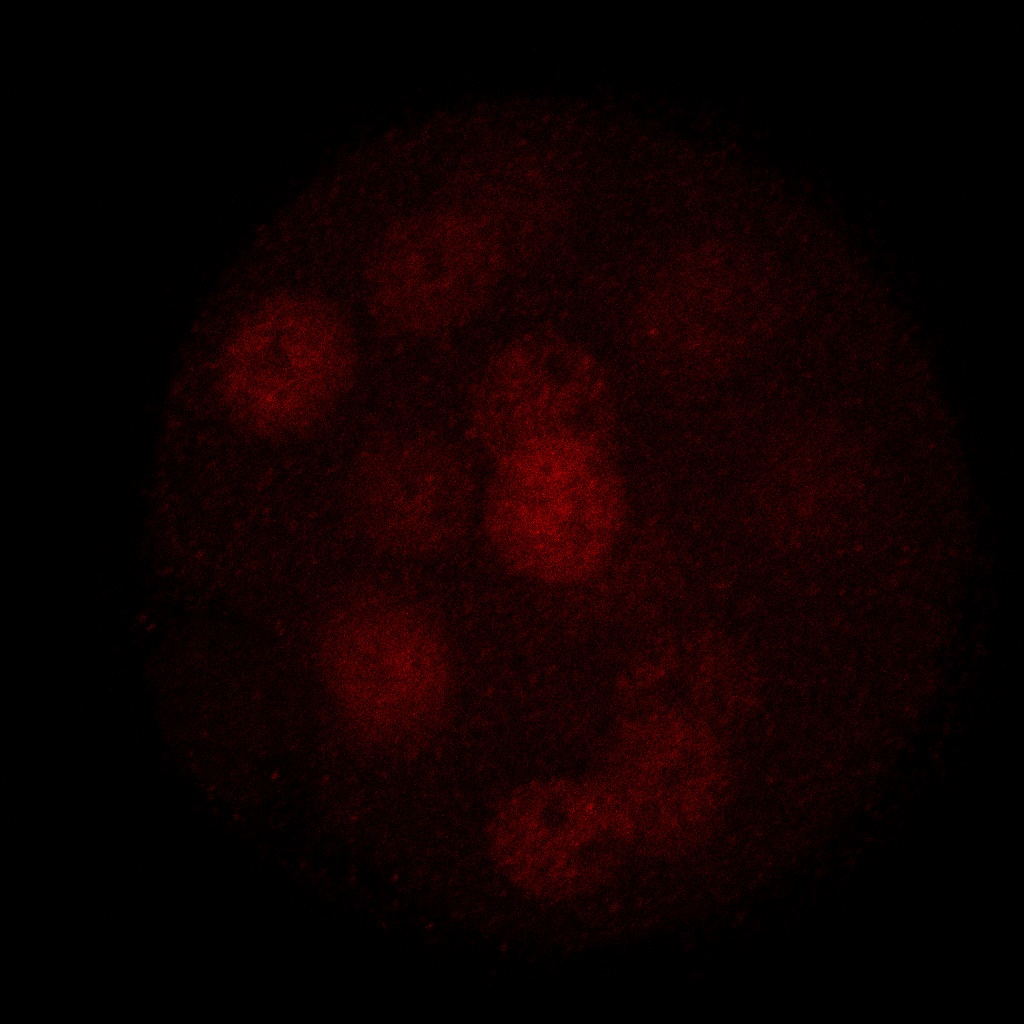

Supplement: Supplementary file 15 — Source data Fig. 5 [file 44318_2024_329_MOESM15_ESM.zip › SD figure 5/5F/Morula_Setd1ab NANOG.jpg]

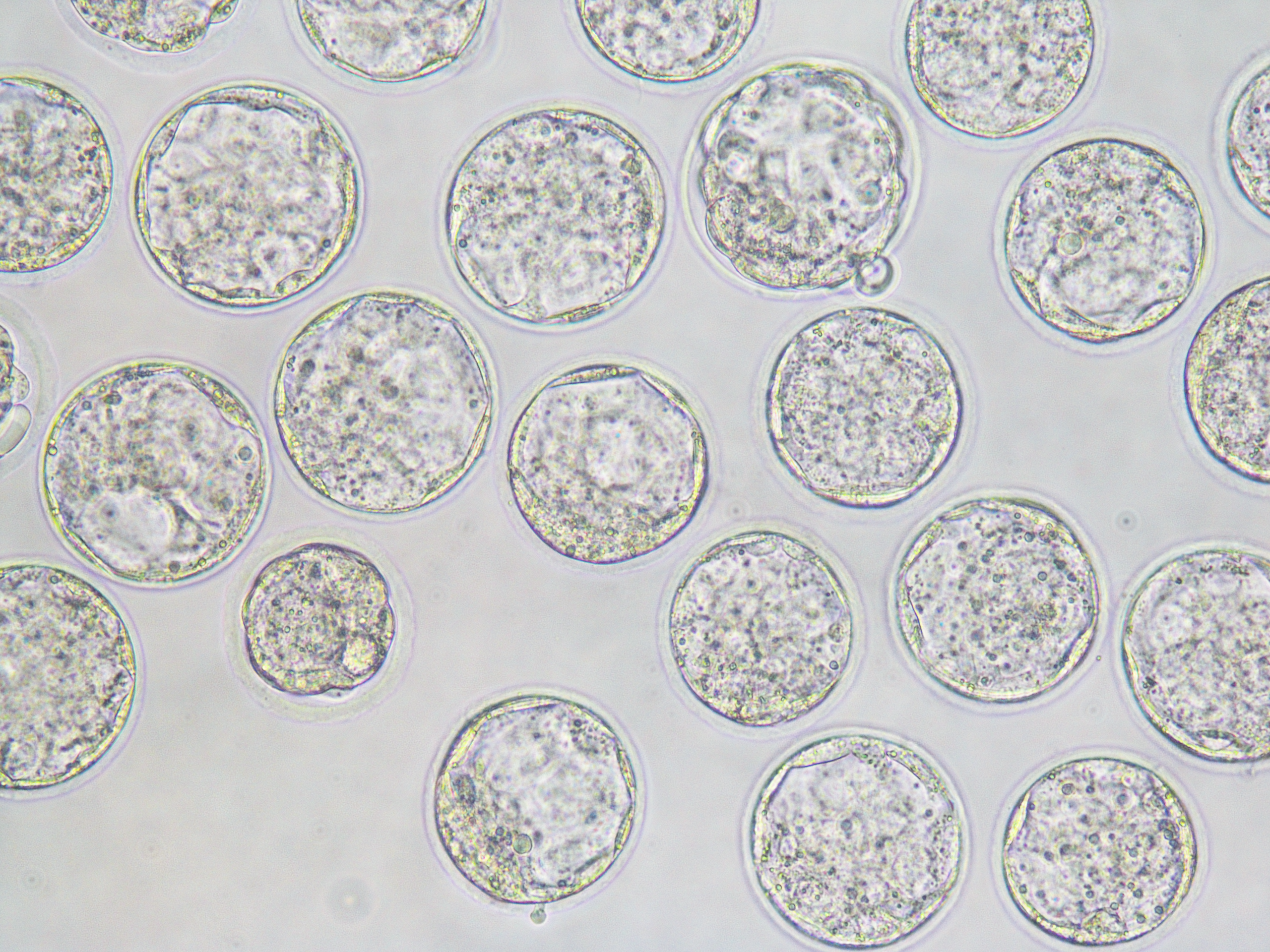

Supplement: Supplementary file 15 — Source data Fig. 5 [file 44318_2024_329_MOESM15_ESM.zip › SD figure 5/5I/Control.tif]

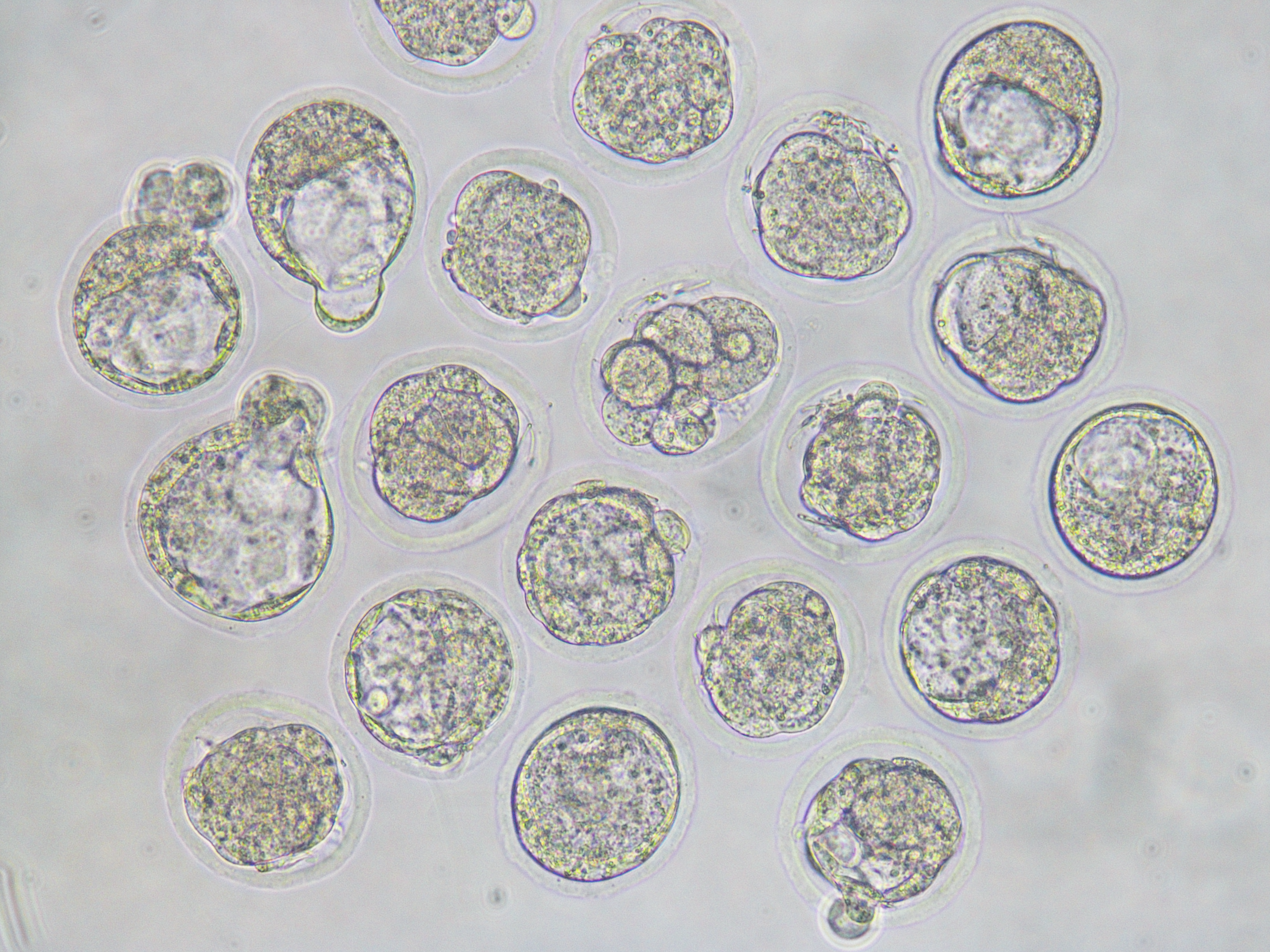

Supplement: Supplementary file 15 — Source data Fig. 5 [file 44318_2024_329_MOESM15_ESM.zip › SD figure 5/5I/Setd1ab KD+hMUT.tif]

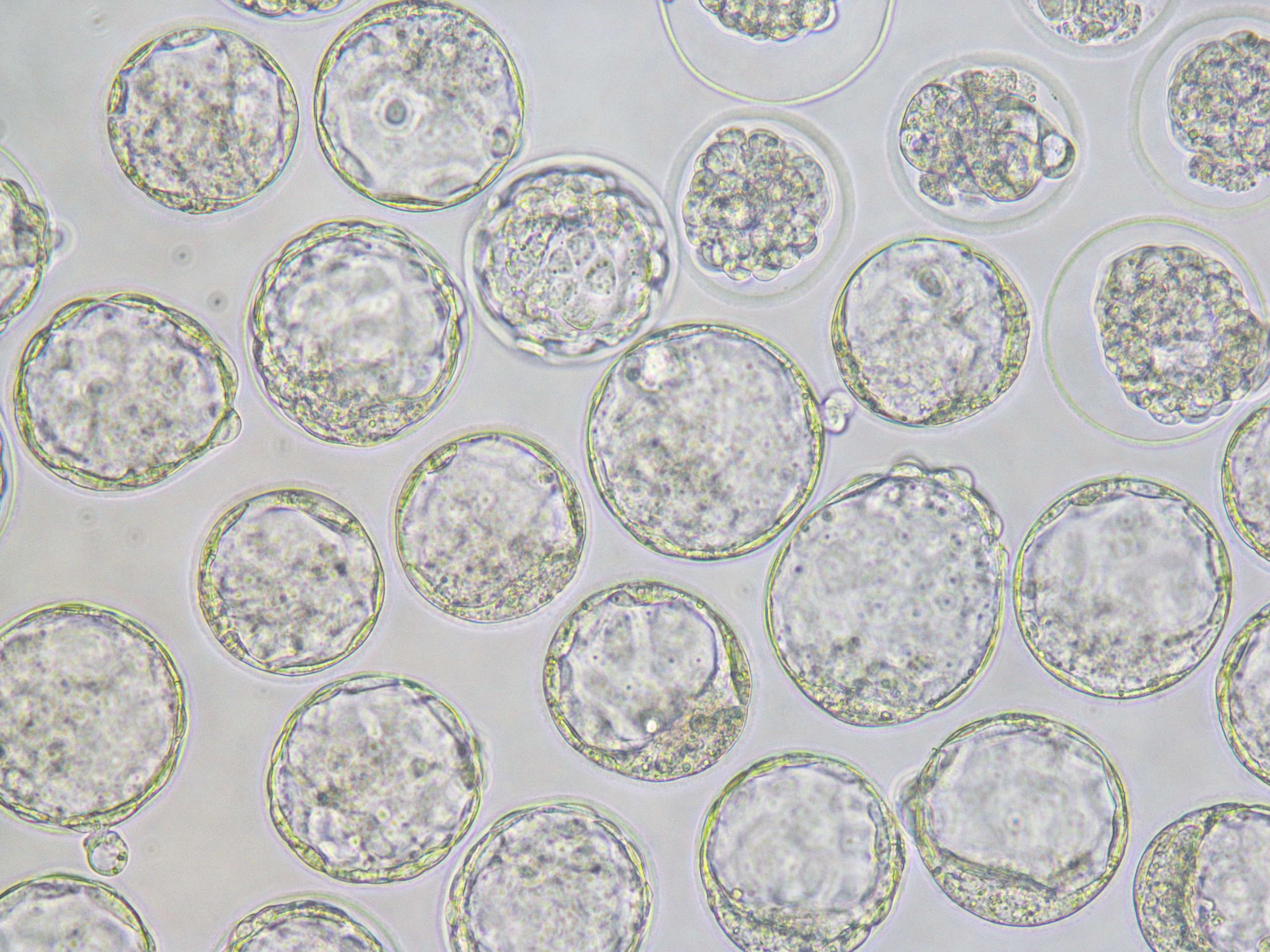

Supplement: Supplementary file 15 — Source data Fig. 5 [file 44318_2024_329_MOESM15_ESM.zip › SD figure 5/5I/Setd1ab KD+hWT.tif]

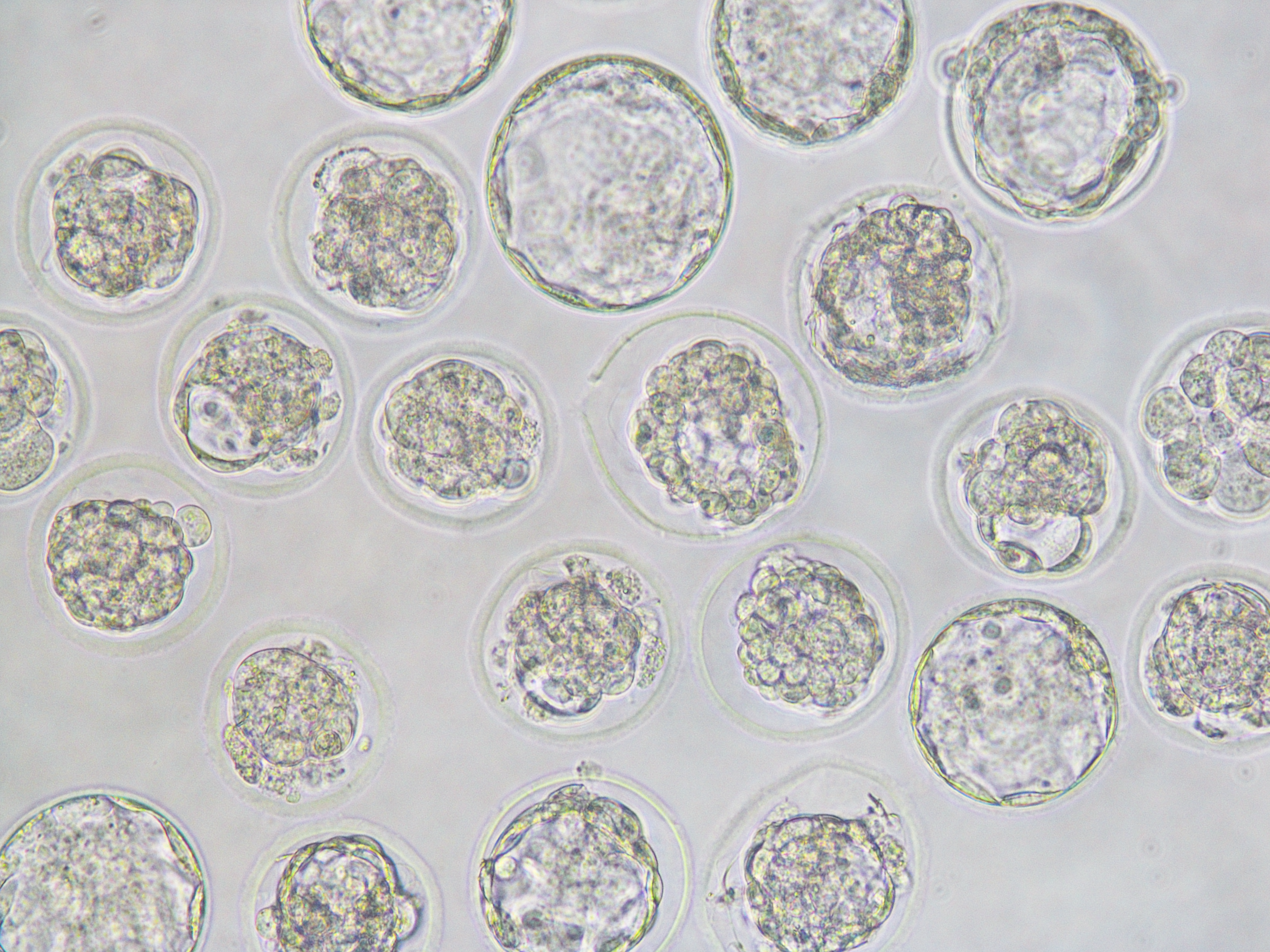

Supplement: Supplementary file 15 — Source data Fig. 5 [file 44318_2024_329_MOESM15_ESM.zip › SD figure 5/5I/Setd1ab KD.tif]

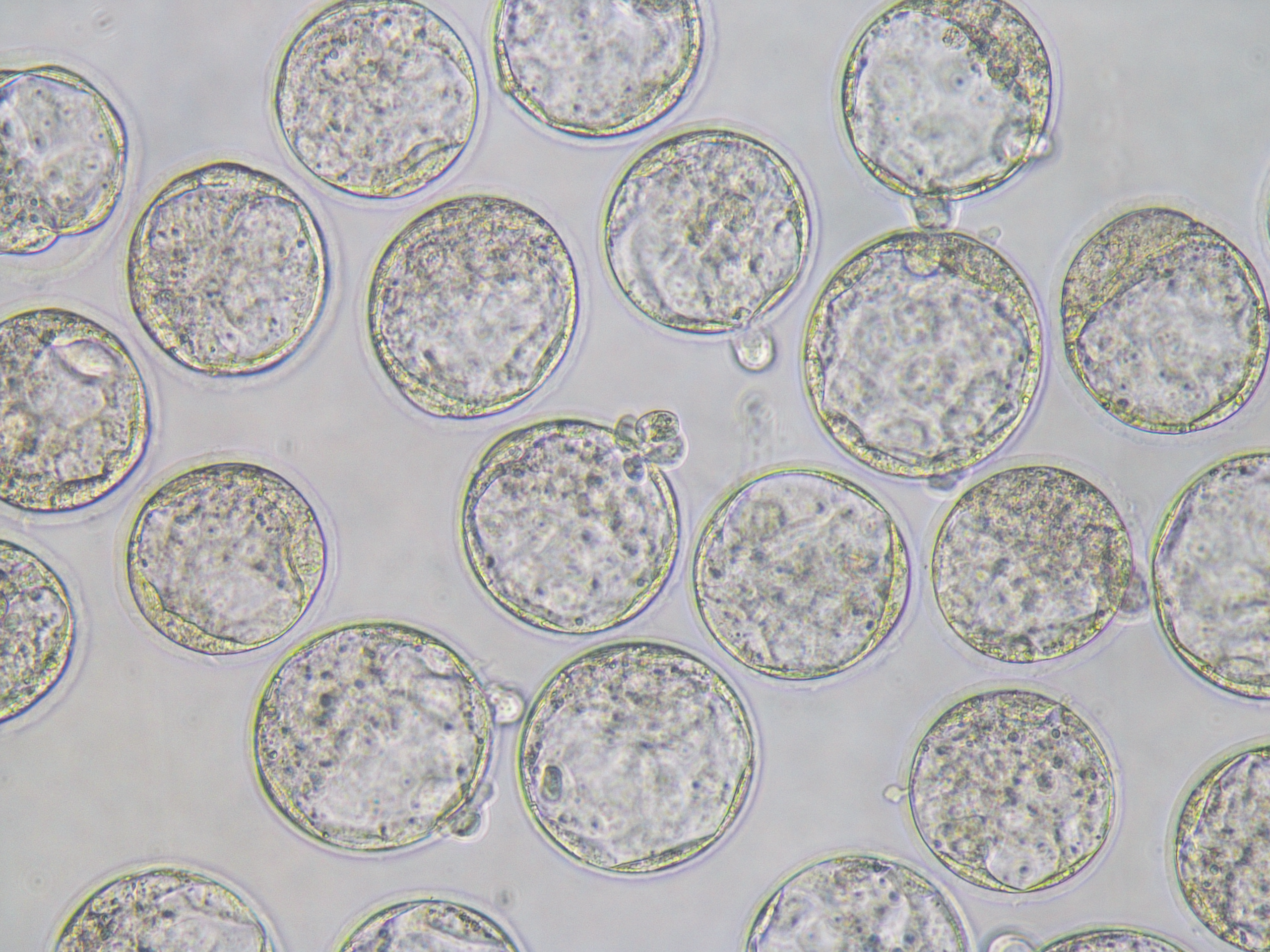

Supplement: Supplementary file 16 — Source data Fig. 6 [file 44318_2024_329_MOESM16_ESM.zip › SD figure 6/6B/Control.tif]

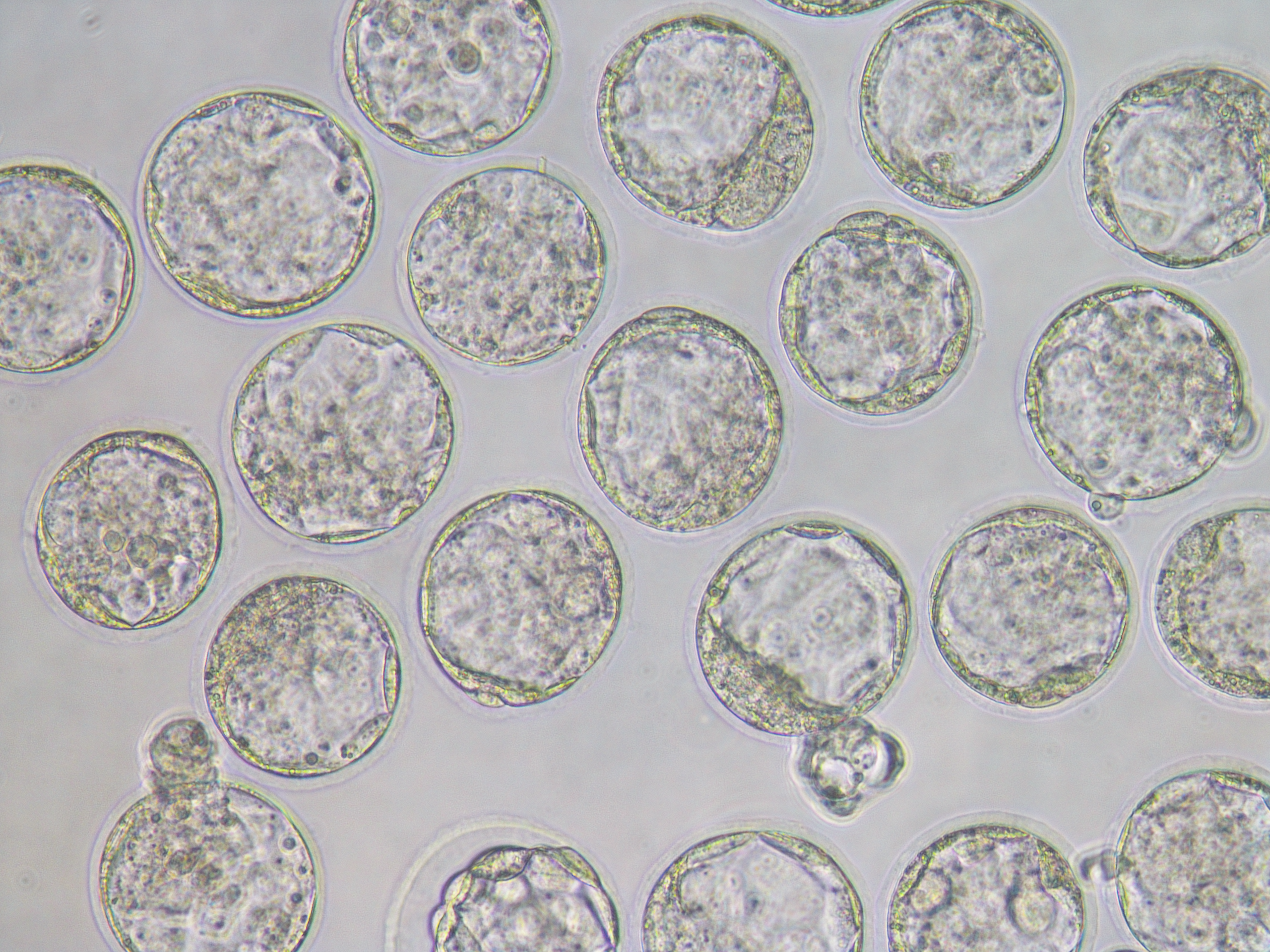

Supplement: Supplementary file 16 — Source data Fig. 6 [file 44318_2024_329_MOESM16_ESM.zip › SD figure 6/6B/CPI-455 18-44h.tif]

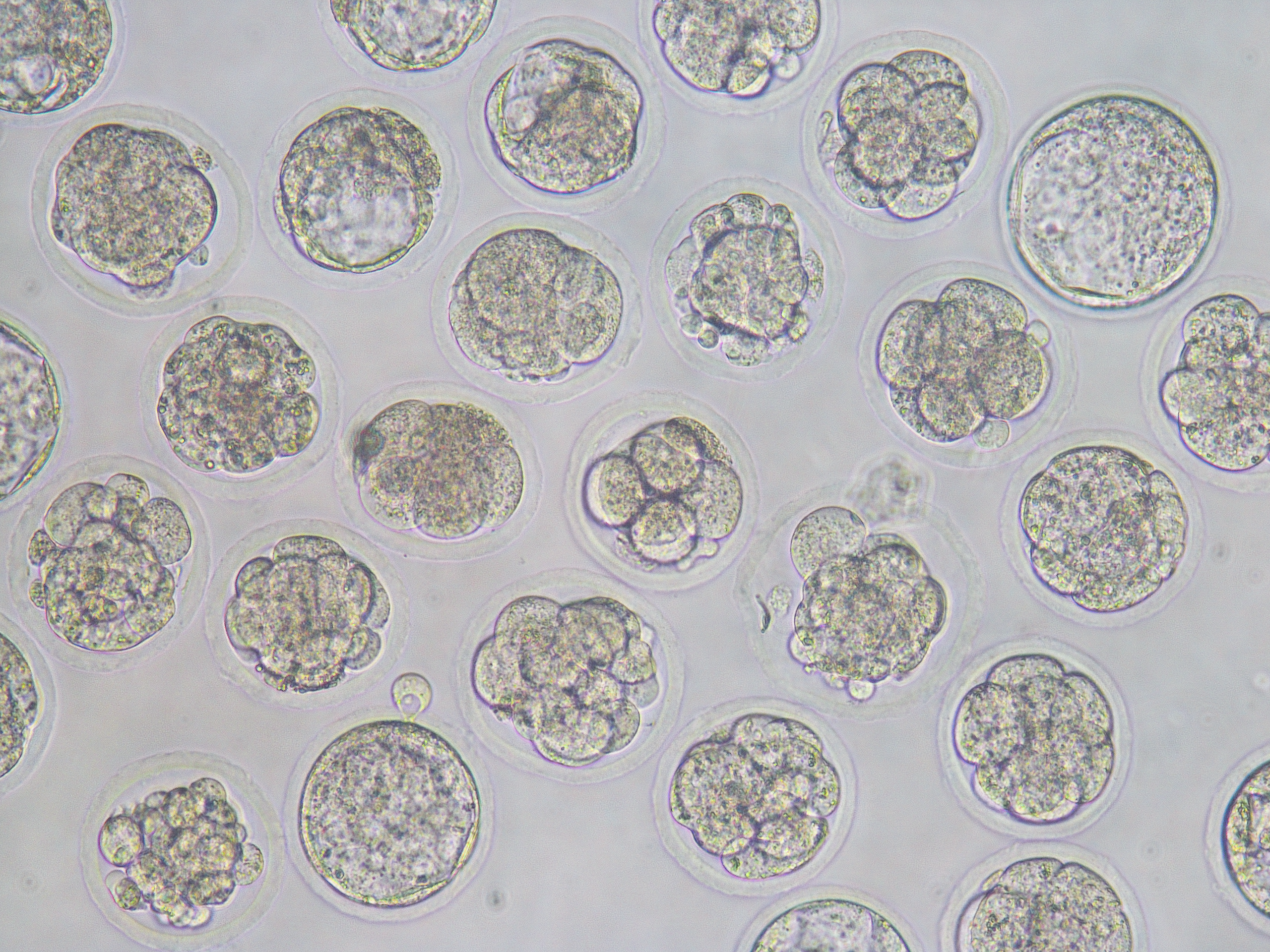

Supplement: Supplementary file 16 — Source data Fig. 6 [file 44318_2024_329_MOESM16_ESM.zip › SD figure 6/6B/CPI-455 18-96h.tif]

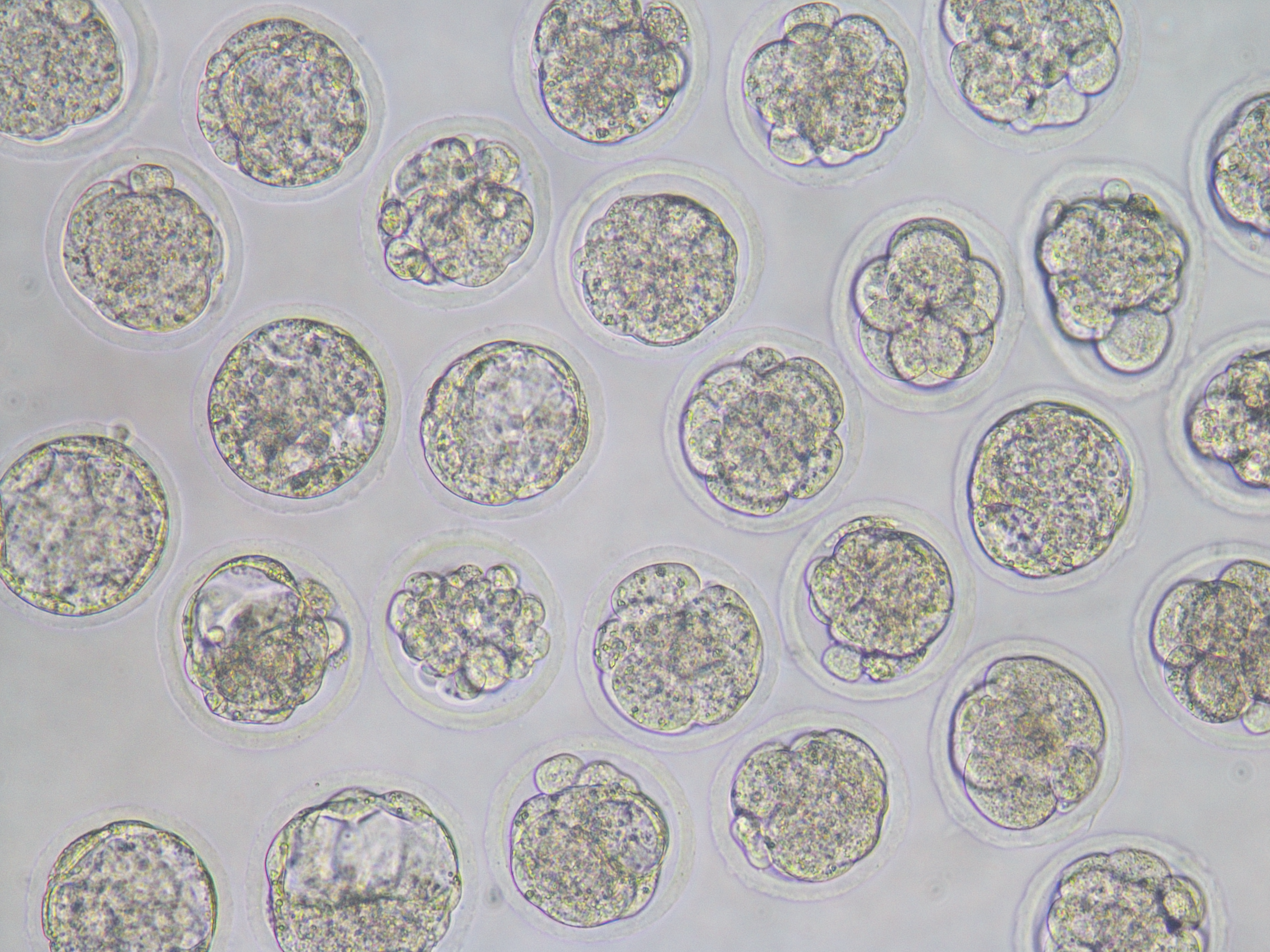

Supplement: Supplementary file 16 — Source data Fig. 6 [file 44318_2024_329_MOESM16_ESM.zip › SD figure 6/6B/CPI-455 44-96h.tif]

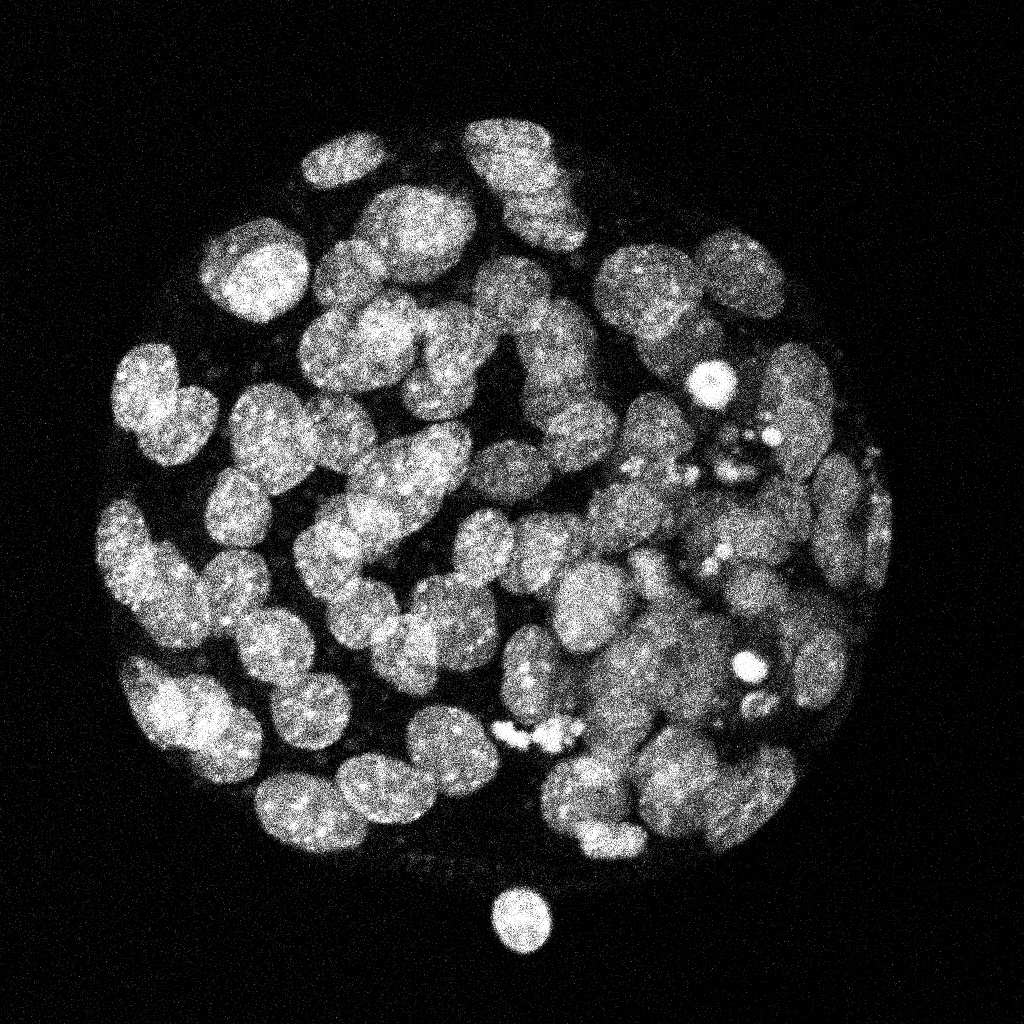

Supplement: Supplementary file 17 — Appendix Figure Source Data [file 44318_2024_329_MOESM17_ESM.zip › SD Appendix/FigS11A/S11A/Blastocyst_Control_DAPI.jpg]

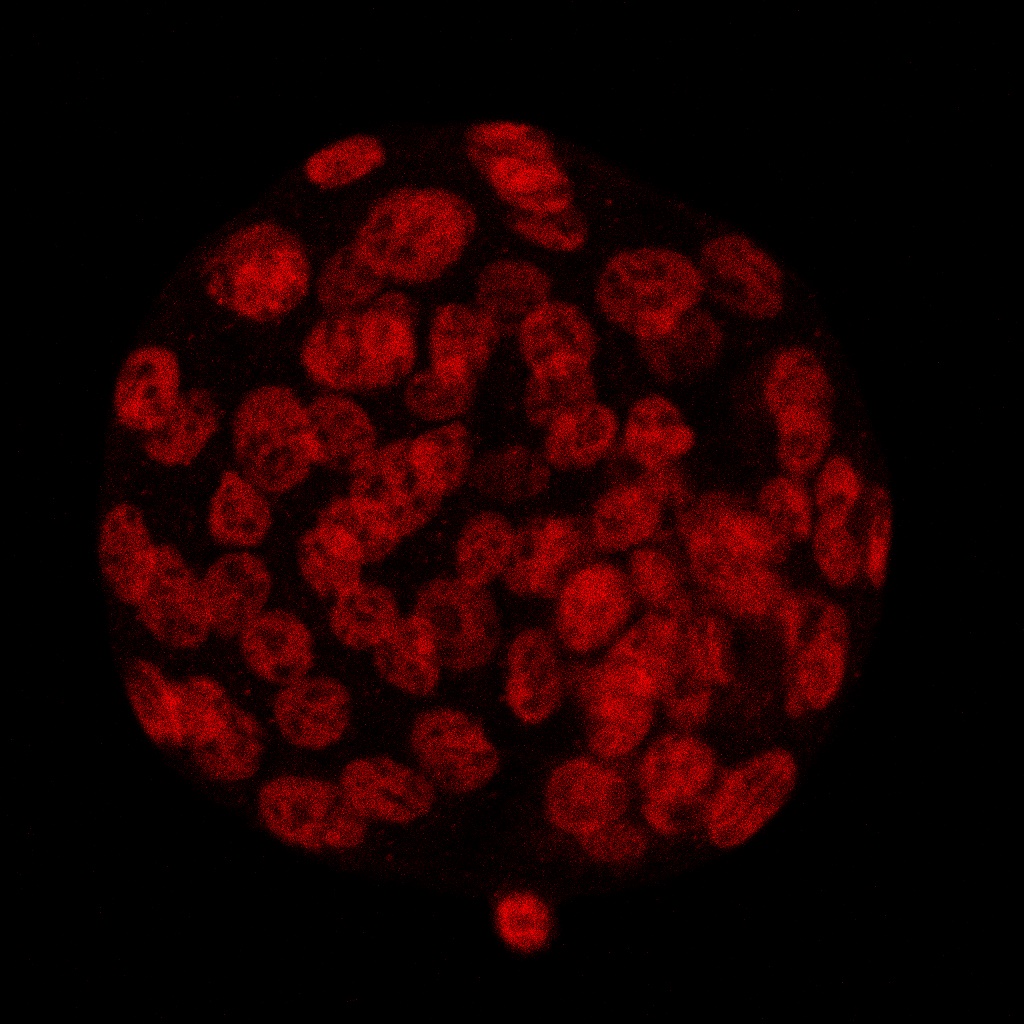

Supplement: Supplementary file 17 — Appendix Figure Source Data [file 44318_2024_329_MOESM17_ESM.zip › SD Appendix/FigS11A/S11A/Blastocyst_Control_SETD1A.jpg]

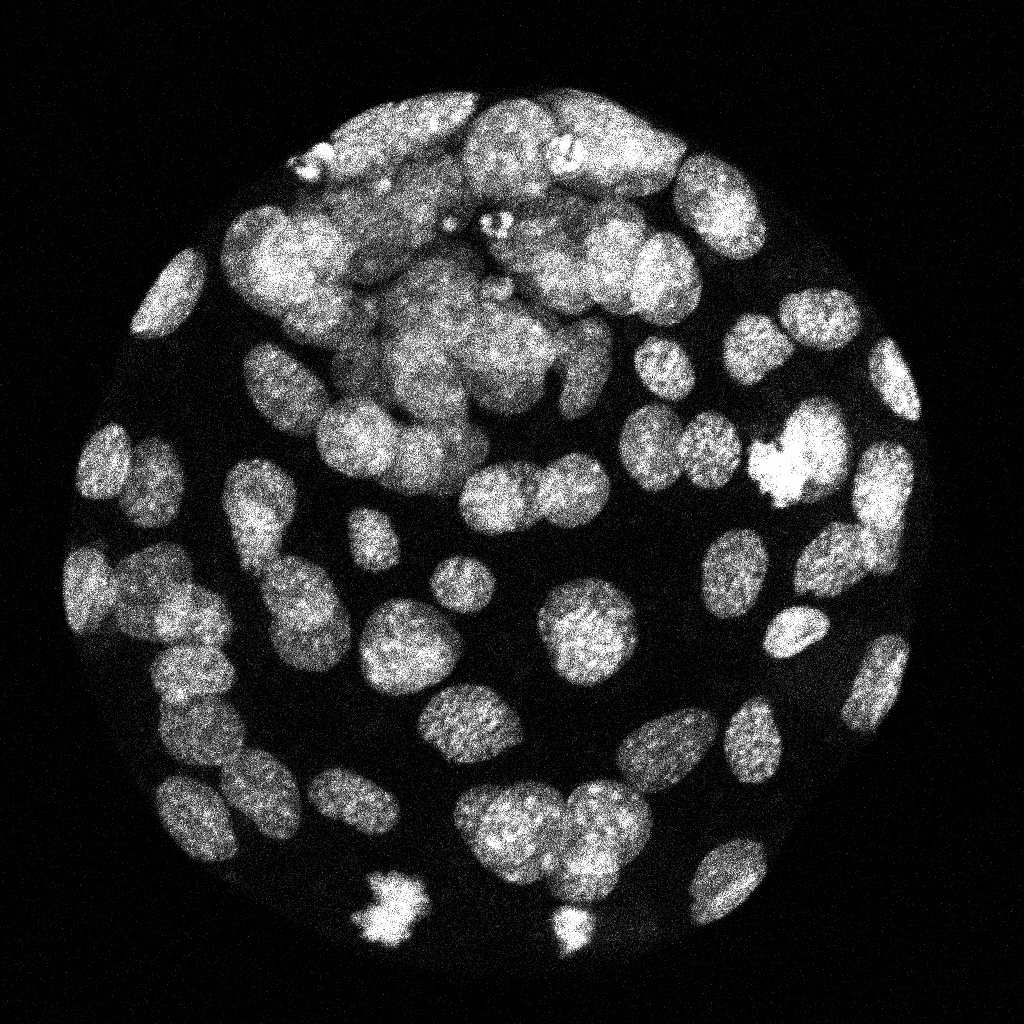

Supplement: Supplementary file 17 — Appendix Figure Source Data [file 44318_2024_329_MOESM17_ESM.zip › SD Appendix/FigS11A/S11A/Blastocyst_Setd1ab KD+hWT mRNA_DAPI.jpg]

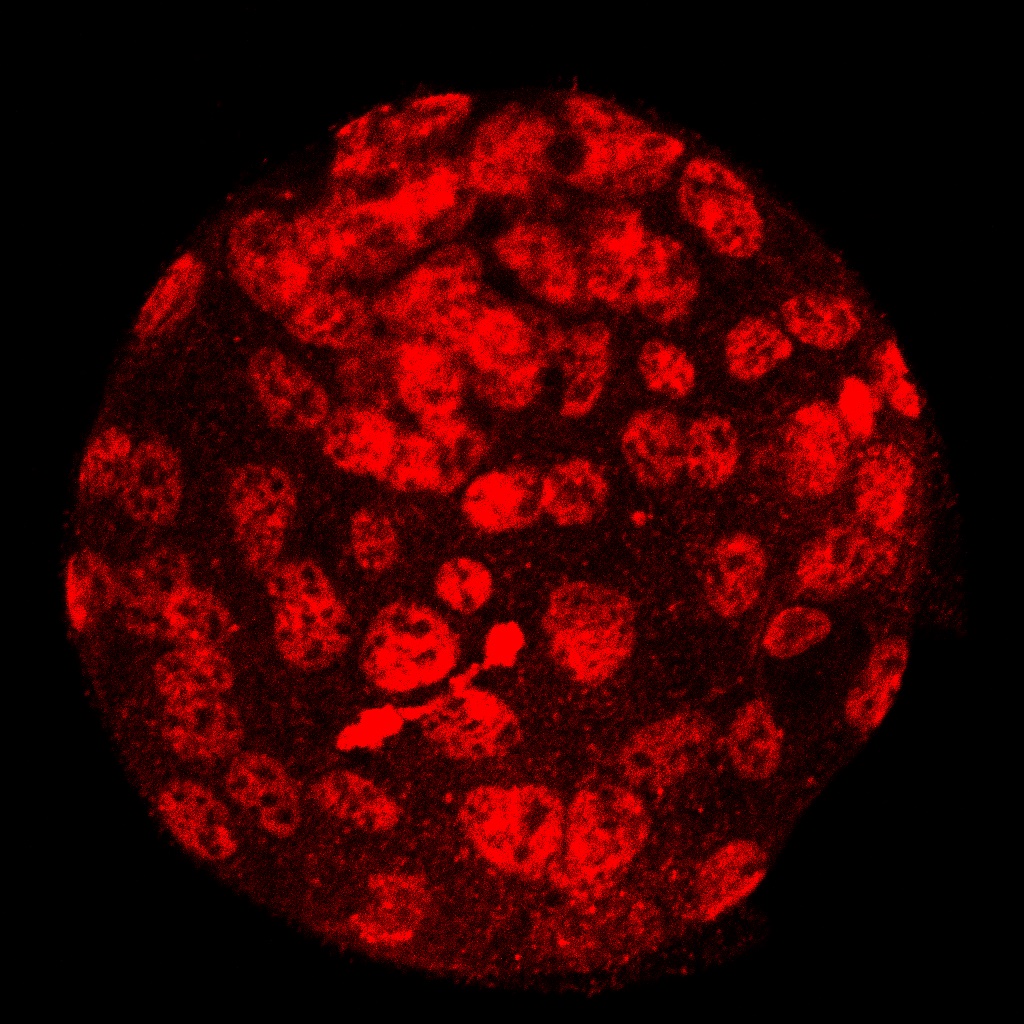

Supplement: Supplementary file 17 — Appendix Figure Source Data [file 44318_2024_329_MOESM17_ESM.zip › SD Appendix/FigS11A/S11A/Blastocyst_Setd1ab KD+hWT mRNA_SETD1A.jpg]

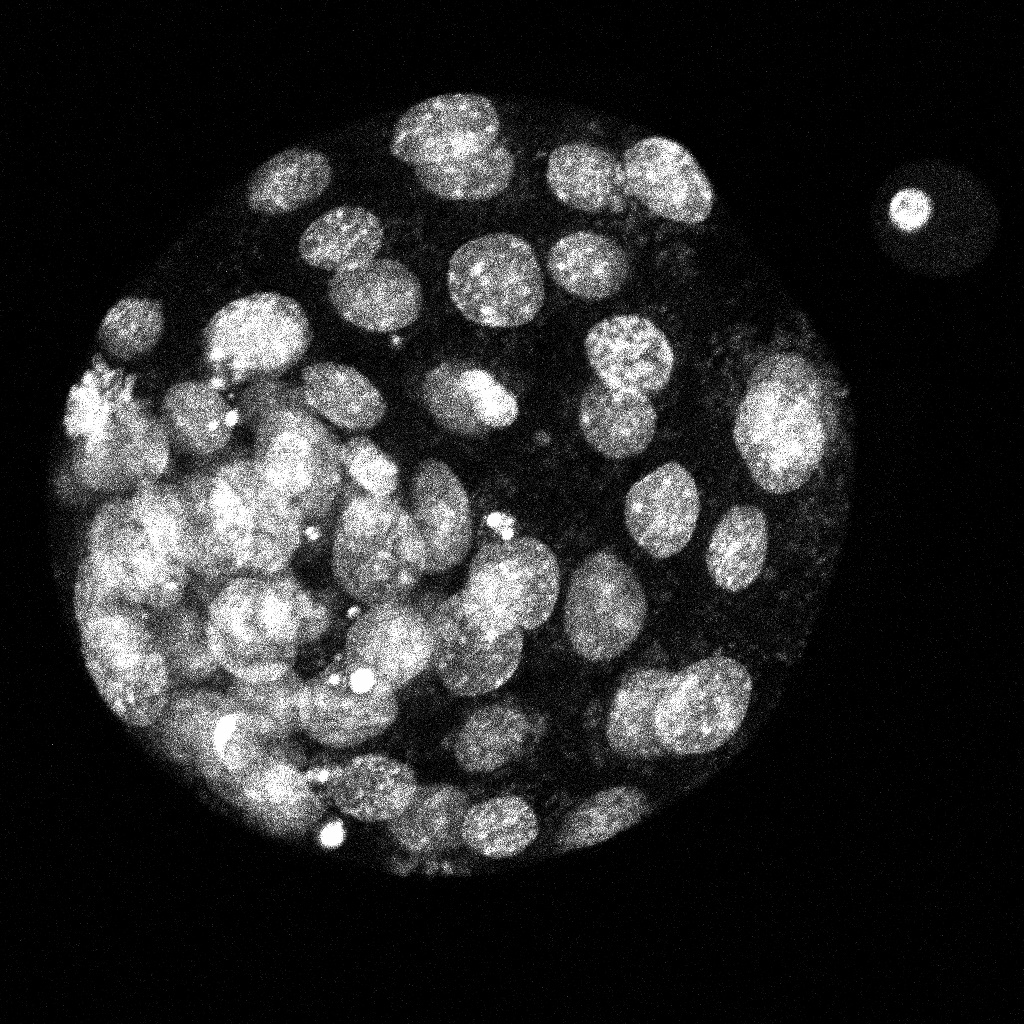

Supplement: Supplementary file 17 — Appendix Figure Source Data [file 44318_2024_329_MOESM17_ESM.zip › SD Appendix/FigS11A/S11A/Blastocyst_Setd1ab KD_DAPI.jpg]

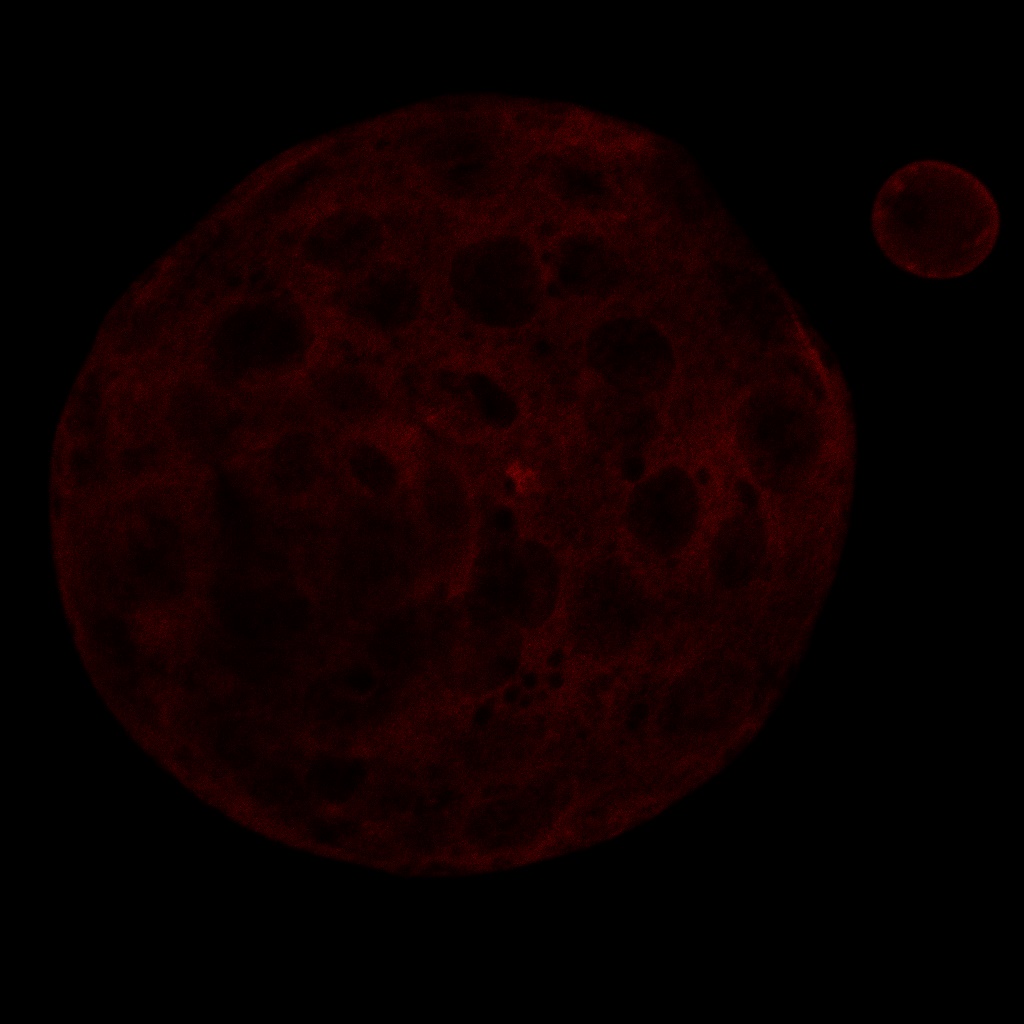

Supplement: Supplementary file 17 — Appendix Figure Source Data [file 44318_2024_329_MOESM17_ESM.zip › SD Appendix/FigS11A/S11A/Blastocyst_Setd1ab KD_SETD1A.jpg]
