# Supplementary figures and images for: Histone methyltransferases MLL2 and SETD1A/B play distinct roles in H3K4me3 deposition during the transition from totipotency to pluripotency (part 3 of 3)
Source: EMBO J. 2024 Dec 5;44(2):437–56. doi: 10.1038/s44318-024-00329-5 (PMC11730331; doi:10.1038/s44318-024-00329-5)

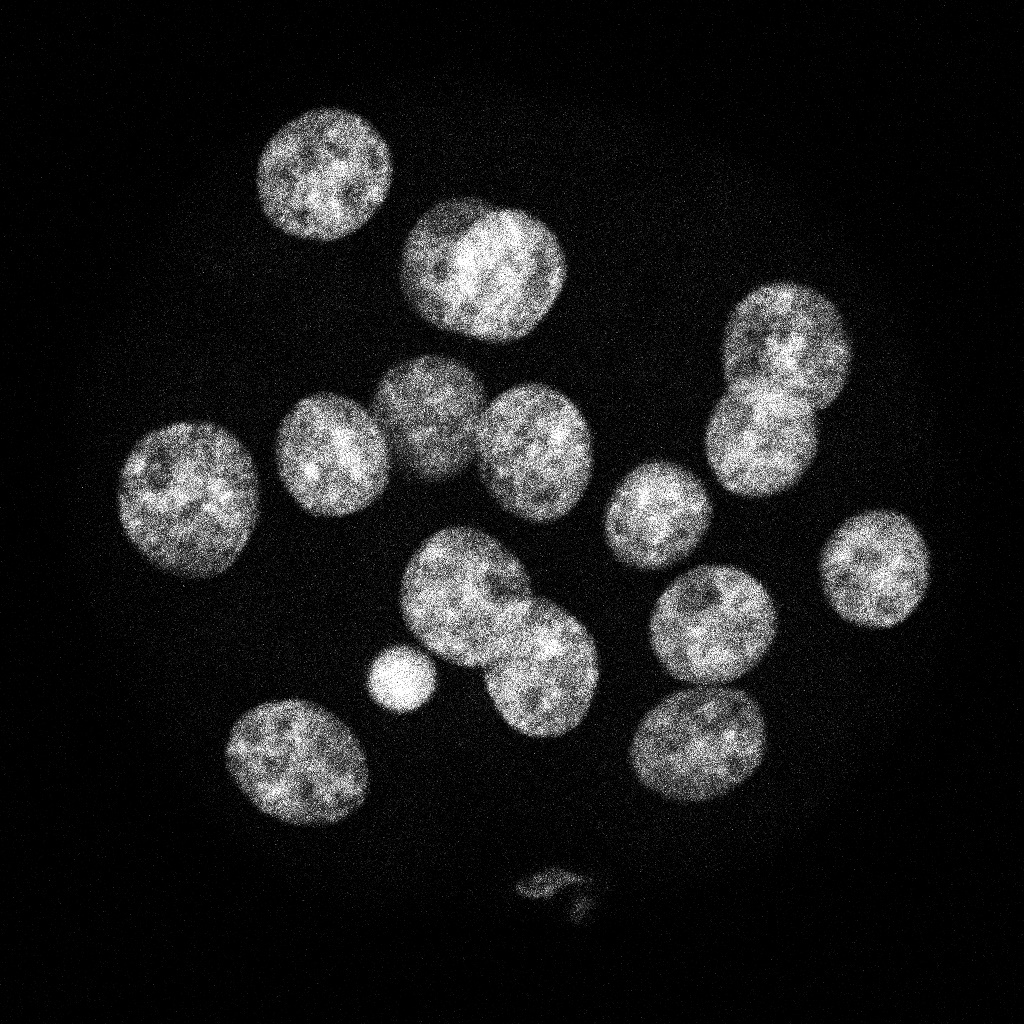

Supplement: Supplementary file 17 — Appendix Figure Source Data [file 44318_2024_329_MOESM17_ESM.zip › SD Appendix/FigS5F/S5F/Morula_Trp+CPI 4h_DAPI.jpg]

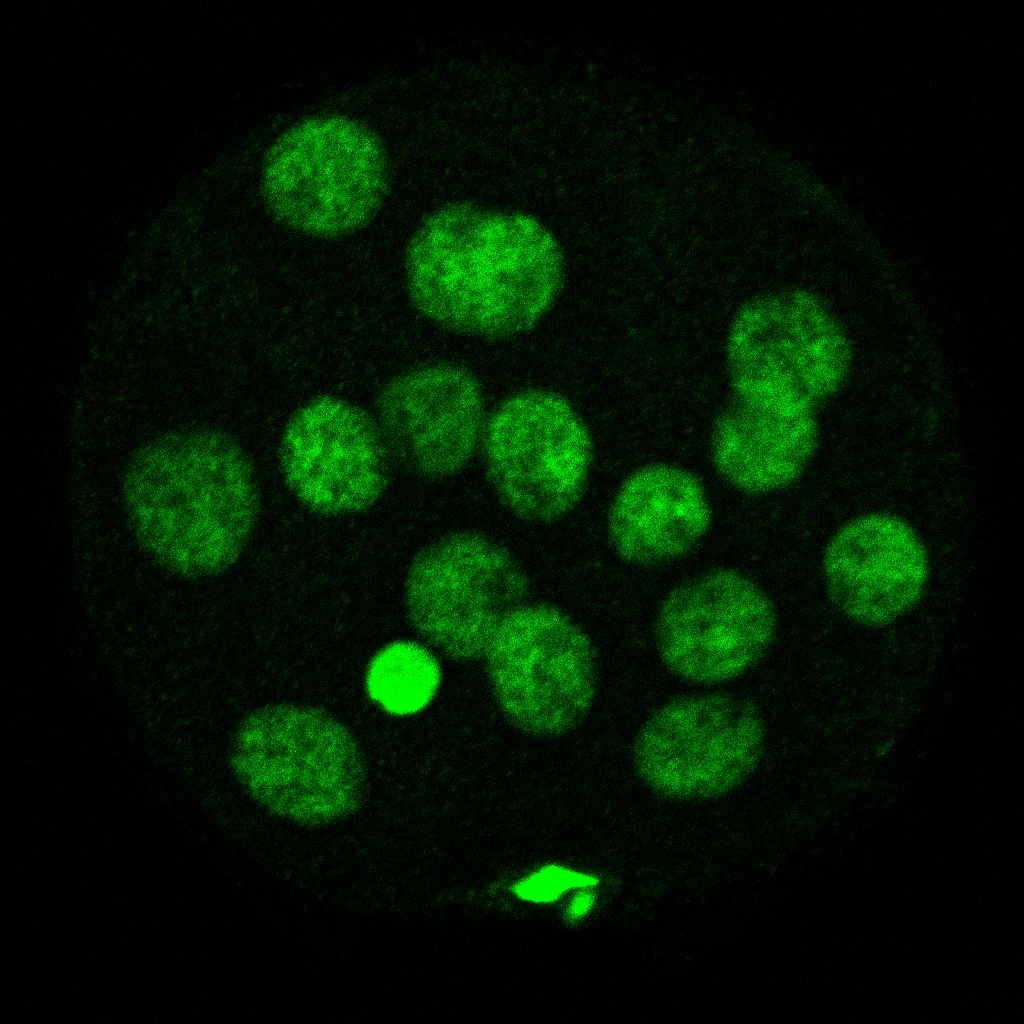

Supplement: Supplementary file 17 — Appendix Figure Source Data [file 44318_2024_329_MOESM17_ESM.zip › SD Appendix/FigS5F/S5F/Morula_Trp+CPI 4h_H3K4me3.jpg]

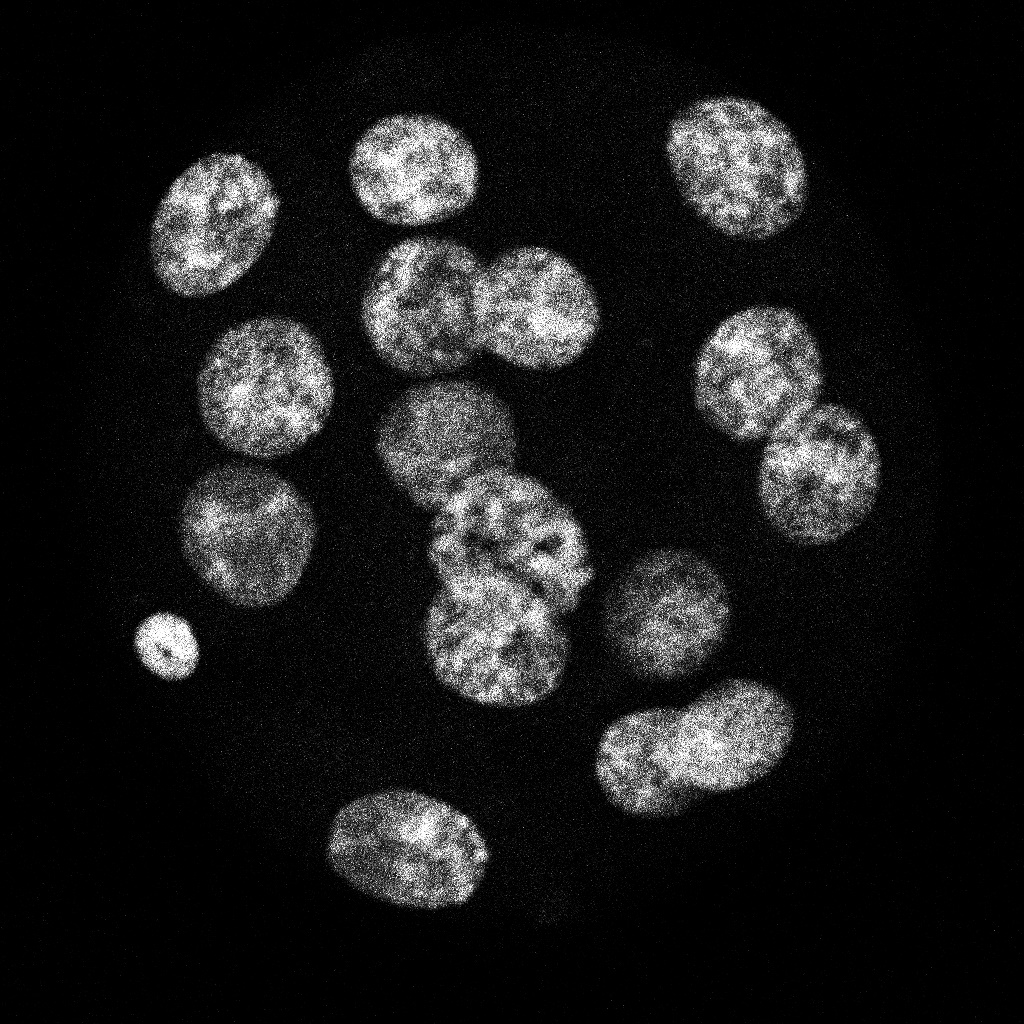

Supplement: Supplementary file 17 — Appendix Figure Source Data [file 44318_2024_329_MOESM17_ESM.zip › SD Appendix/FigS5F/S5F/Morula_Trp+CPI 6h_DAPI.jpg]

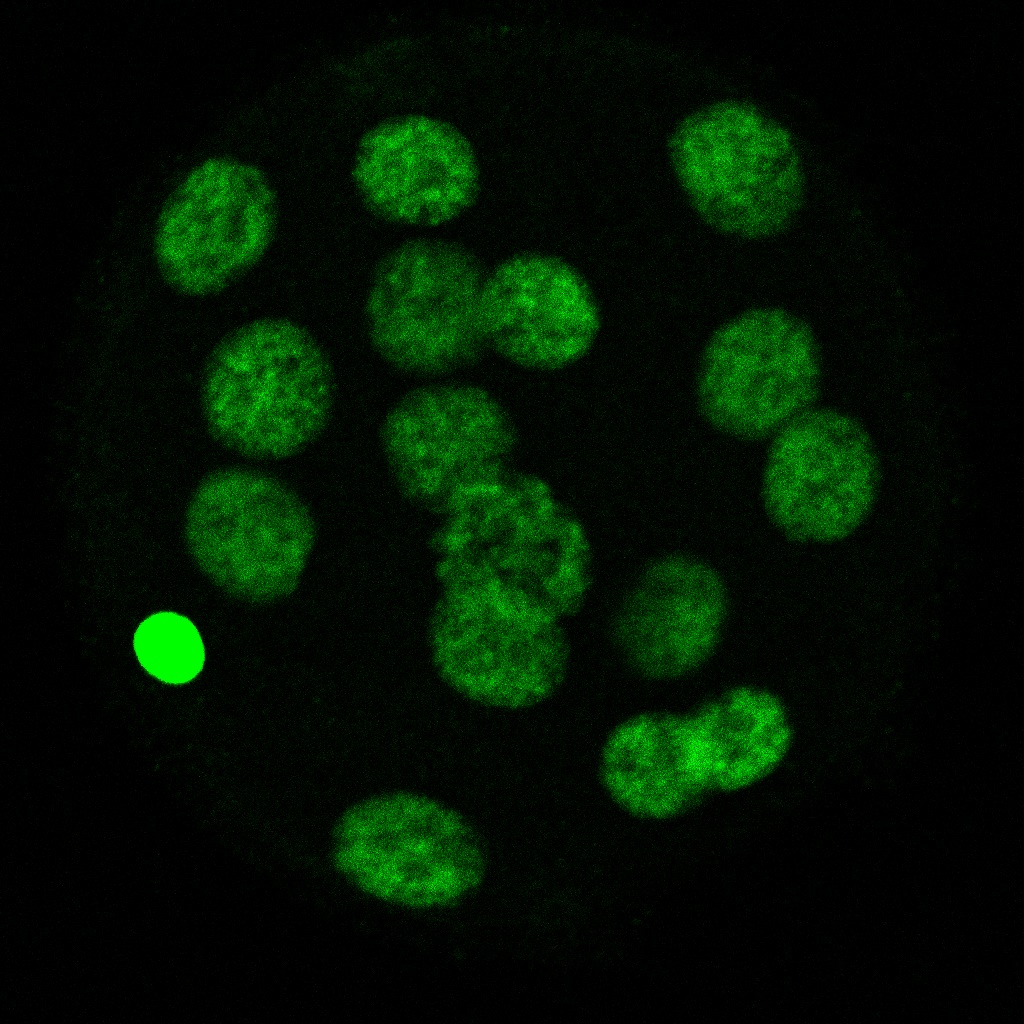

Supplement: Supplementary file 17 — Appendix Figure Source Data [file 44318_2024_329_MOESM17_ESM.zip › SD Appendix/FigS5F/S5F/Morula_Trp+CPI 6h_H3K4me3.jpg]

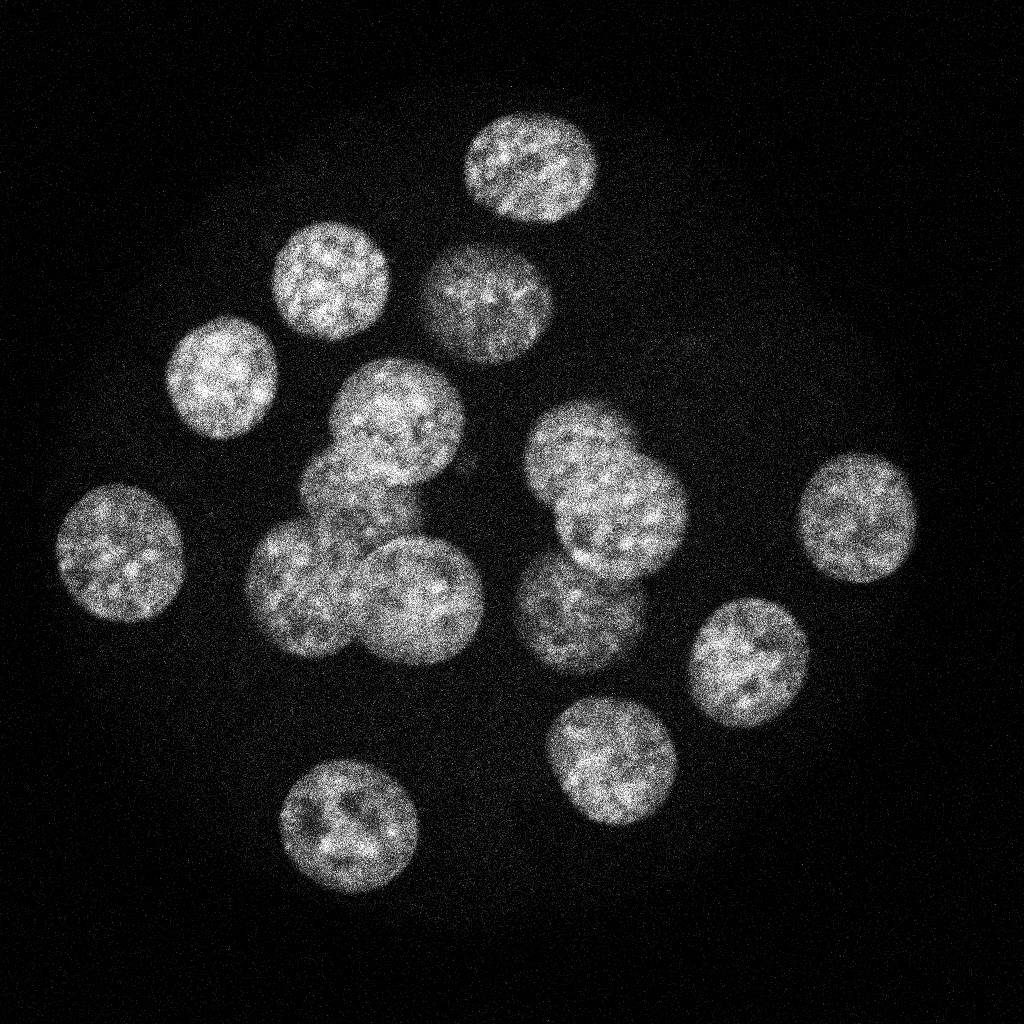

Supplement: Supplementary file 17 — Appendix Figure Source Data [file 44318_2024_329_MOESM17_ESM.zip › SD Appendix/FigS5F/S5F/Morula_Trp2h_DAPI.jpg]

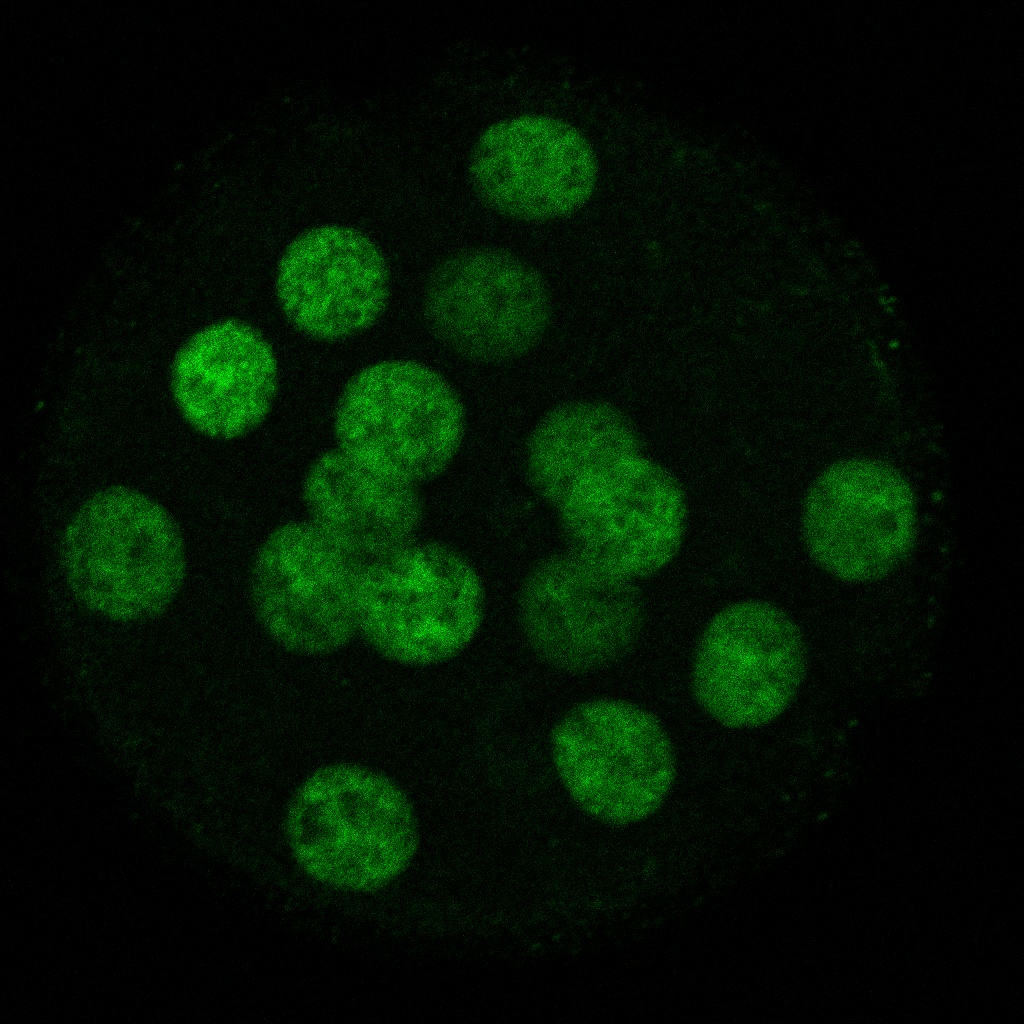

Supplement: Supplementary file 17 — Appendix Figure Source Data [file 44318_2024_329_MOESM17_ESM.zip › SD Appendix/FigS5F/S5F/Morula_Trp2h_H3K4me3.jpg]

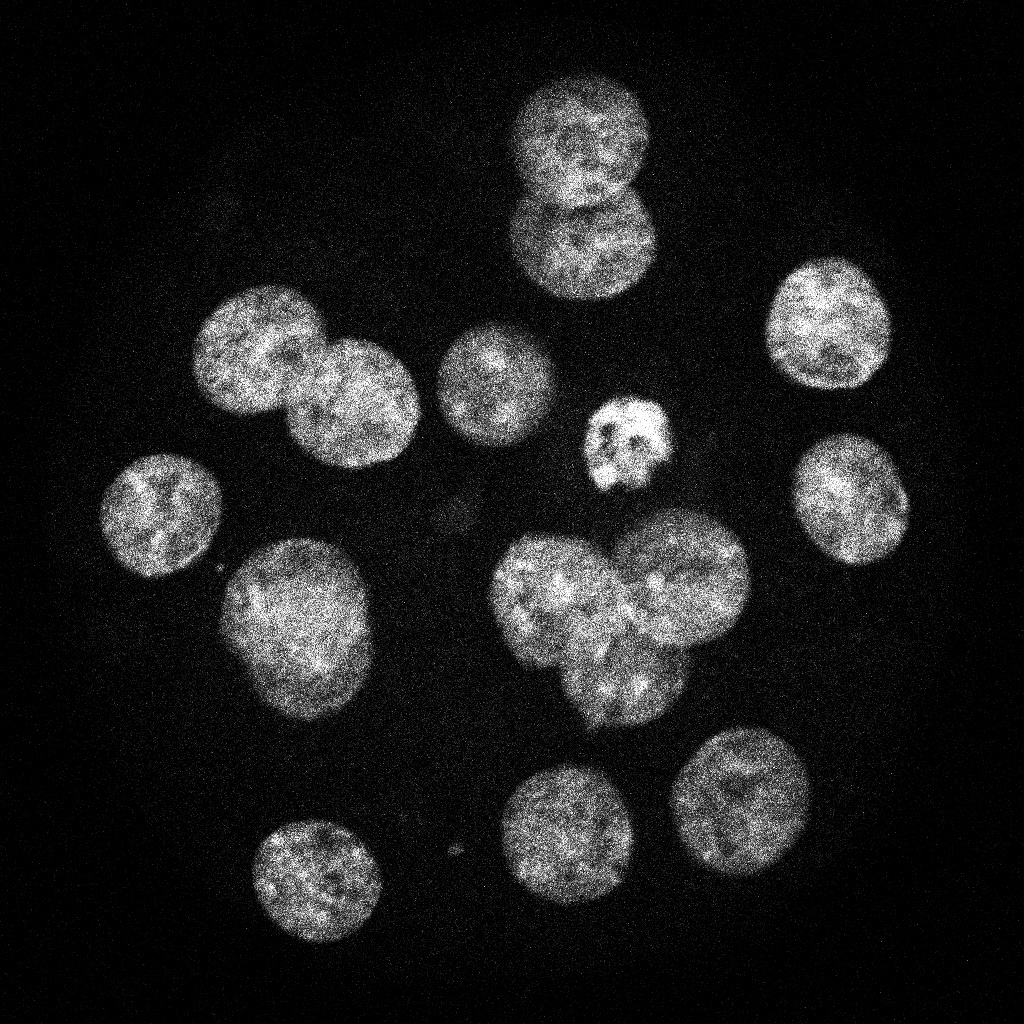

Supplement: Supplementary file 17 — Appendix Figure Source Data [file 44318_2024_329_MOESM17_ESM.zip › SD Appendix/FigS5F/S5F/Morula_Trp4h_DAPI.jpg]

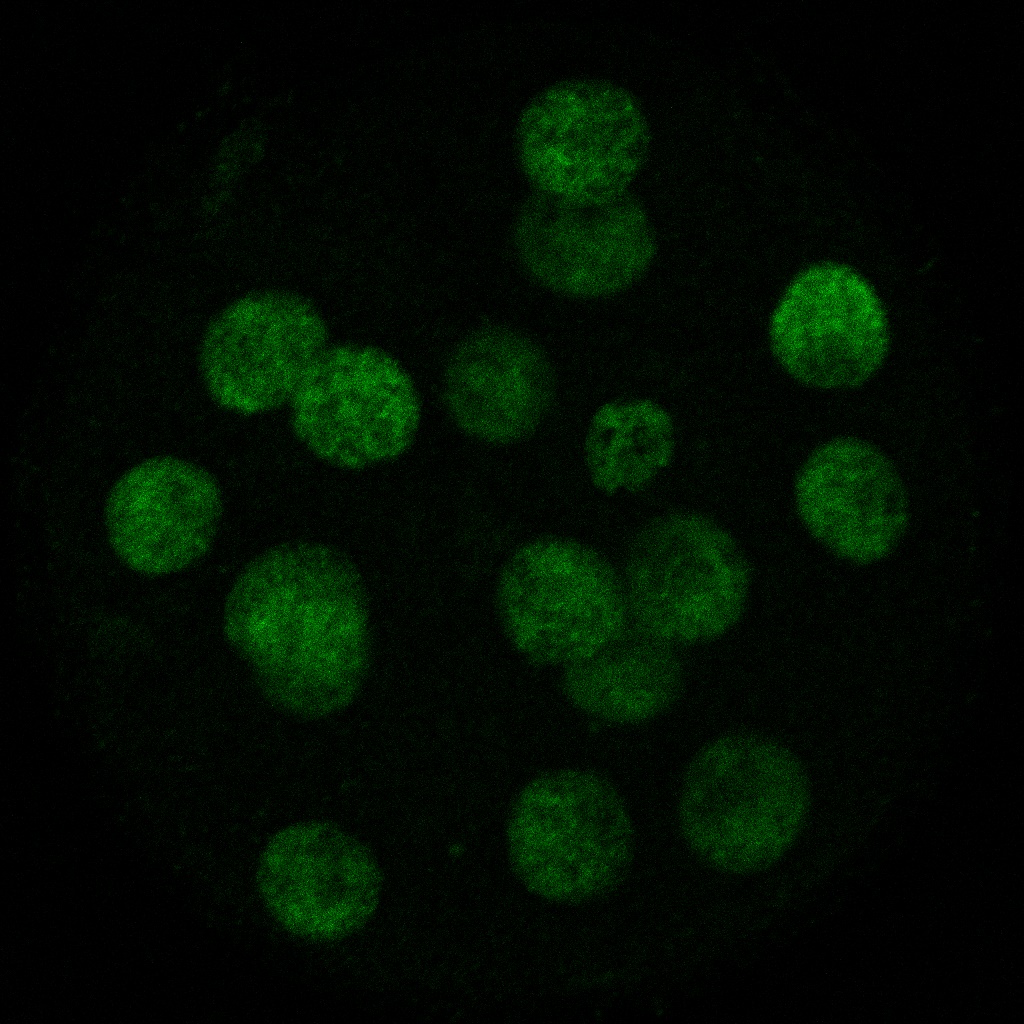

Supplement: Supplementary file 17 — Appendix Figure Source Data [file 44318_2024_329_MOESM17_ESM.zip › SD Appendix/FigS5F/S5F/Morula_Trp4h_H3K4me3.jpg]

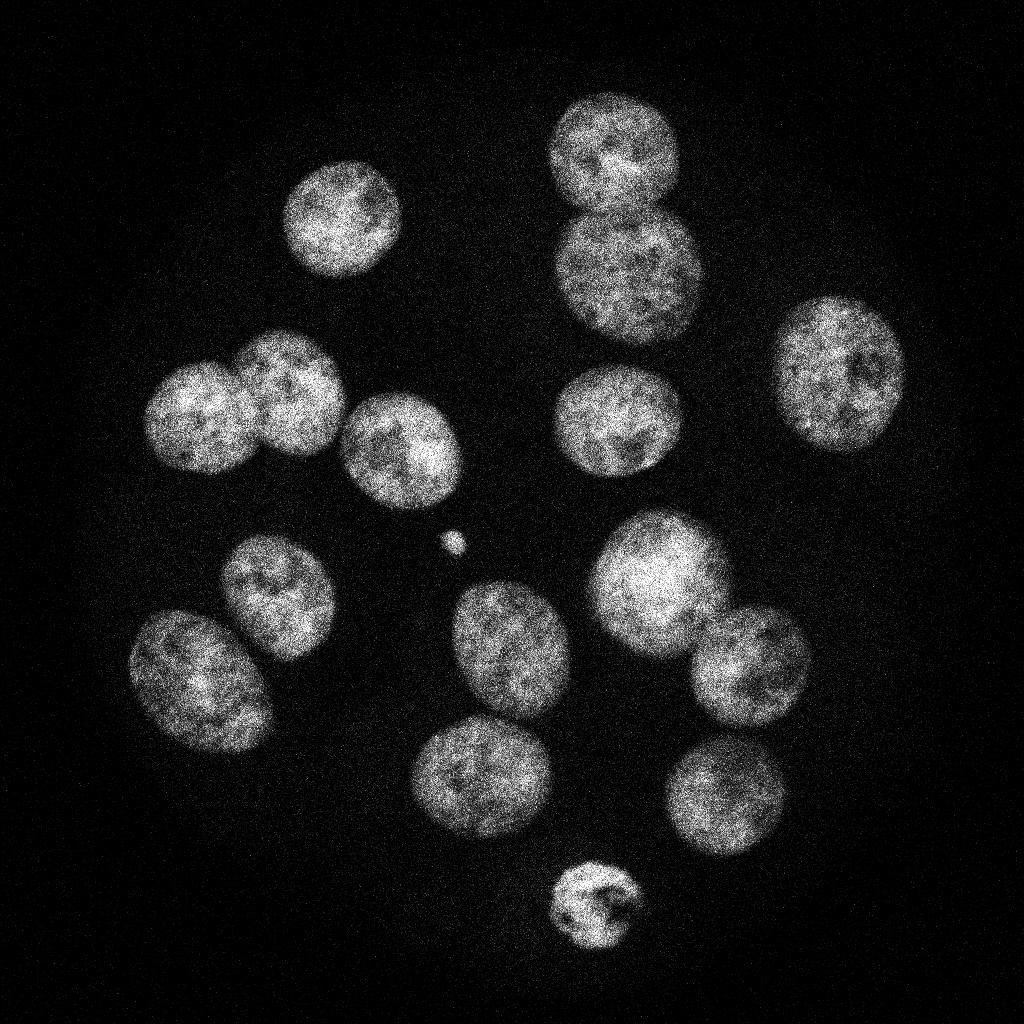

Supplement: Supplementary file 17 — Appendix Figure Source Data [file 44318_2024_329_MOESM17_ESM.zip › SD Appendix/FigS5F/S5F/Morula_Trp6h_DAPI.jpg]

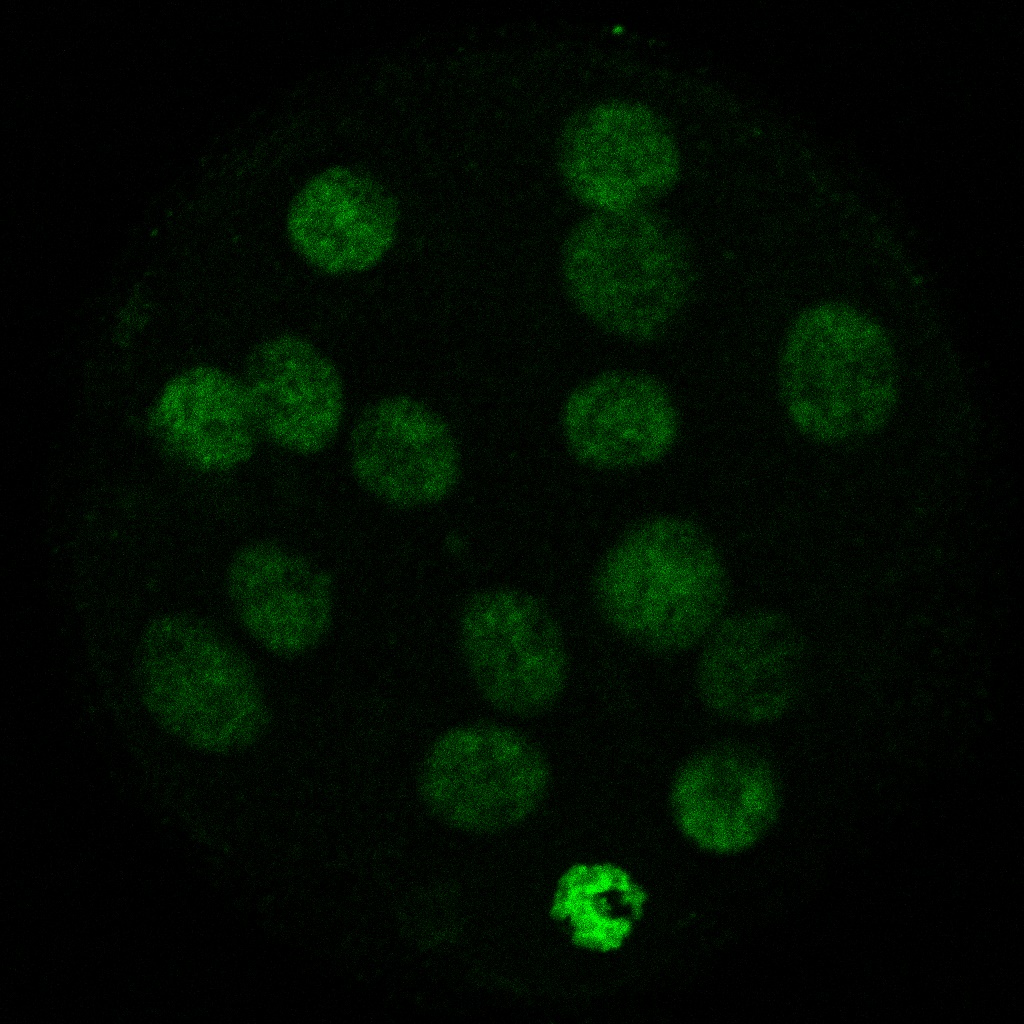

Supplement: Supplementary file 17 — Appendix Figure Source Data [file 44318_2024_329_MOESM17_ESM.zip › SD Appendix/FigS5F/S5F/Morula_Trp6h_H3K4me3.jpg]

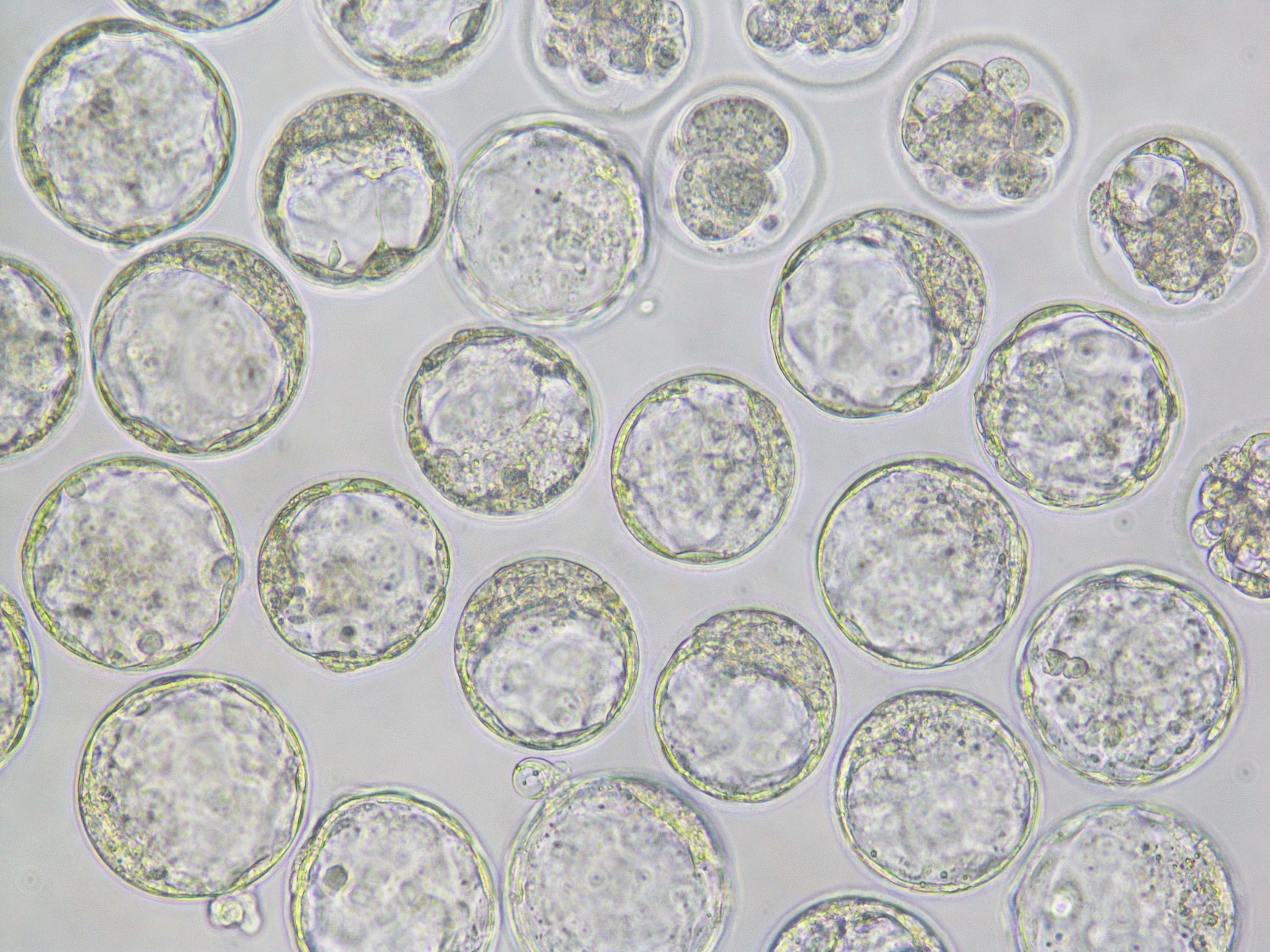

Supplement: Supplementary file 17 — Appendix Figure Source Data [file 44318_2024_329_MOESM17_ESM.zip › SD Appendix/FigS6G/Control.tif]

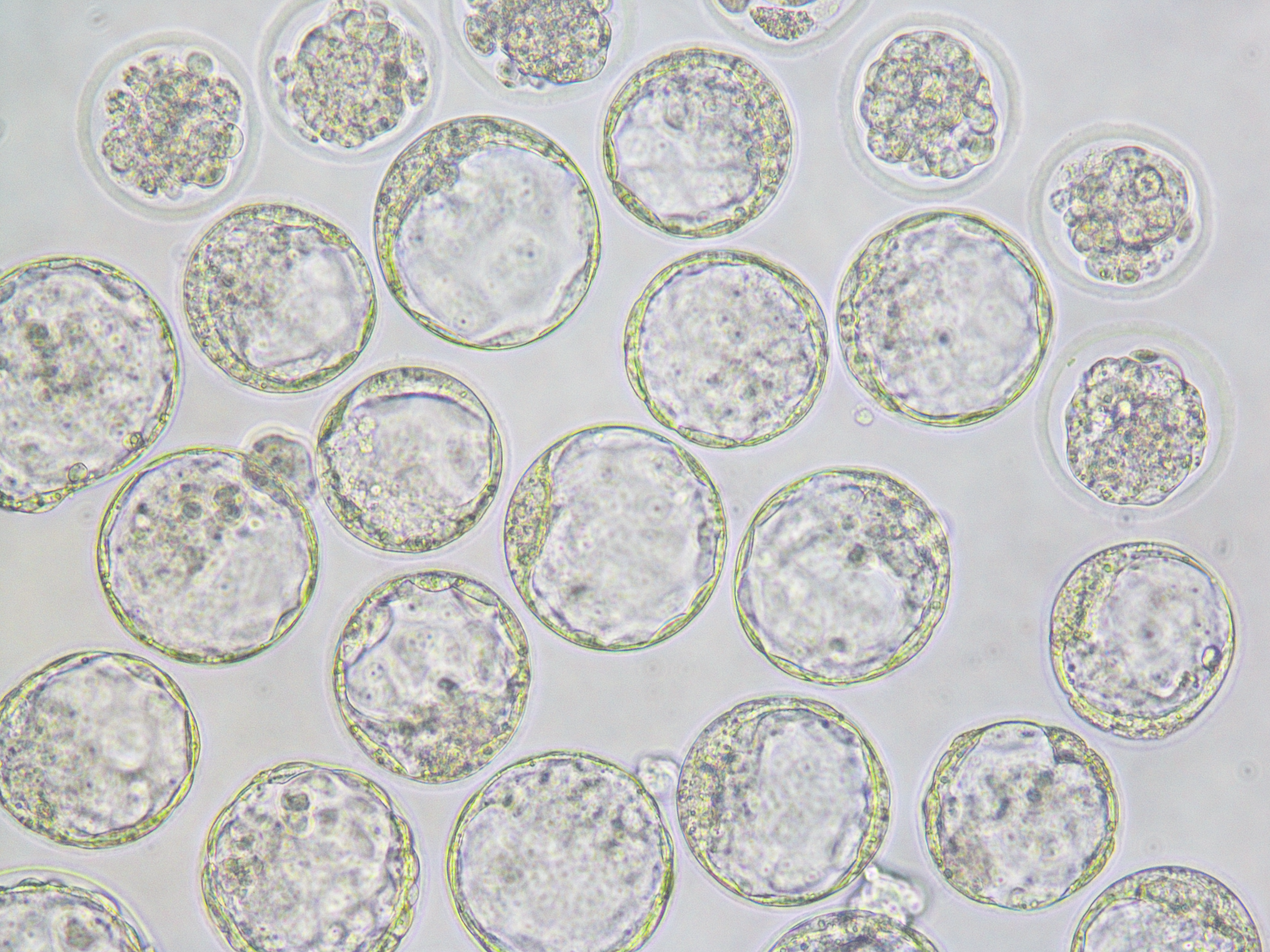

Supplement: Supplementary file 17 — Appendix Figure Source Data [file 44318_2024_329_MOESM17_ESM.zip › SD Appendix/FigS6G/Mll2 KD.tif]

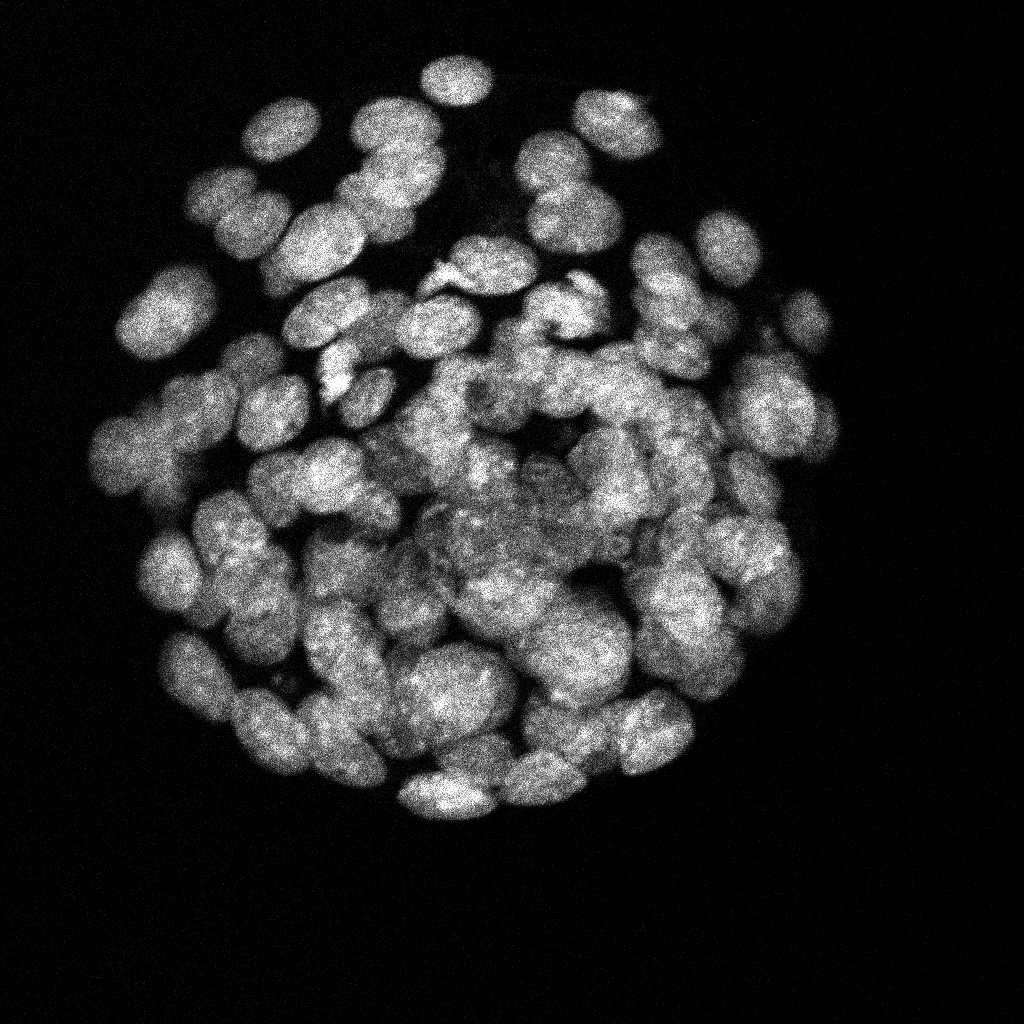

Supplement: Supplementary file 17 — Appendix Figure Source Data [file 44318_2024_329_MOESM17_ESM.zip › SD Appendix/FigS7C/S7C/Blastocyst_Control_DAPI.jpg]

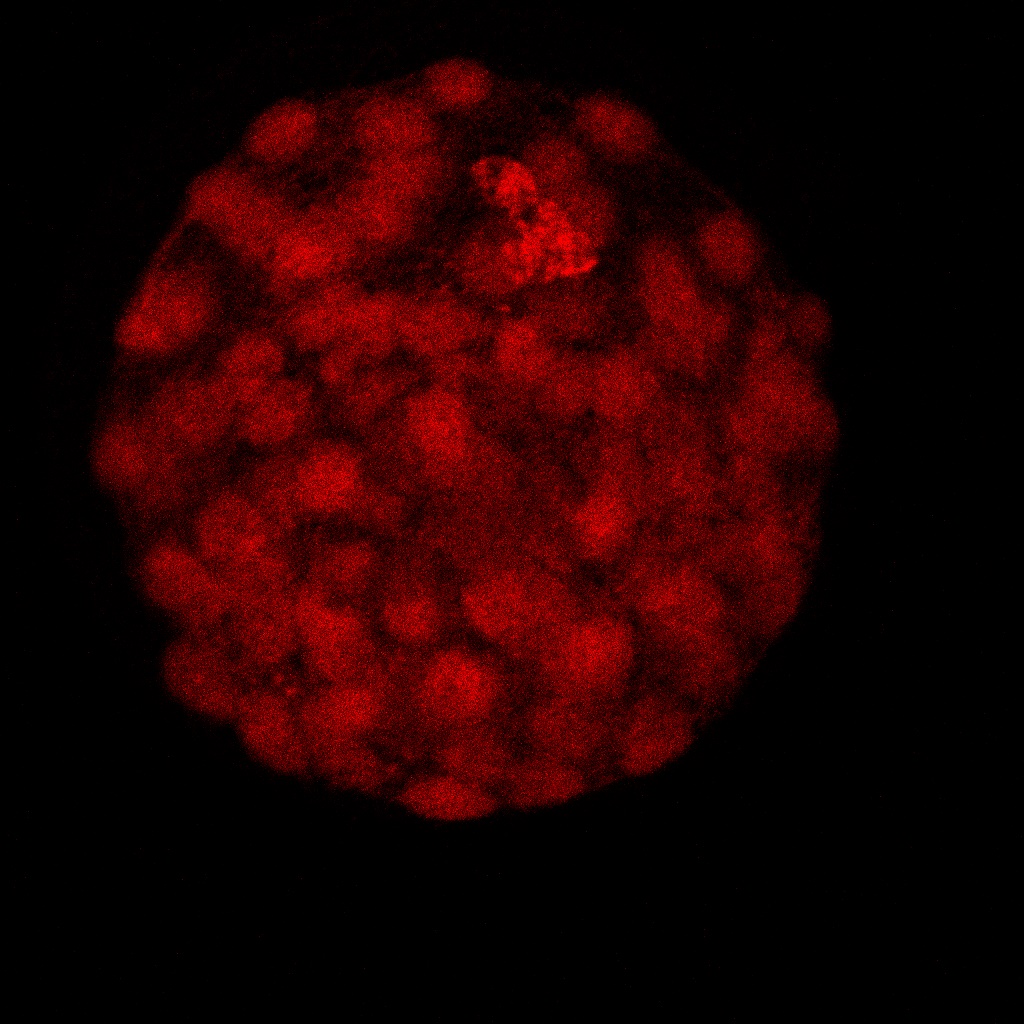

Supplement: Supplementary file 17 — Appendix Figure Source Data [file 44318_2024_329_MOESM17_ESM.zip › SD Appendix/FigS7C/S7C/Blastocyst_Control_MLL2.jpg]

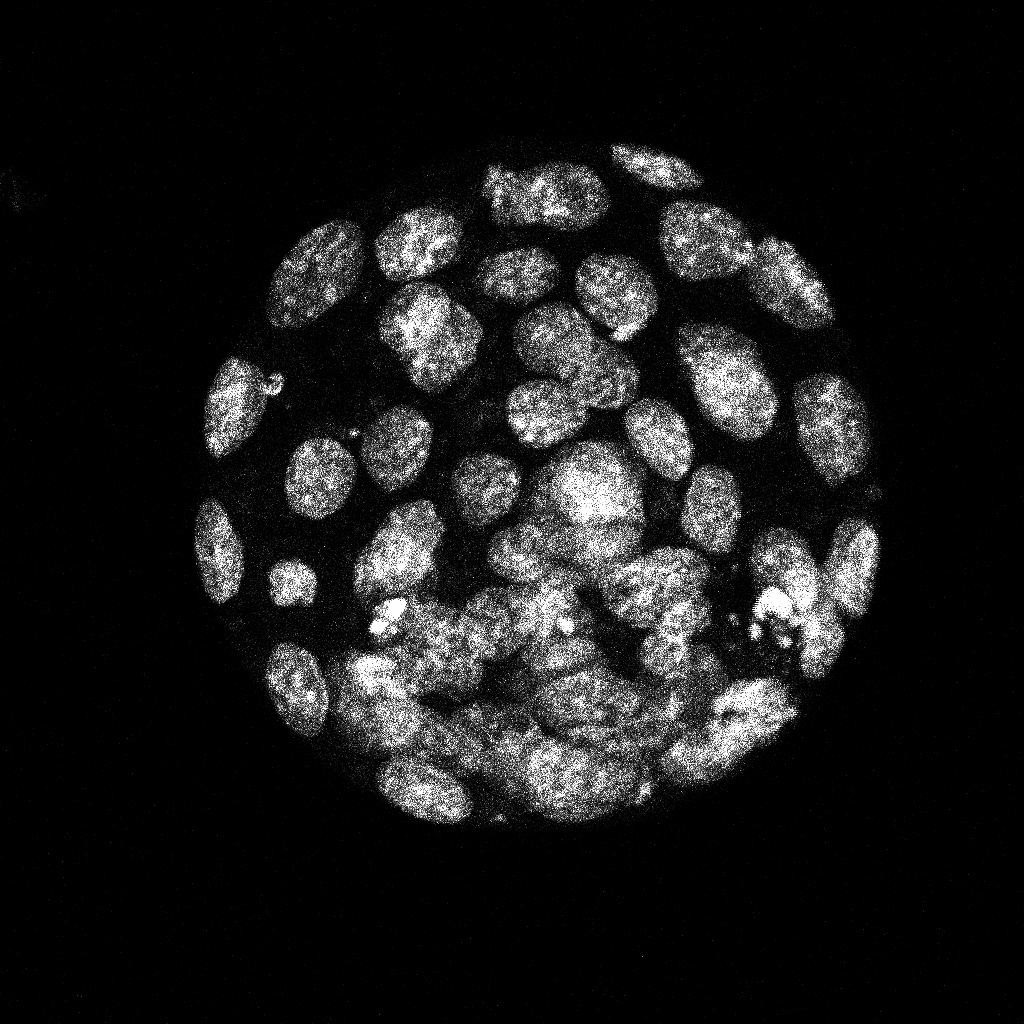

Supplement: Supplementary file 17 — Appendix Figure Source Data [file 44318_2024_329_MOESM17_ESM.zip › SD Appendix/FigS7C/S7C/Blastocyst_Mll2 KD2_DAPI.jpg]

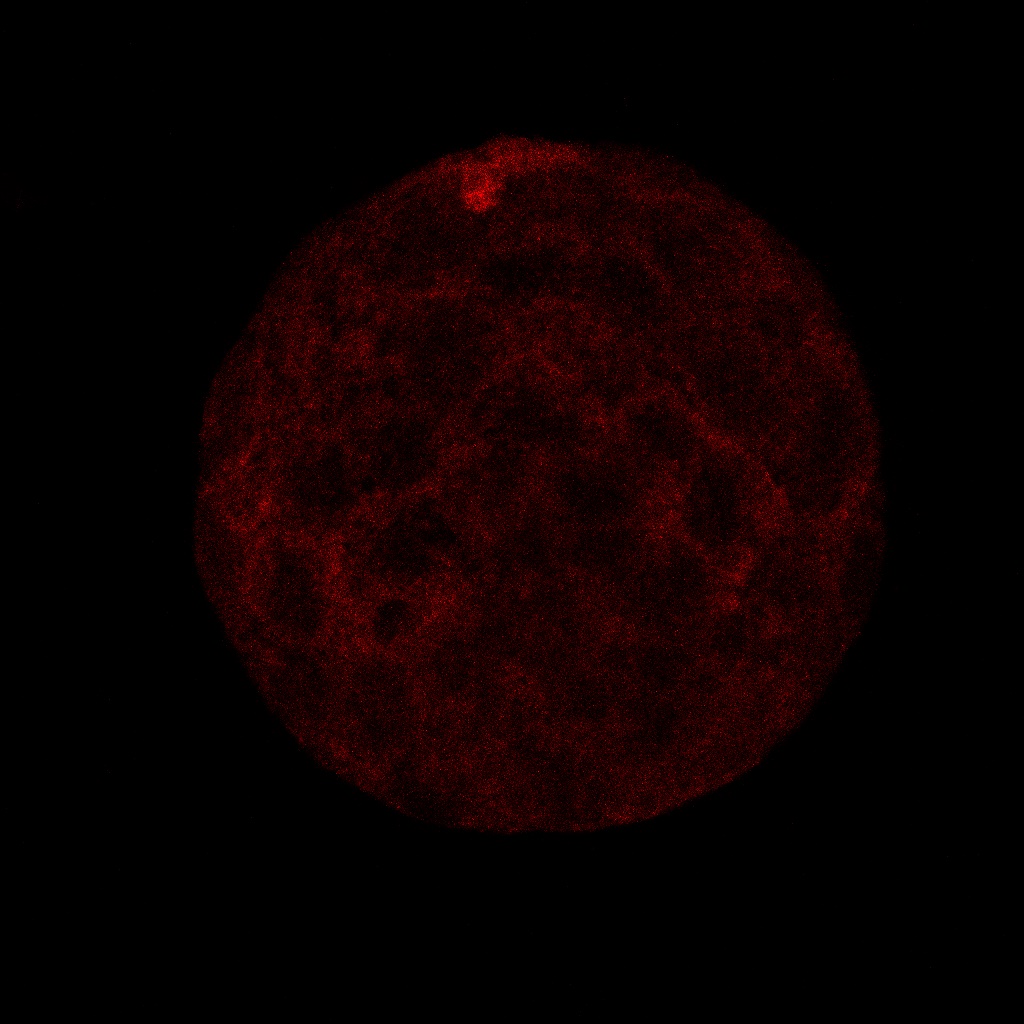

Supplement: Supplementary file 17 — Appendix Figure Source Data [file 44318_2024_329_MOESM17_ESM.zip › SD Appendix/FigS7C/S7C/Blastocyst_Mll2 KD2_MLL2.jpg]

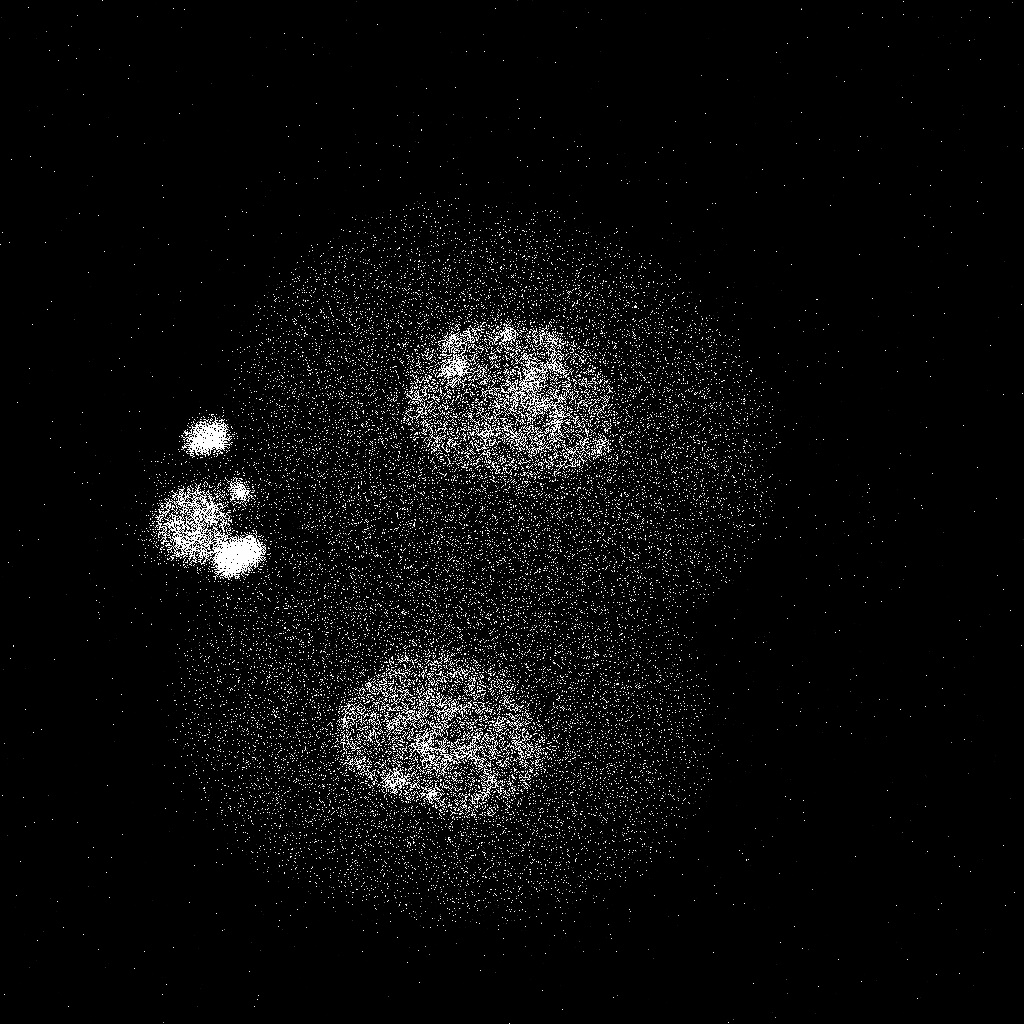

Supplement: Supplementary file 17 — Appendix Figure Source Data [file 44318_2024_329_MOESM17_ESM.zip › SD Appendix/FigS7C/S7C/Late2C_Control_DAPI.jpg]

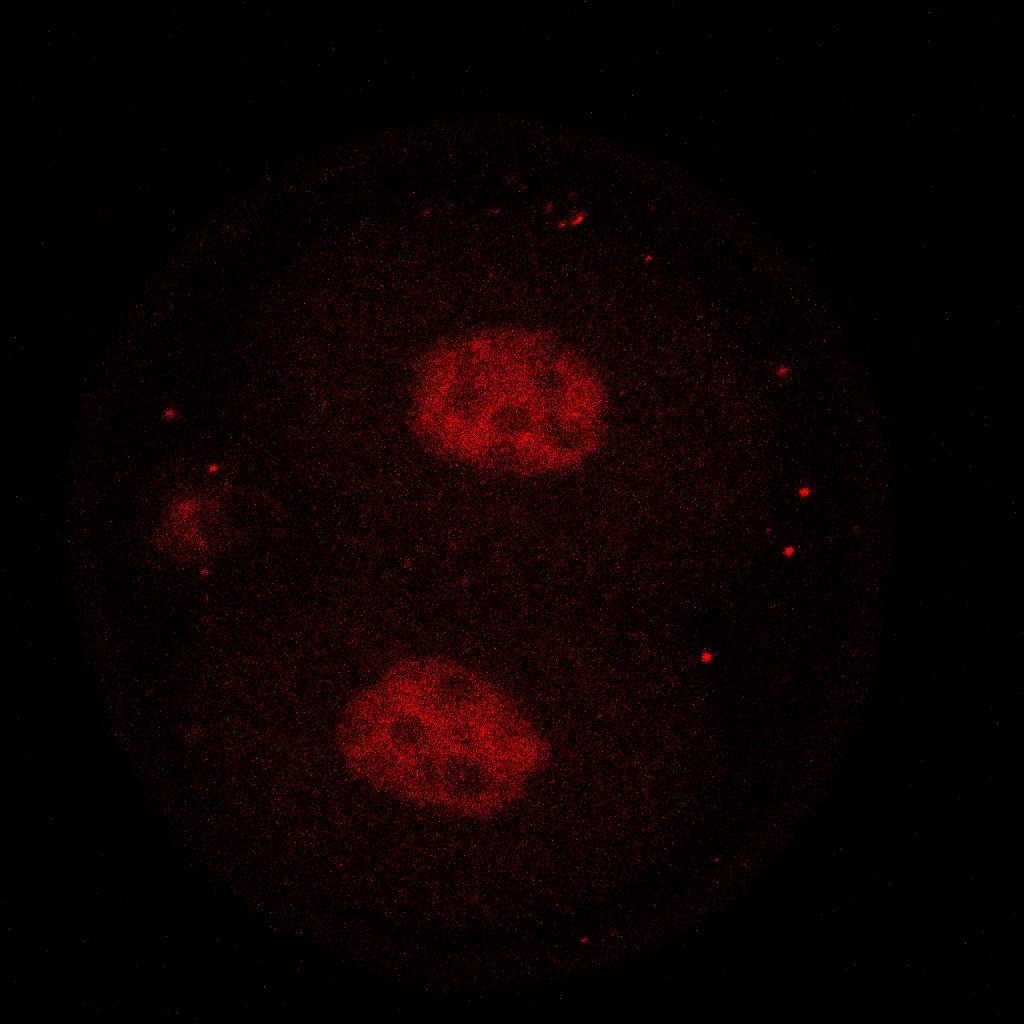

Supplement: Supplementary file 17 — Appendix Figure Source Data [file 44318_2024_329_MOESM17_ESM.zip › SD Appendix/FigS7C/S7C/Late2C_Control_MLL2.jpg]

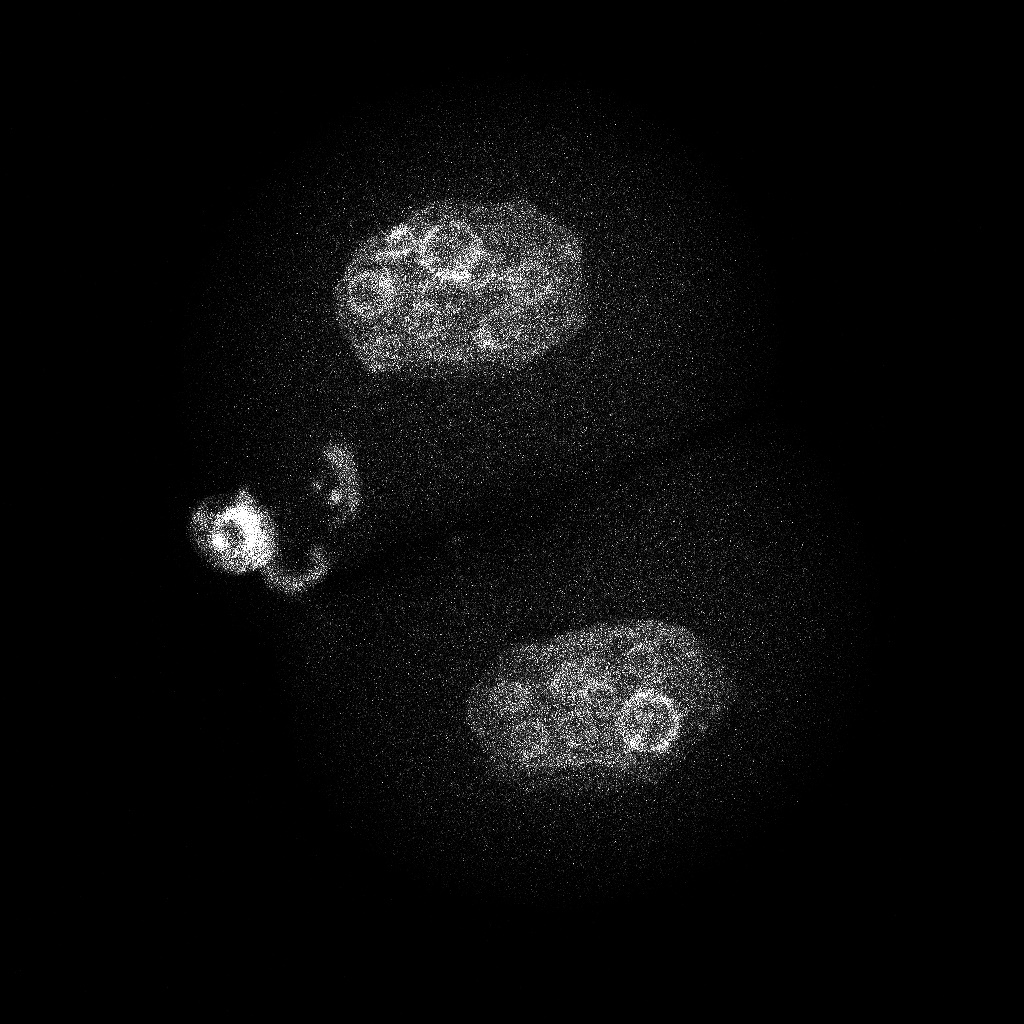

Supplement: Supplementary file 17 — Appendix Figure Source Data [file 44318_2024_329_MOESM17_ESM.zip › SD Appendix/FigS7C/S7C/Late2C_Mll2 KD2_DAPI.jpg]

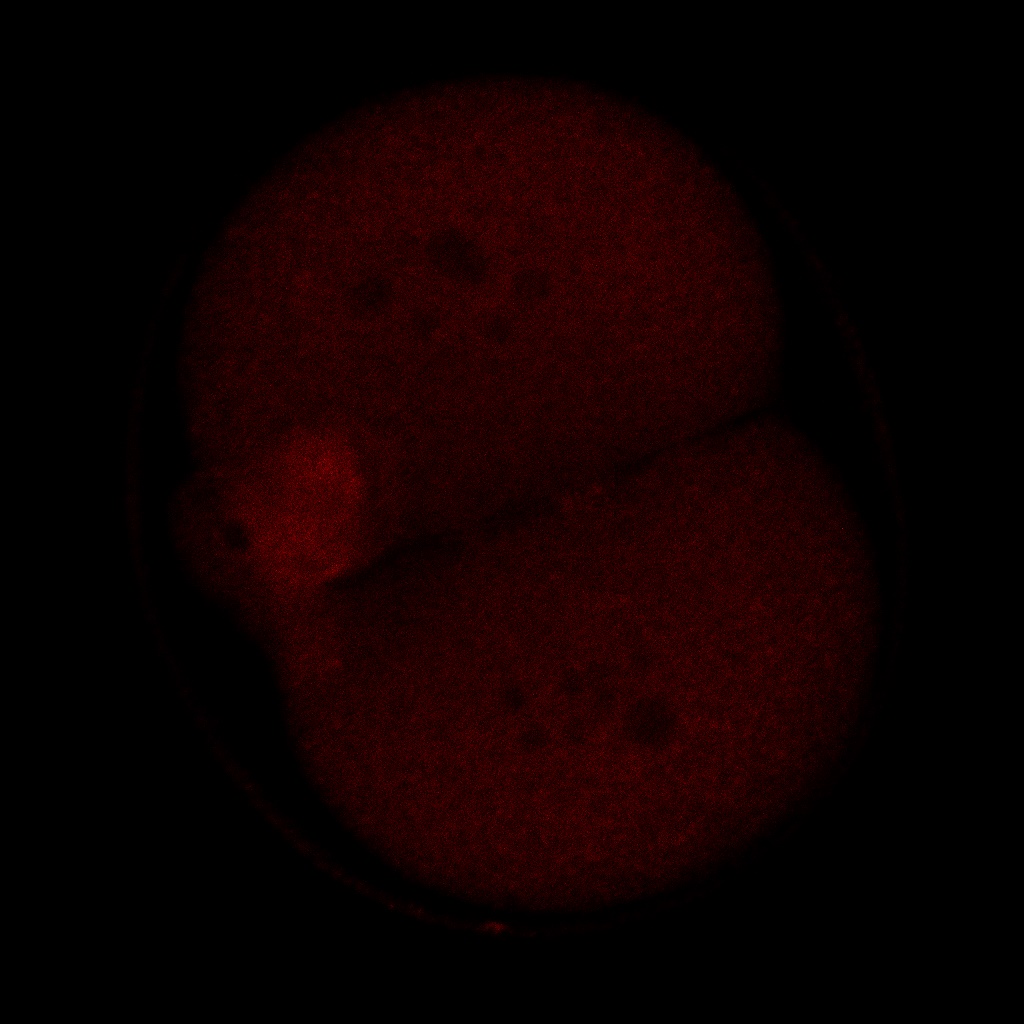

Supplement: Supplementary file 17 — Appendix Figure Source Data [file 44318_2024_329_MOESM17_ESM.zip › SD Appendix/FigS7C/S7C/Late2C_Mll2 KD2_MLL2.jpg]

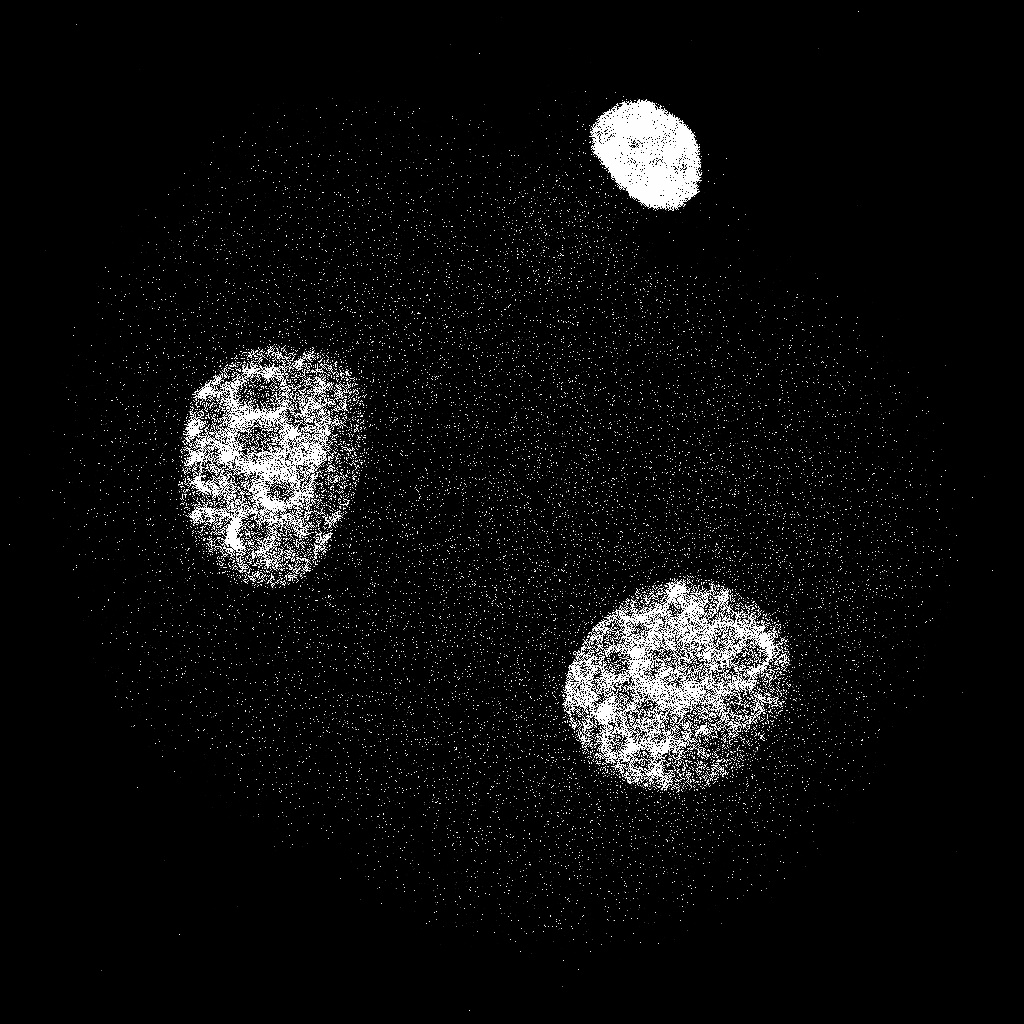

Supplement: Supplementary file 17 — Appendix Figure Source Data [file 44318_2024_329_MOESM17_ESM.zip › SD Appendix/FigS7D/S7D/Late2C_Control_DAPI.jpg]

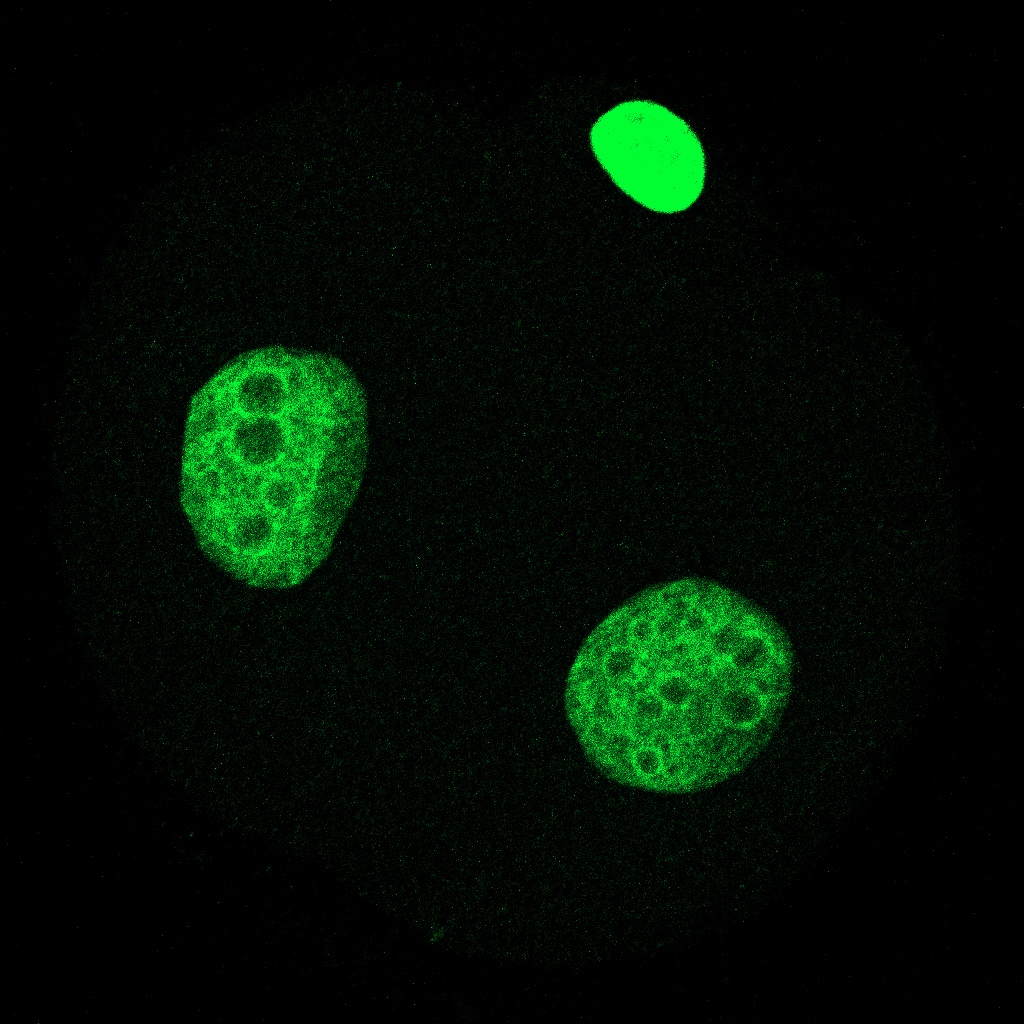

Supplement: Supplementary file 17 — Appendix Figure Source Data [file 44318_2024_329_MOESM17_ESM.zip › SD Appendix/FigS7D/S7D/Late2C_Control_H3K4me3.jpg]

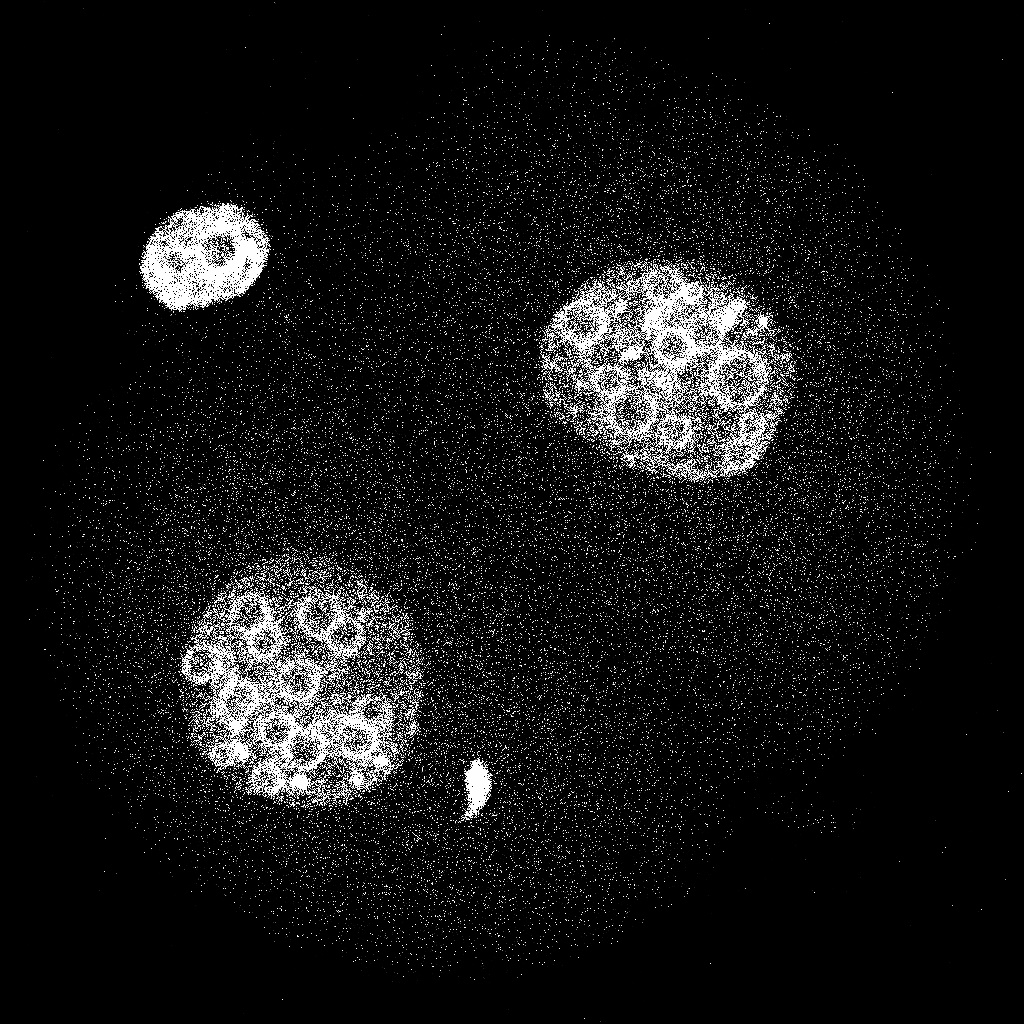

Supplement: Supplementary file 17 — Appendix Figure Source Data [file 44318_2024_329_MOESM17_ESM.zip › SD Appendix/FigS7D/S7D/Late2C_Mll2 KD2_DAPI.jpg]

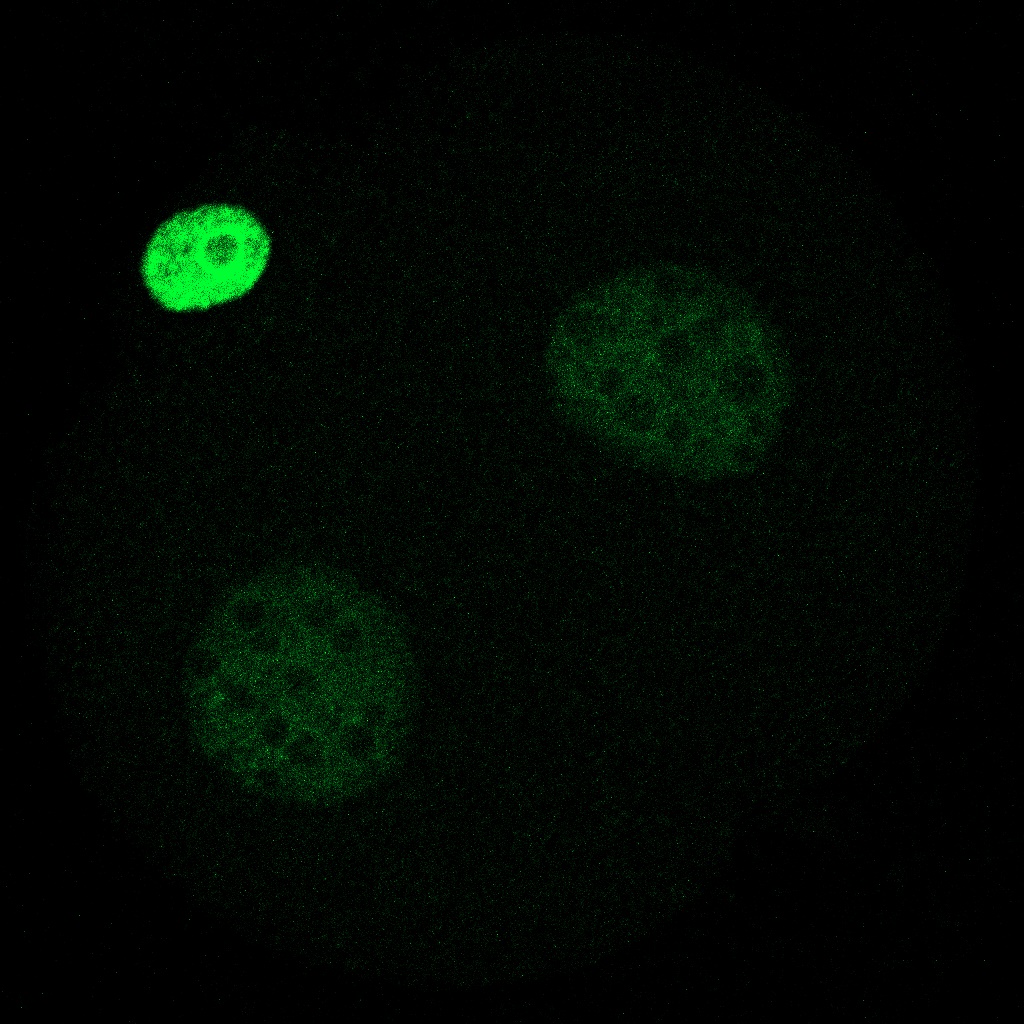

Supplement: Supplementary file 17 — Appendix Figure Source Data [file 44318_2024_329_MOESM17_ESM.zip › SD Appendix/FigS7D/S7D/Late2C_Mll2 KD2_H3K4me3.jpg]

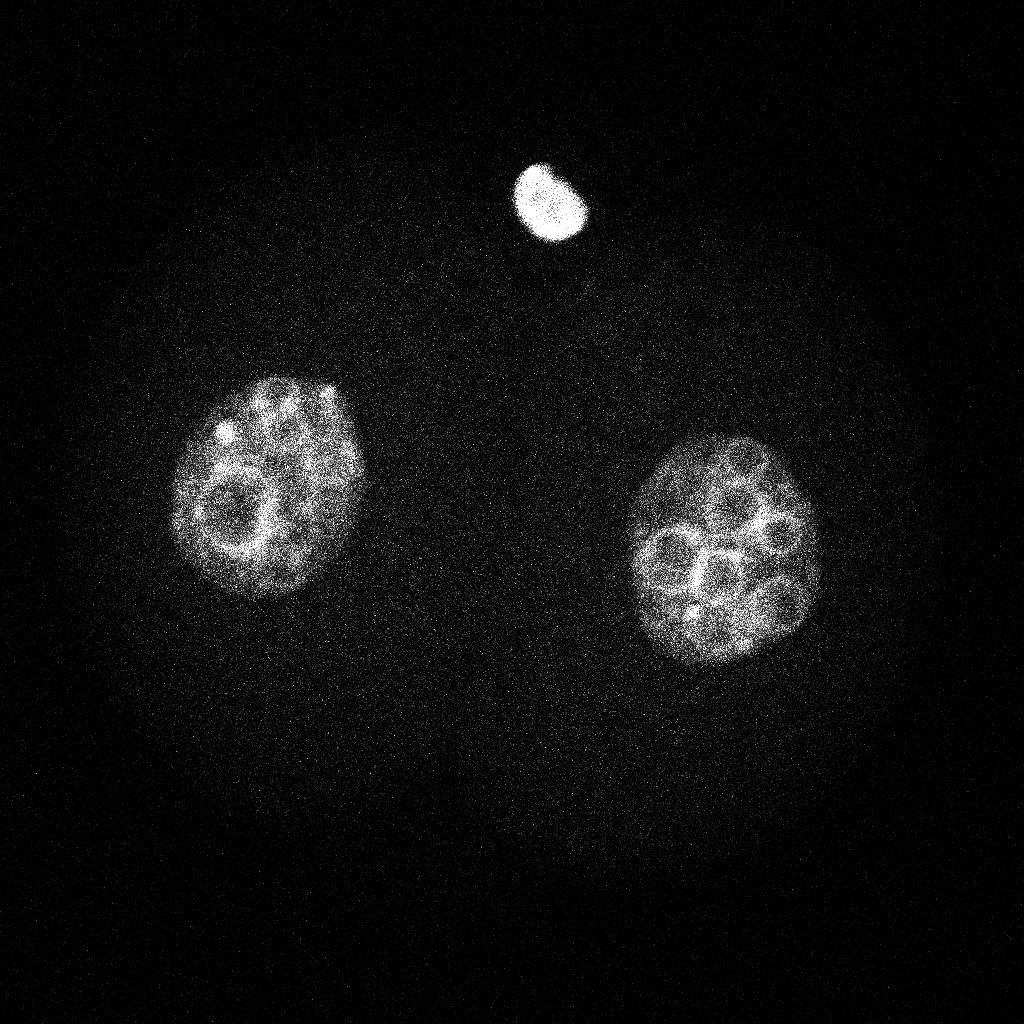

Supplement: Supplementary file 17 — Appendix Figure Source Data [file 44318_2024_329_MOESM17_ESM.zip › SD Appendix/FigS7E/S7E/Late2C_Control_DAPI.jpg]

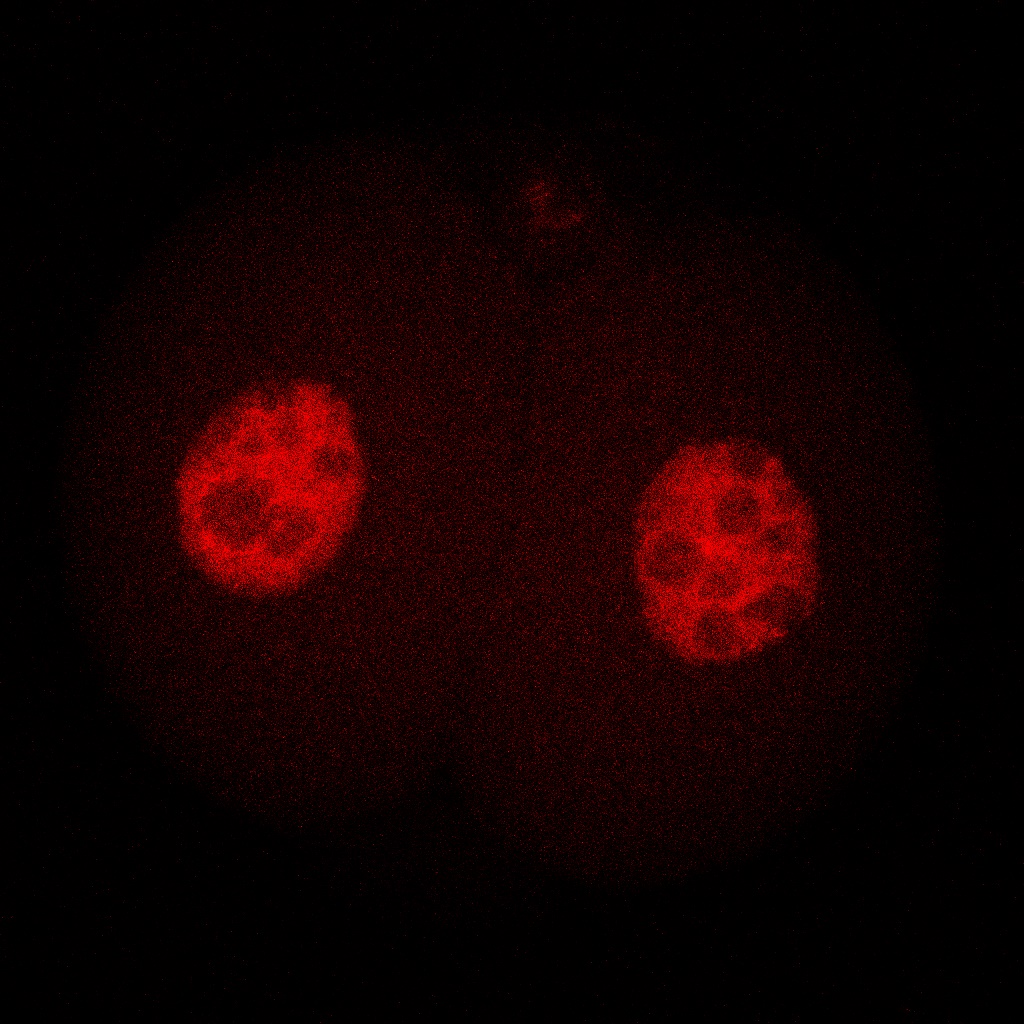

Supplement: Supplementary file 17 — Appendix Figure Source Data [file 44318_2024_329_MOESM17_ESM.zip › SD Appendix/FigS7E/S7E/Late2C_Control_EU.jpg]

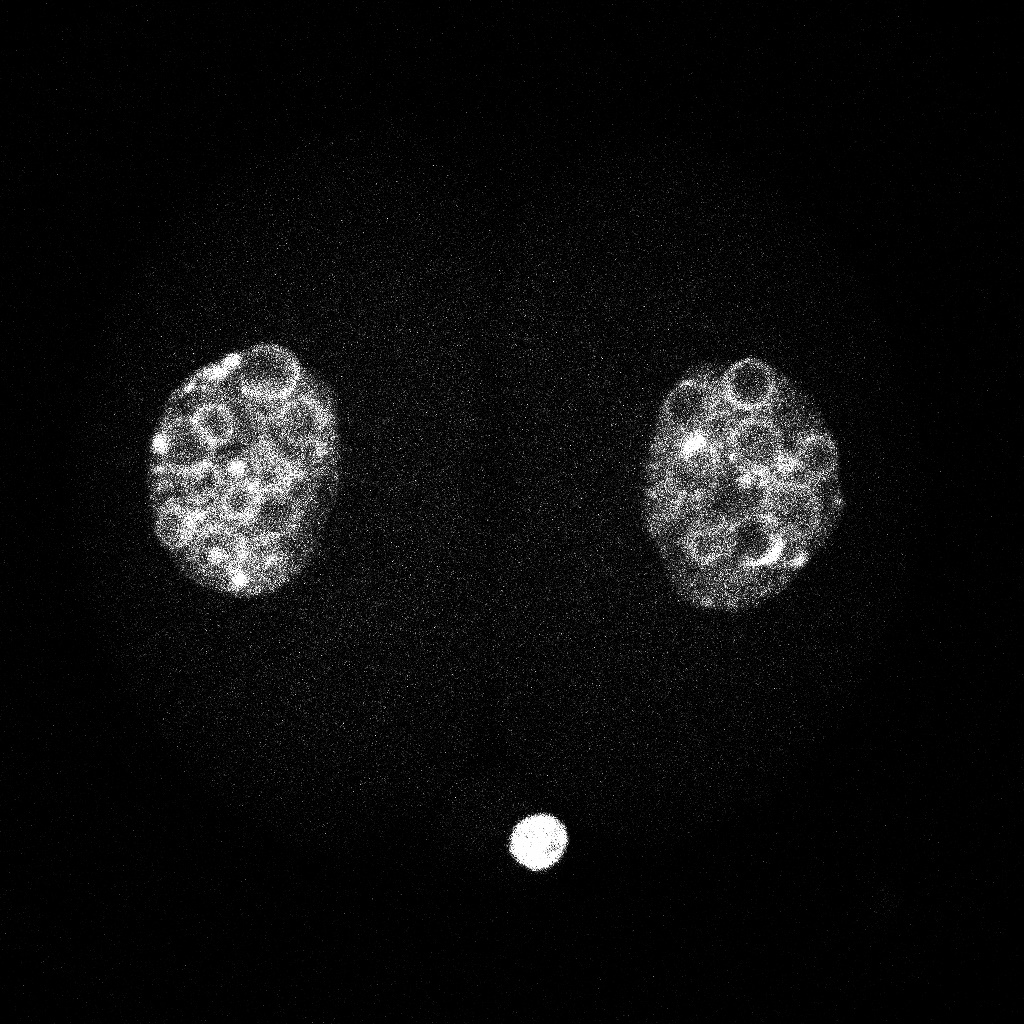

Supplement: Supplementary file 17 — Appendix Figure Source Data [file 44318_2024_329_MOESM17_ESM.zip › SD Appendix/FigS7E/S7E/Late2C_Mll2 KD2_DAPI.jpg]

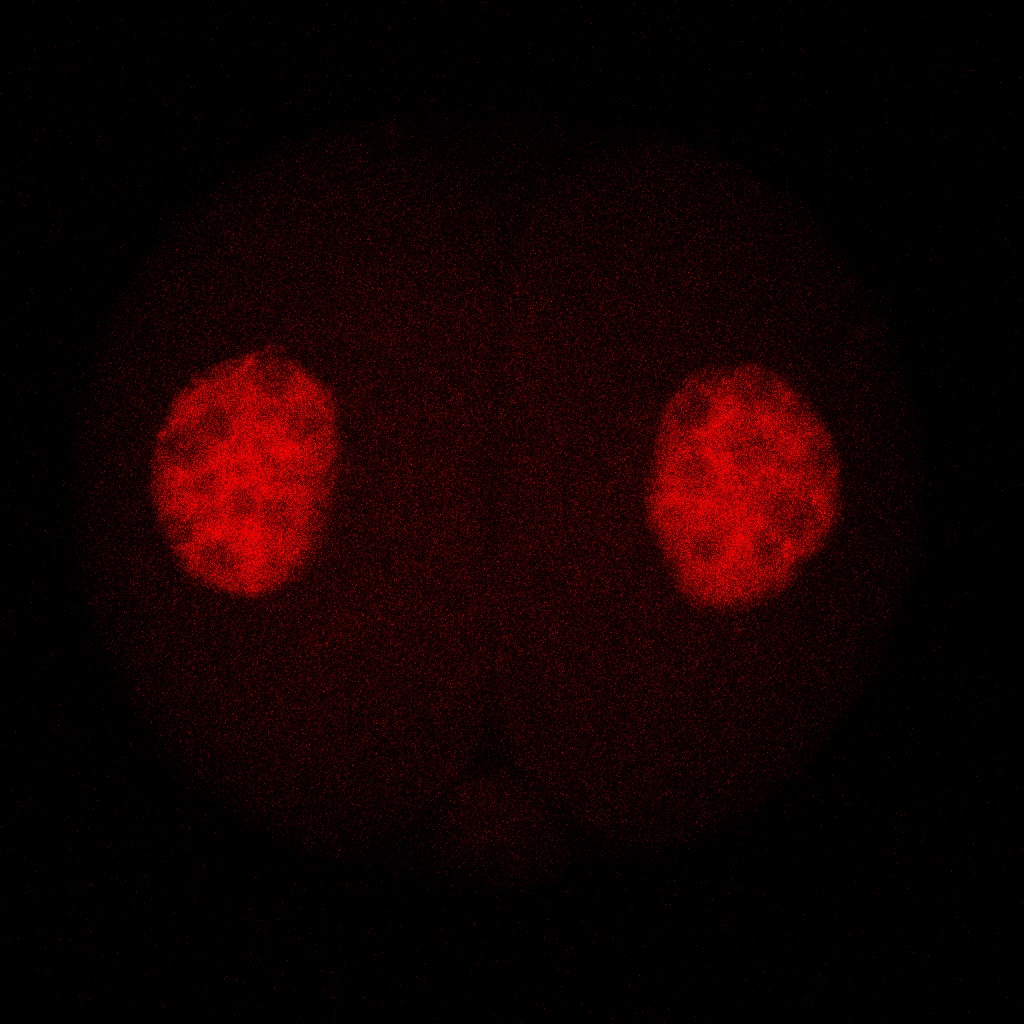

Supplement: Supplementary file 17 — Appendix Figure Source Data [file 44318_2024_329_MOESM17_ESM.zip › SD Appendix/FigS7E/S7E/Late2C_Mll2 KD2_EU.jpg]

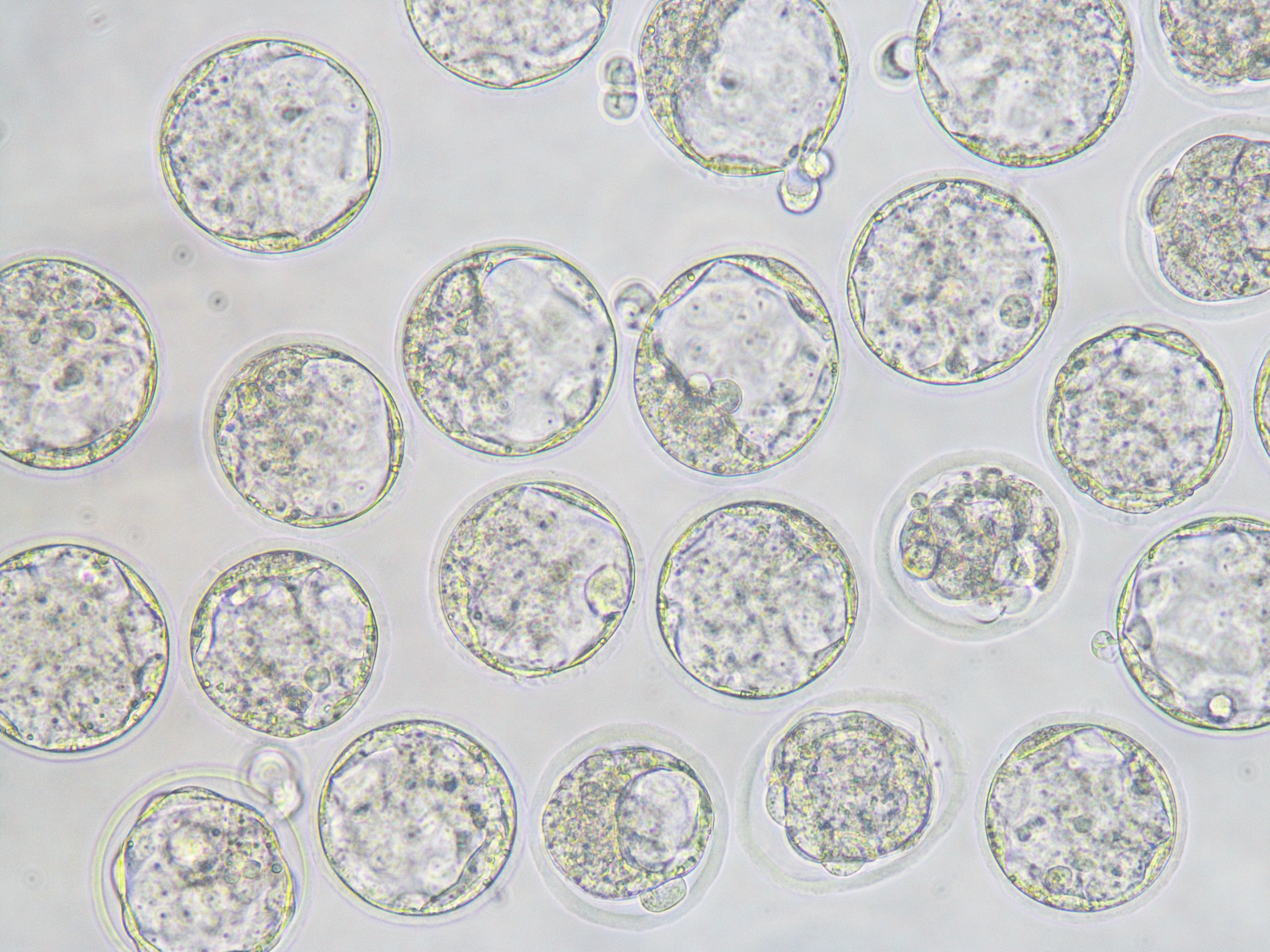

Supplement: Supplementary file 17 — Appendix Figure Source Data [file 44318_2024_329_MOESM17_ESM.zip › SD Appendix/FigS7G/Control.tif]

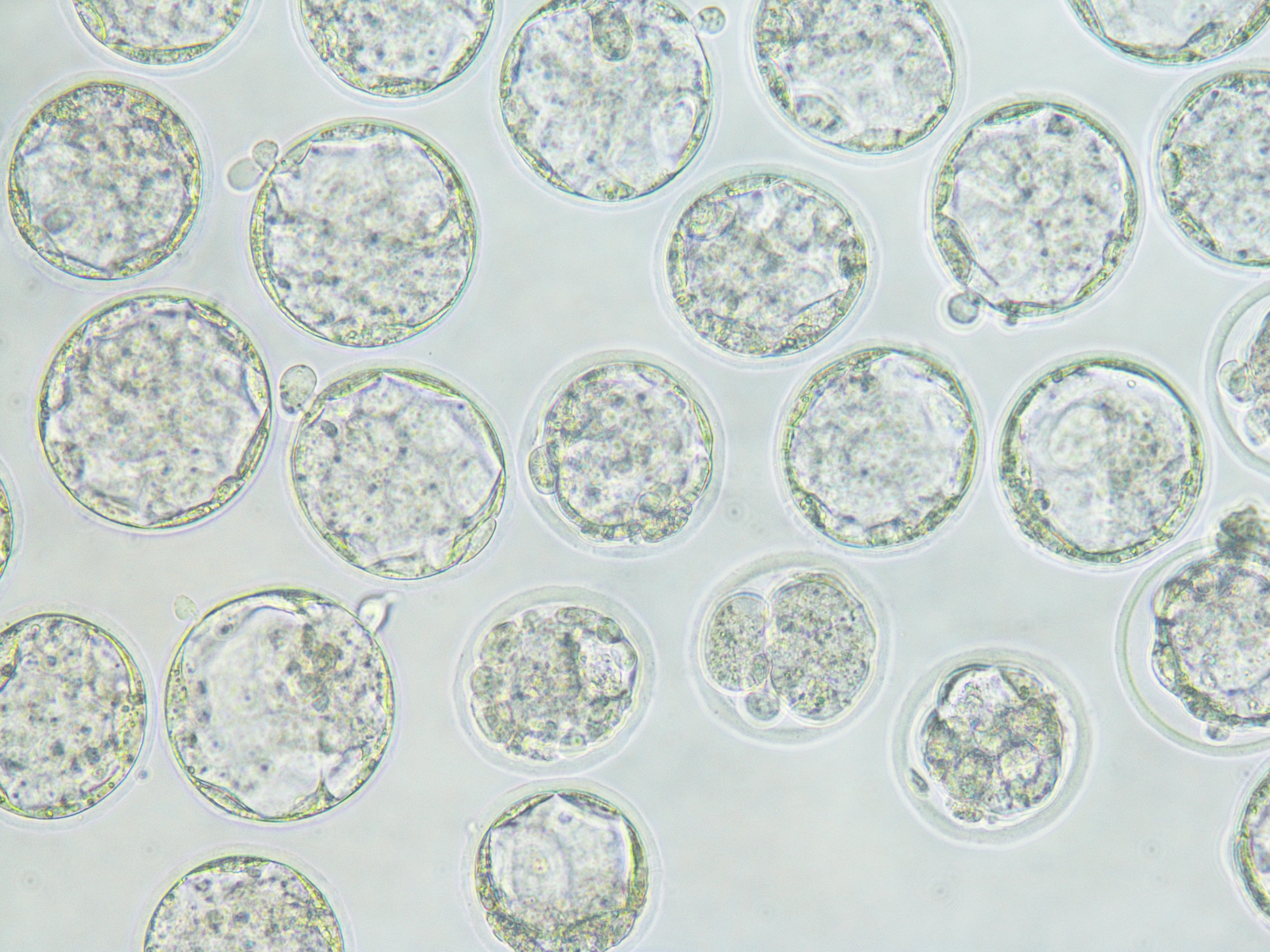

Supplement: Supplementary file 17 — Appendix Figure Source Data [file 44318_2024_329_MOESM17_ESM.zip › SD Appendix/FigS7G/Mll2 KD-2.tif]

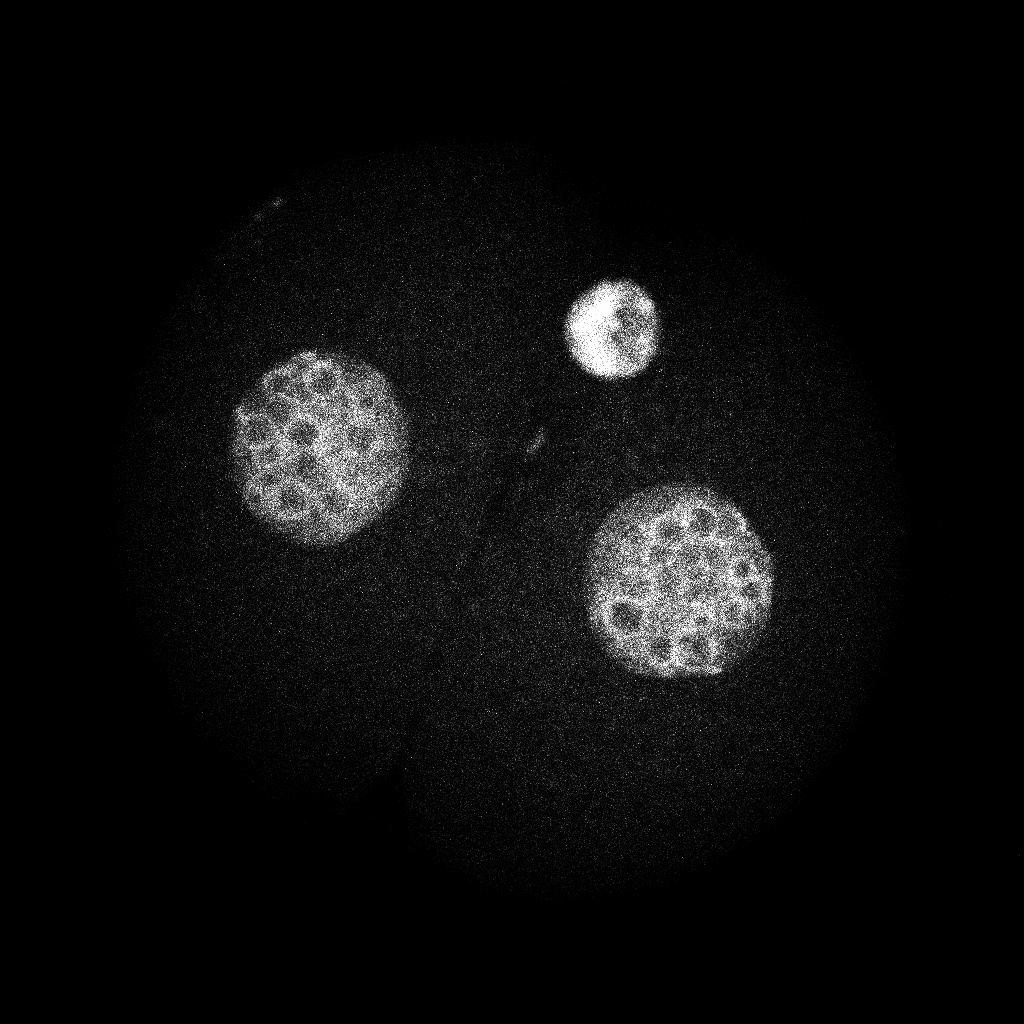

Supplement: Supplementary file 17 — Appendix Figure Source Data [file 44318_2024_329_MOESM17_ESM.zip › SD Appendix/FigS8A/S8A/Early2C_Control_DAPI.jpg]

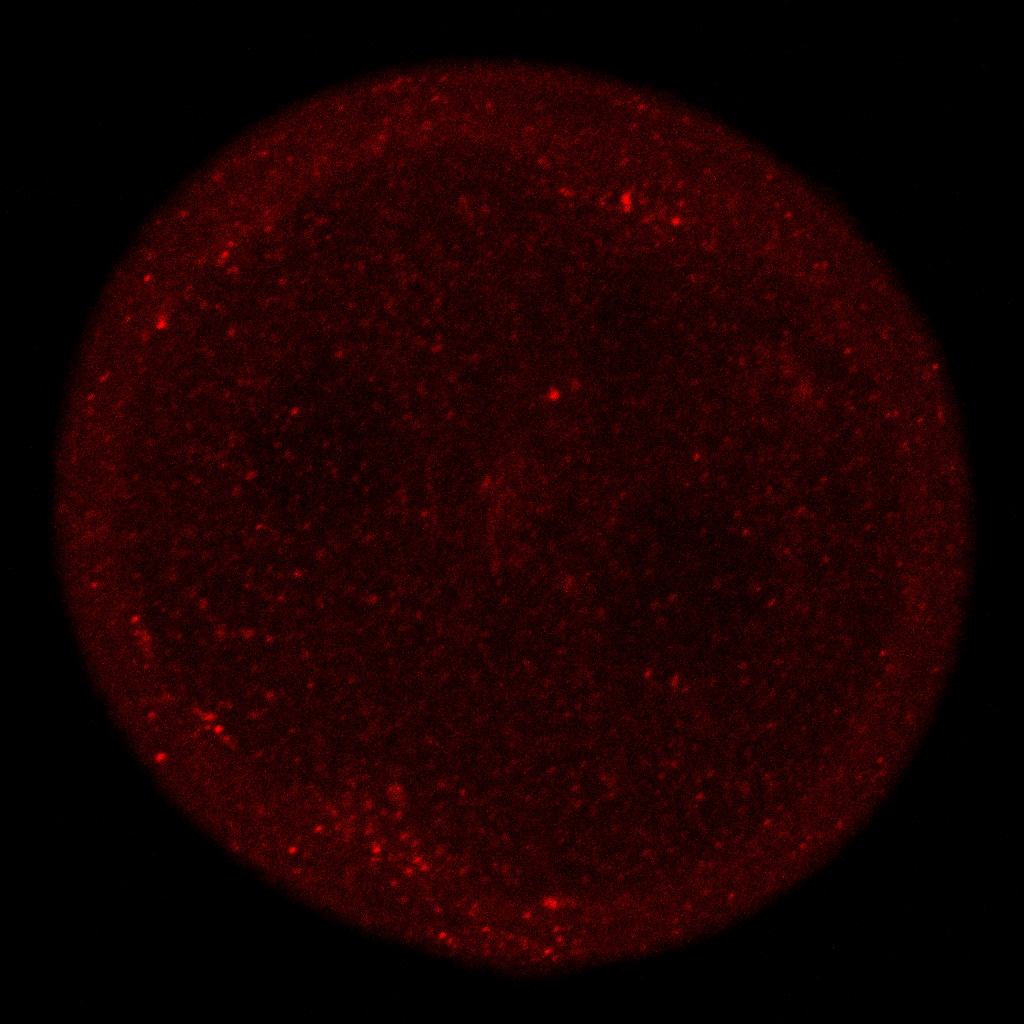

Supplement: Supplementary file 17 — Appendix Figure Source Data [file 44318_2024_329_MOESM17_ESM.zip › SD Appendix/FigS8A/S8A/Early2C_Control_KDM5B.jpg]

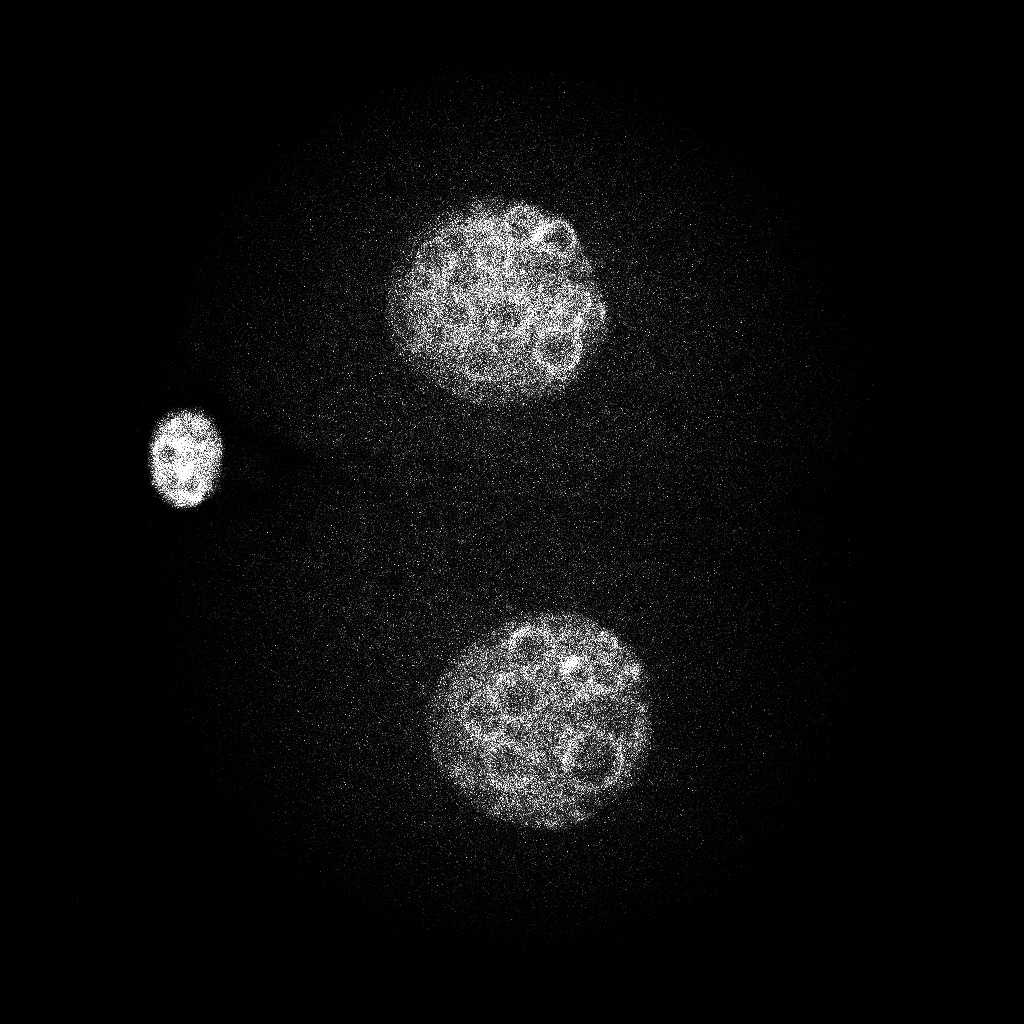

Supplement: Supplementary file 17 — Appendix Figure Source Data [file 44318_2024_329_MOESM17_ESM.zip › SD Appendix/FigS8A/S8A/Early2C_OE Kdm5B mut_DAPI.jpg]

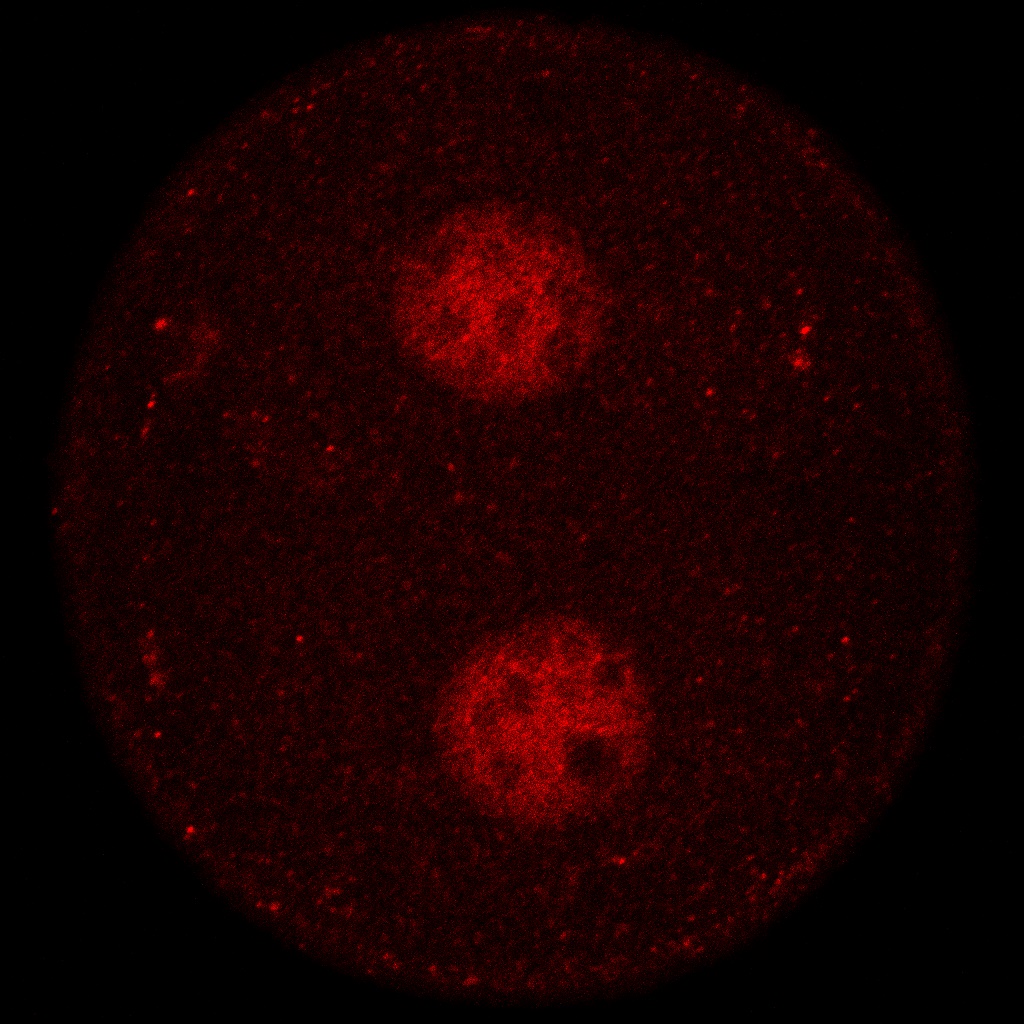

Supplement: Supplementary file 17 — Appendix Figure Source Data [file 44318_2024_329_MOESM17_ESM.zip › SD Appendix/FigS8A/S8A/Early2C_OE Kdm5B mut_KDM5B.jpg]

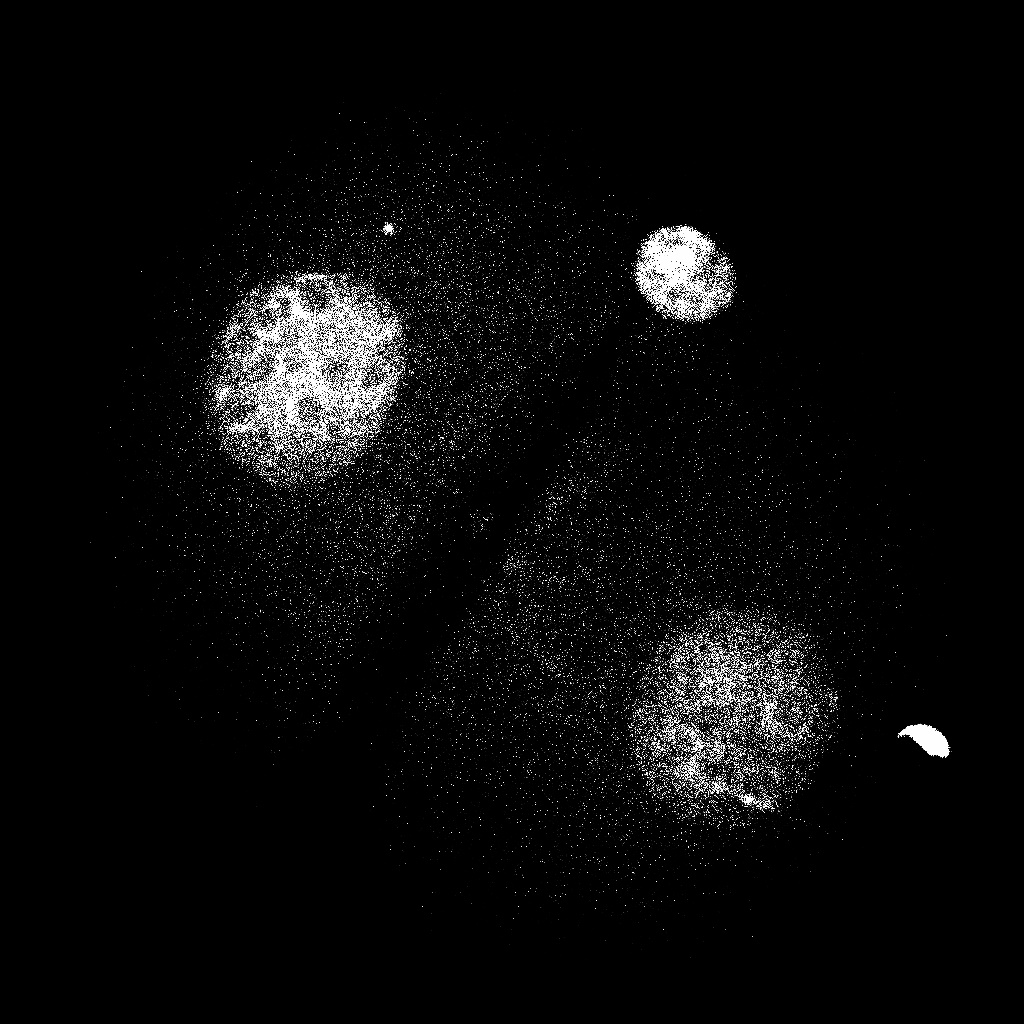

Supplement: Supplementary file 17 — Appendix Figure Source Data [file 44318_2024_329_MOESM17_ESM.zip › SD Appendix/FigS8A/S8A/Early2C_OE Kdm5b WT_DAPI.jpg]

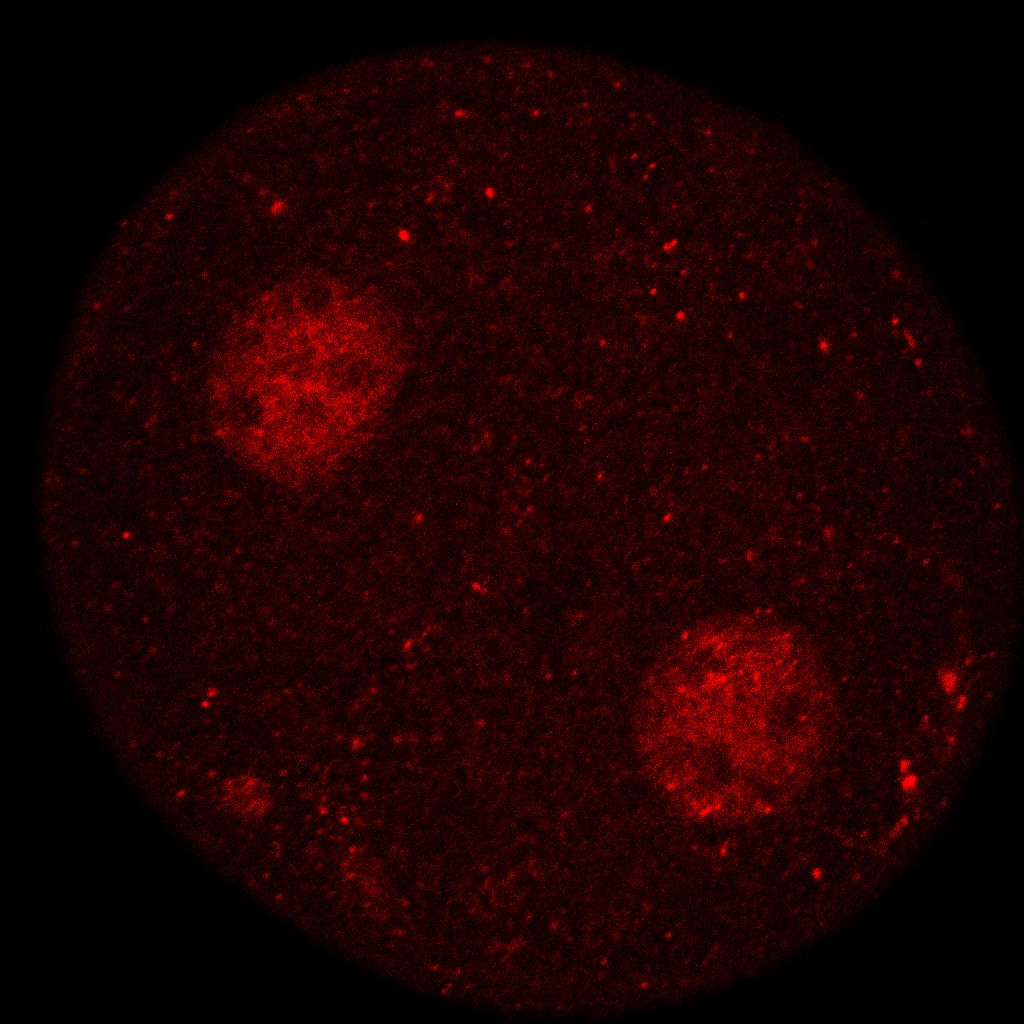

Supplement: Supplementary file 17 — Appendix Figure Source Data [file 44318_2024_329_MOESM17_ESM.zip › SD Appendix/FigS8A/S8A/Early2C_OE Kdm5b WT_KDM5B.jpg]

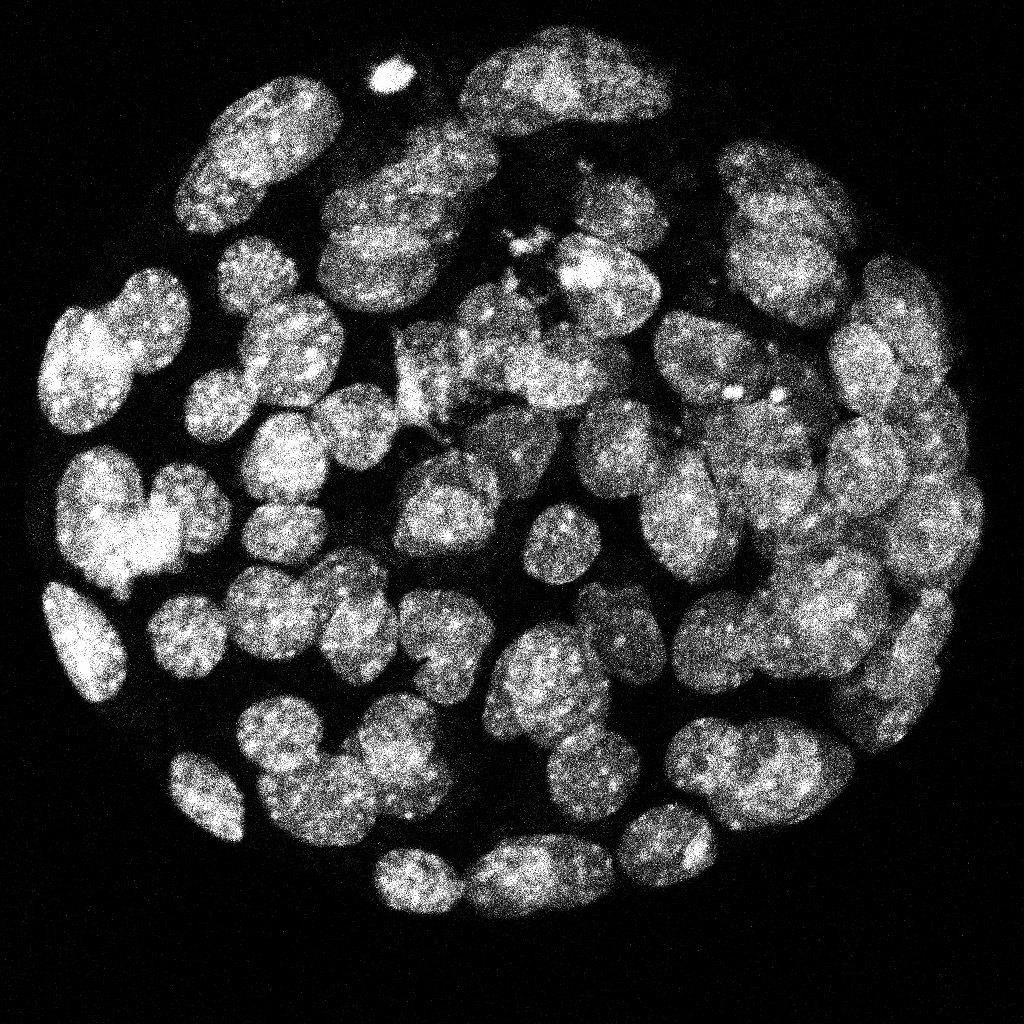

Supplement: Supplementary file 17 — Appendix Figure Source Data [file 44318_2024_329_MOESM17_ESM.zip › SD Appendix/FigS9B/S9B/Blastocyst_Control_DAPI.jpg]

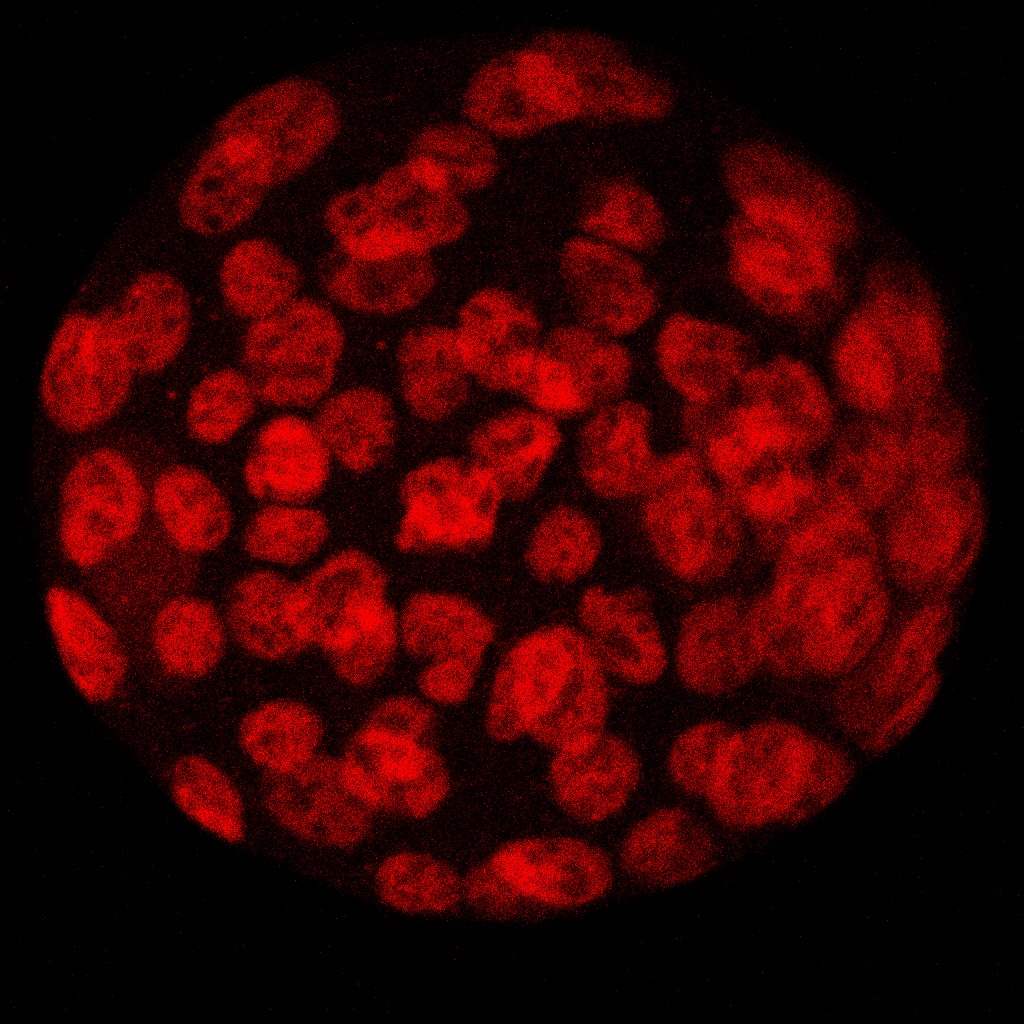

Supplement: Supplementary file 17 — Appendix Figure Source Data [file 44318_2024_329_MOESM17_ESM.zip › SD Appendix/FigS9B/S9B/Blastocyst_Control_KDM5B.jpg]

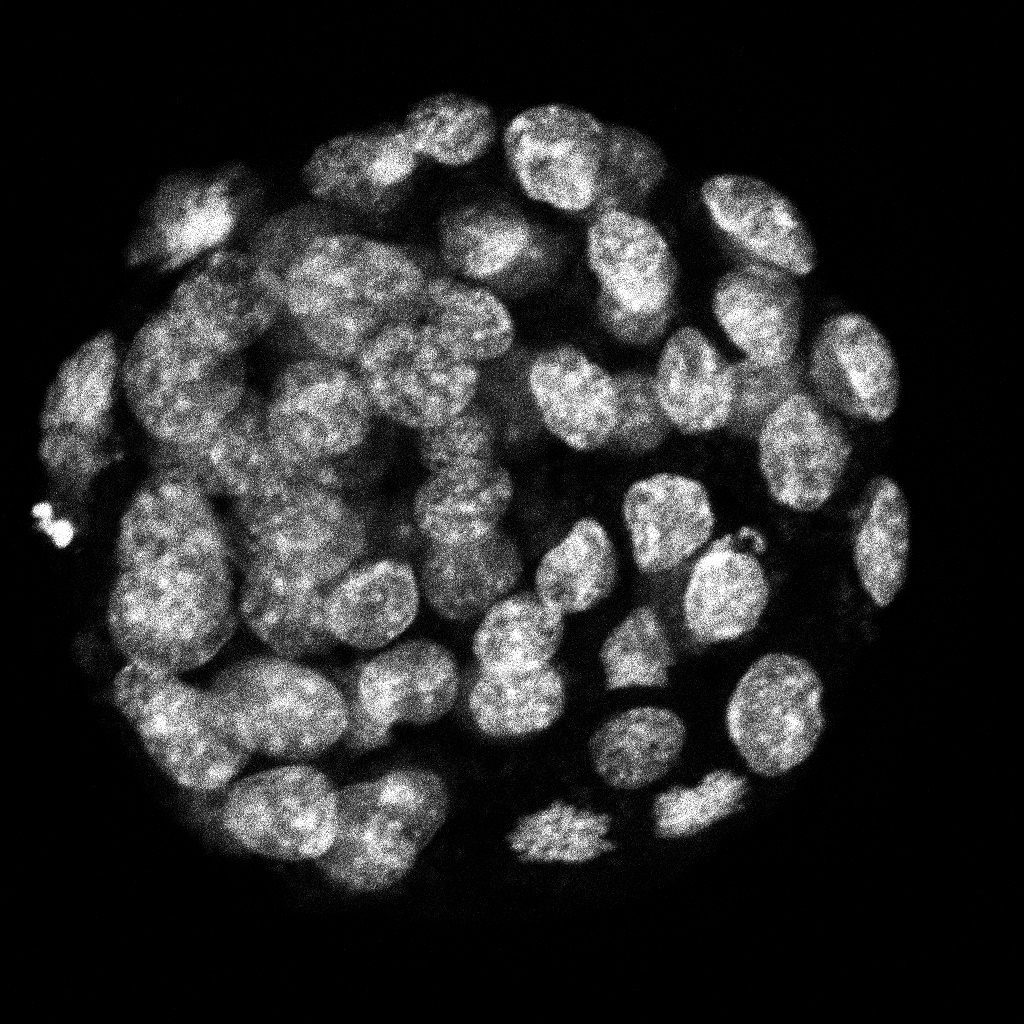

Supplement: Supplementary file 17 — Appendix Figure Source Data [file 44318_2024_329_MOESM17_ESM.zip › SD Appendix/FigS9B/S9B/Blastocyst_Kdm5b KD_DAPI.jpg]

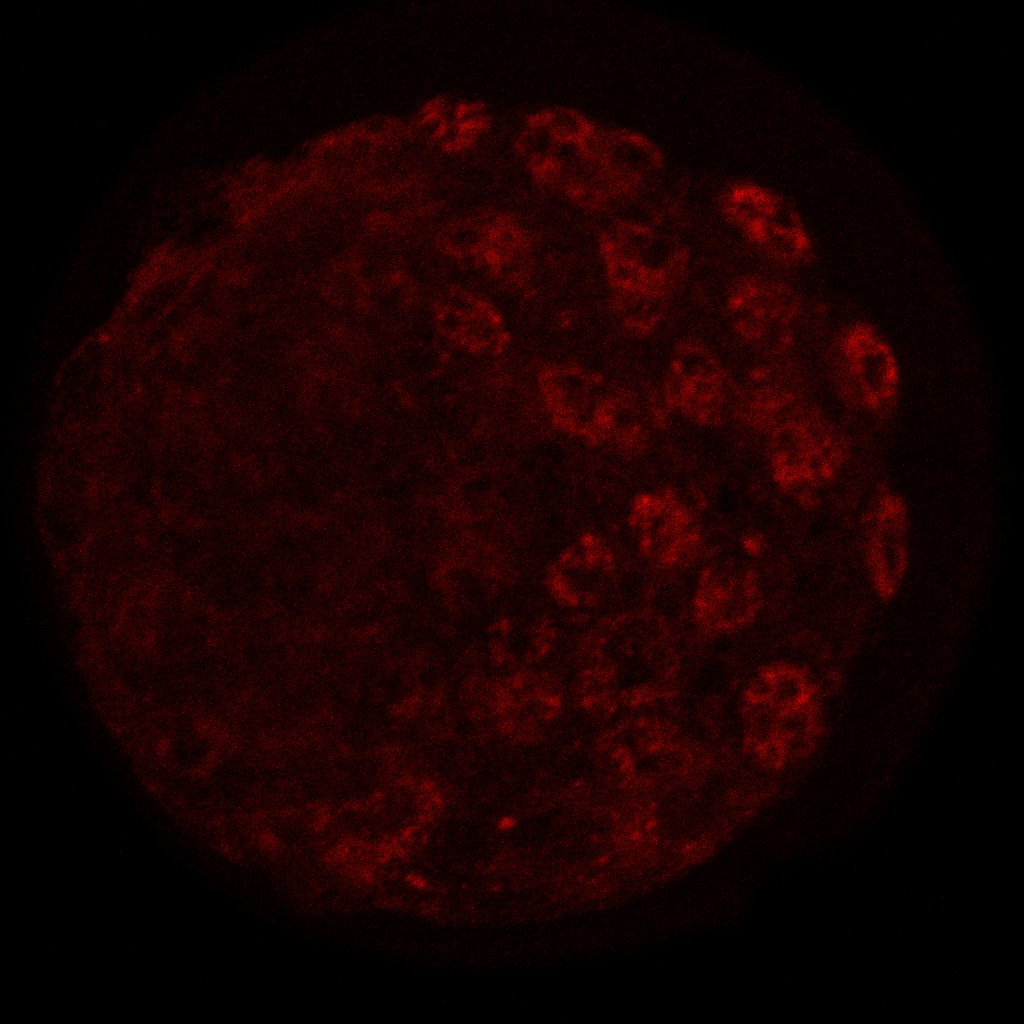

Supplement: Supplementary file 17 — Appendix Figure Source Data [file 44318_2024_329_MOESM17_ESM.zip › SD Appendix/FigS9B/S9B/Blastocyst_Kdm5b KD_KDM5B.jpg]

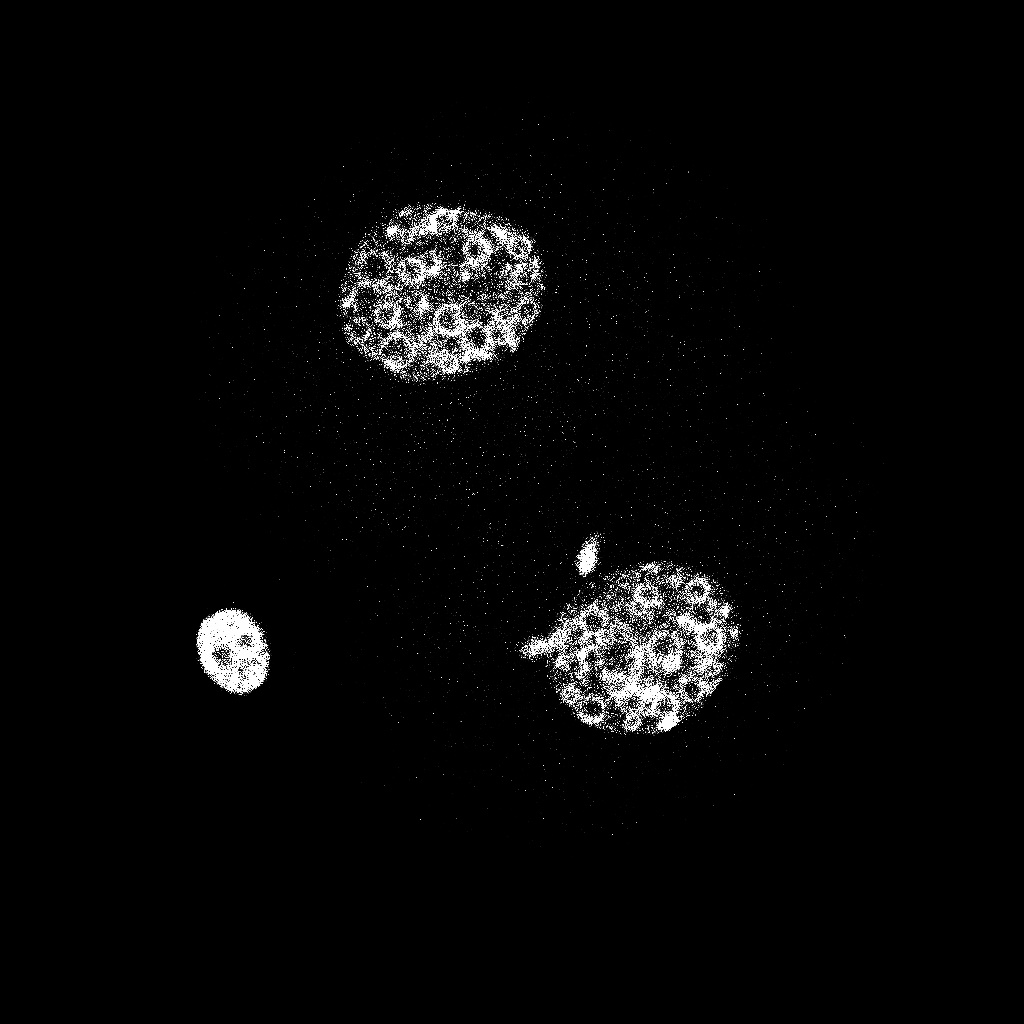

Supplement: Supplementary file 17 — Appendix Figure Source Data [file 44318_2024_329_MOESM17_ESM.zip › SD Appendix/FigS9B/S9B/Late2C_Control_DAPI.jpg]

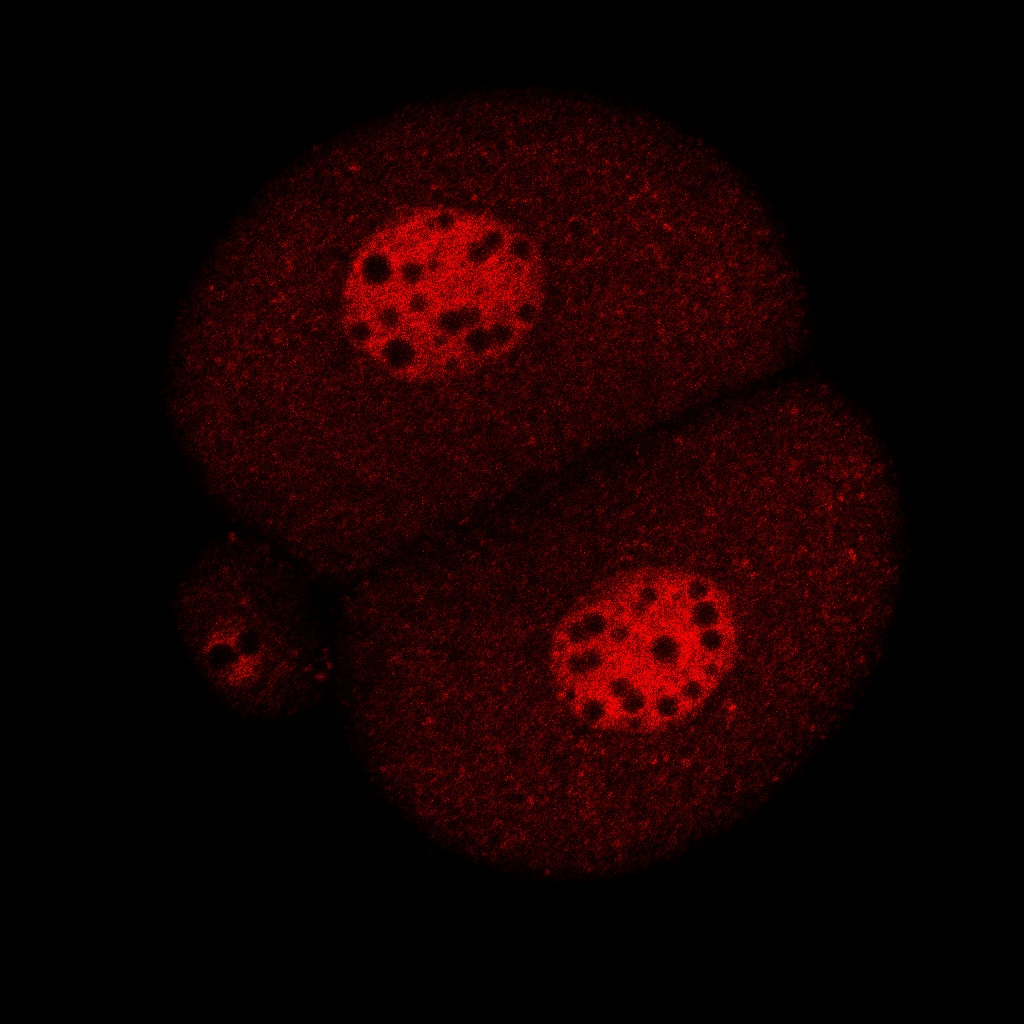

Supplement: Supplementary file 17 — Appendix Figure Source Data [file 44318_2024_329_MOESM17_ESM.zip › SD Appendix/FigS9B/S9B/Late2C_Control_KDM5B.jpg]

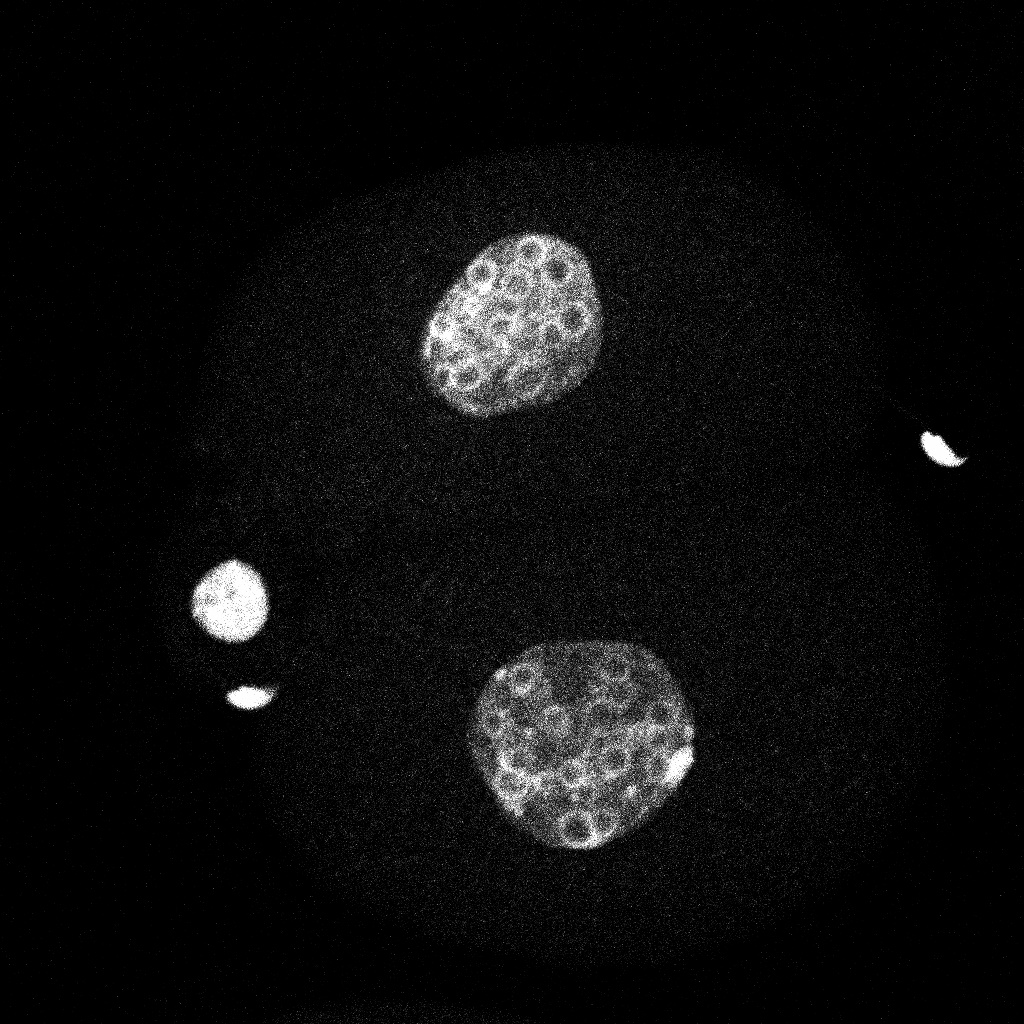

Supplement: Supplementary file 17 — Appendix Figure Source Data [file 44318_2024_329_MOESM17_ESM.zip › SD Appendix/FigS9B/S9B/Late2C_Kdm5b KD_DAPI.jpg]

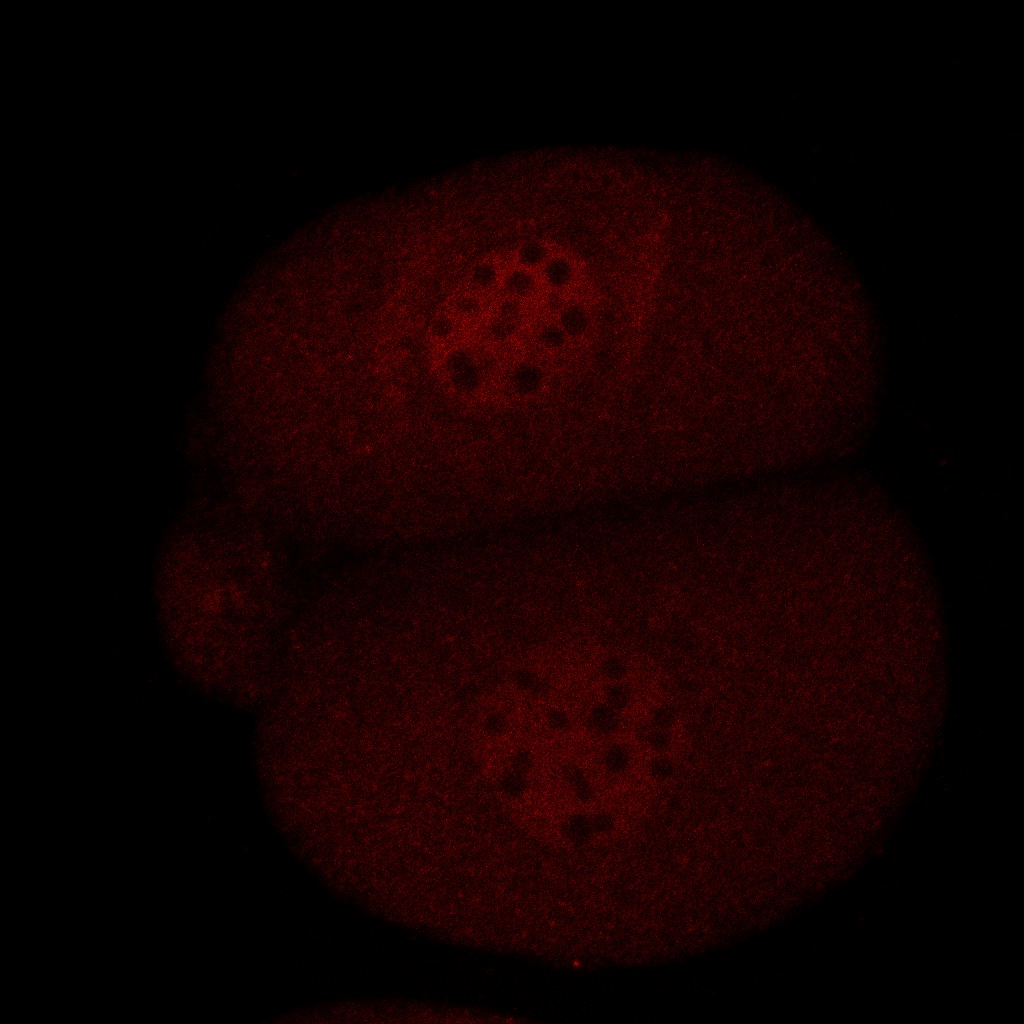

Supplement: Supplementary file 17 — Appendix Figure Source Data [file 44318_2024_329_MOESM17_ESM.zip › SD Appendix/FigS9B/S9B/Late2C_Kdm5b KD_KDM5B.jpg]

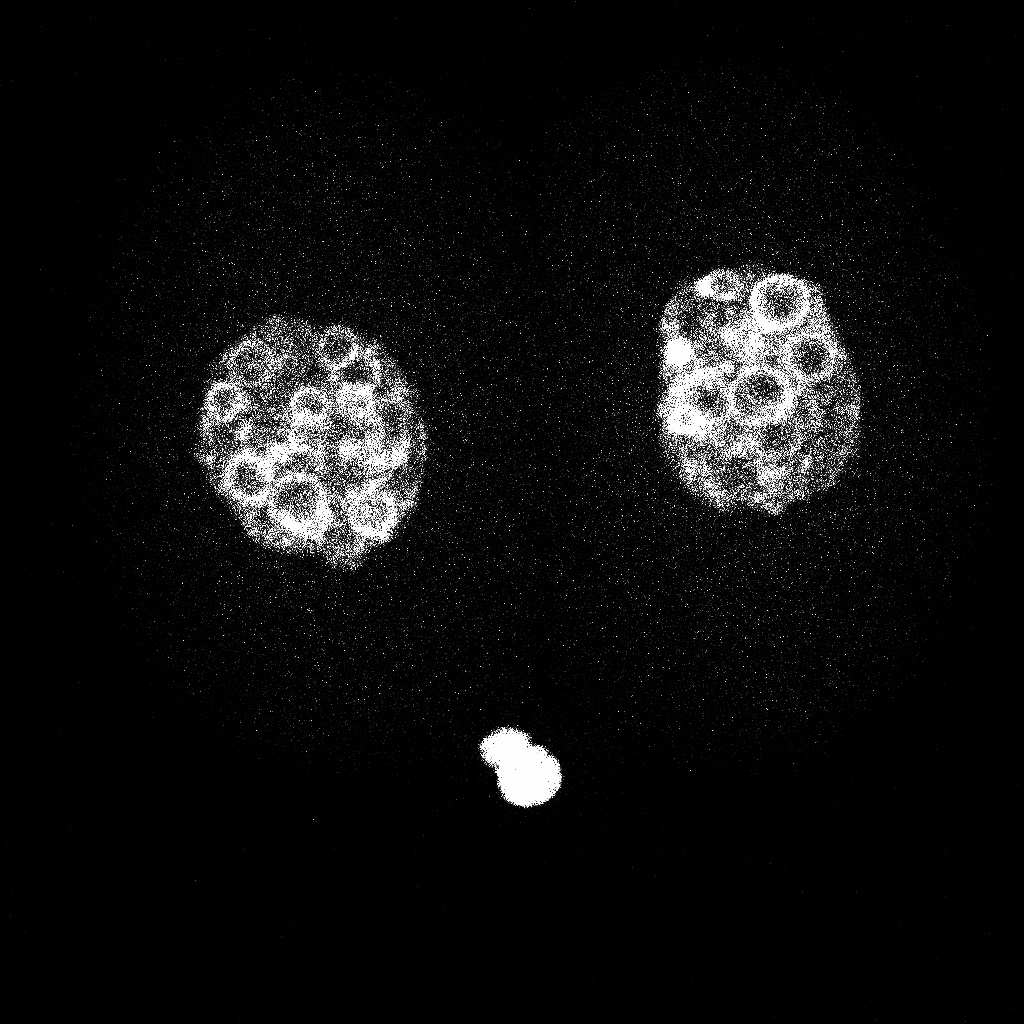

Supplement: Supplementary file 17 — Appendix Figure Source Data [file 44318_2024_329_MOESM17_ESM.zip › SD Appendix/FigS9C/S9C/Late2C_Control_DAPI.jpg]

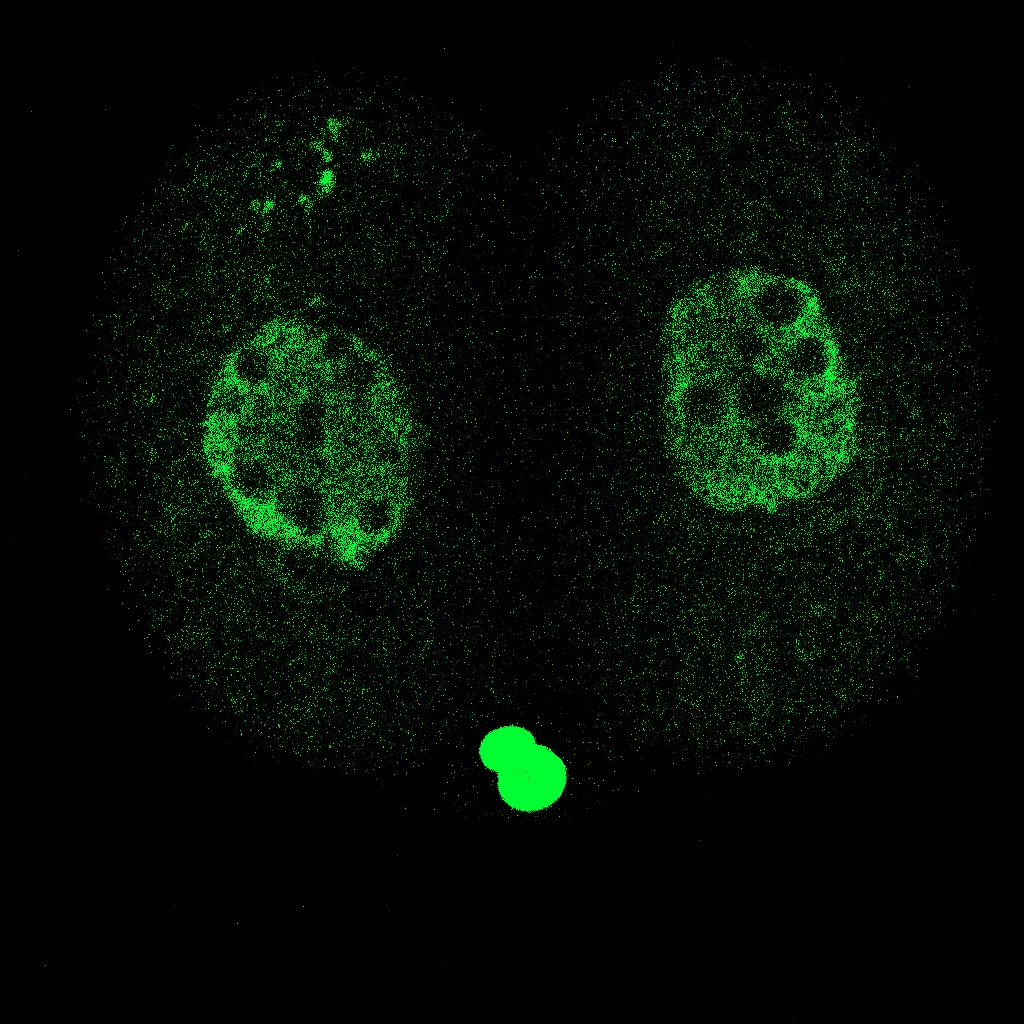

Supplement: Supplementary file 17 — Appendix Figure Source Data [file 44318_2024_329_MOESM17_ESM.zip › SD Appendix/FigS9C/S9C/Late2C_Control_H3K4me3.jpg]

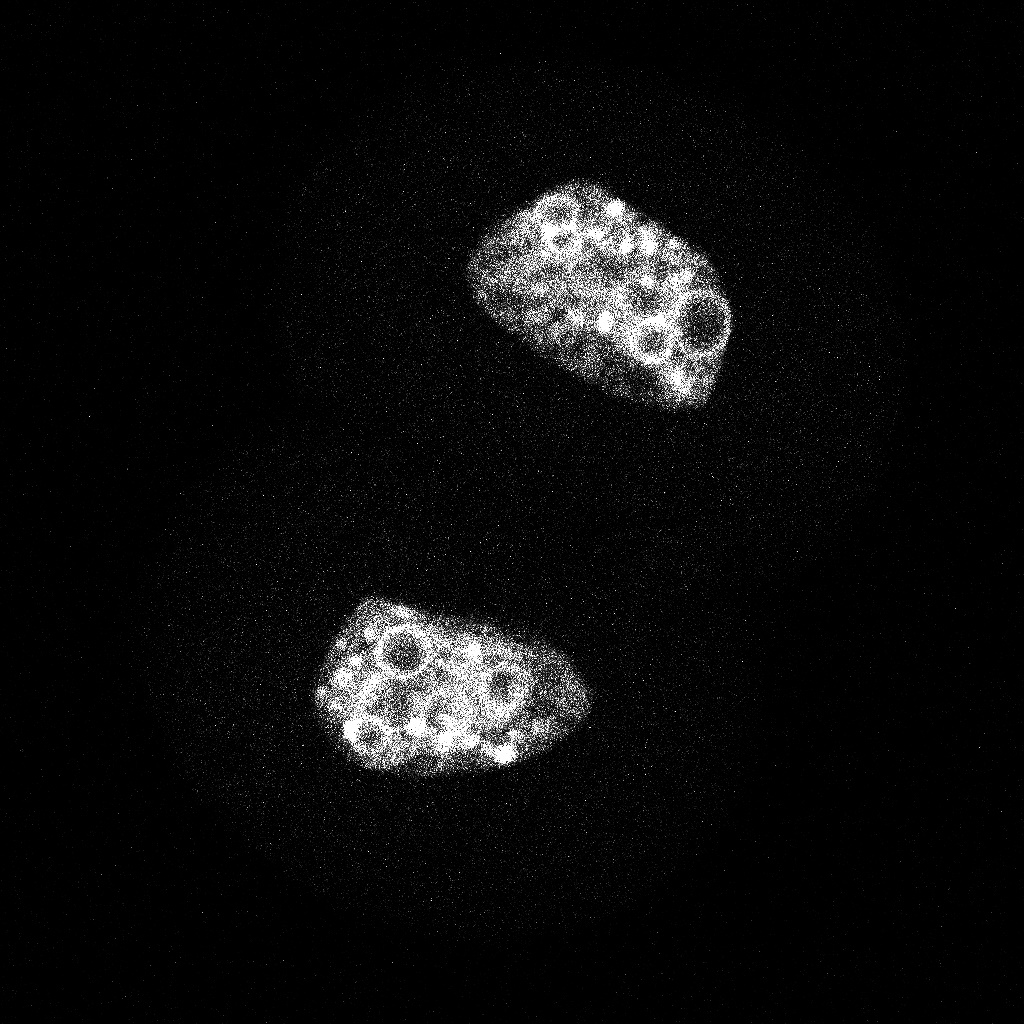

Supplement: Supplementary file 17 — Appendix Figure Source Data [file 44318_2024_329_MOESM17_ESM.zip › SD Appendix/FigS9C/S9C/Late2C_Kdm5ab KD_DAPI.jpg]

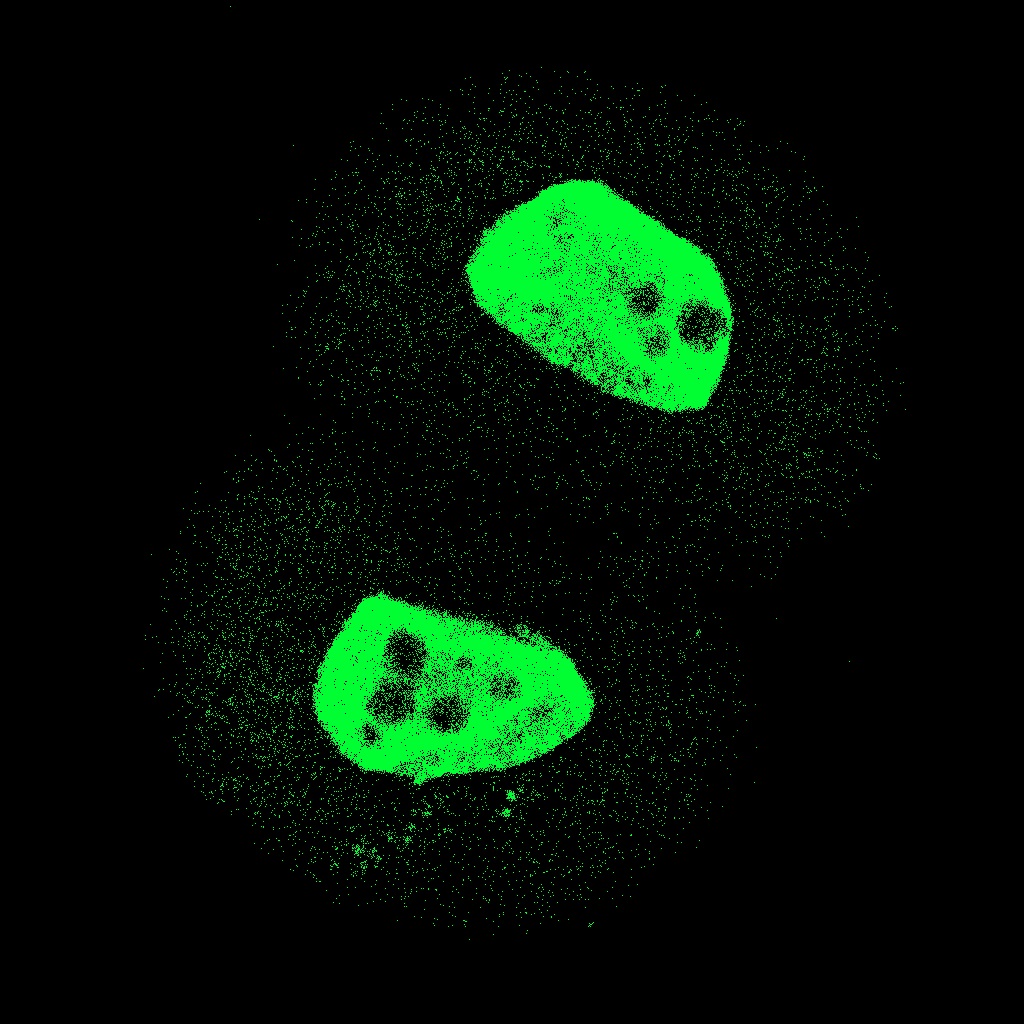

Supplement: Supplementary file 17 — Appendix Figure Source Data [file 44318_2024_329_MOESM17_ESM.zip › SD Appendix/FigS9C/S9C/Late2C_Kdm5ab KD_H3K4me3.jpg]

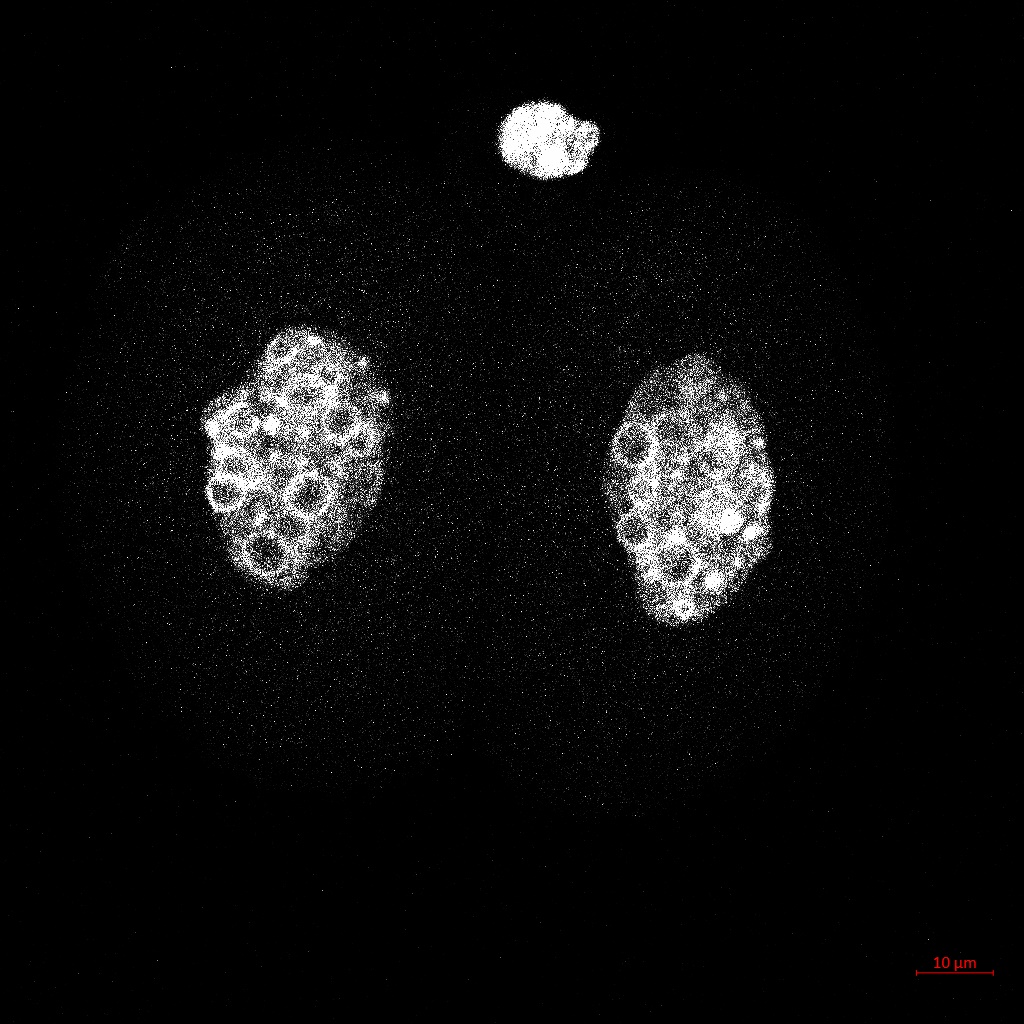

Supplement: Supplementary file 17 — Appendix Figure Source Data [file 44318_2024_329_MOESM17_ESM.zip › SD Appendix/FigS9C/S9C/Late2C_Kdm5b KD_DAPI.jpg]

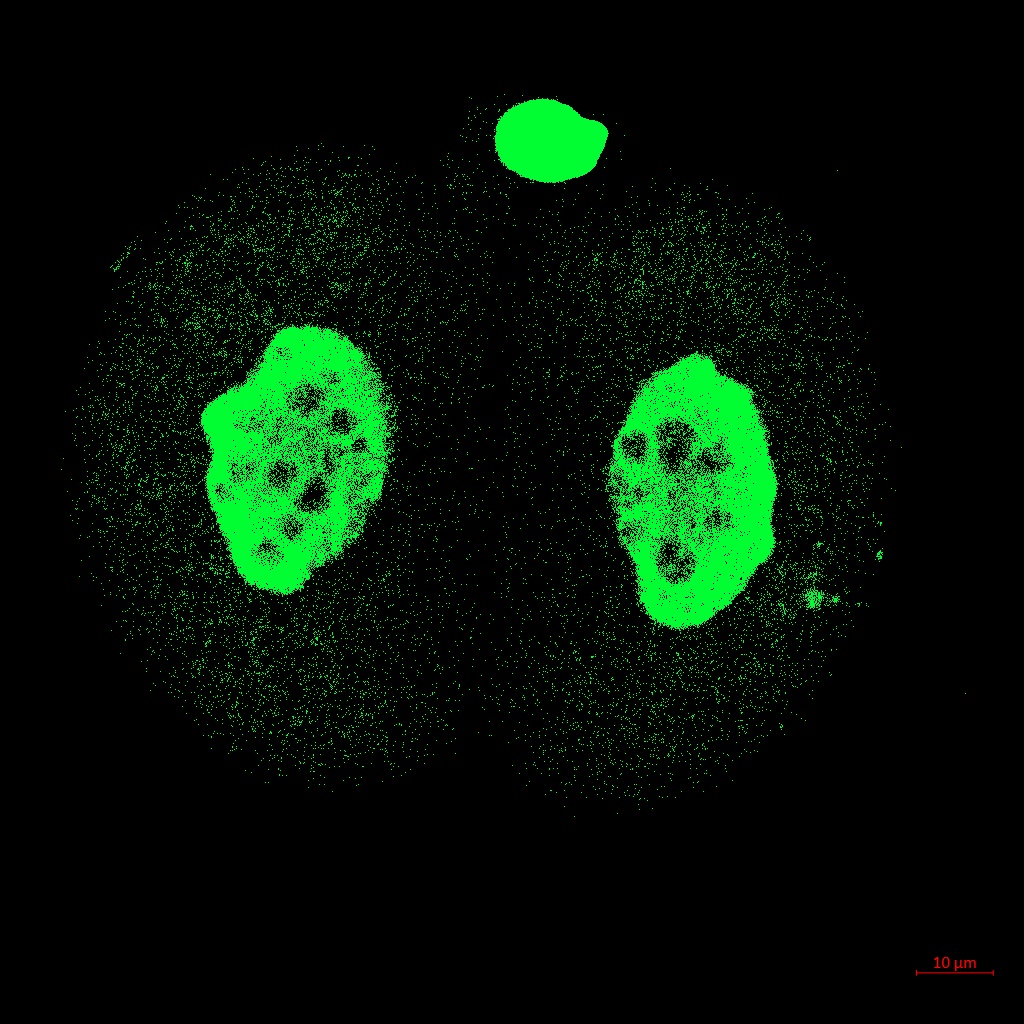

Supplement: Supplementary file 17 — Appendix Figure Source Data [file 44318_2024_329_MOESM17_ESM.zip › SD Appendix/FigS9C/S9C/Late2C_Kdm5b KD_H3K4me3.jpg]

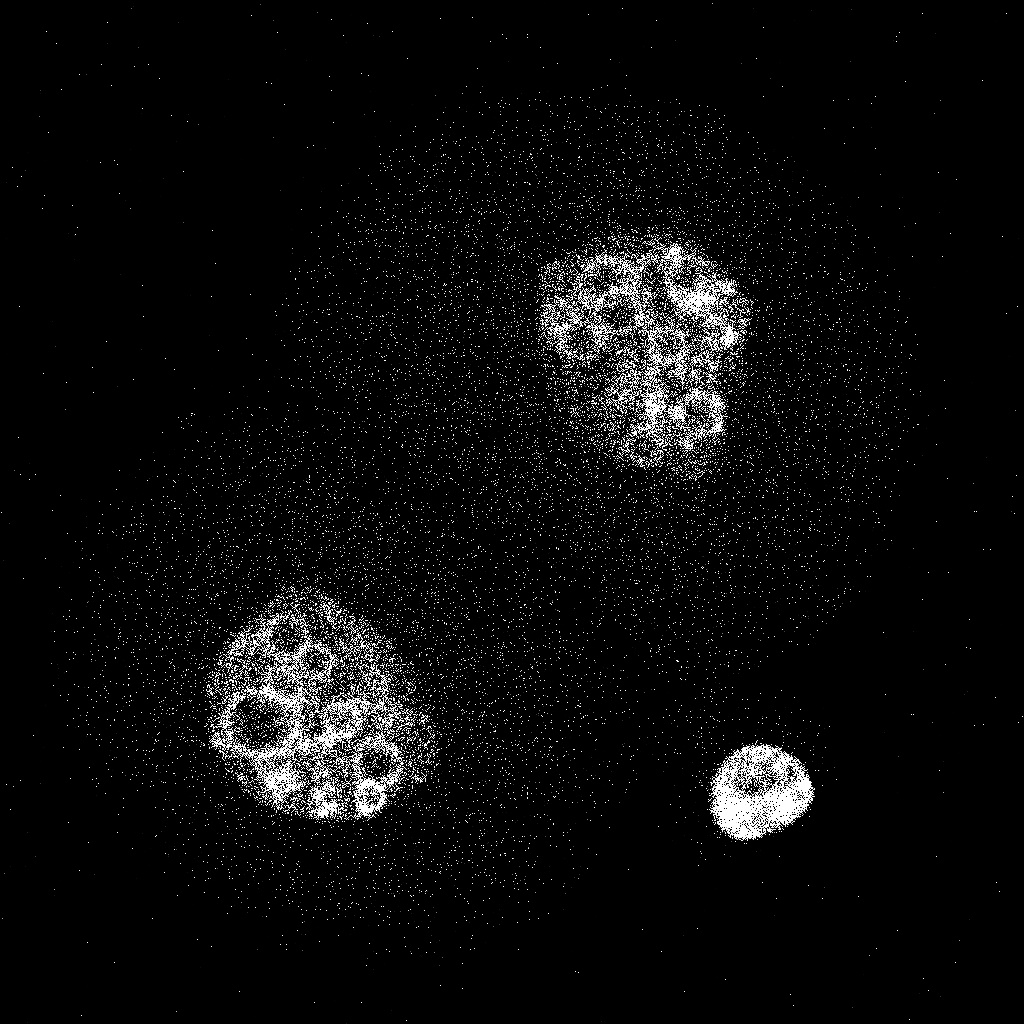

Supplement: Supplementary file 17 — Appendix Figure Source Data [file 44318_2024_329_MOESM17_ESM.zip › SD Appendix/FigS9D/S9D/Late2C_0 μM_DAPI.jpg]

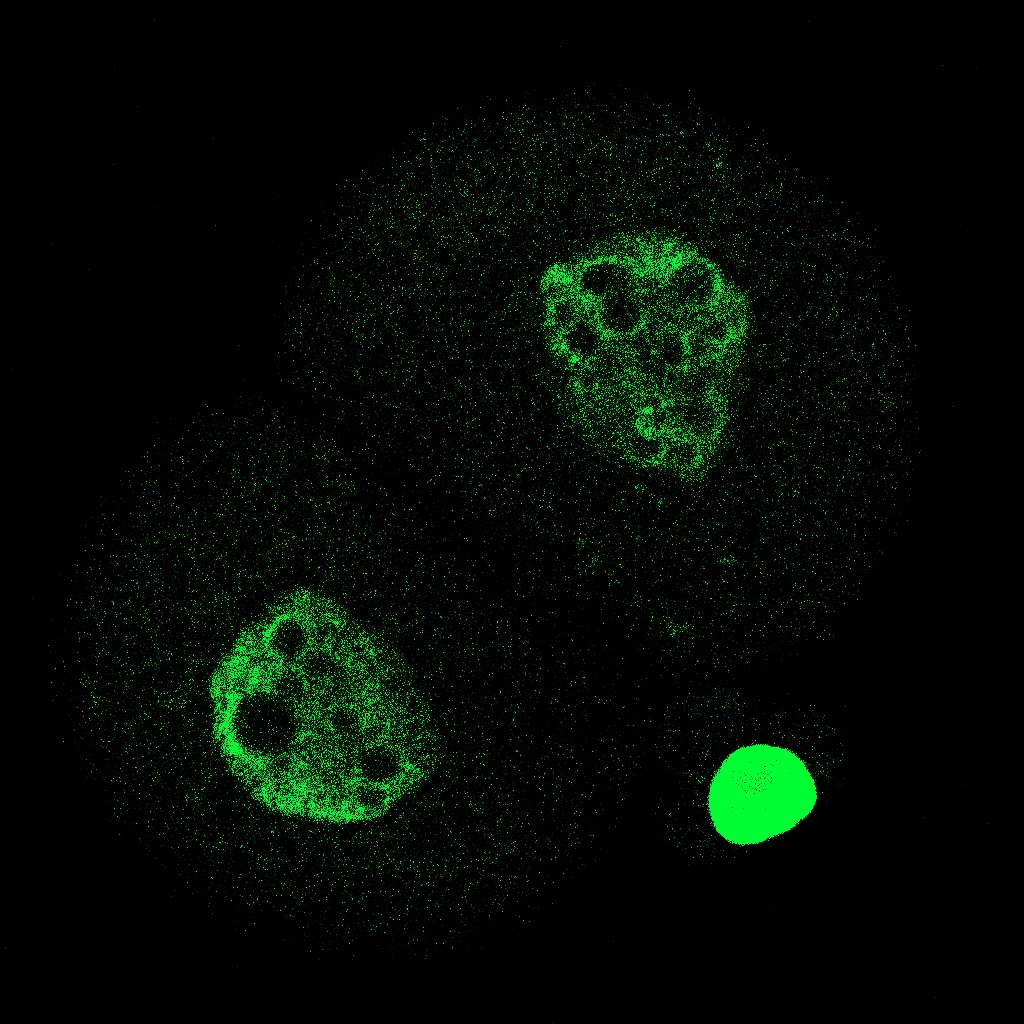

Supplement: Supplementary file 17 — Appendix Figure Source Data [file 44318_2024_329_MOESM17_ESM.zip › SD Appendix/FigS9D/S9D/Late2C_0 μM_H3K4me3.jpg]

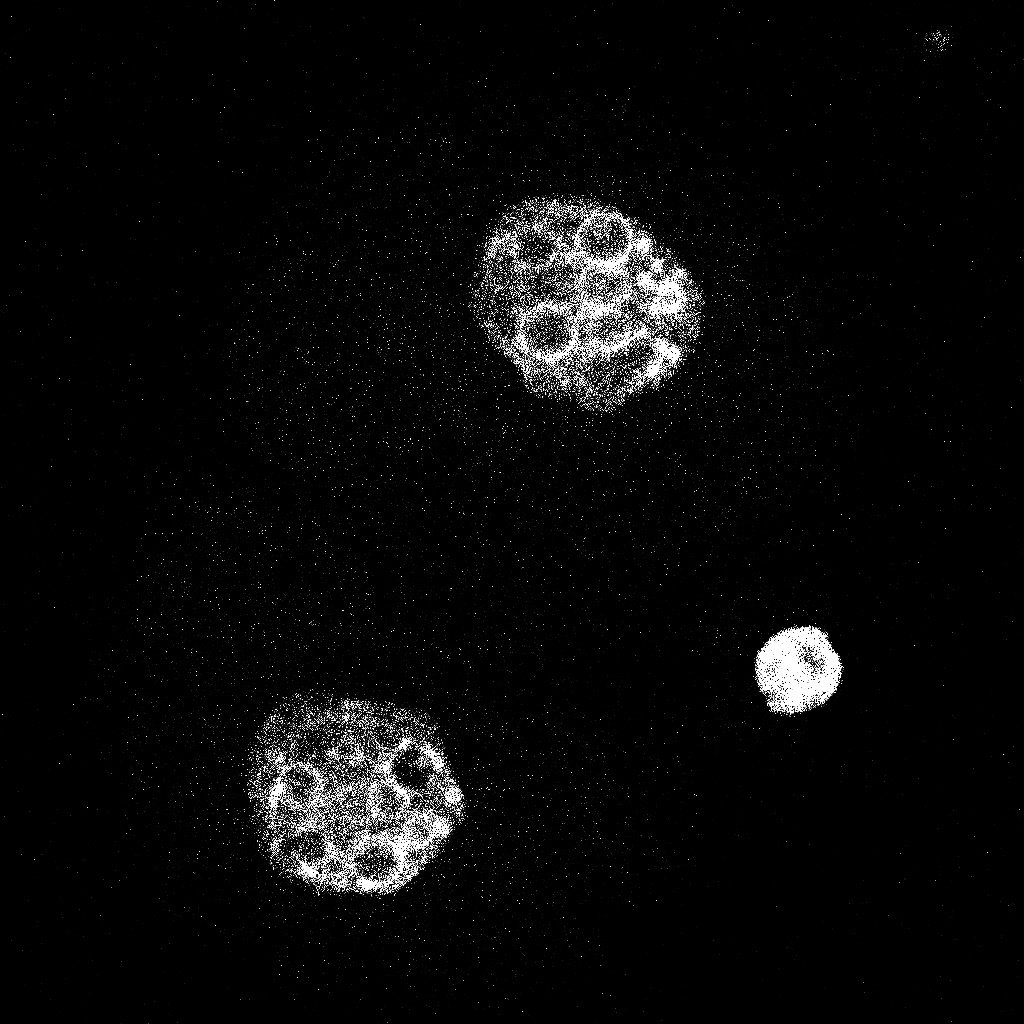

Supplement: Supplementary file 17 — Appendix Figure Source Data [file 44318_2024_329_MOESM17_ESM.zip › SD Appendix/FigS9D/S9D/Late2C_10 μM_DAPI.jpg]

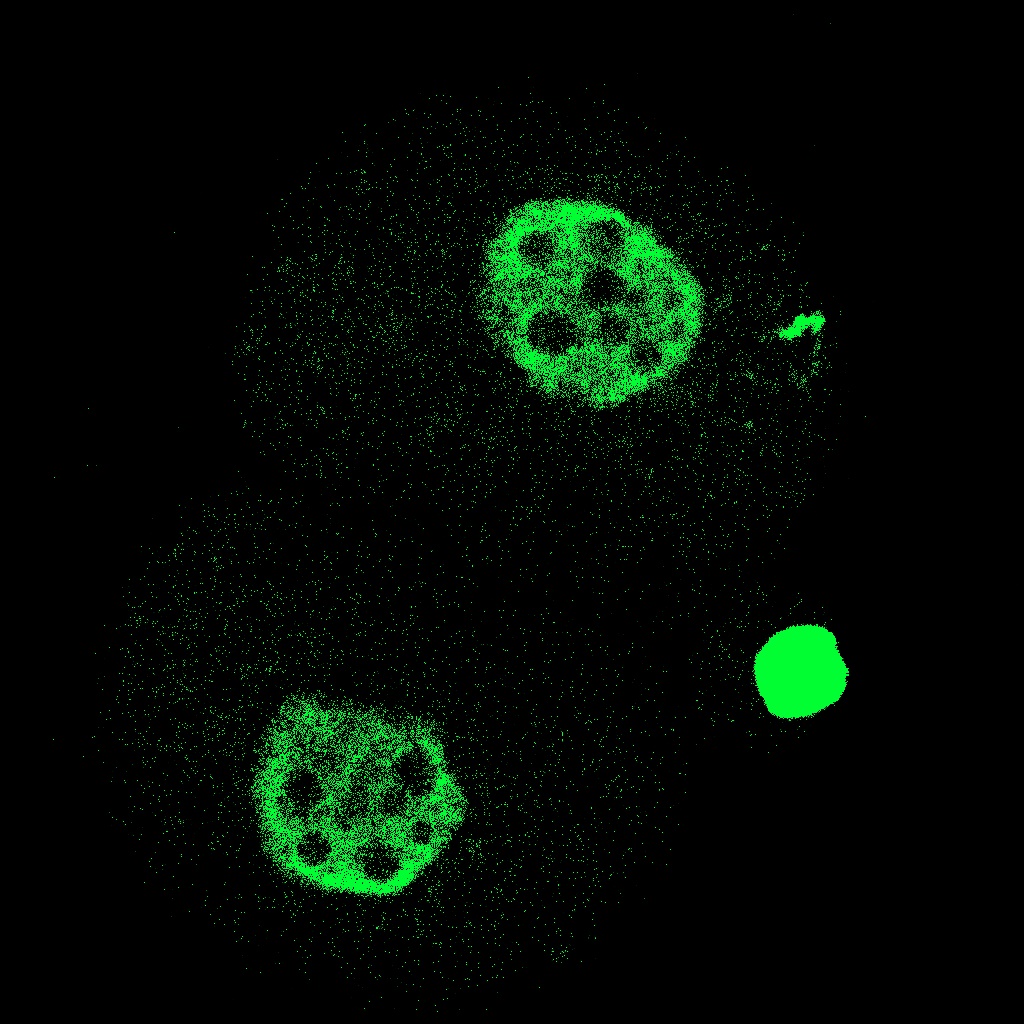

Supplement: Supplementary file 17 — Appendix Figure Source Data [file 44318_2024_329_MOESM17_ESM.zip › SD Appendix/FigS9D/S9D/Late2C_10 μM_H3K4me3.jpg]

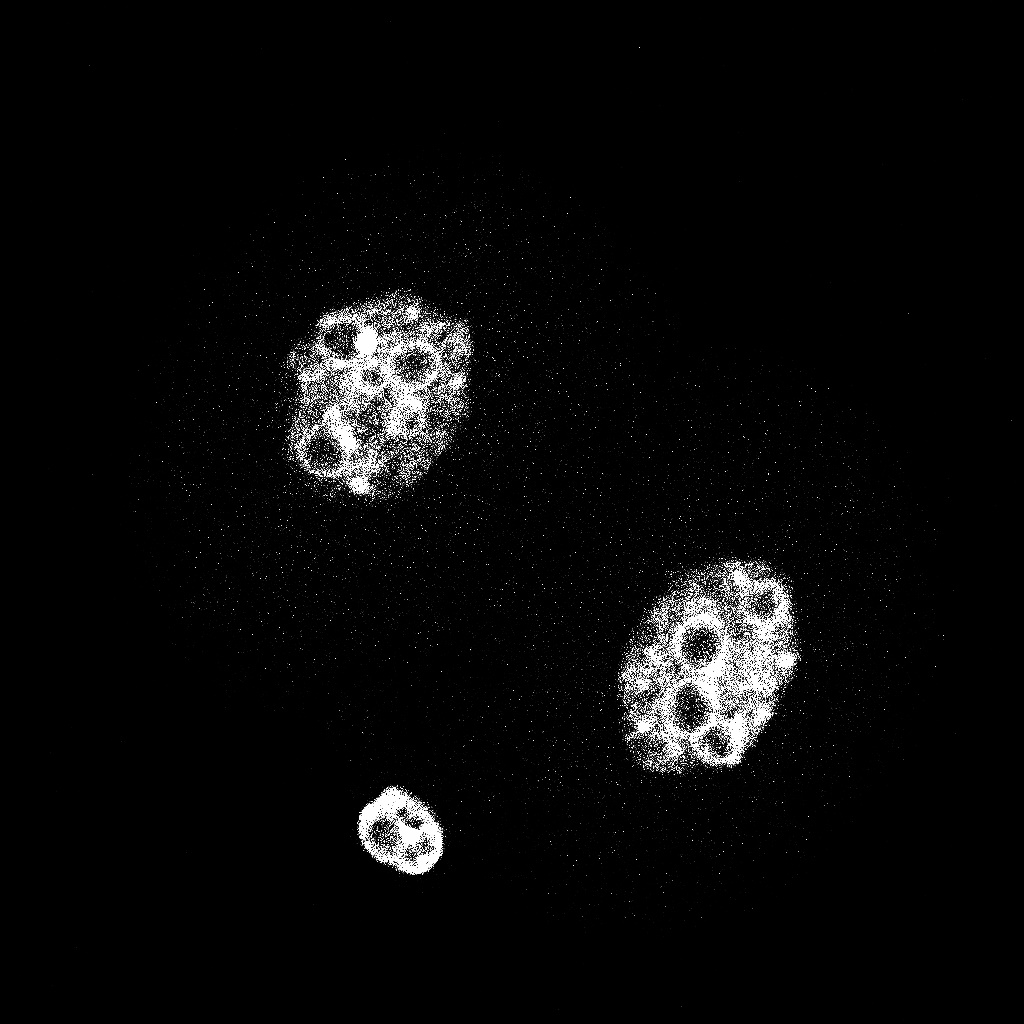

Supplement: Supplementary file 17 — Appendix Figure Source Data [file 44318_2024_329_MOESM17_ESM.zip › SD Appendix/FigS9D/S9D/Late2C_25 μM_DAPI.jpg]

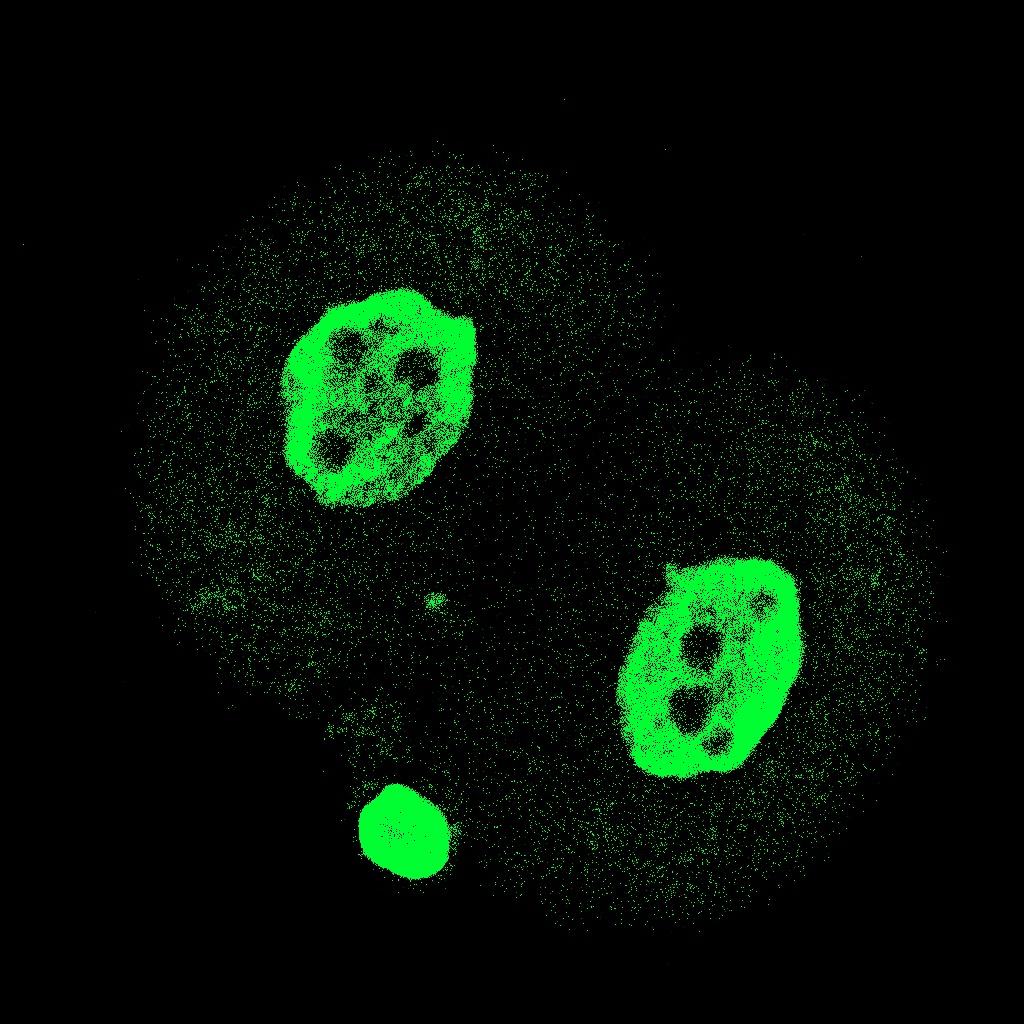

Supplement: Supplementary file 17 — Appendix Figure Source Data [file 44318_2024_329_MOESM17_ESM.zip › SD Appendix/FigS9D/S9D/Late2C_25 μM_H3K4me3.jpg]

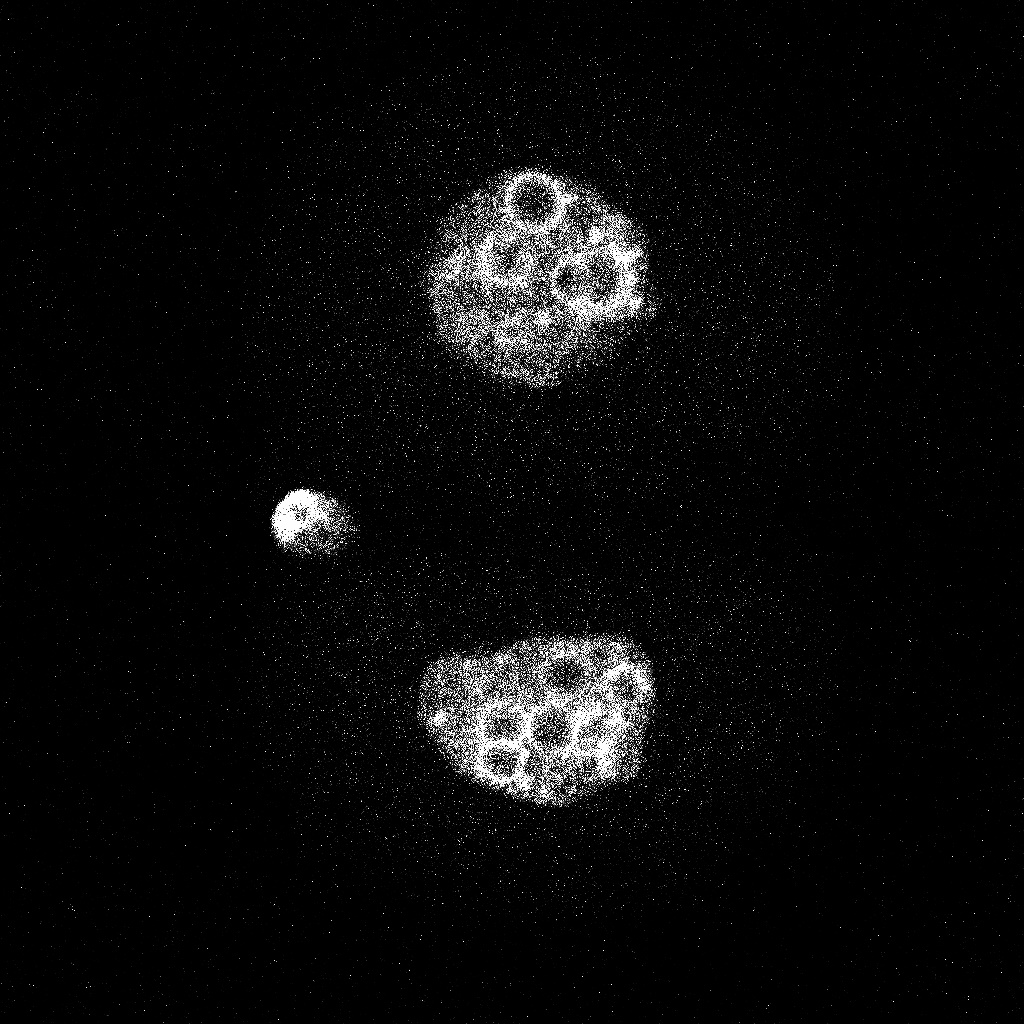

Supplement: Supplementary file 17 — Appendix Figure Source Data [file 44318_2024_329_MOESM17_ESM.zip › SD Appendix/FigS9D/S9D/Late2C_50 μM_DAPI.jpg]

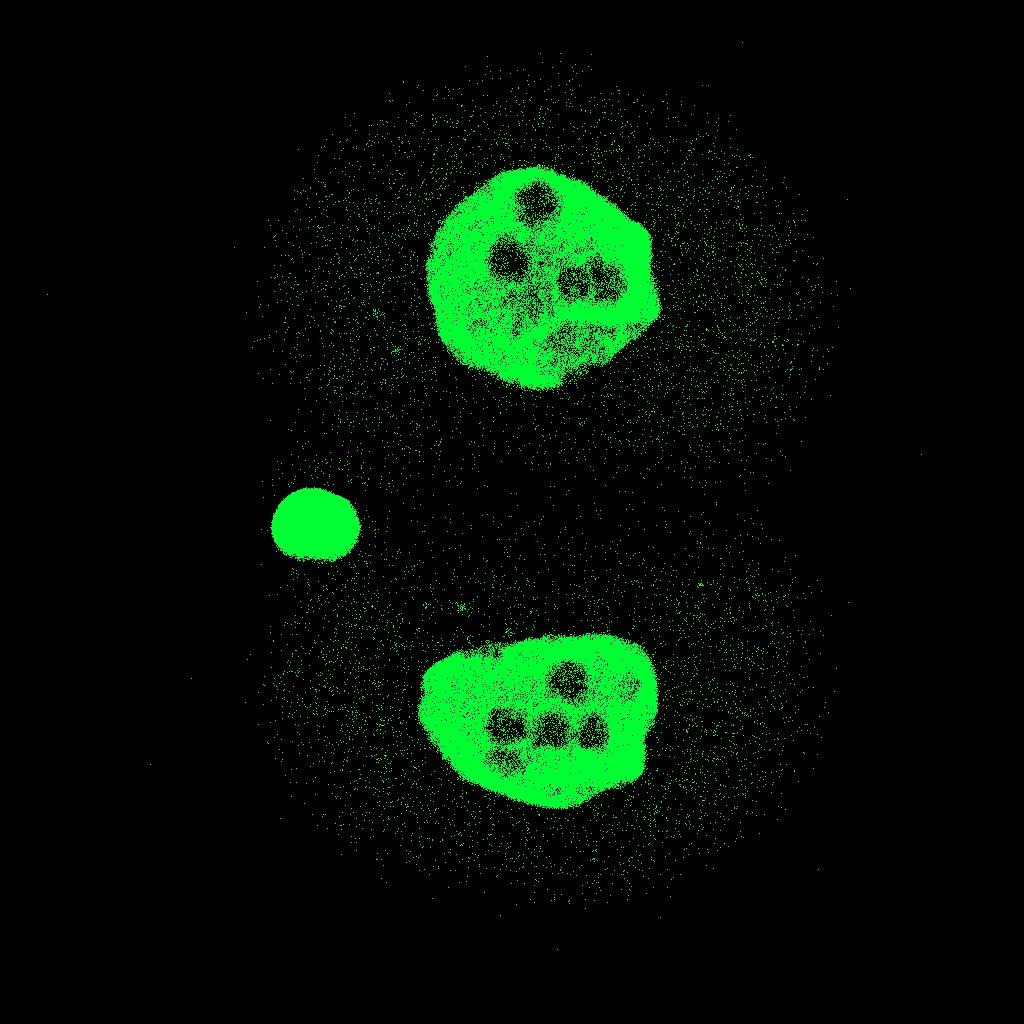

Supplement: Supplementary file 17 — Appendix Figure Source Data [file 44318_2024_329_MOESM17_ESM.zip › SD Appendix/FigS9D/S9D/Late2C_50 μM_H3K4me3.jpg]
