# Supplementary figures and images for: Histone methyltransferases MLL2 and SETD1A/B play distinct roles in H3K4me3 deposition during the transition from totipotency to pluripotency (part 2 of 3)
Source: EMBO J. 2024 Dec 5;44(2):437–56. doi: 10.1038/s44318-024-00329-5 (PMC11730331; doi:10.1038/s44318-024-00329-5)

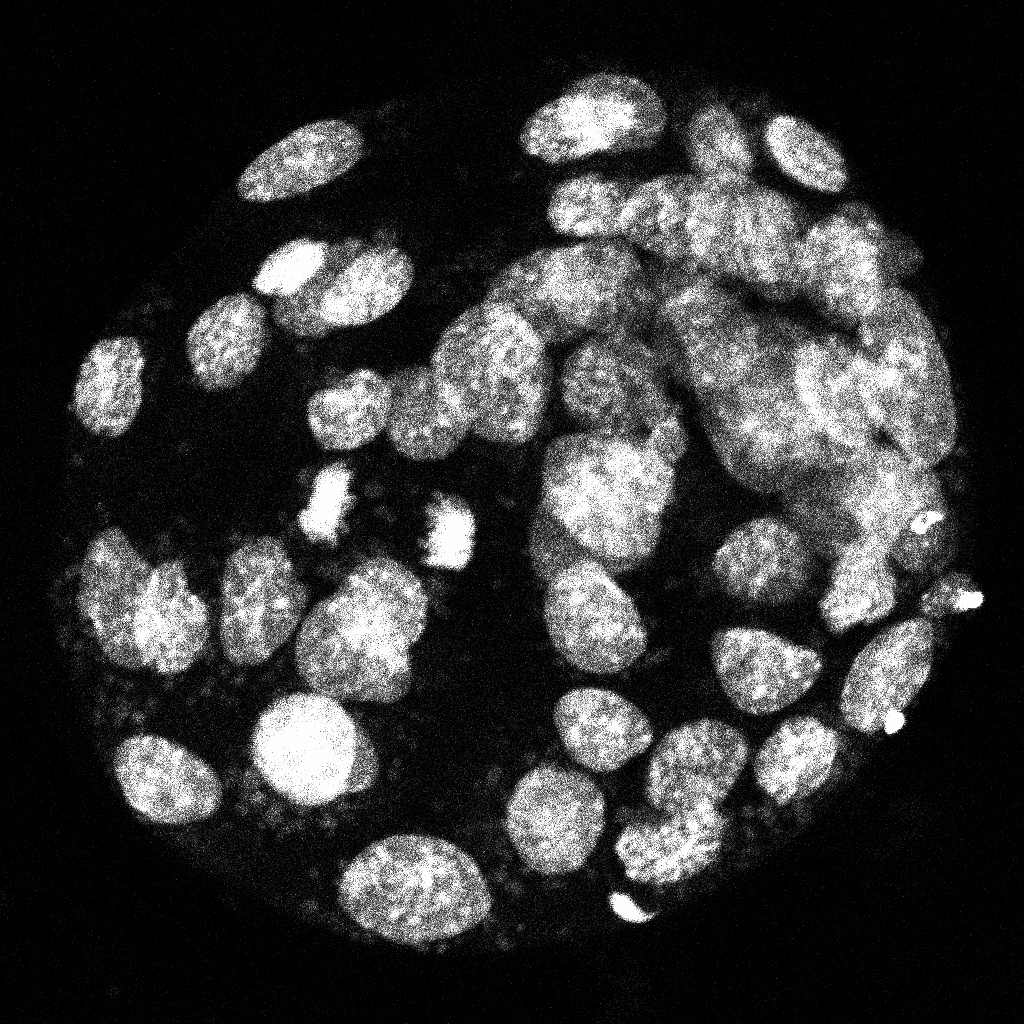

Supplement: Supplementary file 17 — Appendix Figure Source Data [file 44318_2024_329_MOESM17_ESM.zip › SD Appendix/FigS11A/S11A/Blastocyst_Setd1ab+hMUT mRNA_DAPI.jpg]

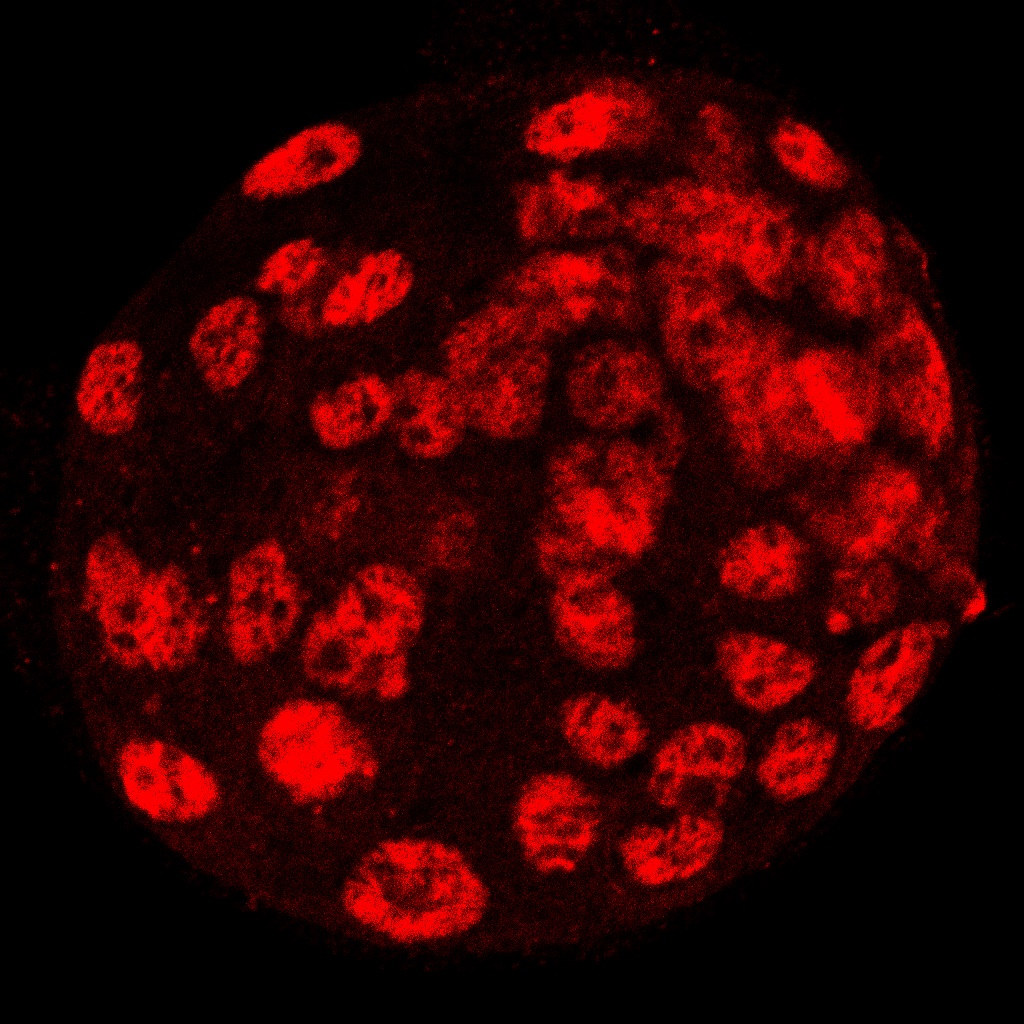

Supplement: Supplementary file 17 — Appendix Figure Source Data [file 44318_2024_329_MOESM17_ESM.zip › SD Appendix/FigS11A/S11A/Blastocyst_Setd1ab+hMUT mRNA_SETD1A.jpg]

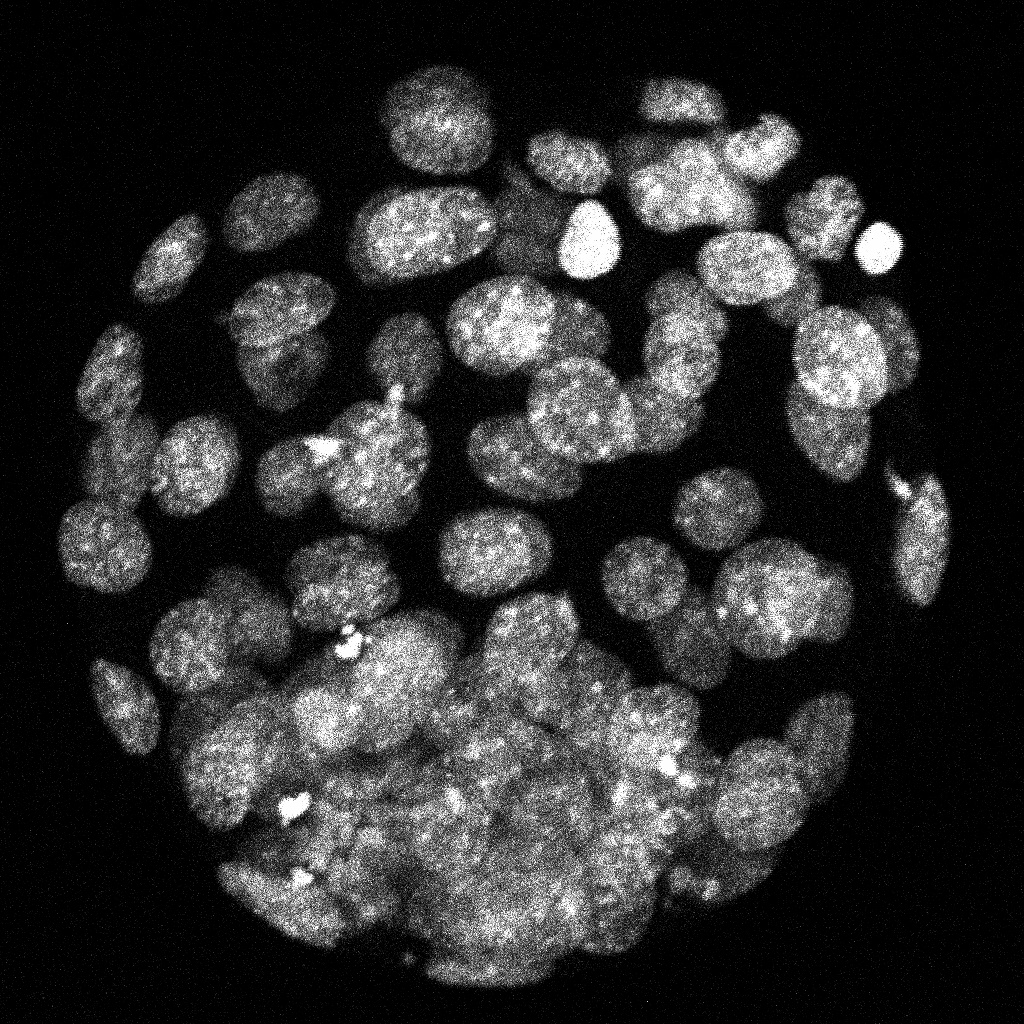

Supplement: Supplementary file 17 — Appendix Figure Source Data [file 44318_2024_329_MOESM17_ESM.zip › SD Appendix/FigS11B/S11B/Blastocyst_Control_DAPI.jpg]

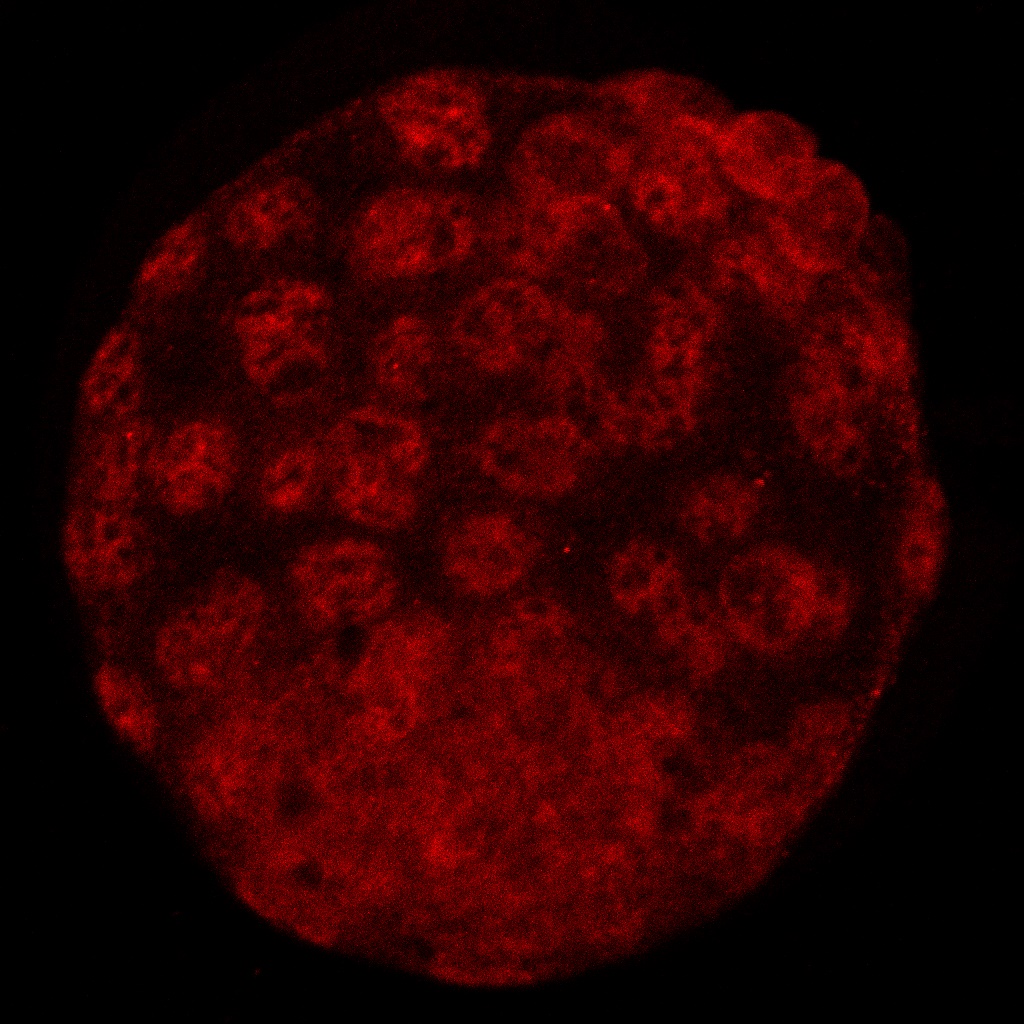

Supplement: Supplementary file 17 — Appendix Figure Source Data [file 44318_2024_329_MOESM17_ESM.zip › SD Appendix/FigS11B/S11B/Blastocyst_Control_SETD1B.jpg]

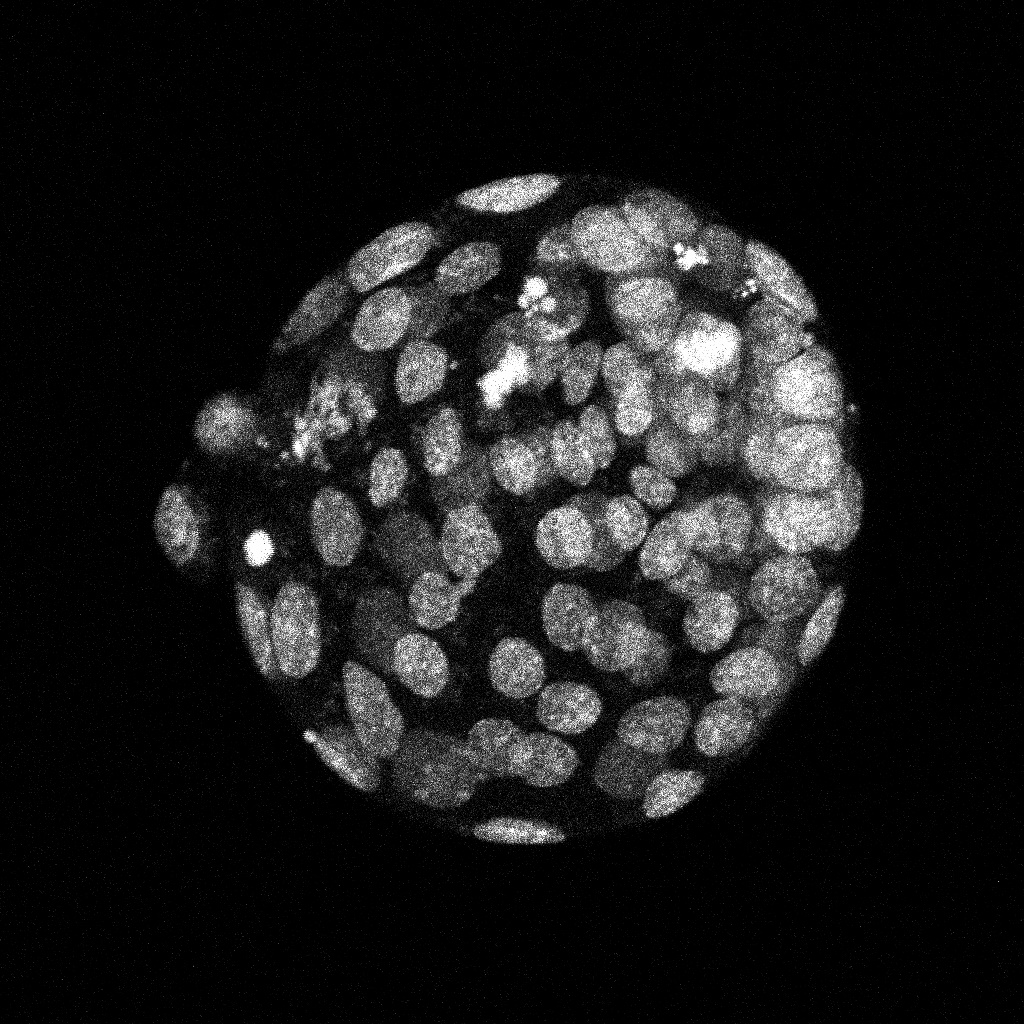

Supplement: Supplementary file 17 — Appendix Figure Source Data [file 44318_2024_329_MOESM17_ESM.zip › SD Appendix/FigS11B/S11B/Blastocyst_Setd1ab KD+hMUT mRNA_DAPI.jpg]

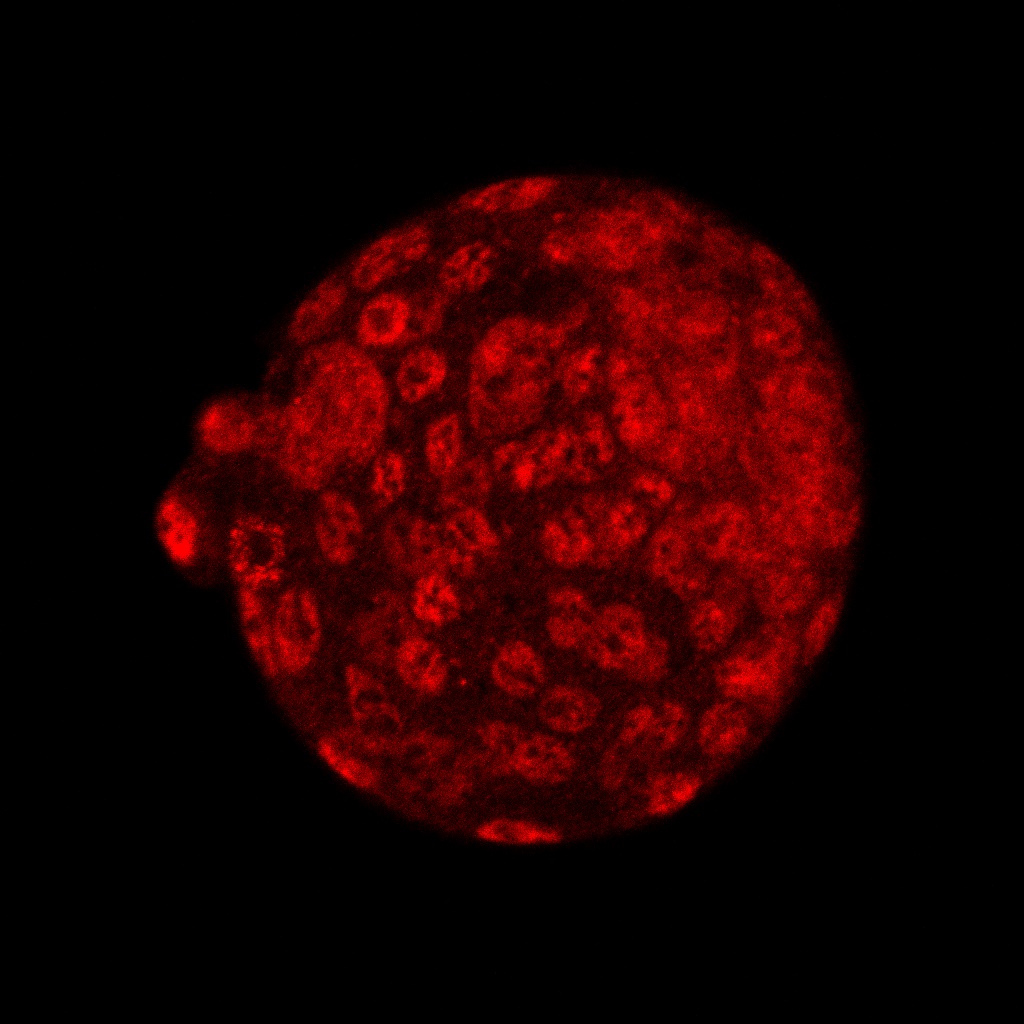

Supplement: Supplementary file 17 — Appendix Figure Source Data [file 44318_2024_329_MOESM17_ESM.zip › SD Appendix/FigS11B/S11B/Blastocyst_Setd1ab KD+hMUT mRNA_SETD1B.jpg]

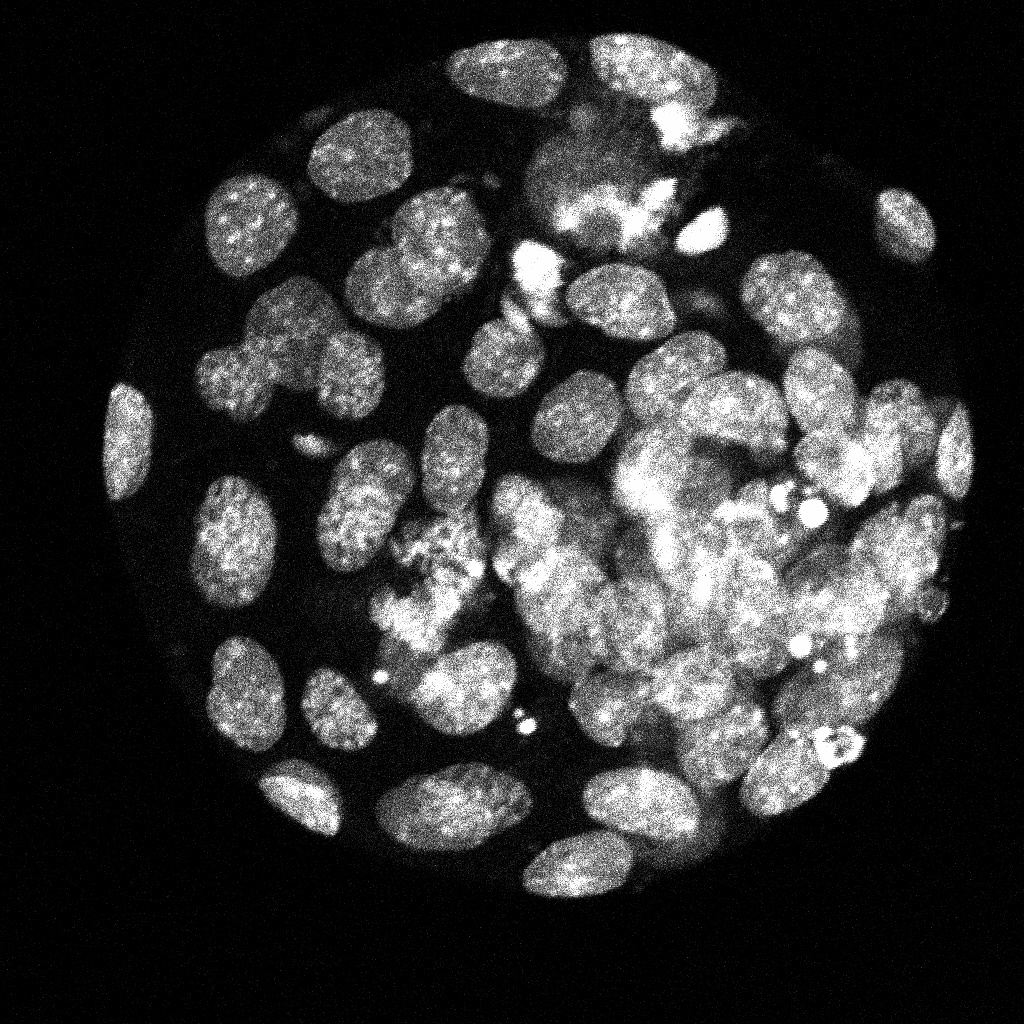

Supplement: Supplementary file 17 — Appendix Figure Source Data [file 44318_2024_329_MOESM17_ESM.zip › SD Appendix/FigS11B/S11B/Blastocyst_Setd1ab KD+hWT mRNA_DAPI.jpg]

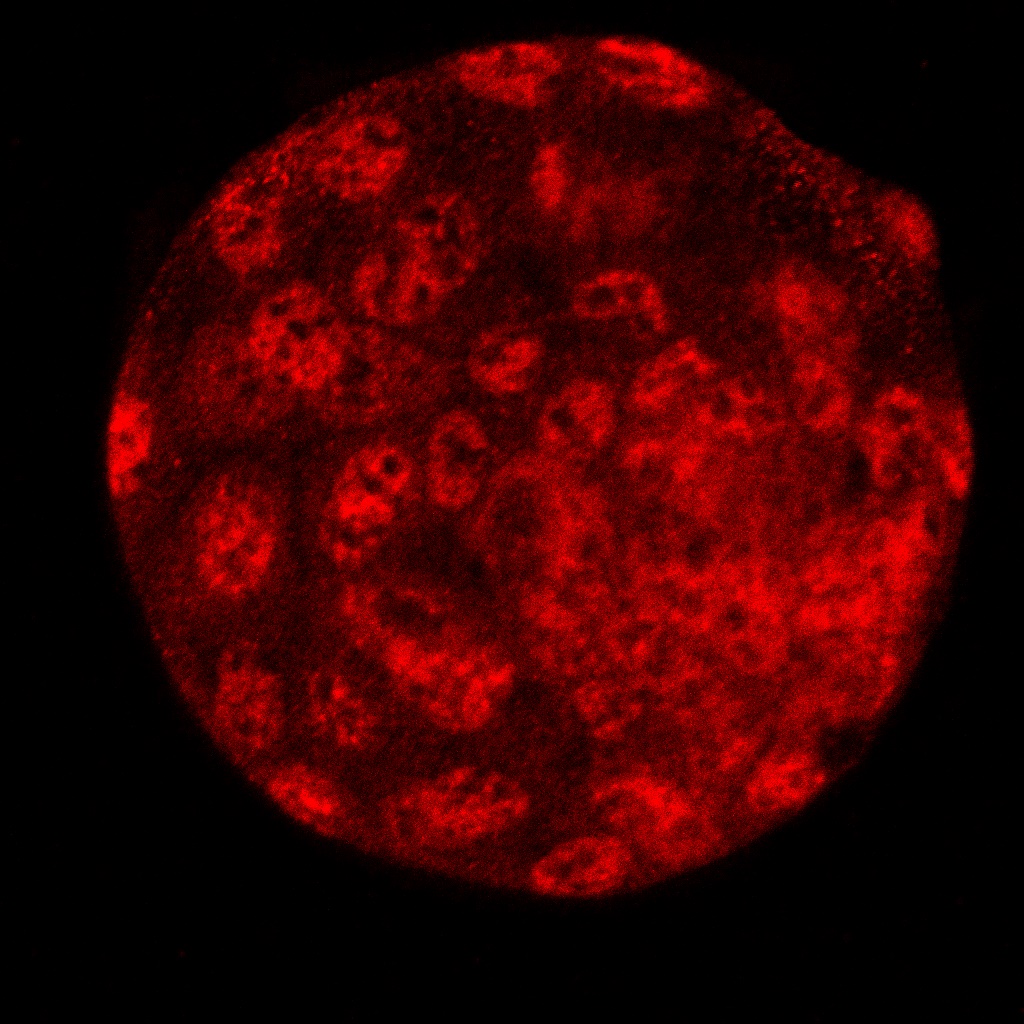

Supplement: Supplementary file 17 — Appendix Figure Source Data [file 44318_2024_329_MOESM17_ESM.zip › SD Appendix/FigS11B/S11B/Blastocyst_Setd1ab KD+hWT mRNA_SETD1B.jpg]

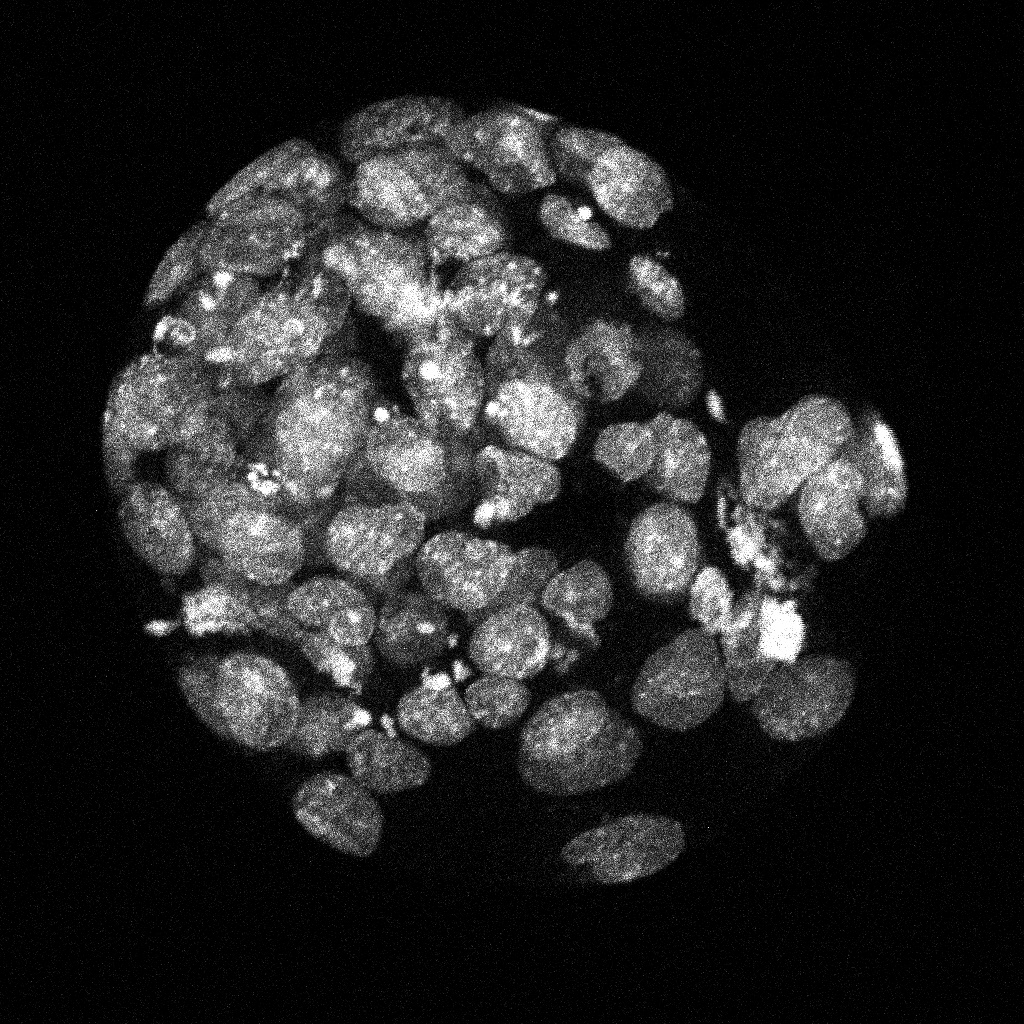

Supplement: Supplementary file 17 — Appendix Figure Source Data [file 44318_2024_329_MOESM17_ESM.zip › SD Appendix/FigS11B/S11B/Blastocyst_Setd1ab KD_DAPI.jpg]

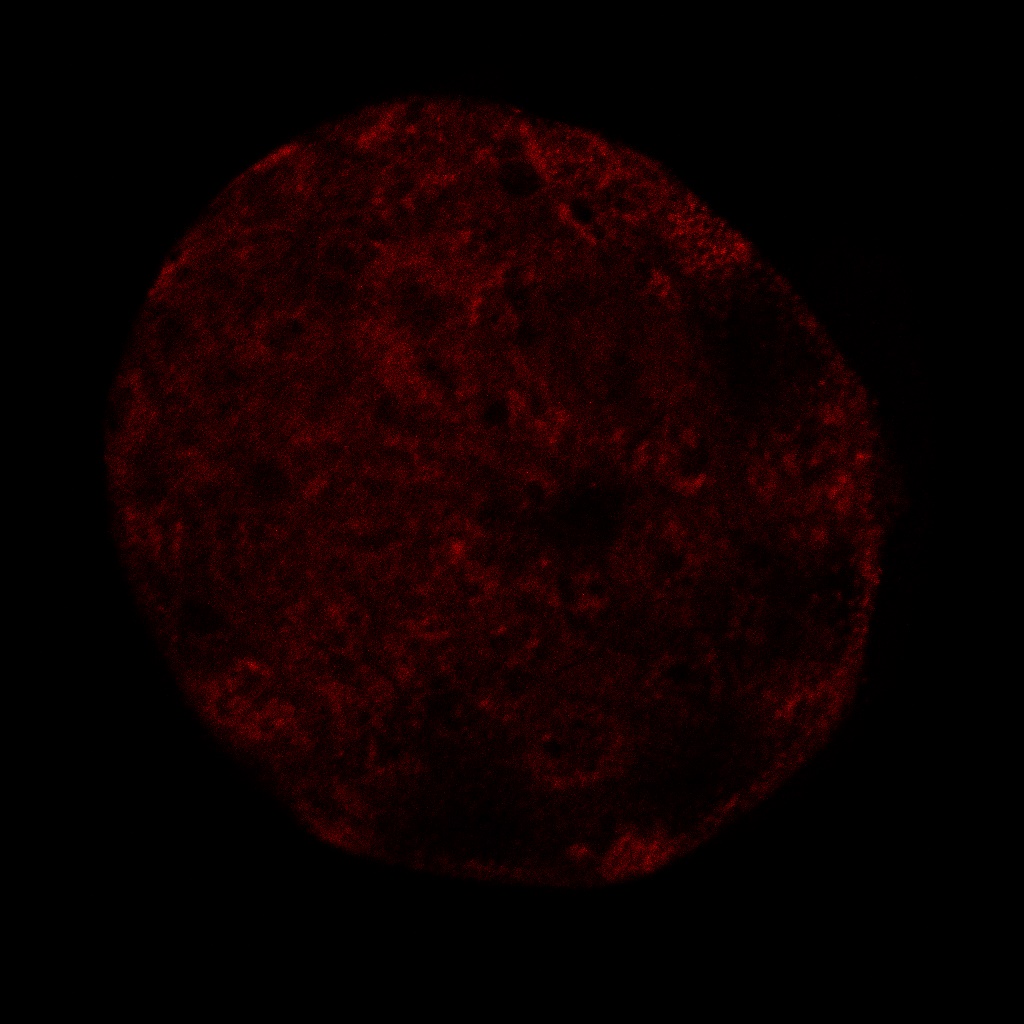

Supplement: Supplementary file 17 — Appendix Figure Source Data [file 44318_2024_329_MOESM17_ESM.zip › SD Appendix/FigS11B/S11B/Blastocyst_Setd1ab KD_SETD1B.jpg]

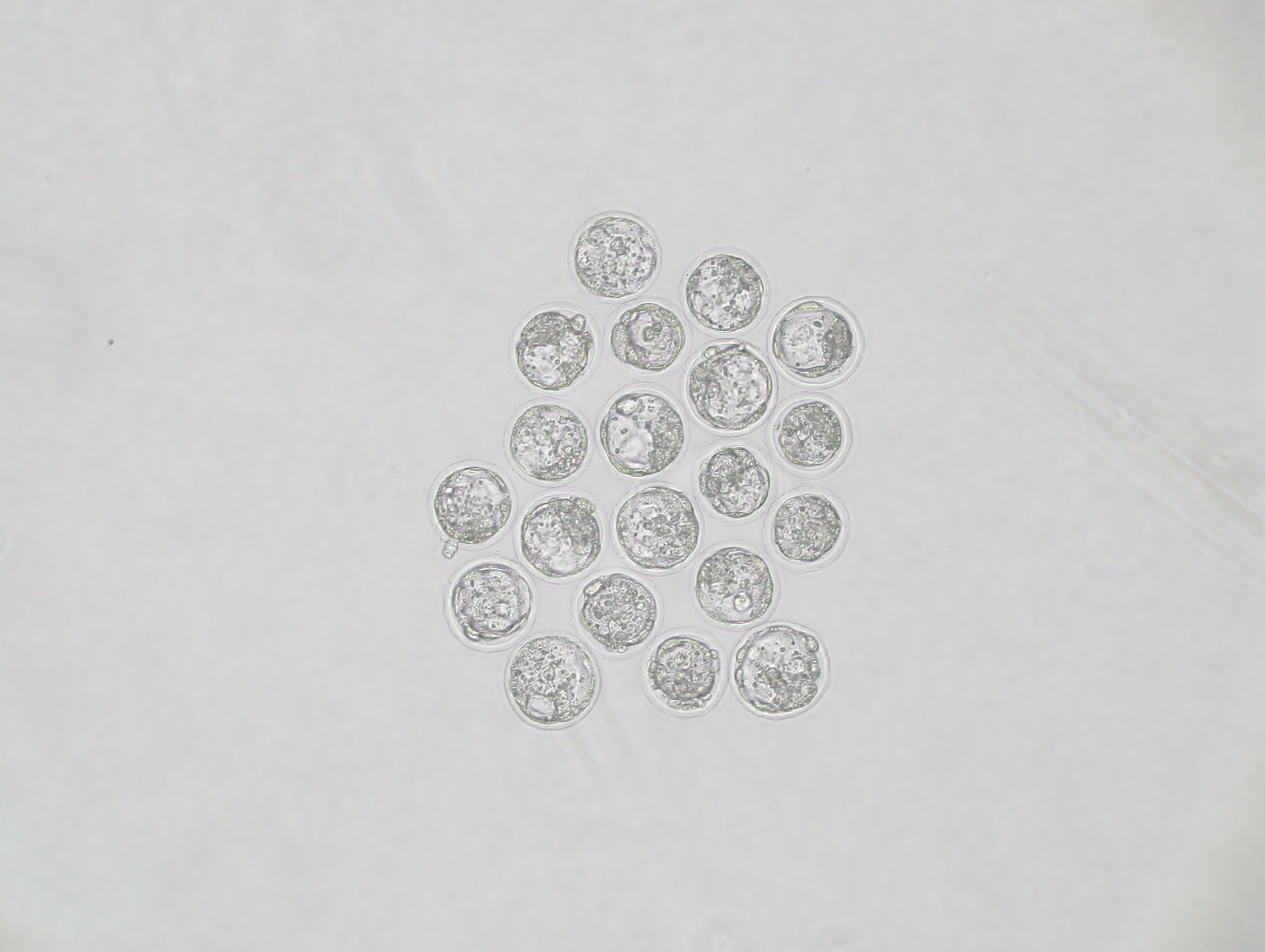

Supplement: Supplementary file 17 — Appendix Figure Source Data [file 44318_2024_329_MOESM17_ESM.zip › SD Appendix/FigS12A/Control.tif]

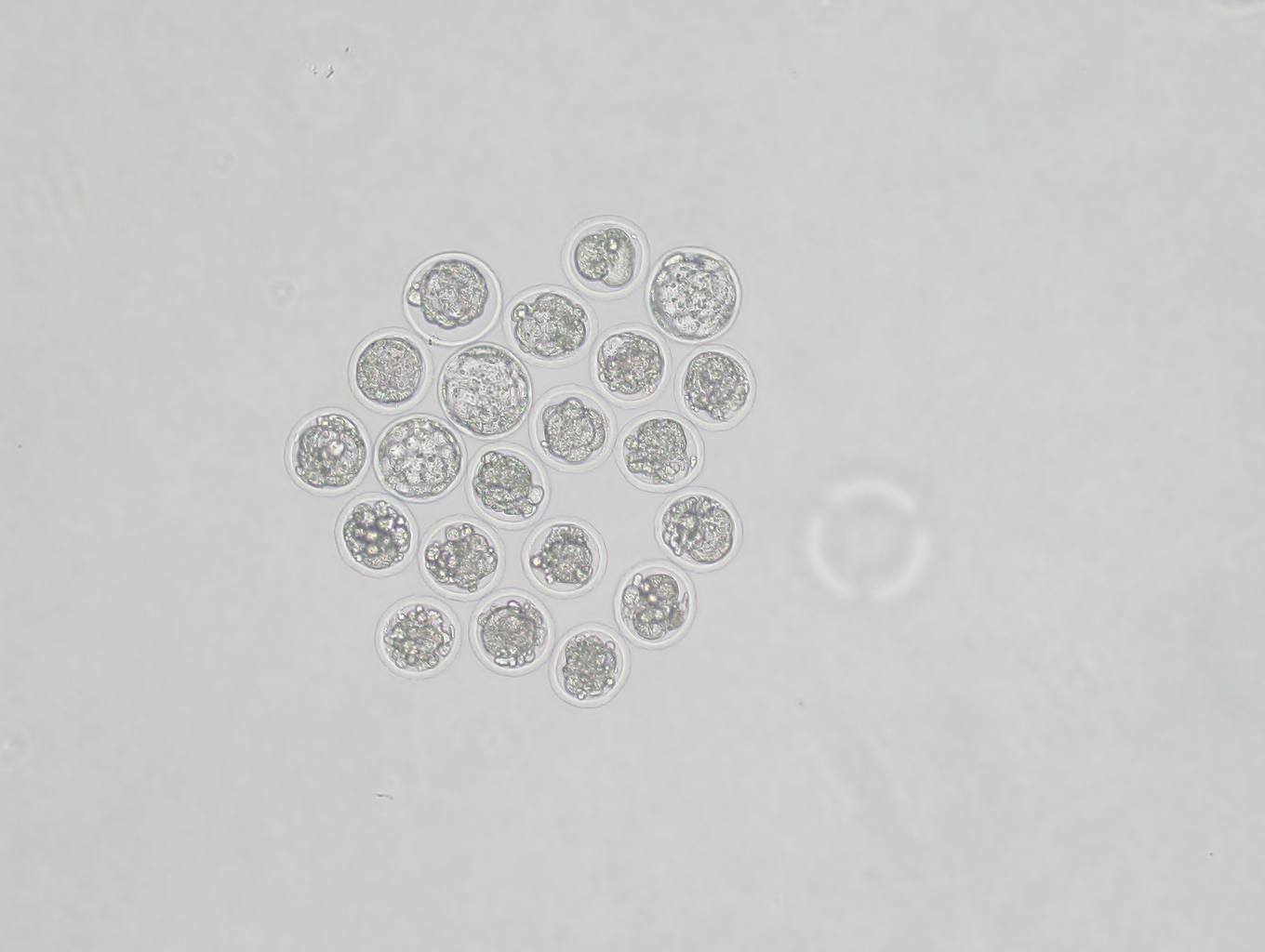

Supplement: Supplementary file 17 — Appendix Figure Source Data [file 44318_2024_329_MOESM17_ESM.zip › SD Appendix/FigS12A/Kdm5b KD.tif]

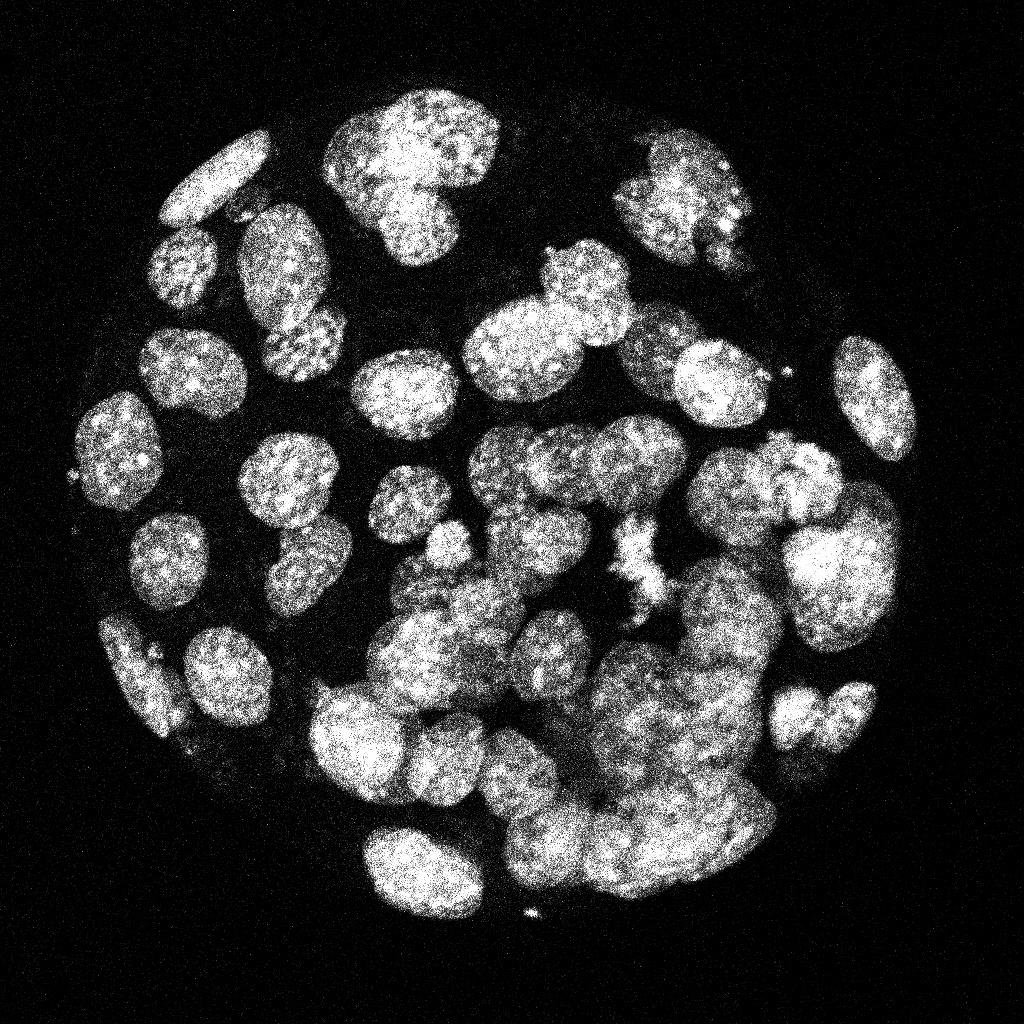

Supplement: Supplementary file 17 — Appendix Figure Source Data [file 44318_2024_329_MOESM17_ESM.zip › SD Appendix/FigS12C/S12C/Blastocyst_Control_DAPI.jpg]

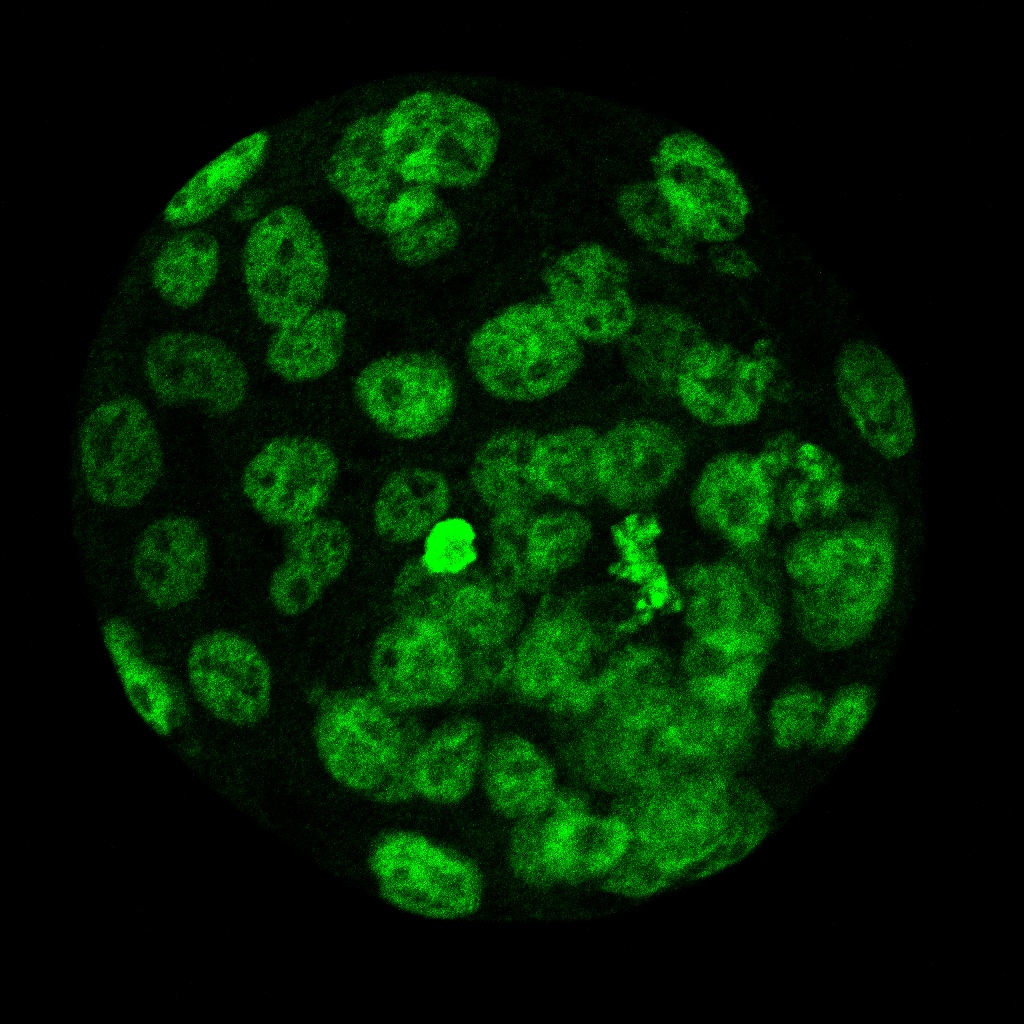

Supplement: Supplementary file 17 — Appendix Figure Source Data [file 44318_2024_329_MOESM17_ESM.zip › SD Appendix/FigS12C/S12C/Blastocyst_Control_H3K4me3.jpg]

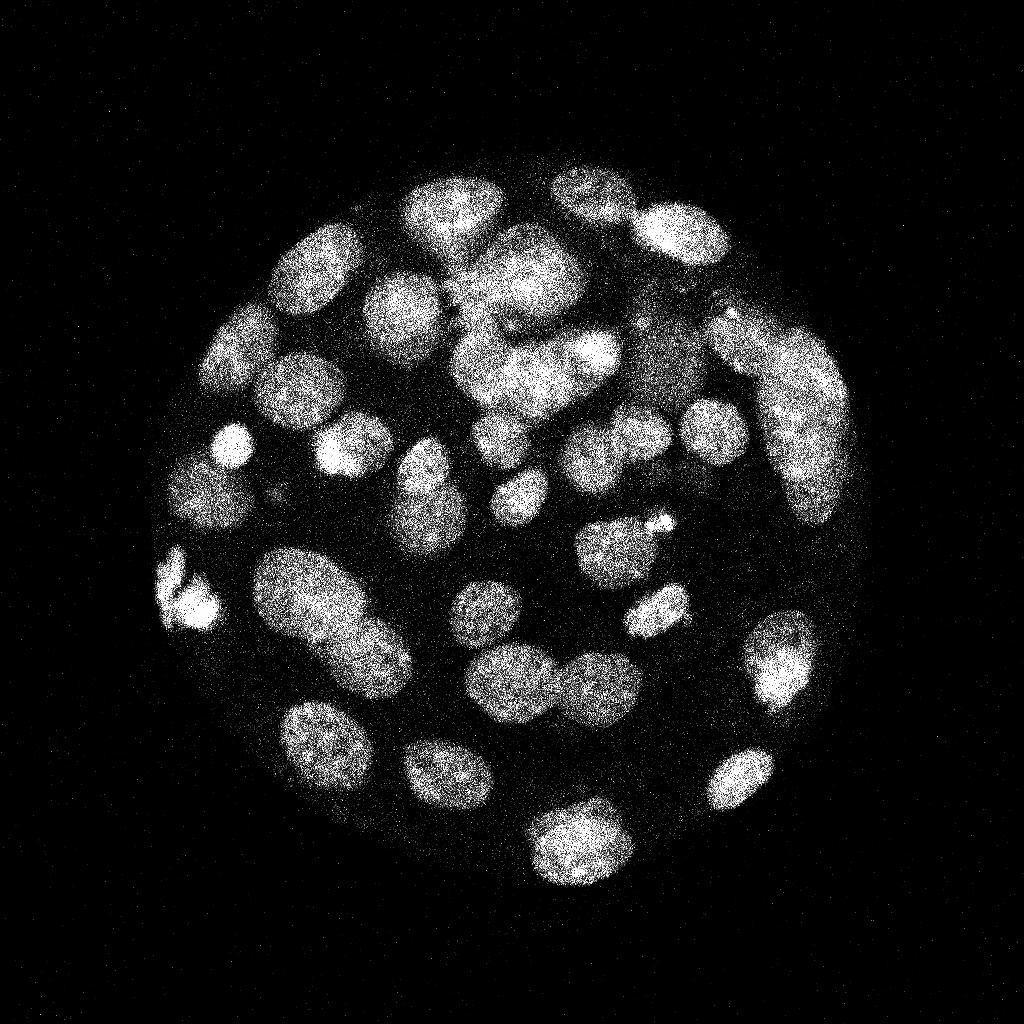

Supplement: Supplementary file 17 — Appendix Figure Source Data [file 44318_2024_329_MOESM17_ESM.zip › SD Appendix/FigS12C/S12C/Blastocyst_Kdm5b KD_DAPI.jpg]

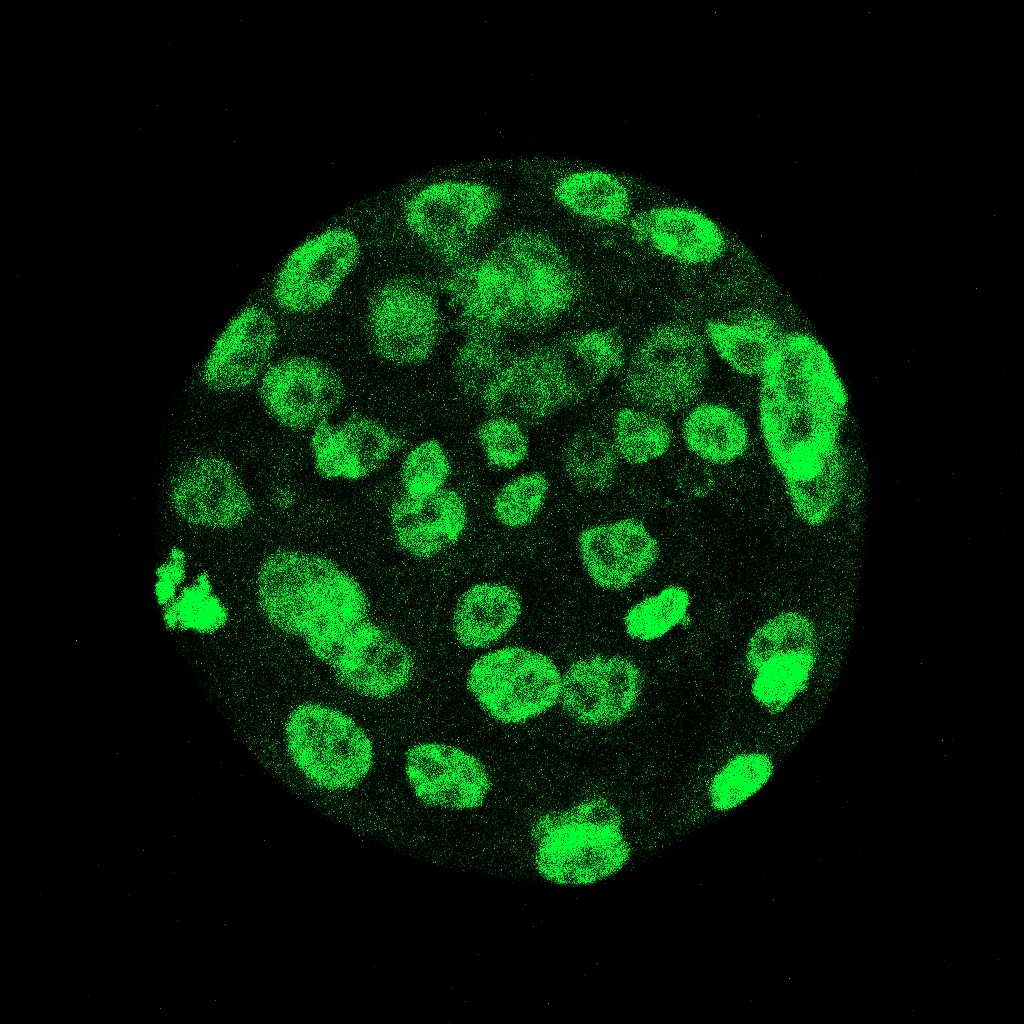

Supplement: Supplementary file 17 — Appendix Figure Source Data [file 44318_2024_329_MOESM17_ESM.zip › SD Appendix/FigS12C/S12C/Blastocyst_Kdm5b KD_H3K4me3.jpg]

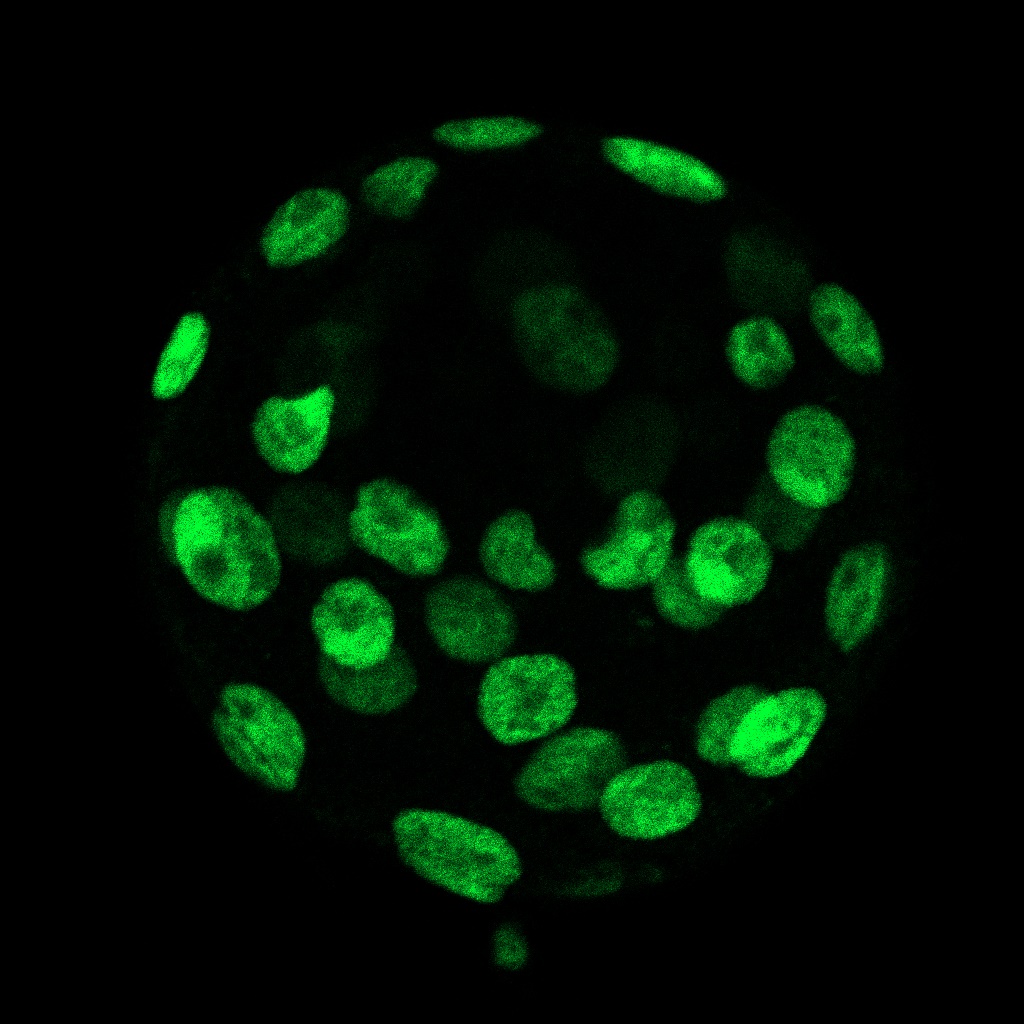

Supplement: Supplementary file 17 — Appendix Figure Source Data [file 44318_2024_329_MOESM17_ESM.zip › SD Appendix/FigS12E/S12E/Blastocyst_Control_CDX2.jpg]

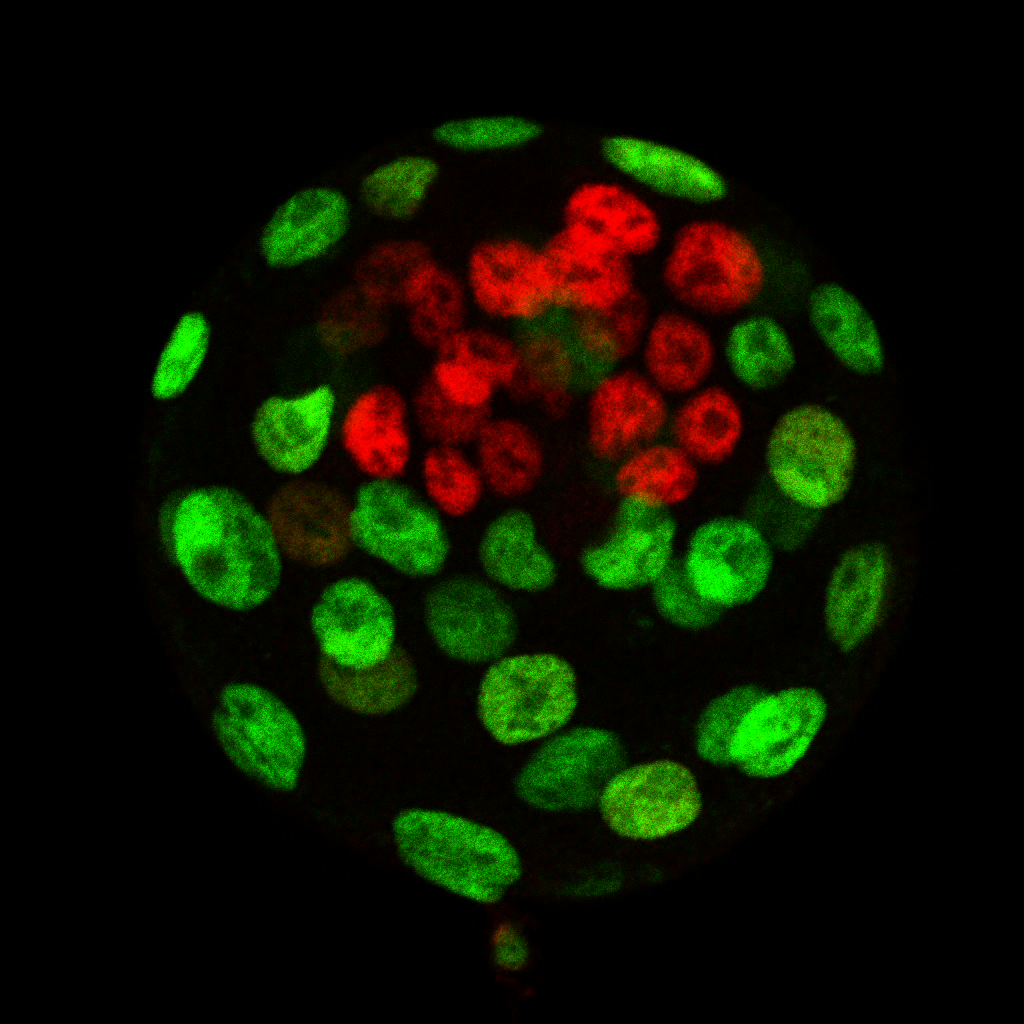

Supplement: Supplementary file 17 — Appendix Figure Source Data [file 44318_2024_329_MOESM17_ESM.zip › SD Appendix/FigS12E/S12E/Blastocyst_Control_Merge.jpg]

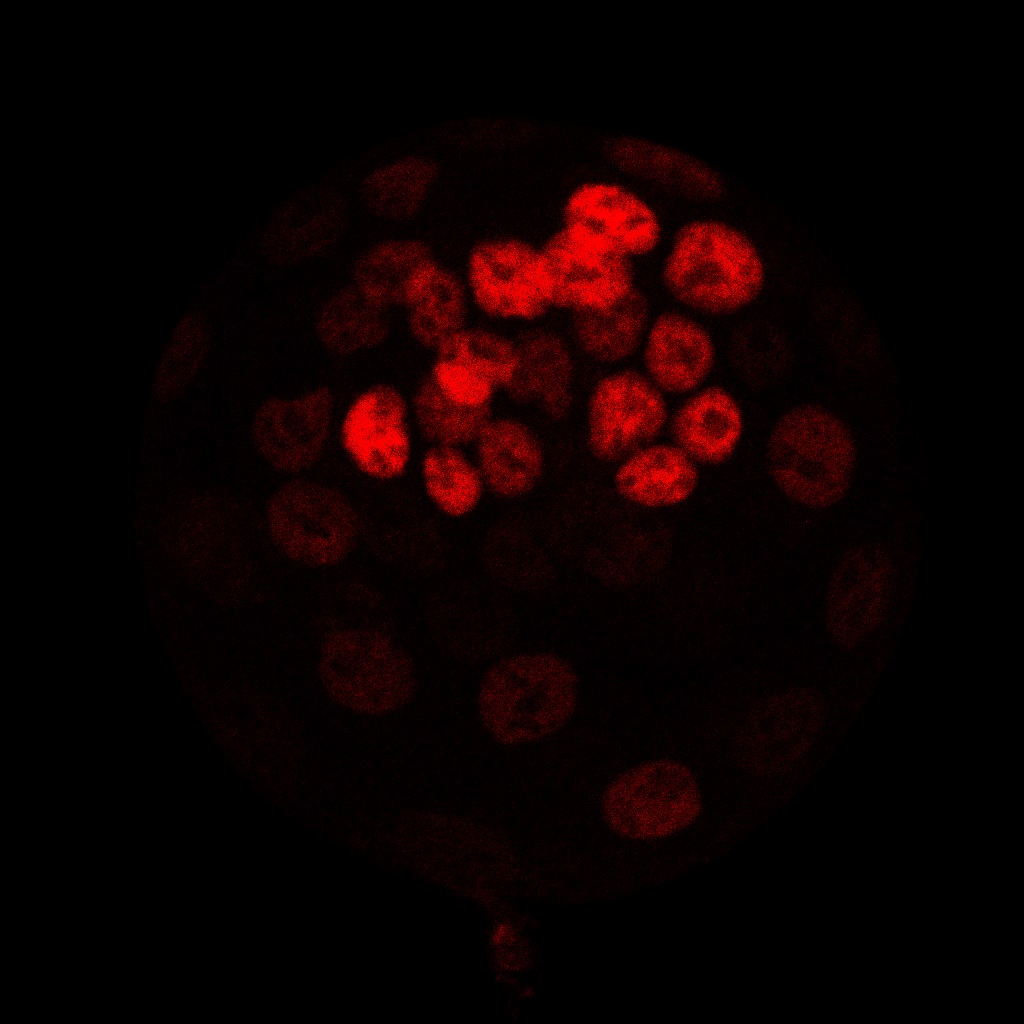

Supplement: Supplementary file 17 — Appendix Figure Source Data [file 44318_2024_329_MOESM17_ESM.zip › SD Appendix/FigS12E/S12E/Blastocyst_Control_NANOG.jpg]

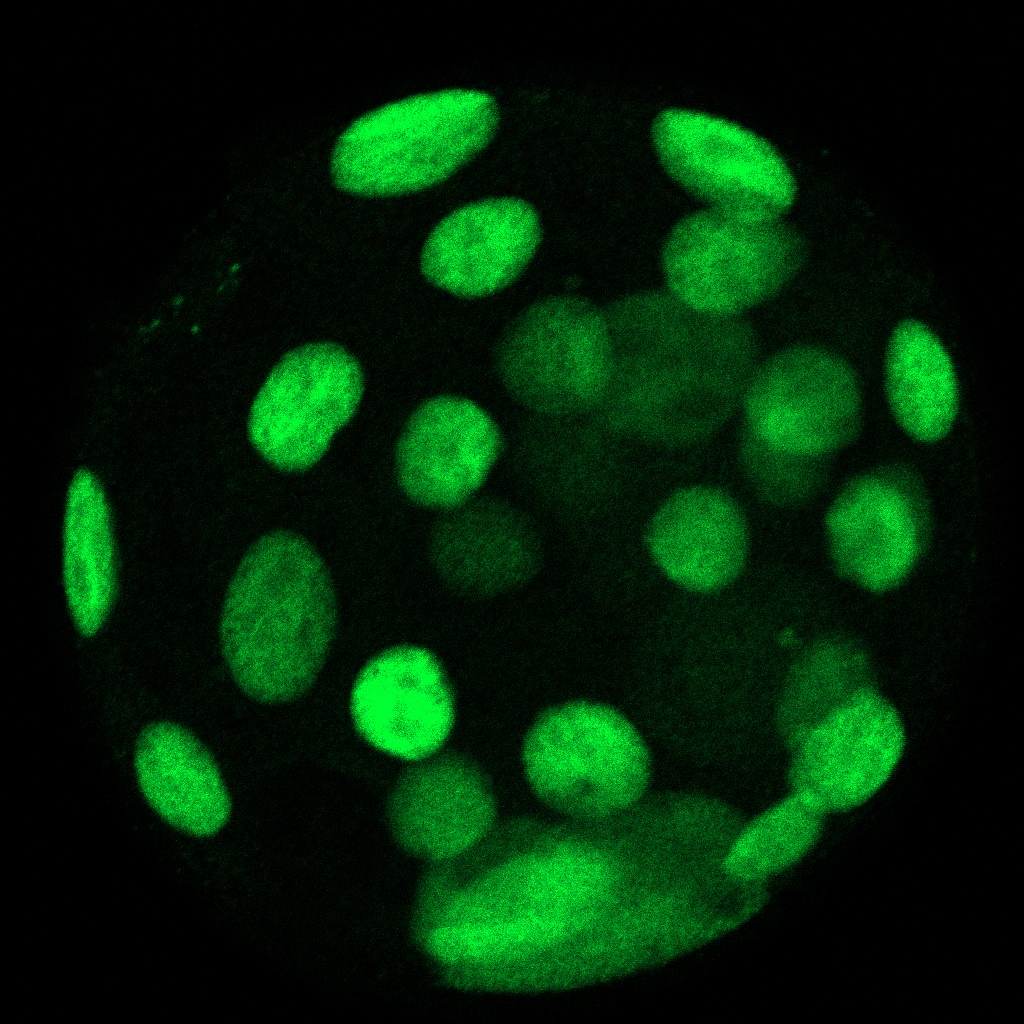

Supplement: Supplementary file 17 — Appendix Figure Source Data [file 44318_2024_329_MOESM17_ESM.zip › SD Appendix/FigS12E/S12E/Blastocyst_Kdm5b KD_CDX2.jpg]

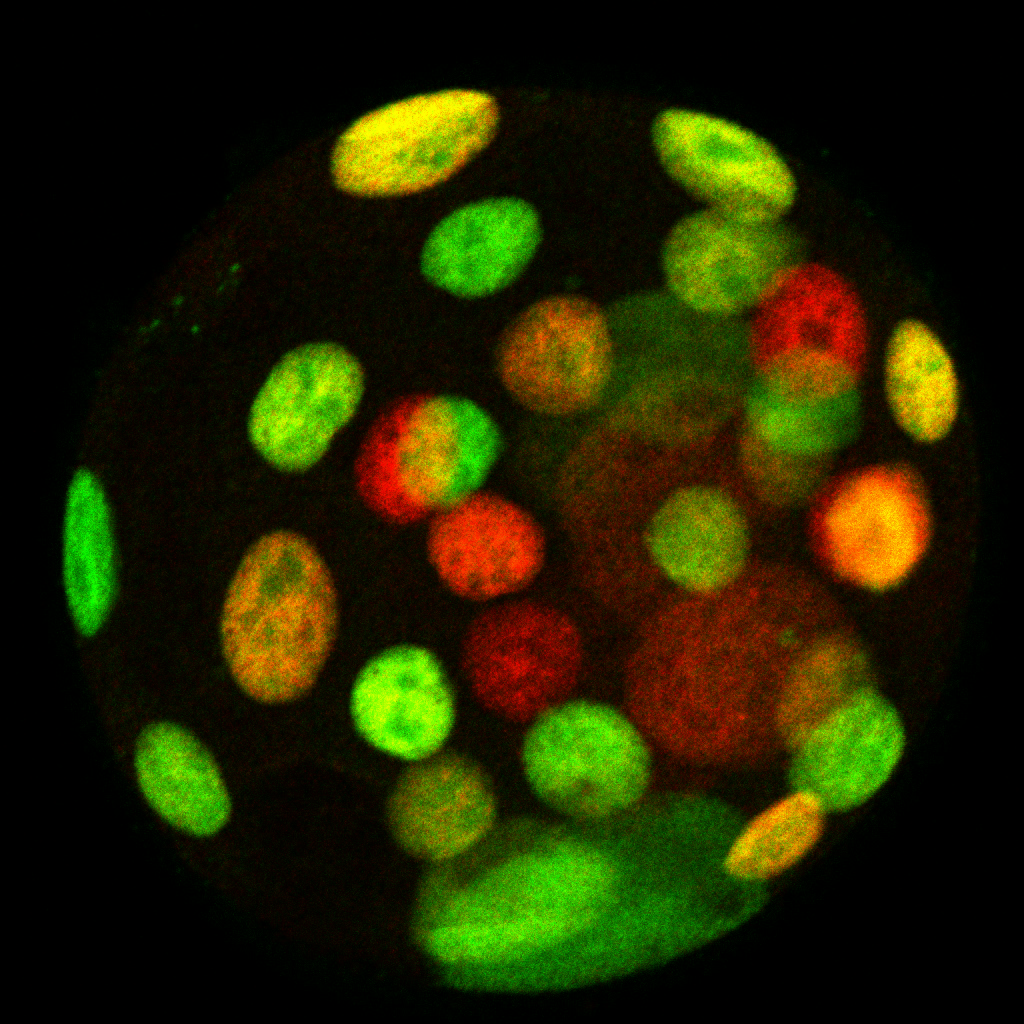

Supplement: Supplementary file 17 — Appendix Figure Source Data [file 44318_2024_329_MOESM17_ESM.zip › SD Appendix/FigS12E/S12E/Blastocyst_Kdm5b KD_Merge.jpg]

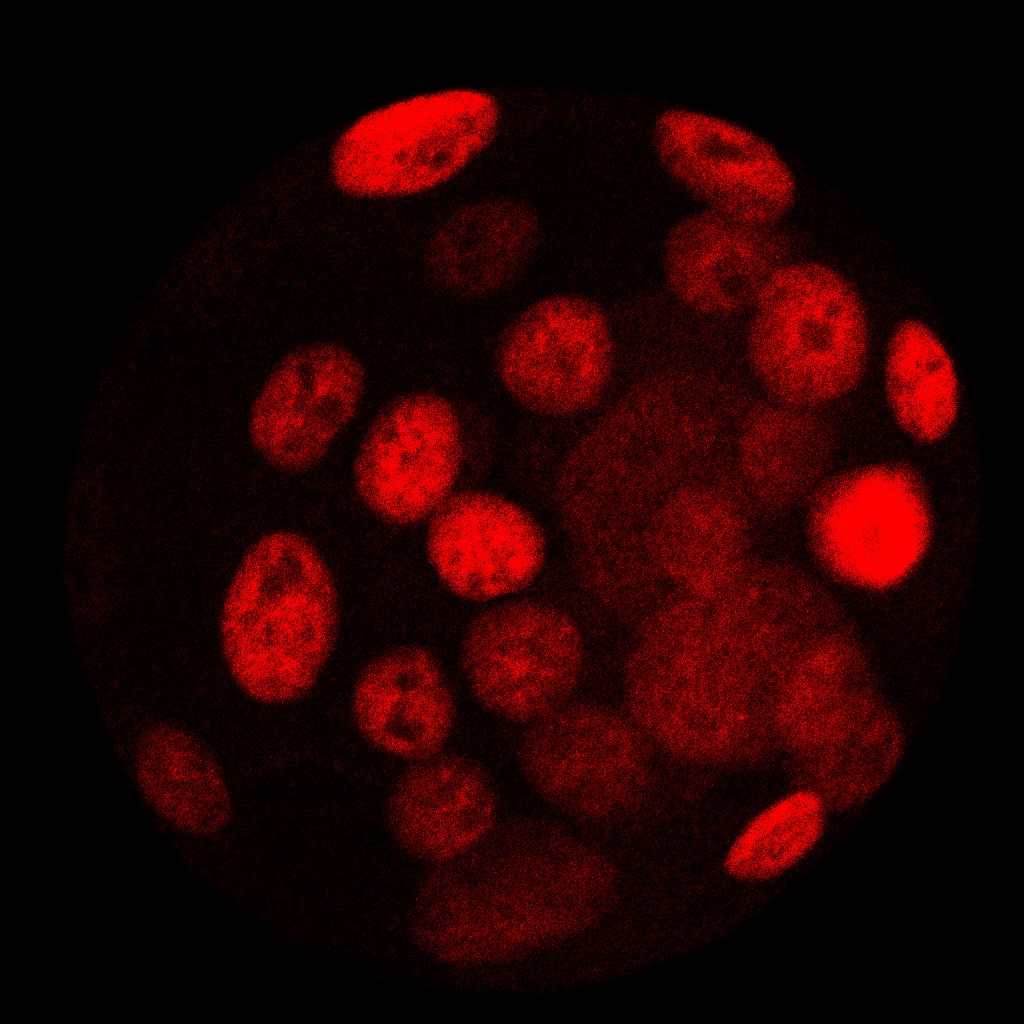

Supplement: Supplementary file 17 — Appendix Figure Source Data [file 44318_2024_329_MOESM17_ESM.zip › SD Appendix/FigS12E/S12E/Blastocyst_Kdm5b KD_NANOG.jpg]

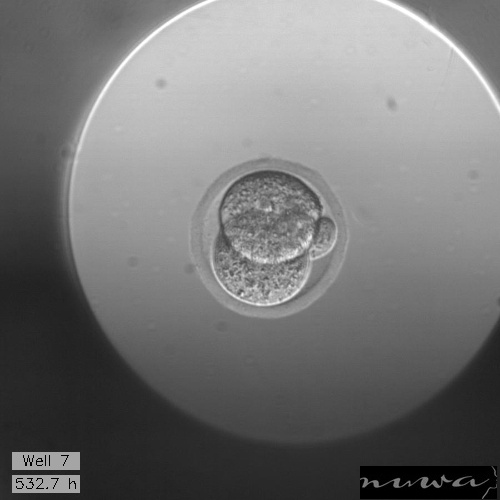

Supplement: Supplementary file 17 — Appendix Figure Source Data [file 44318_2024_329_MOESM17_ESM.zip › SD Appendix/FigS13B/Control_2-cell.jpg]

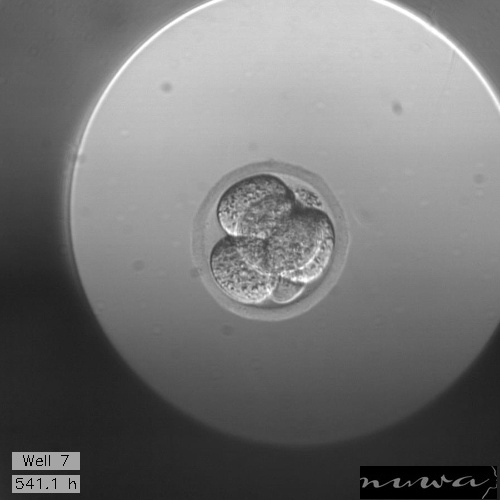

Supplement: Supplementary file 17 — Appendix Figure Source Data [file 44318_2024_329_MOESM17_ESM.zip › SD Appendix/FigS13B/Control_4-cell.jpg]

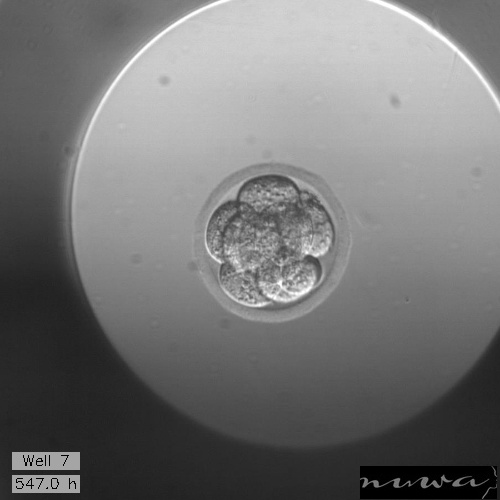

Supplement: Supplementary file 17 — Appendix Figure Source Data [file 44318_2024_329_MOESM17_ESM.zip › SD Appendix/FigS13B/Control_8-cell.jpg]

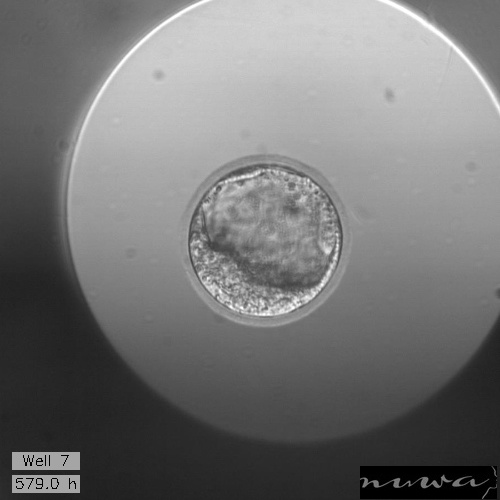

Supplement: Supplementary file 17 — Appendix Figure Source Data [file 44318_2024_329_MOESM17_ESM.zip › SD Appendix/FigS13B/Control_Blastocyst.jpg]

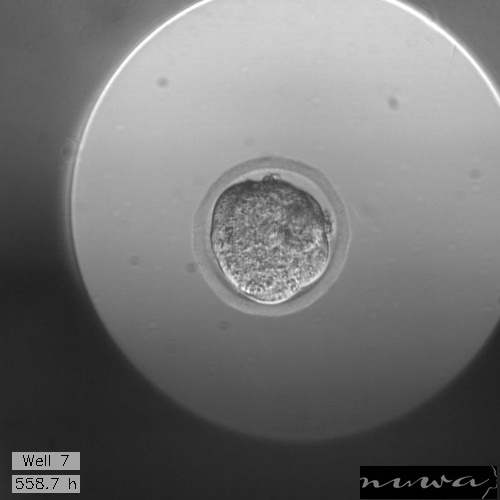

Supplement: Supplementary file 17 — Appendix Figure Source Data [file 44318_2024_329_MOESM17_ESM.zip › SD Appendix/FigS13B/Control_Morula.jpg]

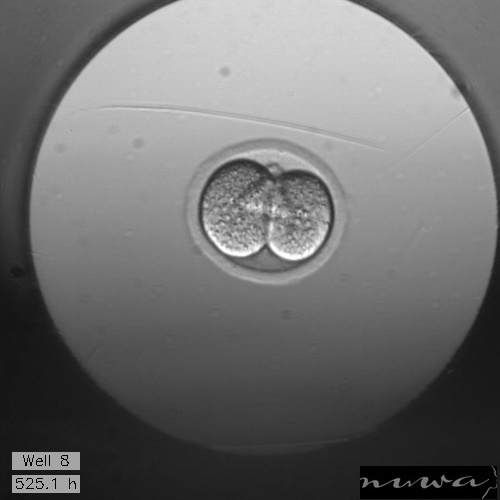

Supplement: Supplementary file 17 — Appendix Figure Source Data [file 44318_2024_329_MOESM17_ESM.zip › SD Appendix/FigS13B/Kdm5b KD+MUT mRNA_2-cell.jpg]

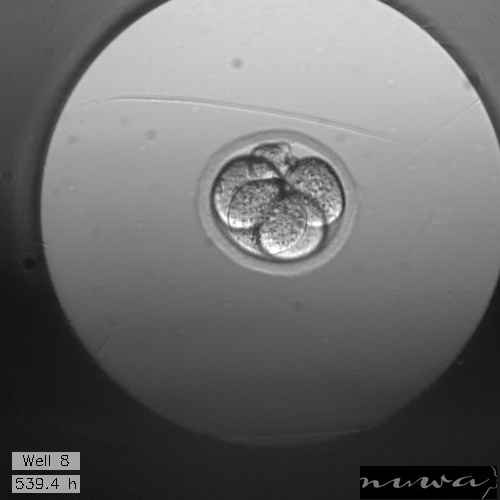

Supplement: Supplementary file 17 — Appendix Figure Source Data [file 44318_2024_329_MOESM17_ESM.zip › SD Appendix/FigS13B/Kdm5b KD+MUT mRNA_4-cell.jpg]

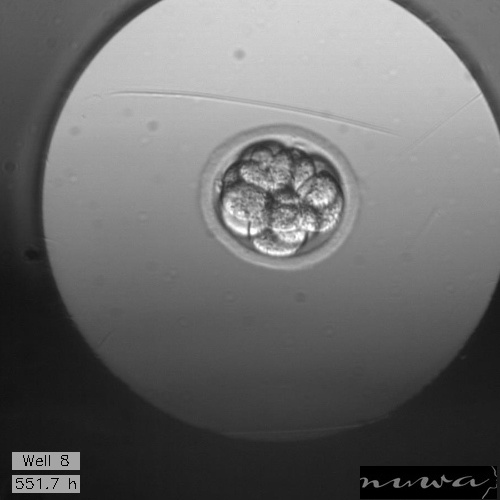

Supplement: Supplementary file 17 — Appendix Figure Source Data [file 44318_2024_329_MOESM17_ESM.zip › SD Appendix/FigS13B/Kdm5b KD+MUT mRNA_8-cell.jpg]

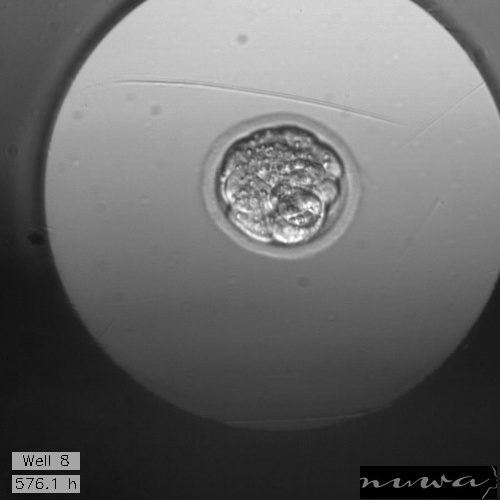

Supplement: Supplementary file 17 — Appendix Figure Source Data [file 44318_2024_329_MOESM17_ESM.zip › SD Appendix/FigS13B/Kdm5b KD+MUT mRNA_Blastocyst.jpg]

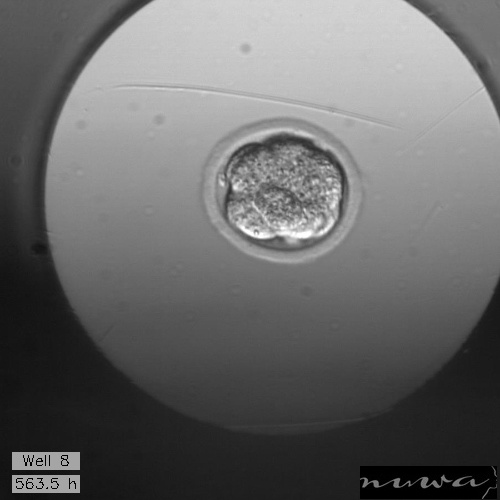

Supplement: Supplementary file 17 — Appendix Figure Source Data [file 44318_2024_329_MOESM17_ESM.zip › SD Appendix/FigS13B/Kdm5b KD+MUT mRNA_Morula.jpg]

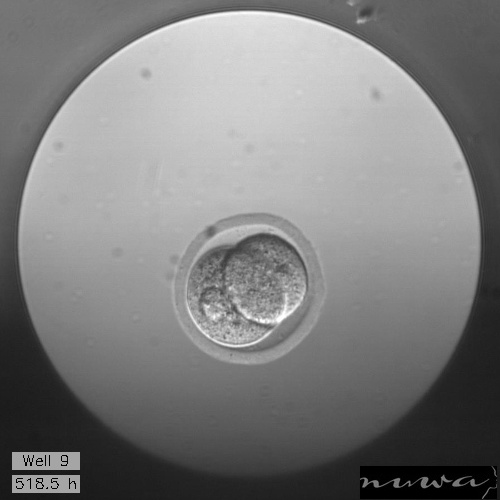

Supplement: Supplementary file 17 — Appendix Figure Source Data [file 44318_2024_329_MOESM17_ESM.zip › SD Appendix/FigS13B/Kdm5b KD+WT mRNA_2-cell.jpg]

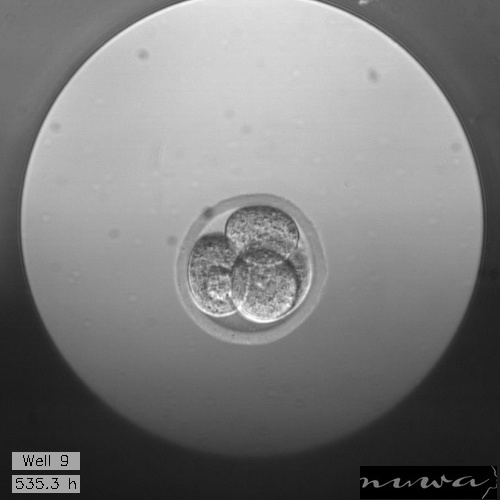

Supplement: Supplementary file 17 — Appendix Figure Source Data [file 44318_2024_329_MOESM17_ESM.zip › SD Appendix/FigS13B/Kdm5b KD+WT mRNA_4-cell.jpg]

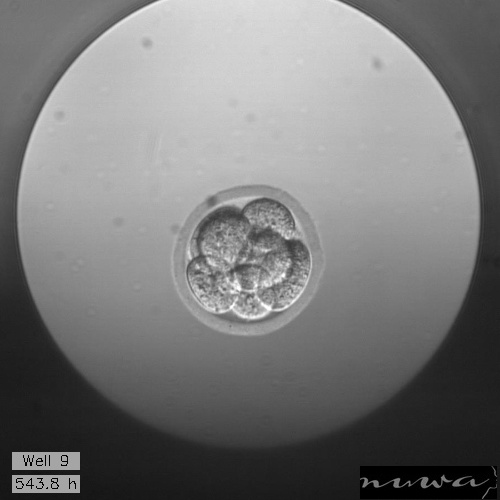

Supplement: Supplementary file 17 — Appendix Figure Source Data [file 44318_2024_329_MOESM17_ESM.zip › SD Appendix/FigS13B/Kdm5b KD+WT mRNA_8-cell.jpg]

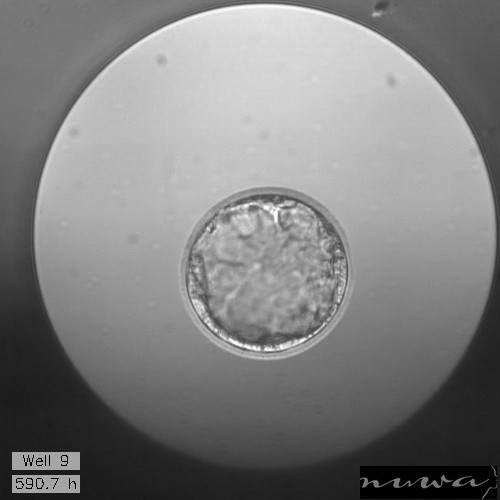

Supplement: Supplementary file 17 — Appendix Figure Source Data [file 44318_2024_329_MOESM17_ESM.zip › SD Appendix/FigS13B/Kdm5b KD+WT mRNA_Blastocyst.jpg]

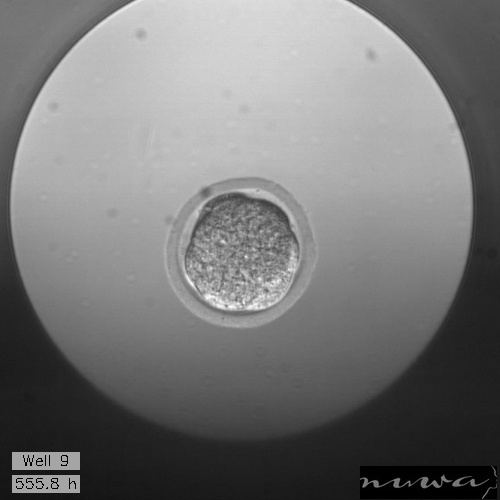

Supplement: Supplementary file 17 — Appendix Figure Source Data [file 44318_2024_329_MOESM17_ESM.zip › SD Appendix/FigS13B/Kdm5b KD+WT mRNA_Morula.jpg]

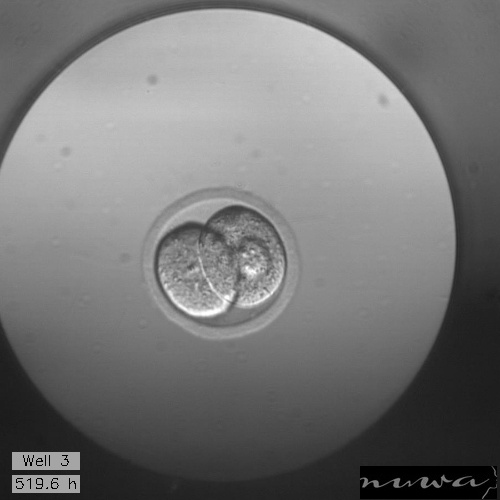

Supplement: Supplementary file 17 — Appendix Figure Source Data [file 44318_2024_329_MOESM17_ESM.zip › SD Appendix/FigS13B/Kdm5b KD_2-cell.jpg]

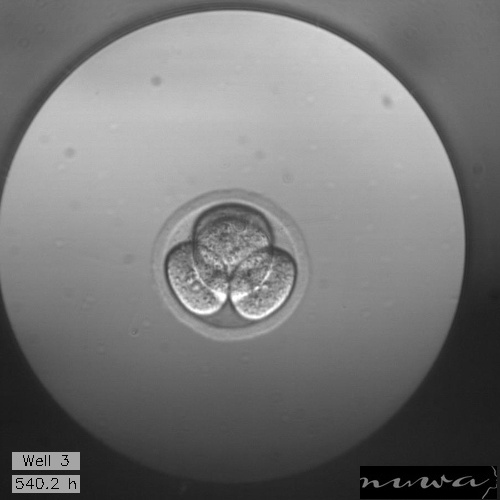

Supplement: Supplementary file 17 — Appendix Figure Source Data [file 44318_2024_329_MOESM17_ESM.zip › SD Appendix/FigS13B/Kdm5b KD_4-cell.jpg]

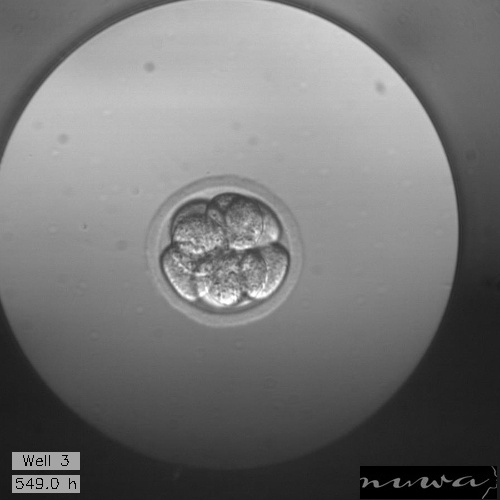

Supplement: Supplementary file 17 — Appendix Figure Source Data [file 44318_2024_329_MOESM17_ESM.zip › SD Appendix/FigS13B/Kdm5b KD_8-cell.jpg]

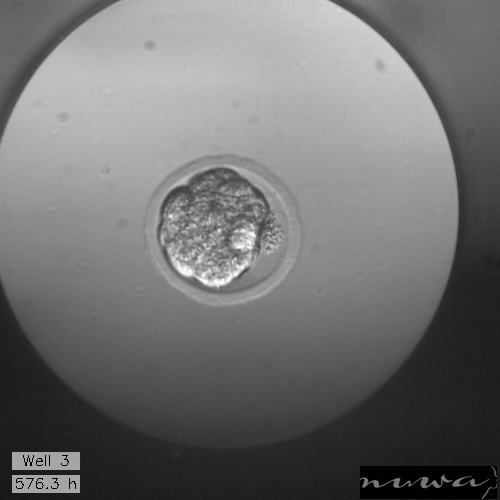

Supplement: Supplementary file 17 — Appendix Figure Source Data [file 44318_2024_329_MOESM17_ESM.zip › SD Appendix/FigS13B/Kdm5b KD_Blastocyst.jpg]

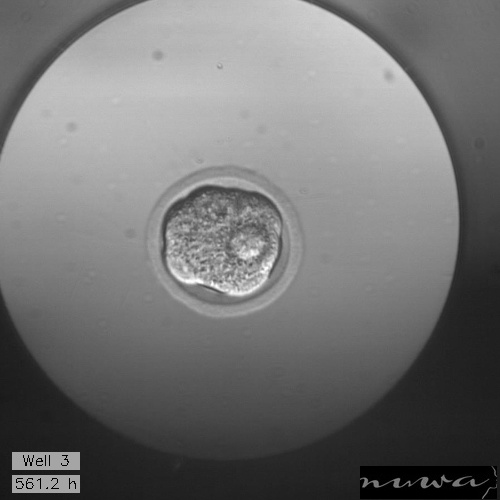

Supplement: Supplementary file 17 — Appendix Figure Source Data [file 44318_2024_329_MOESM17_ESM.zip › SD Appendix/FigS13B/Kdm5b KD_Morula.jpg]

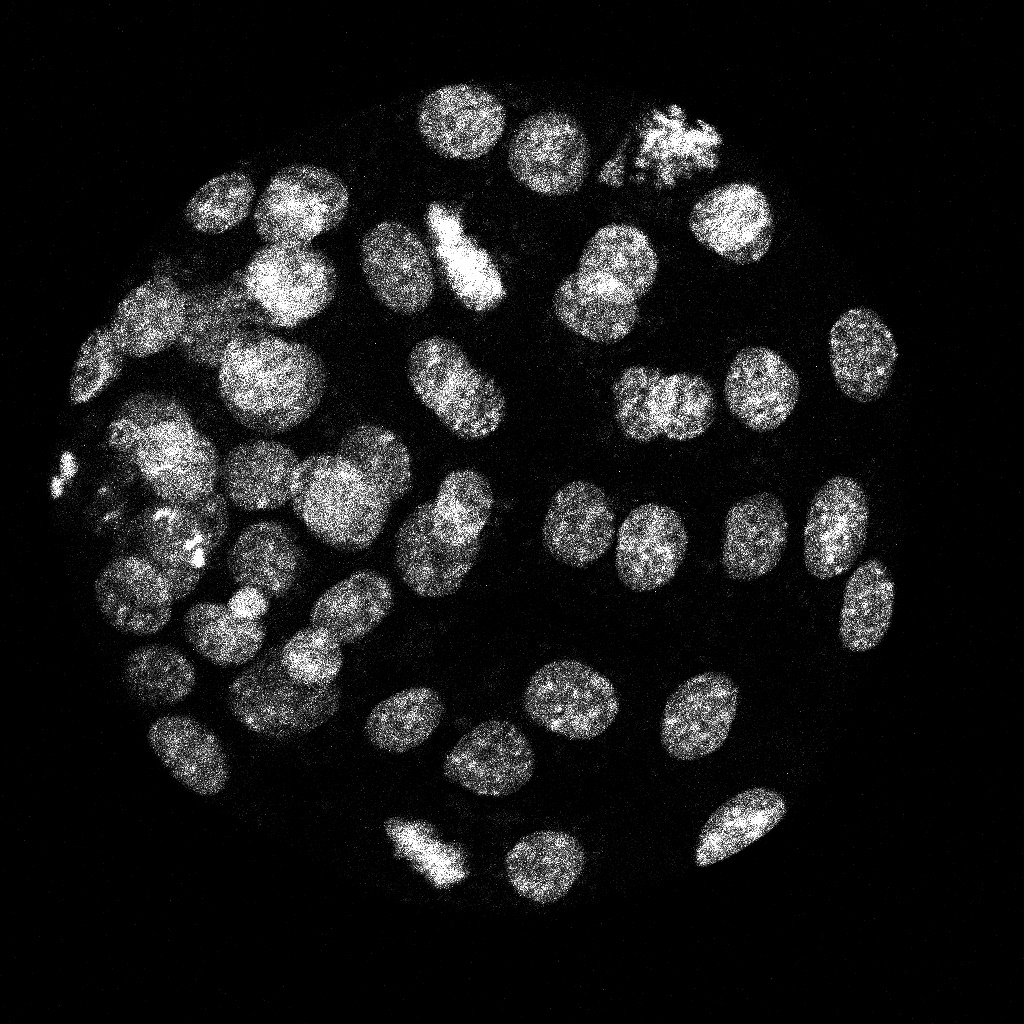

Supplement: Supplementary file 17 — Appendix Figure Source Data [file 44318_2024_329_MOESM17_ESM.zip › SD Appendix/FigS1F/S1F/Blastocyst_Control_DAPI.jpg]

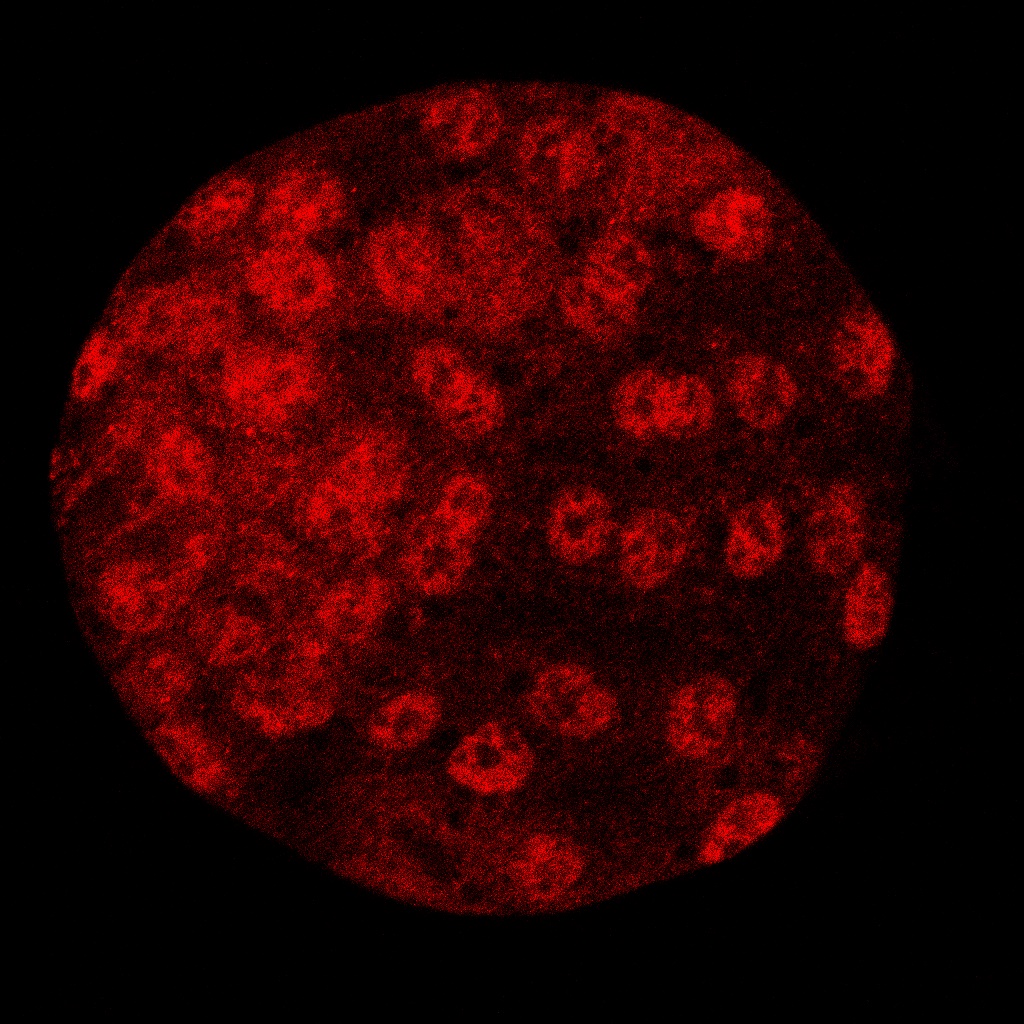

Supplement: Supplementary file 17 — Appendix Figure Source Data [file 44318_2024_329_MOESM17_ESM.zip › SD Appendix/FigS1F/S1F/Blastocyst_Control_MLL2.jpg]

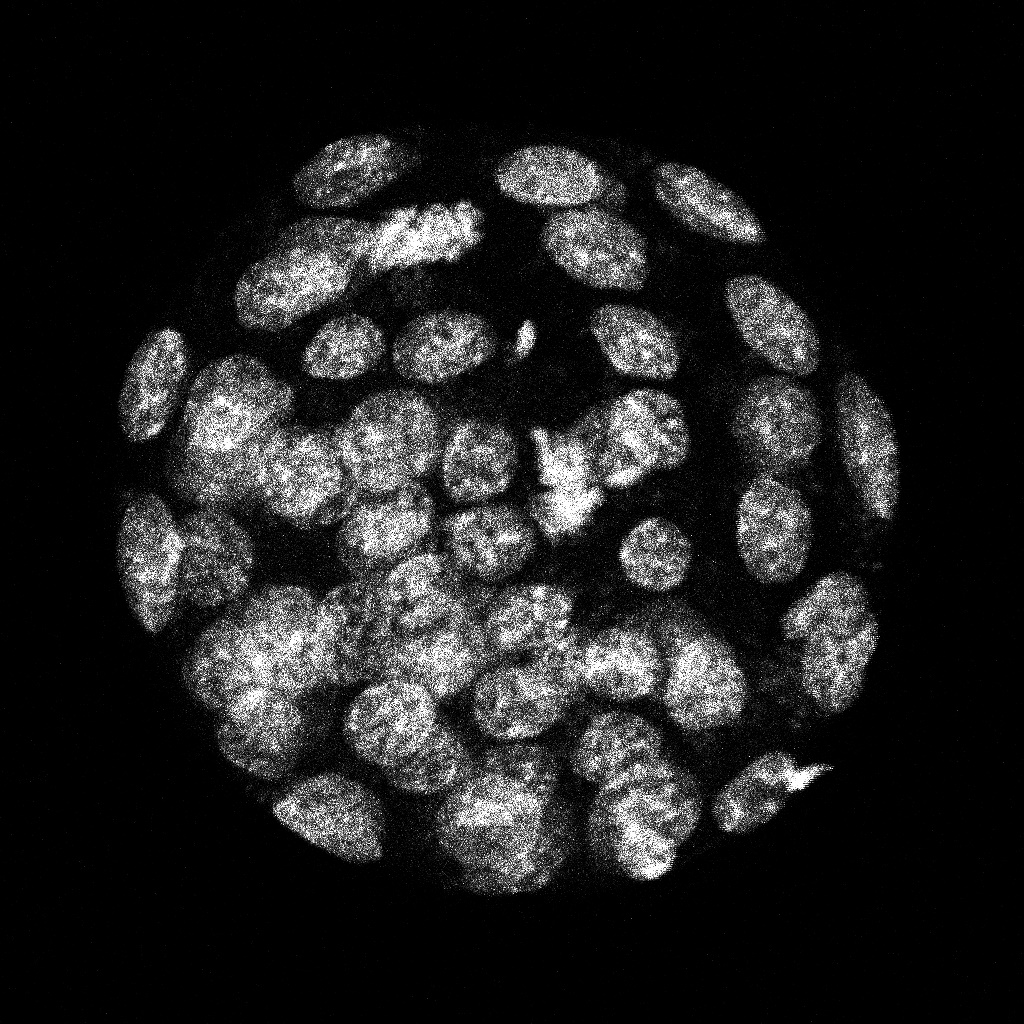

Supplement: Supplementary file 17 — Appendix Figure Source Data [file 44318_2024_329_MOESM17_ESM.zip › SD Appendix/FigS1F/S1F/Blastocyst_Mll2 KD_DAPI.jpg]

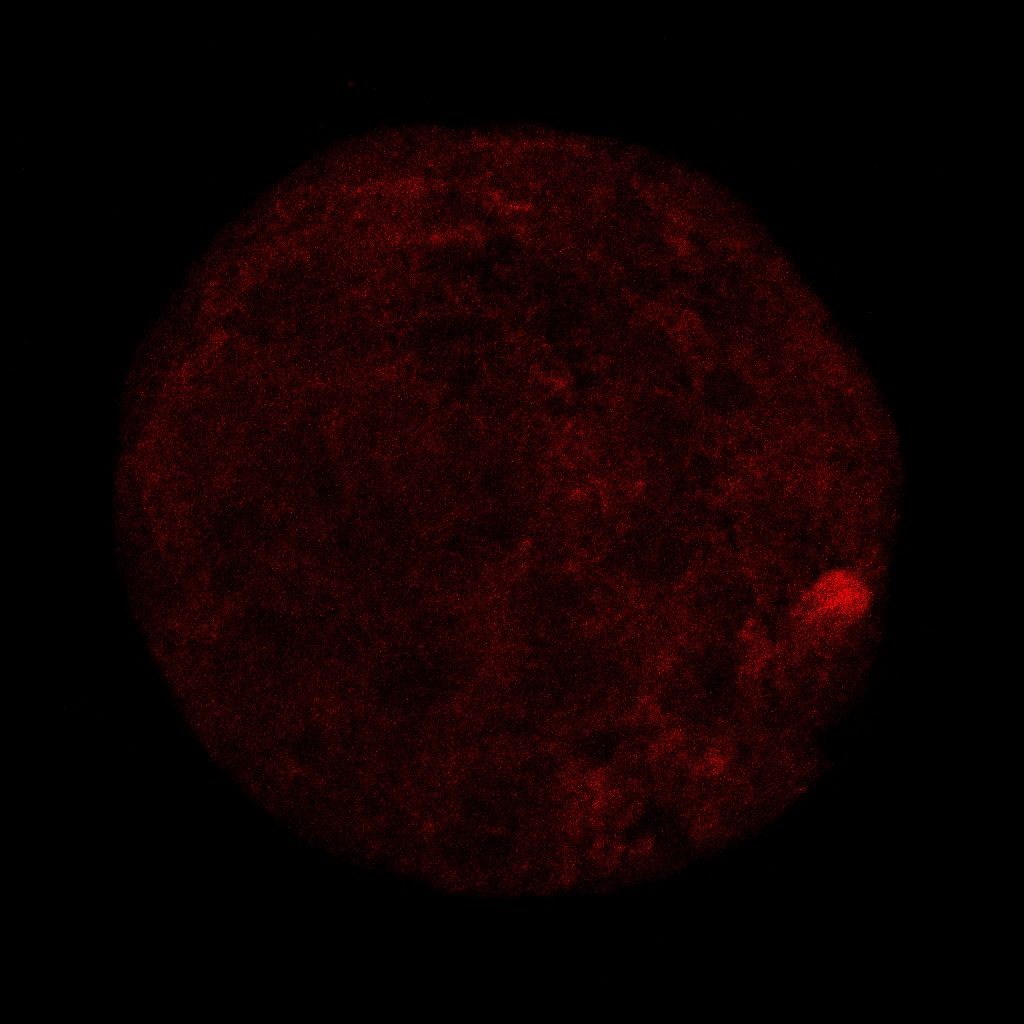

Supplement: Supplementary file 17 — Appendix Figure Source Data [file 44318_2024_329_MOESM17_ESM.zip › SD Appendix/FigS1F/S1F/Blastocyst_Mll2 KD_MLL2.jpg]

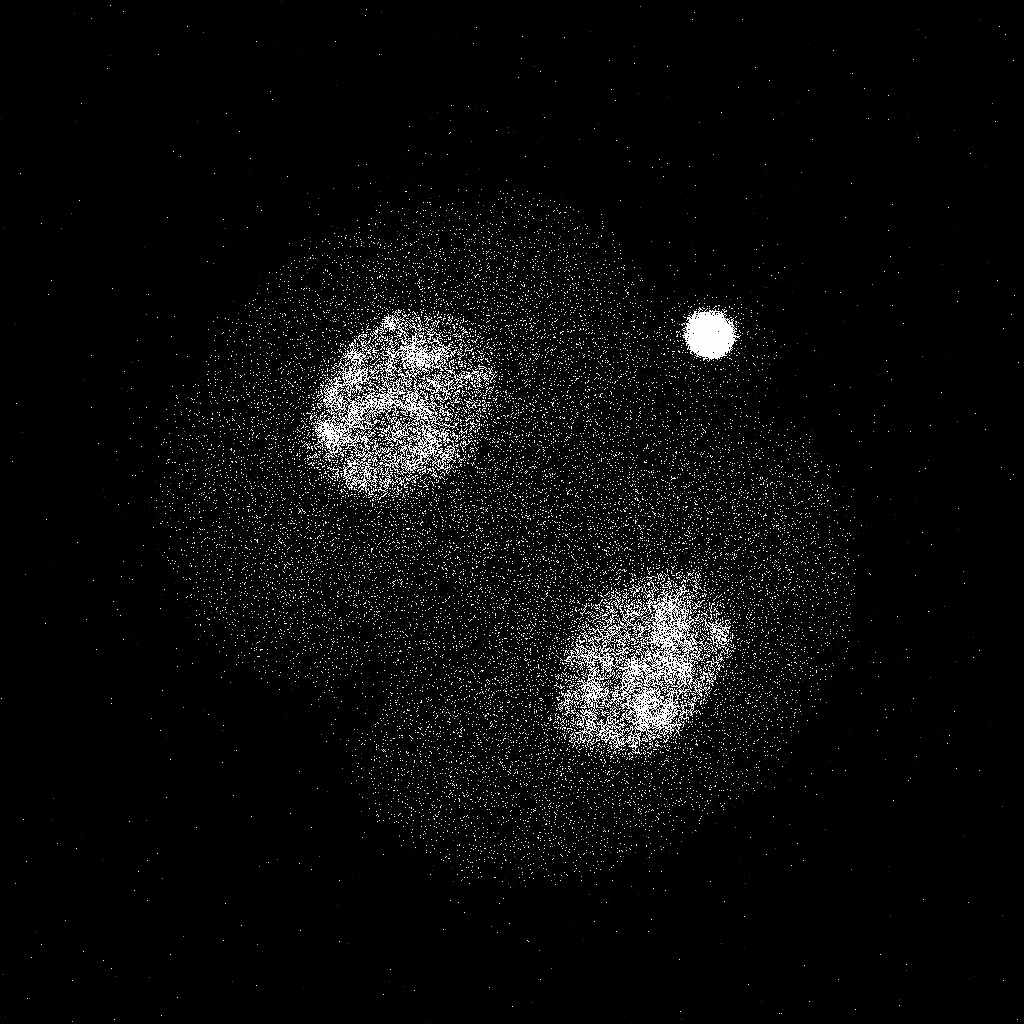

Supplement: Supplementary file 17 — Appendix Figure Source Data [file 44318_2024_329_MOESM17_ESM.zip › SD Appendix/FigS1F/S1F/Late2C_Control_DAPI.jpg]

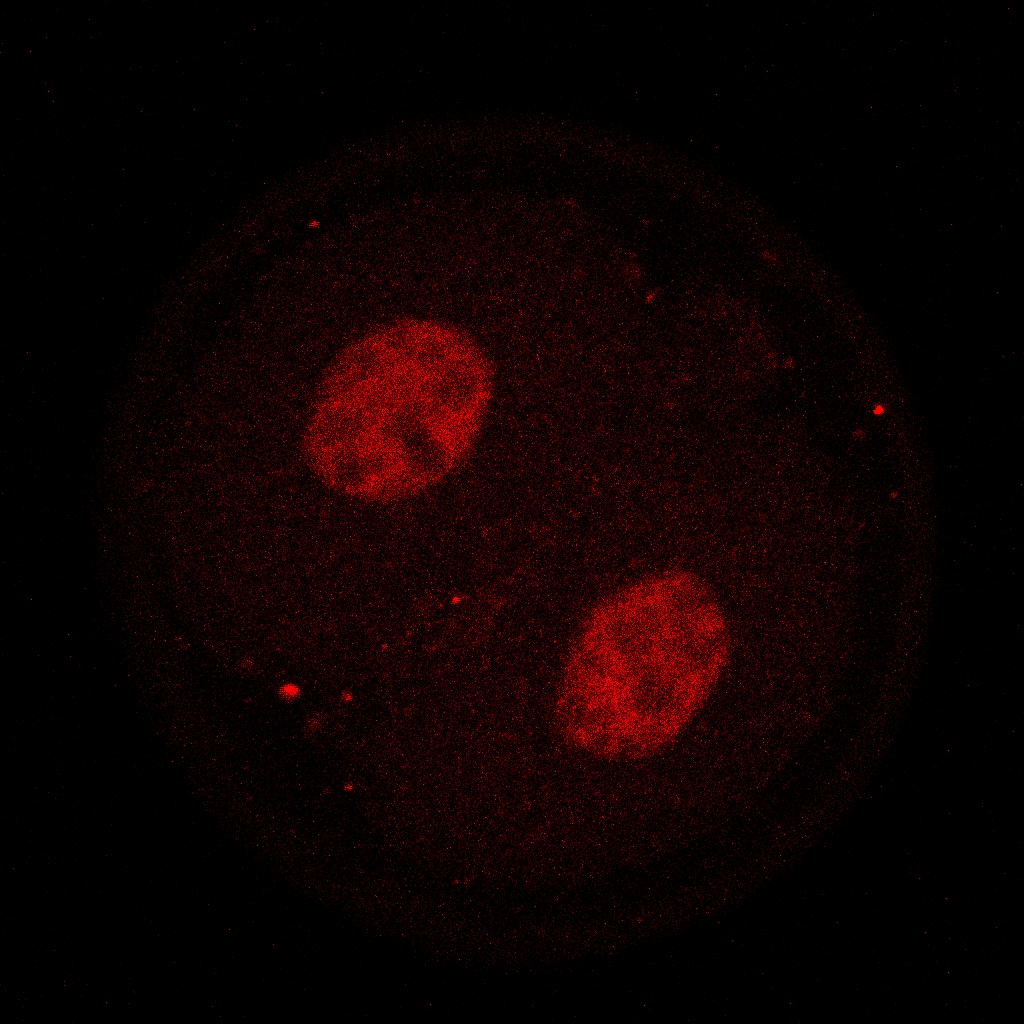

Supplement: Supplementary file 17 — Appendix Figure Source Data [file 44318_2024_329_MOESM17_ESM.zip › SD Appendix/FigS1F/S1F/Late2C_Control_MLL2.jpg]

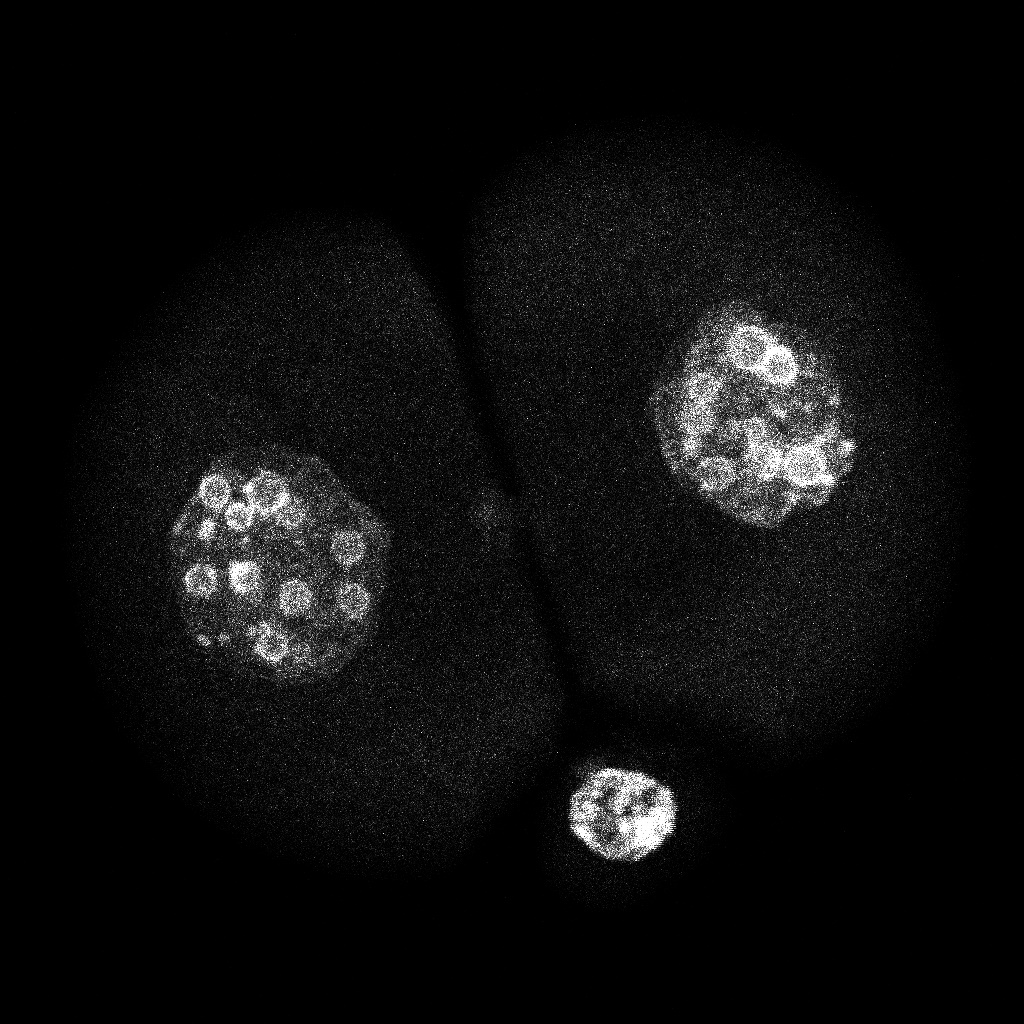

Supplement: Supplementary file 17 — Appendix Figure Source Data [file 44318_2024_329_MOESM17_ESM.zip › SD Appendix/FigS1F/S1F/Late2C_Mll2 KD_DAPI.jpg]

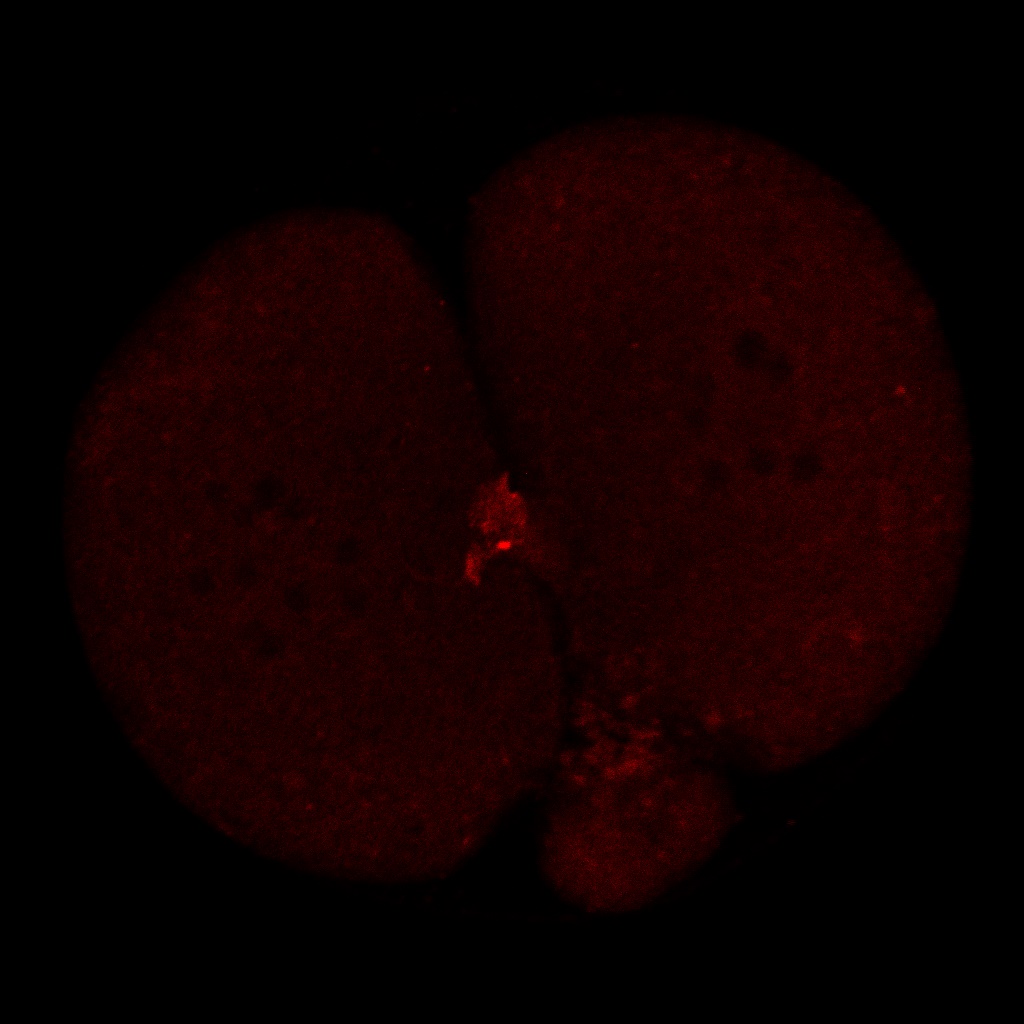

Supplement: Supplementary file 17 — Appendix Figure Source Data [file 44318_2024_329_MOESM17_ESM.zip › SD Appendix/FigS1F/S1F/Late2C_Mll2 KD_MLL2.jpg]

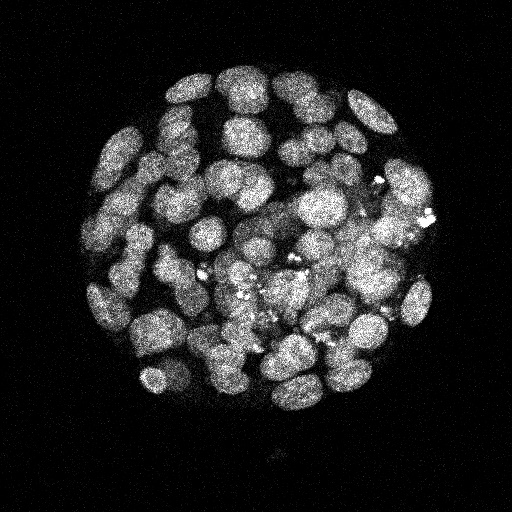

Supplement: Supplementary file 17 — Appendix Figure Source Data [file 44318_2024_329_MOESM17_ESM.zip › SD Appendix/FigS1G/S1G/Blastocyst_Control_DAPI.jpg]

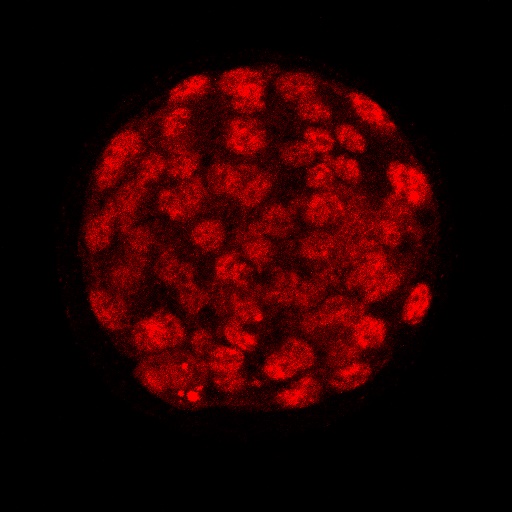

Supplement: Supplementary file 17 — Appendix Figure Source Data [file 44318_2024_329_MOESM17_ESM.zip › SD Appendix/FigS1G/S1G/Blastocyst_Control_SETD1A.jpg]

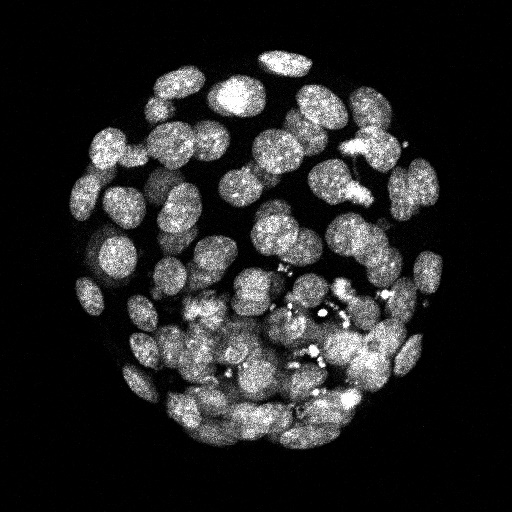

Supplement: Supplementary file 17 — Appendix Figure Source Data [file 44318_2024_329_MOESM17_ESM.zip › SD Appendix/FigS1G/S1G/Blastocyst_Setd1ab KD_DAPI.jpg]

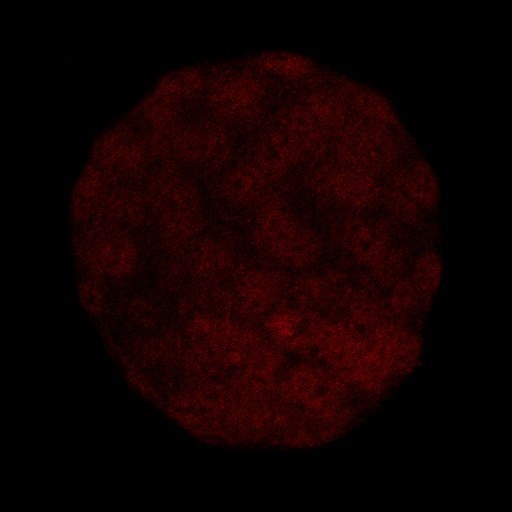

Supplement: Supplementary file 17 — Appendix Figure Source Data [file 44318_2024_329_MOESM17_ESM.zip › SD Appendix/FigS1G/S1G/Blastocyst_Setd1ab KD_SETD1A.jpg]

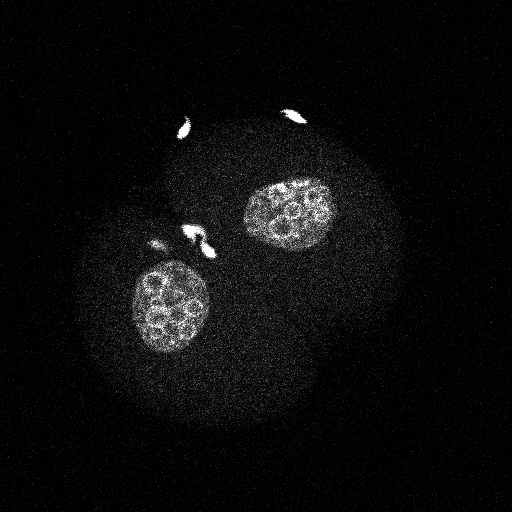

Supplement: Supplementary file 17 — Appendix Figure Source Data [file 44318_2024_329_MOESM17_ESM.zip › SD Appendix/FigS1G/S1G/Late2C_Control_DAPI.jpg]

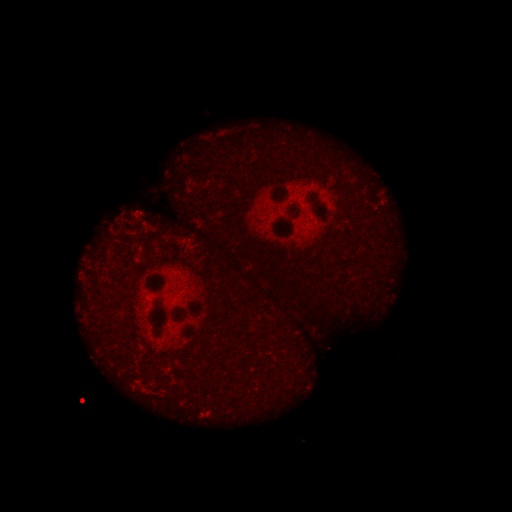

Supplement: Supplementary file 17 — Appendix Figure Source Data [file 44318_2024_329_MOESM17_ESM.zip › SD Appendix/FigS1G/S1G/Late2C_Control_SETD1A.jpg]

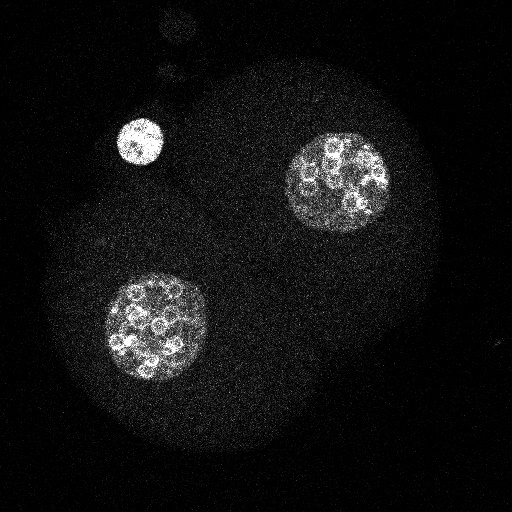

Supplement: Supplementary file 17 — Appendix Figure Source Data [file 44318_2024_329_MOESM17_ESM.zip › SD Appendix/FigS1G/S1G/Late2C_Setd1ab KD_DAPI.jpg]

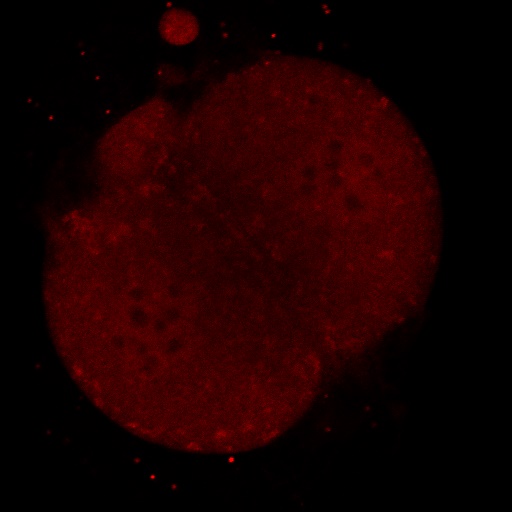

Supplement: Supplementary file 17 — Appendix Figure Source Data [file 44318_2024_329_MOESM17_ESM.zip › SD Appendix/FigS1G/S1G/Late2C_Setd1ab KD_SETD1A.jpg]

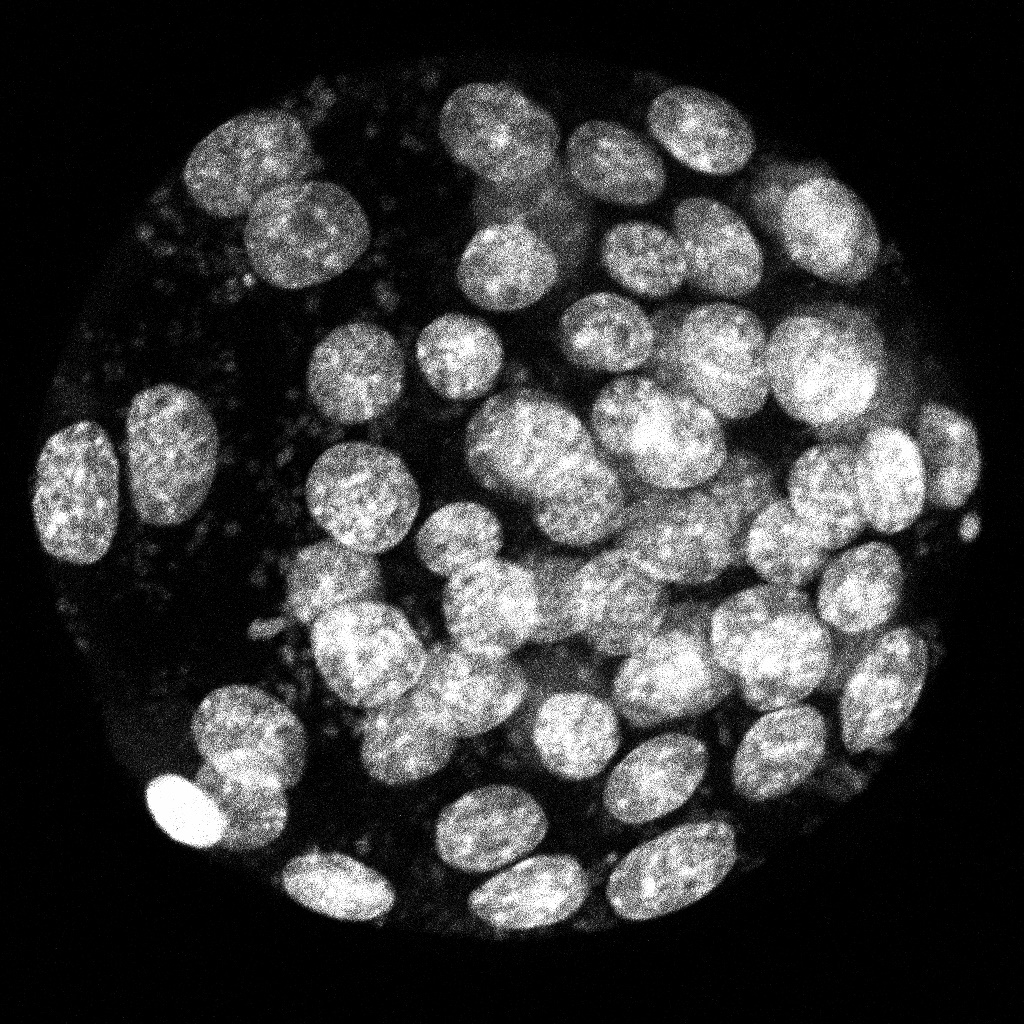

Supplement: Supplementary file 17 — Appendix Figure Source Data [file 44318_2024_329_MOESM17_ESM.zip › SD Appendix/FigS1H/S1H/Blastocyst_Control_DAPI.jpg]

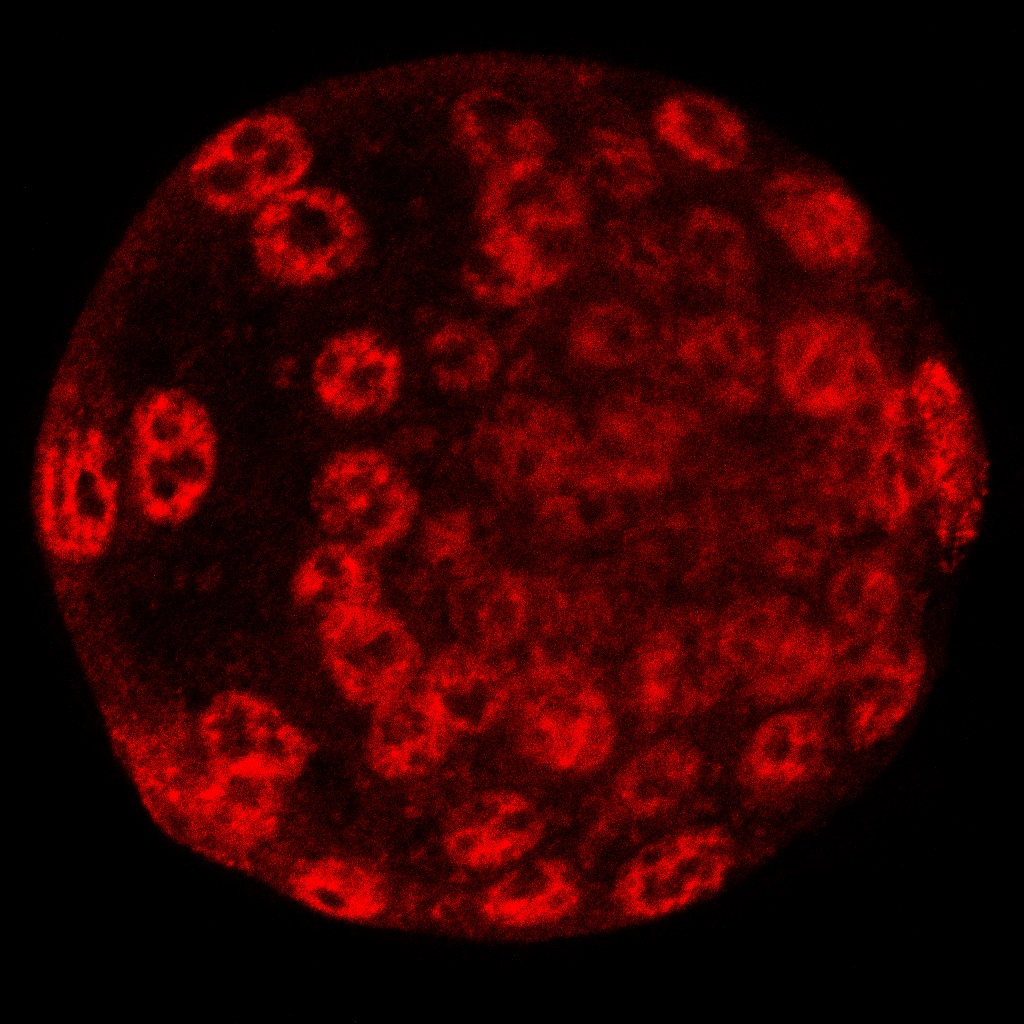

Supplement: Supplementary file 17 — Appendix Figure Source Data [file 44318_2024_329_MOESM17_ESM.zip › SD Appendix/FigS1H/S1H/Blastocyst_Control_SETD1B.jpg]

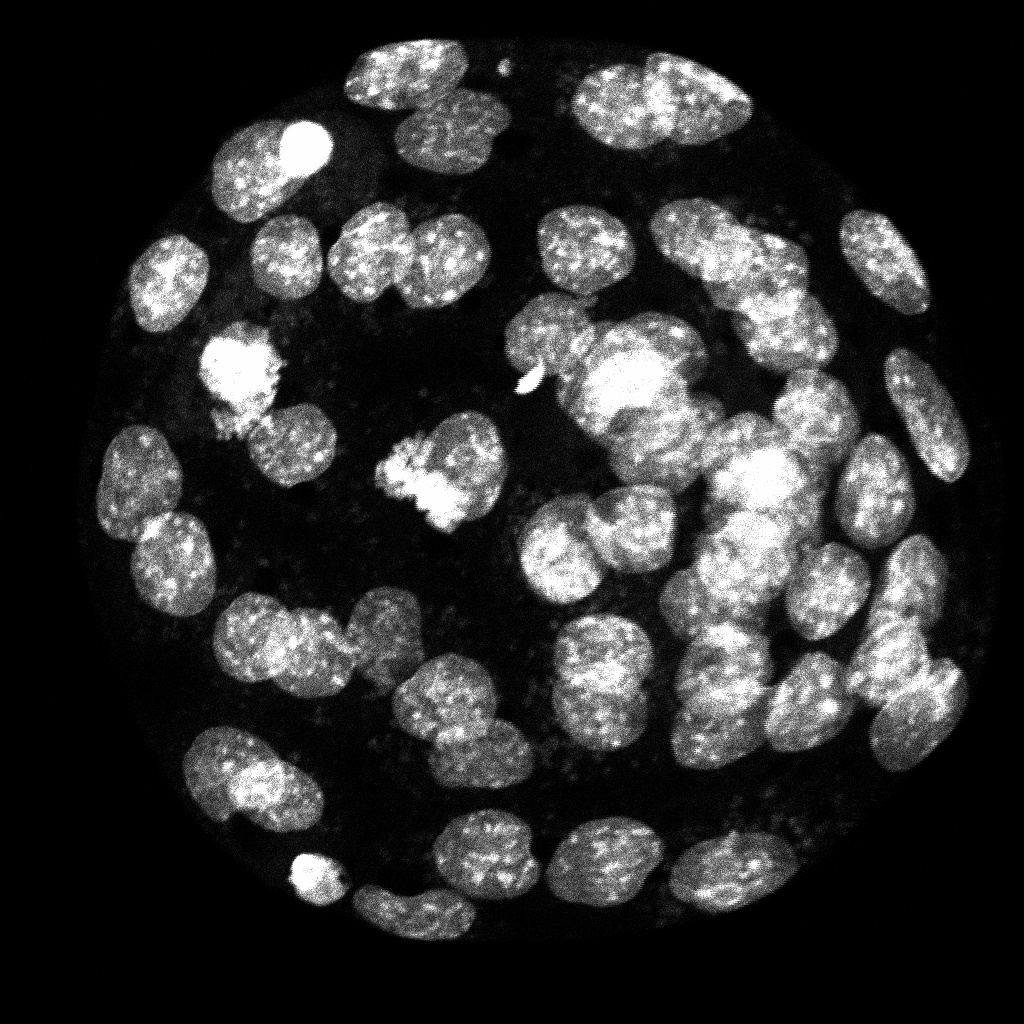

Supplement: Supplementary file 17 — Appendix Figure Source Data [file 44318_2024_329_MOESM17_ESM.zip › SD Appendix/FigS1H/S1H/Blastocyst_Setd1ab KD_DAPI.jpg]

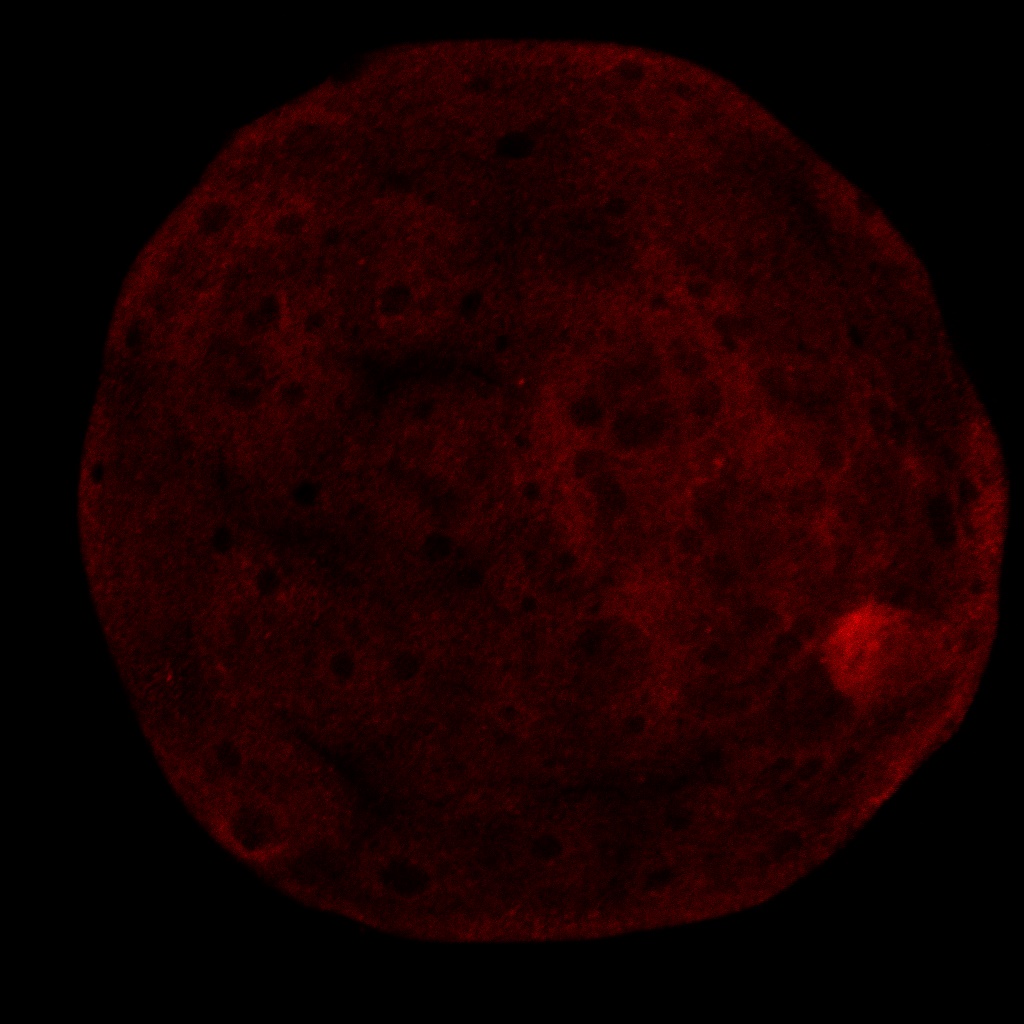

Supplement: Supplementary file 17 — Appendix Figure Source Data [file 44318_2024_329_MOESM17_ESM.zip › SD Appendix/FigS1H/S1H/Blastocyst_Setd1ab KD_SETD1B.jpg]

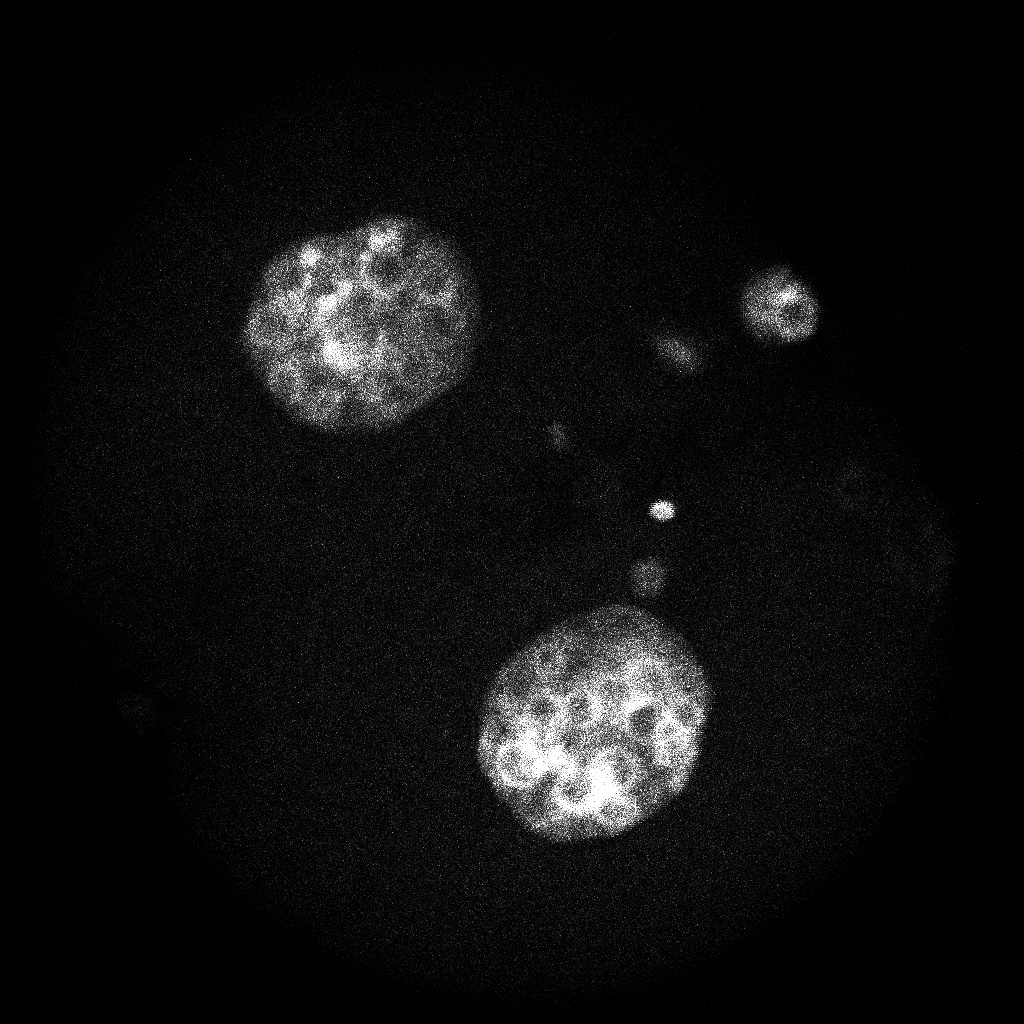

Supplement: Supplementary file 17 — Appendix Figure Source Data [file 44318_2024_329_MOESM17_ESM.zip › SD Appendix/FigS1H/S1H/Late2C_Control_DAPI.jpg]

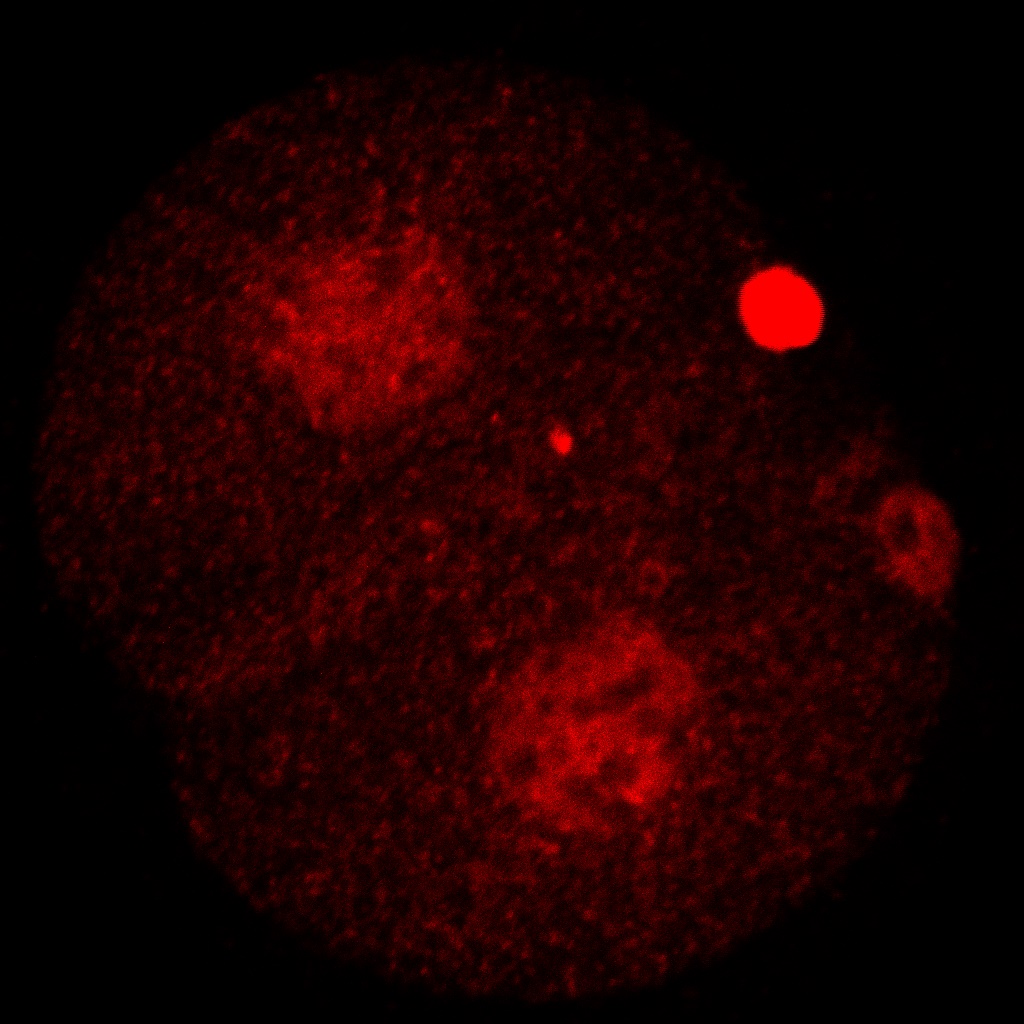

Supplement: Supplementary file 17 — Appendix Figure Source Data [file 44318_2024_329_MOESM17_ESM.zip › SD Appendix/FigS1H/S1H/Late2C_Control_SETD1B.jpg]

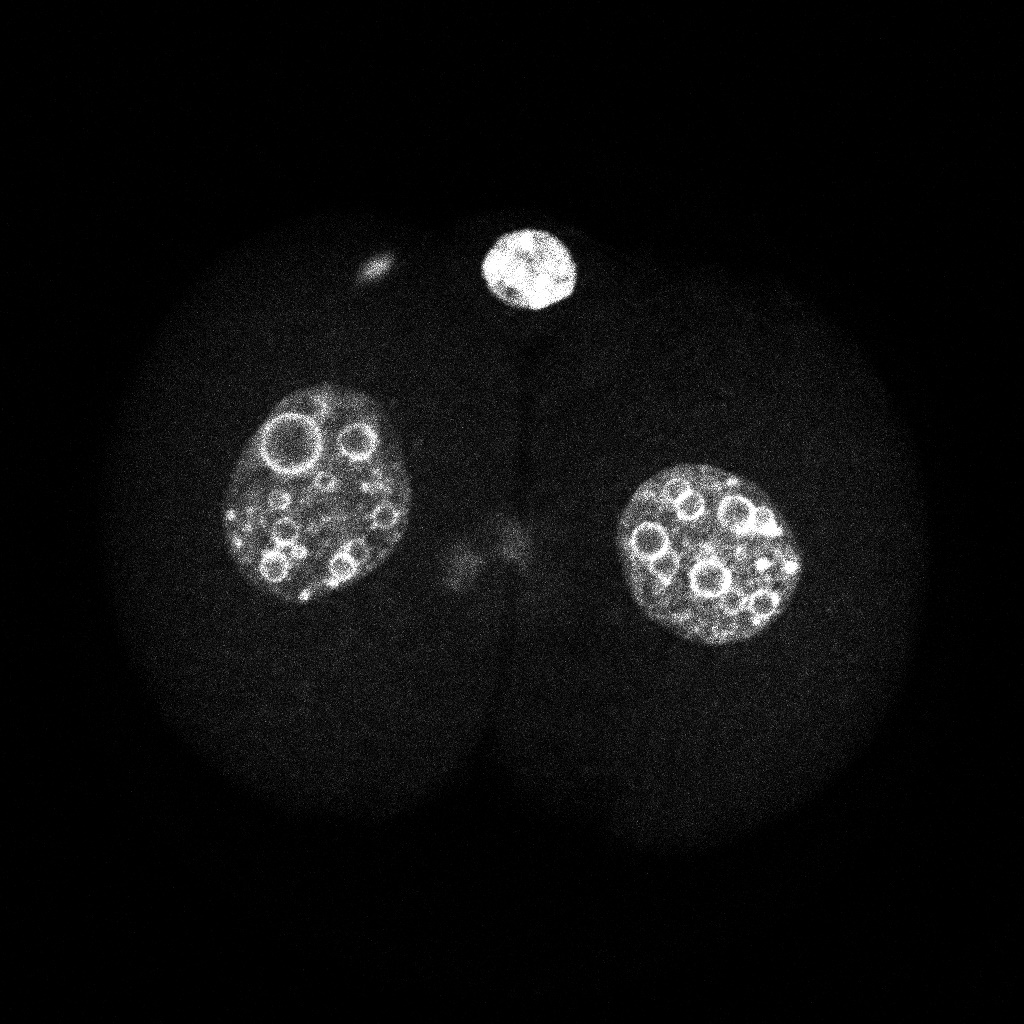

Supplement: Supplementary file 17 — Appendix Figure Source Data [file 44318_2024_329_MOESM17_ESM.zip › SD Appendix/FigS1H/S1H/Late2C_Setd1ab KD_DAPI.jpg]

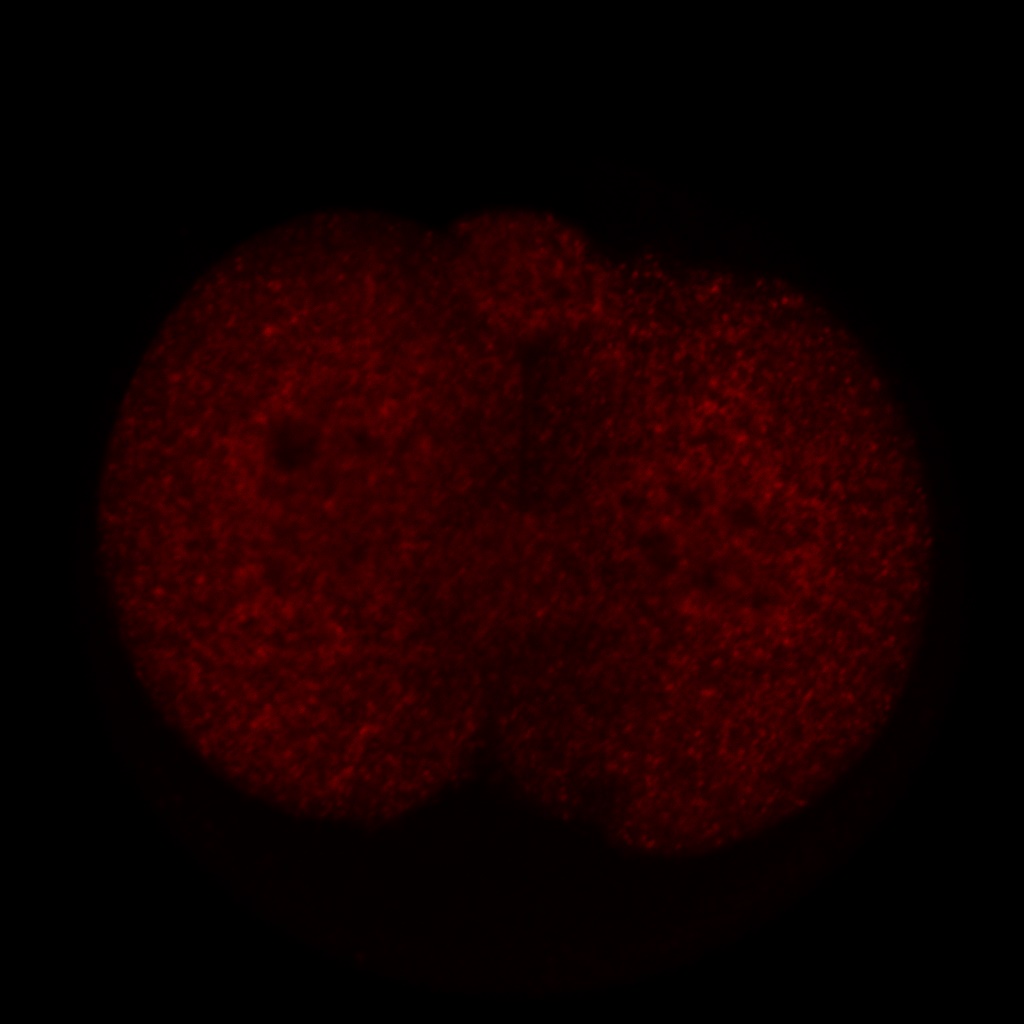

Supplement: Supplementary file 17 — Appendix Figure Source Data [file 44318_2024_329_MOESM17_ESM.zip › SD Appendix/FigS1H/S1H/Late2C_Setd1ab KD_SETD1B.jpg]

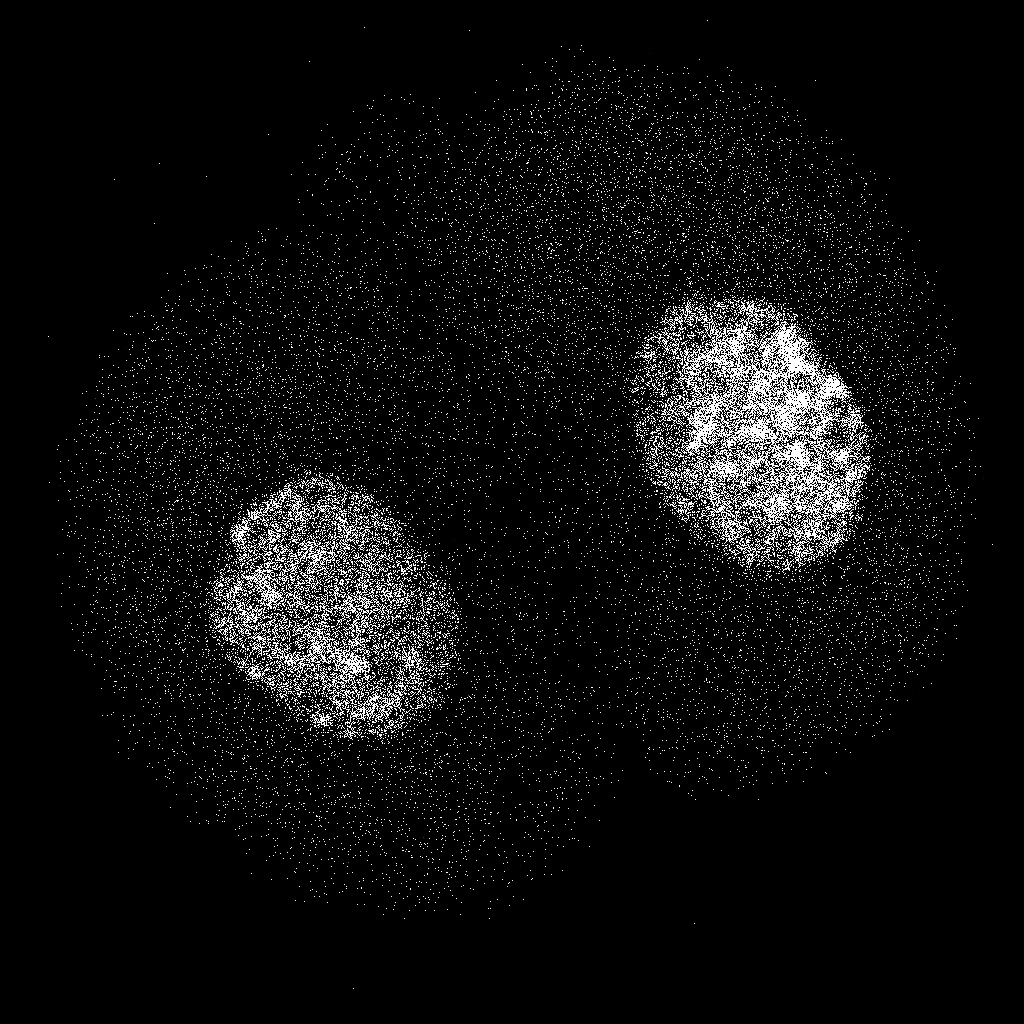

Supplement: Supplementary file 17 — Appendix Figure Source Data [file 44318_2024_329_MOESM17_ESM.zip › SD Appendix/FigS2A/Early2C_Control_DAPI.jpg]

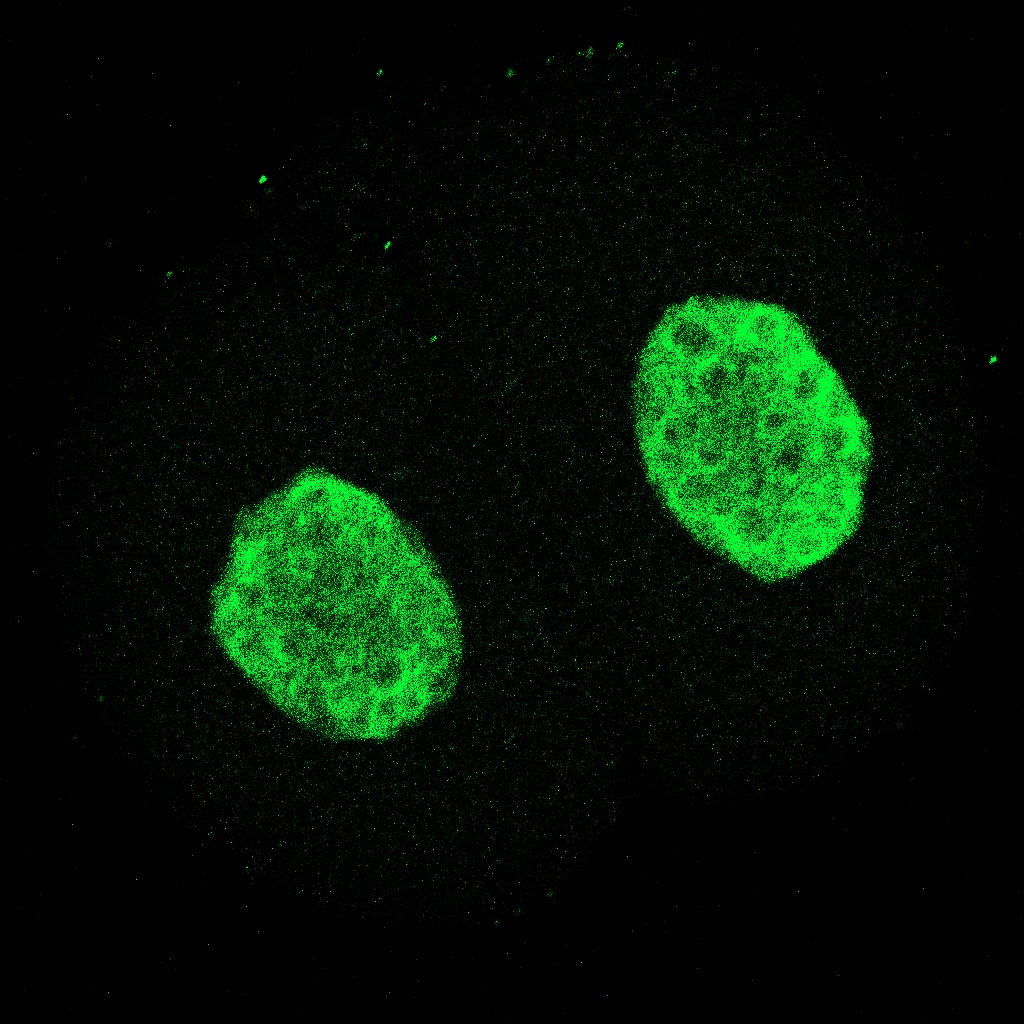

Supplement: Supplementary file 17 — Appendix Figure Source Data [file 44318_2024_329_MOESM17_ESM.zip › SD Appendix/FigS2A/Early2C_Control_H3K4me3.jpg]

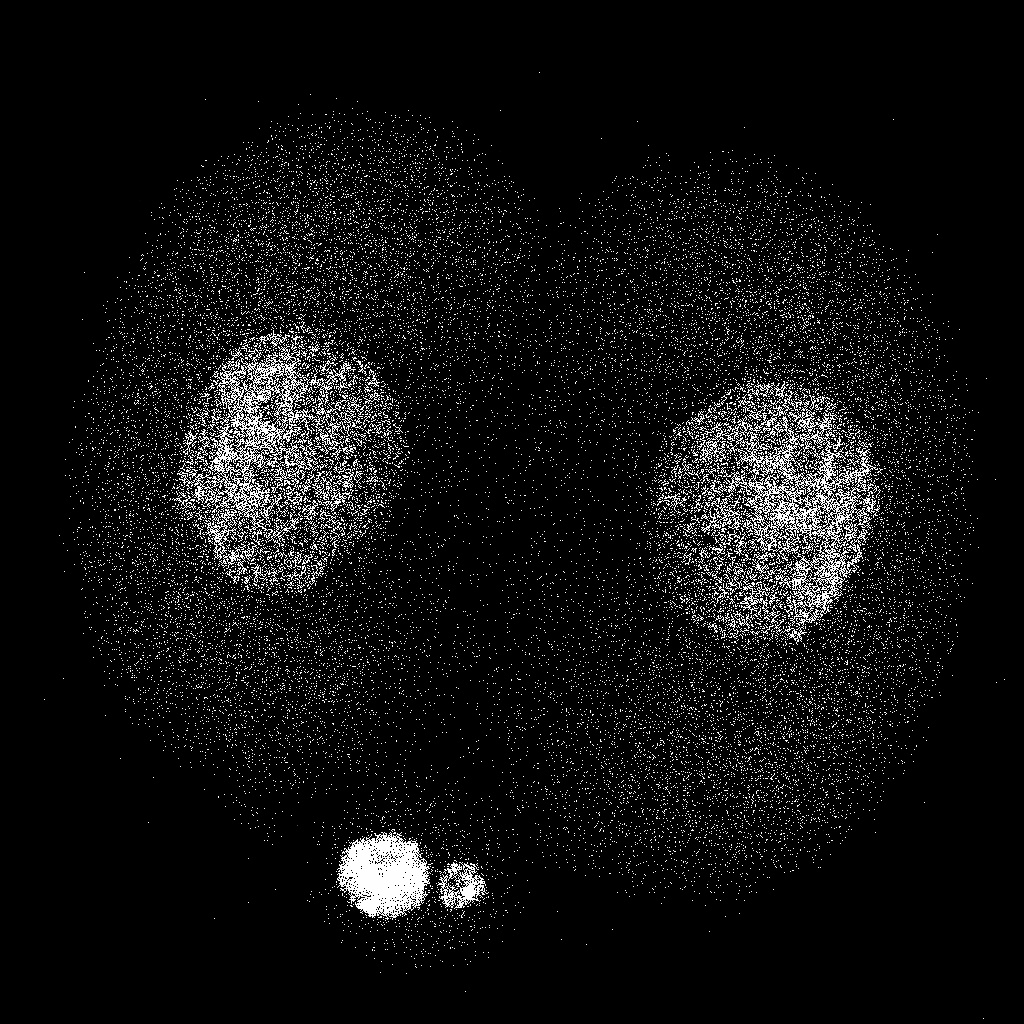

Supplement: Supplementary file 17 — Appendix Figure Source Data [file 44318_2024_329_MOESM17_ESM.zip › SD Appendix/FigS2A/Early2C_Mll2 KD_DAPI.jpg]

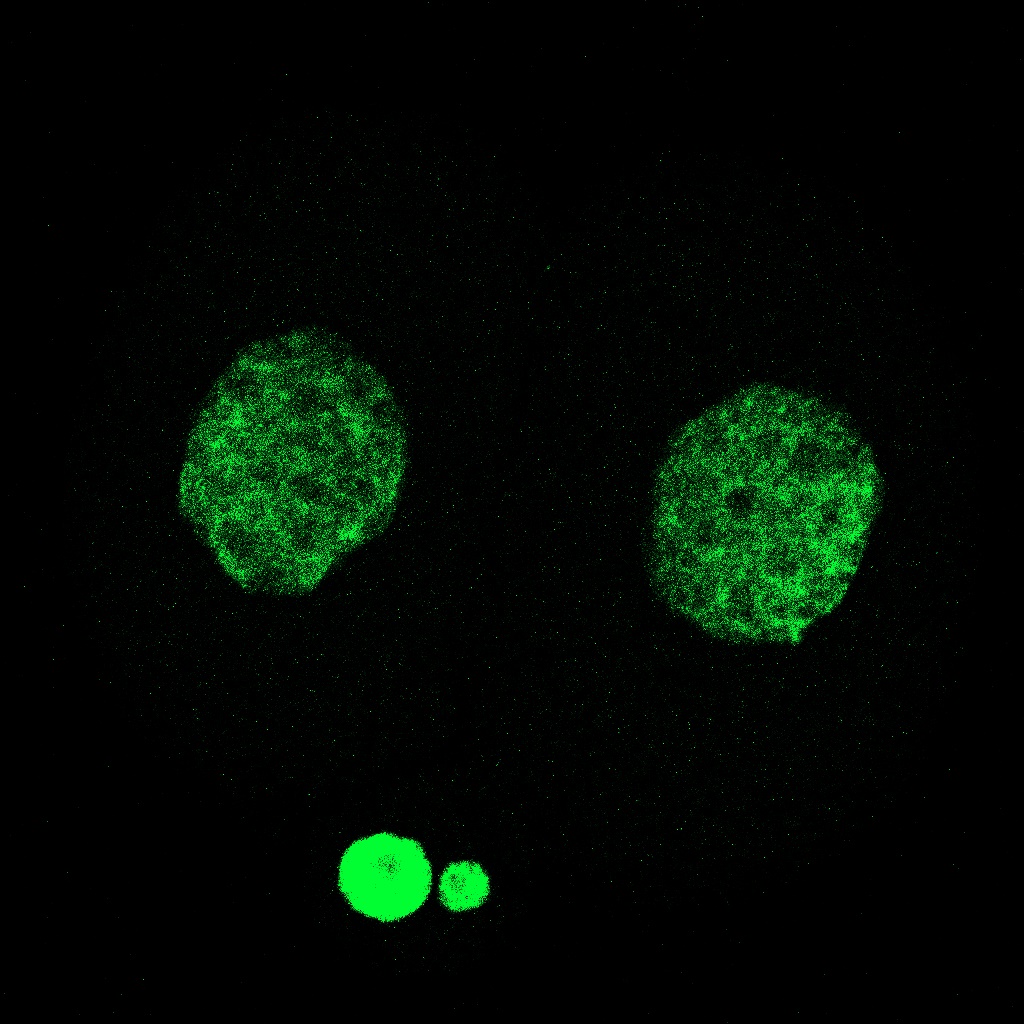

Supplement: Supplementary file 17 — Appendix Figure Source Data [file 44318_2024_329_MOESM17_ESM.zip › SD Appendix/FigS2A/Early2C_Mll2 KD_H3K4me3.jpg]

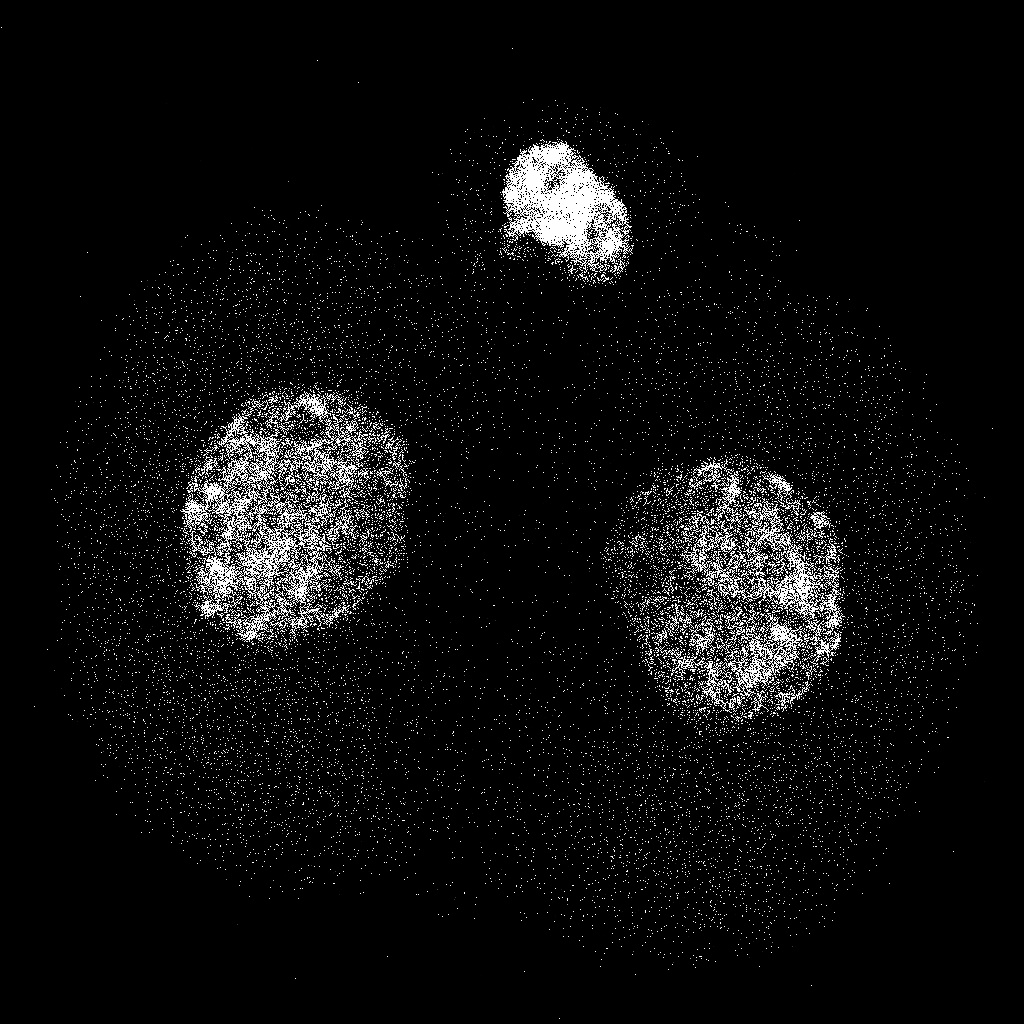

Supplement: Supplementary file 17 — Appendix Figure Source Data [file 44318_2024_329_MOESM17_ESM.zip › SD Appendix/FigS2A/Early2C_Setd1ab KD_DAPI.jpg]

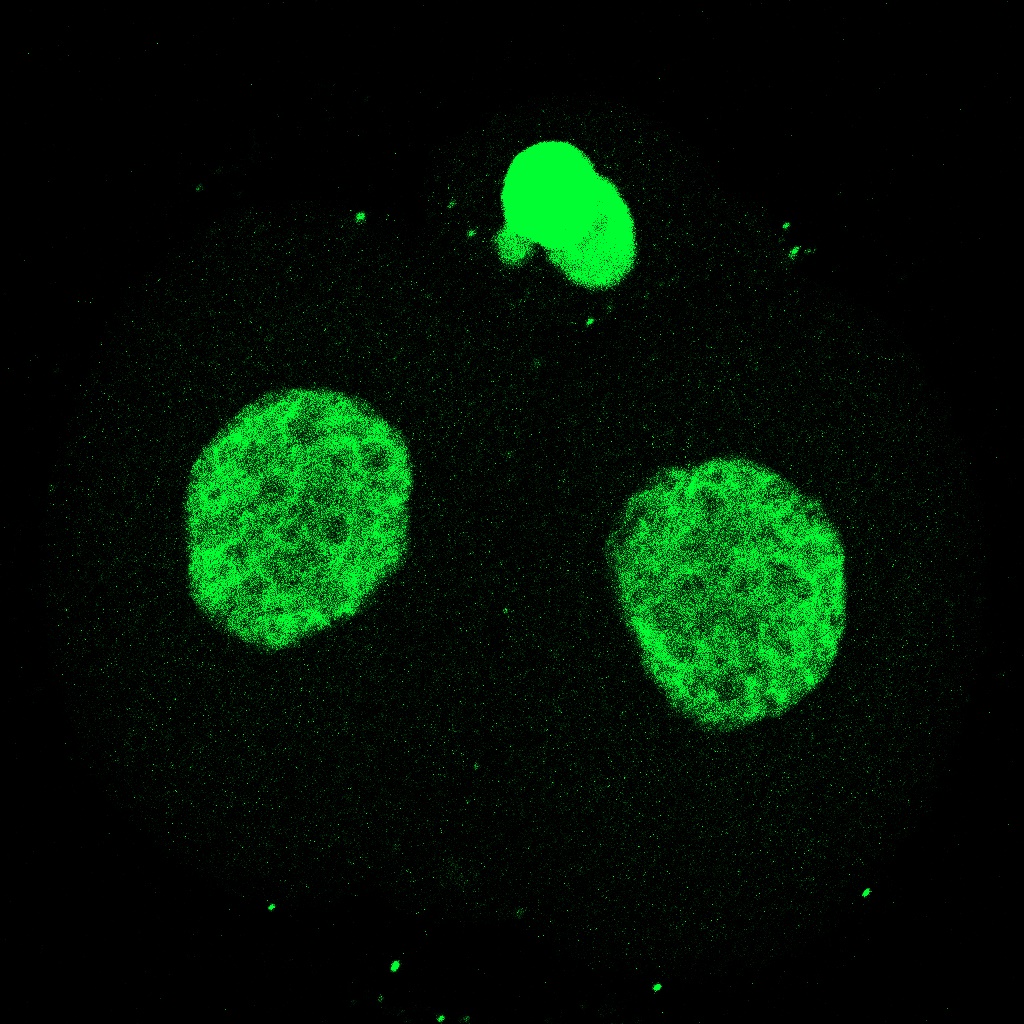

Supplement: Supplementary file 17 — Appendix Figure Source Data [file 44318_2024_329_MOESM17_ESM.zip › SD Appendix/FigS2A/Early2C_Setd1ab KD_H3K4me3.jpg]

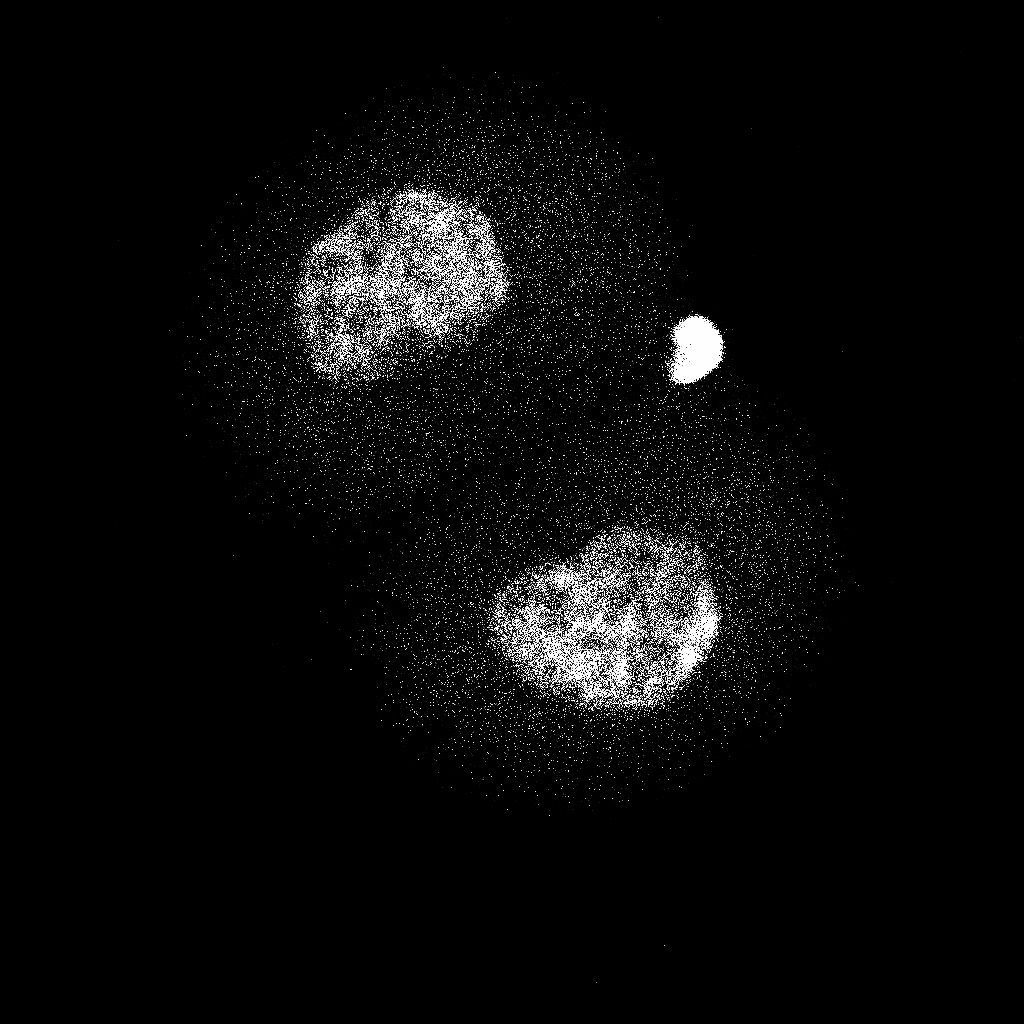

Supplement: Supplementary file 17 — Appendix Figure Source Data [file 44318_2024_329_MOESM17_ESM.zip › SD Appendix/FigS4A/S4A/Late2C_0 μM_DAPI.jpg]

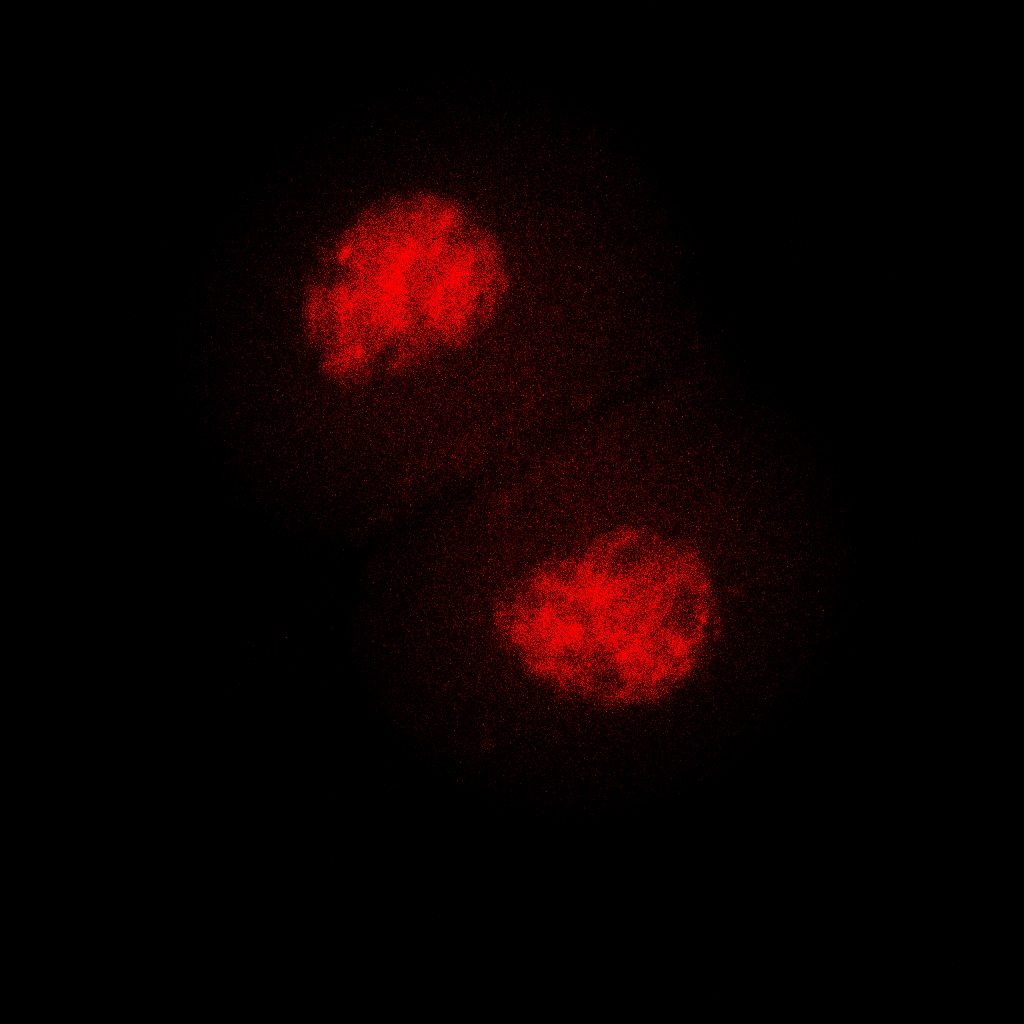

Supplement: Supplementary file 17 — Appendix Figure Source Data [file 44318_2024_329_MOESM17_ESM.zip › SD Appendix/FigS4A/S4A/Late2C_0 μM_EU.jpg]

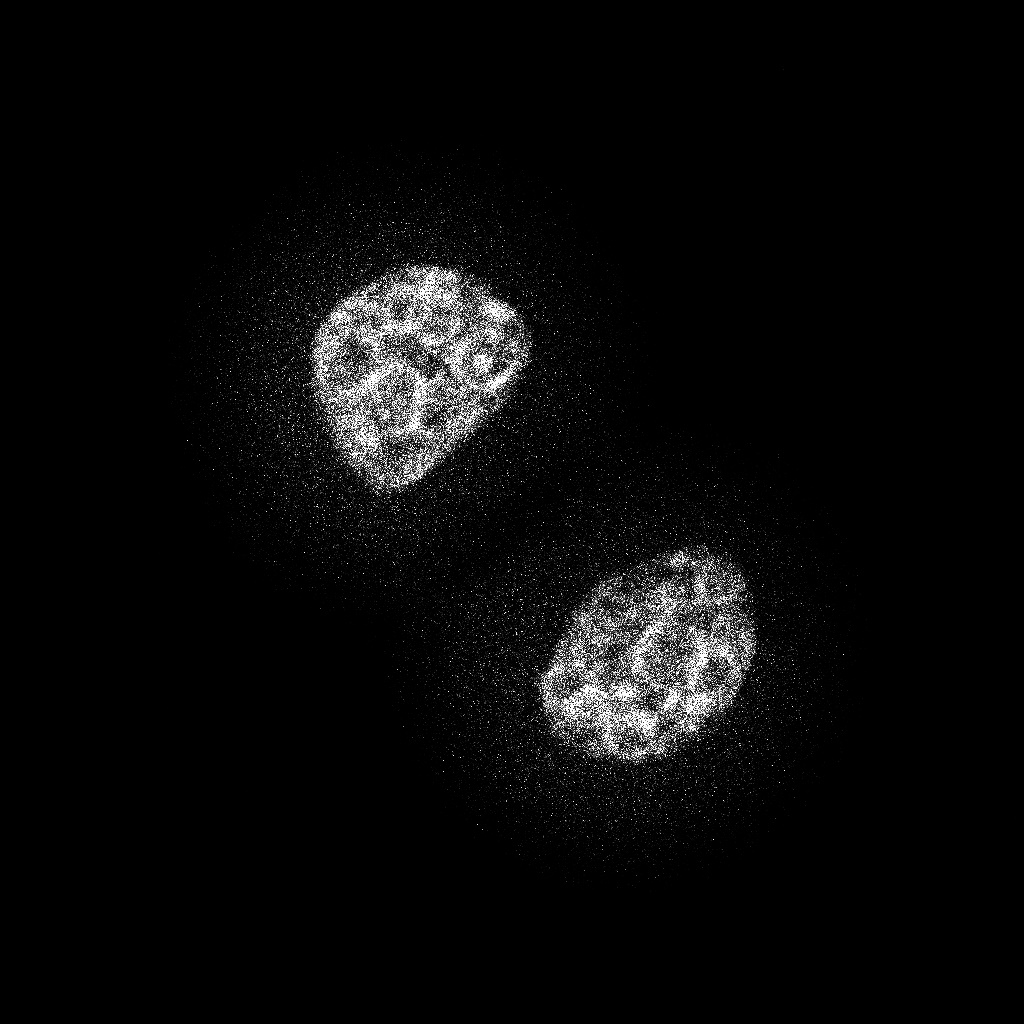

Supplement: Supplementary file 17 — Appendix Figure Source Data [file 44318_2024_329_MOESM17_ESM.zip › SD Appendix/FigS4A/S4A/Late2C_0.01 μM_DAPI.jpg]

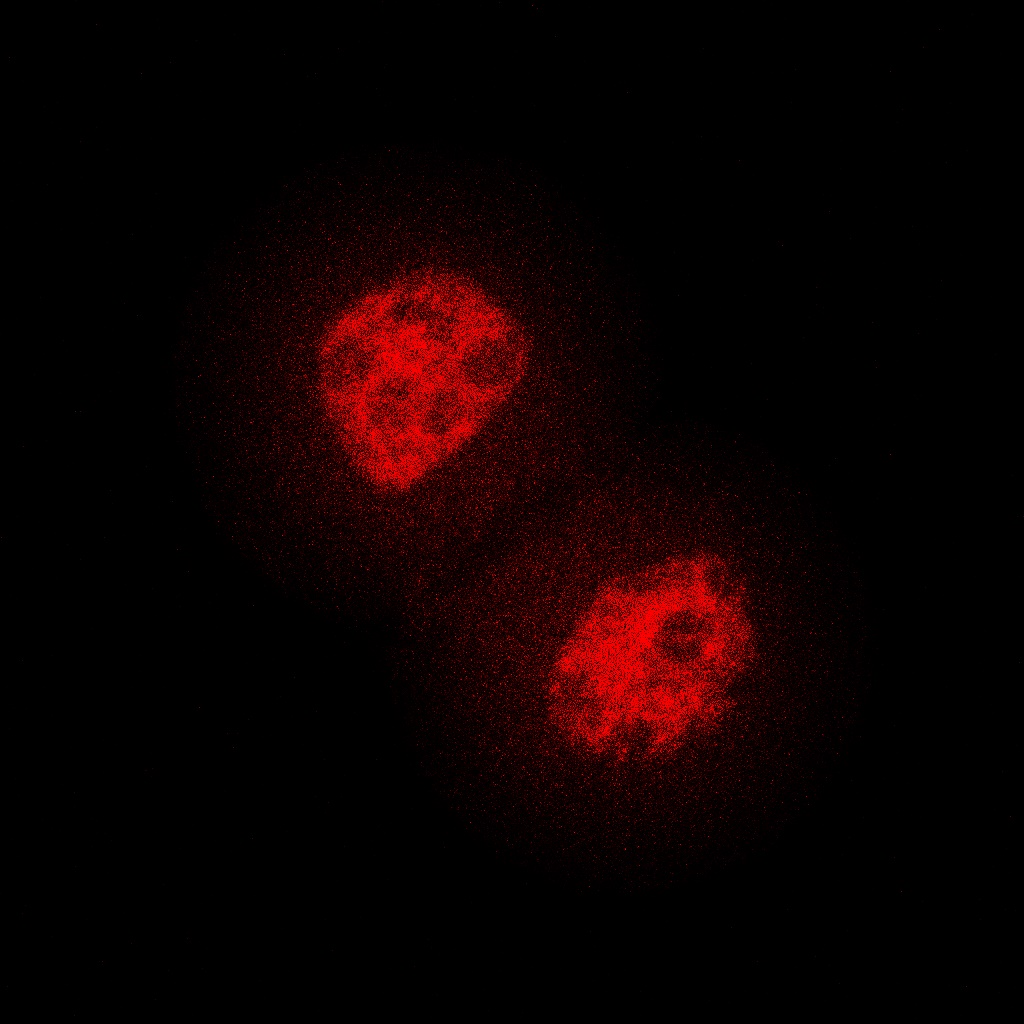

Supplement: Supplementary file 17 — Appendix Figure Source Data [file 44318_2024_329_MOESM17_ESM.zip › SD Appendix/FigS4A/S4A/Late2C_0.01 μM_EU.jpg]

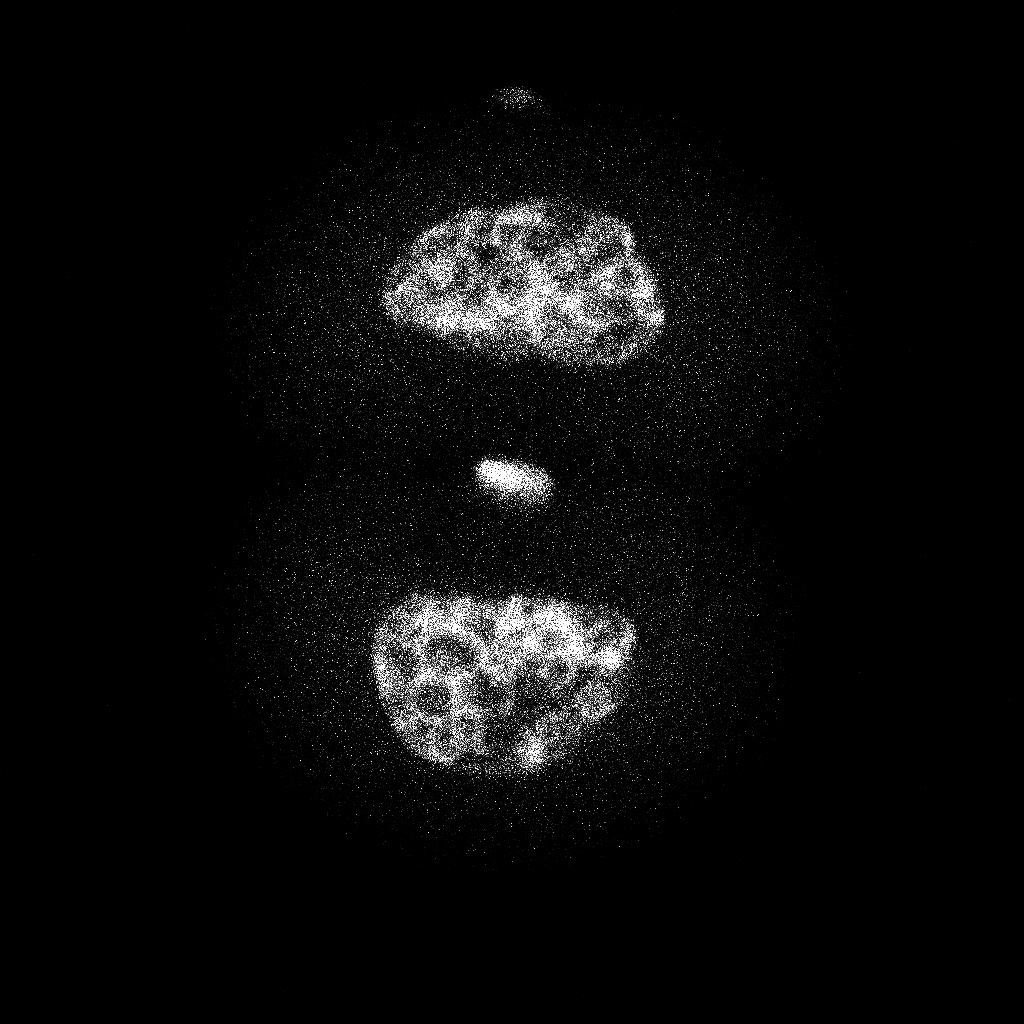

Supplement: Supplementary file 17 — Appendix Figure Source Data [file 44318_2024_329_MOESM17_ESM.zip › SD Appendix/FigS4A/S4A/Late2C_0.1 μM_DAPI.jpg]

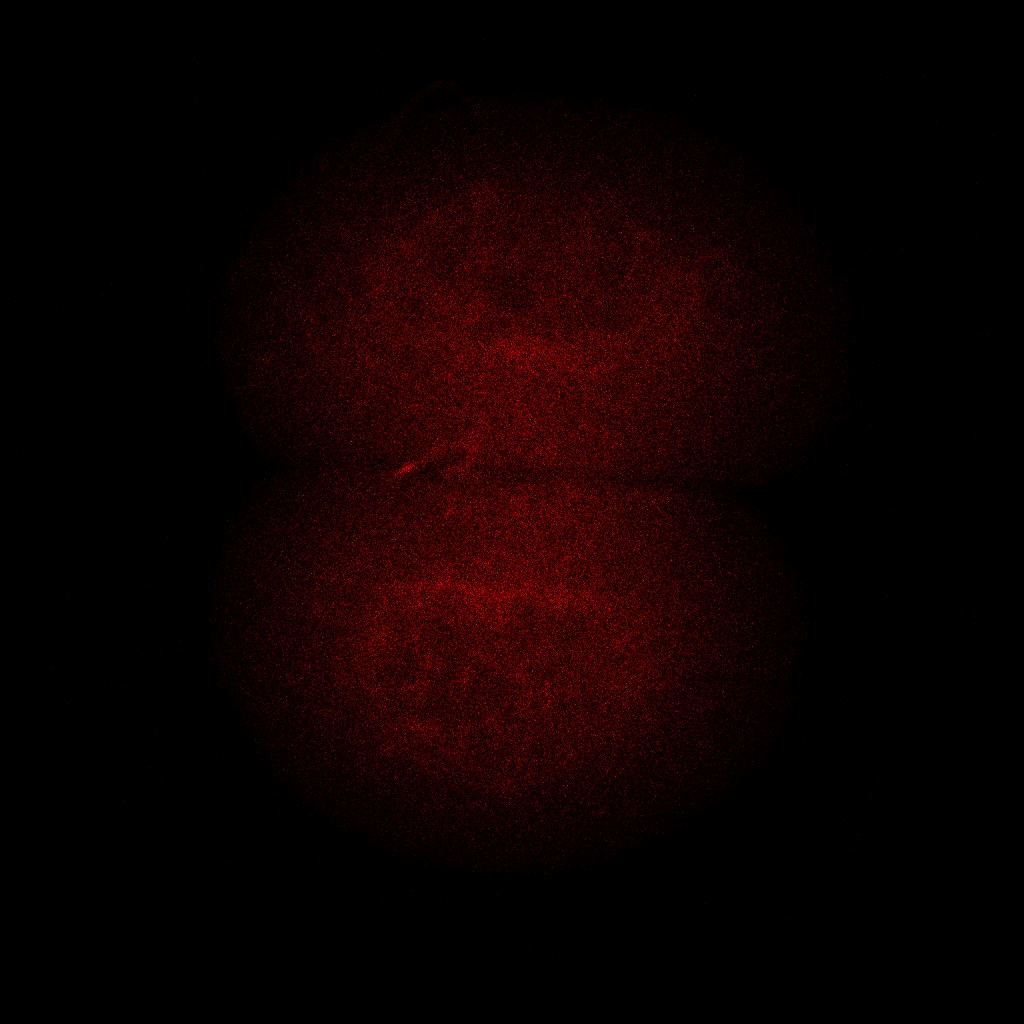

Supplement: Supplementary file 17 — Appendix Figure Source Data [file 44318_2024_329_MOESM17_ESM.zip › SD Appendix/FigS4A/S4A/Late2C_0.1 μM_EU.jpg]

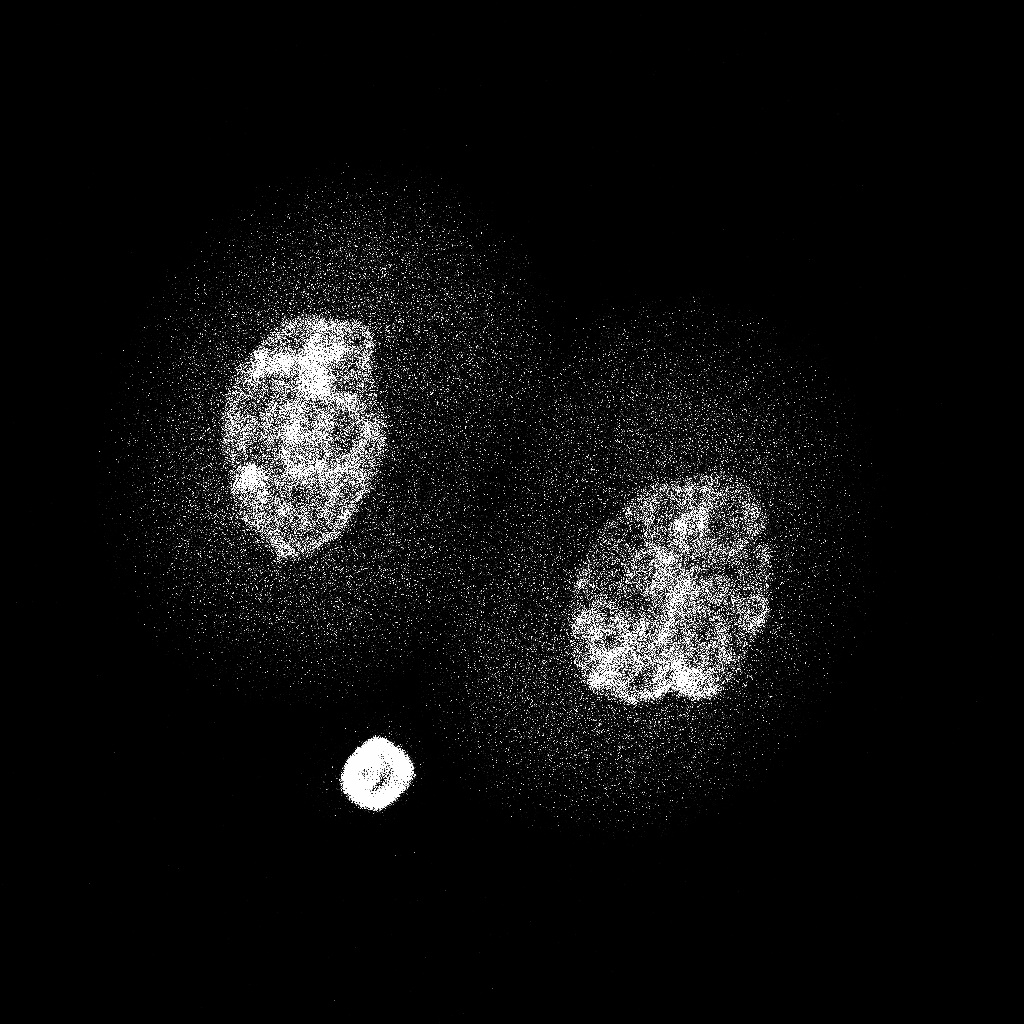

Supplement: Supplementary file 17 — Appendix Figure Source Data [file 44318_2024_329_MOESM17_ESM.zip › SD Appendix/FigS4A/S4A/Late2C_1 μM_DAPI.jpg]

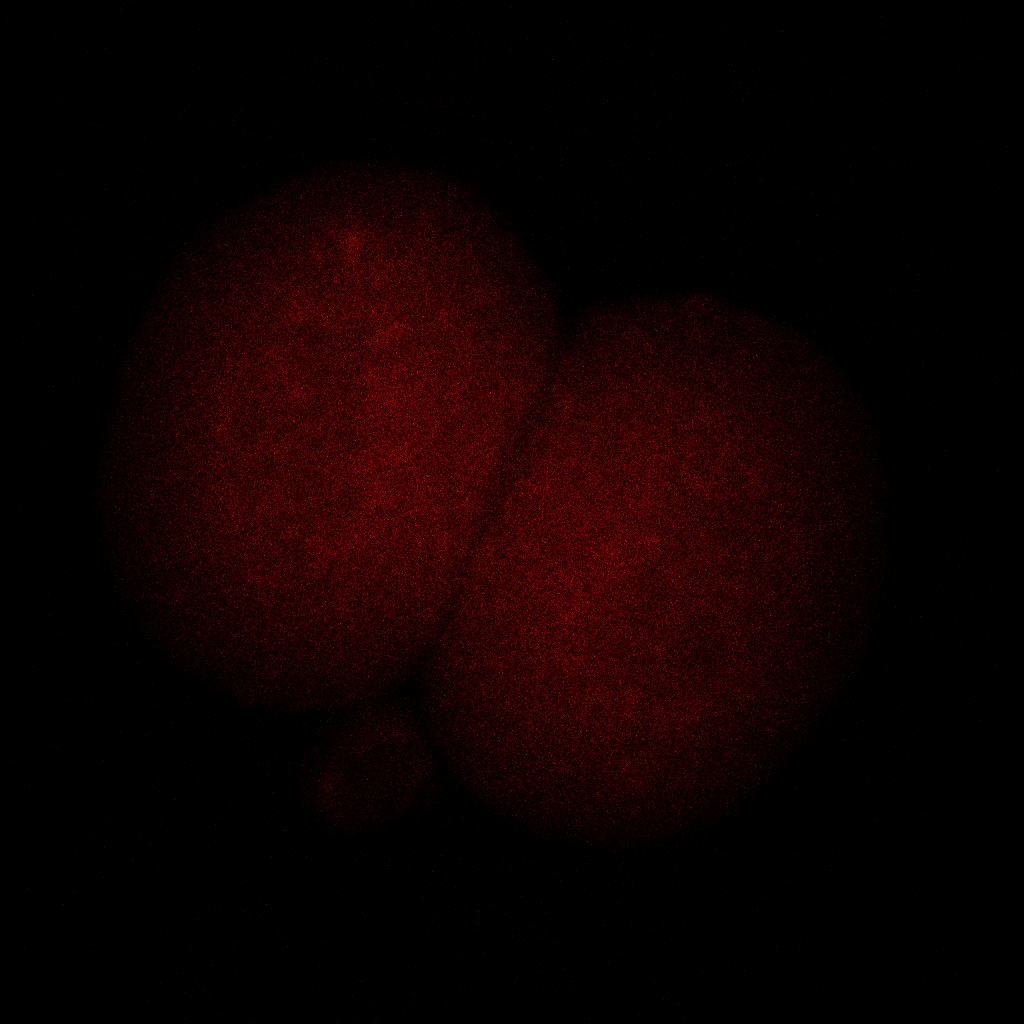

Supplement: Supplementary file 17 — Appendix Figure Source Data [file 44318_2024_329_MOESM17_ESM.zip › SD Appendix/FigS4A/S4A/Late2C_1 μM_EU.jpg]

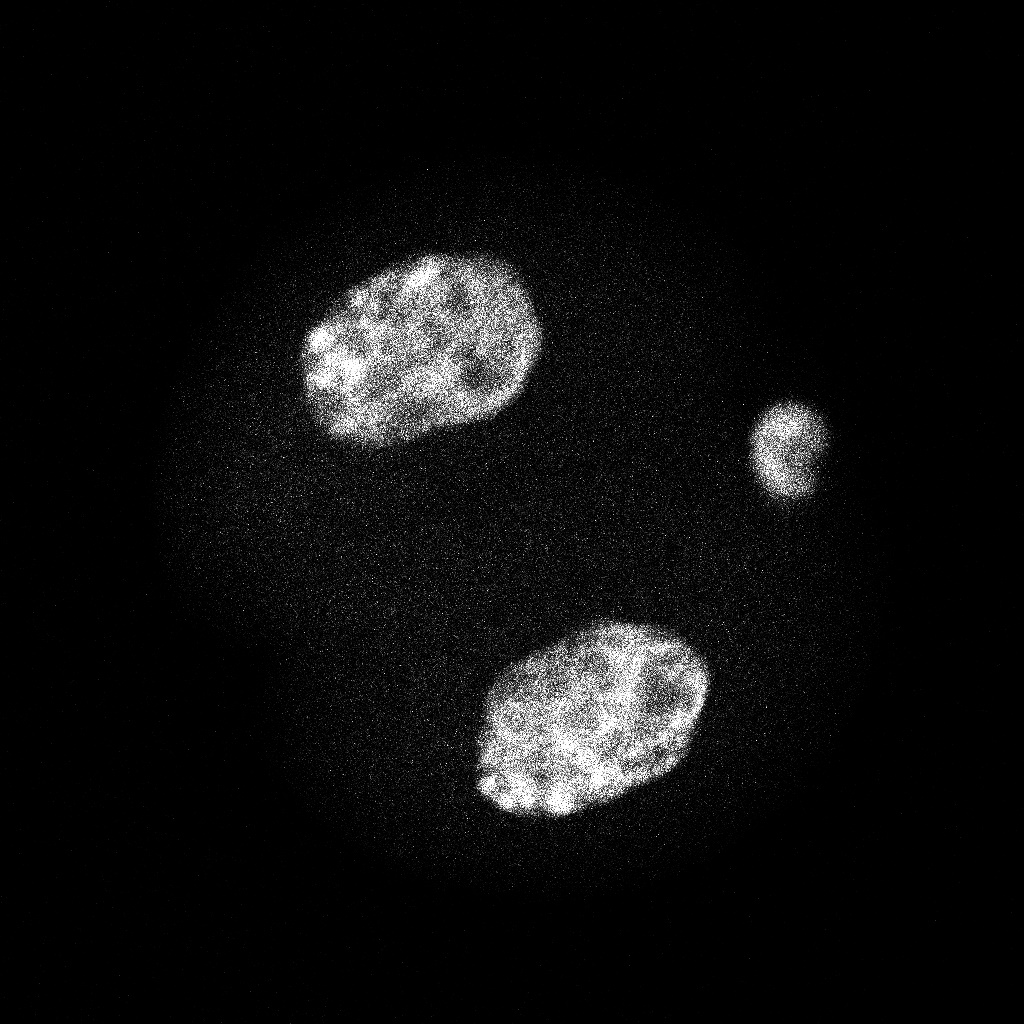

Supplement: Supplementary file 17 — Appendix Figure Source Data [file 44318_2024_329_MOESM17_ESM.zip › SD Appendix/FigS4B/S4B/Late2C_0h_DAPI.jpg]

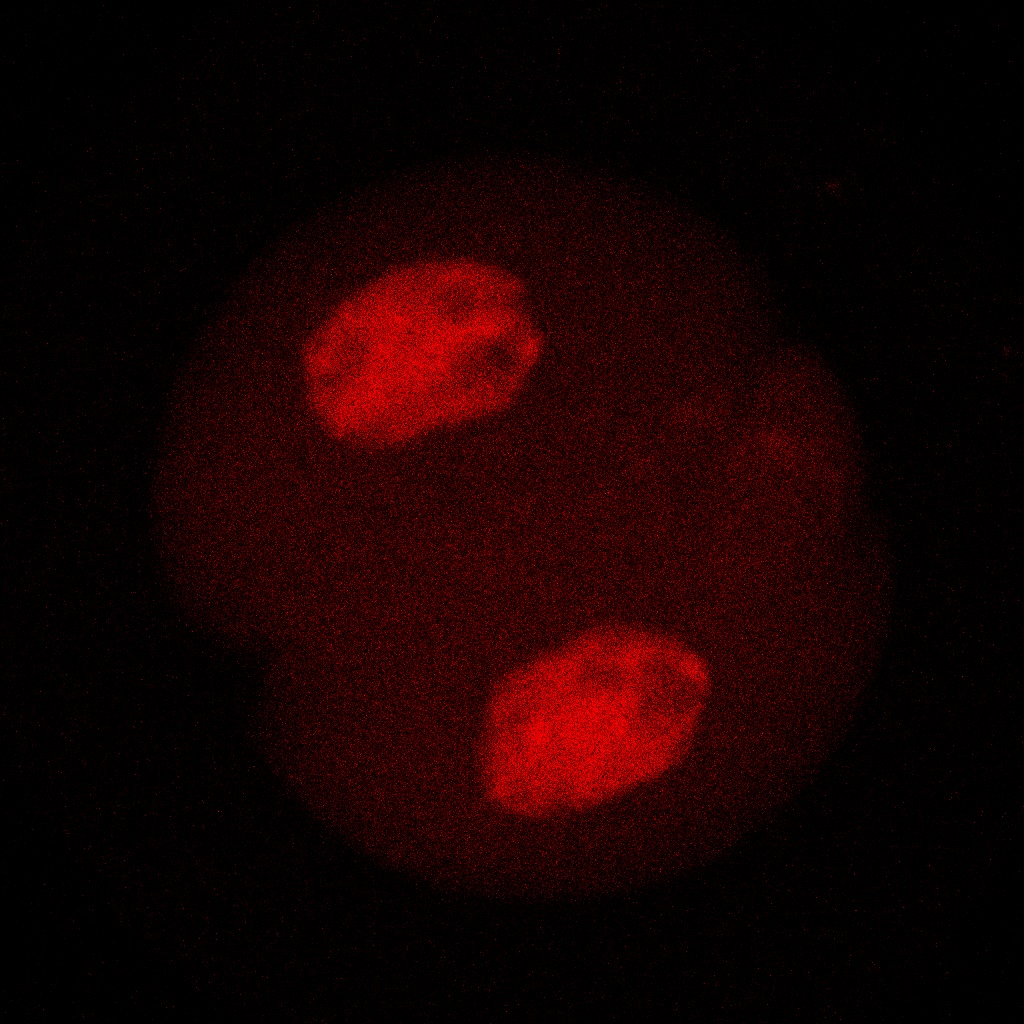

Supplement: Supplementary file 17 — Appendix Figure Source Data [file 44318_2024_329_MOESM17_ESM.zip › SD Appendix/FigS4B/S4B/Late2C_0h_EU.jpg]

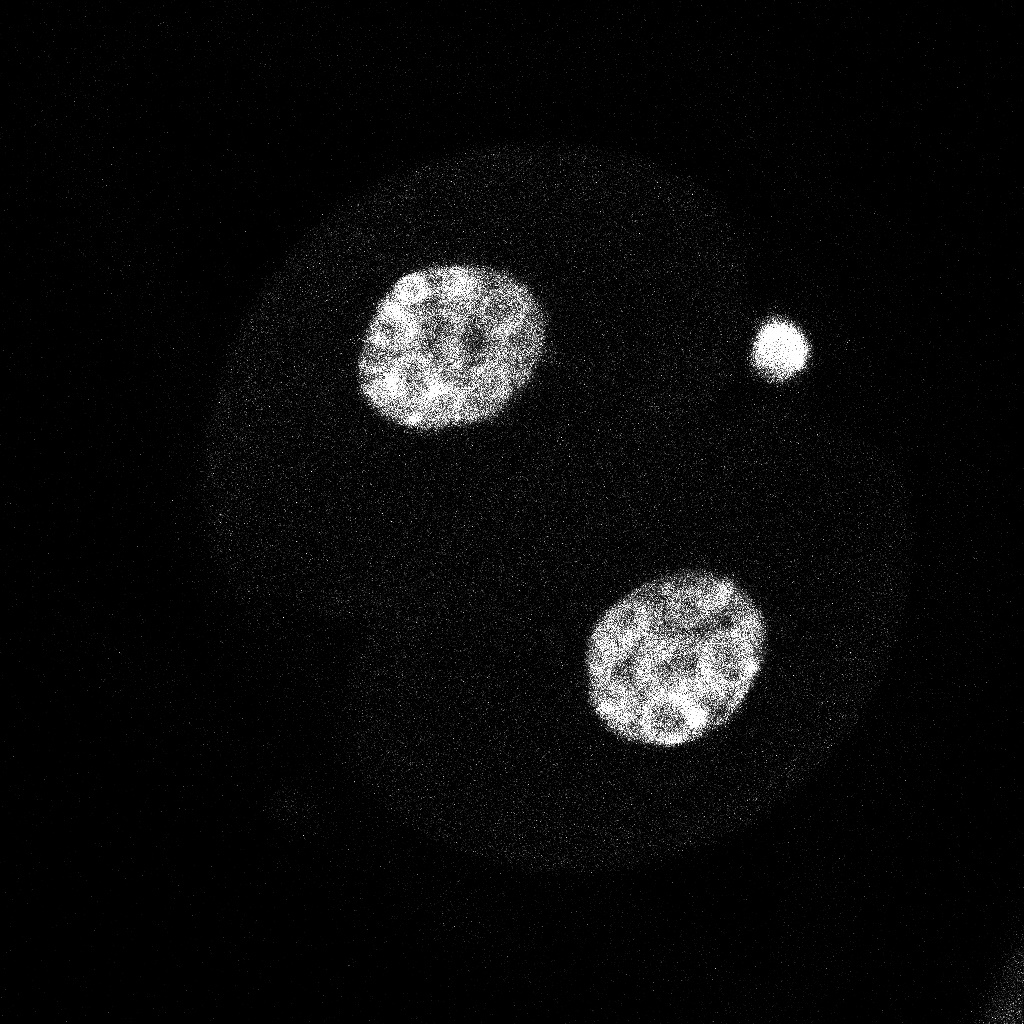

Supplement: Supplementary file 17 — Appendix Figure Source Data [file 44318_2024_329_MOESM17_ESM.zip › SD Appendix/FigS4B/S4B/Late2C_2h_DAPI.jpg]

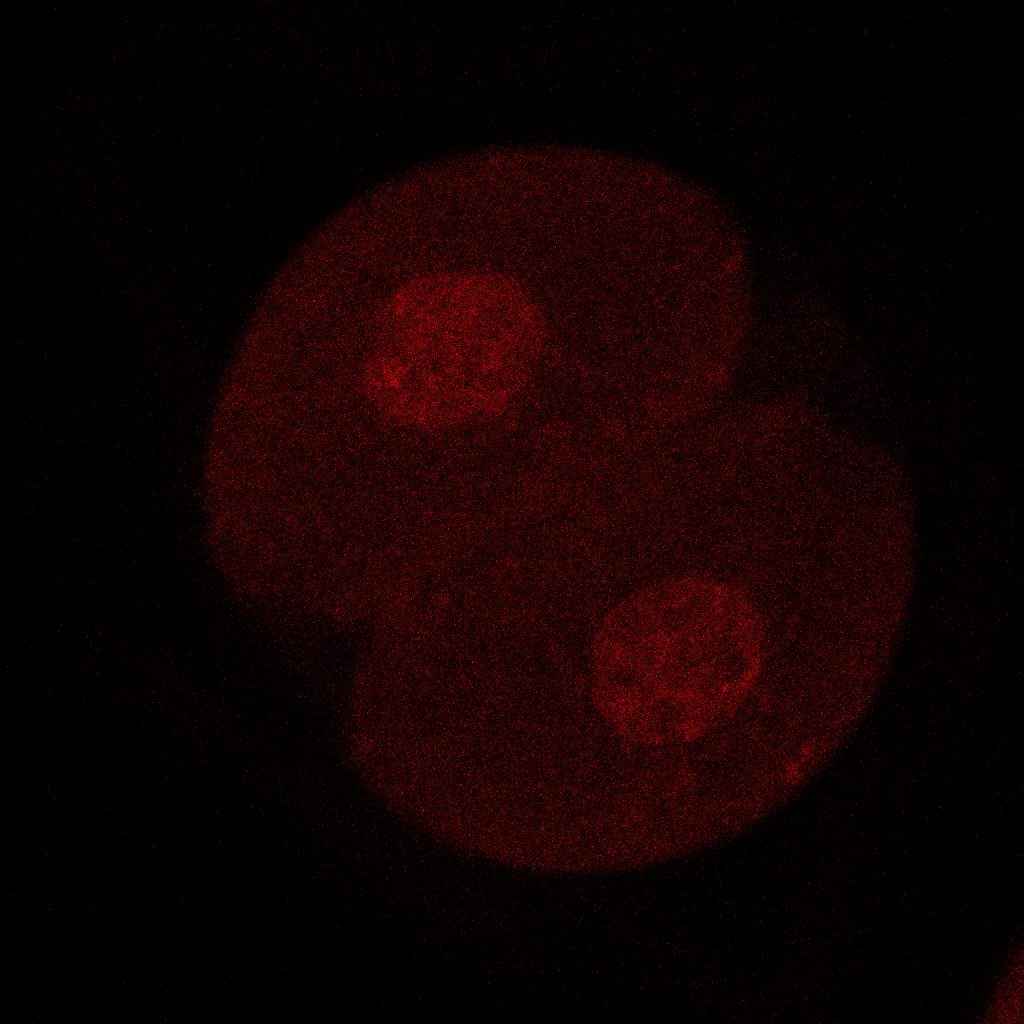

Supplement: Supplementary file 17 — Appendix Figure Source Data [file 44318_2024_329_MOESM17_ESM.zip › SD Appendix/FigS4B/S4B/Late2C_2h_EU.jpg]

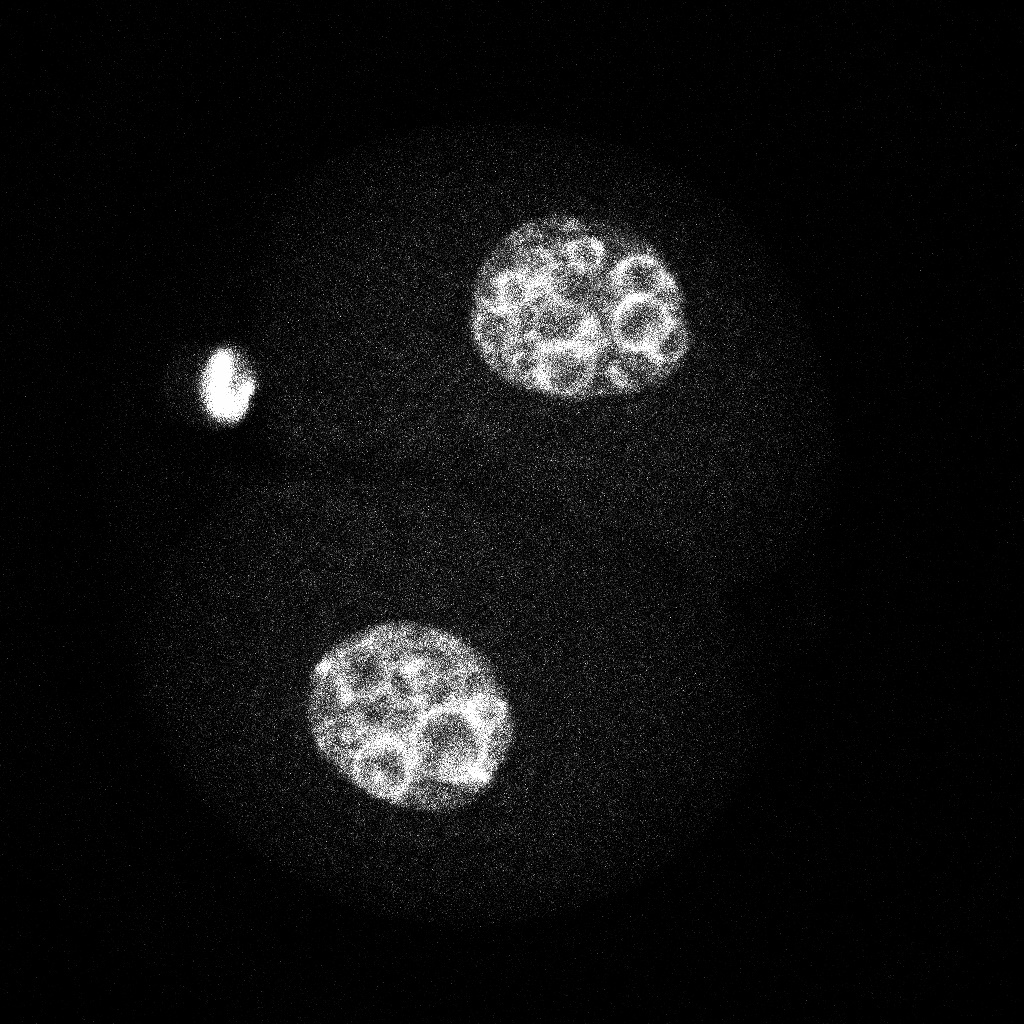

Supplement: Supplementary file 17 — Appendix Figure Source Data [file 44318_2024_329_MOESM17_ESM.zip › SD Appendix/FigS4B/S4B/Late2C_4h_DAPI.jpg]

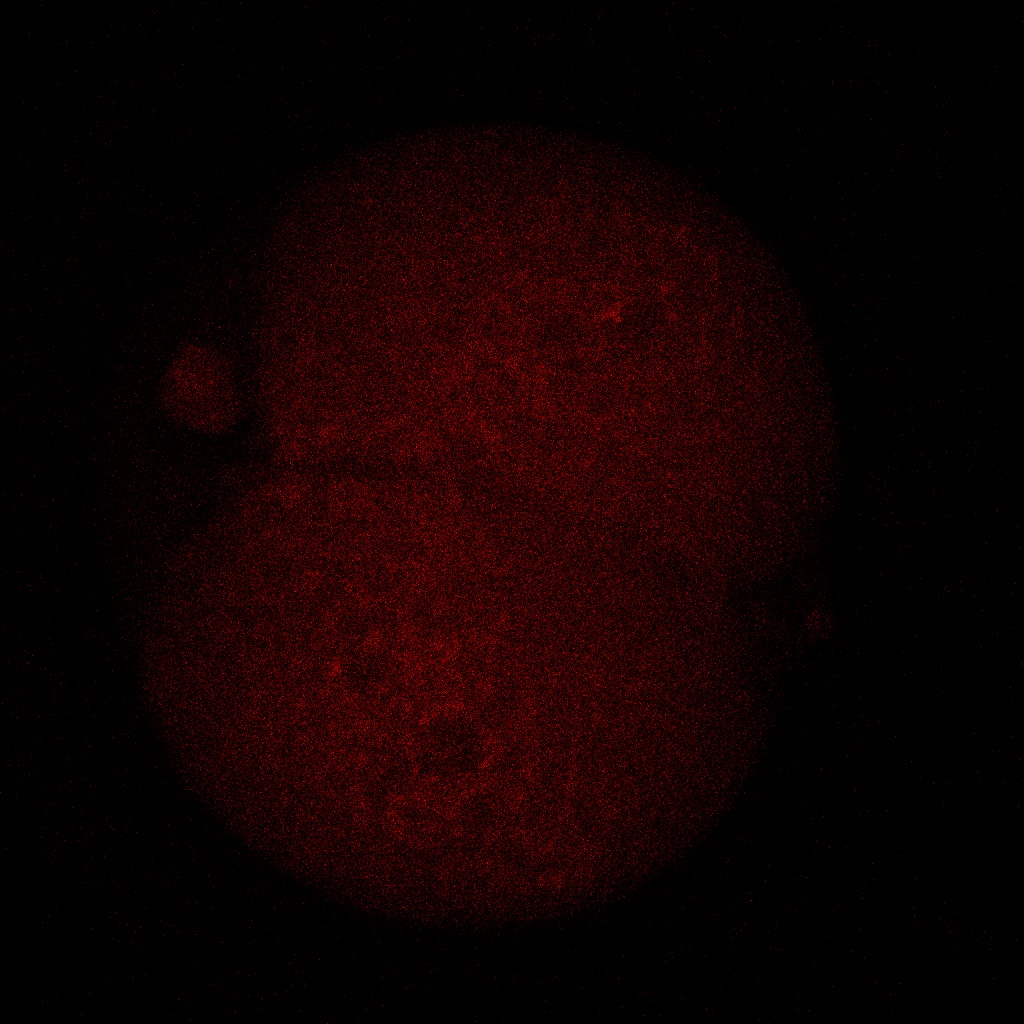

Supplement: Supplementary file 17 — Appendix Figure Source Data [file 44318_2024_329_MOESM17_ESM.zip › SD Appendix/FigS4B/S4B/Late2C_4h_EU.jpg]

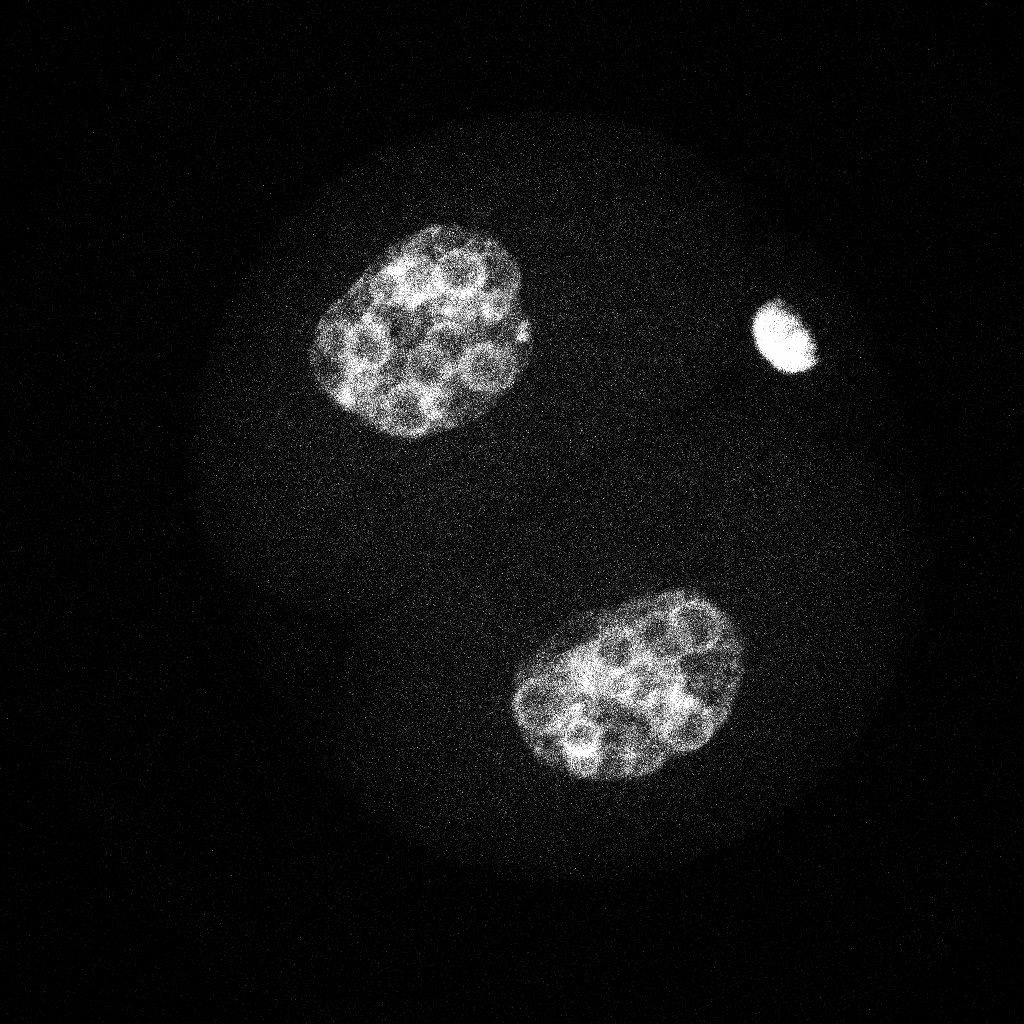

Supplement: Supplementary file 17 — Appendix Figure Source Data [file 44318_2024_329_MOESM17_ESM.zip › SD Appendix/FigS4B/S4B/Late2C_6h_DAPI.jpg]

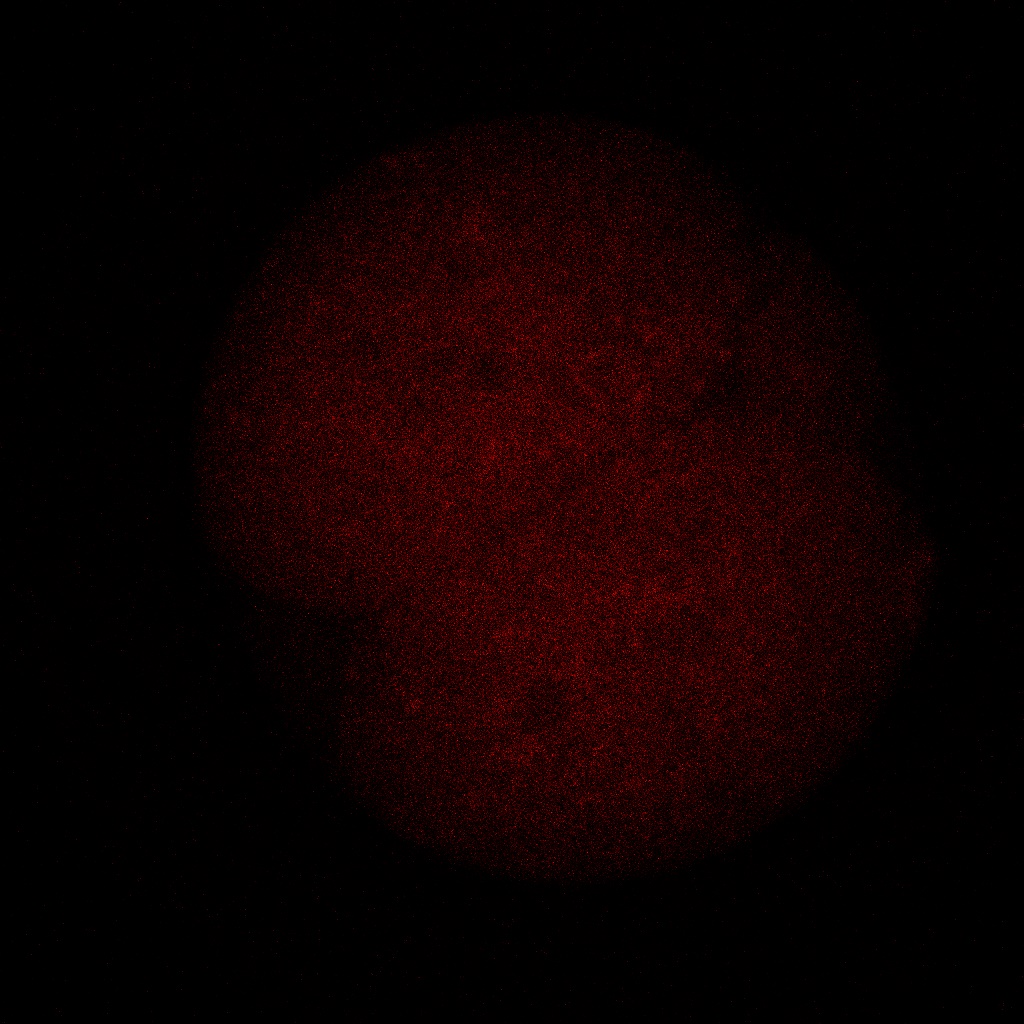

Supplement: Supplementary file 17 — Appendix Figure Source Data [file 44318_2024_329_MOESM17_ESM.zip › SD Appendix/FigS4B/S4B/Late2C_6h_EU.jpg]

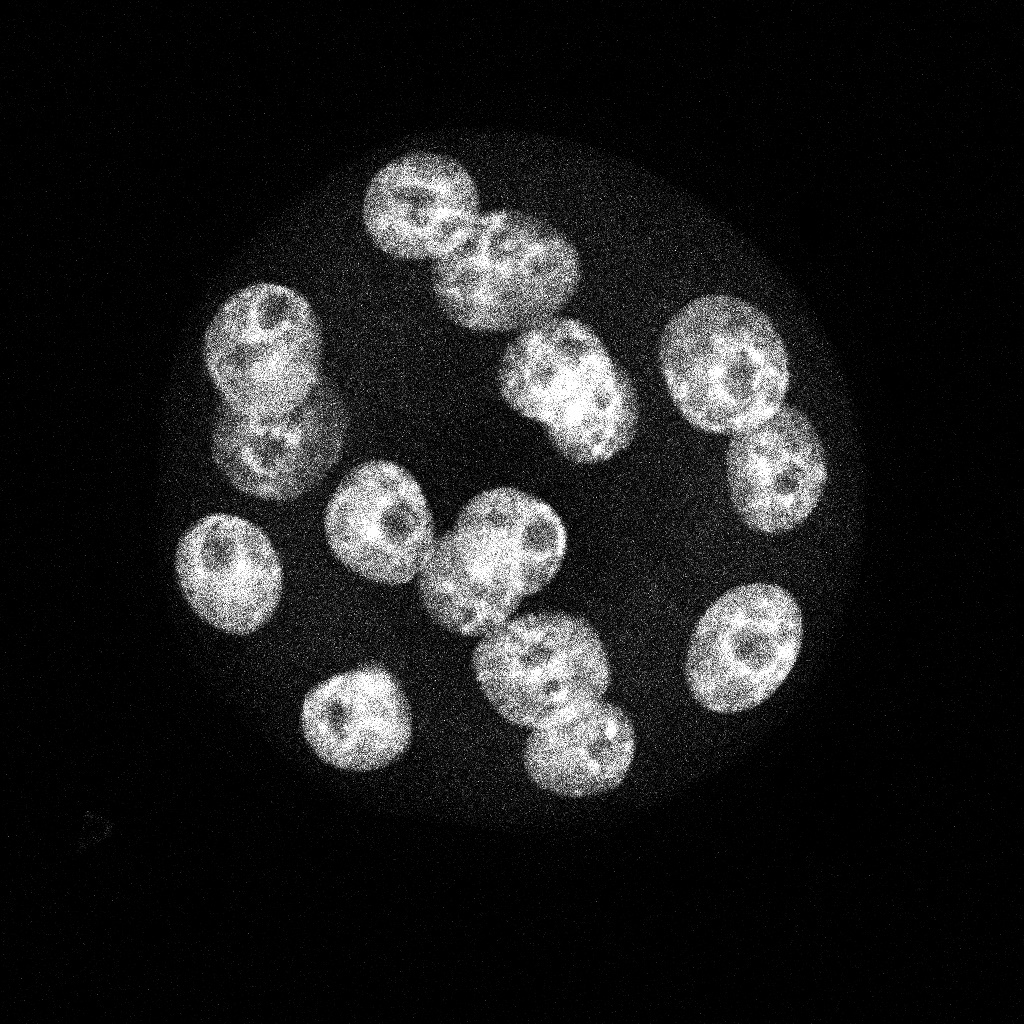

Supplement: Supplementary file 17 — Appendix Figure Source Data [file 44318_2024_329_MOESM17_ESM.zip › SD Appendix/FigS4C/S4C/Morula_0h_DAPI.jpg]

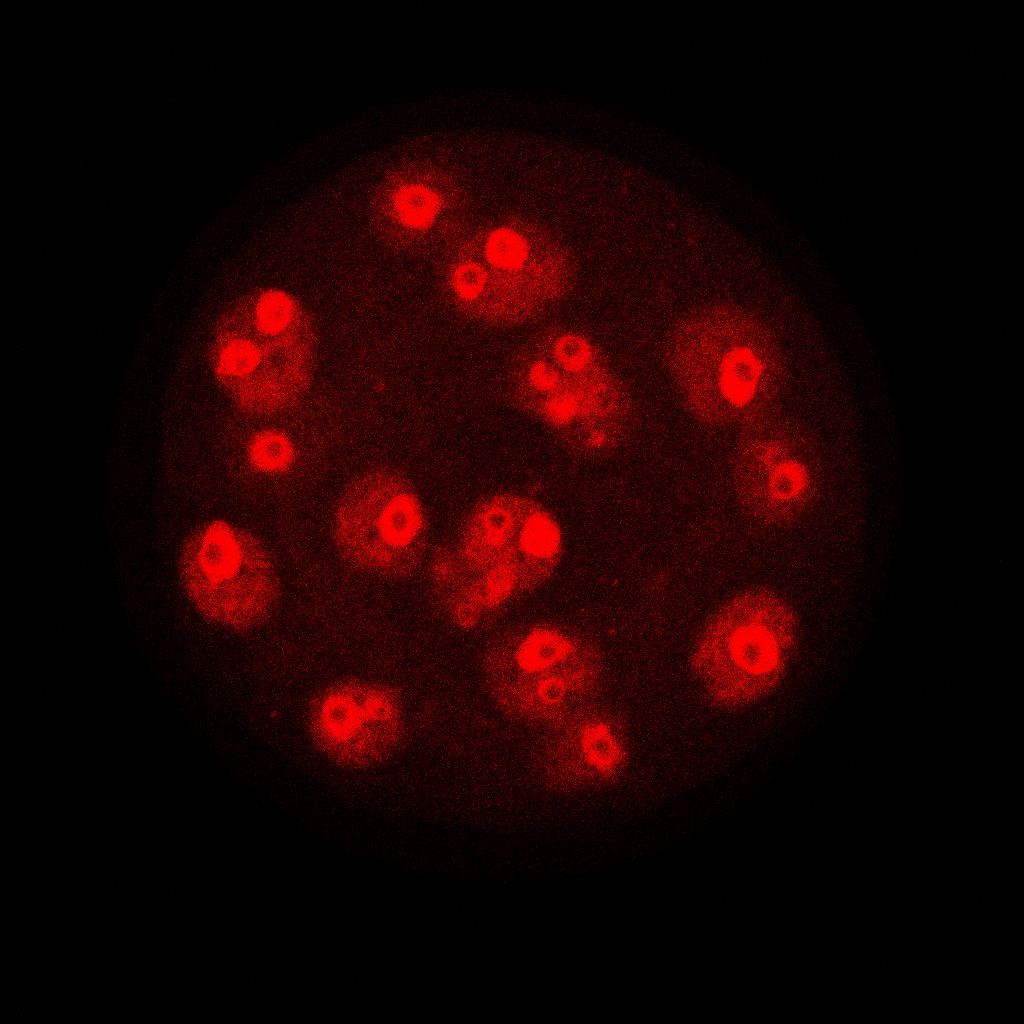

Supplement: Supplementary file 17 — Appendix Figure Source Data [file 44318_2024_329_MOESM17_ESM.zip › SD Appendix/FigS4C/S4C/Morula_0h_EU.jpg]

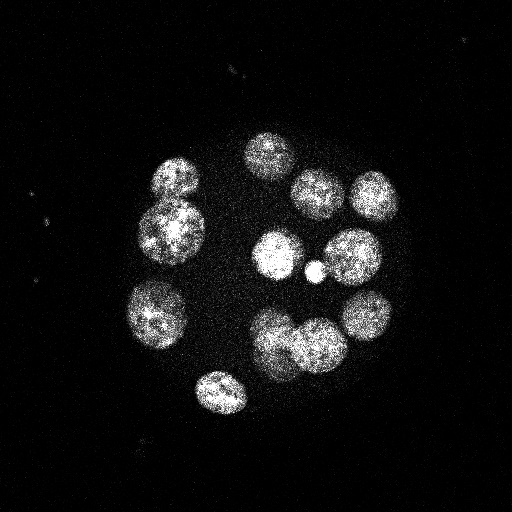

Supplement: Supplementary file 17 — Appendix Figure Source Data [file 44318_2024_329_MOESM17_ESM.zip › SD Appendix/FigS4C/S4C/Morula_2h_DAPI.jpg]

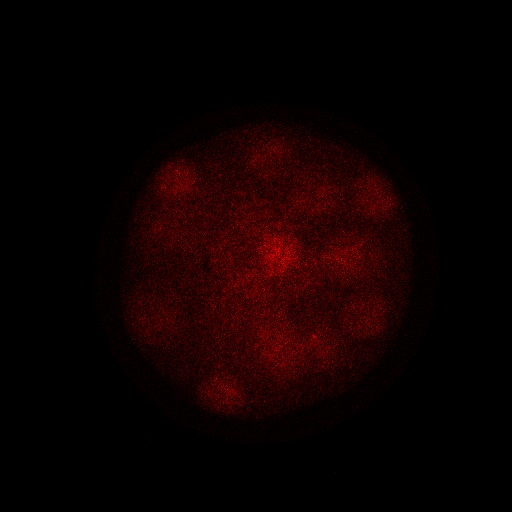

Supplement: Supplementary file 17 — Appendix Figure Source Data [file 44318_2024_329_MOESM17_ESM.zip › SD Appendix/FigS4C/S4C/Morula_2h_EU.jpg]

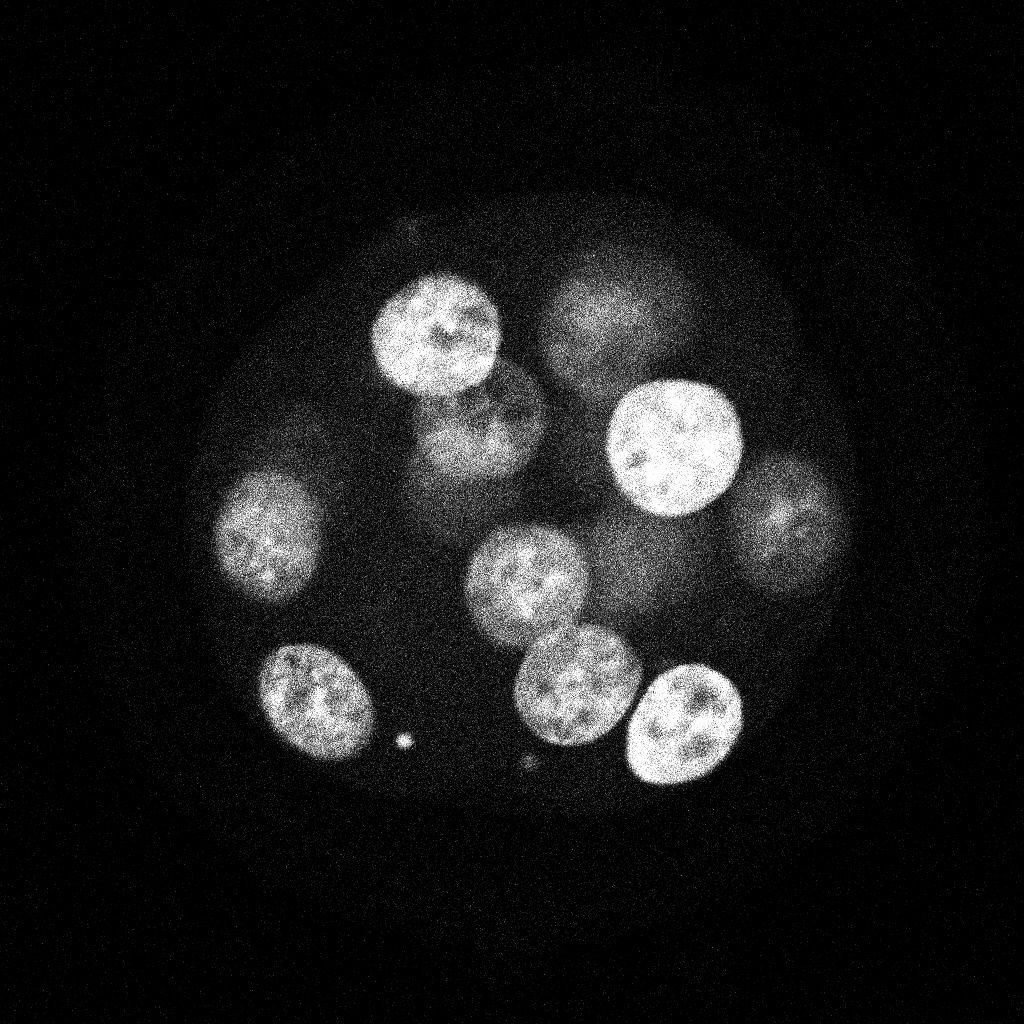

Supplement: Supplementary file 17 — Appendix Figure Source Data [file 44318_2024_329_MOESM17_ESM.zip › SD Appendix/FigS4C/S4C/Morula_4h_DAPI.jpg]

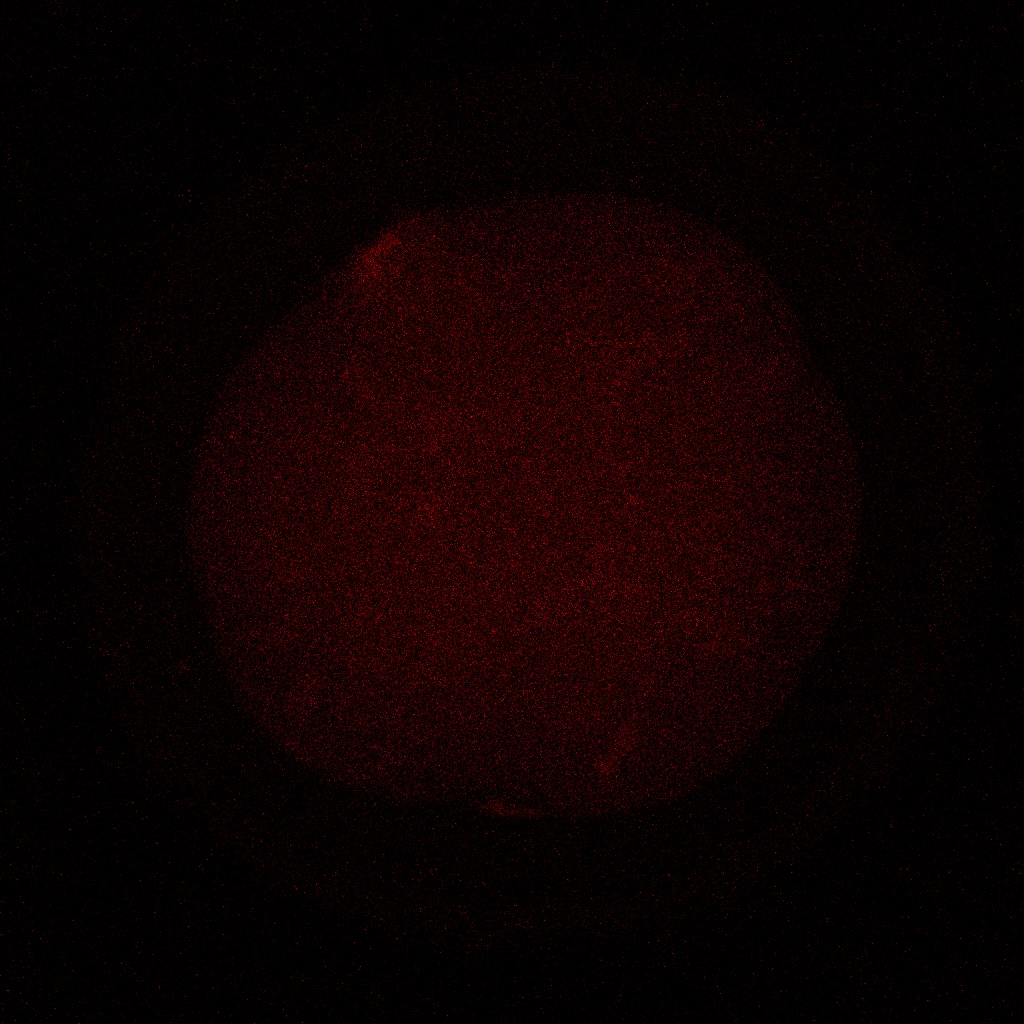

Supplement: Supplementary file 17 — Appendix Figure Source Data [file 44318_2024_329_MOESM17_ESM.zip › SD Appendix/FigS4C/S4C/Morula_4h_EU.jpg]

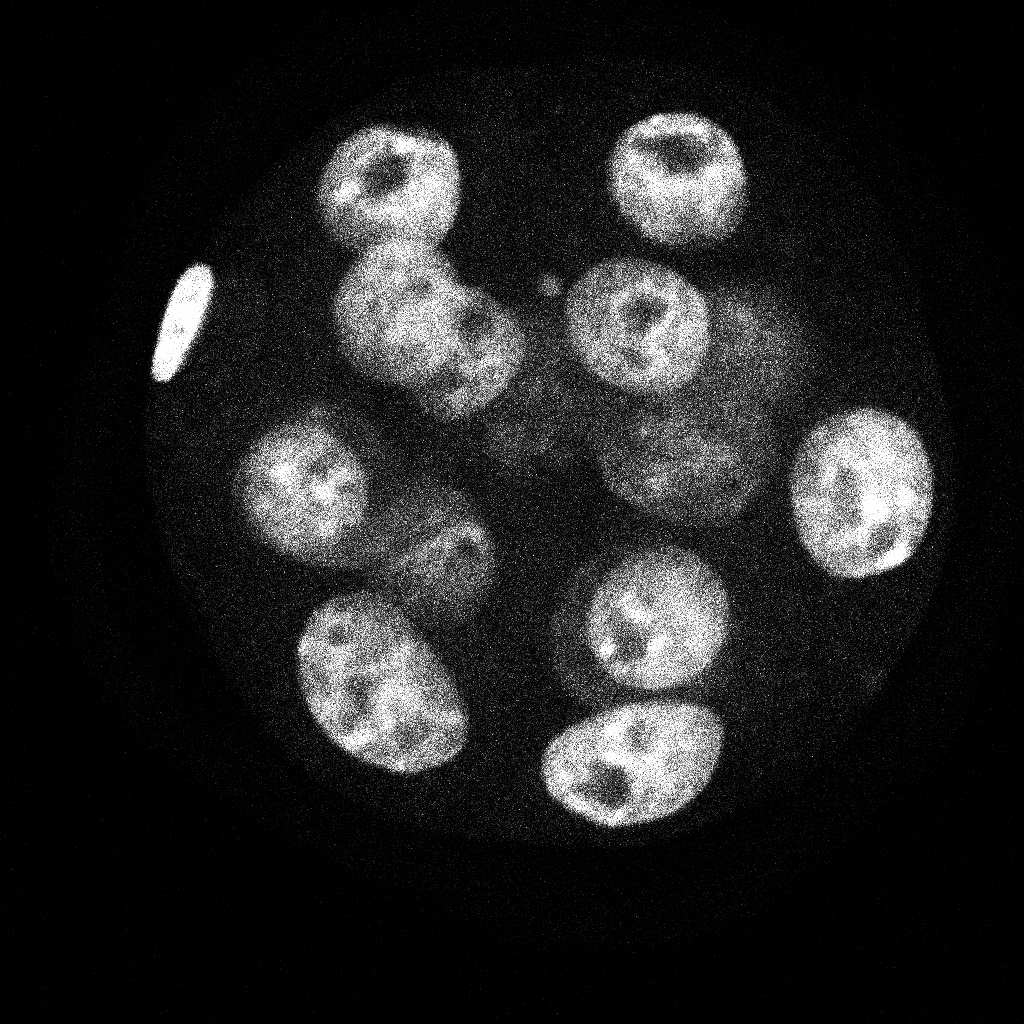

Supplement: Supplementary file 17 — Appendix Figure Source Data [file 44318_2024_329_MOESM17_ESM.zip › SD Appendix/FigS4C/S4C/Morula_6h_DAPI.jpg]

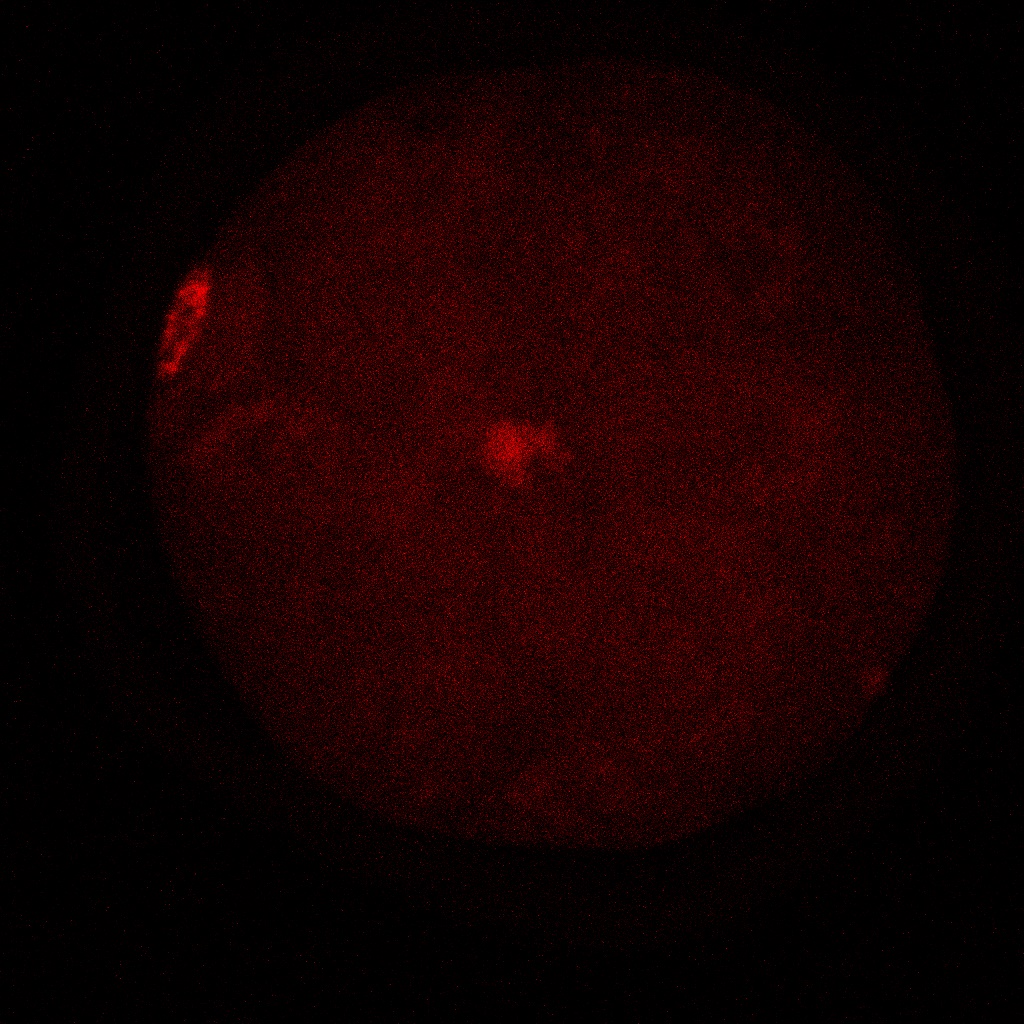

Supplement: Supplementary file 17 — Appendix Figure Source Data [file 44318_2024_329_MOESM17_ESM.zip › SD Appendix/FigS4C/S4C/Morula_6h_EU.jpg]

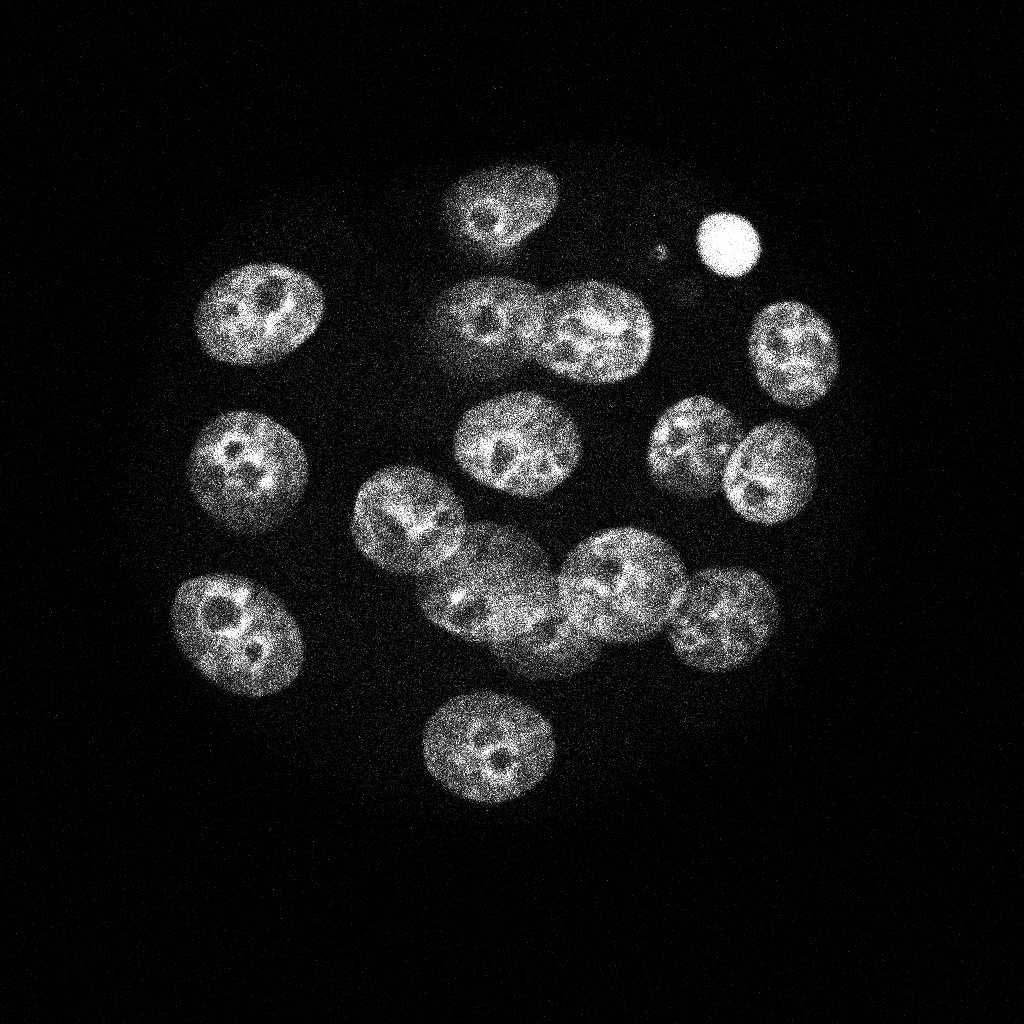

Supplement: Supplementary file 17 — Appendix Figure Source Data [file 44318_2024_329_MOESM17_ESM.zip › SD Appendix/FigS5F/S5F/Morula_0h_DAPI.jpg]

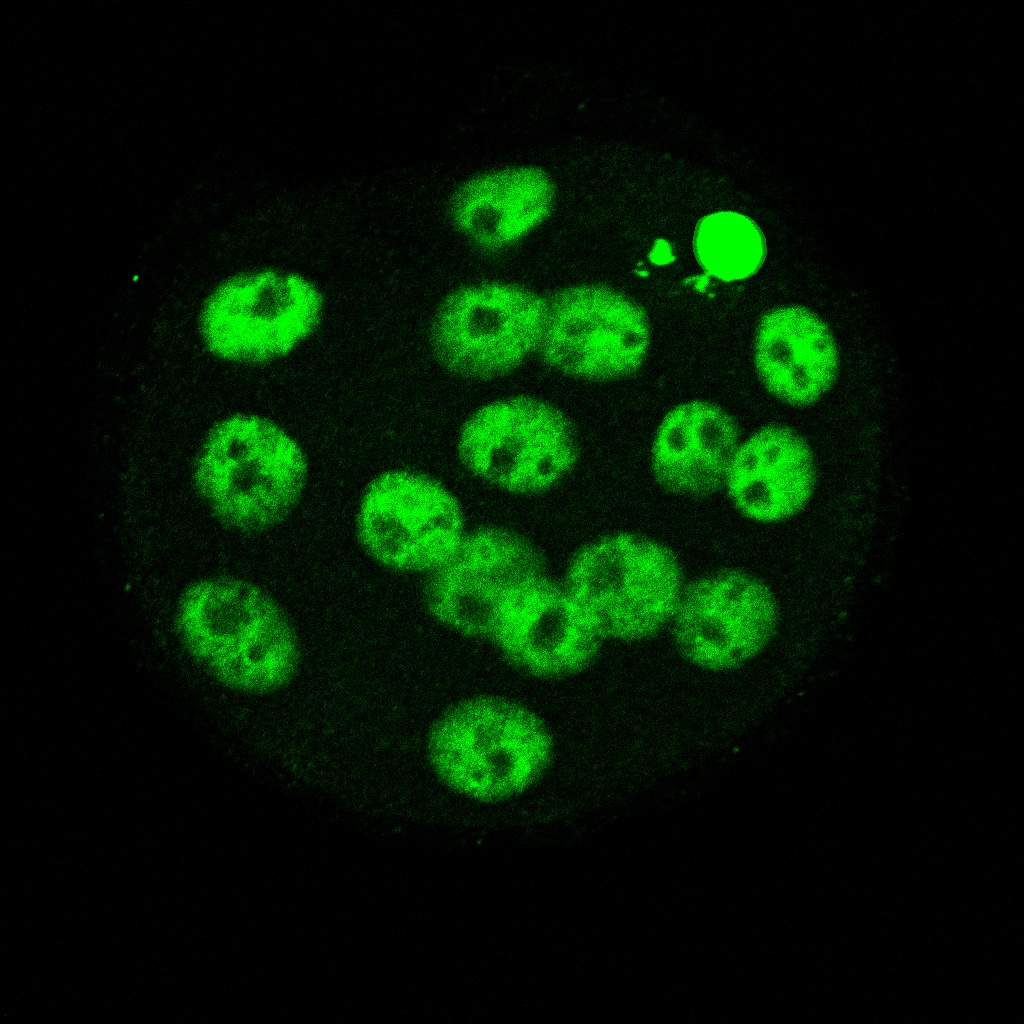

Supplement: Supplementary file 17 — Appendix Figure Source Data [file 44318_2024_329_MOESM17_ESM.zip › SD Appendix/FigS5F/S5F/Morula_0h_H3K4me3.jpg]

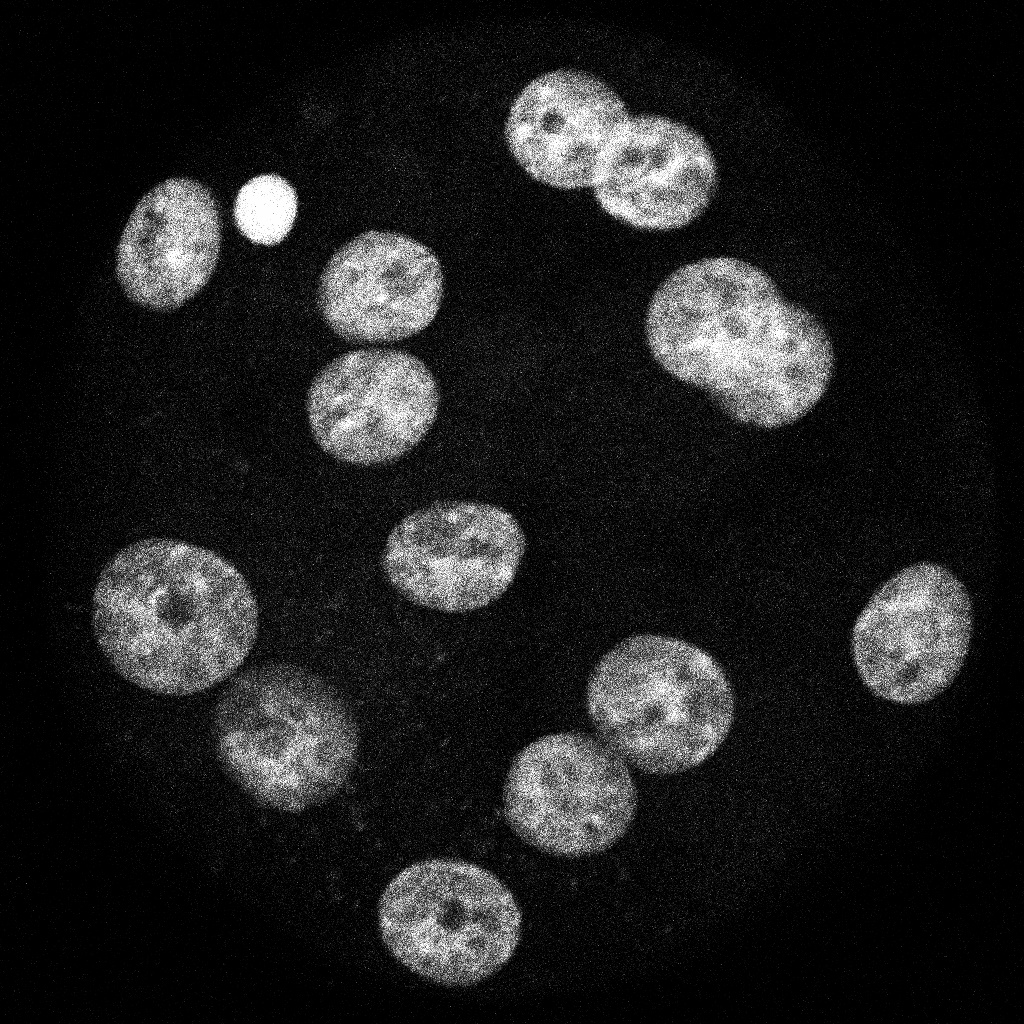

Supplement: Supplementary file 17 — Appendix Figure Source Data [file 44318_2024_329_MOESM17_ESM.zip › SD Appendix/FigS5F/S5F/Morula_Trp+CPI 2h_DAPI.jpg]

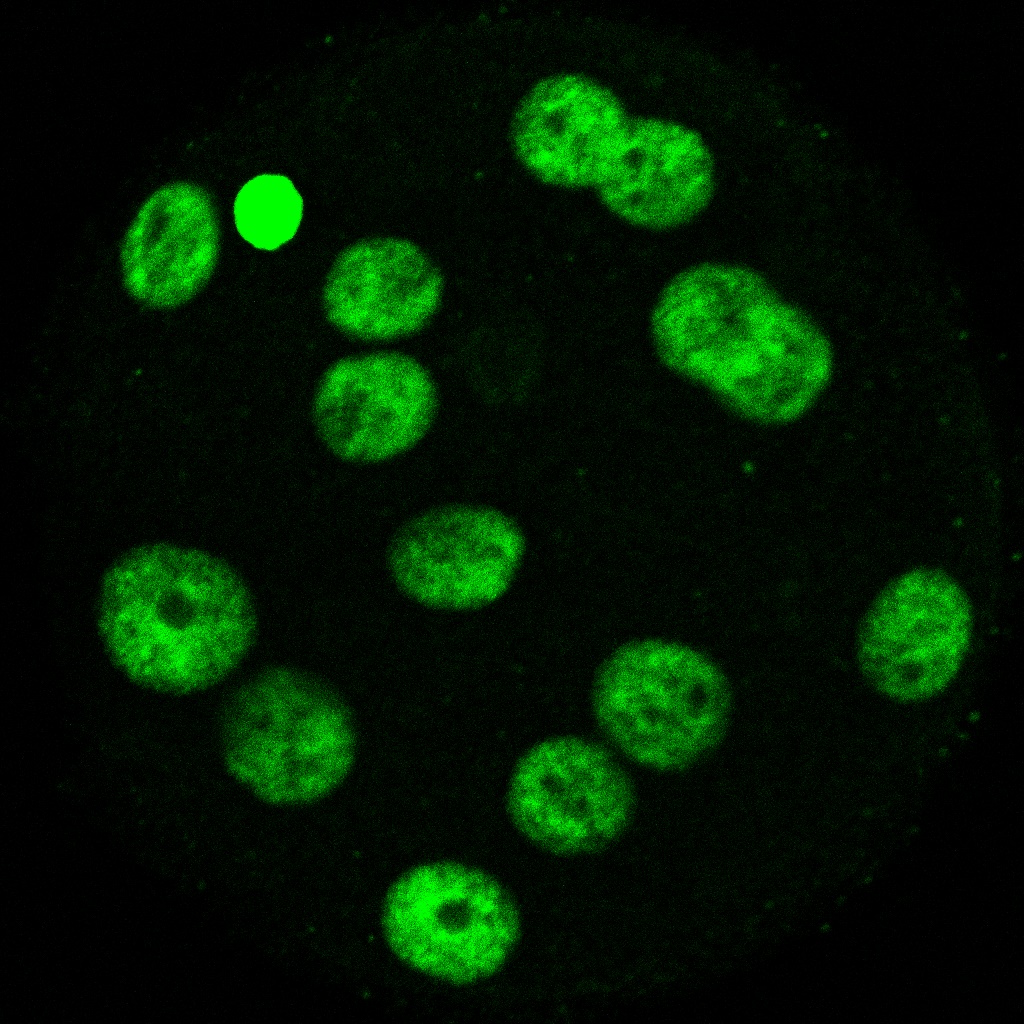

Supplement: Supplementary file 17 — Appendix Figure Source Data [file 44318_2024_329_MOESM17_ESM.zip › SD Appendix/FigS5F/S5F/Morula_Trp+CPI 2h_H3K4me3.jpg]
